# Synthesis of substituted triazole–pyrazole hybrids using triazenylpyrazole precursors

**DOI:** 10.3762/bjoc.20.121

**Published:** 2024-06-20

**Authors:** Simone Gräßle, Laura Holzhauer, Nicolai Wippert, Olaf Fuhr, Martin Nieger, Nicole Jung, Stefan Bräse

**Affiliations:** 1 Institute of Biological and Chemical Systems, Karlsruhe Institute of Technology, Kaiserstraße 12, 76131 Karlsruhe, Germany; 2 Institute of Nanotechnology, Karlsruhe Institute of Technology, Kaiserstraße 12, 76131 Karlsruhe, Germany; 3 Karlsruhe Nano Micro Facility (KNMFi), Karlsruhe Institute of Technology, Kaiserstraße 12, 76131 Karlsruhe, Germany; 4 Department of Chemistry, University of Helsinki, P.O. Box 55 (A. I. Virtasen aukio 1), 00014 Helsinki, Finland; 5 Institute of Organic Chemistry, Karlsruhe Institute of Technology, Kaiserstraße 12, 76131 Karlsruhe, Germany

**Keywords:** azide, click reaction, CuAAC, pyrazole, triazene, triazole

## Abstract

A synthesis route to access triazole–pyrazole hybrids via triazenylpyrazoles was developed. Contrary to existing methods, this route allows the facile *N*-functionalization of the pyrazole before the attachment of the triazole unit via a copper-catalyzed azide–alkyne cycloaddition. The developed methodology was used to synthesize a library of over fifty new multi-substituted pyrazole–triazole hybrids. We also demonstrate a one-pot strategy that renders the isolation of potentially hazardous azides obsolete. In addition, the compatibility of the method with solid-phase synthesis is shown exemplarily.

## Introduction

**Figure 1:**
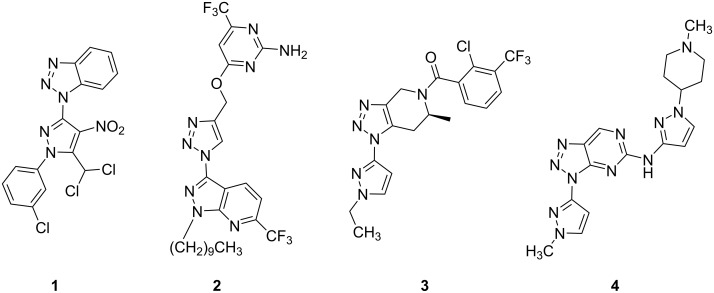
Biologically active pyrazole–triazole hybrids **1**–**4**: inhibitory effect on cholera bacteria [13], antimicrobial properties [14], P2X7 antagonists (depression) [15] and ERK3 inhibition [12].

Nitrogen-containing heterocycles are central scaffolds in medicinal chemistry and are incorporated in most small-molecule drugs [1–2]. We are interested in feasible strategies to synthesize nitrogen-rich heterocyclic scaffolds that can extend the currently available libraries with new drug-like molecules. Our past work on pyrazoles [3–6] and triazoles [7–11] motivated us to search for suitable and versatile strategies to explore access to triazole–pyrazole hybrids. Triazole–pyrazole hybrids, particularly non-fused heterocycles of this class, have not been investigated systematically. Selected known derivatives (Figure 1, **1**–**4**) inhibit the serine-threonine kinase ERK3 [12] or the cholera-causing bacterium *Vibrio cholerae* [13], show antimicrobial properties [14], and can act as P2X7 antagonists, a receptor involved in neuroinflammation and depression [15].

**Scheme 1:**
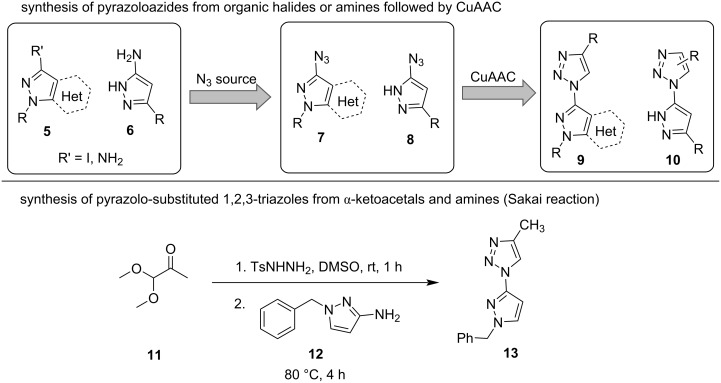
Literature-reported synthetic routes to pyrazole–triazole hybrids: synthesis of azides **7** or **8** from amines and organohalides and subsequent CuAAC to larger heterocyclic systems **9** or non-substituted amine products **10**; Sakai reaction of α-ketoacetal **11** for the synthesis of *N*-substituted derivative **13** [14,16–19].

Pyrazolyltriazoles are most easily obtained via the copper-catalyzed azide–alkyne cycloaddition (CuAAC) from pyrazolyl azides (**7** and **8**). These are usually accessed from the respective amines or organohalides (**5** and **6**, Scheme 1) [14,16–18]. Few examples of triazole–pyrazole hybrids, such as **13**, have also been synthesized through a modified Sakai reaction [19], a reaction cascade involving the elimination of an azole [20] or in the *n*-butyllithium-mediated reaction with alkyl halides [21]. So far, the literature-reported methods are most often limited to *N*-unsubstituted pyrazoles or triazoles and pyrazoles being fused to a second (hetero)cycle; the synthesis of promising multi-substituted structures such as **1** has not yet been described systematically.

## Results and Discussion

Triazenes have previously been established as versatile intermediates and linkers for conventional and solid-phase synthesis [22–25] that can be considered as protected diazonium salts [3]. According to the previous work [3], triazenylpyrazoles could serve as azide sources and thus as building blocks for synthesizing pyrazolyltriazoles by CuAAC reactions. To find a feasible approach to pyrazolyltriazoles of type **1** with a highly substituted scaffold, we decided to explore the benefits of a modification of the triazene-protected pyrazole core. In the next step, a cycloaddition of the gained synthesized azidopyrazoles with different alkynes was to be conducted.

The 3-(3,3-diisopropyltriaz-1-en-1-yl)-1*H*-pyrazole precursors **15a**–**d** were synthesized according to previously reported procedures [3,26–27] via the generation of a diazonium salt from aminopyrazoles **14a**–**d** followed by the addition of diisopropylamine, either in a one-pot synthesis or in two consecutive steps (Table 1). Subsequently, different aliphatic and aromatic substituents were attached to the pyrazole nitrogen by nucleophilic substitution with suitable organohalides **16** and cesium carbonate [3]. Due to the pyrazole tautomerism [28], the formation of two possible regioisomers, **17** and **18**, was anticipated and could be confirmed experimentally. Depending on the employed halide **16**, the distribution of the obtained products varied. A considerable excess of the dominating isomer with yields of up to 70% could be obtained in some cases (see **17f** or **17m**), whereas the isomers were isolated in a 1:1 ratio for compounds **17c** or **17h**. A strong trend towards regioisomer **17** as the main product was observed for substituted phenyl residues, presumably due to the higher steric hindrance (see **17e**–**g**). The results for benzylic residues differed depending on the benzylic residue's functional groups and the pyrazole substitution pattern. For starting materials **15a** and **15d**, an excess of product **17** was usually observed. With the ester-functionalized triazene **15c** and *m*-substituted benzylic reagents, regioisomer **18** was the predominant product (see **18i** and **18j**). In total, 13 groups could be attached to the different triazenylpyrazoles, yielding 18 products (see Table 1).

**Table 1:** Synthesis of triazenylpyrazoles **15a**–**d** and functionalization to *N*-substituted triazenylpyrazoles **17a**–**r** and **18a**–**r**. Conditions i: 1) BF_3_·OEt_2_, isoamyl nitrite, THF, −20 °C, 1 h, 2) diisopropylamine, THF/pyridine/acetonitrile, −20 °C to 21 °C, 17 h; Conditions ii: 1) HCl_aq_ (6 M), NaNO_2_, 0 °C to 5 °C, 1–2 h, 2) diisopropylamine, 0 °C to 21 °C, 16 h. X = F, Br, I.

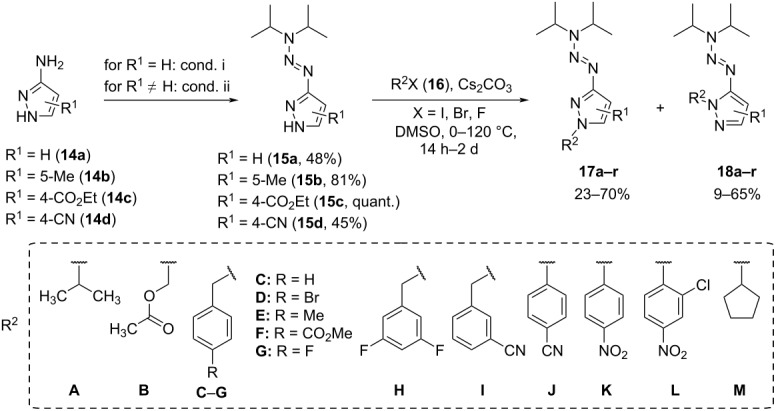					

R^1^	R^2^	Compound	Yield **17** [%]	Compound	Yield **18** [%]

H	**C**	**17a**	59	**18a**	18
H	**D**	**17b**	34	**18b**	28
H	**M**	**17c**	40	**18c**	40
5-Me	**C**	**17d**	23	**18d**	29
5-Me	**J**	**17e**	58	**18e**	12
5-Me	**K**	**17f**	64	**18f**	19
5-Me	**L**	**17g**	61	**18g**	9
4-CO_2_Et	**C**	**17h**	44	**18h**	40
4-CO_2_Et	**H**	**17i**	37	**18i**	63
4-CO_2_Et	**I**	**17j**	35	**18j**	65
4-CO_2_Et	**D**	**17k**	41	**18k**	59
4-CN	**A**	**17l**	35	**18l**	58
4-CN	**B**	**17m**	70	**18m**	22
4-CN	**C**	**17n**	58	**18n**	39
4-CN	**H**	**17o**	51	**18o**	46
4-CN	**E**	**17p**	59	**18p**	40
4-CN	**F**	**17q**	53	**18q**	45
4-CN	**G**	**17r**	54	**18r**	41

**Scheme 2:**
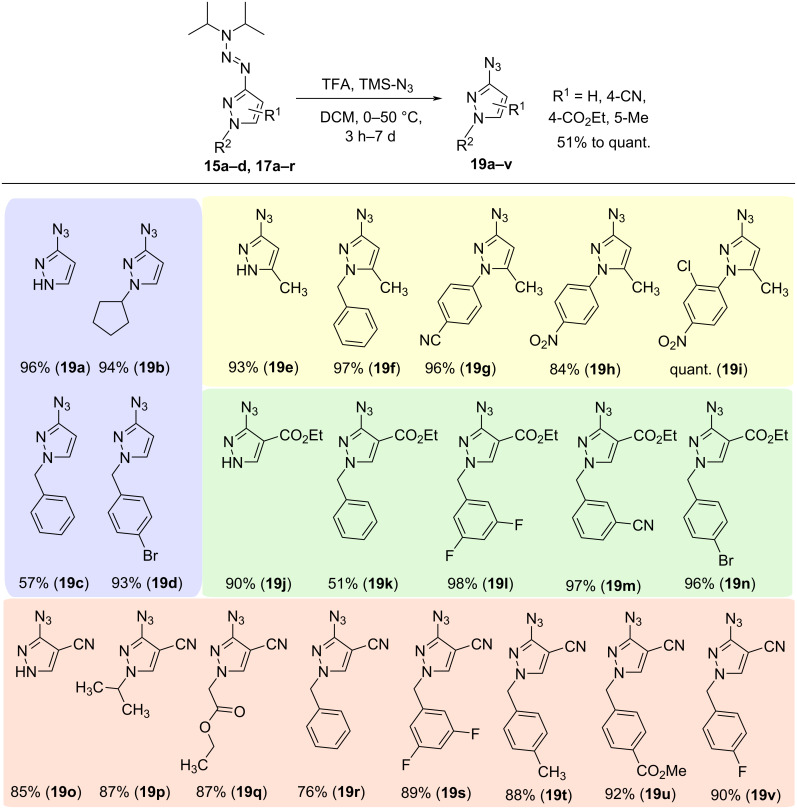
Synthesis of pyrazolyl azides **19a**–**v** via cleavage of the protecting triazene moiety. For compounds **19a**–**h**, **19p**, **19r**, **19t** and **19u**, reaction times of 3–16 h were suitable. The reactions to **19o** and **19v** were stirred for 3 d; for **19q** and **19s**, reaction times of 7 d were necessary.

In analogy to reported procedures for cleavage of polymer-bound triazenes [23], we attempted to develop the first protocol for synthesizing pyrazolyl azides **19** from triazenylpyrazoles. Initial experiments with TFA and trimethylsilyl azide at 0–25 °C in DCM failed for 4-substituted pyrazoles; the formation of the target products was only observed when 5-methylpyrazoles such as **15b** were used. Therefore, a modified procedure was applied, heating the triazenes to 50 °C. This optimization allowed for the isolation of the corresponding azides **19a–v** in yields of 51% to quantitative (Scheme 2), usually with durations of 3–16 h. Longer reaction times were necessary for some CN-substituted triazene derivatives (**19o**, **19q**, **19s**, **19v**), especially in combination with electron-withdrawing functional groups. The developed procedure could only be used to convert isomer **17**. Triazene compounds with the regioisomeric form **18** could not be reacted (see Supporting Information File 1, Scheme S1) even after extended reaction times, only starting material was reisolated, presumably due to the increased stability of isomer **18** towards acids. This corresponds with the results for the previously reported triazene cleavage to diazonium intermediates and subsequent cyclization to triazine derivatives [3].

**Scheme 3:**
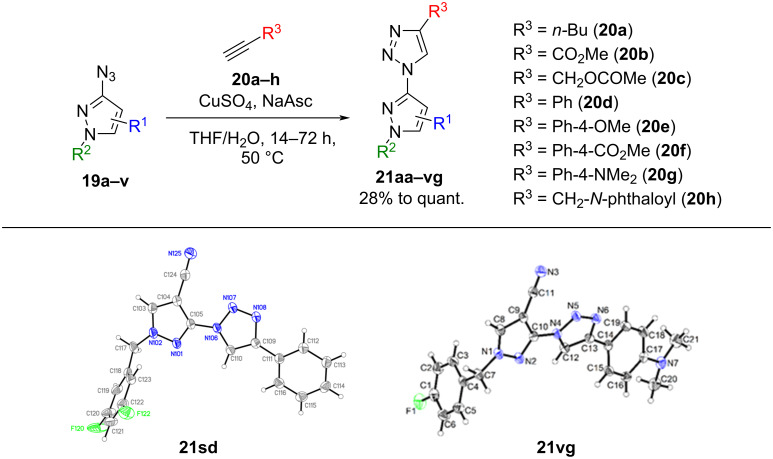
Synthesis of pyrazole–triazole hybrids via CuAAC and ORTEP diagrams of triazole products **21sd** and **21vg** with the thermal ellipsoids shown at 50% probability. NaAsc = sodium ascorbate.

**Figure 2:**
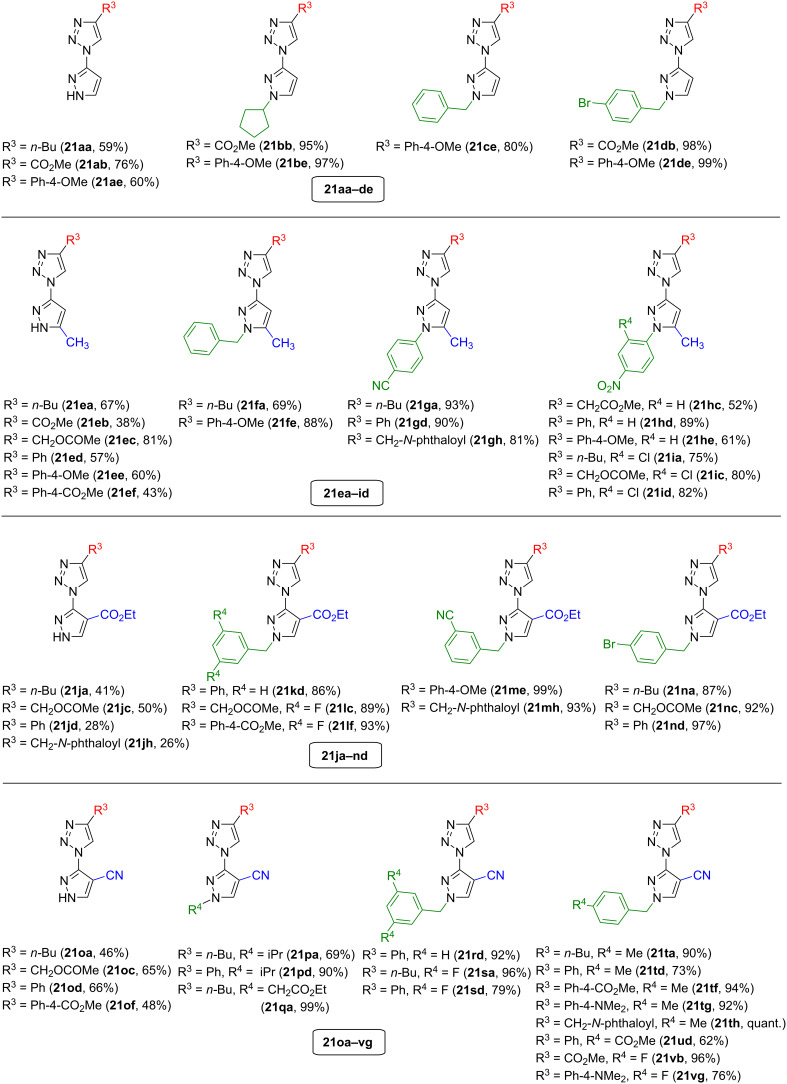
Synthesized triazole–pyrazole hybrids **21aa**–**vg**.

In the next step, the obtained pyrazolyl azides were reacted with different aromatic and aliphatic alkynes **20a**–**h** in a copper-catalyzed azide–alkyne cycloaddition (CuAAC). All attempted reactions could be conducted under standard conditions using copper sulfate and sodium ascorbate in THF/water (depicted in Scheme 3 and Figure 2). For selected derivatives, **21sd** and **21vg**, crystals suitable for single-crystal X-ray diffraction could be obtained and confirmed the product structure with the presumed regioisomer (Scheme 3).

A library of over 50 triazole products **21aa**–**vg** was successfully synthesized with yields ranging from 28% to quantitative, combining four different pyrazole-carbon substitutions and 14 pyrazole-nitrogen substitutions with eight different residues on the to-be-formed triazole (see Figure 2). It could be observed that the cycloaddition proceeds least efficiently with pyrazoles that are not substituted on the nitrogen. The reaction of pyrazolyl azides **19e** and **19j** with phenylacetylene gave the products **21ed** and **21jd** with yields of 57% and 28%, whereas substituted derivatives (e.g., **19g** or **19n**) resulted in yields of over 90% (**21gd** or **21nd**) using the same alkyne. The different substitution patterns on the 4- or 5-position of the pyrazole (R^1^) do not clearly influence the reaction's efficiency. Although the reactions of ethyl 3-azido-1*H*-pyrazole-4-carboxylate (**19j**) resulted in lower yields of the triazole products **21ja**–**jh** compared to pyrazolyl azides **19a**, **19e**, and **19o**, this trend is not continued in the results of the *N*-substituted carboxylate derivatives **19k**–**n**. The effect of the alkyne depends on the substitutions on the pyrazole, and no general trend is visible – reactions with electron-poor, electron-rich as well as sterically demanding alkynes give high product yields, depending on the respective pyrazole. The functionalization of the NH-unsubstituted derivative **21jd** via copper-catalyzed cross-coupling [29] with an electron-rich aryl substituent was exemplarily conducted to further expand the scope of possible products (see Scheme S2, Supporting Information File 1).

**Scheme 4:**
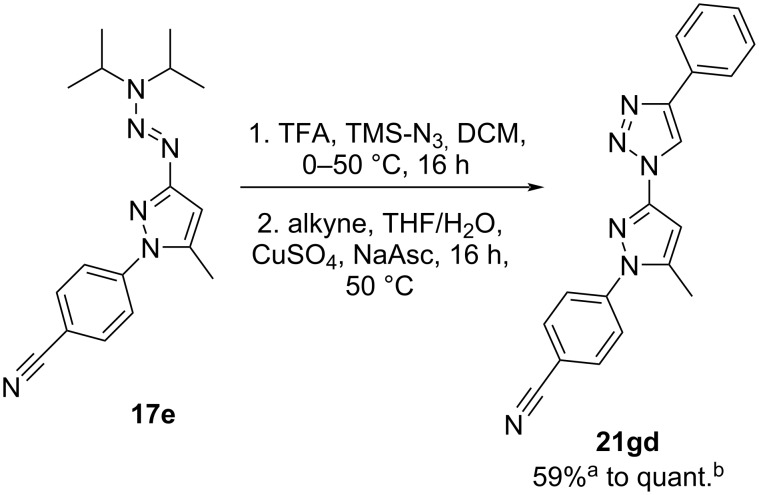
One-pot synthesis of triazole–pyrazole hybrid **21gd**. ^a^One-pot setup yielded **21gd** with unknown impurities; ^b^with an additional evaporation step after the triazene cleavage, a quantitative yield of the target product **21gd** was achieved.

We also investigated the scope and limitations of a one-pot reaction for the triazene cleavage and subsequent CuAAC with the model compound **17e** (see Scheme 4). When conducting the two reaction steps back-to-back in a one-pot setup, a decrease in yield from 86% over two steps (96% and 90%) to 59% of impure product was observed. This is presumably caused by incomplete conversion of the in situ-generated alkyne to the triazole. The decrease of TFA/TMS-N_3_ in the reaction or the addition of an increased amount of alkyne further deteriorated the results. Therefore, we introduced a straightforward evaporation step after completion of the triazene cleavage to remove the residual reagents. The final product **21gd** could be isolated in quantitative yield with this technique, avoiding additional purification steps for the azide intermediate without any losses in product formation.

**Scheme 5:**
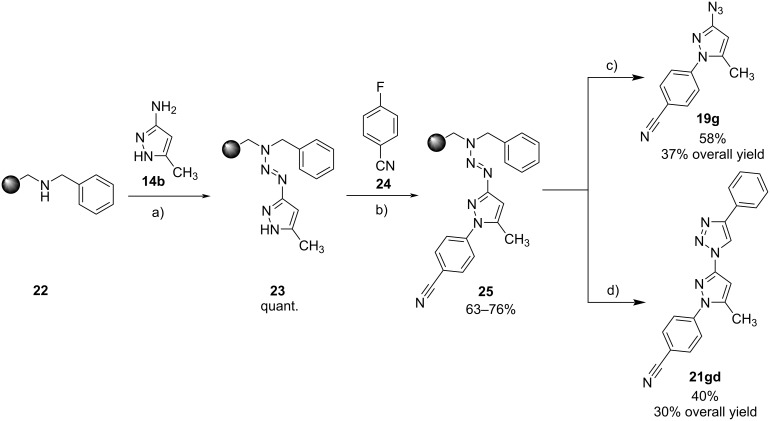
Solid-phase synthesis of azidopyrazole **19g** and triazole–pyrazole hybrid **21gd** by immobilization of aminopyrazole **14b** on benzylamine-substituted Merrifield resin **22**, NH-functionalization and cleavage. Reaction conditions: a) BF_3_∙Et_2_O, isoamyl nitrite, THF/pyridine (9:1), −20 °C to 21 °C, 12 h, b) Cs_2_CO_3_, DMSO, 120 °C, 2–3 d, c) TFA, TMS-N_3_, DCM, 25 °C, 12 h, d) 1. TFA, TMS-N_3_, DCM, 0–50 °C, 12 h; 2. alkyne, THF/H_2_O, CuSO_4_, sodium ascorbate, 16 h, 50 °C.

The developed procedure was exemplarily transferred to solid-phase synthesis. In quantitative yields, 5-methyl-1*H*-pyrazol-3-amine (**14b**) was immobilized on benzylamine resin **22** (Scheme 5). For this purpose, a diazonium intermediate was generated from the pyrazoloamine with BF_3_∙Et_2_O and isoamyl nitrite accordingly to the liquid phase synthesis of **15b**. The subsequent functionalization of resin **23** to the phenyl-substituted derivative **25** was carried out using the nucleophilic substitution procedure reported above with yields of 63–76%. The anticipated formation of a second regioisomer could not be confirmed due to the limited analytical methods available for compounds on solid supports. The cleavage to obtain azidopyrazole **19g** was achieved with a total yield of 37% over all steps, comparable to the total yield of 45% for the stepwise synthesis in the liquid phase. This indicates a material loss due to the non-reactive regioisomer formation in the previous step and a non-quantitative cleavage process. In analogy to the one-pot experiments in solution, a one-pot cleavage from the resin combined with the CuAAC reaction to the triazole–pyrazole hybrid was conducted exemplarily and gave the target product **21gd** in 30% yield.

The solid-phase reaction route allows for roughly equally high overall yields compared to the solution synthesis. It offers the additional benefits of chemistry on solid support: straightforward purification of the resin-bound intermediates by washing steps and a high throughput that allows for faster derivatization. Further research is necessary to establish a protocol for the cleavage of 4-substituted pyrazoles, as the corresponding azides analogous to **19j**–**v** could not be obtained from immobilized triazene precursors.

## Conclusion

A synthesis route to access substituted triazole–pyrazole hybrids from triazenylpyrazoles has been established and applied to obtain a library of over 50 new triazole–pyrazole hybrids. The selective *N*-functionalization of the triazene-protected pyrazoles was conducted, and the cleavage of triazenylpyrazoles to the corresponding azides was described for the first time with regioisomer **17**, whereas regioisomer **18** is acid-insensitive and cannot be converted. The azides were reacted to the respective triazole product in a CuAAC reaction; this step could also successfully be conducted in a sequential one-pot approach from the triazenylpyrazole precursor. The developed protocol was adapted for solid-phase synthesis to demonstrate the applicability of triazenylpyrazoles as immobilized building blocks.

## Supporting Information

The Supporting Information covers detailed material on the conducted experiments and their results. All experimental details, including the analytical description of the obtained target compounds and byproducts, are available in the Supporting Information. Data that refers to the experiments described herein were submitted to the repository chemotion (https://www.chemotion-repository.net/). All DOIs minted for the data are linked in Supporting Information File 1 and the NMR spectra are given in Supporting Information File 2. Information on the availability of the data and the physical material of the target compounds is added to Supporting Information File 3. New data obtained in this study is assigned to the collection embargo number SGV_2021-06-02 (https://dx.doi.org/10.14272/collection/SGV_2021-06-02) [30]. The material obtained in this study was submitted to the Molecule Archive at KIT and can be requested from there (https://compound-platform.eu/home). CCDC 2308695 (**18n**), 2308696 (**21vg**) and 2309318 (**21sd**) contain the supplementary crystallographic data for this paper. These data can be obtained free of charge from The Cambridge Crystallographic Data Centre via https://www.ccdc.cam.ac.uk/data_request/cif.

File 1 Experimental part.

File 2 NMR spectra.

File 3 Information on the availability of the data and the physical material of the target compounds.

## Data Availability

The data generated and analyzed during this study is openly available in the Chemotion repository at https://doi.org/10.14272/collection/SGV_2021-06-02. Crystal structures are made available via the CCDC.
