# Supplementary material for: Synthesis of substituted triazole–pyrazole hybrids using triazenylpyrazole precursors
Source: Beilstein J Org Chem. 2024 Jun 20;20:1396–404. doi: 10.3762/bjoc.20.121 (PMC11196952; doi:10.3762/bjoc.20.121)
Supplement: File 1 — Experimental part. [file Beilstein_J_Org_Chem-20-1396-s001.pdf]

BEILSTEIN JOURNAL OF ORGANIC CHEMISTRY

Supporting Information

for

Synthesis of substituted triazole–pyrazole hybrids using triazenylpyrazole precursors

Simone Gräßle, Laura Holzhauer, Nicolai Wippert, Olaf Fuhr, Martin Nieger, Nicole Jung and Stefan Bräse

Beilstein J. Org. Chem. **2024,** *20,* 1396–1404. doi:10.3762/bjoc.20.121

Experimental part

License and Terms: This is a supporting information file under the terms of the Creative Commons Attribution License (https://creativecommons.org/licenses/by/4.0). Please note that the reuse, redistribution and reproduction in particular requires that the author(s) and source are credited and that individual graphics may be subject to special legal provisions.
The license is subject to the *Beilstein Journal of Organic Chemistry* terms and conditions: (https://www.beilstein-journals.org/bjoc/terms)

Contents

1. General Remarks S2

2. Synthetic Results S6

3. Crystallographic Results S126

4. References S129

1. General Remarks

The starting materials and reagents were purchased from ABCR, ACROS, ALFA AESAR, BLDPHARM, CHEMPUR, FLUKA, FLUOROCHEM, MERCK, SIGMA ALDRICH, STREM, TCI, or THERMO FISHER SCIENTIFIC and used without further purification unless stated otherwise. Solvents of technical grade were purified via distillation prior to use (ethyl acetate, dichloromethane, cyclohexane), solvents of *p.a.* quality were purchased from ACROS, FISHER SCIENTIFIC, SIGMA ALDRICH, ROTH, RIEDEL-DE HAËN or VWR and were used without further purification.

Air- and moisture-sensitive reactions were carried out under nitrogen or argon atmosphere in oven-dried glassware using standard Schlenk techniques.
Reactions in vials were sealed with Crimp caps; both vials and caps were purchased at CHROMA GLOBE.
Solvents were evaporated under reduced pressure at 40 °C using a rotary evaporator. For solvent mixtures, each solvent was measured volumetrically.

Flash Chromatography:
Purifications via flash chromatography were performed using silica gel (SiO_2, 0.040 mm × 0.063 mm, MERCK) and quartz sand (glowed and purified with hydrochlorid acid). After removing the solvent under reduced pressure, the crude products were immobilized on Celite (VWR) and applied to the column as a solid.

For automatic flash chromatography, an INTERCHIM PuriFLASH XS 400, INTERCHIM PuriFLASH 4125 or INTERCHIM PuriFLASH 5.125 was used in combination with hand-packed silica columns (SiO_2, 0.040 mm × 0.063 mm, MERCK) as well as prepacked SIHP (silica high performance, 15 µm, 4 g/12 g/40 g/80 g) columns from INTERCHIM. Fractions were separated and collected using a diode array detector (DAD).

Thin Layer Chromatography (TLC):

Reactions were monitored by thin-layer chromatography (TLC) using silica-coated aluminum plates (MERCK, silica gel 60, F_{254}). UV active compounds were detected with a UV-lamp (HANAU QUARZLAMPEN, Typ 204 AC) at 254 nm and 366 nm excitation. Moreover, Seebach solution (2.5% phosphomolybdic acid, 1.0% cerium(IV)sulfate tetrahydrate, 6.0% conc. H_2SO_4 in H_2O) with subsequent heating of the TLC plate was used to stain the spots.

Liquid Chromatography – Mass Spectrometry (LC-MS):

Liquid-Chromatography Mass Spectrometry (LC-MS) was conducted using a device from AGILENT with HP 1100 MSD G1946 Mass Detector and a Kinetex XB-C18 column (2.6 µm, 100 x 4.60 mm) from PHENOMENEX. API-ES was used as a method of ionization and the following program was applied:

10_99_P (positive polarity): injector volume 10.0 µl, flow rate 1.0 ml/min, run time 20.0 min,
solvent: water (bidistilled) 50%, acetonitrile 20%.

Melting Points:
Melting points were detected on an OptiMelt MPA100 device from STANFORD RESEARCH SYSTEM.

Nuclear Magnetic Resonance Spectroscopy (NMR):
A BRUKER Ascend 400 was used to record NMR spectra; ^{1}H NMR spectra were measured at 400 MHz, ^{13}C NMR spectra at 100 MHz and ^{19}F NMR spectra at 376 MHz. All measurements were conducted at room temperature using chloroform-*d* or DMSO-d_6 acquired from EURISOTOP and SIGMA ALDRICH as solvents. Chemical shifts are given in ppm (parts per million) and referenced in ^{1}H and ^{13}C spectra to the solvent signals of the respective deuterated solvent: Chloroform-*d* (^{1}H: 7.27 ppm, ^{13}C: 77.0 ppm or 77.16 ppm), DMSO-d_6 (^{1}H: 2.50 ppm, ^{13}C: 39.5 ppm).

^{1}H NMR spectra were analyzed according to first order. The signal area was given for multiplets, while the signal center was used for centrosymmetric signals. The signal splitting was characterized using the following abbreviations: s (singlet), d (doublet), t (triplet), q (quartet), m (multiplet), br. s (broad singlet), dd (doublet of the doublet), td (triplet of the doublet), ps (pseudo-singlet) etc. For ^{13}C spectra, the peaks were observed as singlet if not specifically stated otherwise. Coupling constants *J* are given in Hz (Hertz), and the number of bonds between the coupling cores is indicated as a superscripted index in front of the coupling constant. Signals of ^{13}C spectra were assigned using DEPT90 and DEPT135 spectra (distortionless enhanced polarization transfer) and/or HSQC (heteronuclear single quantum coherence) and HMBC (heteronuclear multiple bond correlation).

Infrared Spectroscopy (IR):
IR spectra were measured via ATR (Attenuated Total Reflection) on a BRUKER Alpha. The positions of the absorption bands are given in wavenumbers $\tilde{\nu}$ in cm^{-1} and were measured in the range from 3600 cm^{-1} to 500 cm^{-1}.

Characterization of the absorption bands was done in dependence of the absorption strength with the following abbreviations: vs (very strong, 0−9%), s (strong, 10−39%), m (medium, 40−69%), w (weak, 70−89%), vw (very weak, 90−100%).

Mass Spectrometry (MS):
EI-MS (electron ionization mass spectroscopy) and FAB-MS (fast atom bombardment mass spectrometry) were conducted on a FINNIGAN MAT 95 with 3-nitrobenzyl alcohol (3-NBA) as matrix and reference for high resolution. The intensity of the signals is given relative to the intensity of the highest peak (100%). For the interpretation of the spectra, molecular peaks $[M]^+$, peaks of protonated molecules $[M+H]^+$ and characteristic fragment peaks are indicated with their mass-to-charge ratio (m/z) and their intensity

in percent, relative to the base peak (100%) is given. In case of high-resolution measurements, the maximum tolerated error is ±5 ppm.

ESI-MS (electron spray ionization mass spectrometry) was conducted with a ThermoFisher Q Exactive Plus in positive mode with a voltage of 4kV. The tolerated error is ±5 ppm of the molecular mass. The spectra were interpreted by molecular peaks $[M]^+$, peaks of protonated molecules $[M+H]^+$ and characteristic fragment peaks and indicated with their mass-to-charge ratio (*m/z*).

Elemental Analysis (EA):

Elemental analysis was conducted using an Elementar vario Micro and a Sartorius M2P analytical balance. Calculated and found percentage for carbon (C), hydrogen (H), sulfur (S) and nitrogen (N) are indicated in fractions of 100%.

Single-Crystal X-Ray Diffraction (XRD):

Single crystal X-ray diffraction data for **18n** and **21vg** were collected on a STOE STADI VARI diffractometer with monochromated Ga Kα (1.34143 Å) radiation at low temperature. Using Olex2 [1], the structures were solved with the ShelXT [2] structure solution program using Intrinsic Phasing and refined with the ShelXL [3] refinement package using Least Squares minimization. Refinement was performed with anisotropic temperature factors for all non-hydrogen atoms; hydrogen atoms were calculated on idealized positions.

The single-crystal X-ray diffraction study of **21sd (**NiW-C1184.1; X12101 (sb1181_hy,) was carried out on a Bruker D8 Venture diffractometer with PhotonII detector at 123(2) K using Cu-Kα radiation (λ = 1.54178 Å). Dual space methods (SHELXT) [2] were used for structure solution and refinement was carried out using SHELXL-2014 (full-matrix least-squares on F^2). [3] Hydrogen atoms were refined using a riding model. A semi-empirical absorption correction and an extinction correction were applied.

CCDC 2308695 (SG-V3461-21-30 (**18n**)), 2308696 (SG-V3478-7-10 (**21vg**)) and 2309318 (NiW-C1184.1; X12101 (sb1181_hy, **21sd**)) contain the supplementary crystallographic data for this paper.
These data can be obtained free of charge from The Cambridge Crystallographic Data Centre via www.ccdc.cam.ac.uk/data_request/cif.

Crystal data and structure refinement details of **18n**, **21sd** and **21vg** are summarized in Table S1.

Solid-Phase Synthesis:

Resin reactions in Crimp vials were shaken on orbital shakers from IKA Labortechnik. A KS501 digital was used for reactions at elevated temperatures, a model KS250 basic was used for reactions at ambient temperature. For larger batches in round-bottom flasks, a New Brunswick Scientific Innova 42 incubator was used.

The yield for solid-phase reactions was calculated by dividing the experimentally determined mass m_{ex} through the theoretically calculated mass m_{theory} and multiplying the result with 100. The obtained value was multiplied with the used amount *n* in mmol and divided by the obtained mass of the product resin m_{ex} to obtain the loading of the resin.

$$Yield\ (\%) = \frac{m_{ex}}{m_{theory}} * 100$$

$$Loading\ \left(\frac{mmol}{g}\right) = Yield * \frac{n}{m_{ex}}$$

In case of non-quantitative yields, the calculated yields from the mass difference as well as the theoretical loading for quantitative conversion are given for the resin products.

To analyze the obtained resins, ^{13}C gel NMR spectroscopy was employed. Around 80-100 mg of the product resin was filled in an NMR tube and swollen with chloroform-*d*. For the measurement with the pulse program zgpg 30, a relaxation time of D1 = 0.2 sec and a line broadening of LB = 9.0 Hz were set. The samples were measured with 5120 scans.

2. Synthetic Results

Conversion of Triazenylpyrazoles to Azides:

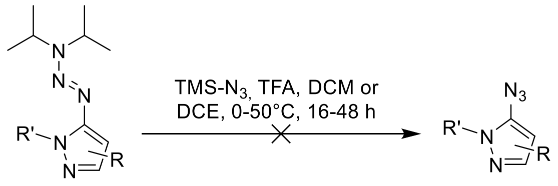

18 **19**

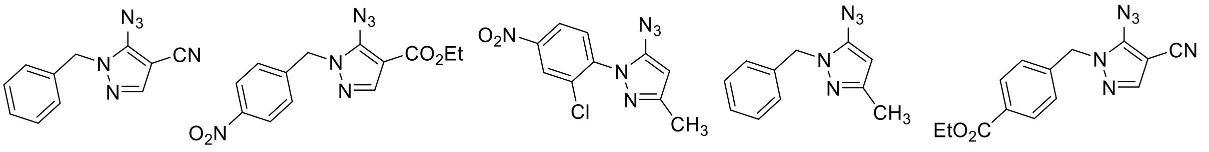

S1a **S1b** **S1c** **S1d** **S1e**

Scheme S1: Synthesis attempts for the generation of azides **19** from triazenylpyrazoles in regioisomeric form **18**. None of the expected products **S1a**-**S1e** could be isolated from the reaction mixture and no conversion of the starting material was observed.

Crystal structure of an *N*-substituted triazenylpyrazole (regioisomer 18):

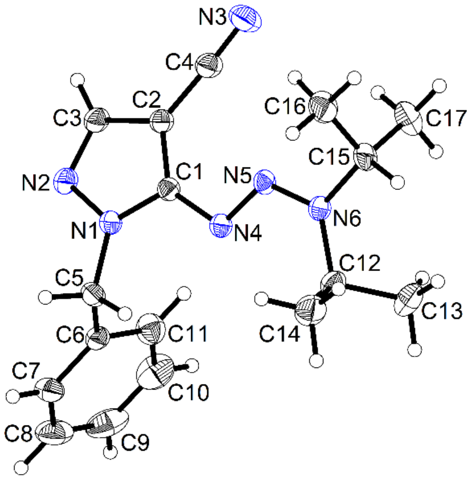

Figure S1: ORTEP diagram of triazenylpyrazole **18n** with the thermal ellipsoids shown at 50% probability.

Introduction of an electron-rich aryl group into the triazole-pyrazole hybrid:

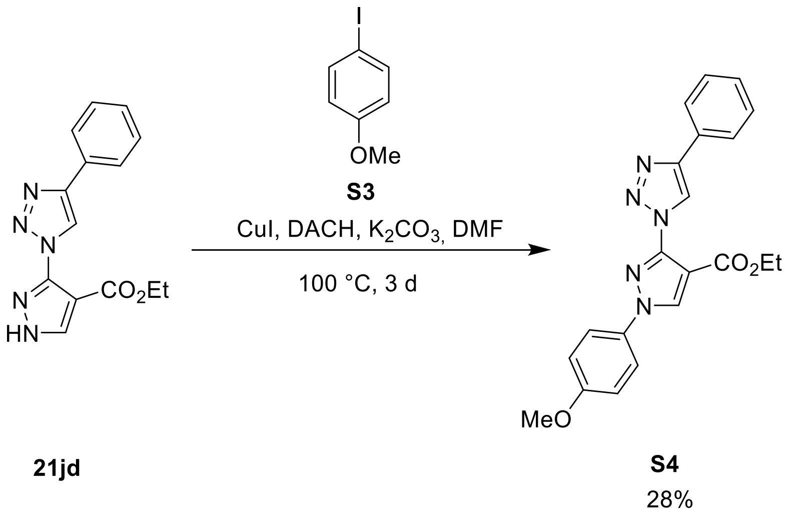

Scheme S2: Copper-catalyzed cross-coupling [4] for the functionalization of unsubstituted triazole-pyrazole hybrids with electron-rich moieties. DACH = 1,2-diaminocyclohexane.

Synthesis of the Compounds:

The synthesis of compounds **15d** and **17p** has been previously reported by our group with identical results [5] and is given below for the reader's convenience. The synthesis of compounds **17m**, **17n**, and **17o** has been previously reported by our group [5]. Below, new results with higher product yields are given.

1*H*-Pyrazole-5-diazonium tetrafluoroborate (S2)

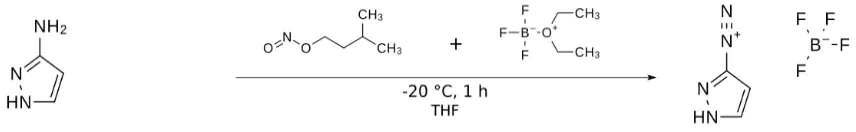

Name {P1|**S2**}: 1*H*-pyrazole-5-diazonium tetrafluoroborate; Formula: $C_3H_3BF_4N_4$; Smiles: c1c[nH]nc1[N+]#N.F[B-](F)(F)F. InChIKey: HZZGLJSNRHKVLS-UHFFFAOYSA-N

1*H*-pyrazol-5-amine (1.00 g, 12.0 mmol, 1.00 equiv) was dissolved under nitrogen in dry THF (33.0 mL) and boron trifluoride etherate (5.12 g, 4.46 mL, 36.1 mmol, 3.00 equiv) was added. The reaction mixture was cooled to -20 °C and 3-methylbutyl nitrite (4.23 g, 4.85 mL, 36.1 mmol, 3.00 equiv) was slowly added and stirred at this temperature for one hour. The precipitate was filtered off and washed with cold diethyl ether. The diazonium salt is directly further reacted.

Additional information on the chemical synthesis is available via Chemotion repository: https://doi.org/10.14272/reaction/SA-FUHFF-UHFFFADPSC-HZZGLJSNRH-UHFFFADPSC-NUHFF-NUHFF-NUHFF-ZZZ

(*E*)-3-(3,3-Diisopropyltriaz-1-en-1-yl)-1*H*-pyrazole (15a)

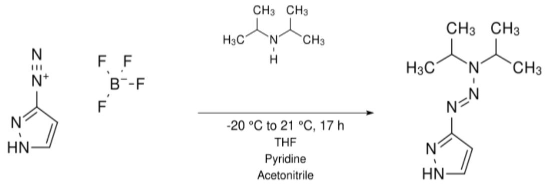

Name {P1|**15a**}: (*E*)-3-(3,3-diisopropyltriaz-1-en-1-yl)-1*H*-pyrazole; Formula: $C_9H_{17}N_5$; Smiles: CC(N(C(C)C)/N=N/c1n[nH]cc1)C. InChIKey: LKGAYQXGDXKVDA-OUKQBFOZSA-N

N-Propan-2-ylpropan-2-amine (1.29 g, 1.78 mL, 12.7 mmol, 1.10 equiv) was dissolved under nitrogen in dry THF (30.0 mL) and pyridine (3.40 mL) 9:1 at -20 °C. 1*H*-Pyrazole-5-diazonium tetrafluoroborate (2.10 g, 11.5 mmol, 1.00 equiv) was dissolved in acetonitrile (5.00 mL) and this solution was slowly added. The reaction mixture was slowly warmed up to 21 °C and stirred at this temperature for 16 hours. As preparation for the column chromatography (dryload), Celite was added (3 g) and the reaction mixture with Celite were evaporated. The obtained crude product was purified via flash-

chromatography (Interchim devices puriFLASH 5.125) on silica gel (PF-15SIHP-F0080) using cyclohexane/ethyl acetate 0% to 50% ethyl acetate in 12 column volumes (1 column volume = 173.2 mL; flow: 34 mL/min). The isolated product (*E*)-3-(3,3-diisopropyltriaz-1-en-1-yl)-1*H*-pyrazole (1.09 g, 5.59 mmol) was obtained as a yellow solid in 48% yield.

R_f = 0.26 (cyclohexane/ethyl acetate 1:1). MP = 106.8–112.9 °C. ^{1}H NMR (400 MHz, Chloroform-d [7.27 ppm], ppm) δ = 10.84 (s, 1H), 7.47 (d, *J* = 2.2 Hz, 1H), 6.22 (d, *J* = 2.2 Hz, 1H), 5.27 (ps, 1H), 3.96 (ps, 1H), 1.28 (ps, 12H); ^{13}C NMR (100 MHz, Chloroform-d [77.0 ppm], ppm) δ = 158.7, 132.9, 93.1, 48.7, 45.7, 23.6 (2C), 19.1 (2C); MS (EI, 70 eV, 40 °C), m/z (%): 195 (98) $[M]^+$, 100 (59), 95 (100), 86 (92), 58 (42). HRMS–EI ($C_9H_{17}N_5$) *(m/z)*: $[M]^+$ Calcd 195.1478; Found 195.1479; IR (ATR, ṽ) = 3153 (w), 3142 (m), 3060 (w), 2972 (s), 2929 (m), 2829 (w), 2765 (w), 1537 (w), 1465 (w), 1424 (vs), 1402 (vs), 1381 (m), 1361 (vs), 1290 (m), 1244 (vs), 1217 (s), 1187 (m), 1152 (vs), 1126 (s), 1098 (s), 1052 (w), 1028 (s), 996 (m), 922 (w), 904 (w), 875 (w), 822 (s), 758 (vs), 720 (w), 707 (m), 601 (m), 582 (w), 558 (m), 521 (s), 484 (w), 469 (w), 441 (w), 418 (w), 391 (w) cm^{-1}.

Additional information on the chemical synthesis is available via Chemotion repository: https://doi.org/10.14272/reaction/SA-FUHFF-UHFFFADPSC-LKGAYQXGDX-UHFFFADPSC-NUHFF-NZGMW-NUHFF-ZZZ

Additional information on the analysis of the target compound is available via Chemotion repository:
https://doi.org/10.14272/LKGAYQXGDXKVDA-OUKQBFOZSA-N.1

(*E*)-3-(3,3-Diisopropyltriaz-1-en-1-yl)-5-methyl-1*H*-pyrazole (15b)

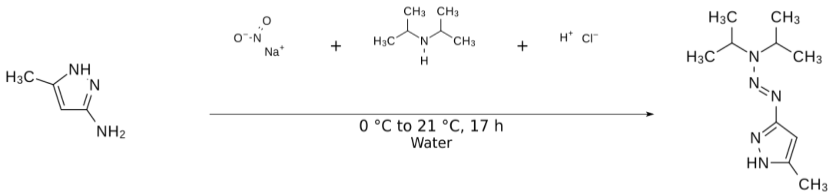

Name {P1|**15b**}: (*E*)-3-(3,3-diisopropyltriaz-1-en-1-yl)-5-methyl-1*H*-pyrazole; Formula: $C_{10}H_{19}N_5$; Smiles: CC(N(C(C)C)/N=N/c1n[nH]c(c1)C)C. InChIKey: SSAXAAXODLOITE-BUHFOSPRSA-N

To a cooled 0 °C solution of 5-methyl-1*H*-pyrazol-3-amine (1.00 g, 10.3 mmol, 1.00 equiv) in aqueous hydrochloric acid (10 mL, 6 M), a solution of sodium nitrite (781 mg, 11.3 mmol, 1.10 equiv) in water (6 mL) was added dropwise. The mixture was stirred for 1 h at 0 °C. Then, a 40% aqueous solution of *N*-propan-2-ylpropan-2-amine (10.4 g, 14.4 mL, 103 mmol, 10.0 equiv) was added. The solution was slowly warmed to room temperature and was stirred in the dark for 16 hours. The reaction mixture was extracted with methylene chloride. The combined organic phases were dried over Na_2SO_4 and filtered. As preparation for column chromatography (dry load), Celite was added (3 g) and the reaction mixture with Celite was evaporated. The obtained crude product was purified *via* flash-chromatography (Interchim devices puriFLASH 5.125) on silica gel (PF-15SIHP-F0080) using cyclohexane/ethyl acetate with 0% to 50% ethyl acetate in 25 column volumes (1 column volume = 173.2 mL;

flow: 34 mL/min). The isolated product (*E*)-3-(3,3-diisopropyltriaz-1-en-1-yl)-5-methyl-1*H*-pyrazole (1.75 g, 8.36 mmol) was obtained as a yellow solid in 81% yield.

R_f = 0.10 (cyclohexane/ethyl acetate 1:1). MP = 125.6–150.2 °C. ^{1}H NMR (400 MHz, Chloroform-d [7.27 ppm], ppm) δ = 8.93 (br. s, 1H), 6.01 (s, 1H), 5.29 (p s, 1H), 3.98 (ps, 1H), 2.31 (s, 3H), 1.36–1.17 (m, 12H); ^{13}C NMR (100 MHz, Chloroform-d [77.0 ppm], ppm) δ = 158.4, 143.9, 93.2, 48.8, 45.9, 23.8 (2C), 19.2 (2C), 12.4. MS (EI, 70 eV, 50 °C), m/z (%): 209 (98) $[M]^+$, 109 (100), 100 (72), 86 (76), 81 (20), 58 (58), 54 (61). HRMS–EI ($C_{10}H_{19}N_5$) *(m/z)*: $[M]^+$ Calcd 209.1635; Found 209.1635; IR (ATR, ṽ) = 3196 (w), 3145 (w), 3101 (w), 3027 (w), 2975 (m), 2929 (m), 2867 (m), 2769 (w), 1594 (w), 1468 (m), 1422 (vs), 1397 (vs), 1378 (m), 1363 (vs), 1309 (s), 1247 (vs), 1221 (vs), 1174 (w), 1156 (vs), 1126 (vs), 1098 (w), 1035 (m), 1021 (vs), 1007 (m), 908 (m), 877 (m), 853 (s), 840 (s), 768 (s), 734 (w), 707 (w), 662 (w), 548 (m), 521 (m), 496 (w), 412 (w) cm^{-1}.

Additional information on the chemical synthesis is available via Chemotion repository: https://doi.org/10.14272/reaction/SA-FUHFF-UHFFFADPSC-SSAXAAXODL-UHFFFADPSC-NUHFF-NICYD-NUHFF-ZZZ

Additional information on the analysis of the target compound is available via Chemotion repository:
https://doi.org/10.14272/SSAXAAXODLOITE-BUHFOSPRSA-N.1

Ethyl (*E*)-3-(3,3-diisopropyltriaz-1-en-1-yl)-1*H*-pyrazole-4-carboxylate (15c)

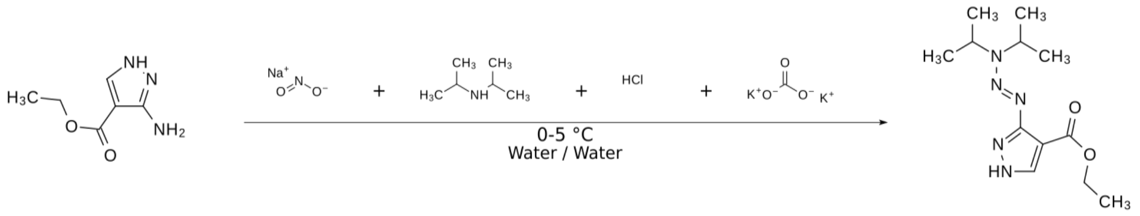

Name {P1|**15c**}: ethyl (*E*)-3-(3,3-diisopropyltriaz-1-en-1-yl)-1*H*-pyrazole-4-carboxylate; Formula: $C_{12}H_{21}N_5O_2$; Smiles: CCOC(=O)c1c[nH]nc1/N=N/N(C(C)C)C(C)C. InChIKey: XLGLVRGMYSSDFW-FOCLMDBBSA-N.

A mixture of ethyl 5-amino-1*H*-pyrazole-4-carboxylate (1.00 g, 6.5 mmol, 1.00 equiv) in conc. hydrochloric acid (940 mg, 2.15 mL, 26 mmol, 3.99 equiv) and 3 mL of water was cooled to 0 °C and a solution of sodium nitrite (667 mg, 9.7 mmol, 1.50 equiv) in 10 mL of water was added. After stirring for 30 min, a mixture of diisopropylamine (848 mg, 1.17 mL, 8.4 mmol, 1.30 equiv) and potassium carbonate (1.78 g, 13 mmol, 2.00 equiv) in 25 mL of water was added. The reaction mixture was stirred at 21 °C until TLC showed that all diazonium salt had disappeared. The reaction mixture was extracted three times with 750 mL of methylene chloride. The combined organic phases were washed with 3x 60 mL of water, dried over Na_2SO_4 and the solvent was evaporated under reduced pressure to give the crude product. The obtained crude product was purified *via* flash-chromatography on silica gel using cyclohexane/ethyl acetate 4:1 to 2:1 to give the target compound ethyl (*E*)-3-(3,3-diisopropyltriaz-1-en-1-yl)-1*H*-pyrazole-4-carboxylate in quantitative yield (1.72 g, 6.45 mmol).

R_f = 0.09 (cyclohexane/ethyl acetate 4:1). ^{1}H NMR (400 MHz, Chloroform-d [7.27 ppm], ppm) δ = 10.56 (br.s, 1H), 7.88 (s, 1H), 5.27 (hept, J = 6.8 Hz, 1H), 4.30 (q, J = 7.1 Hz,

2H), 4.08 (hept, J = 6.6 Hz, 1H), 1.38 (d, J = 6.6 Hz, 6H), 1.34 (t, J = 7.2 Hz, 3H), 1.30 (d, J = 6.8 Hz, 6H); ^{13}C NMR (100 MHz, Chloroform-d [77.16 ppm], ppm) δ = 163.5, 152.3, 142.7 (CH), 104.1, 59.9 (CH_2), 50.7 (CH), 48.1 (CH), 23.7 (2C, CH_3), 19.1 (2C, CH_3), 14.6 (CH_3); MS (EI, *m/z*, 70 eV, 70 °C): 267 (52) $[M]^+$, 222 (14), 167 (41), 112 (30), 111 (20), 109 (10), 100 (89), 99 (12), 96 (14), 95 (40), 86 (100), 84 (100), 70 (10), 69 (21), 68 (13), 67 (12), 66 (13), 58 (41), 52 (11), 51 (22). HRMS ($C_{12}H_{21}O_2N_5$): Calcd 267.1695. Found 267.1697. IR (ATR, ṽ) = 3123, 3091, 3037, 2970, 2932, 2873, 2788, 1715, 1694, 1567, 1489, 1469, 1446, 1409, 1392, 1380, 1367, 1273, 1241, 1213, 1187, 1160, 1103, 1081, 1033, 1014, 955, 946, 908, 884, 850, 823, 782, 741, 721, 703, 643, 611, 601, 572, 554, 534, 450, 409, 395 cm^{-1}.

Additional information on the chemical synthesis is available via Chemotion repository: https://doi.org/10.14272/reaction/SA-FUHFF-UHFFFADPSC-XLGLVRGMYS-UHFFFADPSC-NUHFF-NWPAI-NUHFF-ZZZ

Additional information on the analysis of the target compound is available via Chemotion repository:
https://doi.org/10.14272/XLGLVRGMYSSDFW-FOCLMDBBSA-N.1

(*E*)-3-(3,3-Diisopropyltriaz-1-en-1-yl)-1*H*-pyrazole-4-carbonitrile (15d)

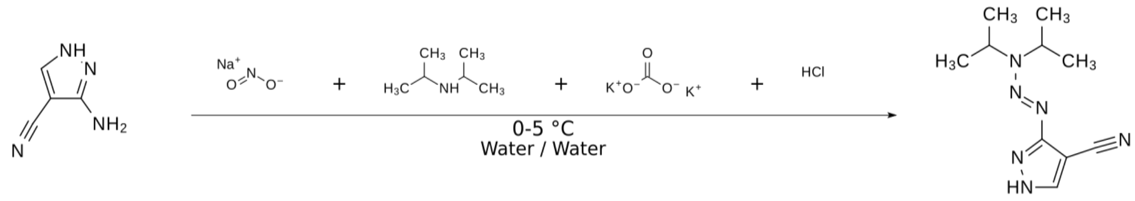

Name {P1|**15d**}: (*E*)-3-(3,3-diisopropyltriaz-1-en-1-yl)-1*H*-pyrazole-4-carbonitrile; Formula: $C_{10}H_{16}N_6$; Smiles: CC(N(C(C)C)/N=N/c1n[nH]cc1C#N)C. InChIKey: UCPMQTTXXMXIFF-CCEZHUSRSA-N

To a mixture of 5-amino-1*H*-pyrazole-4-carbonitrile (2.00 g, 19 mmol, 1.00 equiv) in 6 mL of water, conc. hydrochloric acid (6.17 mL, 74 mmol, 4.00 equiv) was added. The mixture was cooled to 0 °C and a solution of sodium nitrite (1.91 g, 28 mmol, 1.50 equiv) in 20 mL of water was added. Additional 10 mL of water were added to get the slurry able to be stirred. After stirring for 30 min at 0 °C, a mixture of diisopropylamine (2.43 g, 3.37 mL, 24 mmol, 1.30 equiv) and dipotassium carbonate (5.11 g, 37 mmol, 2.00 equiv) in 50 mL of water were added. The reaction mixture was stirred at room temperature until TLC showed that all diazonium salt had disappeared. The reaction mixture was extracted with 3x 150 mL of methylene chloride. Some precipitate was formed between the layers which had to be filtered off. The combined organic phases were washed with 3x 60 mL of water, dried over Na_2SO_4 and the solvent was evaporated under reduced pressure to give the desired product. The obtained crude product was purified *via* flash-chromatography on silica gel using cyclohexane/ethyl acetate 4:1 to 2:1 to give the target compound (*E*)-3-(3,3-diisopropyltriaz-1-en-1-yl)-1*H*-pyrazole-4-carbonitrile in 45% yield (1.85 g, 8.4 mmol).

R_f = 0.08 (cyclohexane/ethyl acetate 4:1). ^{1}H NMR (400 MHz, Chloroform-d [7.27 ppm], ppm) δ = 9.04 (br.s, 1H), 7.76 (s, 1H), 5.27 (hept, J = 6.8 Hz, 1H), 4.08 (hept, J = 6.7 Hz, 1H), 1.44 (d, J = 6.6 Hz, 6H), 1.28 (d, J = 6.8 Hz, 6H); ^{13}C NMR (100 MHz,

Chloroform-d [77.16 ppm], ppm) δ = 158.4, 141.3, 115.4 (CH), 79.7, 50.8 (CH), 47.6 (CH), 23.4 (2C, CH_3), 19.2 (2C, CH_3); EI (*m/z*, 70 eV, 80 °C): 220 (100) $[M]^+$, 135 (23), 134 (11), 120 (35), 109 (16), 108 (37), 100 (32), 86 (87), 84 (38), 70 (15), 69 (13), 65 (23), 58 (74), 52 (21). HRMS ($C_{10}H_{16}N_6$): Calcd 220.1436. Found 220.1438. IR (ATR, ṽ) = 408, 445, 511, 526, 541, 565, 589, 611, 629, 643, 713, 722, 751, 771, 816, 839, 861, 880, 899, 928, 948, 1031, 1067, 1077, 1096, 1130, 1163, 1194, 1217, 1231, 1262, 1295, 1306, 1319, 1339, 1364, 1378, 1392, 1412, 1451, 1466, 1492, 1543, 1572, 1720, 1792, 2183, 2227, 2873, 2934, 2973, 3053, 3095, 3143, 3231 cm^{-1}.

Additional information on the chemical synthesis is available via Chemotion repository: https://doi.org/10.14272/reaction/SA-FUHFF-UHFFFADPSC-UCPMQTTXXM-UHFFFADPSC-NUHFF-NRHPV-NUHFF-ZZZ

Additional information on the analysis of the target compound is available via Chemotion repository:
https://doi.org/10.14272/UCPMQTTXXMXIFF-CCEZHUSRSA-N.1

The synthesis of **15d** has been previously described by our group [5] and is given here for the reader's convenience.

(*E*)-1-Benzyl-3-(3,3-diisopropyltriaz-1-en-1-yl)-1*H*-pyrazole (17a), (*E*)-1-benzyl-5-(3,3-diisopropyltriaz-1-en-1-yl)-1*H*-pyrazole (18a)

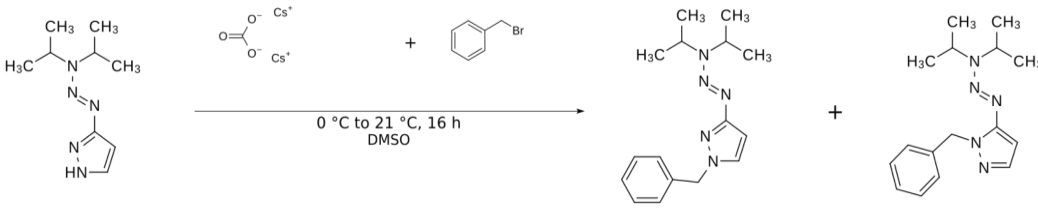

Name {P1|**17a**}: (*E*)-1-benzyl-3-(3,3-diisopropyltriaz-1-en-1-yl)-1*H*-pyrazole; Formula: $C_{16}H_{23}N_5$; Smiles: CC(N(C(C)C)/N=N/c1ccn(n1)Cc1ccccc1)C. InChIKey: MGAVNYXVFXWSSZ-HTXNQAPBSA-N

Name {P2|**18a**}: (*E*)-1-benzyl-5-(3,3-diisopropyltriaz-1-en-1-yl)-1*H*-pyrazole; Formula: $C_{16}H_{23}N_5$; Smiles: CC(N(C(C)C)/N=N/c1ccnn1Cc1ccccc1)C. InChIKey: ZIDRUQYCDSWWJM-VHEBQXMUSA-N

(*E*)-3-(3,3-diisopropyltriaz-1-en-1-yl)-1*H*-pyrazole (750 mg, 3.84 mmol, 1.00 equiv) was dissolved in DMSO (35.0 mL) and cesium carbonate (1.50 g, 4.61 mmol, 1.20 equiv) was added. The reaction mixture was cooled to 0 °C and bromomethylbenzene (1.31 g, 914 µL, 7.68 mmol, 2.00 equiv) was slowly added. After the addition, the reaction mixture was slowly warmed to 21 °C and stirred for 16 hours. Then ice-water was added to the reaction mixture. The aqueous phase was extracted three-times with ethyl acetate and the combined organic phase was washed with water and brine, dried over Na_2SO_4 and filtered. As preparation for the column chromatography (dryload), Celite was added (2.0 g) and the reaction mixture with Celite was evaporated. The obtained crude product was purified via flash-chromatography (Interchim devices puriFLASH 5.125) on silica gel (PF-15SIHP-F0080) using cyclohexane/ethyl acetate 0% to 20% ethyl acetate in 10 column volumes (1 column volume = 173.2 mL; flow: 34 mL/min). The isolated product (*E*)-1-benzyl-3-(3,3-diisopropyltriaz-1-en-1-yl)-1*H*-pyrazole (645 mg, 2.26 mmol) was

obtained as an orange oil in 59% yield and the side product (*E*)-1-benzyl-5-(3,3-diisopropyltriaz-1-en-1-yl)-1*H*-pyrazole (200 mg, 701 µmol) was obtained as an orange solid in 18% yield.

R_f = 0.47 (cyclohexane/ethyl acetate 2:1). ^{1}H NMR (400 MHz, Chloroform-d [7.27 ppm], ppm) δ = 7.35–7.26 (m, 5H), 7.23 (d, *J* = 2.4 Hz, 1H), 6.25 (d, *J* = 2.4 Hz, 1H), 5.40 (ps, 1H), 5.26 (s, 2H), 3.96 (ps, 1H), 1.36–1.22 (m, 12H); ^{13}C NMR (100 MHz, Chloroform-d [77.0 ppm], ppm) δ = 162.2 (C_q), 136.8 (C_q), 130.0 (CH), 128.7 (CH, 2C), 127.8 (CH, 3C), 93.8 (CH), 56.0 (CH_2), 48.6 (CH), 45.5 (CH), 23.9 (CH_3, 2C), 19.3 (CH_3, 2C); MS (EI, 70 eV, 60 °C), m/z (%): 285 (17) $[M]^+$, 185 (57), 158 (24), 130 (15), 104 (17), 100 (11), 91 (100), 86 (10), 77 (19), 65 (19). HRMS–EI ($C_{16}H_{23}N_5$) *(m/z)*: $[M]^+$ Calcd 285.1948; Found 285.1950; IR (ATR, ṽ) = 2972 (m), 2931 (w), 2867 (w), 2834 (w), 2759 (w), 2718 (w), 2489 (w), 1737 (vw), 1703 (vw), 1599 (w), 1587 (w), 1543 (vw), 1517 (m), 1496 (w), 1466 (s), 1456 (s), 1422 (vs), 1400 (vs), 1380 (s), 1361 (vs), 1323 (s), 1241 (vs), 1205 (s), 1184 (m), 1152 (vs), 1128 (s), 1101 (s), 1077 (m), 1055 (s), 1031 (vs), 1006 (m), 990 (m), 977 (m), 956 (m), 907 (m), 877 (w), 830 (m), 806 (w), 754 (vs), 730 (vs), 704 (vs), 626 (m), 615 (m), 586 (m), 575 (m), 555 (m), 523 (m), 510 (s), 472 (m), 458 (m), 436 (w), 424 (w), 415 (w), 401 (w), 390 (m), 375 (w) cm^{-1}.

R_f = 0.56 (cyclohexane/ethyl acetate 2:1). MP = 49.4–50.5 °C. ^{1}H NMR (400 MHz, Chloroform-d [7.27 ppm], ppm) δ = 7.42 (d, *J* = 2.0 Hz, 1H), 7.31–7.20 (m, 5H), 6.09 (d, *J* = 2.1 Hz, 1H), 5.44 (s, 2H), 5.06 (sept, *J* = 6.8 Hz, 1H), 4.00 (sept, *J* = 6.7 Hz, 1H), , 1.34 (d, *J* = 6.0 Hz, 6H), 1.22 (d, *J* = 6.2 Hz, 6H); ^{13}C NMR (100 MHz, Chloroform-d [77.0 ppm], ppm) δ = 151.6 (C_q), 138.9 (CH), 137.9 (C_q), 128.4 (CH, 2C), 127.6 (CH, 2C), 127.2 (CH), 90.3 (CH), 51.6 (CH_2), 49.8 (CH), 46.8 (CH), 23.6 (CH_3, 2C), 19.0 (CH_3, 2C); MS (EI, 70 eV, 60 °C), m/z (%): 285 (100) $[M]^+$, 185 (98), 157 (19), 130 (33), 128 (12), 104 (23), 100 (43), 91 (80), 77 (14), 58 (32). HRMS–EI ($C_{16}H_{23}N_5$) *(m/z)*: $[M]^+$ Calcd 285.1948; Found 285.1949; IR (ATR, ṽ) = 3089 (w), 2975 (m), 2955 (w), 2946 (w), 2932 (w), 2866 (w), 1519 (w), 1511 (w), 1497 (w), 1477 (w), 1468 (w), 1455 (w), 1412 (vs), 1381 (s), 1361 (vs), 1329 (m), 1317 (w), 1303 (w), 1258 (vs), 1218 (vs), 1207 (s), 1154 (s), 1130 (m), 1123 (m), 1102 (vs), 1069 (w), 1043 (s), 1021 (vs), 976 (w), 935 (w), 924 (s), 905 (m), 880 (w), 864 (m), 847 (w), 824 (w), 815 (w), 783 (w), 764 (vs), 722 (vs), 711 (vs), 697 (s), 686 (w), 649 (w), 620 (w), 589 (vs), 575 (m), 555 (s), 531 (s), 504 (w), 473 (w), 462 (w), 442 (w), 424 (w), 409 (w), 377 (w) cm^{-1}.

Additional information on the chemical synthesis is available *via* Chemotion repository: https://doi.org/10.14272/reaction/SA-FUHFF-UHFFFADPSC-FSSAEDRJOX-UHFFFADPSC-NUHFF-NAZOY-NUHFF-ZZZ

Additional information on the analysis of the target compound is available *via* Chemotion repository:
https://doi.org/10.14272/MGAVNYXVFXWSSZ-HTXNQAPBSA-N.1
https://doi.org/10.14272/ZIDRUQYCDSWWJM-VHEBQXMUSA-N.1

(*E*)-1-(4-Bromobenzyl)-3-(3,3-diisopropyltriaz-1-en-1-yl)-1*H*-pyrazole (17b), (*E*)-1-(4-bromobenzyl)-5-(3,3-diisopropyltriaz-1-en-1-yl)-1*H*-pyrazole (18b)

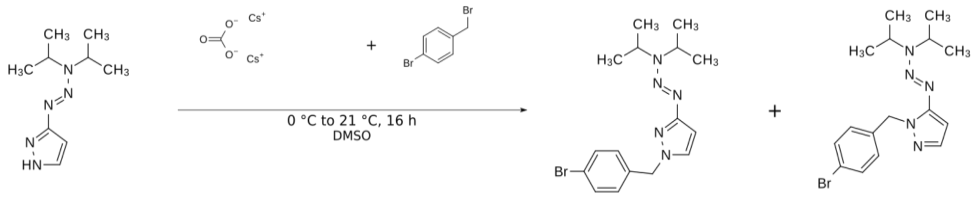

Name {P1|**17b**}: (*E*)-1-(4-bromobenzyl)-3-(3,3-diisopropyltriaz-1-en-1-yl)-1*H*-pyrazole; Formula: $C_{16}H_{22}BrN_5$; Smiles: CC(N(C(C)C)/N=N/c1ccn(n1)Cc1ccc(cc1)Br)C. InChIKey: OJEKMYNEGYNUPP-CZIZESTLSA-N

Name {P2|**18b**}: (*E*)-1-(4-bromobenzyl)-5-(3,3-diisopropyltriaz-1-en-1-yl)-1*H*-pyrazole; Formula: $C_{16}H_{22}BrN_5$; Smiles: Brc1ccc(cc1)Cn1nccc1/N=N/N(C(C)C)C(C)C. InChIKey: PLVZHOAUEJAYPS-FMQUCBEESA-N

(*E*)-3-(3,3-Diisopropyltriaz-1-en-1-yl)-1*H*-pyrazole (500 mg, 2.56 mmol, 1.00 equiv) was dissolved in DMSO (26.0 mL) and cesium carbonate (1.00 g, 3.07 mmol, 1.20 equiv) was added. The reaction mixture was cooled to 0 °C and 1-bromo-4-(bromomethyl)benzene (1.28 g, 5.12 mmol, 2.00 equiv) was slowly added. After the addition, the reaction mixture was slowly warmed to 21 °C and stirred for 16 hours at this temperature. For workup, ice water was added to the reaction mixture. The aqueous phase was extracted three times with ethyl acetate and the combined organic phases were washed with water and brine, dried over Na_2SO_4, filtered and the solvent was removed under reduced pressure. As preparation for column chromatography (dry load), Celite was added (1.5 g) and the reaction mixture with Celite was evaporated. The obtained crude product was purified *via* flash-chromatography (Interchim devices puriFLASH 5.125) on silica gel (PF-15SIHP-F0080) using cyclohexane/ethyl acetate with 0% to 25% ethyl acetate in 12 column volumes. The isolated product (*E*)-1-(4-bromobenzyl)-3-(3,3-diisopropyltriaz-1-en-1-yl)-1*H*-pyrazole (314 mg, 862 µmol) was obtained as a colorless solid in 34% yield and the side product (*E*)-1-(4-bromobenzyl)-5-(3,3-diisopropyltriaz-1-en-1-yl)-1*H*-pyrazole (264 mg, 724 µmol) was obtained as a colorless solid in 28% yield.

R_f = 0.45 (cyclohexane/ethyl acetate 2:1). MP = 79.5–88.9 °C. ^{1}H NMR (400 MHz, Chloroform-d [7.27 ppm], ppm) δ = 7.46–7.43 (m, 2H), 7.24 (d, *J* = 2.3 Hz, 1H), 7.14 (d, *J* = 8.6 Hz, 2H), 6.25 (d, *J* = 2.3 Hz, 1H), 5.38 (ps, 1H), 5.20 (s, 2H), 3.96 (ps, 1H), 1.35 (ps, 6H), 1.22 (ps, 6H); ^{13}C NMR (100 MHz, Chloroform-d [77.0 ppm], ppm) δ = 162.4 (C_q), 136.0 (C_q), 131.7 (CH, 2C), 130.0 (CH), 129.4 (CH, 2C), 121.8 (C_q), 94.0 (CH), 55.2 (CH_2), 48.6 (CH), 45.5 (CH), 23.8 (CH_3, 2C), 19.3 (CH_3, 2C); MS (FAB, 3-NBA), m/z (%): 364/366 (100/91) $[M+1]^+$, 263/265 (97/95), 169/171 (32/30), 154 (24), 136 (23), 107/109 (12/12), 100 (44), 95/97 (19/15), 91 (18). HRMS–FAB ($C_{16}H_{23}N_5Br$) *(m/z)*: $[M+H]^+$ Calcd 364.1131; Found 364.1129; IR (ATR, ṽ) = 2982 (w), 2970 (w), 2929 (w), 2870 (vw), 1683 (vw), 1595 (vw), 1516 (w), 1487 (w), 1466 (w), 1426 (vs), 1404 (vs), 1383 (w), 1360 (s), 1324 (m), 1242 (vs), 1230 (s), 1207 (w), 1193 (m), 1149 (vs), 1126 (s), 1105 (w), 1096 (m), 1069 (m), 1050 (m), 1030 (s), 1013 (m), 1004 (w), 994 (w), 960 (w), 943 (w), 922 (vw), 907 (w), 875 (w), 850 (w), 840 (w), 832 (w), 822 (w), 805 (m), 754 (vs), 722 (m), 708 (w), 686 (w), 639 (w), 605 (w), 585 (w), 557 (w), 527 (m), 509 (m), 483 (m), 421 (w), 390 (w) cm^{-1}.

R_f = 0.58 (cyclohexane/ethyl acetate 2:1). MP = 62.8–64.0 °C. ^{1}H NMR (400 MHz, Chloroform-d [7.27 ppm], ppm) δ = 7.43–7.39 (m, 3H), 7.16–7.12 (m, 2H), 6.10 (d, *J* = 2.1 Hz, 1H), 5.39 (s, 2H), 5.04 (quint, *J* = 6.9 Hz, 1H), 4.01 (quint, *J* = 6.6 Hz, 1H), 1.35 (d, *J* = 6.6 Hz, 6H), 1.23 (d, *J* = 6.8 Hz, 6H); ^{13}C NMR (100 MHz, Chloroform-d [77.0 ppm], ppm) δ = 151.6 (C_q), 139.1 (CH), 137.0 (C_q), 131.5 (CH, 2C), 129.3 (CH, 2C), 121.2 (C_q), 90.4 (CH), 51.0 (CH_2), 49.9 (CH), 46.9 (CH), 23.6 (CH_3, 2C), 19.0 (CH_3, 2C); MS (FAB, 3-NBA), m/z (%): 364/366 (100/93) $[M+1]^+$, 263/265 (14/13), 169/171 (14/14), 154 (13), 136 (11), 100 (19), 95 (11), 91 (10). HRMS–FAB ($C_{16}H_{23}N_5Br$) *(m/z)*: $[M+H]^+$ Calcd 364.1131; Found 364.1129; IR (ATR, ṽ) = 3112 (vw), 2972 (m), 2946 (w), 2931 (w), 2861 (w), 2800 (vw), 1713 (vw), 1591 (vw), 1519 (w), 1487 (m), 1472 (m), 1431 (m), 1407 (vs), 1375 (s), 1361 (vs), 1322 (m), 1295 (w), 1261 (vs), 1222 (vs), 1210 (m), 1167 (m), 1152 (s), 1128 (m), 1109 (s), 1095 (s), 1082 (m), 1067 (s), 1045 (s), 1024 (s), 1013 (vs), 952 (w), 929 (s), 905 (m), 880 (w), 858 (w), 839 (s), 799 (s), 764 (vs), 747 (vs), 715 (w), 693 (w), 686 (w), 649 (m), 622 (m), 584 (m), 555 (vs), 531 (s), 506 (m), 482 (s), 422 (w), 402 (w), 395 (w), 381 (w) cm^{-1}.

Additional information on the chemical synthesis is available *via* Chemotion repository: https://doi.org/10.14272/reaction/SA-FUHFF-UHFFFADPSC-QINWFVLSKF-UHFFFADPSC-NUHFF-NIMLK-NUHFF-ZZZ

Additional information on the analysis of the target compound is available *via* Chemotion repository:
https://doi.org/10.14272/OJEKMYNEGYNUPP-CZIZESTLSA-N.1
https://doi.org/10.14272/PLVZHOAUEJAYPS-FMQUCBEESA-N.1

(*E*)-1-Cyclopentyl-3-(3,3-diisopropyltriaz-1-en-1-yl)-1*H*-pyrazole (17c), (*E*)-1-cyclopentyl-5-(3,3-diisopropyltriaz-1-en-1-yl)-1*H*-pyrazole (18c)

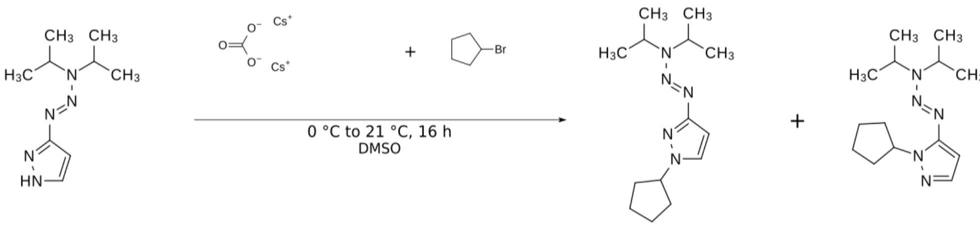

Name {P1|**17c**}: (*E*)-1-cyclopentyl-3-(3,3-diisopropyltriaz-1-en-1-yl)-1*H*-pyrazole; Formula: $C_{14}H_{25}N_5$; Smiles: CC(N(C(C)C)/N=N/c1ccn(n1)C1CCCC1)C. InChIKey: JJSUJDRUQGTCKX-BMRADRMJSA-N

Name {P2|**18c**}: (*E*)-1-cyclopentyl-5-(3,3-diisopropyltriaz-1-en-1-yl)-1*H*-pyrazole; Formula: $C_{14}H_{25}N_5$; Smiles: CC(N(C(C)C)/N=N/c1ccnn1C1CCCC1)C. InChIKey: BWAGQJOINMBVOL-WUKNDPDISA-N

(*E*)-3-(3,3-Diisopropyltriaz-1-en-1-yl)-1*H*-pyrazole (461 mg, 2.36 mmol, 1.00 equiv) was dissolved in DMSO (26.0 mL) and cesium carbonate (923 mg, 2.83 mmol, 1.20 equiv) was added. The reaction mixture was cooled to 0 °C and bromocyclopentane (704 mg, 506 µL, 4.72 mmol, 2.00 equiv) was slowly added. After the addition, the reaction mixture was slowly warmed to 21 °C and stirred for 16 hours at this temperature. For work up ice-water was added to the reaction mixture. The aqueous phase was extracted three-times with ethyl acetate and the combined organic phase was washed with water and brine, dried over Na_2SO_4 and filtered. As

preparation for the column chromatography (dryload), Celite was added (1.5 g) and the reaction mixture with Celite were evaporated. The obtained crude product was purified *via* flash-chromatography (Interchim devices puriFLASH 5.125) on silica gel (PF-15SIHP-F0080) using cyclohexane/ethyl acetate 0% to 20% ethyl acetate in 15 column volumes (1 column volume = 173.2 mL; flow: 34 mL/min). The isolated product (*E*)-1-cyclopentyl-3-(3,3-diisopropyltriaz-1-en-1-yl)-1*H*-pyrazole (251 mg, 952 μmol) was obtained as a yellow solid in 40% yield and the side product (*E*)-1-cyclopentyl-5-(3,3-diisopropyltriaz-1-en-1-yl)-1*H*-pyrazole (249 mg, 945 μmol) was obtained as a yellow oil in 40% yield.

R_f = 0.56 (cyclohexane/ethyl acetate 2:1). MP = 80–86.3 °C. ^{1}H NMR (400 MHz, Chloroform-d [7.27 ppm], ppm) δ = 7.29 (d, *J* = 2.4 Hz, 1H), 6.19 (d, *J* = 2.3 Hz, 1H), 5.47 (ps, 1H), 4.57 (quint, *J* = 7.4 Hz, 1H), 3.94 (ps, 1H), 2.18–2.04 (m, 4H), 1.92–1.82 (m, 2H), 1.72–1.62 (m, 2H), 1.34–1.21 (m, 12H); ^{13}C NMR (100 MHz, Chloroform-d [77.0 ppm], ppm) δ = 162.0, 128.4, 92.4, 63.0, 48.3, 45.2, 32.7 (2C), 24.1 (2C), 23.9 (2C), 19.4 (2C); MS (EI, 70 eV, 60 °C), m/z (%): 263 (28) $[M]^+$, 163 (100), 100 (11). HRMS–EI ($C_{14}H_{25}N_5$) *(m/z)*: $[M]^+$ Calcd 263.2104; Found 263.2107; IR (ATR, ṽ) = 2970 (m), 2956 (m), 2934 (w), 2870 (w), 2761 (vw), 2721 (vw), 2490 (vw), 1514 (w), 1468 (m), 1456 (m), 1439 (w), 1417 (vs), 1404 (vs), 1375 (m), 1361 (s), 1341 (s), 1313 (w), 1293 (w), 1241 (vs), 1225 (vs), 1193 (w), 1153 (vs), 1130 (s), 1096 (m), 1052 (s), 1030 (vs), 1003 (w), 994 (w), 941 (w), 908 (w), 877 (w), 832 (m), 752 (vs), 710 (w), 671 (w), 635 (w), 585 (w), 557 (m), 523 (w), 448 (vw), 414 (w), 382 (w) cm^{-1}.

R_f = 0.69 (cyclohexane/ethyl acetate 2:1). ^{1}H NMR (400 MHz, Chloroform-d [7.27 ppm], ppm) δ = 7.40 (d, *J* = 2.0 Hz, 1H), 6.06 (d, *J* = 2.0 Hz, 1H), 5.09 (quint, *J* = 7.8 Hz, 2H), 4.01 (ps, 1H), 2.19–2.03 (m, 4H), 1.99–1.89 (m, 2H), 1.72–1.62 (m, 2H), 1.32 (m, 12H); ^{13}C NMR (100 MHz, Chloroform-d [77.0 ppm], ppm) δ = 151.4, 138.0, 90.2, 58.0, 49.7, 46.7, 32.3 (2C), 24.8 (2C), 23.7 (2C), 19.1 (2C); MS (EI, 70 eV, 30 °C), m/z (%): 263 (70) $[M]^+$, 163 (100), 100 (23), 95 (20), 67 (12). HRMS–EI ($C_{14}H_{25}N_5$) *(m/z)*: $[M]^+$ Calcd 263.2104; Found 263.2105; IR (ATR, ṽ) = 2969 (m), 2953 (m), 2918 (m), 2868 (w), 2849 (w), 1519 (w), 1462 (s), 1415 (vs), 1381 (s), 1363 (vs), 1315 (w), 1252 (vs), 1222 (vs), 1203 (m), 1157 (vs), 1126 (s), 1101 (s), 1074 (m), 1045 (s), 1023 (vs), 941 (m), 905 (s), 860 (w), 837 (w), 761 (vs), 731 (m), 720 (m), 688 (w), 653 (m), 594 (m), 574 (w), 557 (s), 527 (m), 436 (w), 399 (w) cm^{-1}.

Additional information on the chemical synthesis is available *via* Chemotion repository: https://doi.org/10.14272/reaction/SA-FUHFF-UHFFFADPSC-VFSIPNHSCH-UHFFFADPSC-NUHFF-NDGQT-NUHFF-ZZZ

Additional information on the analysis of the target compound is available *via* Chemotion repository:
https://doi.org/10.14272/JJSUJDRUQGTCKX-BMRADRMJSA-N.1
https://doi.org/10.14272/BWAGQJOINMBVOL-WUKNDPDISA-N.1

(*E*)-1-Benzyl-3-(3,3-diisopropyltriaz-1-en-1-yl)-5-methyl-1*H*-pyrazole (17d), (*E*)-1-benzyl-5-(3,3-diisopropyltriaz-1-en-1-yl)-3-methyl-1*H*-pyrazole (18d)

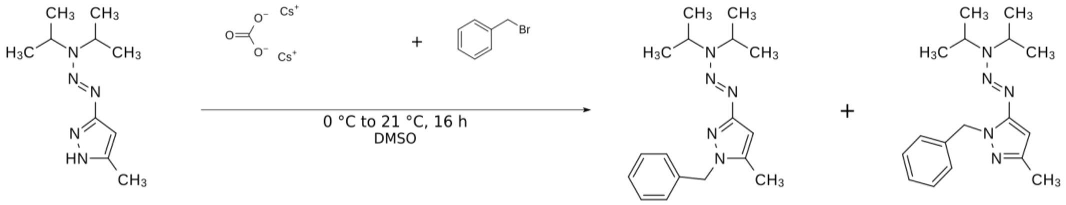

Name {P1|**17d**}: (*E*)-1-benzyl-3-(3,3-diisopropyltriaz-1-en-1-yl)-5-methyl-1*H*-pyrazole; Formula: $C_{17}H_{25}N_5$; Smiles: CC(N(C(C)C)/N=N/c1nn(c(c1)C)Cc1ccccc1)C. InChIKey: FEHNUWIZIPJQLZ-CZIZESTLSA-N

Name {P2|**18d**}: (*E*)-1-benzyl-5-(3,3-diisopropyltriaz-1-en-1-yl)-3-methyl-1*H*-pyrazole; Formula: $C_{17}H_{25}N_5$; Smiles: CC(N(C(C)C)/N=N/c1cc(nn1Cc1ccccc1)C)C. InChIKey: OSZGZNAJFDDFLV-CZIZESTLSA-N

(*E*)-3-(3,3-Diisopropyltriaz-1-en-1-yl)-5-methyl-1*H*-pyrazole (500 mg, 2.39 mmol, 1.00 equiv) was dissolved in DMSO (25.0 mL) and cesium carbonate (934 mg, 2.87 mmol, 1.20 equiv) was added. The reaction mixture was cooled to 0 °C and bromomethylbenzene (817 mg, 568 µL, 4.78 mmol, 2.00 equiv) was slowly added. After the addition, the reaction mixture was slowly warmed to 21 °C and stirred for 16 hours at this temperature. For work up ice-water was added to the reaction mixture. The aqueous phase was extracted three-times with ethyl acetate and the combined organic phase was washed with water and brine, dried over Na_2SO_4 and filtered. As preparation for the column chromatography (dryload), Celite was added (1.5 g) and the reaction mixture with Celite was evaporated. The obtained crude product was purified *via* flash-chromatography (Interchim devices puriFLASH 5.125) on silica gel (PF-15SIHP-F0080) using cyclohexane/ethyl acetate 0% to 20% ethyl acetate in 12 column volumes (1 column volume = 173.2 mL; flow: 34 mL/min). The isolated product (*E*)-1-benzyl-3-(3,3-diisopropyltriaz-1-en-1-yl)-5-methyl-1*H*-pyrazole (162 mg, 542 µmol) was obtained as a yellow oil in 23% yield and the side product (*E*)-1-benzyl-5-(3,3-diisopropyltriaz-1-en-1-yl)-3-methyl-1*H*-pyrazole (207 mg, 690 µmol) was obtained as a yellow solid in 29% yield.

R_f = 0.45 (cyclohexane/ethyl acetate 2:1). ^{1}H NMR (400 MHz, Chloroform-d [7.26 ppm], ppm) δ = 7.31–7.26 (m, 2H), 7.25–7.21 (m, 1H), 7.19–7.17 (m, 2H), 6.06 (d, *J* = 0.7 Hz, 1H), 5.40 (ps, 1H), 5.24 (s, 2H), 3.94 (ps, 1H), 2.16 (d, *J* = 0.6 Hz, 3H), 1.34 (ps, 6H), 1.21 (ps, 6H); ^{13}C NMR (100 MHz, Chloroform-d [77.0 ppm], ppm) δ = 160.9 (C_q), 139.3 (C_q), 137.4 (C_q), 128.6 (CH, 2C), 127.3 (CH), 126.9 (CH, 2C), 93.6 (CH), 52.9 (CH_2), 48.4 (CH), 45.4 (CH), 23.8 (CH_3, 2C), 19.4 (CH_3, 2C), 11.4 (CH_3); MS (EI, 70 eV, 70 °C), m/z (%): 299 (34) $[M]^+$, 199 (97), 171 (10), 130 (10), 118 (10), 100 (20), 91 (100), 86 (10), 58 (15). HRMS–EI ($C_{17}H_{25}N_5$) *(m/z)*: $[M]^+$ Calcd 299.2104; Found 299.2105; IR (ATR, ṽ) = 2972 (m), 2929 (w), 2867 (w), 2759 (w), 2720 (w), 2487 (w), 1605 (w), 1587 (w), 1548 (m), 1496 (w), 1468 (m), 1453 (s), 1424 (vs), 1401 (vs), 1378 (s), 1360 (vs), 1316 (s), 1242 (vs), 1213 (vs), 1156 (vs), 1119 (vs), 1103 (s), 1077 (m), 1041 (s), 1030 (m), 1014 (m), 977 (m), 958 (m), 908 (m), 878 (w), 841 (m), 776 (s), 731 (s), 711 (vs), 696 (s), 657 (m), 632 (w), 616 (w), 591 (m), 574 (m), 552 (s), 524 (m), 510 (m), 458 (m), 426 (w), 421 (w), 404 (w), 390 (w) cm^{-1}.

R_f = 0.55 (cyclohexane/ethyl acetate 2:1). ^{1}H NMR (400 MHz, Chloroform-d [7.26 ppm], ppm) δ = 7.31–7.20 (m, 5H), 5.92 (s, 1H), 5.38 (s, 2H), 5.07–5.00 (m, 1H), 4.03–3.96 (m, 1H), 2.26 (s, 3H), 1.34 (d, J = 4.8 Hz, 6H), 1.21 (d, J = 5.3 Hz, 6H); ^{13}C NMR (100 MHz, Chloroform-d [77.0 ppm], ppm) δ = 152.1 (C_q), 147.8 (C_q), 138.3 (C_q), 128.3 (CH, 2C), 127.4 (CH, 2C), 127.0 (CH), 89.9 (CH), 51.3 (CH_2), 49.7 (CH), 46.7 (CH), 23.6 (CH_3, 2C), 19.0 (CH_3, 2C), 14.1 (CH_3); MS (EI, 70 eV, 50 °C), m/z (%): 299 (100) $[M]^+$, 199 (80), 176 (10), 171 (12), 130 (38), 103 (10), 100 (40), 91 (67), 58 (24). HRMS–EI ($C_{17}H_{25}N_5$) *(m/z)*: $[M]^+$ Calcd 299.2104; Found 299.2106; IR (ATR, ṽ) = 2973 (w), 2928 (w), 2870 (w), 1686 (w), 1605 (w), 1528 (m), 1496 (w), 1465 (m), 1455 (m), 1414 (vs), 1380 (s), 1360 (vs), 1329 (w), 1316 (m), 1300 (w), 1258 (vs), 1222 (vs), 1159 (vs), 1118 (vs), 1096 (m), 1077 (w), 1030 (s), 1017 (s), 977 (w), 948 (w), 916 (m), 860 (w), 844 (w), 819 (w), 802 (w), 768 (s), 735 (m), 710 (s), 696 (s), 663 (w), 642 (m), 619 (w), 592 (w), 572 (m), 550 (m), 530 (m), 486 (w), 456 (w), 418 (w), 402 (w), 390 (w), 378 (w) cm^{-1}.

Additional information on the chemical synthesis is available *via* Chemotion repository: https://doi.org/10.14272/reaction/SA-FUHFF-UHFFFADPSC-WUGJXCOMZV-UHFFFADPSC-NUHFF-NODPP-NUHFF-ZZZ

Additional information on the analysis of the target compound is available *via* Chemotion repository:
https://doi.org/10.14272/FEHNUWIZIPJQLZ-CZIZESTLSA-N.1
https://doi.org/10.14272/OSZGZNAJFDDFLV-CZIZESTLSA-N.1

(*E*)-4-(3-(3,3-Diisopropyltriaz-1-en-1-yl)-5-methyl-1*H*-pyrazol-1-yl)benzonitrile (17e), (*E*)-4-(5-(3,3-diisopropyltriaz-1-en-1-yl)-3-methyl-1*H*-pyrazol-1-yl)benzo-nitrile (18e)

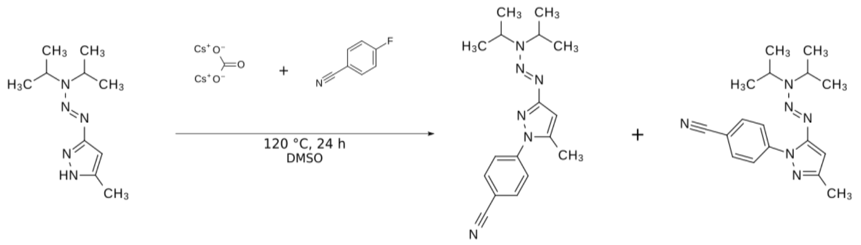

Name {P1|**17e**}: (*E*)-4-(3-(3,3-diisopropyltriaz-1-en-1-yl)-5-methyl-1*H*-pyrazol-1-yl)benzo-nitrile; Formula: $C_{17}H_{22}N_6$; Smiles: N#Cc1ccc(cc1)n1nc(cc1C)/N=N/N(C(C)C)C(C)C. InChIKey: RSDVJJOPRTZAOO-XUTLUUPISA-N

Name {P2|**18e**}: (*E*)-4-(5-(3,3-diisopropyltriaz-1-en-1-yl)-3-methyl-1*H*-pyrazol-1-yl)benzo-nitrile; Formula: $C_{17}H_{22}N_6$; Smiles: N#Cc1ccc(cc1)n1nc(cc1/N=N/N(C(C)C)C(C)C)C. InChIKey: WPXPVVIOPAJXSL-XUTLUUPISA-N

In a vial, (*E*)-3-(3,3-diisopropyltriaz-1-en-1-yl)-5-methyl-1*H*-pyrazole (76.6 mg, 366 µmol, 1.00 equiv) was dissolved in 3 mL of DMSO. Caesium carbonate (140 mg, 430 µmol, 1.17 equiv) and 4-fluorobenzonitrile (86.8 mg, 717 µmol, 1.96 equiv) were added. The vial was closed and stirred at 120 °C for 24 hours. The reaction was quenched with ice and was extracted with ethyl acetate and the combined organic phase was washed with water and brine, dried over Na_2SO_4 and filtered. As

preparation for the column chromatography (dryload), Celite was added and the reaction mixture with Celite was evaporated. The obtained crude product was purified *via* flash-chromatography on silica gel using cyclohexane/ethyl acetate 10:1 to 4:1. The isolated product (*E*)-4-(3-(3,3-diisopropyltriaz-1-en-1-yl)-5-methyl-1*H*-pyrazol-1-yl)benzo-nitrile (65.7 mg, 212 µmol) was obtained as a colorless solid in 58% yield and the side product (*E*)-4-(5-(3,3-diisopropyltriaz-1-en-1-yl)-3-methyl-1*H*-pyrazol-1-yl)benzonitrile (14.1 mg, 45.4 µmol) was obtained as a yellow oil in 12% yield.

R_f = 0.28 (cyclohexane/ethyl acetate 4:1). MP = 120.1–123 °C. ^{1}H NMR (400 MHz, Chloroform-d, ppm) δ = 7.64 (s, 4H), 6.23 (s, 1H), 5.37 (quint, *J* = 6.4 Hz, 1H), 3.92 (quint, *J* = 6.5 Hz, 1H), 2.38 (s, 3H), 1.30 (d, *J* = 6.2 Hz, 6H), 1.15 (d, *J* = 6.5 Hz, 6H); ^{13}C NMR (100 MHz, Chloroform-d, ppm) δ = 163.2 (C_q), 143.7 (C_q), 140.0 (C_q), 133.0 (2C, CH), 123.4 (2C, CH), 118.6 (C_q), 109.4 (C_q), 98.2 (CH), 48.8 (CH), 45.9 (CH), 23.9 (2C, CH_3), 19.4 (2C, CH_3), 13.7 (CH_3); EI (m/z, 70 eV, 110 °C): 310 (37) $[M]^+$, 211 (15), 210 (100), 182 (42), 167 (28), 155 (27), 141 (17), 114 (11), 102 (18), 100 (25), 58 (18). HRMS ($C_{17}H_{22}N_6$): Calcd 310.1906, Found 310.1904; IR (ATR, ṽ) = 2976 (m), 2928 (m), 2871 (w), 2854 (w), 2225 (m), 1606 (s), 1579 (w), 1550 (w), 1509 (vs), 1458 (m), 1411 (vs), 1377 (vs), 1364 (vs), 1351 (vs), 1312 (s), 1266 (s), 1249 (vs), 1222 (vs), 1179 (s), 1159 (vs), 1120 (vs), 1111 (vs), 1043 (s), 1021 (s), 1010 (s), 976 (s), 953 (m), 942 (m), 925 (m), 905 (m), 877 (m), 851 (vs), 832 (vs), 815 (m), 796 (vs), 756 (m), 744 (m), 722 (w), 708 (w), 688 (w), 679 (w), 666 (w), 647 (w), 620 (w), 608 (w), 571 (s), 561 (vs), 544 (vs), 517 (s), 475 (m), 441 (s), 419 (m), 409 (m), 401 (m), 381 (w), 378 (w) cm^{-1}.

R_f = 0.52 (cyclohexane/ethyl acetate 4:1). ^{1}H NMR (400 MHz, Chloroform-d, ppm) δ = 7.96–7.93 (m, 2H), 7.61–7.58 (m, 2H), 6.01 (s, 1H), 4.96 (sept, *J* = 6.8 Hz, 1H), 3.98 (sept, *J* = 6.7 Hz, 1H), 2.38 (s, 3H), 1.31 (d, *J* = 6.5 Hz, 6H), 1.19 (d, *J* = 6.8 Hz, 6H); ^{13}C NMR (100 MHz, Chloroform-d, ppm) δ = 153.6 (C_q), 150.8 (C_q), 143.7 (C_q), 132.5 (2C, CH), 123.1 (2C, CH), 119.0 (C_q), 108.4 (C_q), 92.9 (CH), 50.0 (CH), 47.6 (CH), 23.8 (2C, CH_3), 19.1 (2C, CH_3), 14.2 (CH_3); EI (*m/z*, 70 eV, 70 °C): 311 (13) $[M+H]^+$, 310 (61) $[M]^+$, 299 (26), 210 (53), 200 (11), 199 (82), 182 (12), 181 (20), 141 (59), 131 (26), 114 (10), 102 (12), 100 (52), 91 (100), 69 (57), 58 (58), 57 (12), 54 (10). HRMS ($C_{17}H_{22}N_6$): Calcd 310.1906. Found 310.1904; IR (ATR, ṽ) = 2972 (w), 2928 (w), 2868 (w), 2225 (w), 1604 (m), 1577 (vw), 1538 (m), 1503 (s), 1463 (w), 1432 (w), 1397 (vs), 1378 (vs), 1353 (vs), 1312 (m), 1276 (vs), 1239 (s), 1217 (s), 1193 (w), 1159 (s), 1132 (vs), 1105 (s), 1095 (m), 1041 (w), 1014 (vs), 972 (w), 958 (w), 929 (w), 907 (w), 884 (w), 843 (vs), 832 (s), 781 (vs), 741 (w), 717 (w), 679 (w), 667 (w), 652 (w), 628 (vw), 616 (w), 557 (s), 548 (vs), 538 (vs), 526 (s), 482 (w), 449 (w), 432 (w), 414 (w), 408 (w), 387 (w) cm^{-1}.

Additional information on the chemical synthesis is available *via* Chemotion repository: https://doi.org/10.14272/reaction/SA-FUHFF-UHFFFADPSC-IIORTUPGIQ-UHFFFADPSC-NUHFF-NXCLB-NUHFF-ZZZ

Additional information on the analysis of the target compound is available *via* Chemotion repository:
https://doi.org/10.14272/RSDVJJOPRTZAOO-XUTLUUPISA-N.1
https://doi.org/10.14272/WPXPVVIOPAJXSL-XUTLUUPISA-N.1

(*E*)-3-(3,3-Diisopropyltriaz-1-en-1-yl)-5-methyl-1-(4-nitrophenyl)-1*H*-pyrazole (17f), (*E*)-5-(3,3-diisopropyltriaz-1-en-1-yl)-3-methyl-1-(4-nitrophenyl)-1*H*-pyrazole (18f)

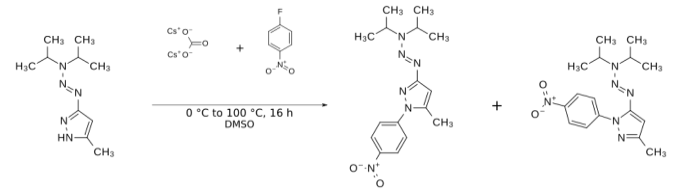

Name {P1|**17f**}: (*E*)-3-(3,3-diisopropyltriaz-1-en-1-yl)-5-methyl-1-(4-nitrophenyl)-1*H*-pyra-zole; Formula: $C_{16}H_{22}N_6O_2$; Smiles: CC(N(C(C)C)/N=N/c1nn(c(c1)C)c1ccc(cc1)[N+](=O)[O-])C. InChIKey: LQZXCDPUXUEILJ-HTXNQAPBSA-N

Name {P2|**18f**}: (*E*)-5-(3,3-diisopropyltriaz-1-en-1-yl)-3-methyl-1-(4-nitrophenyl)-1*H*-pyra-zole; Formula: $C_{16}H_{22}N_6O_2$; Smiles: CC(N(C(C)C)/N=N/c1cc(nn1c1ccc(cc1)[N+](=O)[O-])C)C. InChIKey: VRKHTXPHTAWWEY-HTXNQAPBSA-N

In a vial, (*E*)-3-(3,3-diisopropyltriaz-1-en-1-yl)-5-methyl-1*H*-pyrazole (76.5 mg, 366 µmol, 1.00 equiv) was dissolved in DMSO (3.00 mL) and cesium carbonate (143 mg, 439 µmol, 1.20 equiv) was added. The reaction mixture was cooled to 0 °C and 1-fluoro-4-nitrobenzene (103 mg, 77.6 µL, 731 µmol, 2.00 equiv) was slowly added. After the addition the reaction mixture was slowly warmed to 100 °C and stirred for 16 hours at this temperature. For work up ice-water was added to the reaction mixture. The aqueous phase was extracted three-times with ethyl acetate and the combined organic phase was washed with water and brine, dried over Na_2SO_4 and filtered. As preparation for the column chromatography (dryload), Celite was added and the reaction mixture with Celite was evaporated. The obtained crude product was purified *via* flash-chromatography on silica gel using cyclohexane/ethyl acetate. The isolated product (*E*)-3-(3,3-diisopropyltriaz-1-en-1-yl)-5-methyl-1-(4-nitrophenyl)-1*H*-pyrazole (77.6 mg, 235 µmol, 64% yield) was obtained as an orange solid in 64% yield and the side product (*E*)-5-(3,3-diisopropyltriaz-1-en-1-yl)-3-methyl-1-(4-nitrophenyl)-1*H*-pyrazole (22.7 mg, 68.7 µmol) was obtained as a yellow solid in 19% yield.

R_f = 0.37 (cyclohexane/ethyl acetate 4:1). MP = 103.4–110.5 °C. ^{1}H NMR (400 MHz, Chloroform-d [7.27 ppm], ppm) δ = 8.31–8.29 (m, 2H), 7.77 (d, *J* = 8.4 Hz, 2H), 6.33 (s, 1H), 5.46 (t, *J* = 6.2 Hz, 1H), 4.01 (t, *J* = 6.1 Hz, 1H), 2.49 (s, 3H), 1.38 (d, *J* = 6.1 Hz, 6H), 1.23 (d, *J* = 6.4 Hz, 6H); ^{13}C NMR (100 MHz, Chloroform-d [77.0 ppm], ppm) δ = 163.1 (C_q), 145.1 (C_q), 145.0 (C_q), 140.2 (C_q), 124.6 (2C, CH), 122.8 (2C, CH), 98.6 (CH), 48.8 (CH), 46.1 (CH), 23.8 (2C, CH_3), 19.3 (2C, CH_3), 13.8 (CH_3); EI (m/z, 70 eV, 100 °C): 330 (33) $[M]^+$, 231 (14), 230 (100), 185 (14), 156 (38), 155 (24), 129 (34), 115 (14), 100 (29), 86 (21), 58 (28). HRMS ($C_{16}H_{22}O_2N_6$): Calcd 330.1804, Found 330.1806; IR (ATR, ṽ) = 2975 (w), 2928 (w), 2870 (w), 2837 (w), 1594 (s), 1565 (w), 1513 (vs), 1503 (vs), 1463 (m), 1451 (w), 1415 (vs), 1401 (s), 1367 (s), 1356 (s), 1329 (vs), 1249 (vs), 1221 (vs), 1160 (s), 1126 (vs), 1112 (vs), 1040 (s), 1021 (m), 1011 (m), 980 (s), 924 (w), 907 (m), 878 (m), 846 (vs), 817 (vs), 756 (m), 747 (vs), 718 (w), 707 (m), 688 (s), 662 (w), 632 (m), 619 (m), 606 (w), 557 (m), 543 (m), 526 (s), 516 (s), 494 (m), 432 (m), 425 (m), 404 (m), 384 (m) cm^{-1}.

R_f = 0.50 (cyclohexane/ethyl acetate 4:1). MP = 121.5–126.6 °C. ^{1}H NMR (400 MHz, Chloroform-d [7.27 ppm], ppm) δ = 8.27 (d, J = 9.2 Hz, 2H), 8.10 (d, J = 9.0 Hz, 2H), 6.11 (s, 1H), 5.07 (sept, J = 6.9 Hz, 1H), 4.08 (sept, J = 6.5 Hz, 1H), 2.34 (s, 3H), 1.40 (d, J = 6.6 Hz, 6H), 1.29 (d, J = 6.7 Hz, 6H); ^{13}C NMR (100 MHz, Chloroform-d [77.0 ppm], ppm) δ = 153.9 (C_q), 151.0 (C_q), 145.0 (C_q), 144.6 (C_q), 124.2 (2C, CH), 122.6 (2C, CH), 93.1 (CH), 50.1 (CH), 47.8 (CH), 23.7 (2C, CH_3), 19.1 (2C, CH_3), 14.1 (CH_3); EI (m/z, 70 eV, 90 °C): 331 (23) [M+H]$^+$, 330 (100) [M]$^+$, 231 (14), 230 (89), 202 (38), 185 (13), 161 (18), 156 (13), 131 (21), 100 (62), 69 (15), 58 (48), 54, (13). HRMS ($C_{16}H_{22}O_2N_6$): Calcd 330.1804, Found 330.1805; IR (ATR, ṽ) = 2976 (w), 2962 (w), 2928 (w), 2871 (vw), 1605 (w), 1594 (m), 1543 (w), 1514 (s), 1499 (s), 1462 (w), 1431 (w), 1409 (s), 1392 (vs), 1378 (vs), 1358 (s), 1330 (vs), 1302 (s), 1285 (vs), 1245 (s), 1213 (s), 1170 (w), 1157 (m), 1128 (vs), 1109 (vs), 1096 (s), 1052 (w), 1041 (w), 1011 (vs), 973 (m), 908 (w), 882 (w), 854 (vs), 843 (vs), 820 (m), 781 (vs), 752 (m), 745 (m), 717 (w), 690 (m), 681 (w), 664 (w), 650 (w), 635 (w), 611 (w), 550 (m), 526 (w), 506 (m), 492 (s), 446 (w), 435 (w), 414 (w), 387 (w) cm^{-1}.

Additional information on the chemical synthesis is available *via* Chemotion repository: https://doi.org/10.14272/reaction/SA-FUHFF-UHFFFADPSC-GGOQQTKQMR-UHFFFADPSC-NUHFF-NSUHK-NUHFF-ZZZ

Additional information on the analysis of the target compound is available *via* Chemotion repository:
https://doi.org/10.14272/LQZXCDPUXUEILJ-HTXNQAPBSA-N.1
https://doi.org/10.14272/VRKHTXPHTAWWEY-HTXNQAPBSA-N.1

(*E*)-1-(2-Chloro-4-nitrophenyl)-3-(3,3-diisopropyltriaz-1-en-1-yl)-5-methyl-1*H*-pyrazole (17g), (*E*)-1-(2-chloro-4-nitrophenyl)-5-(3,3-diisopropyltriaz-1-en-1-yl)-3-methyl-1*H*-pyrazole (18g)

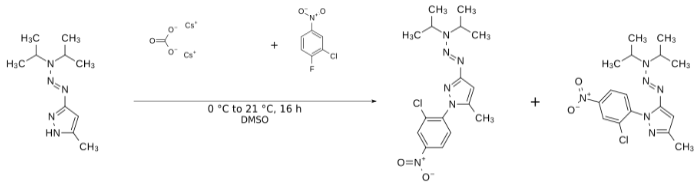

Name {P1|**17g**}: (*E*)-1-(2-chloro-4-nitrophenyl)-3-(3,3-diisopropyltriaz-1-en-1-yl)-5-methyl-1*H*-pyrazole; Formula: $C_{16}H_{21}ClN_6O_2$; Smiles: CC(N(C(C)C)/N=N/c1nn(c(c1)C)c1ccc(cc1Cl)[N+](=O)[O-])C. InChIKey: TYZBFJHLBYOAAU-CZIZESTLSA-N.

Name {P2|**18g**}: (*E*)-1-(2-chloro-4-nitrophenyl)-5-(3,3-diisopropyltriaz-1-en-1-yl)-3-methyl-1*H*-pyrazole; Formula: $C_{16}H_{21}ClN_6O_2$; Smiles: CC(N(C(C)C)/N=N/c1cc(nn1c1ccc(cc1Cl)[N+](=O)[O-])C)C. InChIKey: ACQCSRYTLZFICQ-CZIZESTLSA-N

(*E*)-3-(3,3-Diisopropyltriaz-1-en-1-yl)-5-methyl-1*H*-pyrazole (500 mg, 2.39 mmol, 1.00 equiv) was dissolved in DMSO (24.0 mL) and cesium carbonate (934 mg, 2.87 mmol, 1.20 equiv) was added. The reaction mixture was cooled to 0 °C and 2-chloro-1-fluoro-4-nitrobenzene (839 mg, 4.78 mmol, 2.00 equiv) was slowly added. After the addition, the reaction mixture was slowly warmed to 21 °C and stirred for

16 hours at this temperature. For workup, ice water was added to the reaction mixture. The aqueous phase was extracted three times with ethyl acetate and the combined organic phases were washed with water and brine, dried over Na_2SO_4, filtered and the solvent was removed under reduced pressure. As preparation for column chromatography (dry load), Celite was added (1.5 g) and the reaction mixture with Celite was evaporated. The obtained crude product was purified *via* flash-chromatography (Interchim devices puriFLASH 4125) on silica gel (PF-15SIHP-F0080) using cyclohexane/ethyl acetate with 0% to 20% ethyl acetate in 10 column volumes. The isolated product (*E*)-1-(2-chloro-4-nitrophenyl)-3-(3,3-diisopropyltriaz-1-en-1-yl)-5-methyl-1*H*-pyrazole (530 mg, 1.45 mmol) was obtained as a yellow solid in 61% yield and the side product (*E*)-1-(2-chloro-4-nitrophenyl)-5-(3,3-diisopropyltriaz-1-en-1-yl)-3-methyl-1*H*-pyrazole (80.6 mg, 221 µmol) was obtained as a yellow solid in 9% yield.

R_f = 0.53 (cyclohexane/ethyl acetate 2:1). MP = 138.6–148.2 °C. ^{1}H NMR (400 MHz, Chloroform-d [7.27 ppm], ppm) δ = 8.41 (d, *J* = 2.6 Hz, 1H), 8.23 (dd, *J* = 2.5 Hz, *J* = 8.7 Hz, 1H), 7.67 (d, *J* = 8.8 Hz, 1H), 6.30 (d, *J* = 0.7 Hz, 1H), 5.40 (quint, *J* = 6.3 Hz, 1H), 4.00 (quint, *J* = 6.5 Hz, 1H), 2.20 (d, *J* = 0.6 Hz, 3H), 1.38 (d, *J* = 6.2 Hz, 6H), 1.22 (d, *J* = 6.6 Hz, 6H); ^{13}C NMR (100 MHz, Chloroform-d [77.0 ppm], ppm) δ = 163.5 (C_q), 147.6 (C_q), 143.0 (C_q), 141.7 (C_q), 133.1 (C_q), 130.9 (CH), 125.5 (CH), 122.4 (CH), 95.6 (CH), 48.8 (CH), 45.9 (CH), 23.8 (CH_3, 2C), 19.3 (CH_3 ,2C), 11.8 (CH_3); MS (EI, 70 eV, 110 °C), m/z (%): 364/366 (22/8) $[M]^+$, 293 (16), 264/266 (100/34), 219/221 (27/8), 190/192 (63/21), 163/165 (52/18), 155 (14), 148/150 (10/4), 127 (35), 114 (21), 100 (100), 86 (16), 70 (19), 58 (85). HRMS–EI ($C_{16}H_{21}O_2N_6Cl$) *(m/z)*: $[M]^+$ Calcd 364.1409; Found 364.1408; IR (ATR, ṽ) = 3070 (w), 2975 (w), 2931 (w), 2868 (w), 1587 (w), 1560 (w), 1523 (vs), 1497 (s), 1462 (m), 1445 (w), 1409 (vs), 1377 (vs), 1343 (vs), 1289 (m), 1247 (vs), 1220 (vs), 1156 (vs), 1123 (vs), 1098 (m), 1069 (m), 1038 (s), 1010 (s), 976 (m), 907 (m), 890 (vs), 877 (s), 856 (s), 836 (m), 798 (vs), 773 (vs), 754 (s), 739 (vs), 715 (m), 674 (m), 662 (w), 626 (w), 567 (w), 544 (m), 509 (m), 482 (m), 455 (m), 408 (m) cm^{-1}.

R_f = 0.56 (cyclohexane/ethyl acetate 2:1). MP = 74–77.7 °C. ^{1}H NMR (400 MHz, Chloroform-d [7.27 ppm], ppm) δ = 8.37 (d, *J* = 2.4 Hz, 1H), 8.21 (dd, *J* = 2.4 Hz, *J* = 8.7 Hz, 1H), 7.67 (d, *J* = 8.7 Hz, 1H), 6.07 (s, 1H), 4.82 (sept, *J* = 6.8 Hz, 1H), 3.98 (sept, *J* = 6.5 Hz, 1H), 2.34 (s, 3H), 1.30 (d, *J* = 6.6 Hz, 6H), 1.13 (d, *J* = 6.7 Hz, 6H); ^{13}C NMR (100 MHz, Chloroform-d [77.0 ppm], ppm) δ = 154.0 (C_q), 151.1 (C_q), 147.2 (C_q), 143.0 (C_q), 133.1 (CH), 130.4 (CH), 125.4 (CH), 122.0 (CH), 91.9 (CH), 50.2 (CH), 47.3 (CH), 23.5 (CH_3, 2C), 19.0 (CH_3, 2C), 14.1 (CH_3). MS (EI, 70 eV, 90 °C), m/z (%): 364/366 (100/35) $[M]^+$, 264/266 (85/30), 236/238 (24/7), 190/192 (37/13), 114 (15), 100 (91), 58 (53). HRMS–EI ($C_{16}H_{21}O_2N_6Cl$) *(m/z)*: $[M]^+$ Calcd 364.1409; Found 364.1411; IR (ATR, ṽ) = 3075 (w), 2975 (w), 2929 (w), 2870 (w), 1584 (w), 1520 (vs), 1499 (vs), 1469 (m), 1408 (vs), 1378 (s), 1360 (vs), 1343 (vs), 1296 (s), 1259 (s), 1245 (s), 1218 (s), 1160 (m), 1143 (s), 1125 (vs), 1120 (vs), 1096 (m), 1079 (m), 1037 (m), 1017 (vs), 975 (w), 926 (w), 904 (m), 891 (m), 875 (w), 840 (s), 790 (vs), 768 (vs), 751 (m), 734 (vs), 713 (w), 681 (m), 664 (w), 650 (w), 626 (w), 612 (w), 550 (m), 528 (m), 513 (m), 475 (m), 450 (w), 431 (w), 407 (m), 387 (w) cm^{-1}.

Additional information on the chemical synthesis is available *via* Chemotion repository: https://doi.org/10.14272/reaction/SA-FUHFF-UHFFFADPSC-YQOQMYLZHR-UHFFFADPSC-NUHFF-NODPP-NUHFF-ZZZ

Additional information on the analysis of the target compound is available *via* Chemotion repository:
https://doi.org/10.14272/TYZBFJHLBYOAAU-CZIZESTLSA-N.1
https://doi.org/10.14272/ACQCSRYTLZFICQ-CZIZESTLSA-N.1

Ethyl (*E*)-1-benzyl-3-(3,3-diisopropyltriaz-1-en-1-yl)-1*H*-pyrazole-4-carboxylate (17h), ethyl (*E*)-1-benzyl-5-(3,3-diisopropyltriaz-1-en-1-yl)-1*H*-pyrazole-4-carboxylate (18h)

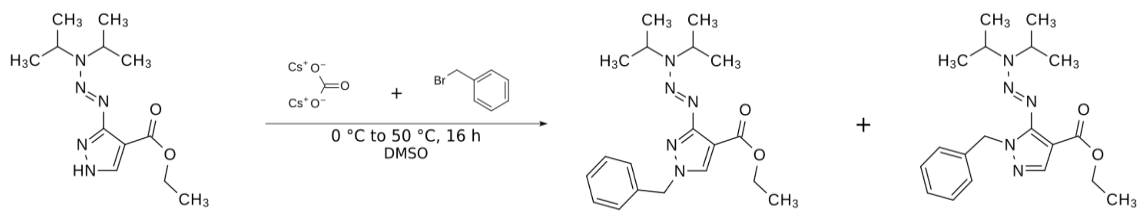

Name {P1|**17h**}: ethyl (*E*)-1-benzyl-3-(3,3-diisopropyltriaz-1-en-1-yl)-1*H*-pyrazole-4-carboxylate; Formula: $C_{19}H_{27}N_5O_2$; Smiles: CCOC(=O)c1cn(nc1/N=N/N(C(C)C)C(C)C)Cc1ccccc1. InChIKey: GKODORMDVGUGHL-LSDHQDQOSA-N

Name {P2|**18h**}: ethyl (*E*)-1-benzyl-5-(3,3-diisopropyltriaz-1-en-1-yl)-1*H*-pyrazole-4-carboxylate; Formula: $C_{19}H_{27}N_5O_2$; Smiles: CCOC(=O)c1cnn(c1/N=N/N(C(C)C)C(C)C)Cc1ccccc1. InChIKey: UBBFWZTUXPGXCP-QURGRASLSA-N

In a vial, ethyl (*E*)-3-(3,3-diisopropyltriaz-1-en-1-yl)-1*H*-pyrazole-4-carboxylate (300 mg, 1.12 mmol, 1.00 equiv) was dissolved in 50 mL of DMSO and the solution was cooled to 0 °C. Cesium Carbonate (439 mg, 1.35 mmol, 1.20 equiv) and bromomethylbenzene (384 mg, 267 µL, 2.24 mmol, 2.00 equiv) were added and the mixture was stirred first at 21 °C for 2 hours, then at 50 °C for 14 hours. The reaction was quenched with ice water and extracted with ethyl acetate (3 x 50 mL). The combined organic layers were washed with brine and dried over Na_2SO_4 and filtered. As preparation for the column chromatography (dryload), Celite was added and the reaction mixture with Celite was evaporated. The obtained crude product was purified *via* flash-chromatography on silica gel using cyclohexane/ethyl acetate 10:1 to 4:1. The isolated product ethyl (*E*)-1-benzyl-3-(3,3-diisopropyltriaz-1-en-1-yl)-1*H*-pyrazole-4-carboxylate (176 mg, 493 µmol) was obtained as a colorless solid in 44% yield and the side product ethyl (*E*)-1-benzyl-5-(3,3-diisopropyltriaz-1-en-1-yl)-1*H*-pyrazole-4-carboxylate (161 mg, 450 µmol) was obtained as a beige solid in 40% yield.

R_f = 0.04 (cyclohexane/ethyl acetate 10:1). MP = 98.4–98.9 °C. ^{1}H NMR (400 MHz, Chloroform-d [7.27 ppm], ppm) δ = 7.63 (s, 1H), 7.31–7.20 (m, 5H), 5.17 (s, 2H), 5.06–4.99 (m, 1H), 4.14 (q, *J* = 7.2 Hz, 2H), 4.06–3.97 (m, 1H), 1.30 (d, *J* = 6.5 Hz, 6H), 1.25 (d, *J* = 6.6 Hz, 6H), 1.20 (t, *J* = 7.2 Hz, 3H); ^{13}C NMR (100 MHz, Chloroform-d [77.0 ppm], ppm) δ = 163.1 (C_q), 159.7 (C_q), 135.3 (C_q), 134.0 (CH), 128.8 (CH, 2C), 128.3 (CH), 128.2 (CH, 2C), 106.5 (C_q), 59.8 (CH_2), 56.4 (CH_2), 50.4 (CH), 46.6 (CH), 23.2 (CH_3, 2C), 19.0 (CH_3, 2C), 14.4 (CH_3). MS (FAB, 3-NBA): *m/z* (%) = 359 (22) $[M+2H]^+$, 358 (100) $[M+H]^+$, 357 (16) $[M]^+$, 312 (14), 257 (49), 100 (27), 99 (12), 91 (57), 69 (10), 57 (13), 55 (12). HRMS–FAB *(m/z)*: $[M+H]^+$ Calcd for $C_{19}H_{28}O_2N_5$, 358.2243; Found, 358.2241; IR (ATR, ṽ) = 3114 (w), 3067 (w), 2983 (w), 2972 (w), 2931 (w), 2871 (w),

1683 (vs), 1643 (w), 1605 (vw), 1544 (s), 1496 (w), 1485 (w), 1466 (m), 1451 (s), 1404 (vs), 1374 (s), 1363 (s), 1346 (m), 1334 (w), 1327 (m), 1302 (w), 1268 (s), 1238 (vs), 1221 (vs), 1152 (vs), 1136 (m), 1122 (vs), 1103 (vs), 1077 (m), 1030 (s), 1024 (s), 989 (m), 963 (w), 928 (w), 905 (w), 892 (w), 873 (w), 863 (w), 820 (w), 781 (vs), 771 (m), 732 (vs), 718 (s), 705 (m), 696 (m), 652 (w), 635 (w), 623 (w), 582 (w), 562 (m), 551 (m), 528 (w), 490 (w), 462 (m), 442 (w), 421 (w), 407 (w), 391 (w), 378 (w) cm^{-1}.

R_f = 0.16 (cyclohexane/ethyl acetate 10:1). MP = 57.7–59.3 °C. ^{1}H NMR (400 MHz, Chloroform-d [7.27 ppm], ppm) δ = 7.86 (s, 1H), 7.30–7.20 (m, 3H), 7.17–7.15 (m, 2H), 5.32 (s, 2H), 5.19 (quint, *J* = 6.8 Hz, 1H), 4.23 (q, *J* = 7.1 Hz, 2H), 4.01 (quint, *J* = 6.6 Hz, 1H), 1.32–1.25 (m, 15H); ^{13}C NMR (100 MHz, Chloroform-d [77.0 ppm], ppm) δ = 163.2 (C_q), 151.9 (C_q), 141.6 (CH), 136.9 (C_q), 128.4 (CH, 2C), 127.4 (CH), 127.2 (CH, 2C), 102.9 (C_q), 59.5 (CH_2), 52.4 (CH_2), 50.2 (CH), 47.1 (CH), 23.4 (CH_3, 2C), 18.9 (CH_3, 2C), 14.4 (CH_3); MS (FAB, 3-NBA): *m/z* (%) = 359 (21) $[M+2H]^+$, 358 (100) $[M+H]^+$, 357 (12) $[M]^+$, 312 (28), 257 (13), 229 (21), 111 (10), 109 (15), 100 (25), 97 (21), 95 (23), 93 (10), 91 (64). HRMS–FAB *(m/z)*: $[M+H]^+$ Calcd for $C_{19}H_{28}O_2N_5$, 358.2243; Found, 358.2241; IR (ATR, ṽ) = 3114 (vw), 3088 (vw), 3063 (vw), 3033 (vw), 2975 (w), 2934 (w), 2905 (w), 2871 (w), 1715 (vs), 1698 (vs), 1606 (w), 1587 (w), 1538 (s), 1496 (w), 1470 (s), 1455 (m), 1407 (vs), 1383 (vs), 1361 (vs), 1279 (s), 1247 (vs), 1214 (vs), 1177 (vs), 1156 (vs), 1129 (s), 1096 (vs), 1075 (s), 1030 (vs), 1001 (m), 946 (w), 928 (w), 908 (m), 884 (m), 868 (m), 837 (m), 775 (s), 730 (vs), 718 (s), 696 (s), 653 (w), 605 (m), 575 (m), 561 (m), 540 (w), 487 (w), 459 (w), 426 (w), 411 (w), 395 (w) cm^{-1}.

Additional information on the chemical synthesis is available *via* Chemotion repository: https://doi.org/10.14272/reaction/SA-FUHFF-UHFFFADPSC-GWVLJZRVEA-UHFFFADPSC-NUHFF-NMXFL-NUHFF-ZZZ

Additional information on the analysis of the target compound is available *via* Chemotion repository:
https://doi.org/10.14272/GKODORMDVGUGHL-LSDHQDQOSA-N.1
https://doi.org/10.14272/UBBFWZTUXPGXCP-QURGRASLSA-N.1

Ethyl (*E*)-1-(3,5-difluorobenzyl)-3-(3,3-diisopropyltriaz-1-en-1-yl)-1*H*-pyrazole-4-carboxylate (17i), ethyl (*E*)-1-(3,5-difluorobenzyl)-5-(3,3-diisopropyltriaz-1-en-1-yl)-1*H*-pyrazole-4-carboxylate (18i)

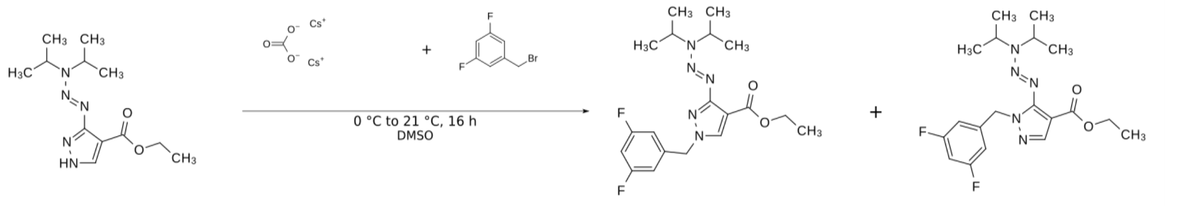

Name {P1|**17i**}: ethyl (*E*)-1-(3,5-difluorobenzyl)-3-(3,3-diisopropyltriaz-1-en-1-yl)-1*H*-pyrazole-4-carboxylate; Formula: $C_{19}H_{25}F_2N_5O_2$; Smiles: CCOC(=O)c1cn(nc1/N=N/N(C(C)C)C(C)C)Cc1cc(F)cc(c1)F. InChIKey: HJNVDSRILWJTQV-ZNTNEXAZSA-N

Name {P2|**18i**}: ethyl (*E*)-1-(3,5-difluorobenzyl)-5-(3,3-diisopropyltriaz-1-en-1-yl)-1*H*-pyrazole-4-carboxylate; Formula: $C_{19}H_{25}F_2N_5O_2$; Smiles:

CCOC(=O)c1cnn(c1/N=N/N(C(C)C)C(C)C)Cc1cc(F)cc(c1)F. InChIKey: XCKSGKZXEXJBOY-WCWDXBQESA-N

Ethyl (*E*)-3-(3,3-diisopropyltriaz-1-en-1-yl)-1*H*-pyrazole-4-carboxylate (500 mg, 1.87 mmol, 1.00 equiv) was dissolved in DMSO (19.0 mL) and cesium carbonate (731 mg, 2.24 mmol, 1.20 equiv) was added. The reaction mixture was cooled to 0 °C and 1-(bromomethyl)-3,5-difluorobenzene (774 mg, 3.74 mmol, 2.00 equiv) was slowly added. After the addition, the reaction mixture was slowly warmed to 21 °C and stirred for 16 hours at this temperature. For workup, ice water was added to the reaction mixture. The aqueous phase was extracted three times with ethyl acetate and the combined organic phase was washed with water and brine, dried over Na_2SO_4, filtered and the solvent was removed under reduced pressure. As preparation for column chromatography (dry load), Celite was added (1.5 g) and the reaction mixture with Celite was evaporated. The obtained crude product was purified *via* flash-chromatography (Interchim devices puriFLASH 4125) on silica gel (PF-15SIHP-F0080) using cyclohexane/ethyl acetate with 0% to 34% ethyl acetate in 15 column volumes. The isolated product ethyl (*E*)-1-(3,5-difluorobenzyl)-3-(3,3-diisopropyltriaz-1-en-1-yl)-1*H*-pyrazole-4-carboxylate (270 mg, 687 µmol) was obtained as a light yellow solid in 37% yield and the side product ethyl (*E*)-1-(3,5-difluorobenzyl)-5-(3,3-diisopropyltriaz-1-en-1-yl)-1*H*-pyrazole-4-carboxylate (461 mg, 1.17 mmol) was obtained as a colorless oil in 63% yield.

R_f = 0.67 (cyclohexane/ethyl acetate 1:1). MP = 106.1–114.6 °C. ^{1}H NMR (400 MHz, Chloroform-d [7.27 ppm], ppm) δ = 7.80 (s, 1H), 6.81–6.73 (m, 3H), 5.22 (s, 2H), 5.14 (quint, *J* = 6.5 Hz, 1H), 4.25 (q, *J* = 7.1 Hz, 2H), 4.13–4.05 (m, 1H), 1.38–1.29 (m, 15H); ^{13}C NMR (100 MHz, Chloroform-d [77.0 ppm], ppm) δ = 163.2 (dd, *J* = 12.3 Hz, *J* = 249.7 Hz, C_q, 2C), 162.9 (C_q), 160.1 (C_q), 139.5 (t, *J* = 8.9 Hz, C_q), 134.5 (CH), 110.7 (dd, *J* = 7.7 Hz, *J* = 19.3 Hz, CH, 2C), 107.1 (C_q), 103.7 (t, *J* = 25.4 Hz, CH), 60.0 (CH_2), 55.3 (CH_2), 50.5 (CH), 46.8 (CH), 23.3 (CH_3, 2C), 19.0 (CH_3, 2C), 14.4 (CH_3); ^{19}F NMR (376 MHz, ppm) δ = -108.68; MS (EI, 70 eV, 110 °C), m/z (%): 393 (17) $[M]^+$, 293 (100), 237 (12), 140 (11), 127 (84), 100 (42), 58 (12). HRMS–EI ($C_{19}H_{25}O_2N_5F_2$) *(m/z)*: $[M]^+$ Calcd 393.1971; Found 393.1969; IR (ATR, ṽ) = 3122 (w), 2979 (w), 2968 (w), 2932 (w), 1676 (vs), 1623 (m), 1596 (m), 1538 (vs), 1460 (m), 1448 (m), 1415 (vs), 1368 (s), 1358 (s), 1343 (vs), 1317 (vs), 1259 (vs), 1241 (m), 1218 (vs), 1171 (s), 1157 (s), 1146 (s), 1118 (vs), 1096 (vs), 1044 (s), 1023 (s), 1007 (s), 997 (s), 977 (m), 946 (m), 922 (w), 891 (w), 870 (s), 854 (vs), 822 (w), 779 (vs), 762 (vs), 738 (w), 720 (w), 708 (s), 671 (w), 639 (vs), 608 (m), 579 (w), 547 (w), 534 (m), 521 (m), 509 (m), 449 (w), 401 (w), 382 (w) cm^{-1}.

R_f = 0.80 (cyclohexane/ethyl acetate 1:1). ^{1}H NMR (400 MHz, Chloroform-d [7.27 ppm], ppm) δ = 7.86 (s, 1H), 6.68–6.64 (m, 3H), 5.27 (s, 2H), 5.16 (sept, *J* = 6.9 Hz, 1H), 4.23 (q, *J* = 7.1 Hz, 2H), 4.02 (sept, *J* = 6.6 Hz, 1H), 1.32–1.24 (m, 15H); ^{13}C NMR (100 MHz, Chloroform-d [77.0 ppm], ppm) δ = 163.0 (dd, *J* = 249.1, 12.6 Hz, C_q, 2C), 163.0 (C_q), 151.9 (C_q), 141.9 (CH), 140.9 (t, *J* = 9.2 Hz, C_q), 110.1 (dd, *J* = 6.9 Hz, *J* = 18.5 Hz, CH, 2C), 103.9 (C_q), 102.9 (t, *J* = 25.3 Hz, CH), 59.7 (CH_2), 51.6 (t, *J* = 1.9 Hz, CH_2), 50.3 (CH), 47.3 (CH), 23.3 (CH_3, 2C), 18.8 (CH_3, 2C), 14.4 (CH_3); ^{19}F NMR (376 MHz, ppm) δ = -109.50; MS (FAB, 3-NBA), m/z (%): 394 (100) $[M+1]^+$, 348 (28), 293 (16), 265 (24), 127 (28), 100 (30), 95 (11). HRMS–FAB ($C_{19}H_{26}O_2N_5F_2$) *(m/z)*: $[M+H]^+$ Calcd 394.2049; Found 394.2048; IR (ATR, ṽ) = 3106 (vw), 3080 (vw), 2976 (w), 2944 (w), 2908 (w), 2871 (w), 1680 (vs), 1626 (m), 1595 (m), 1536 (m), 1470 (s), 1463 (vs), 1445 (s), 1425 (s), 1414 (vs), 1397 (vs), 1383 (vs), 1353 (vs), 1310 (s), 1255 (vs), 1215 (vs), 1184 (vs), 1154 (s), 1132 (m), 1118 (vs), 1109 (vs), 1096 (vs), 1034

(vs), 989 (vs), 973 (s), 952 (w), 936 (s), 904 (m), 898 (m), 885 (m), 871 (w), 853 (vs), 837 (vs), 775 (vs), 720 (w), 703 (w), 681 (s), 662 (w), 632 (s), 602 (s), 560 (m), 554 (m), 537 (w), 511 (s), 475 (w), 422 (w), 405 (w), 394 (w) cm^{-1}.

Additional information on the chemical synthesis is available *via* Chemotion repository: https://doi.org/10.14272/reaction/SA-FUHFF-UHFFFADPSC-TWYQGEODAN-UHFFFADPSC-NUHFF-NQYFZ-NUHFF-ZZZ

Additional information on the analysis of the target compound is available *via* Chemotion repository:
https://doi.org/10.14272/HJNVDSRILWJTQV-ZNTNEXAZSA-N.1
https://doi.org/10.14272/XCKSGKZXEXJBOY-WCWDXBQESA-N.1

Ethyl (*E*)-1-(3-cyanobenzyl)-3-(3,3-diisopropyltriaz-1-en-1-yl)-1*H*-pyrazole-4-carboxylate (**17j**), **ethyl (*E*)-1-(3-cyanobenzyl)-5-(3,3-diisopropyltriaz-1-en-1-yl)-1*H*-pyrazole-4-carboxylate** (**18j**)

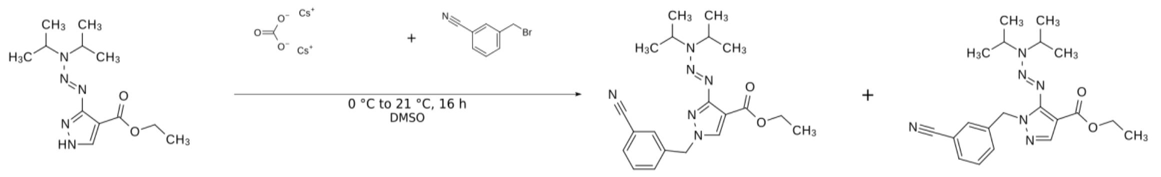

Name {P1|**17j**}: ethyl (*E*)-1-(3-cyanobenzyl)-3-(3,3-diisopropyltriaz-1-en-1-yl)-1*H*-pyrazole-4-carboxylate; Formula: $C_{20}H_{26}N_6O_2$; Smiles: CCOC(=O)c1cn(nc1/N=N/N(C(C)C)C(C)C)Cc1cccc(c1)C#N. InChIKey: NUENLEYWZUNRJY-ZNTNEXAZSA-N

Name {P2|**18j**}: ethyl (*E*)-1-(3-cyanobenzyl)-5-(3,3-diisopropyltriaz-1-en-1-yl)-1*H*-pyrazole-4-carboxylate; Formula: $C_{20}H_{26}N_6O_2$; Smiles: CCOC(=O)c1cnn(c1/N=N/N(C(C)C)C(C)C)Cc1cccc(c1)C#N. InChIKey: WTPBPRNMORPMPY-WCWDXBQESA-N

Ethyl (*E*)-3-(3,3-diisopropyltriaz-1-en-1-yl)-1*H*-pyrazole-4-carboxylate (500 mg, 1.87 mmol, 1.00 equiv) was dissolved in DMSO (19.0 mL) and cesium carbonate (731 mg, 2.24 mmol, 1.20 equiv) was added. The reaction mixture was cooled to 0 °C and 3-(bromomethyl)benzonitrile (733 mg, 3.74 mmol, 2.00 equiv) was slowly added. After the addition, the reaction mixture was slowly warmed to 21 °C and stirred for 16 hours at this temperature. For workup, ice water was added to the reaction mixture. The aqueous phase was extracted three times with ethyl acetate and the combined organic phases were washed with water and brine, dried over Na_2SO_4, filtered and the solvent was removed under reduced pressure. As preparation for column chromatography (dry load), Celite was added (1.5 g) and the reaction mixture with Celite was evaporated. The obtained crude product was purified *via* flash-chromatography (Interchim devices puriFLASH 4125) on silica gel (PF-15SIHP-F0080) using cyclohexane/ethyl acetate with 0% to 50% ethyl acetate in 15 column volumes. The isolated product ethyl (*E*)-1-(3-cyanobenzyl)-3-(3,3-diisopropyltriaz-1-en-1-yl)-1*H*-pyrazole-4-carboxylate (252 mg, 658 µmol) was obtained as colorless solid in 35% yield and the side product ethyl (*E*)-1-(3-cyanobenzyl)-5-(3,3-diisopropyltriaz-1-en-1-

yl)-1*H*-pyrazole-4-carboxylate (463 mg, 1.21 mmol) was obtained as a yellow oil in 65% yield.

R_f = 0.35 (cyclohexane/ethyl acetate 1:1). MP = 99.3–104.3 °C. ^{1}H NMR (400 MHz, Chloroform-d [7.27 ppm], ppm) δ = 7.81 (s, 1H), 7.60 (dt, *J* = 7.2, 1.7 Hz, 1H), 7.57–7.53 (m, 1H), 7.51–7.44 (m, 2H), 5.26 (s, 2H), 5.13 (sept, *J* = 6.9 Hz, 1H), 4.24 (q, *J* = 7.1 Hz, 2H), 4.14–4.03 (m, 1H), 1.37–1.28 (m, 15H). ^{13}C NMR (100 MHz, Chloroform-d [77.0 ppm], ppm) δ = 162.9 (C_q), 160.2 (C_q), 137.4 (C_q), 134.4 (CH), 132.0 (CH), 131.8 (CH), 131.1 (CH), 129.6 (CH), 118.2 (C_q), 113.0 (C_q), 107.1 (C_q), 59.9 (CH_2), 55.3 (CH_2), 50.4 (CH), 46.7 (CH), 23.2 (CH_3, 2C), 19.0 (CH_3 ,2C), 14.4 (CH_3). MS (EI, 70 eV, 140 °C), m/z (%): 382 (21) $[M]^+$, 282 (100), 226 (17), 210 (14), 129 (10), 116 (92), 100 (43), 89 (11), 58 (11). HRMS–EI ($C_{20}H_{26}O_2N_6$) *(m/z)*: $[M]^+$ Calcd 382.2112; Found 382.2113; IR (ATR, ṽ) = 3112 (vw), 3065 (vw), 2980 (w), 2941 (w), 2931 (w), 2905 (vw), 2870 (vw), 2228 (w), 1684 (vs), 1547 (m), 1483 (w), 1458 (w), 1441 (w), 1429 (w), 1400 (vs), 1384 (vs), 1368 (s), 1350 (s), 1336 (w), 1273 (s), 1245 (vs), 1215 (s), 1154 (vs), 1123 (vs), 1108 (vs), 1096 (vs), 1028 (s), 1009 (m), 999 (w), 925 (w), 899 (w), 874 (w), 863 (w), 817 (m), 796 (m), 781 (vs), 759 (w), 727 (w), 708 (w), 686 (s), 630 (m), 572 (w), 557 (m), 534 (w), 443 (m), 418 (w) cm^{-1}.

R_f = 0.52 (cyclohexane/ethyl acetate 1:1). ^{1}H NMR (400 MHz, Chloroform-d [7.27 ppm], ppm) δ = 7.84 (s, 1H), 7.54–7.49 (m, 1H), 7.44–7.32 (m, 3H), 5.31 (s, 2H), 5.14 (sept, *J* = 6.8 Hz, 1H), 4.21 (q, *J* = 7.2 Hz, 2H), 4.01 (sept, *J* = 6.6 Hz, 1H), 1.30–1.23 (m, 15H); ^{13}C NMR (100 MHz, Chloroform-d [77.0 ppm], ppm) δ = 162.9 (C_q), 151.8 (C_q), 141.9 (CH), 138.5 (C_q), 131.6 (CH), 131.1 (CH), 130.7 (CH), 129.3 (CH), 118.3 (C_q), 112.5 (C_q), 103.0 (C_q), 59.6 (CH_2), 51.4 (CH_2), 50.3 (CH), 47.3 (CH), 23.3 (CH_3, 2C), 18.8 (CH_3, 2C), 14.3 (CH_3); MS (FAB, 3-NBA), m/z (%): 383 (100) $[M+1]^+$, 337 (21), 282 (11), 208 (11), 116 (14), 100 (19). HRMS–FAB ($C_{20}H_{27}O_2N_6$) *(m/z)*: $[M+H]^+$ Calcd 383.2190; Found 383.2188; IR (ATR, ṽ) = 2976 (w), 2935 (w), 2907 (w), 2873 (w), 2230 (w), 1714 (vs), 1698 (vs), 1605 (w), 1585 (w), 1540 (s), 1470 (s), 1407 (vs), 1381 (vs), 1361 (vs), 1319 (m), 1300 (m), 1279 (s), 1249 (vs), 1214 (vs), 1181 (vs), 1157 (vs), 1130 (s), 1099 (vs), 1031 (vs), 1001 (m), 946 (m), 909 (m), 882 (m), 836 (m), 776 (vs), 730 (s), 687 (s), 649 (w), 623 (w), 588 (m), 561 (m), 554 (m), 487 (w), 479 (w), 462 (w), 443 (w), 429 (w), 409 (w), 397 (w), 385 (w), 378 (w) cm^{-1}.

Additional information on the chemical synthesis is available *via* Chemotion repository: https://doi.org/10.14272/reaction/SA-FUHFF-UHFFFADPSC-XLLCQXNBYD-UHFFFADPSC-NUHFF-NQYFZ-NUHFF-ZZZ

Additional information on the analysis of the target compound is available *via* Chemotion repository:
https://doi.org/10.14272/NUENLEYWZUNRJY-ZNTNEXAZSA-N.1
https://doi.org/10.14272/WTPBPRNMORPMPY-WCWDXBQESA-N.1

Ethyl (*E*)-1-(4-bromobenzyl)-3-(3,3-diisopropyltriaz-1-en-1-yl)-1*H*-pyrazole-4-carboxylate (**17k**), **ethyl (*E*)-1-(4-bromobenzyl)-5-(3,3-diisopropyltriaz-1-en-1-yl)-1*H*-pyrazole-4-carboxylate** (**18k**)

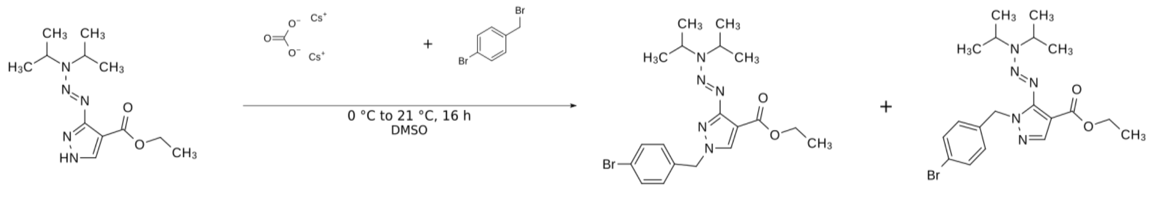

Name {P1|**17k**}: ethyl (*E*)-1-(4-bromobenzyl)-3-(3,3-diisopropyltriaz-1-en-1-yl)-1*H*-pyrazole-4-carboxylate; Formula: $C_{19}H_{26}BrN_5O_2$; Smiles: CCOC(=O)c1cn(nc1/N=N/N(C(C)C)C(C)C)Cc1ccc(cc1)Br. InChIKey: ZYMNXIBCZDWSHX-XTQSDGFTSA-N

Name {P2|**18k**}: ethyl (*E*)-1-(4-bromobenzyl)-5-(3,3-diisopropyltriaz-1-en-1-yl)-1*H*-pyrazole-4-carboxylate; Formula: $C_{19}H_{26}BrN_5O_2$; Smiles: CCOC(=O)c1cnn(c1/N=N/N(C(C)C)C(C)C)Cc1ccc(cc1)Br. InChIKey: FFWKUULMXDRWPI-GHVJWSGMSA-N

Ethyl (*E*)-3-(3,3-diisopropyltriaz-1-en-1-yl)-1*H*-pyrazole-4-carboxylate (500 mg, 1.87 mmol, 1.00 equiv) was dissolved in DMSO (19.0 mL) and cesium carbonate (731 mg, 2.24 mmol, 1.20 equiv) was added. The reaction mixture was cooled to 0 °C and 1-bromo-4-(bromomethyl)benzene (935 mg, 3.74 mmol, 2.00 equiv) was slowly added. After the addition, the reaction mixture was slowly warmed to 21 °C and stirred for 16 hours at this temperature. For work-up, ice water was added to the reaction mixture. The aqueous phase was extracted three times with ethyl acetate and the combined organic phase was washed with water and brine, dried over Na_2SO_4 and filtered. As preparation for the column chromatography (dryload), Celite was added (1.5 g) and the reaction mixture with Celite was evaporated. The obtained crude product was purified *via* flash-chromatography (Interchim devices puriFLASH 5.125) on silica gel (PF-15SIHP-F0080) using cyclohexane/ethyl acetate 0% to 40% ethyl acetate in 8 column volumes (1 column volume = 173.2 mL; flow: 34 mL/min). The isolated product ethyl (*E*)-1-(4-bromobenzyl)-3-(3,3-diisopropyltriaz-1-en-1-yl)-1*H*-pyrazole-4-carboxylate (338 mg, 775 µmol) was obtained as a colorless solid in 41% yield and the side product ethyl (*E*)-1-(4-bromobenzyl)-5-(3,3-diisopropyltriaz-1-en-1-yl)-1*H*-pyrazole-4-carboxylate (485 mg, 1.11 mmol) was obtained as a colorless oil in 59% yield.

R_f = 0.27 (cyclohexane/ethyl acetate 2:1). MP = 92.7–106.6 °C. ^{1}H NMR (400 MHz, Chloroform-d [7.26 ppm], ppm) δ = 7.71 (s, 1H), 7.49–7.45 (m, 2H), 7.16–7.13 (m, 2H), 5.18 (s, 2H), 5.14–5.08 (m, 1H), 4.22 (q, *J* = 7.1 Hz, 2H), 4.11–4.04 (m, 1H), 1.36 (d, *J* = 6.6 Hz, 6H), 1.31 (d, *J* = 6.8 Hz, 6H), 1.28 (t, *J* = 7.1 Hz, 3H). ^{13}C NMR (100 MHz, Chloroform-d [77.0 ppm], ppm) δ = 163.0 (C_q), 159.8 (C_q), 134.4 (C_q), 134.1 (CH), 132.0 (CH, 2C), 129.7 (CH, 2C), 122.4 (C_q), 106.8 (C_q), 59.9 (CH_2), 55.6 (CH_2), 50.5 (CH), 46.7 (CH), 23.2 (CH_3, 2C), 19.0 (CH_3, 2C), 14.4 (CH_3); MS (EI, 70 eV, 130 °C), m/z (%): 435/437 (4/4) $[M]^+$, 335/337 (26/24), 308/310 (21/22), 264/266 (15/14), 169/171 (100/95), 100 (56), 90 (41), 58 (21). HRMS–EI ($C_{19}H_{26}O_2N_5Br$) *(m/z)*: $[M]^+$ Calcd 435.1264; Found 435.1263; IR (ATR, ṽ) = 3119 (w), 3072 (w), 2983 (w), 2970 (w), 2929 (w), 2868 (w), 1681 (vs), 1545 (vs), 1489 (m), 1466 (m), 1452 (s), 1448 (s),

1404 (vs), 1378 (s), 1361 (vs), 1347 (s), 1324 (m), 1272 (vs), 1241 (vs), 1220 (vs), 1163 (vs), 1152 (vs), 1123 (vs), 1105 (vs), 1098 (vs), 1072 (vs), 1028 (s), 1023 (s), 1014 (vs), 992 (s), 963 (m), 936 (w), 926 (w), 891 (m), 861 (w), 841 (m), 813 (s), 803 (vs), 779 (vs), 761 (s), 720 (m), 707 (m), 691 (w), 652 (w), 623 (m), 609 (w), 557 (s), 533 (m), 510 (w), 482 (vs), 450 (w), 415 (w), 377 (m) cm^{-1}.

R_f = 0.45 (cyclohexane/ethyl acetate 2:1). ^1H NMR (400 MHz, Chloroform-d [7.27 ppm], ppm) δ = 7.85 (s, 1H), 7.41–7.37 (m, 2H), 7.05–7.01 (m, 2H), 5.26 (s, 2H), 5.16 (sept, *J* = 6.8 Hz, 1H), 4.23 (q, *J* = 7.1 Hz, 2H), 4.01 (sept, *J* = 6.7 Hz, 1H), 1.31–1.24 (m, 15H); ^{13}C NMR (100 MHz, Chloroform-d [77.0 ppm], ppm) δ = 163.1 (C_q), 151.8 (C_q), 141.7 (CH), 136.0 (C_q), 131.5 (CH, 2C), 128.9 (CH, 2C), 121.3 (C_q), 102.9 (C_q), 59.6 (CH_2), 51.7 (CH_2), 50.3 (CH), 47.2 (CH), 23.4 (CH_3, 2C), 18.9 (CH_3, 2C), 14.4 (CH_3); MS (FAB, 3-NBA), m/z (%): 436/438 (100/92) $[M+1]^+$, 390/392 (28/27), 335/337 (14/15), 307/309 (20/20), 169/171 (56/53), 100 (61). HRMS–FAB ($C_{19}H_{27}O_2N_5Br$) *(m/z)*: $[M+H]^+$ Calcd 436.1343; Found 436.1341; IR (ATR, ṽ) = 2975 (w), 2932 (w), 2905 (w), 2873 (w), 1717 (vs), 1698 (s), 1592 (w), 1538 (s), 1487 (s), 1470 (s), 1405 (vs), 1381 (vs), 1361 (vs), 1281 (s), 1247 (vs), 1214 (vs), 1177 (vs), 1157 (vs), 1129 (s), 1095 (vs), 1071 (s), 1030 (vs), 1011 (vs), 953 (m), 926 (m), 908 (m), 882 (m), 870 (m), 836 (s), 802 (s), 776 (vs), 727 (w), 698 (w), 656 (w), 625 (m), 599 (m), 561 (m), 535 (w), 494 (m), 477 (m) cm^{-1}.

Additional information on the chemical synthesis is available *via* Chemotion repository: https://doi.org/10.14272/reaction/SA-FUHFF-UHFFFADPSC-VJXASIWJXV-UHFFFADPSC-NUHFF-NCPJL-NUHFF-ZZZ

Additional information on the analysis of the target compound is available *via* Chemotion repository:
https://doi.org/10.14272/ZYMNXIBCZDWSHX-XTQSDGFTSA-N.1
https://doi.org/10.14272/FFWKUULMXDRWPI-GHVJWSGMSA-N.1

(*E*)-3-(3,3-Diisopropyltriaz-1-en-1-yl)-1-isopropyl-1*H*-pyrazole-4-carbonitrile (17l), (*E*)-5-(3,3-diisopropyltriaz-1-en-1-yl)-1-isopropyl-1*H*-pyrazole-4-carbo-nitrile (18l)

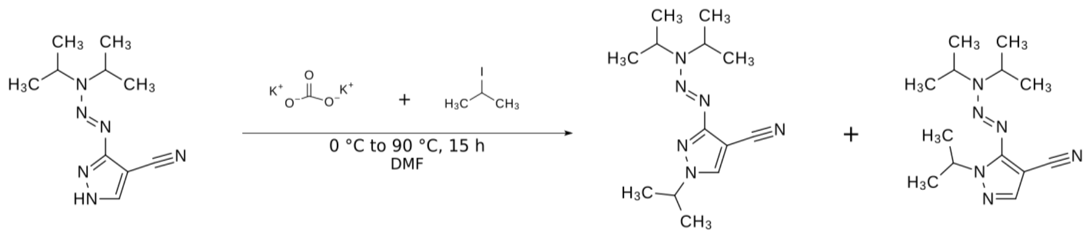

Name {P1|**17l**}: (*E*)-3-(3,3-diisopropyltriaz-1-en-1-yl)-1-isopropyl-1*H*-pyrazole-4-carbo-nitrile; Formula: $C_{13}H_{22}N_6$; Smiles: N#Cc1cn(nc1/N=N/N(C(C)C)C(C)C)C(C)C. InChIKey: XUZXRALOPOUFGP-BMRADRMJSA-N

Name {P2|**18l**}: (*E*)-5-(3,3-diisopropyltriaz-1-en-1-yl)-1-isopropyl-1*H*-pyrazole-4-carbo-nitrile; Formula: $C_{13}H_{22}N_6$; Smiles: N#Cc1cnn(c1/N=N/N(C(C)C)C(C)C)C(C)C. InChIKey: IJEPRRMQECALCS-WUKNDPDISA-N

To a solution of (*E*)-3-(3,3-diisopropyltriaz-1-en-1-yl)-1*H*-pyrazole-4-carbonitrile (504 mg, 2.29 mmol, 1.00 equiv) in 10 mL of anhydrous DMF, potassium carbonate

(376 mg, 2.72 mmol, 1.19 equiv) was added at 0 °C and the resulting mixture was stirred for 45 minutes. 2-Iodopropane (463 mg, 272 µL, 2.72 mmol, 1.19 equiv) was added slowly over 15 minutes and the reaction mixture was heated to 90 °C and stirred for 14 hours. Then the mixture was cooled, poured into ice cold water and the aqueous phase was extracted with ethyl acetate (3 x 20 mL). The combined organic layers were dried over Na_2SO_4 and filtered. As preparation for the column chromatography (dryload), Celite was added and the reaction mixture with Celite was evaporated. The obtained crude product was purified *via* flash-chromatography on silica gel using cyclohexane/ethyl acetate 20:1 to 4:1. The isolated product (*E*)-3-(3,3-diisopropyltriaz-1-en-1-yl)-1-isopropyl-1*H*-pyrazole-4-carbonitrile (211 mg, 805 µmol) was obtained as a colorless solid in 35% yield and the side product (*E*)-5-(3,3-diisopropyltriaz-1-en-1-yl)-1-isopropyl-1*H*-pyrazole-4-carbonitrile (347 mg, 1.32 mmol) was obtained as a colorless solid in 58% yield.

R_f = 0.31 (cyclohexane/ethyl acetate 4:1). MP = 103.8–104.7 °C. ^{1}H NMR (400 MHz, Chloroform-d [7.27 ppm], ppm) δ = 7.70 (s, 1H), 5.47 (sept, *J* = 6.7 Hz, 1H), 4.41 (sept, *J* = 6.7 Hz, 1H), 4.02 (sept, *J* = 6.5 Hz, 1H), 1.53 (d, *J* = 6.7 Hz, 6H), 1.45 (d, *J* = 6.6 Hz, 6H), 1.24 (d, *J* = 6.7 Hz, 6H); ^{13}C NMR (100 MHz, Chloroform-d [77.0 ppm], ppm) δ = 162.5 (C_q), 133.2 (CH), 115.5 (C_q), 80.1 (C_q), 54.8 (CH), 49.5 (CH), 46.1 (CH), 23.2 (CH_3, 2C), 22.4 (CH_3, 2C), 19.2 (CH_3, 2C); MS (FAB, 3-NBA): *m/z* (%) = 264 (16) $[M+2H]^+$, 263 (100) $[M+H]^+$, 262 (25) $[M]^+$, 162 (35), 154 (10), 136 (10), 100 (24). HRMS–FAB *(m/z)*: $[M+H]^+$ calcd for $C_{13}H_{23}N_6$, 263.1984; found, 263.1986; IR (ATR, ṽ) = 3118 (w), 3060 (vw), 2975 (m), 2929 (w), 2876 (w), 2225 (m), 2169 (vw), 1540 (m), 1463 (w), 1453 (w), 1439 (w), 1405 (vs), 1384 (vs), 1364 (vs), 1254 (vs), 1225 (vs), 1190 (m), 1181 (m), 1150 (vs), 1129 (s), 1101 (s), 1074 (w), 1028 (s), 997 (m), 946 (w), 929 (w), 909 (w), 880 (w), 839 (m), 764 (vw), 720 (w), 681 (w), 643 (w), 629 (m), 557 (w), 538 (w), 469 (w), 421 (vw) cm^{-1}.

R_f = 0.46 (cyclohexane/ethyl acetate 4:1). MP = 102.1–102.6 °C. ^{1}H NMR (400 MHz, Chloroform-d [7.27 ppm], ppm) δ = 7.64 (s, 1H), 5.19 (sept, *J* = 6.9 Hz, 1H), 4.89 (sept, *J* = 6.6 Hz, 1H), 4.10 (sept, *J* = 6.5 Hz, 1H), 1.48 (d, *J* = 3.8 Hz, 6H), 1.46 (d, *J* = 3.7 Hz, 6H), 1.31 (d, *J* = 6.7 Hz, 6H); ^{13}C NMR (100 MHz, Chloroform-d [77.0 ppm], ppm) δ = 153.7 (C_q), 141.7 (CH), 116.1 (C_q), 77.4 (C_q), 51.1 (CH), 49.7 (CH), 47.7 (CH), 23.1 (CH_3, 2C), 21.8 (CH_3, 2C), 18.9 (CH_3, 2C); MS (FAB, 3-NBA): *m/z* (%) = 264 (15) $[M+2H]^+$, 263 (100) $[M+H]^+$, 262 (43) $[M]^+$, 162 (10), 100 (11). HRMS–FAB *(m/z)*: $[M+H]^+$ Calcd for $C_{13}H_{23}N_6$, 263.1984; Found, 263.1984; IR (ATR, ṽ) = 3111 (vw), 2970 (w), 2934 (w), 2873 (vw), 2221 (m), 1680 (vw), 1534 (m), 1483 (w), 1469 (w), 1426 (vs), 1400 (vs), 1380 (vs), 1366 (vs), 1276 (s), 1259 (m), 1218 (s), 1193 (w), 1174 (m), 1153 (m), 1132 (m), 1098 (vs), 1060 (w), 1031 (w), 1024 (w), 1003 (vs), 932 (w), 908 (m), 884 (w), 841 (m), 734 (vw), 714 (w), 703 (w), 657 (w), 633 (w), 592 (w), 551 (w), 526 (m), 460 (w), 443 (w), 419 (vw), 405 (vw), 391 (vw) cm^{-1}.

Additional information on the chemical synthesis is available *via* Chemotion repository: https://doi.org/10.14272/reaction/SA-FUHFF-UHFFFADPSC-NRCRXOUYQJ-UHFFFADPSC-NUHFF-NDGQT-NUHFF-ZZZ

Additional information on the analysis of the target compound is available *via* Chemotion repository:
https://doi.org/10.14272/XUZXRALOPOUFGP-BMRADRMJSA-N.1
https://doi.org/10.14272/IJEPRRMQECALCS-WUKNDPDISA-N.1

Ethyl (*E*)-2-(4-cyano-3-(3,3-diisopropyltriaz-1-en-1-yl)-1*H*-pyrazol-1-yl)acetate (**17m**), **ethyl (*E*)-2-(4-cyano-5-(3,3-diisopropyltriaz-1-en-1-yl)-1*H*-pyrazol-1-yl)acetate** (**18m**)

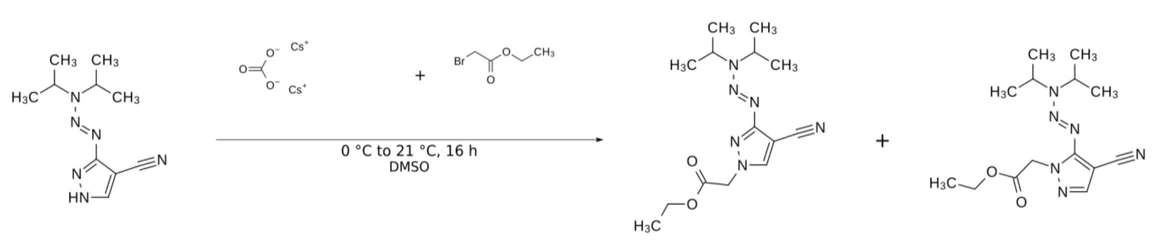

Name {P1|**17m**}: ethyl (*E*)-2-(4-cyano-3-(3,3-diisopropyltriaz-1-en-1-yl)-1*H*-pyrazol-1-yl)-acetate; Formula: $C_{14}H_{22}N_6O_2$; Smiles: CCOC(=O)Cn1nc(c(c1)C#N)/N=N/N(C(C)C)C(C)C. InChIKey: GMSYXQOQYOUAHU-FBMGVBCBSA-N

Name {P2|**18m**}: ethyl (*E*)-2-(4-cyano-5-(3,3-diisopropyltriaz-1-en-1-yl)-1*H*-pyrazol-1-yl)acetate; Formula: $C_{14}H_{22}N_6O_2$; Smiles: CCOC(=O)Cn1ncc(c1/N=N/N(C(C)C)C(C)C)C#N. InChIKey: UVXRQPKXJULFTR-ISLYRVAYSA-N

(*E*)-3-(3,3-Diisopropyltriaz-1-en-1-yl)-1*H*-pyrazole-4-carbonitrile (500 mg, 2.27 mmol, 1.00 equiv) was dissolved in DMSO (23.0 mL) and cesium carbonate (887 mg, 2.72 mmol, 1.20 equiv) was added. The reaction mixture was cooled to 0 °C and ethyl 2-bromoacetate (758 mg, 503 µL, 4.54 mmol, 2.00 equiv) was slowly added. After the addition, the reaction mixture was slowly warmed to 21 °C and stirred for 16 hours at this temperature. For workup, ice water was added to the reaction mixture. The aqueous phase was extracted three times with ethyl acetate and the combined organic phases were washed with water and brine, dried over Na_2SO_4, filtered and the solvent was removed under reduced pressure. As preparation for column chromatography (dry load), Celite was added (1.5 g) and the reaction mixture with Celite was evaporated. The obtained crude product was purified *via* flash-chromatography (Interchim devices puriFLASH 5.125) on silica gel (PF-15SIHP-F0040) using cyclohexane/ethyl acetate with 0% to 40% ethyl acetate in 12 column volumes. The isolated product ethyl (*E*)-2-(4-cyano-3-(3,3-diisopropyltriaz-1-en-1-yl)-1*H*-pyrazol-1-yl)acetate (486 mg, 1.59 mmol) was obtained as a colourless oil in 70% yield and the side product ethyl (*E*)-2-(4-cyano-5-(3,3-diisopropyltriaz-1-en-1-yl)-1*H*-pyrazol-1-yl)acetate (154 mg, 503 µmol) was obtained as a colourless solid in 22% yield.

R_f = 0.55 (cyclohexane/ethyl acetate 1:1). ^{1}H NMR (400 MHz, Chloroform-d [7.27 ppm], ppm) δ = 7.81 (s, 1H), 5.38 (sept, *J* = 6.8 Hz, 1H), 4.84 (s, 2H), 4.26 (q, *J* = 7.2 Hz, 2H), 4.04 (sept, *J* = 6.7 Hz, 1H), 1.45 (d, *J* = 6.6 Hz, 6H), 1.30 (t, *J* = 7.2 Hz, 3H), 1.25 (d, *J* = 6.7 Hz, 6H); ^{13}C NMR (100 MHz, Chloroform-d [77.0 ppm], ppm) δ = 166.7 (C_q), 162.8 (C_q), 137.3 (CH), 114.8 (C_q), 82.4 (C_q), 62.3 (CH_2), 53.5 (CH_2), 49.9 (CH), 46.6 (CH), 23.2 (CH_3, 2C), 19.1 (CH_3, 2C), 14.1 (CH_3); MS (EI, 70 eV, 80 °C), m/z (%): 306 (35) $[M]^+$, 206 (100), 199 (24), 169 (10), 155 (39), 131 (14), 100 (41), 91 (41), 69 (52), 59 (23). HRMS–EI ($C_{14}H_{22}O_2N_6$) *(m/z)*: $[M]^+$ Calcd 306.1799; Found 306.1798; IR (ATR, ṽ) = 3131 (vw), 3063 (vw), 2976 (w), 2936 (w), 2227 (m), 1748 (vs), 1544 (s), 1465 (m), 1409 (vs), 1370 (vs), 1356 (vs), 1299 (w), 1261 (vs), 1210 (vs), 1157 (vs), 1130 (s), 1099 (s), 1031 (vs), 1006 (s), 970 (w), 909 (m), 874 (m), 843 (m), 806 (m), 720 (s), 697 (m), 639 (m), 620 (w), 577 (w), 561 (m), 531 (m), 384 (w) cm^{-1}.

R_f = 0.60 (cyclohexane/ethyl acetate 1:1). MP = 79.5–84.5 °C. ^{1}H NMR (400 MHz, Chloroform-d [7.27 ppm], ppm) δ = 7.67 (s, 1H), 5.11 (sept, *J* = 6.8 Hz, 1H), 4.95 (s, 2H), 4.22 (q, *J* = 7.2 Hz, 2H), 4.10 (sept, *J* = 6.6 Hz, 1H), 1.45 (d, *J* = 6.6 Hz, 6H), 1.28–1.24 (m, 9H); ^{13}C NMR (100 MHz, Chloroform-d [77.0 ppm], ppm) δ = 167.3 (C_q), 155.2 (C_q), 142.4 (CH), 115.4 (C_q), 77.9 (C_q), 61.8 (CH_2), 51.5 (CH), 49.8 (CH_2), 48.1 (CH), 23.0 (CH_3, 2C), 18.7 (CH_3, 2C), 14.1 (CH_3); MS (EI, 70 eV, 70 °C), m/z (%): 306 (96) $[M]^+$, 233 (15), 206 (78), 161 (12), 150 (15), 122 (48), 106 (10), 100 (100), 94 (13), 84 (21), 78 (16), 70 (10), 58 (27). HRMS–EI ($C_{14}H_{22}O_2N_6$) *(m/z)*: $[M]^+$ Calcd 306.1799; Found 306.1800; IR (ATR, ṽ) = 2982 (w), 2939 (w), 2876 (w), 2220 (m), 1735 (vs), 1538 (m), 1502 (w), 1465 (w), 1417 (vs), 1391 (vs), 1375 (vs), 1361 (vs), 1341 (s), 1313 (w), 1293 (m), 1278 (vs), 1255 (vs), 1241 (vs), 1211 (vs), 1191 (s), 1179 (s), 1162 (vs), 1143 (s), 1135 (s), 1102 (vs), 1051 (s), 1028 (vs), 1020 (vs), 945 (m), 911 (m), 882 (w), 857 (s), 850 (m), 805 (w), 779 (m), 730 (w), 711 (s), 646 (m), 628 (w), 575 (m), 564 (m), 543 (m), 528 (m), 514 (w), 432 (w), 422 (w) cm^{-1}.

Additional information on the chemical synthesis is available *via* Chemotion repository: https://doi.org/10.14272/reaction/SA-FUHFF-UHFFFADPSC-HLSFJQKZZG-UHFFFADPSC-NUHFF-NQFOL-NUHFF-ZZZ.1

Additional information on the analysis of the target compound is available *via* Chemotion repository:
https://doi.org/10.14272/GMSYXQOQYOUAHU-FBMGVBCBSA-N.2
https://doi.org/10.14272/UVXRQPKXJULFTR-ISLYRVAYSA-N.2

The synthesis of this compound has been previously described by our group with yields of 54% for **17m** and 15% for **18m**: [5]
https://dx.doi.org/10.14272/reaction/SA-FUHFF-UHFFFADPSC-HLSFJQKZZG-UHFFFADPSC-NUHFF-NQFOL-NUHFF-ZZZ

(*E*)-1-Benzyl-3-(3,3-diisopropyltriaz-1-en-1-yl)-1*H*-pyrazole-4-carbonitrile (17n), (*E*)-1-benzyl-5-(3,3-diisopropyltriaz-1-en-1-yl)-1*H*-pyrazole-4-carbonitrile (18n)

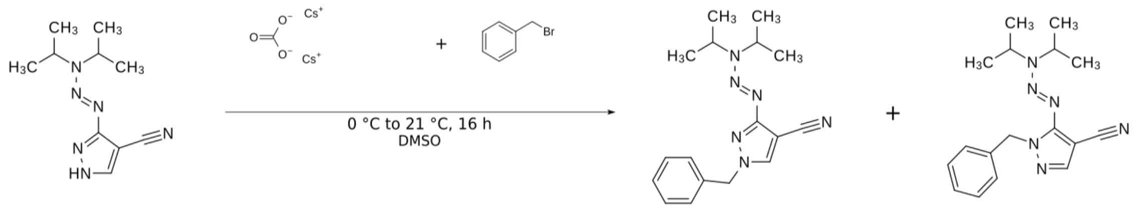

Name {P1|**17n**}: (*E*)-1-benzyl-3-(3,3-diisopropyltriaz-1-en-1-yl)-1*H*-pyrazole-4-carbonitrile; Formula: $C_{17}H_{22}N_6$; Smiles: N#Cc1cn(nc1/N=N/N(C(C)C)C(C)C)Cc1ccccc1. InChIKey: AONLLYWWGOIMLR-XUTLUUPISA-N

Name {P2|**18n**}: (*E*)-1-benzyl-5-(3,3-diisopropyltriaz-1-en-1-yl)-1*H*-pyrazole-4-carbonitrile; Formula: $C_{17}H_{22}N_6$; Smiles: CC(N(C(C)C)/N=N/c1n(ncc1C#N)Cc1ccccc1)C. InChIKey: AQYSAXXLCHFEGV-QZQOTICOSA-N

(*E*)-3-(3,3-Diisopropyltriaz-1-en-1-yl)-1*H*-pyrazole-4-carbonitrile (500 mg, 2.27 mmol, 1.00 equiv) was dissolved in DMSO (23.0 mL) and cesium carbonate (887 mg, 2.72 mmol, 1.20 equiv) was added. The reaction mixture was cooled to 0 °C and bromomethylbenzene (776 mg, 540 µL, 4.54 mmol, 2.00 equiv) was slowly added. After the addition, the reaction mixture was slowly warmed to 21 °C and stirred for 16 hours at this temperature. For workup, ice water was added to the reaction mixture. The aqueous phase was extracted three times with ethyl acetate and the combined organic phases were washed with water and brine, dried over Na_2SO and filtered. As preparation for column chromatography (dry load), Celite was added (1.5 g) and the reaction mixture with Celite was evaporated. The obtained crude product was purified *via* flash-chromatography (Interchim devices puriFLASH 5.125) on silica gel (PF-15SIHP-F0080) using cyclohexane/ethyl acetate with 0% to 20% ethyl acetate in 17 column volumes. The isolated product (*E*)-1-benzyl-3-(3,3-diisopropyltriaz-1-en-1-yl)-1*H*-pyrazole-4-carbonitrile (411 mg, 1.32 mmol) was obtained as a colorless solid in 58% yield and the side product (*E*)-1-benzyl-5-(3,3-diisopropyltriaz-1-en-1-yl)-1*H*-pyrazole-4-carbonitrile (276 mg, 889 µmol) was obtained as a colourless solid in 39% yield.

R_f = 0.24 (cyclohexane/ethyl acetate 4:1). MP = 138.1–138.9 °C. ^{1}H NMR (400 MHz, Chloroform-d [7.27 ppm], ppm) δ = 7.62 (s, 1H), 7.41–7.29 (m, 5H), 5.42 (sept, *J* = 6.8 Hz, 1H), 5.24 (s, 2H), 4.03 (sept, *J* = 6.6 Hz, 1H), 1.45 (d, *J* = 6.6 Hz, 6H), 1.25 (d, *J* = 6.8 Hz, 6H); ^{13}C NMR (100 MHz, Chloroform-d [77.0 ppm], ppm) δ = 162.8 (C_q), 135.5 (CH), 134.7 (C_q), 129.0 (CH, 2C), 128.7 (CH), 128.2 (CH, 2C), 115.1 (C_q), 81.4 (C_q), 56.7 (CH_2), 49.8 (CH), 46.4 (CH), 23.2 (CH_3, 2C), 19.2 (CH_3, 2C); MS (EI, 70 eV, 120 °C), m/z (%): 310 (52) $[M]^+$, 267 (16), 210 (100), 155 (16), 100 (45), 91 (86), 58 (16). HRMS–EI ($C_{17}H_{22}N_6$) *(m/z)*: $[M]^+$ Calcd 310.1900; Found 310.1899; IR (ATR, ṽ) = 3128 (vw), 3112 (vw), 3061 (vw), 3033 (vw), 2975 (w), 2963 (w), 2932 (w), 2874 (vw), 2218 (m), 1538 (s), 1496 (vw), 1456 (w), 1448 (w), 1414 (vs), 1367 (s), 1353 (m), 1259 (vs), 1227 (s), 1207 (w), 1187 (w), 1154 (vs), 1132 (w), 1101 (m), 1081 (w), 1031 (m), 1007 (w), 948 (vw), 911 (w), 885 (vw), 840 (w), 820 (w), 805 (w), 752 (m), 720 (w), 707 (vs), 677 (w), 645 (w), 632 (w), 591 (w), 555 (w), 534 (m), 467 (w), 418 (vw), 375 (w) cm^{-1}.

R_f = 0.33 (cyclohexane/ethyl acetate 4:1). MP = 123.8–124.9 °C. ^{1}H NMR (400 MHz, Chloroform-d [7.27 ppm], ppm) δ = 7.66 (s, 1H), 7.34–7.25 (m, 5H), 5.37 (s, 2H), 5.17 (sept, *J* = 6.6 Hz, 1H), 4.09 (sept, *J* = 6.5 Hz, 1H), 1.45 (d, *J* = 6.6 Hz, 6H), 1.27 (d, *J* = 6.6 Hz, 6H); ^{13}C NMR (100 MHz, Chloroform-d [77.0 ppm], ppm) δ = 154.6 (C_q), 142.3 (CH), 136.3 (C_q), 128.6 (CH, 2C), 127.8 (CH), 127.7 (CH, 2C), 115.8 (C_q), 77.7 (C_q), 52.1 (CH_2), 51.3 (CH), 48.0 (CH), 23.1 (CH_3, 2C), 18.8 (CH_3, 2C); MS (EI, 70 eV, 90 °C), m/z (%): 310 (100) $[M]^+$, 210 (43), 100 (44), 91 (68), 58 (14). HRMS–EI ($C_{17}H_{22}N_6$) *(m/z)*: $[M]^+$ Calcd 310.1900; Found 310.1902; IR (ATR, ṽ) = 3112 (vw), 3033 (vw), 2987 (w), 2975 (w), 2941 (w), 2873 (vw), 2220 (s), 1533 (m), 1494 (w), 1466 (w), 1455 (m), 1441 (w), 1419 (vs), 1391 (m), 1375 (vs), 1363 (vs), 1313 (w), 1272 (s), 1238 (vs), 1211 (s), 1164 (m), 1132 (m), 1105 (vs), 1078 (w), 1038 (w), 1026 (vs), 983 (w), 963 (w), 945 (w), 935 (w), 909 (w), 884 (s), 851 (w), 813 (w), 785 (m), 724 (vs), 710 (s), 691 (vs), 666 (w), 630 (w), 615 (w), 595 (w), 567 (w), 550 (m), 533 (s), 516 (w), 458 (w), 450 (w), 421 (w), 385 (w) cm^{-1}.

Additional information on the chemical synthesis is available *via* Chemotion repository: https://doi.org/10.14272/reaction/SA-FUHFF-UHFFFADPSC-KRKANRSFKB-UHFFFADPSC-NUHFF-NVGOA-NUHFF-ZZZ.1

Additional information on the analysis of the target compound is available *via* Chemotion repository:
https://doi.org/10.14272/AONLLYWWGOIMLR-XUTLUUPISA-N.2
https://doi.org/10.14272/AQYSAXXLCHFEGV-QZQOTICOSA-N.2

The synthesis of this compound has been previously described by our group with yields of 54% for **17n** and 36% for **18n**: [5]
https://doi.org/10.14272/reaction/SA-FUHFF-UHFFFADPSC-KRKANRSFKB-UHFFFADPSC-NUHFF-NVGOA-NUHFF-ZZZ

(*E*)-1-(3,5-Difluorobenzyl)-3-(3,3-diisopropyltriaz-1-en-1-yl)-1*H*-pyrazole-4-carbonitrile (**17o**), **(*E*)-1-(3,5-difluorobenzyl)-5-(3,3-diisopropyltriaz-1-en-1-yl)-1*H*-pyrazole-4-carbonitrile** (**18o**)

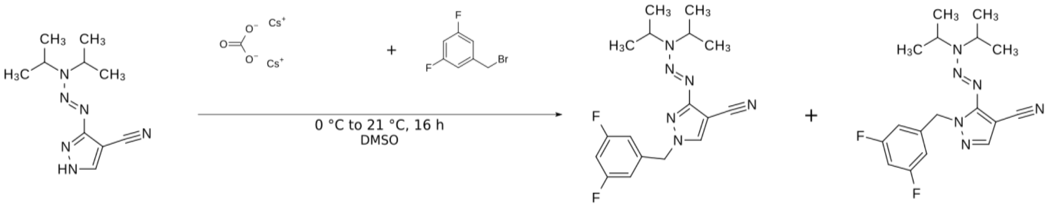

Name {P1|**17o**}: (*E*)-1-(3,5-difluorobenzyl)-3-(3,3-diisopropyltriaz-1-en-1-yl)-1*H*-pyrazole-4-carbonitrile; Formula: $C_{17}H_{20}F_2N_6$; Smiles: N#Cc1cn(nc1/N=N/N(C(C)C)C(C)C)Cc1cc(F)cc(c1)F. InChIKey: WSQPCVJPBSUDNZ-XTQSDGFTSA-N

Name {P2|**18o**}: (*E*)-1-(3,5-difluorobenzyl)-5-(3,3-diisopropyltriaz-1-en-1-yl)-1*H*-pyrazole-4-carbonitrile; Formula: $C_{17}H_{20}F_2N_6$; Smiles: CC(N(C(C)C)/N=N/c1n(ncc1C#N)Cc1cc(F)cc(c1)F)C. InChIKey: ZFEWCQZPKGQXCJ-GHVJWSGMSA-N

(*E*)-3-(3,3-Diisopropyltriaz-1-en-1-yl)-1*H*-pyrazole-4-carbonitrile (1.00 g, 4.54 mmol, 1.00 equiv) was dissolved in DMSO (46.0 mL) and cesium carbonate (1.77 g, 5.45 mmol, 1.20 equiv) was added. The reaction mixture was cooled to 0 °C and 1-(bromomethyl)-3,5-difluorobenzene (1.88 g, 1.17 mL, 9.08 mmol, 2.00 equiv) was slowly added. After the addition, the reaction mixture was slowly warmed to 21 °C and stirred for 16 hours at this temperature. For workup, ice water was added to the reaction mixture. The aqueous phase was extracted three times with ethyl acetate and the combined organic phase was washed with water and brine, dried over Na_2SO_4 and filtered. As preparation for column chromatography (dryload), Celite was added (3.0 g) and the reaction mixture with Celite were evaporated. The obtained crude product was purified *via* flash-chromatography (Interchim devices puriFLASH 5.125) on silica gel (PF-15SIHP-F0080) using cyclohexane/ethyl acetate with 0% to 30% ethyl acetate in 15 column volumes (1 column volume = 173.2 mL; flow: 34 mL/min). The isolated product (*E*)-1-(3,5-difluorobenzyl)-3-(3,3-diisopropyltriaz-1-en-1-yl)-1*H*-pyrazole-4-carbonitrile (795 mg, 2.30 mmol) was obtained as a colourless solid in 51% yield and the side product (*E*)-1-(3,5-difluorobenzyl)-5-(3,3-diisopropyltriaz-1-en-1-yl)-1*H*-

pyrazole-4-carbonitrile (719 mg, 2.08 mmol) was obtained as a colourless solid in 46% yield.

R_f = 0.43 (cyclohexane/ethyl acetate 2:1). MP = 144.9–145.7 °C. ^{1}H NMR (400 MHz, Chloroform-d [7.27 ppm], ppm) δ = 7.74 (s, 1H), 6.80–6.75 (m, 3H), 5.41 (sept, J = 6.8 Hz, 1H), 5.20 (s, 2H), 4.04 (sept, J = 6.7 Hz, 1H), 1.46 (d, J = 6.6 Hz, 6H), 1.25 (d, J = 6.8 Hz, 6H); ^{13}C NMR (100 MHz, Chloroform-d [77.0 ppm], ppm) δ = 163.3 (dd, J = 250.3, 12.6 Hz, C_q, 2C), 163.2 (C_q), 138.7 (t, J = 8.9 Hz, C_q), 135.9 (CH), 114.8 (CH), 110.7 (dd, J = 7.7 Hz, J = 19.3 Hz, 2C, CH), 104.0 (t, J = 25.0 Hz, CH), 82.0 (C_q), 55.6 (t, J = 2.3 Hz, CH_2), 49.8, 46.5, 23.2 (2C, CH_3), 19.1 (2C, CH_3); ^{19}F NMR (376 MHz, ppm) δ = -108.21–-108.29 (m); MS (EI, 70 eV, 120 °C), m/z (%): 346 (42) $[M]^+$, 303 (11), 246 (89), 191 (12), 127 (100), 100 (70), 58 (49). HRMS–EI ($C_{17}H_{20}N_6F_2$) *(m/z)*: $[M]^+$ Calcd 346.1712; Found 346.1712; IR (ATR, ṽ) = 3125 (vw), 3055 (vw), 2989 (w), 2976 (w), 2962 (w), 2934 (w), 2873 (vw), 2221 (s), 1625 (m), 1598 (m), 1540 (s), 1462 (m), 1439 (m), 1412 (vs), 1368 (s), 1356 (s), 1329 (w), 1317 (s), 1262 (vs), 1230 (s), 1188 (w), 1156 (vs), 1143 (m), 1119 (vs), 1099 (s), 1031 (s), 1014 (s), 997 (s), 976 (w), 948 (w), 909 (w), 892 (w), 873 (m), 849 (vs), 839 (vs), 796 (m), 734 (m), 718 (s), 690 (m), 654 (s), 633 (m), 612 (w), 582 (m), 561 (w), 535 (s), 521 (w), 510 (m), 476 (w), 466 (vw), 458 (w), 419 (vw), 375 (w) cm^{-1}.

R_f = 0.53 (cyclohexane/ethyl acetate 2:1). MP = 145.7–146.9 °C. ^{1}H NMR (400 MHz, Chloroform-d [7.27 ppm], ppm) δ = 7.68 (s, 1H), 6.79–6.70 (m, 3H), 5.33 (s, 2H), 5.15 (sept, J = 6.8 Hz, 1H), 4.12 (sept, J = 6.5 Hz, 1H), 1.47 (d, J = 6.6 Hz, 6H), 1.28 (d, J = 6.8 Hz, 6H). ^{13}C NMR (100 MHz, Chloroform-d [77.0 ppm], ppm) δ = 163.1 (dd, J = 249.4, 12.7 Hz, C_q, 2C), 154.8 (C_q), 142.6 (CH), 140.1 (t, J = 8.9 Hz, C_q), 115.5 (C_q), 110.7 (dd, J = 6.9 Hz, J = 18.5 Hz, CH, 2C), 103.3 (t, J = 25.0 Hz, CH), 78.0 (C_q), 51.4 (CH), 51.3 (t, J = 2.3 Hz, CH_2), 48.1 (CH), 23.1 (2C, CH_3), 18.8 (2C, CH_3). ^{19}F NMR (376 MHz, ppm) δ = -109.08–-109.16 (m); MS (FAB, 3-NBA), m/z (%): 347 (100) $[M+1]^+$, 154 (20), 136 (15), 127 (13), 100 (10). HRMS–FAB ($C_{17}H_{21}N_6F_2$) *(m/z)*: $[M+H]^+$ Calcd 347.1790; Found 347.1792; IR (ATR, ṽ) = 3128 (vw), 3072 (vw), 3058 (vw), 2985 (w), 2946 (w), 2880 (vw), 2218 (s), 1742 (vw), 1625 (m), 1599 (m), 1536 (m), 1493 (w), 1466 (m), 1446 (m), 1424 (vs), 1392 (w), 1366 (vs), 1349 (vs), 1315 (s), 1293 (w), 1271 (m), 1242 (s), 1221 (vs), 1205 (s), 1173 (m), 1164 (m), 1122 (vs), 1102 (vs), 1043 (w), 1028 (vs), 990 (m), 935 (s), 911 (m), 884 (w), 873 (m), 858 (vs), 847 (vs), 773 (s), 735 (w), 713 (s), 697 (m), 663 (m), 632 (s), 606 (m), 588 (m), 561 (m), 544 (m), 530 (s), 517 (m), 509 (m), 480 (w), 469 (w), 463 (w), 449 (w), 421 (w), 399 (w), 380 (w) cm^{-1}.

Additional information on the chemical synthesis is available *via* Chemotion repository: https://doi.org/10.14272/reaction/SA-FUHFF-UHFFFADPSC-RPEOLKMCSJ-UHFFFADPSC-NUHFF-NJTWA-NUHFF-ZZZ.1

Additional information on the analysis of the target compound is available *via* Chemotion repository:
https://doi.org/10.14272/WSQPCVJPBSUDNZ-XTQSDGFTSA-N.2
https://doi.org/10.14272/ZFEWCQZPKGQXCJ-GHVJWSGMSA-N.2

The synthesis of this compound has been previously described by our group with yields of 48% for **17o** and 42% for **18o**: [5]
https://doi.org/10.14272/reaction/SA-FUHFF-UHFFFADPSC-RPEOLKMCSJ-UHFFFADPSC-NUHFF-NJTWA-NUHFF-ZZZ

(*E*)-3-(3,3-Diisopropyltriaz-1-en-1-yl)-1-(4-methylbenzyl)-1*H*-pyrazole-4-carbonitrile (17p), (*E*)-5-(3,3-diisopropyltriaz-1-en-1-yl)-1-(4-methylbenzyl)-1*H*-pyrazole-4-carbonitrile (18p)

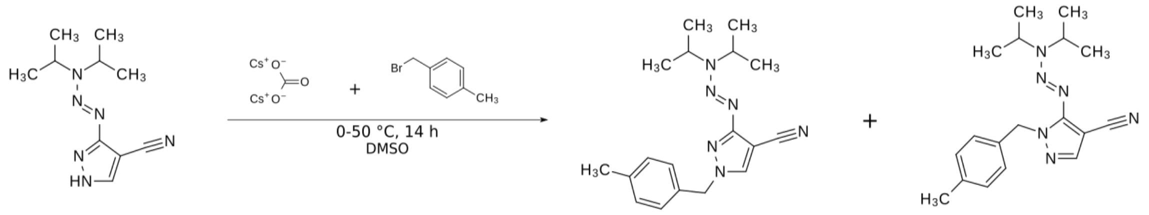

Name {P1|**17p**}: (*E*)-3-(3,3-diisopropyltriaz-1-en-1-yl)-1-(4-methylbenzyl)-1H-pyrazole-4-carbonitrile; Formula: $C_{18}H_{24}N_6$; Smiles: N#Cc1cn(nc1/N=N/N(C(C)C)C(C)C)Cc1ccc(cc1)C. InChIKey: GJXVYPGROXVEAZ-LSDHQDQOSA-N

Name {P2|**18p**}: (*E*)-5-(3,3-diisopropyltriaz-1-en-1-yl)-1-(4-methylbenzyl)-1H-pyrazole-4-carbonitrile; Formula: $C_{18}H_{24}N_6$; Smiles: N#Cc1cnn(c1/N=N/N(C(C)C)C(C)C)Cc1ccc(cc1)C. InChIKey: RAZAPXAZBSDVNH-QURGRASLSA-N

In a vial, (*E*)-3-(3,3-diisopropyltriaz-1-en-1-yl)-1*H*-pyrazole-4-carbonitrile (563 mg, 2.55 mmol, 1.00 equiv) was dissolved in 20 mL of DMSO. The solution was cooled to 0 °C. Cesium carbonate (1.00 g, 3.07 mmol, 1.20 equiv) and 1-(bromomethyl)-4-methylbenzene (700 mg, 3.78 mmol, 1.48 equiv) were added. The mixture was stirred first at 21 º for 2 hours, then at 50 °C for 12 hours. The reaction was quenched with ice and extracted with ethyl acetate (3x 20 mL). The solvent was removed under reduced pressure to give the crude product. The obtained crude product was purified *via* flash-chromatography on silica gel using cyclohexane/ethyl acetate 10:1 to 4:1, giving 3-(3,3-diisopropyltriaz-1-en-1-yl)-1-(4-methylbenzyl)-1*H*-pyrazole-4-carbonitrile (485 mg, 1.50 mmol, 59% yield) as a white solid and 5-(3,3-diisopropyltriaz-1-en-1-yl)-1-(4-methylbenzyl)-1*H*-pyrazole-4-carbonitrile (329 mg, 1.01 mmol, 40% yield) as a white solid.

R_f = 0.34 (cyclohexane/ethyl acetate 4:1). ^{1}H NMR (400 MHz, Chloroform-d, ppm) δ = 7.58 (s, 1H), 7.21–7.10 (m, 4H), 5.39 (sept, *J* = 7.1 Hz, 1H), 5.16 (s, 2H), 4.01 (sept, *J* = 6.6 Hz, 1H), 2.33 (s, 3H), 1.43 (d, *J* = 6.7 Hz, 6H), 1.23 (d, *J* = 6.8 Hz, 6H); ^{13}C NMR (100 MHz, Chloroform-d, ppm) δ = 162.8, 138.6, 135.5 (CH), 131.7, 129.8 (CH, 2C), 128.4 (CH, 2C), 115.3, 81.3, 56.6 (CH_2), 49.8 (CH), 46.4 (CH), 23.3 (CH_3, 2C), 21.2 (CH_3), 19.3 (CH_3, 2C); MS (FAB, 3-NBA): *m/z* (%) = 326 (22) $[M+2H]^+$, 325 (100) $[M+H]^+$, 324 (20) $[M]^+$, 224 (20), 154 (10), 105 (45), 100 (11), 95 (10). HRMS–FAB *(m/z)*: $[M+H]^+$ calcd for $C_{18}H_{25}N_6$, 325.2141; found, 325.2139; IR (ATR, ṽ) = 3132 (vw), 2978 (w), 2931 (w), 2873 (vw), 2218 (vs), 1615 (vw), 1540 (s), 1513 (w), 1468 (w), 1456 (m), 1439 (w), 1414 (vs), 1405 (vs), 1384 (s), 1370 (vs), 1353 (s), 1310 (w), 1264 (vs), 1232 (s), 1203 (w), 1157 (vs), 1132 (m), 1115 (w), 1099 (m), 1031 (m), 1021 (w), 1001 (w), 932 (vw), 912 (w), 843 (w), 820 (s), 782 (s), 752 (s), 717 (vs), 693 (m), 640 (w), 630 (m), 585 (w), 560 (w), 537 (m), 507 (w), 475 (m), 385 (vw) cm^{-1}.

R_f = 0.43 (cyclohexane/ethyl acetate 4:1). ^{1}H NMR (400 MHz, Chloroform-d [7.27 ppm], ppm) δ = 7.64 (s, 1H), 7.22–7.07 (m, 4H), 5.32 (s, 2H), 5.19 (sept, *J* = 6.8 Hz, 1H), 4.10 (sept, *J* = 6.6 Hz, 1H), 2.31 (s, 3H), 1.45 (d, *J* = 6.6 Hz, 6H), 1.28 (d, *J* = 6.8 Hz, 6H). ^{13}C NMR (100 MHz, Chloroform-d [77.16 ppm], ppm) δ = 154.7, 142.4 (CH), 137.8, 133.5, 129.4 (CH, 2C), 128.0 (CH, 2C), 116.0, 77.8, 52.0 (CH_2), 51.4 (CH), 48.1 (CH), 23.2 (CH_3, 2C), 21.2 (CH_3), 19.0 (CH_3, 2C); MS (FAB, 3-NBA): *m/z* (%) = 326 (22) $[M+2H]^+$, 325 (100) $[M+H]^+$, 324 (29) $[M]^+$, 105 (49), 100 (11). HRMS–FAB *(m/z)*: $[M+H]^+$ calcd for $C_{18}H_{25}N_6$, 325.2141; found, 325.2140; IR (ATR, ṽ) = 3121 (vw), 2972 (w), 2945 (w), 2873 (w), 2221 (vs), 1741 (vw), 1615 (vw), 1537 (m), 1514 (w), 1489 (w), 1466 (w), 1422 (vs), 1394 (vs), 1378 (vs), 1366 (vs), 1322 (w), 1279 (m), 1266 (s), 1238 (vs), 1208 (s), 1173 (w), 1162 (m), 1133 (m), 1102 (vs), 1026 (vs), 990 (w), 945 (w), 925 (w), 909 (w), 881 (w), 873 (m), 851 (w), 839 (w), 817 (m), 810 (w), 762 (s), 754 (vs), 728 (w), 707 (m), 659 (w), 642 (vw), 618 (m), 592 (w), 562 (w), 548 (w), 534 (m), 513 (m), 472 (s), 453 (w), 421 (w), 390 (vw) cm^{-1}.

Additional information on the chemical synthesis is available *via* Chemotion repository: https://doi.org/10.14272/reaction/SA-FUHFF-UHFFFADPSC-UZTCZZRTCD-UHFFFADPSC-NUHFF-NPQQK-NUHFF-ZZZ

Additional information on the analysis of the target compound is available *via* Chemotion repository:
https://doi.org/10.14272/GJXVYPGROXVEAZ-LSDHQDQOSA-N.1
https://doi.org/10.14272/RAZAPXAZBSDVNH-QURGRASLSA-N.1

The synthesis of **17p** and **18p** has been previously described by our group [5] and is given here for the reader's convenience.

Methyl (*E*)-4-((4-cyano-3-(3,3-diisopropyltriaz-1-en-1-yl)-1*H*-pyrazol-1-yl)methyl)benzoate (17q), methyl (*E*)-4-((4-cyano-5-(3,3-diisopropyltriaz-1-en-1-yl)-1*H*-pyrazol-1-yl)methyl)benzoate (18q)

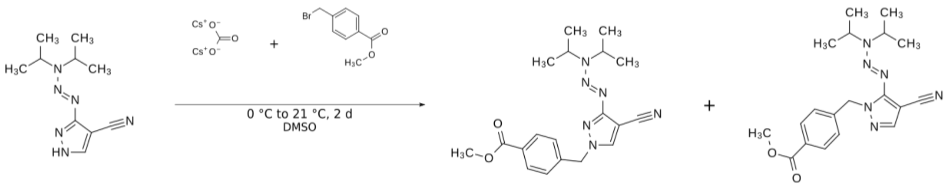

Name {P1|**17q**}: methyl (*E*)-4-((4-cyano-3-(3,3-diisopropyltriaz-1-en-1-yl)-1*H*-pyrazol-1-yl)methyl)benzoate; Formula: $C_{19}H_{24}N_6O_2$; Smiles: COC(=O)c1ccc(cc1)Cn1nc(c(c1)C#N)/N=N/N(C(C)C)C(C)C. InChIKey: FZUUXBMFWLBSAE-XTQSDGFTSA-N

Name {P2|**18q**}: methyl (*E*)-4-((4-cyano-5-(3,3-diisopropyltriaz-1-en-1-yl)-1*H*-pyrazol-1-yl)methyl)benzoate; Formula: $C_{19}H_{24}N_6O_2$; Smiles: COC(=O)c1ccc(cc1)Cn1ncc(c1/N=N/N(C(C)C)C(C)C)C#N. InChIKey: HQEHAWORIRDNAT-GHVJWSGMSA-N

(*E*)-3-(3,3-Diisopropyltriaz-1-en-1-yl)-1*H*-pyrazole-4-carbonitrile (77.8 mg, 353 µmol, 1.00 equiv) was dissolved in DMSO (3.0 mL) and cesium carbonate (138 mg, 424 µmol, 1.20 equiv) was added. The reaction mixture was cooled to 0 °C and methyl

4-(bromomethyl)benzoate (162 mg, 706 µmol, 2.00 equiv) was slowly added. After the addition, the reaction mixture was slowly warmed to 21 °C and stirred for 2 days at this temperature. For work up, ice-water was added to the reaction mixture. The aqueous phase was extracted three times with ethyl acetate and the combined organic phase was washed with water and brine, dried over Na_2SO_4 and filtered. As preparation for the column chromatography (dryload), Celite was added (0.3 g) and the reaction mixture with Celite was evaporated. The obtained crude product was purified *via* flash-chromatography on silica gel using cyclohexane/ethyl acetate 10:1 to 4:1. The isolated product methyl (*E*)-4-((4-cyano-3-(3,3-diisopropyltriaz-1-en-1-yl)-1*H*-pyrazol-1-yl)methyl)benzoate (68.7 mg, 186 µmol) was obtained as a colorless solid in 53% yield and the side product methyl (*E*)-4-((4-cyano-5-(3,3-diisopropyltriaz-1-en-1-yl)-1*H*-pyrazol-1-yl)methyl)benzoate (58.9 mg, 160 µmol) was obtained as a colorless solid in 45% yield.

R_f = 0.11 (cyclohexane/ethyl acetate 4:1). ^{1}H NMR (400 MHz, Chloroform-d [7.26 ppm], ppm) δ = 8.01–7.98 (m, 2H), 7.70 (s, 1H), 7.33–7.30 (m, 2H), 5.38 (sept, *J* = 6.8 Hz, 1H), 5.27 (s, 2H), 4.01 (sept, *J* = 6.7 Hz, 1H), 3.89 (s, 3H), 1.42 (d, *J* = 6.6 Hz, 6H), 1.22 (d, *J* = 6.7 Hz, 6H); ^{13}C NMR (100 MHz, Chloroform-d [77.16 ppm], ppm) δ = 166.5, 163.0, 139.9, 136.0 (CH), 130.4, 130.3 (CH, 2C), 127.9 (CH, 2C), 115.0, 81.8, 56.3 (CH_2), 52.3 (CH_3), 49.9 (CH), 46.6 (CH), 23.3 (CH_3, 2C), 19.2 (CH_3, 2C); MS (FAB, 3-NBA), m/z (%): 369 (100) $[M+H]^+$, 268 (22), 154 (20), 149 (25), 137 (10), 136 (14), 100 (13). HRMS–FAB ($C_{19}H_{24}N_6O_2$) *(m/z)*: $[M+H]^+$ Calcd 369.2039; Found 369.2038; IR (ATR, ṽ) = 3126, 2978, 2952, 2934, 2874, 2217, 1718, 1612, 1579, 1537, 1458, 1446, 1412, 1381, 1368, 1349, 1323, 1315, 1276, 1259, 1231, 1187, 1157, 1133, 1099, 1033, 1021, 1004, 965, 911, 871, 836, 796, 764, 715, 652, 637, 613, 582, 562, 533, 517, 503, 467, 419, 419, 409, 409, 380 cm^{-1}.

R_f = 0.13 (cyclohexane/ethyl acetate 4:1). ^{1}H NMR (400 MHz, Chloroform-d [7.26 ppm], ppm) δ = 8.00–7.97 (m, 2H), 7.67 (s, 1H), 7.31–7.28 (m, 2H), 5.42 (s, 2H), 5.12 (sept, *J* = 7.0 Hz, 1H), 4.09 (sept, *J* = 6.6 Hz, 1H), 3.90 (s, 3H), 1.45 (d, *J* = 6.6 Hz, 6H), 1.24 (ps, 6H); ^{13}C NMR (100 MHz, Chloroform-d, ppm) δ = 166.8, 154.9, 142.6 (CH), 141.5, 130.1 (CH, 2C), 129.9, 127.7 (CH, 2C), 115.8, 78.1, 52.3 (CH_3), 52.0 (CH_2), 51.6 (CH), 48.3 (CH), 23.2 (CH_3, 2C), 19.0 (CH_3, 2C); MS (FAB, 3-NBA), m/z (%): 369 (100) $[M+H]^+$,268 (11), 154 (5), 149 (14). HRMS–FAB ($C_{19}H_{25}N_6O_2$) *(m/z)*: $[M+H]^+$ Calcd 369.2039; Found 369.2039; IR (ATR, ṽ) = 2976, 2951, 2873, 2220, 1812, 1720, 1613, 1578, 1536, 1490, 1470, 1417, 1392, 1380, 1361, 1312, 1272, 1238, 1211, 1177, 1162, 1130, 1102, 1067, 1021, 965, 929, 908, 882, 850, 839, 800, 788, 772, 748, 728, 711, 703, 656, 637, 629, 613, 588, 564, 548, 533, 489, 421, 402 cm^{-1}.

Additional information on the chemical synthesis is available *via* Chemotion repository: https://doi.org/10.14272/reaction/SA-FUHFF-UHFFFADPSC-ZACAHENACG-UHFFFADPSC-NUHFF-NJTWA-NUHFF-ZZZ

Additional information on the analysis of the target compound is available *via* Chemotion repository:
https://doi.org/10.14272/FZUUXBMFWLBSAE-XTQSDGFTSA-N.1
https://doi.org/10.14272/HQEHAWORIRDNAT-GHVJWSGMSA-N.1

(*E*)-3-(3,3-Diisopropyltriaz-1-en-1-yl)-1-(4-fluorobenzyl)-1*H*-pyrazole-4-carbonitrile (**17r**), **(*E*)-5-(3,3-diisopropyltriaz-1-en-1-yl)-1-(4-fluorobenzyl)-1*H*-pyrazole-4-carbonitrile** (**18r**)

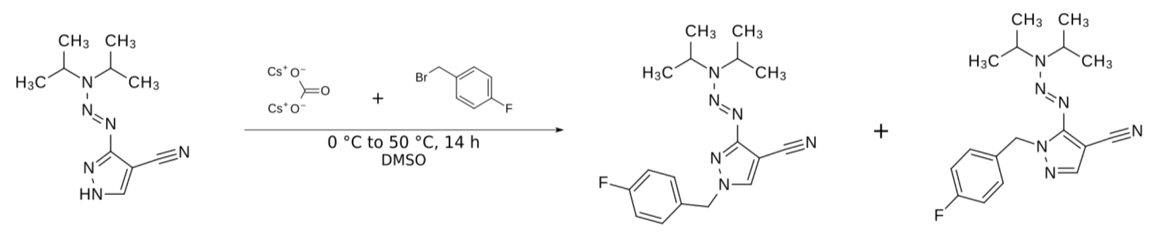

Name {P1|**17r**}: (*E*)-3-(3,3-diisopropyltriaz-1-en-1-yl)-1-(4-fluorobenzyl)-1*H*-pyrazole-4-carbonitrile; Formula: $C_{17}H_{21}FN_6$; Smiles: N#Cc1cn(nc1/N=N/N(C(C)C)C(C)C)Cc1ccc(cc1)F. InChIKey: ADGUCVKMBUJWJM-LSDHQDQOSA-N

Name {P2|**18r**}: (*E*)-5-(3,3-diisopropyltriaz-1-en-1-yl)-1-(4-fluorobenzyl)-1*H*-pyrazole-4-carbonitrile; Formula: $C_{17}H_{21}FN_6$; Smiles: N#Cc1cnn(c1/N=N/N(C(C)C)C(C)C)Cc1ccc(cc1)F. InChIKey: HEZWXNUXEKJBQT-QURGRASLSA-N

In a vial, (*E*)-3-(3,3-diisopropyltriaz-1-en-1-yl)-1*H*-pyrazole-4-carbonitrile (490 mg, 2.22 mmol, 1.00 equiv) was dissolved in DMSO (16.7 mL). The solution was cooled to 0 °C. Cesium Carbonate (865 mg, 2.66 mmol, 1.19 equiv) and 1-(bromomethyl)-4-fluorobenzene (850 mg, 560 µL, 4.49 mmol, 2.02 equiv) were added. The mixture was stirred first at 21 °C for 2 hours, then at 50 °C for 12 hours. The reaction was quenched with ice water and extracted with ethyl aceate (3 x 25 mL). The combined organic layers were washed with brine and dried over Na_2SO_4 and filtered. As preparation for the column chromatography (dryload), Celite was added and the reaction mixture with Celite was evaporated. The obtained crude product was purified *via* flash-chromatography on silica gel using cyclohexane/ethyl acetate 10:1 to 4:1. The isolated product (*E*)-3-(3,3-diisopropyltriaz-1-en-1-yl)-1-(4-fluorobenzyl)-1*H*-pyrazole-4-carbonitrile (396 mg, 1.20 mmol) was obtained as a colorless solid in 54% yield and the side product (*E*)-5-(3,3-diisopropyltriaz-1-en-1-yl)-1-(4-fluorobenzyl)-1*H*-pyrazole-4-carbonitrile (298 mg, 907 µmol) was obtained as a colorless solid in 41% yield.

R_f = 0.27 (cyclohexane/ethyl acetate 4:1). MP = 145.4–150 °C. ^{1}H NMR (400 MHz, Chloroform-d [7.26 ppm], ppm) δ = 7.64 (s, 1H), 7.31–7.27 (m, 2H), 7.07–7.03 (m, 2H), 5.41 (sept, *J* = 6.7 Hz, 1H), 5.19 (s, 2H), 4.03 (sept, *J* = 6.6 Hz, 1H), 1.44 (d, *J* = 6.6 Hz, 6H), 1.24 (d, *J* = 6.8 Hz, 6H); ^{13}C NMR (100 MHz, Chloroform-d [77.0 ppm], ppm) δ = 162.9 (C_q), 162.8 (d, *J* = 248.1 Hz, C_q), 135.4 (CH), 130.6 (d, *J* = 3.9 Hz, C_q), 130.0 (d, *J* = 7.7 Hz, CH, 2C), 115.9 (d, *J* = 21.6 Hz, CH, 2C), 115.0 (C_q), 81.5 (C_q), 55.9 (CH_2), 49.7 (CH), 46.4 (CH), 23.2 (CH_3, 2C), 19.1 (CH_3, 2C); ^{19}F NMR (375 MHz, Chloroform-d, ppm) δ = -112.98; MS (FAB, 3-NBA): *m/z* (%) = 330 (20) $[M+2H]^+$, 329 (100) $[M+H]^+$, 328 (20) $[M]^+$, 228 (35), 154 (15), 147 (11), 136 (21), 109 (66), 100 (19), 95 (11), 91 (13). HRMS–FAB *(m/z)*: $[M+H]^+$ Calcd for $C_{17}H_{22}N_6F$, 329.1890; Found, 329.1891; IR (ATR, ṽ) = 3126 (w), 3074 (vw), 2978 (w), 2932 (w), 2873 (w), 2812 (vw), 2220 (vs), 2164 (vw), 1795 (vw), 1669 (vw), 1608 (w), 1538 (s), 1510 (vs), 1460 (w), 1448 (w), 1412 (vs), 1374 (s), 1368 (vs), 1354 (s), 1330 (w), 1261 (vs), 1222 (vs), 1153 (vs), 1132 (s), 1099 (s), 1030 (s), 1007 (s), 959 (w), 943 (w), 911 (w), 882 (w), 857 (m),

836 (vs), 792 (s), 765 (s), 718 (s), 691 (m), 642 (m), 628 (m), 585 (w), 561 (w), 535 (m), 524 (m), 514 (m), 482 (s), 459 (w), 421 (w), 378 (w) cm^{-1}.

R_f = 0.38 (cyclohexane/ethyl acetate 4:1). MP = 94.6–96.1 °C. ^{1}H NMR (400 MHz, Chloroform-d [7.27 ppm], ppm) δ = 7.67 (s, 1H), 7.29–7.26 (m, 2H), 7.04–7.00 (m, 2H), 5.36–5.34 (m, 2H), 5.20 (sept, *J* = 6.1 Hz, 1H), 4.13 (sept, *J* = 6.7 Hz, 1H), 1.48 (d, *J* = 6.7 Hz, 6H), 1.31 (d, *J* = 6.8 Hz, 6H); ^{13}C NMR (100 MHz, Chloroform-d [77.0 ppm], ppm) δ = 162.3 (d, *J* = 246.6 Hz, C_q), 154.5 (C_q), 142.3 (CH), 132.1 (d, *J* = 3.1 Hz, C_q), 129.5 (d, *J* = 7.7 Hz, CH, 2C), 115.6 (C_q), 115.5 (d, *J* = 21.6 Hz, CH, 2C), 77.8 (C_q), 51.3 (CH_2), 51.3 (CH), 48.0 (CH), 23.0 (CH_3, 2C), 18.8 (CH_3, 2C); ^{19}F NMR (375 MHz, Chloroform-d, ppm) δ = -114.33; MS (FAB, 3-NBA): *m/z* (%) = 330 (21) $[M+2H]^+$, 329 (100) $[M+H]^+$, 328 (32) $[M]^+$, 154 (15), 136 (11), 109 (39), 100 (14), 97 (15), 95 (13). HRMS–FAB *(m/z)*: $[M+H]^+$ Calcd for $C_{17}H_{22}N_6F$, 329.1890; Found, 329.1889; IR (ATR, ṽ) = 3116 (w), 3060 (vw), 2982 (w), 2962 (w), 2936 (w), 2873 (w), 2856 (w), 2220 (s), 1874 (vw), 1762 (vw), 1608 (w), 1536 (m), 1510 (s), 1490 (w), 1469 (w), 1460 (w), 1417 (vs), 1391 (vs), 1377 (vs), 1364 (vs), 1313 (w), 1273 (vs), 1241 (s), 1214 (vs), 1164 (s), 1154 (vs), 1142 (s), 1133 (m), 1103 (vs), 1038 (w), 1026 (vs), 955 (w), 935 (w), 925 (w), 911 (w), 884 (s), 846 (w), 816 (vs), 782 (m), 756 (w), 728 (w), 705 (m), 693 (w), 662 (w), 642 (w), 620 (w), 589 (w), 551 (w), 533 (m), 509 (vs), 492 (vs), 463 (w), 456 (w), 445 (vw), 426 (w), 415 (w) cm^{-1}.

Additional information on the chemical synthesis is available *via* Chemotion repository: https://doi.org/10.14272/reaction/SA-FUHFF-UHFFFADPSC-ZIHLMBSODJ-UHFFFADPSC-NUHFF-NPQQK-NUHFF-ZZZ

Additional information on the analysis of the target compound is available *via* Chemotion repository:
https://doi.org/10.14272/ADGUCVKMBUJWJM-LSDHQDQOSA-N.1
https://doi.org/10.14272/HEZWXNUXEKJBQT-QURGRASLSA-N.1

5-Azido-1*H*-pyrazole (19a)

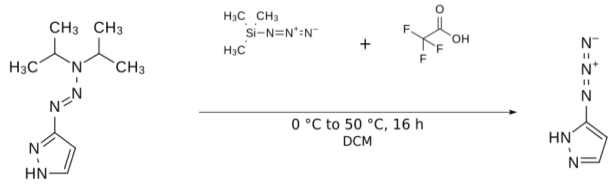

Name {P1|**19a**}: 5-azido-1*H*-pyrazole; Formula: $C_3H_3N_5$; Smiles: [N-]=[N+]=Nc1cc[nH]n1. InChIKey: KKKGXLQRKPKJON-UHFFFAOYSA-N

(*E*)-3-(3,3-Diisopropyltriaz-1-en-1-yl)-1*H*-pyrazole (200 mg, 1.02 mmol, 1.00 equiv) was dissolved in methylene chloride (15.0 mL). The solution was cooled to 0 °C and then azido(trimethyl)silane (885 mg, 1.02 mL, 7.68 mmol, 7.50 equiv) and 2,2,2-trifluoroacetic acid (1.17 g, 784 µL, 10.2 mmol, 10.00 equiv) were added. The reaction mixture was slowly warmed to 50 °C while constantly being stirred. The reaction progress was monitored *via* TLC. After full conversion (16 h), the solvent was reduced under vacuum. As preparation for the column chromatography (dryload), Celite was added (0.6 g) and the reaction mixture with Celite were evaporated. The obtained crude product was purified *via* flash-chromatography (Interchim devices puriFLASH

5.125) on silica gel (PF-15SIHP-F0025) using cyclohexane/ethyl acetate 0% to 35% ethyl acetate in 15 column volumes. The isolated product 5-azido-1*H*-pyrazole (107 mg, 980 µmol) was obtained as a light yellow oil in 96% yield.

R_f = 0.52 (cyclohexane/ethyl acetate 1:1). ^{1}H NMR (400 MHz, Chloroform-d [7.27 ppm], ppm) δ = 10.22 (s, 1H), 7.55 (d, *J* = 2.4 Hz, 1H), 6.02 (d, *J* = 2.4 Hz, 1H); ^{13}C NMR (100 MHz, Chloroform-d [77.0 ppm], ppm) δ = 148.5, 130.7, 96.1; MS (EI, 70 eV, 20 °C), m/z (%): 109 (44) $[M]^+$, 81 (31), 52 (100). HRMS–EI ($C_3H_3N_5$) *(m/z)*: $[M]^+$ Calcd 109.0383; Found 109.0384; IR (ATR, ṽ) = 3160 (vs), 3146 (vs), 3126 (vs), 3054 (s), 2975 (vs), 2928 (vs), 2840 (s), 2785 (m), 2765 (m), 2704 (w), 2656 (w), 2529 (w), 2492 (w), 2404 (w), 2220 (vw), 2180 (w), 2108 (vs), 2074 (vs), 2026 (m), 2011 (m), 1994 (m), 1952 (w), 1742 (w), 1628 (w), 1575 (w), 1537 (s), 1480 (vs), 1465 (vs), 1361 (s), 1305 (vs), 1222 (vs), 1186 (vs), 1089 (m), 1061 (w), 1044 (vs), 1007 (m), 993 (s), 922 (s), 873 (w), 799 (s), 751 (vs), 745 (vs), 680 (m), 602 (vs), 569 (w), 528 (vs), 486 (w), 399 (vs), 378 (w) cm^{-1}.

Additional information on the chemical synthesis is available *via* Chemotion repository: https://doi.org/10.14272/reaction/SA-FUHFF-UHFFFADPSC-KKKGXLQRKP-UHFFFADPSC-NUHFF-NUHFF-NUHFF-ZZZ

Additional information on the analysis of the target compound is available *via* Chemotion repository:
https://doi.org/10.14272/KKKGXLQRKPKJON-UHFFFAOYSA-N.1

This compound has been previously synthesized in literature. [6]

3-Azido-1-cyclopentylpyrazole (19b)

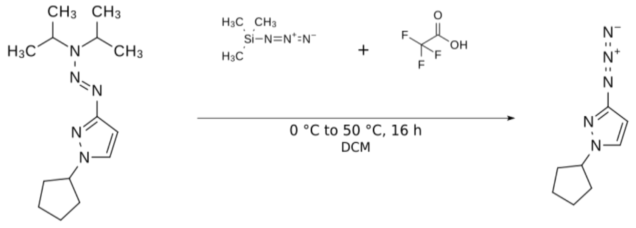

Name {P1|**19b**}: 3-azido-1-cyclopentylpyrazole; Formula: $C_8H_{11}N_5$; Smiles: [N-]=[N+]=Nc1ccn(n1)C1CCCC1. InChIKey: CEJMUQXTWWQJBT-UHFFFAOYSA-N

(*E*)-1-Cyclopentyl-3-(3,3-diisopropyltriaz-1-en-1-yl)-1*H*-pyrazole (211 mg, 801 µmol, 1.00 equiv) was dissolved in methylene chloride (20 mL). The solution was cooled to 0 °C and then azido(trimethyl)silane (692 mg, 797 µL, 6.01 mmol, 7.50 equiv) and 2,2,2-trifluoroacetic acid (913 mg, 613 µL, 8.01 mmol, 10.0 equiv) were added. The reaction mixture was slowly warmed to 50 °C while constantly stirring. The reaction progress was monitored *via* TLC. The reaction was finished after 16 hours of stirring at 50 °C. As preparation for the column chromatography (dry load), Celite was added (0.6 g) and the reaction mixture with Celite was evaporated. The obtained crude product was purified *via* flash-chromatography (Interchim devices puriFLASH 5.125) on silica gel (PF-15SIHP-F0040) using cyclohexane/ethyl acetate 0% to 20% ethyl acetate in 15 column volumes (1 column volume = 91.9 mL; flow: 26 mL/min). The isolated product 3-azido-1-cyclopentyl-pyrazole (134 mg, 753 µmol) was obtained as a yellow oil in 94% yield.

R_f = 0.90 (cyclohexane/ethyl acetate 2:1). ^{1}H NMR (400 MHz, Chloroform-d [7.27 ppm], ppm) δ = 7.31 (d, *J* = 2.3 Hz, 1H), 5.86 (d, *J* = 2.4 Hz, 1H), 4.53 (quint, *J* = 7.1 Hz, 1H), 2.16–2.07 (m, 2H), 2.03–1.94 (m, 2H), 1.91–1.81 (m, 2H), 1.74–1.63 (m, 2H); ^{13}C NMR (100 MHz, Chloroform-d [77.0 ppm], ppm) δ = 147.0 (C_q), 129.3 (CH), 95.4 (CH), 63.3 (CH), 32.8 (CH_2, 2C), 24.0 (CH_2, 2C); MS (EI, 70 eV, 20 °C), m/z (%): 177 (27) $[M]^+$, 69 (100). HRMS–EI ($C_8H_{11}N_5$) *(m/z)*: $[M]^+$ Calcd 177.1009; Found 177.1008; IR (ATR, ṽ) = 2958 (w), 2873 (w), 2115 (vs), 1510 (s), 1472 (vs), 1424 (s), 1374 (m), 1353 (m), 1313 (w), 1239 (m), 1177 (w), 1045 (m), 994 (m), 942 (w), 895 (w), 833 (w), 785 (m), 742 (s), 633 (m), 530 (m), 397 (m), 382 (m) cm^{-1}.

Additional information on the chemical synthesis is available *via* Chemotion repository: https://doi.org/10.14272/reaction/SA-FUHFF-UHFFFADPSC-CEJMUQXTWW-UHFFFADPSC-NUHFF-NUHFF-NUHFF-ZZZ.1

Additional information on the analysis of the target compound is available *via* Chemotion repository:
https://doi.org/10.14272/CEJMUQXTWWQJBT-UHFFFAOYSA-N.2

3-Azido-1-benzyl-pyrazole (19c)

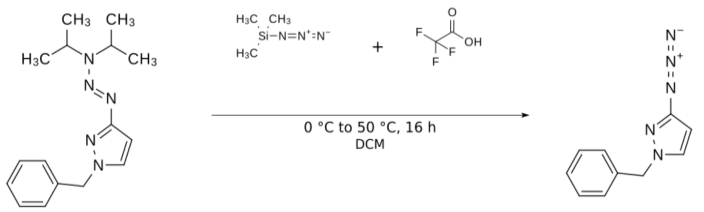

Name {P1|**19c**}: 3-azido-1-benzyl-pyrazole; Formula: $C_{10}H_9N_5$; Smiles: [N-]=[N+]=Nc1ccn(n1)Cc1ccccc1. InChIKey: VYAIIDYJDVIYCE-UHFFFAOYSA-N

(*E*)-1-Benzyl-3-(3,3-diisopropyltriaz-1-en-1-yl)-1*H*-pyrazole (522 mg, 1.83 mmol, 1.00 equiv) was dissolved in methylene chloride (30.0 mL). The solution was cooled to 0 °C and then azido(trimethyl)silane (1.58 g, 1.82 mL, 13.7 mmol, 7.50 equiv) and 2,2,2-trifluoroacetic acid (2.08 g, 1.40 mL, 18.3 mmol, 10.0 equiv) were added. The reaction mixture was slowly warmed to 50 °C while constantly being stirred. The reaction progress was monitored *via* TLC and the reaction was finished after 16 hours of stirring at 50 °C. As preparation for the column chromatography (dry load), Celite was added (1.5 g) and the reaction mixture with Celite was evaporated. The obtained crude product was purified *via* flash-chromatography (Interchim devices puriFLASH 5.125) on silica gel (PF-15SIHP-F0040) using cyclohexane/ethyl acetate with 0% to 20% ethyl acetate in 15 column volumes (1 column volume = 91.9 mL; flow: 26 mL/min). The isolated product 3-azido-1-benzyl-pyrazole (209 mg, 1.05 mmol) was obtained as an orange oil in 57% yield.

R_f = 0.72 (cyclohexane/ethyl acetate 2:1). ^{1}H NMR (400 MHz, Chloroform-d [7.27 ppm], ppm) δ = 7.44–7.36 (m, 3H), 7.33–7.31 (m, 1H), 7.29–7.26 (m, 2H), 6.01 (d, *J* = 2.3 Hz, 1H), 5.29 (s, 2H). ^{13}C NMR (100 MHz, Chloroform-d [77.0 ppm], ppm) δ = 148.0 (C_q), 135.4 (C_q), 131.7 (CH), 128.9 (CH, 2C), 128.4 (CH), 127.8 (CH, 2C), 96.7 (CH), 56.0 (CH_2); MS (EI, 70 eV, 30 °C), m/z (%): 199 (9) $[M]^+$, 91 (100), 65 (9). HRMS–EI ($C_{10}H_9N_5$) *(m/z)*: $[M]^+$ Calcd 199.0852; Found 199.0854; IR (ATR, ṽ) = 2115 (vs), 1514 (s), 1497 (w), 1473 (vs), 1455 (s), 1419 (s), 1374 (w), 1356 (w), 1326 (m), 1245 (s),

1203 (w), 1190 (w), 1163 (w), 1077 (w), 1047 (m), 1028 (w), 994 (w), 795 (w), 747 (s), 710 (s), 696 (m), 628 (w), 575 (w), 528 (m), 460 (w), 438 (w) cm^{-1}.

Additional information on the chemical synthesis is available *via* Chemotion repository: https://doi.org/10.14272/reaction/SA-FUHFF-UHFFFADPSC-VYAIIDYJDV-UHFFFADPSC-NUHFF-NUHFF-NUHFF-ZZZ

Additional information on the analysis of the target compound is available *via* Chemotion repository:
https://doi.org/10.14272/VYAIIDYJDVIYCE-UHFFFAOYSA-N.1

3-Azido-1-(4-bromobenzyl)pyrazole (19d)

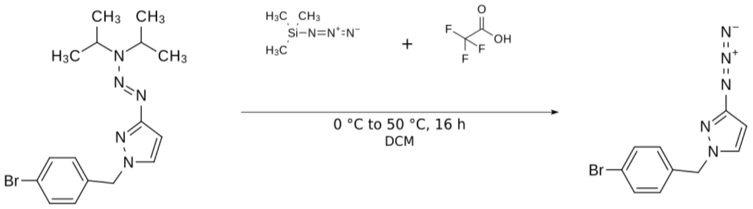

Name {P1|**19d**}: 3-azido-1-(4-bromobenzyl)pyrazole; Formula: $C_{10}H_8BrN_5$; Smiles: [N-]=[N+]=Nc1ccn(n1)Cc1ccc(cc1)Br. InChIKey: MPLVWOQSFNIWDU-UHFFFAOYSA-N

(*E*)-1-(4-Bromobenzyl)-3-(3,3-diisopropyltriaz-1-en-1-yl)-1*H*-pyrazole (288 mg, 789 µmol, 1.00 equiv) was dissolved in methylene chloride (20 mL). The solution was cooled to 0 °C and then azido(trimethyl)silane (682 mg, 786 µL, 5.92 mmol, 7.50 equiv) and 2,2,2-trifluoroacetic acid (900 mg, 604 µL, 7.89 mmol, 10.0 equiv) were added. The reaction mixture was slowly warmed to 50 °C while constantly being stirred. The reaction progress was monitored *via* TLC (16 h). As preparation for the column chromatography (dryload), Celite was added (0.9 g) and the reaction mixture with Celite were evaporated. The obtained crude product was purified *via* flash-chromatography (Interchim devices puriFLASH 5.125) on silica gel (PF-15SIHP-F0040) using cyclohexane/ethyl acetate 0% to 20% ethyl acetate in 15 column volumes (1 column volume = 91.9 mL; flow: 26 mL/min). The isolated product 3-azido-1-(4-bromobenzyl)pyrazole (205 mg, 737 µmol) was obtained as a light yellow solid in 93% yield.

R_f = 0.65 (cyclohexane/ethyl acetate 2:1). ^{1}H NMR (400 MHz, Chloroform-d [7.27 ppm], ppm) δ = 7.50–7.47 (m, 2H), 7.26 (d, *J* = 2.2 Hz, 1H), 7.12–7.09 (m, 2H), 5.92 (d, *J* = 2.2 Hz, 1H), 5.16 (s, 2H); ^{13}C NMR (100 MHz, Chloroform-d [77.0 ppm], ppm) δ = 148.1, 135.0, 132.0 (2C), 131.4, 129.3 (2C), 122.3, 96.9, 55.5; MS (EI, 70 eV, 80 °C), m/z (%): 277/279 (13/12) [M]$^+$, 169/171 (100/96), 90 (28). HRMS–EI ($C_{10}H_8N_5Br$) *(m/z)*: [M]$^+$ Calcd 276.9958; Found 276.9957; IR (ATR, ṽ) = 3132 (w), 3111 (w), 2946 (vw), 2152 (vw), 2119 (vs), 2034 (w), 1711 (vw), 1592 (vw), 1510 (s), 1487 (w), 1475 (vs), 1438 (m), 1419 (s), 1405 (m), 1367 (w), 1349 (w), 1323 (m), 1315 (m), 1298 (w), 1247 (vs), 1203 (w), 1179 (w), 1157 (w), 1106 (w), 1068 (m), 1060 (s), 1011 (m), 996 (w), 976 (vw), 962 (w), 861 (vw), 841 (m), 816 (w), 803 (m), 785 (s), 766 (vs), 737 (vs), 681 (s), 637 (m), 608 (w), 527 (vs), 487 (s), 446 (s), 404 (w), 381 (m) cm^{-1}.

Additional information on the chemical synthesis is available *via* Chemotion repository:
https://doi.org/10.14272/reaction/SA-FUHFF-UHFFFADPSC-MPLVWOQSFN-UHFFFADPSC-NUHFF-NUHFF-NUHFF-ZZZ

Additional information on the analysis of the target compound is available *via* Chemotion repository:
https://doi.org/10.14272/MPLVWOQSFNIWDU-UHFFFAOYSA-N.1

3-Azido-5-methyl-1*H*-pyrazole (19e)

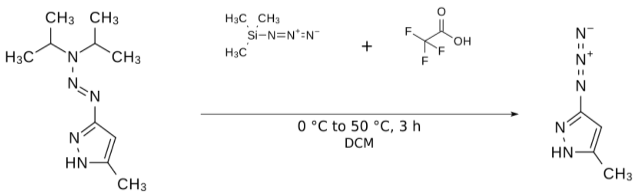

Name {P1|**19e**}: 3-azido-5-methyl-1*H*-pyrazole; Formula: $C_4H_5N_5$; Smiles: [N-]=[N+]=Nc1cc([nH]n1)C. InChIKey: ASYITVIZQLPDSI-UHFFFAOYSA-N

(*E*)-1-(4-bromobenzyl)-3-(3,3-diisopropyltriaz-1-en-1-yl)-1*H*-pyrazole (288 mg, 789 µmol, 1.00 equiv) was dissolved in methylene chloride (20 mL). The solution was cooled to 0 °C and then azido(trimethyl)silane (682 mg, 786 µL, 5.92 mmol, 7.50 equiv) and 2,2,2-trifluoroacetic acid (900 mg, 604 µL, 7.89 mmol, 10.0 equiv) were added. The reaction mixture was slowly warmed to 50 °C while constantly being stirred. The reaction progress was monitored *via* TLC. The reaction was finished after 16 hours stirring at 50 °C. As preparation for the column chromatography (dry load), Celite was added (0.6 g) and the reaction mixture with additional Celite was evaporated. The obtained crude product was purified *via* flash-chromatography (Interchim devices puriFLASH 5.125) on silica gel (PF-15SIHP-F0025) using cyclohexane/ethyl acetate with 0% to 50% ethyl acetate in 25 column volumes (1 column volume = 63.8 mL; flow: 15 mL/min). The isolated product 3-azido-5-methyl-1*H*-pyrazole (109 mg, 886 µmol) was obtained as a light brown solid in 93% yield.

R_f = 0.55 (cyclohexane/ethyl acetate 1:1). MP = 112.3–115 °C. ^{1}H NMR (400 MHz, Chloroform-d [7.27 ppm], ppm) δ = 10.49 (br, s, 1H), 5.77 (s, 1H), 2.33 (s, 3H); ^{13}C NMR (100 MHz, Chloroform-d [77.0 ppm], ppm) δ = 148.5, 141.7, 95.1, 11.2; MS (EI, 70 eV, 30 °C), m/z (%): 123 (100) $[M]^+$, 95 (48), 66 (32), 64 (20), 52 (31). HRMS–EI ($C_4H_5N_5$) *(m/z)*: $[M]^+$ Calcd 123.0539; Found 123.0541; IR (ATR, ṽ) = 3196 (w), 3129 (m), 3108 (m), 3082 (w), 3033 (w), 2982 (m), 2953 (m), 2925 (m), 2874 (m), 2851 (m), 2775 (m), 2731 (m), 2591 (w), 2516 (w), 2490 (w), 2397 (w), 2380 (w), 2116 (vs), 2038 (m), 2026 (m), 1655 (w), 1592 (s), 1453 (vs), 1432 (vs), 1380 (m), 1307 (m), 1261 (s), 1248 (vs), 1163 (s), 1055 (w), 1021 (vs), 1000 (vs), 860 (s), 817 (s), 795 (s), 761 (s), 747 (vs), 738 (vs), 683 (w), 664 (s), 633 (w), 527 (m), 516 (m), 445 (m), 381 (w) cm^{-1}.

Additional information on the chemical synthesis is available *via* Chemotion repository:
https://doi.org/10.14272/reaction/SA-FUHFF-UHFFFADPSC-ASYITVIZQL-UHFFFADPSC-NUHFF-NUHFF-NUHFF-ZZZ

Additional information on the analysis of the target compound is available *via* Chemotion repository: https://doi.org/10.14272/ASYITVIZQLPDSI-UHFFFAOYSA-N.1

This compound has been previously synthesized in literature.[7]

3-Azido-1-benzyl-5-methyl-pyrazole (19f)

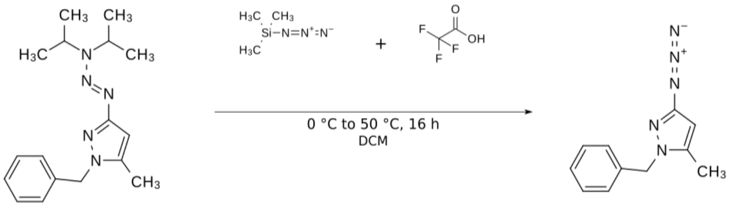

Name {P1|**19f**}: 3-azido-1-benzyl-5-methyl-pyrazole; Formula: $C_{11}H_{11}N_5$; Smiles: [N-]=[N+]=Nc1nn(c(c1)C)Cc1ccccc1. InChIKey: CUZSOFWTCLMWBL-UHFFFAOYSA-N

(*E*)-1-benzyl-3-(3,3-diisopropyltriaz-1-en-1-yl)-5-methyl-1*H*-pyrazole (157 mg, 524 µmol, 1.00 equiv) was dissolved in methylene chloride (10.0 mL). The solution was cooled to 0 °C and then azido(trimethyl)silane (453 mg, 522 µL, 3.93 mmol, 7.50 equiv) and 2,2,2-trifluoroacetic acid (598 mg, 402 µL, 5.24 mmol, 10.0 equiv) were added. The reaction mixture was slowly warmed to 50 °C while constantly being stirred. The reaction progress was monitored *via* TLC and the reaction was finished after 16 hours of stirring at 50 °C. As preparation for the column chromatography (dry load), Celite was added (0.5 g) and the reaction mixture with Celite was evaporated. The obtained crude product was purified *via* flash-chromatography (Interchim devices puriFLASH 5.125) on silica gel (PF-15SIHP-F0025) using cyclohexane/ethyl acetate with 0% to 20% ethyl acetate in 15 column volumes (1 column volume = 63.8 mL; flow: 15 mL/min). The isolated product 3-azido-1-benzyl-5-methyl-pyrazole (109 mg, 509 µmol) was obtained as a brown oil in 97% yield.

R_f = 0.67 (cyclohexane/ethyl acetate 2:1). ^{1}H NMR (400 MHz, Chloroform-d [7.27 ppm], ppm) δ = 7.32–7.25 (m, 3H), 7.11–7.09 (m, 2H), 5.74 (d, *J* = 0.5 Hz, 1H), 5.19 (s, 2H), 2.16 (d, *J* = 0.6 Hz, 3H); ^{13}C NMR (100 MHz, Chloroform-d [77.0 ppm], ppm) δ = 146.7 (C_q), 141.0 (C_q), 136.4 (C_q), 128.8 (CH, 2C), 127.7 (CH), 126.7 (CH, 2C), 96.6 (CH), 52.9 (CH_2), 11.4 (CH_3); MS (EI, 70 eV, 20 °C), m/z (%): 213 (10) $[M]^+$, 91 (100). HRMS–EI ($C_{11}H_{11}N_5$) *(m/z)*: $[M]^+$ Calcd 213.1009; Found 213.1008; IR (ATR, ṽ) = 2932 (vw), 2257 (vw), 2116 (vs), 1544 (s), 1496 (w), 1483 (m), 1451 (vs), 1388 (m), 1356 (w), 1317 (m), 1300 (w), 1237 (m), 1200 (w), 1180 (w), 1157 (w), 1123 (w), 1077 (w), 1028 (w), 1017 (w), 1011 (w), 979 (w), 963 (w), 899 (w), 830 (w), 766 (m), 724 (s), 697 (s), 671 (w), 633 (w), 618 (w), 603 (w), 575 (w), 530 (w), 517 (w), 503 (w), 489 (w), 455 (w) cm^{-1}.

Additional information on the chemical synthesis is available *via* Chemotion repository: https://doi.org/10.14272/reaction/SA-FUHFF-UHFFFADPSC-CUZSOFWTCL-UHFFFADPSC-NUHFF-NUHFF-NUHFF-ZZZ

Additional information on the analysis of the target compound is available *via* Chemotion repository:
https://doi.org/10.14272/CUZSOFWTCLMWBL-UHFFFAOYSA-N.1

4-(3-Azido-5-methyl-1*H*-pyrazol-1-yl)benzonitrile (**19g**)

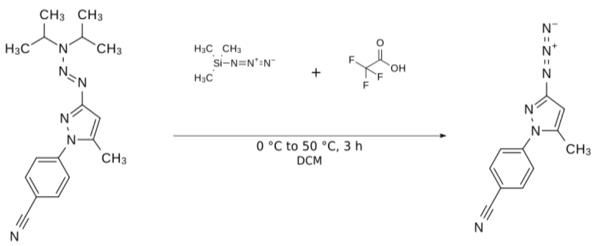

Name {P1|**19g**}: 4-(3-azido-5-methyl-1*H*-pyrazol-1-yl)benzonitrile; Formula: $C_{11}H_8N_6$; Smiles: [N-]=[N+]=Nc1nn(c(c1)C)c1ccc(cc1)C#N. InChIKey: KIQBPEXSAZKVRR-UHFFFAOYSA-N

(*E*)-4-(3-(3,3-Diisopropyltriaz-1-en-1-yl)-5-methyl-1*H*-pyrazol-1-yl)benzonitrile (134 mg, 431 µmol, 1.00 equiv) was dissolved in methylene chloride (10.0 mL). The solution was cooled to 0 °C and then azido(trimethyl)silane (372 mg, 429 µL, 3.23 mmol, 7.50 equiv) and 2,2,2-trifluoroacetic acid (492 mg, 330 µL, 4.31 mmol, 10.00 equiv) were added. The reaction mixture was slowly warmed to 50 °C while constantly being stirred. The reaction progress was monitored *via* TLC (3 h). As preparation for the column chromatography (dryload), Celite was added (0.5 g) and the reaction mixture with additional Celite was evaporated. The obtained crude product was purified *via* flash-chromatography (Interchim devices puriFLASH 5.125) on silica gel (PF-15SIHP-F0025) using cyclohexane/ethyl acetate 0% to 35% ethyl acetate in 20 column volumes (1 column volume = 63.8 mL; flow: 15 mL/min). The isolated product 4-(3-azido-5-methyl-1*H*-pyrazol-1-yl)benzonitrile (84.9 mg, 379 µmol) was obtained as a colorless solid in 88% yield. The reaction was repeated with a reaction time of 16 h and a yield of 96%.

R_f = 0.58 (cyclohexane/ethyl acetate 2:1). MP = 94.1–95.4 °C. ^{1}H NMR (400 MHz, Chloroform-d [7.27 ppm], ppm) δ = 7.78–7.75 (m, 2H), 7.64–7.61 (m, 2H), 5.93 (s, 1H), 2.42 (s, 3H); ^{13}C NMR (100 MHz, Chloroform-d [77.0 ppm], ppm) δ = 149.6 (C_q), 142.7 (C_q), 141.5 (C_q), 133.2 (2C, CH), 123.9 (2C, CH), 118.2 (C_q), 110.6 (C_q), 100.0 (CH), 13.3 (CH_3); MS (EI, 70 eV, 40 °C), m/z (%): 224 (23) $[M]^+$, 196 (15), 130 (53), 102 (100), 75 (12), 58 (17). HRMS–EI ($C_{11}H_8N_6$) *(m/z)*: $[M]^+$ Calcd 224.0805; Found 224.0803; IR (ATR, ṽ) = 3135 (w), 3072 (w), 2921 (w), 2470 (vw), 2388 (vw), 2357 (vw), 2264 (vw), 2228 (m), 2121 (vs), 2051 (w), 1915 (vw), 1606 (m), 1581 (w), 1547 (s), 1511 (vs), 1476 (s), 1451 (vs), 1432 (vs), 1377 (vs), 1315 (w), 1298 (w), 1271 (w), 1238 (vs), 1181 (w), 1146 (w), 1111 (w), 1030 (w), 1014 (w), 977 (m), 843 (vs), 823 (w), 812 (w), 793 (m), 783 (vs), 722 (w), 693 (w), 677 (w), 654 (w), 620 (w), 575 (m), 560 (s), 547 (s), 530 (m), 518 (s), 500 (w), 458 (m), 404 (w) cm^{-1}.

Additional information on the chemical synthesis is available *via* Chemotion repository:
https://doi.org/10.14272/reaction/SA-FUHFF-UHFFFADPSC-KIQBPEXSAZ-UHFFFADPSC-NUHFF-NUHFF-NUHFF-ZZZ
https://dx.doi.org/10.14272/reaction/SA-FUHFF-UHFFFADPSC-KIQBPEXSAZ-UHFFFADPSC-NUHFF-NUHFF-NUHFF-ZZZ.2

Additional information on the analysis of the target compound is available *via* Chemotion repository:
https://doi.org/10.14272/KIQBPEXSAZKVRR-UHFFFAOYSA-N.1
https://dx.doi.org/10.14272/KIQBPEXSAZKVRR-UHFFFAOYSA-N.3

3-Azido-5-methyl-1-(4-nitrophenyl)-1*H*-pyrazole (19h)

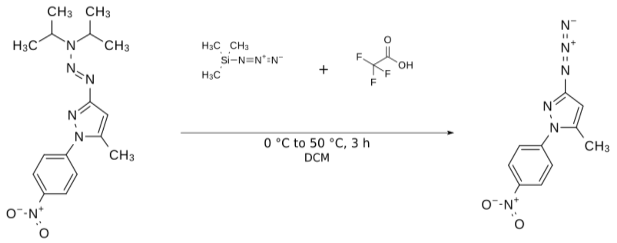

Name {P1|**19h**}: 3-azido-5-methyl-1-(4-nitrophenyl)-1H-pyrazole; Formula: $C_{10}H_8N_6O_2$; Smiles: [N-]=[N+]=Nc1nn(c(c1)C)c1ccc(cc1)[N+](=O)[O-]. InChIKey: HISYWPICEGTOTQ-UHFFFAOYSA-N

(*E*)-3-(3,3-Diisopropyltriaz-1-en-1-yl)-5-methyl-1-(4-nitrophenyl)-1*H*-pyrazole (100 mg, 303 µmol, 1.00 equiv) was dissolved in methylene chloride (10.0 mL). The solution was cooled to 0 °C and then azido(trimethyl)silane (262 mg, 301 µL, 2.27 mmol, 7.50 equiv) and 2,2,2-trifluoroacetic acid (345 mg, 232 µL, 3.03 mmol, 10.00 equiv) were added. The reaction mixture was slowly warmed to 50 °C while constantly being stirred. The reaction progress was monitored *via* TLC (3h). As preparation for the column chromatography (dryload), Celite was added (0.3 g) and the reaction mixture with additional Celite was evaporated. The obtained crude product was purified *via* flash-chromatography (Interchim devices puriFLASH 5.125) on silica gel (PF-15SIHP-F0025) using cyclohexane/ethyl acetate 0% to 20% ethyl acetate in 25 column volumes. The isolated product 3-azido-5-methyl-1-(4-nitrophenyl)-1*H*-pyrazole (62.3 mg, 255 µmol) was obtained as a yellow solid in 84% yield.

R_f = 0.21 (cyclohexane/ethyl acetate 10:1). MP = 102.2–106 °C. ^{1}H NMR (400 MHz, Chloroform-d [7.27 ppm], ppm) δ = 8.37–8.33 (m, 2H), 7.71–7.67 (m, 2H), 5.96 (s, 1H), 2.46 (s, 3H); ^{13}C NMR (100 MHz, Chloroform-d [77.0 ppm], ppm) δ = 149.9, 145.9, 144.3, 141.7, 124.8 (2C), 123.6 (2C), 100.4, 13.5; MS (EI, 70 eV, 70 °C), m/z (%): 244 (19) $[M]^+$, 216 (24), 150 (98), 122 (100), 117 (16), 92 (26), 76 (73), 66 (23). HRMS–EI ($C_{10}H_8O_2N_6$) *(m/z)*: $[M]^+$ Calcd 244.0703; Found 244.0704; IR (ATR, ṽ) = 3122 (w), 3091 (w), 2915 (w), 2122 (vs), 2030 (w), 1606 (m), 1595 (vs), 1555 (s), 1511 (vs), 1500 (vs), 1460 (vs), 1432 (s), 1380 (s), 1339 (vs), 1326 (vs), 1272 (s), 1249 (vs), 1181 (m), 1137 (s), 1108 (vs), 1026 (s), 1006 (s), 979 (vs), 849 (vs), 815 (m), 773 (vs), 749 (vs), 731 (s), 693 (s), 676 (s), 636 (m), 619 (w), 557 (m), 540 (m), 526 (m), 490 (m), 473 (s), 422 (w), 401 (m) cm^{-1}.

Additional information on the chemical synthesis is available *via* Chemotion repository: https://doi.org/10.14272/reaction/SA-FUHFF-UHFFFADPSC-HISYWPICEG-UHFFFADPSC-NUHFF-NUHFF-NUHFF-ZZZ

Additional information on the analysis of the target compound is available *via* Chemotion repository: https://doi.org/10.14272/HISYWPICEGTOTQ-UHFFFAOYSA-N.1

3-Azido-1-(2-chloro-4-nitrophenyl)-5-methyl-1*H*-pyrazole (**19i**)

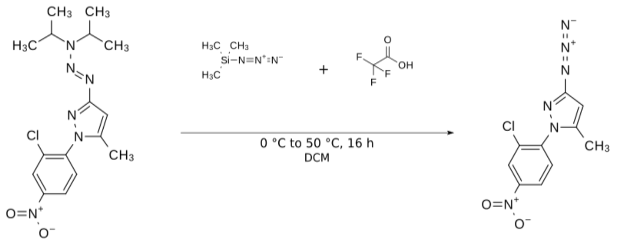

Name {P1|**19i**}: 3-azido-1-(2-chloro-4-nitrophenyl)-5-methyl-1*H*-pyrazole; Formula: $C_{10}H_7ClN_6O_2$; Smiles: [N-]=[N+]=Nc1nn(c(c1)C)c1ccc(cc1Cl)[N+](=O)[O-]. InChIKey: GQMYHPXVWDUZCD-UHFFFAOYSA-N

(*E*)-1-(2-chloro-4-nitrophenyl)-3-(3,3-diisopropyltriaz-1-en-1-yl)-5-methyl-1*H*-pyrazole (460 mg, 1.26 mmol, 1.00 equiv) was dissolved in methylene chloride (30.0 mL). The solution was cooled to 0 °C and then azido(trimethyl)silane (1.09 g, 1.26 mL, 9.46 mmol, 7.50 equiv) and 2,2,2-trifluoroacetic acid (1.44 g, 966 µL, 12.6 mmol, 10.00 equiv) were added. The reaction mixture was slowly warmed to 50 °C while constantly being stirred. The reaction progress was monitored *via* TLC. After full conversion (16 h), the solvent was reduced under vacuum. As preparation for column chromatography (dry load), Celite was added (1.5 g) and the reaction mixture with Celite was evaporated. The obtained crude product was purified *via* flash-chromatography (Interchim devices puriFLASH 5.125) on silica gel (PF-15SIHP-F0080) using cyclohexane/ethyl acetate with 0% to 30% ethyl acetate in 15 column volumes. The isolated product 3-azido-1-(2-chloro-4-nitrophenyl)-5-methyl-1*H*-pyrazole (351 mg, 1.26 mmol) was obtained as an orange oil in quantitative yield.

R_f = 0.61 (cyclohexane/ethyl acetate 2:1). ^{1}H NMR (400 MHz, Chloroform-d [7.27 ppm], ppm) δ = 8.44 (d, *J* = 2.4 Hz, 1H), 8.27 (dd, *J* = 2.4 Hz, *J* = 8.7 Hz, 1H), 7.63 (d, *J* = 8.7 Hz, 1H), 5.93 (d, *J* = 0.5 Hz, 1H), 2.17 (d, *J* = 0.5 Hz, 3H); ^{13}C NMR (100 MHz, Chloroform-d [77.0 ppm], ppm) δ = 150.0 (C_q), 148.1 (C_q), 143.2 (C_q), 142.0 (C_q), 133.7 (C_q), 130.8 (CH), 125.8 (CH), 122.7 (CH), 98.0 (CH), 11.7 (CH_3); MS (EI, 70 eV, 70 °C), m/z (%): 280/278 (7/21) $[M]^+$, 250/252 (14/5), 186/184 (33/100), 158/156 (23/71), 128/126 (5/14), 112/110 (10/29), 75 (31), 74, 66 (15). HRMS–EI ($C_{10}H_7O_2N_6Cl$) *(m/z)*: $[M]^+$ Calcd 278.0314; Found 278.0313; IR (ATR, ṽ) = 3106 (vw), 3087 (vw), 2928 (vw), 2864 (vw), 2255 (vw), 2118 (vs), 2047 (w), 1602 (w), 1587 (w), 1551 (s), 1528 (vs), 1496 (vs), 1448 (vs), 1434 (vs), 1402 (m), 1374 (s), 1344 (vs), 1295 (m), 1237 (s), 1136 (m), 1118 (m), 1071 (w), 1038 (w), 1017 (m), 976 (w), 888 (s), 840 (m), 812 (m), 766 (s), 745 (s), 715 (m), 693 (w), 669 (w), 633 (w), 554 (w), 531 (w), 490 (w), 473 (w), 438 (w) cm^{-1}.

Additional information on the chemical synthesis is available *via* Chemotion repository: https://doi.org/10.14272/reaction/SA-FUHFF-UHFFFADPSC-GQMYHPXVWD-UHFFFADPSC-NUHFF-NUHFF-NUHFF-ZZZ

Additional information on the analysis of the target compound is available *via* Chemotion repository:
https://doi.org/10.14272/GQMYHPXVWDUZCD-UHFFFAOYSA-N.1

Ethyl 5-azido-1*H*-pyrazole-4-carboxylate (19j)

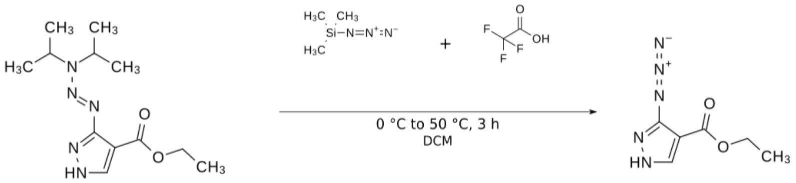

Name {P1|**19j**}: ethyl 5-azido-1*H*-pyrazole-4-carboxylate; Formula: $C_6H_7N_5O_2$; Smiles: [N-]=[N+]=Nc1n[nH]cc1C(=O)OCC. InChIKey: FSJHWJVEYJIYBP-UHFFFAOYSA-N

Ethyl (*E*)-3-(3,3-diisopropyltriaz-1-en-1-yl)-1*H*-pyrazole-4-carboxylate (122 mg, 456 µmol, 1.00 equiv) was dissolved in methylene chloride (10.0 mL). The solution was cooled to 0 °C and then azido(trimethyl)silane (394 mg, 454 µL, 3.42 mmol, 7.50 equiv) and 2,2,2-trifluoroacetic acid (520 mg, 349 µL, 4.56 mmol, 10.00 equiv) were added. The reaction mixture was slowly warmed to 50 °C while constantly being stirred. The reaction progress was monitored *via* TLC and was finished after 3 hours. As preparation for the column chromatography (dryload), Celite was added (0.5 g) and the reaction mixture with additional Celite was evaporated. The obtained crude product was purified *via* flash-chromatography (Interchim devices puriFLASH 5.125) on silica gel (PF-15SIHP-F0025) using cyclohexane/ethyl acetate 0% to 35% ethyl acetate in 20 column volumes (1 column volume = 63.8 mL; flow: 15 mL/min). The isolated product ethyl 5-azido-1*H*-pyrazole-4-carboxylate (74.4 mg, 411 µmol) was obtained as a colorless solid in 90% yield.

R_f = 0.29 (cyclohexane/ethyl acetate 2:1). MP = 92.2–93.7 °C. ^{1}H NMR (400 MHz, Chloroform-d [7.27 ppm], ppm) δ = 9.32 (br.s, 1H), 8.02 (s, 1H), 4.33 (q, J = 7.2 Hz, 2H), 1.37 (t, J = 7.2 Hz, 3H); ^{13}C NMR (100 MHz, Chloroform-d [77.0 ppm], ppm) δ = 161.8, 148.5, 134.6, 105.2, 60.7, 14.3; MS (EI, 70 eV, 70 °C), m/z (%): 181 (30) $[M]^+$, 136 (18), 81 (25), 68 (11), 52 (100). HRMS–EI ($C_6H_7O_2N_5$) *(m/z)*: $[M]^+$ Calcd 181.0594; Found 181.0596; IR (ATR, ṽ) = 3230 (s), 2992 (w), 2962 (w), 2946 (w), 2907 (w), 2121 (vs), 1681 (vs), 1547 (vs), 1500 (vs), 1472 (vs), 1448 (s), 1392 (m), 1368 (m), 1324 (vs), 1276 (w), 1197 (vs), 1176 (vs), 1113 (w), 1085 (vs), 1017 (s), 933 (m), 871 (w), 851 (w), 781 (m), 761 (vs), 684 (w), 605 (vs), 531 (m), 492 (m), 401 (m) cm^{-1}.

Additional information on the chemical synthesis is available *via* Chemotion repository: https://doi.org/10.14272/reaction/SA-FUHFF-UHFFFADPSC-FSJHWJVEYJ-UHFFFADPSC-NUHFF-NUHFF-NUHFF-ZZZ

Additional information on the analysis of the target compound is available *via* Chemotion repository:
https://doi.org/10.14272/FSJHWJVEYJIYBP-UHFFFAOYSA-N.1

This compound has been previously synthesized and the analytical data reported herein are consistent with the literature. [8]

3-Azido-1-benzyl-pyrazole-4-carboxylic acid ethyl ester (19k)

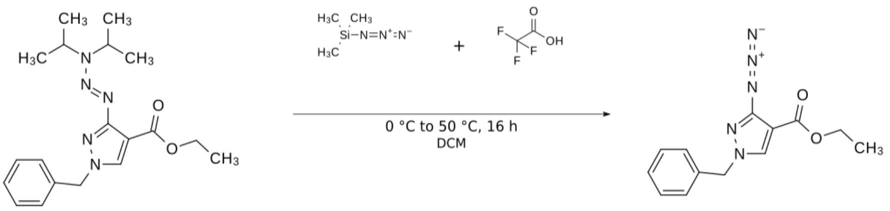

Name {P1|**19k**}: 3-azido-1-benzyl-pyrazole-4-carboxylic acid ethyl ester; Formula: $C_{13}H_{13}N_5O_2$; Smiles: CCOC(=O)c1cn(nc1N=[N+]=[N-])Cc1ccccc1. InChIKey: KCAXRYJMUFAPLD-UHFFFAOYSA-N

Ethyl (*E*)-1-benzyl-3-(3,3-diisopropyltriaz-1-en-1-yl)-1*H*-pyrazole-4-carboxylate (137 mg, 382 µmol, 1.00 equiv) was dissolved in methylene chloride (10.0 mL). The solution was cooled to 0 °C and then azido(trimethyl)silane (330 mg, 380 µL, 2.87 mmol, 7.50 equiv) and 2,2,2-trifluoroacetic acid (436 mg, 293 µL, 3.82 mmol, 10.00 equiv) were added. The reaction mixture was slowly warmed to 50 °C while constantly being stirred. The reaction progress was monitored *via* TLC. After full conversion (16 h), the solvent was reduced under vacuum. As preparation for the column chromatography (dryload), Celite was added (0.5 g) and the reaction mixture with additional Celite was evaporated. The obtained crude product was purified *via* flash-chromatography (Interchim devices puriFLASH 5.125) on silica gel (PF-15SIHP-F0025) using cyclohexane/ethyl acetate 0% to 30% ethyl acetate in 25 column volumes. The isolated product 3-azido-1-benzyl-pyrazole-4-carboxylic acid ethyl ester (53.2 mg, 196 µmol) was obtained as a colorless oil in 51% yield.

R_f = 0.60 (cyclohexane/ethyl acetate 2:1). ^{1}H NMR (400 MHz, Chloroform-d [7.27 ppm], ppm) δ = 7.84 (s, 1H), 7.37–7.28 (m, 3H), 7.26–7.23 (m, 2H), 5.19 (s, 2H), 4.33 (q, *J* = 7.2 Hz, 2H), 1.37 (t, *J* = 7.2 Hz, 3H); ^{13}C NMR (100 MHz, Chloroform-d [77.0 ppm], ppm) δ = 162.4, 141.3, 139.7, 135.5, 128.8 (2C), 128.2, 127.6 (2C), 105.0, 60.4, 52.2, 14.4; MS (EI, 70 eV, 40 °C), m/z (%): 271 (5) $[M]^+$, 198 (6), 143 (6), 91 (100), 65 (12). HRMS–EI ($C_{13}H_{13}O_2N_5$) *(m/z)*: $[M]^+$ Calcd 271.1064; Found 271.1065; IR (ATR, ṽ) = 2980 (w), 2143 (vs), 1704 (vs), 1628 (w), 1608 (w), 1547 (vs), 1487 (vs), 1455 (s), 1438 (s), 1421 (m), 1397 (m), 1378 (s), 1354 (m), 1312 (vs), 1252 (w), 1215 (vs), 1188 (vs), 1112 (m), 1081 (vs), 1071 (vs), 1030 (vs), 1003 (m), 870 (m), 837 (m), 820 (m), 773 (vs), 708 (vs), 694 (s), 647 (m), 618 (w), 586 (m), 555 (w), 541 (w), 518 (w), 489 (w), 472 (w), 456 (w), 401 (w), 385 (w) cm^{-1}.

Additional information on the chemical synthesis is available *via* Chemotion repository: https://doi.org/10.14272/reaction/SA-FUHFF-UHFFFADPSC-KCAXRYJMUF-UHFFFADPSC-NUHFF-NUHFF-NUHFF-ZZZ

Additional information on the analysis of the target compound is available *via* Chemotion repository: https://doi.org/10.14272/KCAXRYJMUFAPLD-UHFFFAOYSA-N.1

Ethyl 3-azido-1-(3,5-difluorobenzyl)-1*H*-pyrazole-4-carboxylate (**19l**)

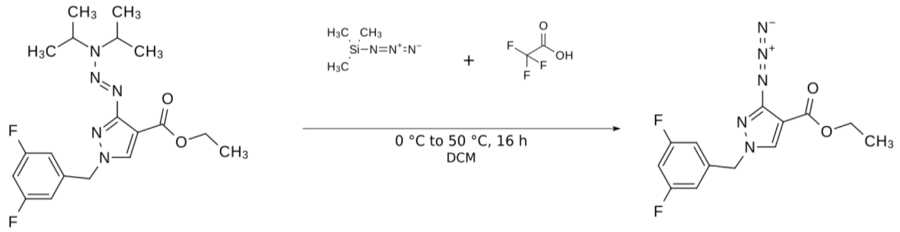

Name {P1|**19l**}: ethyl 3-azido-1-(3,5-difluorobenzyl)-1*H*-pyrazole-4-carboxylate; Formula: $C_{13}H_{11}F_2N_5O_2$; Smiles: CCOC(=O)c1cn(nc1N=[N+]=[N-])Cc1cc(F)cc(c1)F. InChIKey: BZRWHBSLSABJOL-UHFFFAOYSA-N

Ethyl (*E*)-1-(3,5-difluorobenzyl)-3-(3,3-diisopropyltriaz-1-en-1-yl)-1*H*-pyrazole-4-carboxylate (214 mg, 544 µmol, 1.00 equiv) was dissolved in methylene chloride (13.0 mL). The solution was cooled to 0 °C and then azido(trimethyl)silane (470 mg, 542 µL, 4.08 mmol, 7.50 equiv) and 2,2,2-trifluoroacetic acid (621 mg, 417 µL, 5.44 mmol, 10.00 equiv) were added. The reaction mixture was slowly warmed to 50 °C while constantly being stirred. The reaction progress was monitored *via* TLC (16 h). As preparation for the column chromatography (dry load), Celite was added (0.9 g) and the reaction mixture with Celite was evaporated. The obtained crude product was purified *via* flash-chromatography (Interchim devices puriFLASH 5.125) on silica gel (PF-15SIHP-F0025) using cyclohexane/ethyl acetate with 0% to 35% ethyl acetate in 15 column volumes (1 column volume = 63.8 mL; flow: 15 mL/min). The isolated product ethyl 3-azido-1-(3,5-difluorobenzyl)-1*H*-pyrazole-4-carboxylate (163 mg, 531 µmol) was obtained as a light yellow solid in 98% yield.

R_f = 0.52 (cyclohexane/ethyl acetate 2:1). MP = 81–83.8 °C. ^{1}H NMR (400 MHz, Chloroform-d [7.27 ppm], ppm) δ = 7.81 (s, 1H), 6.83–6.74 (m, 3H), 5.17 (s, 2H), 4.29 (q, *J* = 7.1 Hz, 2H), 1.34 (t, *J* = 7.2 Hz, 3H); ^{13}C NMR (100 MHz, Chloroform-d [77.0 ppm], ppm) δ = 163.3 (dd, *J* = 12.3 Hz, *J* = 250.5 Hz, C_q, 2C), 161.4 (C_q), 148.8 (C_q), 138.5 (t, *J* = 8.9 Hz, C_q), 135.0 (CH), 110.9 (m, CH, 2C), 105.8 (C_q), 104.1 (t, *J* = 25.4 Hz, CH), 60.5 (CH_2), 55.6 (t, *J* = 2.3 Hz, CH_2), 14.3 (CH_3); ^{19}F NMR (376 MHz, ppm) δ = -108.08; MS (EI, 70 eV, 70 °C), m/z (%): 307 (8) $[M]^+$, 179 (22), 152 (12), 127 (100). HRMS–EI ($C_{13}H_{11}O_2N_5F_2$) *(m/z)*: $[M]^+$ Calcd 307.0875; Found 307.0874; IR (ATR, ṽ) = 3135 (w), 3091 (w), 2996 (vw), 2136 (s), 1684 (vs), 1643 (w), 1621 (m), 1596 (s), 1543 (vs), 1483 (m), 1462 (vs), 1452 (s), 1441 (m), 1373 (m), 1357 (m), 1334 (s), 1317 (vs), 1283 (vs), 1237 (w), 1210 (vs), 1159 (w), 1139 (w), 1120 (vs), 1096 (vs), 1061 (w), 1016 (s), 1000 (s), 973 (w), 945 (m), 885 (m), 863 (vs), 789 (w), 776 (vs), 747 (vs), 677 (m), 646 (m), 620 (s), 603 (s), 554 (m), 531 (w), 521 (m), 510 (m), 411 (w), 404 (w) cm^{-1}.

Additional information on the chemical synthesis is available *via* Chemotion repository: https://doi.org/10.14272/reaction/SA-FUHFF-UHFFFADPSC-BZRWHBSLSA-UHFFFADPSC-NUHFF-NUHFF-NUHFF-ZZZ

Additional information on the analysis of the target compound is available *via* Chemotion repository:
https://doi.org/10.14272/BZRWHBSLSABJOL-UHFFFAOYSA-N.1

Ethyl 3-azido-1-(3-cyanobenzyl)-1*H*-pyrazole-4-carboxylate (**19m**)

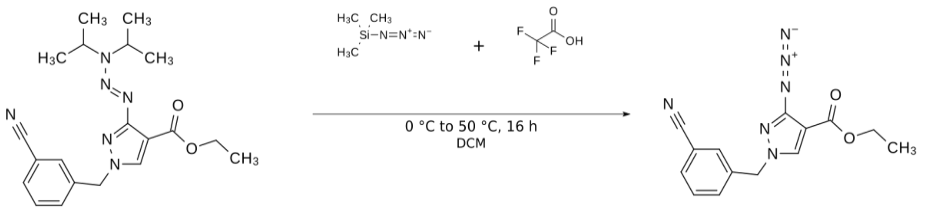

Name {P1|**19m**}: ethyl 3-azido-1-(3-cyanobenzyl)-1*H*-pyrazole-4-carboxylate; Formula: $C_{14}H_{12}N_6O_2$; Smiles: CCOC(=O)c1cn(nc1N=[N+]=[N-])Cc1cccc(c1)C#N. InChIKey: OZCFNQRRYBYULQ-UHFFFAOYSA-N

Ethyl (*E*)-1-(3-cyanobenzyl)-3-(3,3-diisopropyltriaz-1-en-1-yl)-1*H*-pyrazole-4-carboxylate (196 mg, 514 µmol, 1.00 equiv) was dissolved in methylene chloride (13.0 mL). The solution was cooled to 0 °C and then azido(trimethyl)silane (444 mg, 511 µL, 3.85 mmol, 7.50 equiv) and 2,2,2-trifluoroacetic acid (586 mg, 393 µL, 5.14 mmol, 10.00 equiv) were added. The reaction mixture was slowly warmed to 50 °C while constantly being stirred. The reaction progress was monitored *via* TLC. After full conversion (16 h), the solvent was reduced under vacuum. As preparation for column chromatography (dry load), Celite was added (1.0 g) and the reaction mixture with Celite was evaporated. The obtained crude product was purified *via* flash-chromatography (Interchim devices puriFLASH 5.125) on silica gel (PF-15SIHP-F0025) using cyclohexane/ethyl acetate with 0% to 35% ethyl acetate in 15 column volumes. The isolated product ethyl 3-azido-1-(3-cyanobenzyl)-1*H*-pyrazole-4-carboxylate (147 mg, 497 µmol) was obtained as a colourless solid in 97% yield.

R_f = 0.31 (cyclohexane/ethyl acetate 2:1). MP = 115.9–161.2 °C. ^{1}H NMR (400 MHz, Chloroform-d [7.27 ppm], ppm) δ = 7.82 (s, 1H), 7.68–7.65 (m, 1H), 7.54–7.49 (m, 3H), 5.23 (s, 2H), 4.30 (q, *J* = 7.2 Hz, 2H), 1.34 (t, *J* = 7.1 Hz, 3H); ^{13}C NMR (100 MHz, Chloroform-d [77.0 ppm], ppm) δ = 161.4 (C_q), 148.9 (C_q), 136.5 (C_q), 135.0 (CH), 132.3 (CH), 132.1 (CH), 131.2 (CH), 129.9 (CH), 118.1 (C_q), 113.3 (C_q), 105.8 (C_q), 60.6 (CH_2), 55.7 (CH_2), 14.3 (CH_3); MS (EI, 70 eV, 100 °C), m/z (%): 296 (7) $[M]^+$, 168 (20), 152 (12), 116 (100), 89 (24). HRMS–EI ($C_{14}H_{12}O_2N_6$) *(m/z)*: $[M]^+$ Calcd 296.1016; Found 296.1017; IR (ATR, ṽ) = 3123 (w), 3081 (w), 2990 (w), 2227 (w), 2119 (vs), 1694 (vs), 1555 (vs), 1477 (m), 1460 (vs), 1446 (vs), 1384 (w), 1366 (m), 1350 (w), 1323 (m), 1281 (vs), 1224 (vs), 1191 (s), 1176 (m), 1159 (w), 1094 (vs), 1060 (m), 1018 (m), 1007 (s), 973 (w), 922 (w), 892 (m), 860 (w), 790 (s), 772 (vs), 741 (m), 714 (m), 684 (vs), 643 (m), 612 (m), 595 (s), 569 (w), 552 (w), 530 (m), 443 (m), 405 (w), 387 (w) cm^{-1}.

Additional information on the chemical synthesis is available *via* Chemotion repository: https://doi.org/10.14272/reaction/SA-FUHFF-UHFFFADPSC-OZCFNQRRYB-UHFFFADPSC-NUHFF-NUHFF-NUHFF-ZZZ

Additional information on the analysis of the target compound is available *via* Chemotion repository:
https://doi.org/10.14272/OZCFNQRRYBYULQ-UHFFFAOYSA-N.1

Ethyl 3-azido-1-(4-bromobenzyl)-1*H*-pyrazole-4-carboxylate (**19n**)

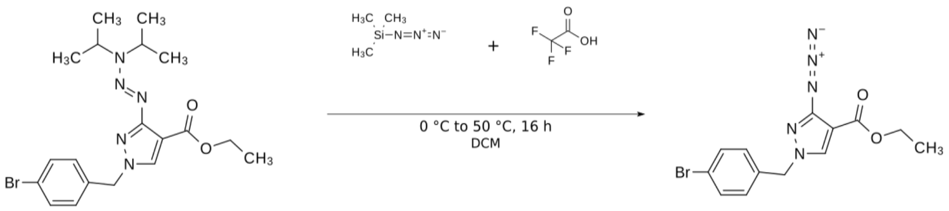

Name {P1|**19n**}: ethyl 3-azido-1-(4-bromobenzyl)-1*H*-pyrazole-4-carboxylate; Formula: $C_{13}H_{12}BrN_5O_2$; Smiles: CCOC(=O)c1cn(nc1N=[N+]=[N-])Cc1ccc(cc1)Br. InChIKey: XWYLZJSXZASHCL-UHFFFAOYSA-N

Ethyl (*E*)-1-(4-bromobenzyl)-3-(3,3-diisopropyltriaz-1-en-1-yl)-1*H*-pyrazole-4-carboxylate (288 mg, 661 µmol, 1.00 equiv) was dissolved in methylene chloride (13.0 mL). The solution was cooled to 0 °C and then azido(trimethyl)silane (571 mg, 658 µL, 4.96 mmol, 7.50 equiv) and 2,2,2-trifluoroacetic acid (754 mg, 506 µL, 6.61 mmol, 10.00 equiv) were added. The reaction mixture was slowly warmed to 50 °C while constantly being stirred. The reaction progress was monitored *via* TLC. After full conversion (16 h), the solvent was reduced under vacuum. As preparation for column chromatography (dry load), Celite was added (1.0 g) and the reaction mixture with Celite was evaporated. The obtained crude product was purified *via* flash-chromatography (Interchim devices puriFLASH 5.125) on silica gel (PF-15SIHP-F0025) using cyclohexane/ethyl acetate 0% to 30% ethyl acetate in 15 column volumes. The isolated product ethyl 3-azido-1-(4-bromobenzyl)-1*H*-pyrazole-4-carboxylate (223 mg, 637 µmol) was obtained as a colorless solid in 96% yield.

R_f = 0.53 (cyclohexane/ethyl acetate 2:1). MP = 94.7–95.2 °C. ^{1}H NMR (400 MHz, Chloroform-d [7.27 ppm], ppm) δ = 7.74 (s, 1H), 7.54–7.51 (m, 2H), 7.17–7.14 (m, 2H), 5.14 (s, 2H), 4.28 (q, *J* = 7.2 Hz, 2H), 1.33 (t, *J* = 7.1 Hz, 3H); ^{13}C NMR (100 MHz, Chloroform-d [77.0 ppm], ppm) δ = 161.5 (C_q), 148.5 (C_q), 134.7 (CH), 133.6 (C_q), 132.2 (CH, 2C), 129.7 (CH, 2C), 122.9 (C_q), 105.5 (C_q), 60.5 (CH_2), 56.0 (CH_2), 14.4 (CH_3); MS (EI, 70 eV, 110 °C), m/z (%): 349/351 (4/4) $[M]^+$, 169/171 (100/94), 90 (29). HRMS–EI ($C_{13}H_{12}O_2N_5Br$) *(m/z)*: $[M]^+$ Calcd 349.0169; Found 349.0169; IR (ATR, ṽ) = 3119 (w), 3077 (w), 2985 (w), 2962 (w), 2936 (w), 2924 (w), 2901 (w), 2868 (w), 2129 (vs), 1681 (vs), 1594 (w), 1545 (vs), 1477 (vs), 1466 (vs), 1428 (m), 1407 (w), 1392 (w), 1380 (m), 1364 (s), 1329 (m), 1316 (w), 1306 (w), 1285 (vs), 1272 (s), 1213 (vs), 1198 (vs), 1171 (s), 1113 (w), 1094 (vs), 1067 (vs), 1021 (s), 1010 (vs), 943 (m), 880 (m), 861 (w), 843 (s), 816 (m), 805 (s), 788 (s), 773 (vs), 737 (vs), 683 (m), 640 (s), 630 (w), 616 (vs), 530 (m), 517 (m), 484 (s), 418 (w), 378 (s) cm^{-1}.

Additional information on the chemical synthesis is available *via* Chemotion repository: https://doi.org/10.14272/reaction/SA-FUHFF-UHFFFADPSC-XWYLZJSXZA-UHFFFADPSC-NUHFF-NUHFF-NUHFF-ZZZ

Additional information on the analysis of the target compound is available *via* Chemotion repository:
https://doi.org/10.14272/XWYLZJSXZASHCL-UHFFFAOYSA-N.1

5-Azido-1*H*-pyrazole-4-carbonitrile (**19o**)

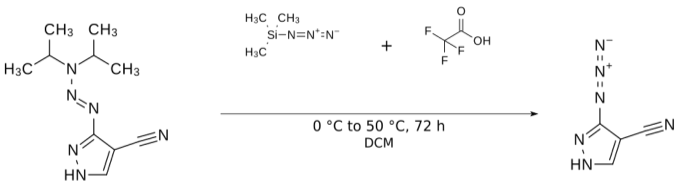

Name {P1|**19o**}: 5-azido-1H-pyrazole-4-carbonitrile; Formula: $C_4H_2N_6$; Smiles: [N-]=[N+]=Nc1n[nH]cc1C#N. InChIKey: FMLLTFUXLLAWNL-UHFFFAOYSA-N

(*E*)-3-(3,3-Diisopropyltriaz-1-en-1-yl)-1*H*-pyrazole-4-carbonitrile (100 mg, 454 µmol, 1.00 equiv) was dissolved in methylene chloride (8.0 mL). The solution was cooled to 0 °C and then azido(trimethyl)silane (392 mg, 452 µL, 3.40 mmol, 7.50 equiv) and 2,2,2-trifluoroacetic acid (518 mg, 348 µL, 4.54 mmol, 10.00 equiv) were added. The reaction mixture was slowly warmed to 50 °C while constantly being stirred. The reaction progress was monitored *via* LC-MS. After full conversion (72 h), the solvent was reduced under vacuum. As preparation for the column chromatography (dryload), Celite was added (0.3 g) and the reaction mixture with additional Celite was evaporated. The obtained crude product was purified *via* flash-chromatography (Interchim devices puriFLASH 5.125) on silica gel (PF-15SIHP-F0025) using cyclohexane/ethyl acetate 0% to 35% ethyl acetate in 25 column volumes. The isolated product 5-azido-1*H*-pyrazole-4-carbonitrile (51.7 mg, 386 µmol) was obtained as a colorless oil in 85% yield.

R_f = 0.19 (cyclohexane/ethyl acetate 1:1). ^{1}H NMR (400 MHz, DMSO-d_6 [2.50 ppm], ppm) δ = 13.66 (s, 1H), 8.55 (d, J = 1.2 Hz, 1H); ^{13}C NMR (100 MHz, DMSO-d_6 [39.5 ppm], ppm) δ = 149.3, 137.4, 112.3, 81.4; MS (EI, 70 eV, 60 °C), m/z (%): 134 (69) $[M]^+$, 131 (13), 119 (15), 106 (31), 77 (100), 69 (42), 51 (67). HRMS–EI ($C_4H_2N_6$) *(m/z)*: $[M]^+$ Calcd 134.0335; Found 134.0337; IR (ATR, ṽ) = 3274 (m), 3165 (m), 3089 (w), 2921 (w), 2495 (vw), 2422 (vw), 2356 (vw), 2281 (vw), 2242 (s), 2183 (vw), 2138 (vs), 2044 (w), 2030 (w), 1704 (vw), 1601 (vw), 1550 (w), 1500 (s), 1468 (vs), 1375 (s), 1307 (s), 1249 (vs), 1176 (s), 1108 (w), 1068 (s), 1023 (w), 979 (m), 942 (m), 933 (w), 851 (w), 824 (w), 799 (m), 769 (w), 725 (vs), 688 (vs), 613 (vs), 598 (vs), 530 (s), 506 (m), 493 (w) cm^{-1}.

Additional information on the chemical synthesis is available *via* Chemotion repository: https://doi.org/10.14272/reaction/SA-FUHFF-UHFFFADPSC-FMLLTFUXLL-UHFFFADPSC-NUHFF-NUHFF-NUHFF-ZZZ

Additional information on the analysis of the target compound is available *via* Chemotion repository:
https://doi.org/10.14272/FMLLTFUXLLAWNL-UHFFFAOYSA-N.1

3-Azido-1-propan-2-yl-pyrazole-4-carbonitrile (19p)

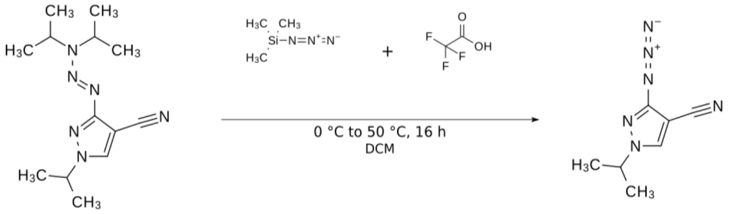

Name {P1|**19p**}: 3-azido-1-propan-2-yl-pyrazole-4-carbonitrile; Formula: $C_7H_8N_6$; Smiles: [N-]=[N+]=Nc1nn(cc1C#N)C(C)C. InChIKey: XHQDUCMTSCUCRX-UHFFFAOYSA-N

(*E*)-3-(3,3-Diisopropyltriaz-1-en-1-yl)-1-isopropyl-1*H*-pyrazole-4-carbonitrile (197 mg, 751 µmol, 1.00 equiv) was dissolved in methylene chloride (12.0 mL). The solution was cooled to 0 °C and then azido(trimethyl)silane (648 mg, 747 µL, 5.63 mmol, 7.50 equiv) and 2,2,2-trifluoroacetic acid (856 mg, 575 µL, 7.51 mmol, 10.00 equiv) were added. The reaction mixture was slowly warmed to 50 °C while constantly being stirred. The reaction progress was monitored *via* TLC. After full conversion (16 h), the solvent was reduced under vacuum. As preparation for the column chromatography (dryload), Celite was added (0.6 g) and the reaction mixture with additional Celite was evaporated. The obtained crude product was purified *via* flash-chromatography (Interchim devices puriFLASH 5.125) on silica gel (PF-15SIHP-F0025) using cyclohexane/ethyl acetate 0% to 35% ethyl acetate in 25 column volumes. The isolated product 3-azido-1-propan-2-yl-pyrazole-4-carbonitrile (115 mg, 651 µmol) was obtained as a colorless oil in 87% yield.

R_f = 0.45 (cyclohexane/ethyl acetate 2:1). ^{1}H NMR (400 MHz, Chloroform-d [7.27 ppm], ppm) δ = 7.69 (s, 1H), 4.40 (sept, *J* = 6.6 Hz, 1H), 1.51 (d, *J* = 6.8 Hz, 6H); ^{13}C NMR (100 MHz, Chloroform-d [77.0 ppm], ppm) δ = 149.8, 133.0, 111.6, 82.8, 55.5, 22.3 (2C); MS (EI, 70 eV, 20 °C), m/z (%): 176 (66) $[M]^+$, 148 (14), 106 (11), 77 (100), 63 (10), 52 (11), 51 (46). HRMS–EI ($C_7H_8N_6$) *(m/z)*: $[M]^+$ Calcd 176.0805; Found 176.0806; IR (ATR, ṽ) = 3131 (vw), 3075 (vw), 2983 (w), 2936 (vw), 2367 (vw), 2232 (s), 2130 (vs), 1543 (s), 1476 (vs), 1391 (m), 1373 (m), 1339 (w), 1244 (vs), 1180 (s), 1149 (m), 1132 (m), 1094 (w), 1075 (m), 992 (m), 890 (w), 829 (m), 693 (m), 656 (w), 639 (m), 619 (m), 530 (w), 514 (w), 503 (w), 452 (w), 418 (w), 375 (w) cm^{-1}.

Additional information on the chemical synthesis is available *via* Chemotion repository: https://doi.org/10.14272/reaction/SA-FUHFF-UHFFFADPSC-XHQDUCMTSC-UHFFFADPSC-NUHFF-NUHFF-NUHFF-ZZZ

Additional information on the analysis of the target compound is available *via* Chemotion repository:
https://doi.org/10.14272/XHQDUCMTSCUCRX-UHFFFAOYSA-N.1

Ethyl 2-(3-azido-4-cyano-1*H*-pyrazol-1-yl)acetate (19q)

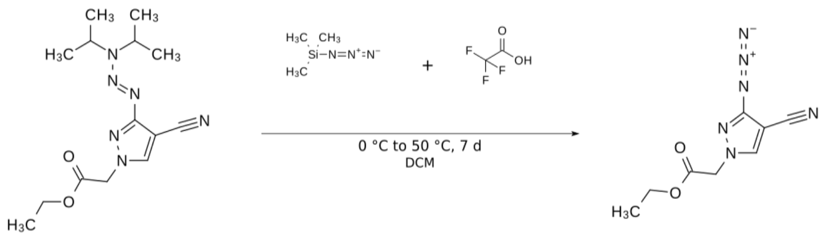

Name {P1|**19q**}: ethyl 2-(3-azido-4-cyano-1*H*-pyrazol-1-yl)acetate; Formula: $C_8H_8N_6O_2$; Smiles: CCOC(=O)Cn1nc(c(c1)C#N)N=[N+]=[N-]. InChIKey: HEIIGJFYWAHNFX-UHFFFAOYSA-N

Ethyl (*E*)-2-(4-cyano-3-(3,3-diisopropyltriaz-1-en-1-yl)-1*H*-pyrazol-1-yl)acetate (428 mg, 1.40 mmol, 1.00 equiv) was dissolved in methylene chloride (30.0 mL). The solution was cooled to 0 °C and azido(trimethyl)silane (1.21 g, 1.39 mL, 10.5 mmol, 7.50 equiv) and 2,2,2-trifluoroacetic acid (1.59 g, 1.07 mL, 14.0 mmol, 10.0 equiv) were added. The reaction mixture was slowly warmed to 50 °C and stirred for 7 days. As preparation for the column chromatography (dryload), Celite was added (1.5 g) and the reaction mixture with Celite was evaporated. The obtained crude product was purified *via* flash-chromatography (Interchim devices puriFLASH 5.125) on silica gel (PF-15SIHP-F0080) using cyclohexane/ethyl acetate 0% to 20% ethyl acetate in 15 column volumes (1 column volume = 173.2 mL; flow: 34 mL/min). The isolated product ethyl 2-(3-azido-4-cyano-1*H*-pyrazol-1-yl)acetate (268 mg, 1.22 mmol) was obtained as a colorless solid in 87% yield.

R_f = 0.32 (cyclohexane/ethyl acetate 2:1). MP = 47.9–49.8 °C. ^{1}H NMR (400 MHz, Chloroform-d [7.27 ppm], ppm) δ = 7.80 (s, 1H), 4.83 (s, 2H), 4.28 (q, *J* = 7.2 Hz, 2H), 1.32 (t, *J* = 7.2 Hz, 3H); ^{13}C NMR (100 MHz, Chloroform-d [77.0 ppm], ppm) δ = 166.1 (C_q), 150.7 (C_q), 137.4 (CH), 111.0 (C_q), 84.9 (C_q), 62.6 (CH_2), 53.7 (CH_2), 14.0 (CH_3); MS (EI, 70 eV, 50 °C), m/z (%): 220 (17) $[M]^+$, 192 (15), 105 (100), 88 (27), 77 (72), 70 (13), 64 (12), 59 (20), 51 (10). HRMS–EI ($C_8H_8O_2N_6$) *(m/z)*: $[M]^+$ Calcd 220.0703; Found 220.0704; IR (ATR, ṽ) = 3139 (vw), 3080 (vw), 2986 (w), 2367 (vw), 2235 (s), 2140 (vs), 1744 (vs), 1628 (w), 1550 (vs), 1475 (vs), 1418 (m), 1395 (m), 1375 (vs), 1354 (m), 1332 (w), 1299 (w), 1245 (vs), 1213 (vs), 1166 (vs), 1136 (s), 1096 (m), 1024 (vs), 1006 (vs), 975 (m), 938 (m), 877 (s), 847 (s), 749 (s), 691 (vs), 628 (s), 578 (m), 530 (s), 509 (m), 442 (m), 426 (m), 412 (m), 395 (s) cm^{-1}.

Additional information on the chemical synthesis is available *via* Chemotion repository: https://doi.org/10.14272/reaction/SA-FUHFF-UHFFFADPSC-HEIIGJFYWA-UHFFFADPSC-NUHFF-NUHFF-NUHFF-ZZZ

Additional information on the analysis of the target compound is available *via* Chemotion repository:
https://doi.org/10.14272/HEIIGJFYWAHNFX-UHFFFAOYSA-N.1

3-Azido-1-benzyl-1*H*-pyrazole-4-carbonitrile (19r)

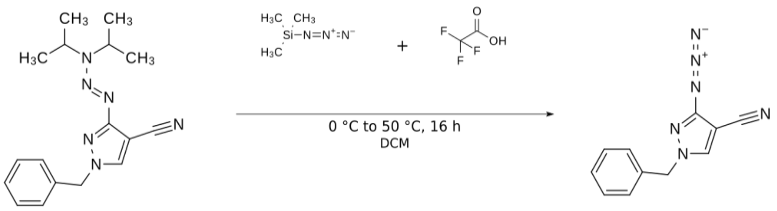

Name {P1|**19r**}: 3-azido-1-benzyl-1*H*-pyrazole-4-carbonitrile; Formula: $C_{11}H_8N_6$; Smiles: [N-]=[N+]=Nc1nn(cc1C#N)Cc1ccccc1. InChIKey: BZGHBVIXEDKDEK-UHFFFAOYSA-N

(*E*)-1-Benzyl-3-(3,3-diisopropyltriaz-1-en-1-yl)-1*H*-pyrazole-4-carbonitrile (367 mg, 1.18 mmol, 1.00 equiv) was dissolved in methylene chloride (22.4 mL). The solution was cooled to 0 °C and then azido(trimethyl)silane (1.02 g, 1.18 mL, 8.86 mmol, 7.50 equiv) and 2,2,2-trifluoroacetic acid (1.35 g, 904 µL, 11.8 mmol, 10.00 equiv) were added. The reaction mixture was slowly warmed to 50 °C while constantly being stirred. The reaction progress was monitored *via* TLC. After full conversion (16 h), the solvent was reduced under vacuum. As preparation for the column chromatography (dryload), Celite was added (1.2 g) and the reaction mixture with additional Celite was evaporated. The obtained crude product was purified *via* flash-chromatography (Interchim devices puriFLASH 4125) on silica gel (PF-15SIHP-F0040) using cyclohexane/ethyl acetate 0% to 25% ethyl acetate in 15 column volumes. The isolated product 3-azido-1-benzyl-1*H*-pyrazole-4-carbonitrile (202 mg, 899 µmol) was obtained as a colorless oil in 76% yield.

R_f = 0.43 (cyclohexane/ethyl acetate 2:1). ^{1}H NMR (400 MHz, Chloroform-d [7.27 ppm], ppm) δ = 7.58 (s, 1H), 7.43–7.36 (m, 3H), 7.30–7.24 (m, 2H), 5.20 (s, 2H); ^{13}C NMR (100 MHz, Chloroform-d [77.0 ppm], ppm) δ = 150.4, 135.5, 133.7, 129.2 (2C), 129.1, 128.3 (2C), 111.2, 83.9, 57.1; MS (EI, 70 eV, 70 °C), m/z (%): 224 (11) $[M]^+$, 91 (100), 65 (12). HRMS–EI ($C_{11}H_8N_6$) *(m/z)*: $[M]^+$ Calcd 224.0805; Found 224.0806; IR (ATR, ṽ) = 3126 (w), 3068 (w), 3036 (w), 2958 (w), 2915 (w), 2506 (vw), 2376 (vw), 2232 (s), 2143 (vs), 2139 (vs), 2099 (s), 1698 (w), 1677 (w), 1541 (s), 1476 (vs), 1456 (vs), 1439 (vs), 1375 (m), 1358 (m), 1333 (w), 1307 (w), 1278 (w), 1256 (vs), 1207 (m), 1186 (w), 1173 (w), 1160 (vs), 1122 (m), 1078 (w), 1028 (w), 1001 (w), 967 (w), 949 (w), 918 (w), 853 (m), 824 (m), 807 (w), 772 (m), 705 (vs), 688 (vs), 643 (s), 619 (s), 585 (m), 554 (m), 528 (m), 504 (m), 456 (w), 399 (s), 377 (w) cm^{-1}.

Additional information on the chemical synthesis is available *via* Chemotion repository: https://doi.org/10.14272/reaction/SA-FUHFF-UHFFFADPSC-BZGHBVIXED-UHFFFADPSC-NUHFF-NUHFF-NUHFF-ZZZ

Additional information on the analysis of the target compound is available *via* Chemotion repository:
https://doi.org/10.14272/BZGHBVIXEDKDEK-UHFFFAOYSA-N.1

3-Azido-1-(3,5-difluorobenzyl)-1*H*-pyrazole-4-carbonitrile (19s)

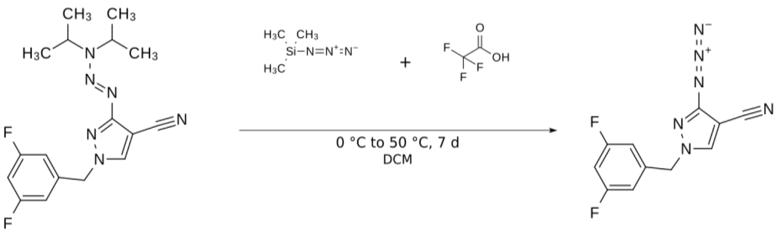

Name {P1|**19s**}: 3-azido-1-(3,5-difluorobenzyl)-1*H*-pyrazole-4-carbonitrile; Formula: $C_{11}H_6F_2N_6$; Smiles: [N-]=[N+]=Nc1nn(cc1C#N)Cc1cc(F)cc(c1)F. InChIKey: WBMURKUEYYRUPK-UHFFFAOYSA-N

(*E*)-1-(3,5-difluorobenzyl)-3-(3,3-diisopropyltriaz-1-en-1-yl)-1*H*-pyrazole-4-carbonitrile (730 mg, 2.11 mmol, 1.00 equiv) was dissolved in methylene chloride (50.0 mL). The solution was cooled to 0 °C and then azido(trimethyl)silane (1.82 g, 2.10 mL, 15.8 mmol, 7.50 equiv) and 2,2,2-trifluoroacetic acid (2.40 g, 1.61 mL, 21.1 mmol, 10.0 equiv) were added. The reaction mixture was slowly warmed to 50 °C while constantly being stirred. The reaction progress was monitored *via* LC-MS. The reaction was finished after 7 days of stirring at 50 °C. As preparation for the column chromatography (dry load), Celite was added (2.0 g) and the reaction mixture with Celite was evaporated. The obtained crude product was purified *via* flash-chromatography (Interchim devices puriFLASH 5.125) on silica gel (PF-15SIHP-F0080) using cyclohexane/ethyl acetate with 0% to 35% ethyl acetate in 12 column volumes (1 column volume = 173.2 mL; flow: 34 mL/min). The isolated product 3-azido-1-(3,5-difluorobenzyl)-1*H*-pyrazole-4-carbonitrile (490 mg, 1.88 mmol) was obtained as a colourless solid in 89% yield.

R_f = 0.51 (cyclohexane/ethyl acetate 2:1). MP = 95–95.6 °C. ^{1}H NMR (400 MHz, Chloroform-d [7.27 ppm], ppm) δ = 7.71 (s, 1H), 6.86–6.76 (m, 3H), 5.19 (s, 2H); ^{13}C NMR (100 MHz, Chloroform-d [77.0 ppm], ppm) δ = 163.3 (dd, *J* = 12.3 Hz, *J* = 250.5 Hz, C_q, 2C), 151.0 (C_q), 137.7 (t, *J* = 8.9 Hz, C_q), 135.9 (CH), 110.9 (C_q), 110.9 (dd, *J* = 7.7 Hz, *J* = 19.3 Hz, CH, 2C), 104.5 (t, *J* = 25.0 Hz, CH), 84.6 (C_q), 56.0 (t, *J* = 2.3 Hz, CH_2); ^{19}F NMR (376 MHz, ppm) δ = -107.61; MS (EI, 70 eV, 60 °C), m/z (%): 260 (10) $[M]^+$, 127 (100). HRMS–EI ($C_{11}H_6N_6F_2$) *(m/z)*: $[M]^+$ Calcd 260.0617; Found 260.0615; IR (ATR, ṽ) = 3126 (w), 3065 (w), 2360 (vw), 2232 (s), 2140 (vs), 2089 (m), 1623 (m), 1598 (vs), 1543 (vs), 1475 (vs), 1453 (vs), 1436 (s), 1375 (s), 1360 (m), 1313 (vs), 1248 (vs), 1160 (s), 1142 (w), 1115 (vs), 1006 (vs), 997 (s), 979 (w), 939 (m), 874 (m), 850 (vs), 742 (vs), 691 (s), 681 (vs), 653 (s), 620 (vs), 606 (m), 577 (s), 526 (m), 509 (vs), 494 (m), 398 (vs) cm^{-1}.

Additional information on the chemical synthesis is available *via* Chemotion repository: https://doi.org/10.14272/reaction/SA-FUHFF-UHFFFADPSC-WBMURKUEYY-UHFFFADPSC-NUHFF-NUHFF-NUHFF-ZZZ

Additional information on the analysis of the target compound is available *via* Chemotion repository: https://doi.org/10.14272/WBMURKUEYYRUPK-UHFFFAOYSA-N.1

3-Azido-1-(4-methylbenzyl)-1*H*-pyrazole-4-carbonitrile (19t)

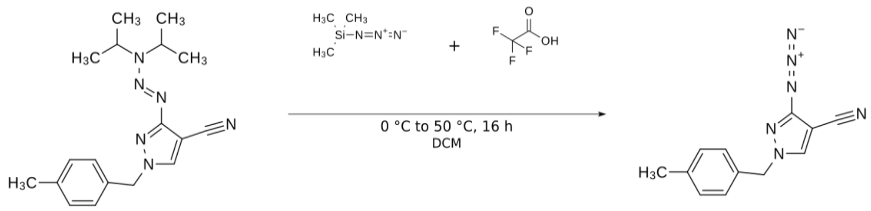

Name {P1|**19t**}: 3-azido-1-(4-methylbenzyl)-1*H*-pyrazole-4-carbonitrile; Formula: $C_{12}H_{10}N_6$; Smiles: [N-]=[N+]=Nc1nn(cc1C#N)Cc1ccc(cc1)C. InChIKey: NMQYIKIIUJYZLG-UHFFFAOYSA-N

(*E*)-3-(3,3-diisopropyltriaz-1-en-1-yl)-1-(4-methylbenzyl)-1*H*-pyrazole-4-carbonitrile (101 mg, 310 µmol, 1.00 equiv) was dissolved in methylene chloride (7.0 mL). The solution was cooled to 0 °C and then azido(trimethyl)silane (268 mg, 309 µL, 2.33 mmol, 7.50 equiv) and 2,2,2-trifluoroacetic acid (354 mg, 237 µL, 3.10 mmol, 10.00 equiv) were added. The reaction mixture was slowly warmed to 50 °C while constantly being stirred. The reaction progress was monitored *via* TLC. After full conversion (16 h), the solvent was reduced under vacuum. As preparation for column chromatography (dry load), Celite was added (0.3 g) and the reaction mixture with additional Celite was evaporated. The obtained crude product was purified *via* flash-chromatography (Interchim devices puriFLASH 5.125) on silica gel (PF-15SIHP-F0025) using cyclohexane/ethyl acetate with 0% to 25% ethyl acetate in 20 column volumes. The isolated product 3-azido-1-(4-methylbenzyl)-1*H*-pyrazole-4-carbonitrile (65.2 mg, 274 µmol) was obtained as a colorless solid in 88% yield.

R_f = 0.58 (cyclohexane/ethyl acetate 2:1). MP = 100–101.5 °C. ^{1}H NMR (400 MHz, Chloroform-d [7.27 ppm], ppm) δ = 7.54 (s, 1H), 7.20 (dd, *J* = 8.1 Hz, *J* = 18.1 Hz, 4H), 5.16 (s, 2H), 2.38 (s, 3H); ^{13}C NMR (100 MHz, Chloroform-d [77.0 ppm], ppm) δ = 150.4, 139.2, 135.3, 130.6, 129.9 (2C), 128.4 (2C), 111.3, 83.8, 56.9, 21.2; MS (EI, 70 eV, 80 °C), m/z (%): 238 (17) $[M]^+$, 105 (100). HRMS–EI ($C_{12}H_{10}N_6$) *(m/z)*: $[M]^+$ Calcd 238.0961; Found 238.0962; IR (ATR, ṽ) = 3129 (w), 3074 (w), 2948 (w), 2921 (w), 2853 (w), 2359 (w), 2231 (s), 2139 (vs), 2099 (m), 2033 (w), 1914 (w), 1687 (vw), 1615 (vw), 1545 (s), 1514 (w), 1470 (vs), 1458 (s), 1429 (s), 1377 (s), 1349 (m), 1323 (w), 1309 (w), 1244 (vs), 1214 (w), 1203 (m), 1186 (w), 1153 (s), 1115 (w), 1099 (w), 1038 (w), 1023 (w), 1000 (m), 969 (w), 939 (w), 851 (w), 839 (s), 820 (m), 812 (m), 755 (s), 727 (vs), 690 (vs), 635 (s), 623 (s), 575 (m), 528 (m), 503 (m), 469 (s), 401 (s), 375 (m) cm^{-1}.

Additional information on the chemical synthesis is available *via* Chemotion repository: https://doi.org/10.14272/reaction/SA-FUHFF-UHFFFADPSC-NMQYIKIIUJ-UHFFFADPSC-NUHFF-NUHFF-NUHFF-ZZZ

Additional information on the analysis of the target compound is available *via* Chemotion repository:
https://doi.org/10.14272/NMQYIKIIUJYZLG-UHFFFAOYSA-N.1

Methyl 4-((3-azido-4-cyano-1*H*-pyrazol-1-yl)methyl)benzoate (19u)

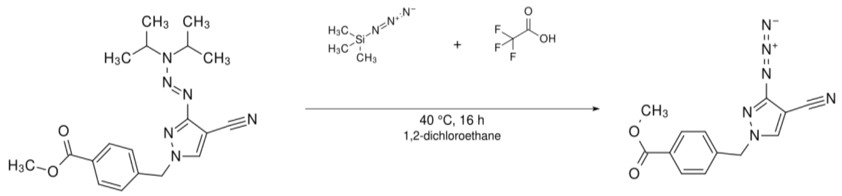

Name {P1|**19u**}: methyl 4-((3-azido-4-cyano-1*H*-pyrazol-1-yl)methyl)benzoate; Formula: $C_{13}H_{10}N_6O_2$; Smiles: [N-]=[N+]=Nc1nn(cc1C#N)Cc1ccc(cc1)C(=O)OC. InChIKey: CKRUYGWWCFZDPF-UHFFFAOYSA-N.

Methyl (E)-4-((4-cyano-3-(3,3-diisopropyltriaz-1-en-1-yl)-1*H*-pyrazol-1-yl)methyl)benzoate (51.3 mg, 139 µmol, 1.00 equiv) was dissolved in 1,2-dichloroethane (2.98 mL). The solution was cooled to 0 °C and then azido(trimethyl)silane (120 mg, 139 µL, 1.04 mmol, 7.50 equiv) and 2,2,2-trifluoroacetic acid (159 mg, 107 µL, 1.39 mmol, 10.0 equiv) were added. The reaction mixture was slowly warmed to 40 °C while constantly being stirred. The reaction progress was monitored *via* TLC. After full conversion (16 h), the solvent was reduced under vacuum. The crude product methyl 4-((3-azido-4-cyano-1H-pyrazol-1-yl)methyl)benzoate (36.0 mg, 128 µmol) was analyzed by LC-MS and obtained in 92% yield. It was used for the subsequent CuAAC reaction step to compound **21ud** without further purification.

3-Azido-1-(4-fluorobenzyl)-1*H*-pyrazole-4-carbonitrile (19v)

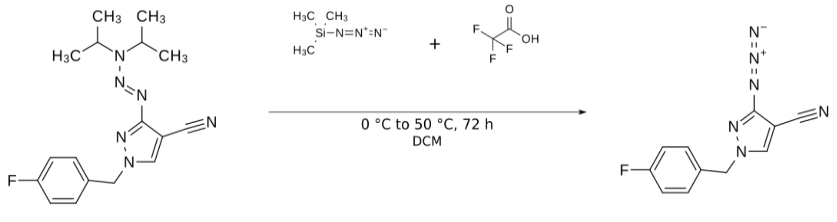

Name {P1|**19v**}: 3-azido-1-(4-fluorobenzyl)-1*H*-pyrazole-4-carbonitrile; Formula: $C_{11}H_7FN_6$; Smiles: [N-]=[N+]=Nc1nn(cc1C#N)Cc1ccc(cc1)F. InChIKey: KJPMFOGUNJZONN-UHFFFAOYSA-N

(*E*)-3-(3,3-diisopropyltriaz-1-en-1-yl)-1-(4-fluorobenzyl)-1*H*-pyrazole-4-carbonitrile (100 mg, 305 µmol, 1.00 equiv) was dissolved in methylene chloride (7.0 mL). The solution was cooled to 0 °C and then azido(trimethyl)silane (264 mg, 304 µL, 2.29 mmol, 7.50 equiv) and 2,2,2-trifluoroacetic acid (348 mg, 234 µL, 3.05 mmol, 10.00 equiv) were added. The reaction mixture was slowly warmed to 50 °C while constantly being stirred. The reaction progress was monitored *via* LC-MS. After full conversion (72 h), the solvent was reduced under vacuum. As preparation for the column chromatography (dryload), Celite was added (0.3 g) and the reaction mixture with additional Celite was evaporated. The obtained crude product was purified *via*

flash-chromatography (Interchim devices puriFLASH 5.125) on silica gel (PF-15SIHP-F0025) using cyclohexane/ethyl acetate 0% to 35% ethyl acetate in 20 column volumes. The isolated product 3-azido-1-(4-fluorobenzyl)-1*H*-pyrazole-4-carbonitrile (66.9 mg, 276 µmol) was obtained as colorless oil in 90% yield.

R_f = 0.45 (cyclohexane/ethyl acetate 2:1). ^{1}H NMR (400 MHz, Chloroform-d [7.27 ppm], ppm) δ = 7.61 (s, 1H), 7.31–7.27 (m, 2H), 7.13–7.08 (m, 2H), 5.18 (s, 2H); ^{13}C NMR (100 MHz, Chloroform-d [77.0 ppm], ppm) δ = 163.0 (d, *J* = 249 Hz), 150.6, 135.4, 130.2 (d, *J* = 8.5 Hz, 2C), 129.6 (d, *J* = 3.9 Hz), 116.2 (d, *J* = 21.6 Hz, 2C), 111.2, 84.0, 56.3; ^{19}F NMR (376 MHz, ppm) δ = -112.04; MS (EI, 70 eV, 80 °C), m/z (%): 242 (13) $[M]^+$, 109 (100), 83 (10). HRMS–EI ($C_{11}H_7N_6F$) *(m/z)*: $[M]^+$ Calcd 242.0711; Found 242.0712; IR (ATR, ṽ) = 3126 (m), 3067 (w), 2918 (w), 2849 (w), 2376 (w), 2230 (vs), 2146 (s), 2106 (s), 1892 (w), 1604 (w), 1541 (s), 1510 (s), 1476 (vs), 1438 (vs), 1419 (m), 1388 (w), 1377 (s), 1358 (s), 1324 (w), 1299 (w), 1254 (s), 1221 (vs), 1150 (vs), 1120 (s), 1095 (m), 1018 (m), 1000 (m), 948 (w), 850 (vs), 833 (vs), 824 (s), 769 (vs), 732 (vs), 686 (vs), 635 (s), 619 (vs), 577 (s), 528 (m), 518 (m), 506 (s), 482 (vs), 456 (w), 422 (s), 395 (vs) cm^{-1}.

Additional information on the chemical synthesis is available *via* Chemotion repository: https://doi.org/10.14272/reaction/SA-FUHFF-UHFFFADPSC-KJPMFOGUNJ-UHFFFADPSC-NUHFF-NUHFF-NUHFF-ZZZ

Additional information on the analysis of the target compound is available *via* Chemotion repository:
https://doi.org/10.14272/KJPMFOGUNJZONN-UHFFFAOYSA-N.1

4-Butyl-1-(1*H*-pyrazol-3-yl)-1*H*-1,2,3-triazole (21aa)

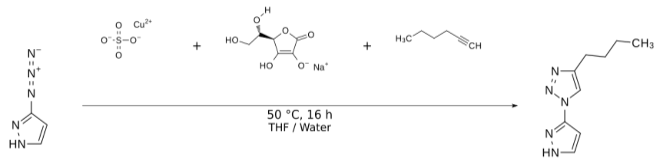

Name {P1|**21aa**}: 4-butyl-1-(1*H*-pyrazol-3-yl)-1*H*-1,2,3-triazole; Formula: $C_9H_{13}N_5$; Smiles: CCCCc1cn(nn1)c1cc[nH]n1. InChIKey: NLSVJZHXBPJQPM-UHFFFAOYSA-N

In a vial, 5-azido-1*H*-pyrazole (72.3 mg, 663 µmol, 1.00 equiv) was dissolved in THF (8.00 mL) and water (8.00 mL) (1:1) and copper sulfate (21.2 mg, 133 µmol, 0.20 equiv), sodium ascorbate (131 mg, 663 µmol, 1.00 equiv) and hex-1-yne (70.8 mg, 98.3 µL, 862 µmol, 1.30 equiv) were added. The reaction mixture was stirred at 50 °C for 16 hours. The reaction was allowed to reach room temperature, was diluted in water and extracted three times with methylene chloride. The combined organic phases where washed with water and brine, dried over Na_2SO_4 and filtered. As preparation for the column chromatography (dry load), Celite was added (0.3 g) and the reaction mixture with Celite was evaporated. The obtained crude product was purified *via* flash-chromatography (Interchim devices puriFLASH 5.125) on silica gel (PF-15SIHP-F0025) using cyclohexane/ethyl acetate 0% to 20% ethyl acetate in 15 column volumes (1 column volume = 63.8 mL; flow: 15 mL/min). The isolated product

4-butyl-1-(1*H*-pyrazol-3-yl)-1*H*-1,2,3-triazole (75.4 mg, 394 μmol) was obtained as a colorless solid in 59% yield.

R_f = 0.35 (cyclohexane/ethyl acetate 1:1). ^{1}H NMR (400 MHz, Chloroform-d [7.27 ppm], ppm) δ = 7.95 (s, 1H), 7.73 (d, *J* = 2.4 Hz, 1H), 6.76 (d, *J* = 2.4 Hz, 1H), 2.82 (t, *J* = 7.6 Hz, 2H), 1.77–1.69 (m, 2H), 1.43 (h, *J* = 7.3 Hz, 2H), 0.96 (t, *J* = 7.4 Hz, 3H) Missing Signal (1H, NH); ^{13}C NMR (100 MHz, Chloroform-d [77.0 ppm], ppm) δ = 148.5 (C_q), 147.2 (C_q), 130.7 (CH), 118.8 (CH), 96.9 (CH), 31.4 (CH_2), 25.2 (CH_2), 22.2 (CH_2), 13.8 (CH_3); MS (EI, 70 eV, 70 °C), m/z (%): 191 (2) $[M]^+$, 162 (13), 148 (14), 120 (100), 94 (12). HRMS–EI ($C_9H_{13}N_5$) *(m/z)*: $[M]^+$ Calcd 191.1165; Found 191.1166; IR (ATR, ṽ) = 3126 (m), 3115 (m), 3075 (w), 2986 (w), 2951 (s), 2919 (s), 2857 (s), 2806 (m), 2747 (w), 2691 (w), 1557 (m), 1534 (vs), 1489 (w), 1463 (w), 1453 (w), 1441 (w), 1378 (m), 1366 (w), 1312 (w), 1231 (vs), 1204 (w), 1193 (w), 1187 (w), 1103 (m), 1082 (w), 1055 (vs), 1038 (vs), 1020 (w), 939 (s), 919 (m), 849 (s), 817 (m), 766 (vs), 754 (vs), 713 (w), 691 (w), 652 (w), 618 (w), 514 (w), 490 (w), 405 (w), 401 (w), 378 (w) cm^{-1}.

Additional information on the chemical synthesis is available *via* Chemotion repository: https://doi.org/10.14272/reaction/SA-FUHFF-UHFFFADPSC-NLSVJZHXBP-UHFFFADPSC-NUHFF-NUHFF-NUHFF-ZZZ

Additional information on the analysis of the target compound is available *via* Chemotion repository:
https://doi.org/10.14272/NLSVJZHXBPJQPM-UHFFFAOYSA-N.1

Methyl 1-(1*H*-pyrazol-5-yl)triazole-4-carboxylate (21ab)

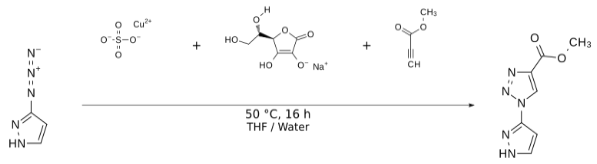

Name {P1|**21ab**}: methyl 1-(1H-pyrazol-5-yl)triazole-4-carboxylate; Formula: $C_7H_7N_5O_2$; Smiles: COC(=O)c1nnn(c1)c1n[nH]cc1. InChIKey: PWKBPRHBEDYORH-UHFFFAOYSA-N

In a vial, 5-azido-1*H*-pyrazole (83.0 mg, 761 μmol, 1.00 equiv) was dissolved in THF (8.00 mL) and water (8.00 mL) (1:1) and copper;sulfate (24.3 mg, 152 μmol, 0.20 equiv), sodium ascorbate (151 mg, 761 μmol, 1.00 equiv) and methyl prop-2-ynoate (83.2 mg, 88.0 μL, 989 μmol, 1.30 equiv) were added. The reaction mixture was stirred at 50 °C for 16 hours. For work, up the reaction mixture was diluted in water and extracted three times with methylene chloride. The combined organic phase was washed with water and brine, dried over Na_2SO_4 and filtered. As preparation for the column chromatography (dry load), Celite was added (0.3 g) and the reaction mixture with Celite was evaporated. The obtained crude product was purified *via* flash-chromatography (Interchim devices puriFLASH 5.125) on silica gel (PF-15SIHP-F0025) using cyclohexane/ethyl acetate with 0% to 100% ethyl acetate in 20 column volumes (1 column volume = 63.8 mL; flow: 15 mL/min). The isolated product methyl 1-(1*H*-pyrazol-5-yl)triazole-4-carboxylate (111 mg, 576 μmol) was obtained as a colorless solid in 76% yield.

R_f = 0.18 (cyclohexane/ethyl acetate 1:1). MP = 226–229.7 °C. ^{1}H NMR (400 MHz, DMSO-d_6 [2.50 ppm], ppm) δ = 13.36 (s, 1H), 9.16 (s, 1H), 7.98 (dd, J = 1.7 Hz, J = 2.3 Hz, 1H), 6.78 (t, J = 2.2 Hz, 1H), 3.87 (s, 3H); ^{13}C NMR (100 MHz, DMSO-d_6 [39.5 ppm], ppm) δ = 160.5 (C_q), 145.2 (C_q), 139.0 (C_q), 131.5 (CH), 126.7 (CH), 97.1 (CH), 52.0 (CH_3); MS (EI, 70 eV, 120 °C), m/z (%): 193 (24) [M]$^+$, 164 (19), 134 (100), 122 (29), 106 (53), 94 (16), 81 (12), 78 (10), 52 (27). HRMS–EI ($C_7H_7O_2N_5$) *(m/z)*: [M]$^+$ Calcd 193.0594; Found 193.0596; IR (ATR, ṽ) = 3162 (m), 3153 (m), 3132 (m), 2985 (w), 2966 (w), 2921 (m), 2851 (w), 1724 (vs), 1547 (s), 1538 (s), 1475 (w), 1436 (s), 1397 (s), 1387 (m), 1377 (m), 1354 (s), 1275 (w), 1259 (vs), 1230 (w), 1205 (vs), 1160 (s), 1099 (m), 1071 (s), 1047 (vs), 1040 (vs), 1013 (s), 946 (w), 935 (s), 924 (s), 878 (s), 839 (vs), 824 (vs), 807 (vs), 769 (vs), 693 (s), 613 (s), 554 (m), 473 (m), 439 (s), 416 (w), 388 (w) cm^{-1}.

Additional information on the chemical synthesis is available *via* Chemotion repository: https://doi.org/10.14272/reaction/SA-FUHFF-UHFFFADPSC-PWKBPRHBED-UHFFFADPSC-NUHFF-NUHFF-NUHFF-ZZZ

Additional information on the analysis of the target compound is available *via* Chemotion repository:
https://doi.org/10.14272/PWKBPRHBEDYORH-UHFFFAOYSA-N.1

This compound has been previously synthesized and the analytical data reported herein are consistent with the literature. [9]

4-(4-Methoxyphenyl)-1-(1*H*-pyrazol-3-yl)-1*H*-1,2,3-triazole (**21ae**)

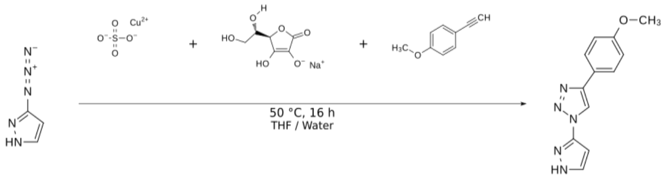

Name {P1|**21ae**}: 4-(4-methoxyphenyl)-1-(1*H*-pyrazol-3-yl)-1*H*-1,2,3-triazole; Formula: $C_{12}H_{11}N_5O$; Smiles: COc1ccc(cc1)c1nnn(c1)c1n[nH]cc1. InChIKey: PQOYNAQPLQYWLU-UHFFFAOYSA-N

In a vial, 5-azido-1*H*-pyrazole (50.0 mg, 458 µmol, 1.00 equiv) was dissolved in THF (6.00 mL) and water (6.00 mL) (1:1) and copper;sulfate (14.6 mg, 91.7 µmol, 0.20 equiv), sodium ascorbate (90.8 mg, 458 µmol, 1.00 equiv) and 1-ethynyl-4-methoxybenzene (78.7 mg, 77.3 µL, 596 µmol, 1.30 equiv) were added. The reaction mixture was stirred at 50 °C for 16 hours. For work, up the reaction mixture was diluted in water and extracted three times with methylene chloride. The combined organic phases were washed with water and brine, dried over Na_2SO_4 and filtered. As preparation for the column chromatography (dry load), Celite was added (0.2 g) and the reaction mixture with Celite was evaporated. The obtained crude product was purified *via* flash-chromatography (Interchim devices puriFLASH 5.125) on silica gel (PF-15SIHP-F0012) using cyclohexane/ethyl acetate with 0% to 50% ethyl acetate in 15 column volumes (1 column volume = 39.3 mL; flow: 15 mL/min). The isolated product 4-(4-methoxyphenyl)-1-(1*H*-pyrazol-3-yl)-1*H*-1,2,3-triazole (66.1 mg, 274 µmol) was obtained as a colourless solid in 60% yield.

R_f = 0.29 (cyclohexane/ethyl acetate 1:1). MP = 205.4–246.9 °C. ^{1}H NMR (400 MHz, DMSO-d_6 [2.50 ppm], ppm) δ = 13.27 (s, 1H), 8.98 (s, 1H), 7.97 (dd, J = 1.7 Hz, J = 2.2 Hz, 1H), 7.93–7.89 (m, 2H), 7.05–7.02 (m, 2H), 6.73 (t, J = 2.1 Hz, 1H), 3.80 (s, 3H); ^{13}C NMR (100 MHz, DMSO-d_6 [39.5 ppm], ppm) δ = 159.3 (C_q), 146.6 (C_q), 146.1 (C_q), 131.3 (CH), 126.8 (CH, 2C), 122.7 (C_q), 118.2(CH), 114.3 (CH, 2C), 96.4 (CH), 55.2 (CH_3); MS (EI, 70 eV, 160 °C), m/z (%): 241 (21) $[M]^+$, 213 (100), 198 (50), 186 (29), 170 (14), 132 (16), 89 (15). HRMS–EI ($C_{12}H_{11}ON_5$) *(m/z)*: $[M]^+$ Calcd 241.0958; Found 241.0960; IR (ATR, ṽ) = 3281 (w), 3139 (m), 3118 (m), 3071 (w), 3029 (w), 2993 (w), 2970 (w), 2929 (m), 2901 (w), 2876 (w), 2837 (w), 1618 (m), 1561 (vs), 1541 (w), 1493 (vs), 1487 (vs), 1462 (m), 1453 (m), 1443 (m), 1426 (w), 1407 (w), 1357 (w), 1339 (w), 1307 (m), 1292 (w), 1249 (vs), 1232 (vs), 1174 (vs), 1123 (w), 1109 (w), 1065 (s), 1054 (m), 1030 (vs), 982 (m), 976 (w), 938 (m), 918 (m), 894 (w), 858 (w), 819 (vs), 795 (vs), 764 (vs), 751 (vs), 727 (s), 704 (m), 693 (w), 656 (w), 633 (w), 620 (vs), 613 (vs), 526 (s), 511 (m), 477 (w), 446 (w), 429 (w), 408 (w) cm^{-1}.

Additional information on the chemical synthesis is available *via* Chemotion repository: https://doi.org/10.14272/reaction/SA-FUHFF-UHFFFADPSC-PQOYNAQPLQ-UHFFFADPSC-NUHFF-NUHFF-NUHFF-ZZZ

Additional information on the analysis of the target compound is available *via* Chemotion repository:
https://doi.org/10.14272/PQOYNAQPLQYWLU-UHFFFAOYSA-N.1

Methyl 1-(1-cyclopentyl-1*H*-pyrazol-3-yl)-1*H*-1,2,3-triazole-4-carboxylate (21bb)

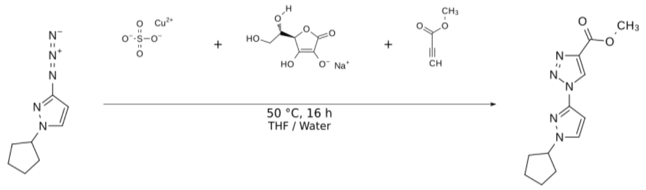

Name {P1|**21bb**}: methyl 1-(1-cyclopentyl-1*H*-pyrazol-3-yl)-1*H*-1,2,3-triazole-4-carboxylate; Formula: $C_{12}H_{15}N_5O_2$; Smiles: COC(=O)c1nnn(c1)c1ccn(n1)C1CCCC1. InChIKey: RZTMDUAYRSTLAQ-UHFFFAOYSA-N

In a vial, 3-azido-1-cyclopentyl-pyrazole (50.0 mg, 282 μmol, 1.00 equiv) was dissolved in THF (8.00 mL) and water (8.00 mL) (1:1) and copper;sulfate (9.01 mg, 56.4 μmol, 0.20 equiv), sodium ascorbate (55.9 mg, 282 μmol, 1.00 equiv) and methyl prop-2-ynoate (30.8 mg, 32.6 μL, 367 μmol, 1.30 equiv) were added. The reaction mixture was stirred at 50 °C for 16 hours. For work up, the reaction mixture was diluted in water and extracted three times with methylene chloride. The combined organic phases where washed with water and brine, dried over Na_2SO_4 and filtered. As preparation for the column chromatography (dry load), Celite was added (0.2 g) and the reaction mixture with Celite was evaporated. The obtained crude product was purified *via* flash-chromatography (Interchim devices puriFLASH 5.125) on silica gel (PF-15SIHP-F0012) using cyclohexane/ethyl acetate with 0% to 35% ethyl acetate in 20 column volumes (1 column volume = 39.3 mL; flow: 15 mL/min). The isolated product methyl 1-(1-cyclopentyl-1*H*-pyrazol-3-yl)-1*H*-1,2,3-triazole-4-carboxylate (69.8 mg, 267 μmol) was obtained as a yellow oil in 95% yield.

R_f = 0.61 (cyclohexane/ethyl acetate 1:1). ^{1}H NMR (400 MHz, Chloroform-d [7.27 ppm], ppm) δ = 8.70 (s, 1H), 7.49 (d, *J* = 2.4 Hz, 1H), 6.72 (d, *J* = 2.3 Hz, 1H), 4.69–4.62 (m, 1H), 3.99 (s, 3H), 2.23–2.14 (m, 2H), 2.09–2.00 (m, 2H), 1.95–1.85 (m, 2H), 1.79–1.68 (m, 2H). ^{13}C NMR (100 MHz, Chloroform-d [77.0 ppm], ppm) δ = 161.1 (C_q), 145.0 (C_q), 139.7 (C_q), 130.0 (CH) , 125.0 (CH), 97.1 (CH), 63.7 (CH), 52.2 (CH_3), 33.0 (CH_2, 2C), 24.1 (CH_2, 2C); MS (EI, 70 eV, 80 °C), m/z (%): 261 (42) $[M]^+$, 202 (21), 165 (100), 160 (13), 133 (68), 122 (33), 107 (72), 94 (17), 84 (25), 79 (15), 69 (31), 52 (27). HRMS–EI ($C_{12}H_{15}O_2N_5$) *(m/z)*: $[M]^+$ Calcd 261.1220; Found 261.1222; IR (ATR, ṽ) = 3128 (w), 3118 (w), 3078 (w), 2952 (m), 2927 (m), 2868 (m), 2809 (w), 2747 (w), 2690 (w), 1735 (m), 1727 (m), 1537 (vs), 1487 (w), 1463 (w), 1453 (w), 1436 (m), 1380 (m), 1363 (w), 1322 (m), 1313 (m), 1249 (w), 1231 (vs), 1204 (s), 1194 (s), 1156 (m), 1103 (m), 1082 (w), 1037 (vs), 938 (s), 919 (m), 899 (w), 841 (m), 806 (m), 766 (vs), 754 (vs), 713 (w), 693 (w), 646 (w), 618 (w), 551 (w), 541 (w), 531 (w), 507 (w), 490 (w), 445 (w), 402 (w), 378 (w) cm^{-1}.

Additional information on the chemical synthesis is available *via* Chemotion repository: https://doi.org/10.14272/reaction/SA-FUHFF-UHFFFADPSC-RZTMDUAYRS-UHFFFADPSC-NUHFF-NUHFF-NUHFF-ZZZ

Additional information on the analysis of the target compound is available *via* Chemotion repository:
https://doi.org/10.14272/RZTMDUAYRSTLAQ-UHFFFAOYSA-N.1

1-(1-Cyclopentyl-1*H*-pyrazol-3-yl)-4-(4-methoxyphenyl)-1*H*-1,2,3-triazole (**21be**)

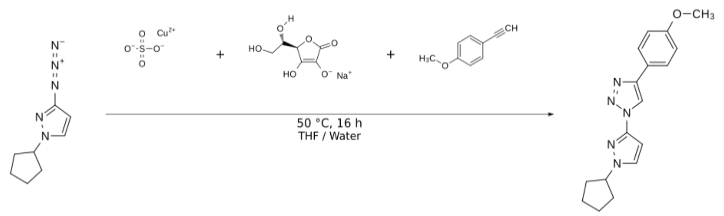

Name {P1|**21be**}: 1-(1-cyclopentyl-1*H*-pyrazol-3-yl)-4-(4-methoxyphenyl)-1*H*-1,2,3-triazole; Formula: $C_{17}H_{19}N_5O$; Smiles: COc1ccc(cc1)c1nnn(c1)c1ccn(n1)C1CCCC1. InChIKey: SYHGOJNCDVCRGL-UHFFFAOYSA-N

In a vial, 3-azido-1-cyclopentyl-pyrazole (50.0 mg, 282 µmol, 1.00 equiv) was dissolved in THF (8.00 mL) and water (8.00 mL) (1:1) and copper;sulfate (9.01 mg, 56.4 µmol, 0.20 equiv), sodium ascorbate (55.9 mg, 282 µmol, 1.00 equiv) and 1-ethynyl-4-methoxybenzene (48.5 mg, 47.6 µL, 367 µmol, 1.30 equiv) were added. The reaction mixture was stirred at 50 °C for 16 hours. For work up the reaction mixture was diluted in water and extracted three times with methylene chloride. The combined organic phase was washed with water and brine, dried over Na_2SO_4 and filtered. As preparation for the column chromatography (dryload), Celite was added (0.2 g) and the reaction mixture with Celite were evaporated. The obtained crude product was purified *via* flash-chromatography (Interchim devices puriFLASH 5.125) on silica gel (PF-15SIHP-F0012) using cyclohexane/ethyl acetate 0% to 25% ethyl acetate in 20 column volumes (1 column volume = 39.3 mL; flow: 15 mL/min). The isolated product 1-(1-cyclopentyl-1*H*-pyrazol-3-yl)-4-(4-methoxyphenyl)-1*H*-1,2,3-triazole (84.6 mg, 273 µmol) was obtained as a yellow oil in 97% yield.

R_f = 0.65 (cyclohexane/ethyl acetate 1:1). ^{1}H NMR (400 MHz, Chloroform-d [7.27 ppm], ppm) δ = 8.32 (s, 1H), 7.87–7.83 (m, 2H), 7.48 (d, *J* = 2.3 Hz, 1H), 7.00–6.97 (m, 2H), 6.71 (d, *J* = 2.4 Hz, 1H), 4.65 (quint, *J* = 7.0 Hz, 1H), 3.85 (s, 3H), 2.24–2.15 (m, 2H), 2.11–2.02 (m, 2H), 1.95–1.86 (m, 2H), 1.79–1.71 (m, 2H); ^{13}C NMR (100 MHz, Chloroform-d [77.0 ppm], ppm) δ = 159.7, 147.4, 145.9, 129.6, 127.2 (2C), 123.1, 116.4, 114.2 (2C), 96.7, 63.6, 55.3, 33.0 (2C), 24.0 (2C); MS (EI, 70 eV, 130 °C), m/z (%): 309 (18) $[M]^+$, 281 (29), 274 (14), 266 (26), 259 (12), 252 (100), 231 (88), 225 (11), 220 (10), 213 (91), 203 (38), 198 (59), 187 (10), 170 (10), 159 (13), 146 (10), 132 (16), 121 (11), 105 (14), 89 (21), 81 (12), 77 (14), 69 (18), 58 (17). HRMS–EI ($C_{17}H_{19}ON_5$) *(m/z)*: $[M]^+$ Calcd 309.1584; Found 309.1583; IR (ATR, ṽ) = 3159 (w), 3115 (w), 2999 (w), 2962 (w), 2948 (w), 2915 (w), 2871 (w), 2840 (w), 1754 (vw), 1621 (w), 1567 (s), 1547 (s), 1520 (w), 1496 (vs), 1476 (m), 1462 (m), 1452 (m), 1439 (m), 1425 (m), 1400 (w), 1385 (w), 1309 (w), 1289 (s), 1247 (vs), 1228 (vs), 1201 (w), 1171 (s), 1153 (w), 1126 (w), 1108 (w), 1091 (w), 1065 (w), 1047 (s), 1028 (vs), 997 (m), 972 (m), 950 (w), 936 (m), 909 (w), 899 (w), 884 (w), 853 (w), 826 (vs), 805 (w), 785 (vs), 771 (s), 759 (vs), 701 (w), 691 (w), 650 (w), 637 (m), 619 (s), 544 (w), 523 (vs), 507 (m), 449 (w), 421 (w), 399 (w), 385 (w) cm^{-1}.

Additional information on the chemical synthesis is available *via* Chemotion repository: https://doi.org/10.14272/reaction/SA-FUHFF-UHFFFADPSC-SYHGOJNCDV-UHFFFADPSC-NUHFF-NUHFF-NUHFF-ZZZ

Additional information on the analysis of the target compound is available *via* Chemotion repository:
https://doi.org/10.14272/SYHGOJNCDVCRGL-UHFFFAOYSA-N.1

1-(1-Benzyl-1*H*-pyrazol-3-yl)-4-(4-methoxyphenyl)-1*H*-1,2,3-triazole (21ce)

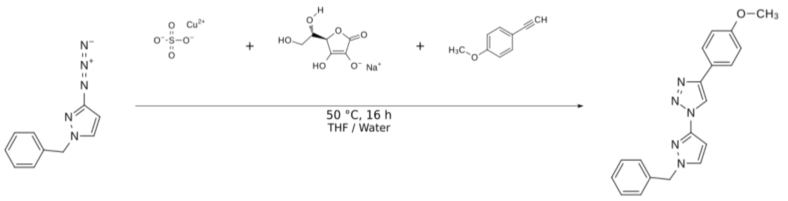

Name {P1|**21ce**}: 1-(1-benzyl-1*H*-pyrazol-3-yl)-4-(4-methoxyphenyl)-1*H*-1,2,3-triazole; Formula: $C_{19}H_{17}N_5O$; Smiles: COc1ccc(cc1)c1nnn(c1)c1ccn(n1)Cc1ccccc1. InChIKey: JMPSOWDQCFVJPL-UHFFFAOYSA-N

In a vial, 3-azido-1-benzyl-pyrazole (158 mg, 794 µmol, 1.00 equiv) was dissolved in THF (10.00 mL) and water (10.00 mL) (1:1) and copper;sulfate (25.3 mg, 159 µmol, 0.200 equiv), sodium ascorbate (157 mg, 794 µmol, 1.00 equiv) and 1-ethynyl-4-methoxybenzene (136 mg, 134 µL, 1.03 mmol, 1.30 equiv) were added. The reaction mixture was stirred at 50 °C for 16 hours. Then it was diluted with water and extracted three times with methylene chloride. The combined organic phase was washed with water and brine, dried over Na_2SO_4 and filtered. As preparation for the column chromatography (dryload), Celite was added (0.5 g) and the reaction mixture with Celite was evaporated. The obtained crude product was purified *via* flash-chromatography (Interchim devices puriFLASH 5.125) on silica gel (PF-15SIHP-F0025) using cyclohexane/ethyl acetate 0% to 33% ethyl acetate in 15 column volumes (1 column volume = 63.8 mL; flow: 15 mL/min). The isolated product 1-(1-

benzyl-1*H*-pyrazol-3-yl)-4-(4-methoxyphenyl)-1*H*-1,2,3-triazole (211 mg, 636 µmol) was obtained as a colorless solid in 80% yield.

R_f = 0.30 (cyclohexane/ethyl acetate 2:1). MP = 131.7–132.8 °C. ^{1}H NMR (400 MHz, Chloroform-d [7.27 ppm], ppm) δ = 8.33 (s, 1H), 7.86–7.82 (m, 2H), 7.44 (d, *J* = 2.4 Hz, 1H), 7.41–7.35 (m, 3H), 7.29–7.27 (m, 2H), 7.01–6.97 (m, 2H), 6.78 (d, *J* = 2.4 Hz, 1H), 5.33 (s, 2H), 3.85 (s, 3H); ^{13}C NMR (100 MHz, Chloroform-d [77.0 ppm], ppm) δ = 159.7 (C_q), 147.5 (C_q), 146.5 (C_q), 135.5 (C_q), 131.5 (CH), 128.9 (CH, 2C), 128.4 (CH), 127.7 (CH, 2C), 127.1 (CH, 2C), 123.0 (C_q), 116.3 (CH), 114.2 (CH, 2C), 97.7 (CH), 56.4 (CH_2), 55.3 (CH_3); MS (FAB, 3-NBA), m/z (%): 332 (100) $[M+1]^+$, 303 (14), 212 (19), 154 (14), 136 (10), 91 (26). HRMS–FAB ($C_{19}H_{18}ON_5$) *(m/z)*: $[M+H]^+$ Calcd 332.1506; Found 332,1505; IR (ATR, ṽ) = 3112 (w), 2958 (w), 2935 (w), 1616 (w), 1562 (m), 1538 (m), 1520 (w), 1490 (vs), 1456 (m), 1432 (w), 1421 (w), 1349 (w), 1333 (w), 1302 (w), 1282 (w), 1247 (vs), 1227 (s), 1220 (s), 1205 (w), 1194 (m), 1171 (s), 1122 (w), 1108 (w), 1048 (m), 1027 (vs), 997 (w), 973 (w), 936 (m), 924 (w), 836 (s), 822 (w), 803 (w), 788 (vs), 758 (vs), 710 (vs), 684 (m), 652 (w), 633 (m), 618 (s), 572 (w), 528 (m), 494 (w), 466 (w), 429 (w), 394 (w), 375 (w) cm^{-1}.

Additional information on the chemical synthesis is available *via* Chemotion repository: https://doi.org/10.14272/reaction/SA-FUHFF-UHFFFADPSC-JMPSOWDQCF-UHFFFADPSC-NUHFF-NUHFF-NUHFF-ZZZ

Additional information on the analysis of the target compound is available *via* Chemotion repository:
https://doi.org/10.14272/JMPSOWDQCFVJPL-UHFFFAOYSA-N.1

Methyl 1-(1-(4-bromobenzyl)-1*H*-pyrazol-3-yl)-1*H*-1,2,3-triazole-4-carboxylate (21db)

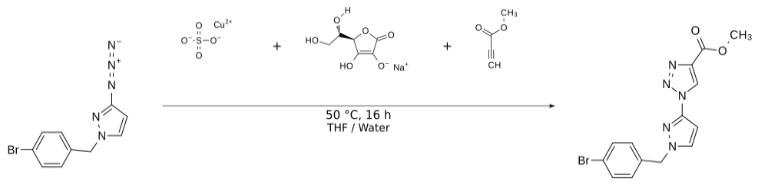

Name {P1|**21db**}: methyl 1-(1-(4-bromobenzyl)-1*H*-pyrazol-3-yl)-1*H*-1,2,3-triazole-4-carboxylate; Formula: $C_{14}H_{12}BrN_5O_2$; Smiles: COC(=O)c1nnn(c1)c1ccn(n1)Cc1ccc(cc1)Br. InChIKey: CNHRLRYEDLASIW-UHFFFAOYSA-N

In a vial, 3-azido-1-(4-bromobenzyl)pyrazole (50.0 mg, 180 µmol, 1.00 equiv) was dissolved in THF (8.00 mL) and water (8.00 mL) (1:1) and copper;sulfate (5.74 mg, 36.0 µmol, 0.20 equiv), sodium ascorbate (35.6 mg, 180 µmol, 1.00 equiv) and methyl prop-2-ynoate (19.6 mg, 20.8 µL, 234 µmol, 1.30 equiv) were added. The reaction mixture was stirred at 50 °C for 16 hours. For work up the reaction mixture was diluted in water and extracted three times with methylene chloride. The combined organic phase was washed with water and brine, dried over Na_2SO_4 and filtered. As preparation for the column chromatography (dryload), Celite was added (0.5 g) and the reaction mixture with Celite were evaporated. The obtained crude product was purified *via* flash-chromatography (Interchim devices puriFLASH 5.125) on silica gel

(PF-15SIHP-F0012) using cyclohexane/ethyl acetate 0% to 50% ethyl acetate in 15 column volumes (1 column volume = 39.3 mL; flow: 15 mL/min). The isolated product methyl 1-(1-(4-bromobenzyl)-1*H*-pyrazol-3-yl)-1*H*-1,2,3-triazole-4-carboxylate (64.0 mg, 177 µmol) was obtained as a colorless solid in 98% yield.

R_f = 0.45 (cyclohexane/ethyl acetate 1:1). MP = 140.1–141.2 °C. ^{1}H NMR (400 MHz, Chloroform-d [7.27 ppm], ppm) δ = 8.66 (s, 1H), 7.49–7.46 (m, 3H), 7.13 (d, *J* = 8.4 Hz, 2H), 6.75 (d, *J* = 2.4 Hz, 1H), 5.26 (s, 2H), 3.95 (s, 3H); ^{13}C NMR (100 MHz, Chloroform-d [77.0 ppm], ppm) δ = 160.8, 145.7, 139.7, 134.2, 132.0 (2C), 131.8, 129.4 (2C), 124.9, 122.5, 98.2, 55.8, 52.2; MS (FAB, 3-NBA), m/z (%): 362/364 (100/97) $[M+1]^+$, 307 (14), 304 (10), 289 (10), 219 (23), 169/171 (37/36), 154 (87), 147/149 (12/11), 136 (72), 129/131 (10/11), 121/119 (14/15), 115 (13), 107 (30), 95 (22), 91 (35), 89 (24). HRMS–FAB ($C_{14}H_{13}O_2N_5Br$) *(m/z)*: $[M+H]^+$ Calcd 362.0247; Found 362.0245; IR (ATR, ṽ) = 3146 (w), 3122 (w), 3034 (w), 2955 (w), 2924 (w), 2849 (w), 1897 (vw), 1731 (vs), 1689 (w), 1592 (w), 1554 (vs), 1544 (s), 1487 (m), 1458 (w), 1432 (s), 1392 (m), 1374 (w), 1353 (w), 1333 (w), 1312 (vs), 1298 (s), 1258 (s), 1231 (vs), 1194 (vs), 1156 (vs), 1108 (w), 1095 (w), 1072 (s), 1044 (m), 1033 (vs), 1014 (vs), 993 (m), 935 (s), 880 (w), 850 (w), 843 (m), 820 (w), 806 (vs), 771 (s), 755 (vs), 748 (vs), 693 (m), 684 (w), 645 (w), 632 (w), 611 (w), 534 (w), 500 (s), 484 (w), 460 (w), 392 (w) cm^{-1}.

Additional information on the chemical synthesis is available *via* Chemotion repository: https://doi.org/10.14272/reaction/SA-FUHFF-UHFFFADPSC-CNHRLRYEDL-UHFFFADPSC-NUHFF-NUHFF-NUHFF-ZZZ

Additional information on the analysis of the target compound is available *via* Chemotion repository:
https://doi.org/10.14272/CNHRLRYEDLASIW-UHFFFAOYSA-N.1

1-(1-(4-Bromobenzyl)-1*H*-pyrazol-3-yl)-4-(4-methoxyphenyl)-1*H*-1,2,3-triazole (21de)

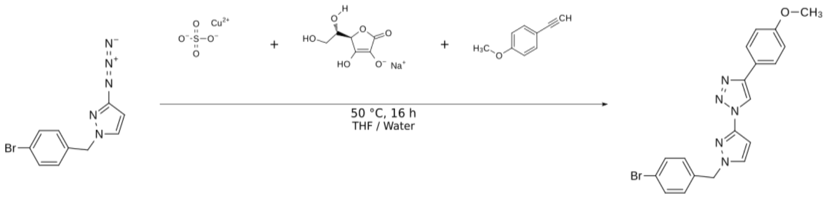

Name {P1|**21de**}: 1-(1-(4-bromobenzyl)-1*H*-pyrazol-3-yl)-4-(4-methoxyphenyl)-1*H*-1,2,3-triazole; Formula: $C_{19}H_{16}BrN_5O$; Smiles: COc1ccc(cc1)c1nnn(c1)c1ccn(n1)Cc1ccc(cc1)Br. InChIKey: QXNOXDRDOZJZFO-UHFFFAOYSA-N

In a vial, 3-azido-1-(4-bromobenzyl)pyrazole (50.0 mg, 180 µmol, 1.00 equiv) was dissolved in THF (8.00 mL) and water (8.00 mL) (1:1) and copper;sulfate (5.74 mg, 36.0 µmol, 0.200 equiv), sodium ascorbate (35.6 mg, 180 µmol, 1.00 equiv) and 1-ethynyl-4-methoxybenzene (30.9 mg, 30.3 µL, 234 µmol, 1.30 equiv) were added. The reaction mixture was stirred at 50 °C for 16 hours. For work up the reaction mixture was diluted in water and extracted three times with methylene chloride. The combined organic phase was washed with water and brine, dried over Na_2SO_4 and filtered. As

preparation for the column chromatography (dryload), Celite was added (0.2 g) and the reaction mixture with Celite were evaporated. The obtained crude product was purified *via* flash-chromatography (Interchim devices puriFLASH 5.125) on silica gel (PF-15SIHP-F0012) using cyclohexane/ethyl acetate 0% to 40% ethyl acetate in 15 column volumes (1 column volume = 39.3 mL; flow: 15 mL/min). The isolated product 1-(1-(4-bromobenzyl)-1*H*-pyrazol-3-yl)-4-(4-methoxyphenyl)-1*H*-1,2,3-triazole (73.1 mg, 178 µmol) was obtained as a colorless solid in 99% yield.

R_f = 0.59 (cyclohexane/ethyl acetate 1:1). MP = 138–141 °C. ^{1}H NMR (400 MHz, Chloroform-d [7.27 ppm], ppm) δ = 8.30 (s, 1H), 7.83–7.81 (m, 2H), 7.50–7.47 (m, 2H), 7.45 (d, *J* = 2.3 Hz, 1H), 7.13 (d, *J* = 8.4 Hz, 2H), 6.99–6.96 (m, 2H), 6.77 (d, *J* = 2.4 Hz, 1H), 5.25 (s, 2H), 3.84 (s, 3H); ^{13}C NMR (100 MHz, Chloroform-d [77.0 ppm], ppm) δ = 159.7, 147.5, 146.6, 134.6, 132.0 (2C), 131.6, 129.3 (2C), 127.1 (2C), 122.8, 122.4, 116.3, 114.2 (2C), 97.8, 55.7, 55.3; MS (FAB, 3-NBA), m/z (%): 410/412 (100/97) $[M+1]^+$, 381/383 (12/13), 307 (13), 212 (70), 171/169 (26/27), 154 (62), 136 (45), 121 (12), 107 (13), 89 (16). HRMS–FAB ($C_{19}H_{17}ON_5Br$) *(m/z)*: $[M+H]^+$ Calcd 410.0611; Found 410.0610; IR (ATR, ṽ) = 3149 (w), 3111 (w), 3065 (w), 3009 (vw), 2956 (w), 2927 (w), 2837 (w), 1616 (w), 1561 (w), 1541 (m), 1520 (w), 1494 (vs), 1456 (w), 1439 (m), 1425 (w), 1404 (w), 1288 (m), 1251 (s), 1227 (s), 1196 (w), 1184 (w), 1171 (s), 1123 (w), 1106 (w), 1064 (m), 1048 (m), 1024 (vs), 1009 (m), 973 (m), 936 (w), 836 (vs), 799 (vs), 751 (vs), 704 (w), 696 (w), 653 (w), 637 (w), 628 (w), 619 (m), 585 (w), 526 (s), 494 (s), 450 (m), 424 (w), 409 (w), 398 (w), 391 (w), 382 (w) cm^{-1}.

Additional information on the chemical synthesis is available *via* Chemotion repository: https://doi.org/10.14272/reaction/SA-FUHFF-UHFFFADPSC-QXNOXDRDOZ-UHFFFADPSC-NUHFF-NUHFF-NUHFF-ZZZ

Additional information on the analysis of the target compound is available *via* Chemotion repository:
https://doi.org/10.14272/QXNOXDRDOZJZFO-UHFFFAOYSA-N.1

4-Butyl-1-(5-methyl-1*H*-pyrazol-3-yl)-1*H*-1,2,3-triazole (21ea)

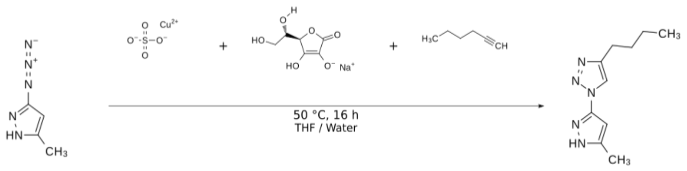

Name {P1|**21ea**}: 4-butyl-1-(5-methyl-1*H*-pyrazol-3-yl)-1*H*-1,2,3-triazole; Formula: $C_{10}H_{15}N_5$; Smiles: CCCCc1nnn(c1)c1n[nH]c(c1)C. InChIKey: QKYKTOGQTOIHKO-UHFFFAOYSA-N

In a vial, 3-azido-5-methyl-1*H*-pyrazole (163 mg, 1.32 mmol, 1.00 equiv) was dissolved in THF (20.00 mL) and water (20.00 mL) (1:1) and copper;sulfate (42.3 mg, 265 µmol, 0.20 equiv), sodium ascorbate (262 mg, 1.32 mmol, 1.00 equiv) and hex-1-yne (141 mg, 196 µL, 1.72 mmol, 1.30 equiv) were added. The reaction mixture was stirred at 50 °C for 16 hours. For workup, the reaction mixture was diluted in water and extracted three times with methylene chloride. The combined organic phases were washed with water and brine, dried over Na_2SO_4, filtered and the solvent was removed

under reduced pressure. As preparation for column chromatography (dry load), Celite was added (0.6 g) and the reaction mixture with Celite was evaporated. The obtained crude product was purified *via* flash-chromatography (Interchim devices puriFLASH 5.125) on silica gel (PF-15SIHP-F0025) using cyclohexane/ethyl acetate with 0% to 50% ethyl acetate in 17 column volumes. The isolated product 4-butyl-1-(5-methyl-1*H*-pyrazol-3-yl)-1*H*-1,2,3-triazole (182 mg, 887 µmol) was obtained as a light brown solid in 67% yield.

R_f = 0.30 (cyclohexane/ethyl acetate 1:1). MP = 235–238 °C. ^{1}H NMR (400 MHz, Chloroform-d [7.27 ppm], ppm) δ = 10.65 (br, s, 1H), 7.90 (s, 1H), 6.49 (s, 1H), 2.79 (t, *J* = 7.7 Hz, 2H), 2.39 (s, 3H), 1.74–1.67 (m, 2H), 1.42 (h, *J* = 7.3 Hz, 2H), 0.94 (t, *J* = 7.3 Hz, 3H); ^{13}C NMR (100 MHz, Chloroform-d [77.0 ppm], ppm) δ = 148.3 (C_q), 147.3 (C_q), 141.5 (C_q), 118.6 (CH), 96.0 (CH), 31.3 (CH_2), 25.2 (CH_2), 22.2 (CH_2), 13.7 (CH_3), 11.1 (CH_3); MS (EI, 70 eV, 110 °C), m/z (%): 205 (2) $[M]^+$, 176 (13), 162 (11), 134 (100), 108 (15). HRMS–EI ($C_{10}H_{15}N_5$) *(m/z)*: $[M]^+$ Calcd 205.1322; Found 205.1321; IR (ATR, ṽ) = 3189 (w), 3159 (w), 3106 (w), 3057 (w), 2982 (w), 2952 (vs), 2931 (s), 2863 (s), 2744 (w), 1587 (m), 1557 (m), 1538 (vs), 1485 (w), 1458 (s), 1443 (m), 1380 (w), 1360 (w), 1306 (w), 1247 (m), 1224 (vs), 1174 (w), 1071 (m), 1047 (vs), 1011 (vs), 965 (w), 948 (vs), 898 (w), 834 (vs), 813 (vs), 786 (vs), 778 (vs), 742 (vs), 688 (w), 671 (w), 645 (s), 517 (w), 455 (w), 399 (m), 387 (w) cm^{-1}.

Additional information on the chemical synthesis is available *via* Chemotion repository: https://doi.org/10.14272/reaction/SA-FUHFF-UHFFFADPSC-QKYKTOGQTO-UHFFFADPSC-NUHFF-NUHFF-NUHFF-ZZZ

Additional information on the analysis of the target compound is available *via* Chemotion repository:
https://doi.org/10.14272/QKYKTOGQTOIHKO-UHFFFAOYSA-N.1

Methyl 1-(5-methyl-1*H*-pyrazol-3-yl)triazole-4-carboxylate (21eb)

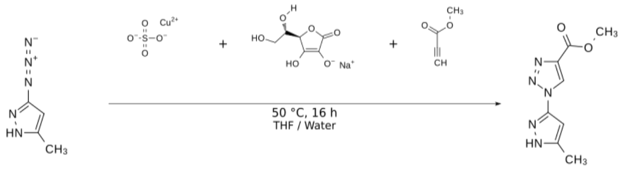

Name {P1|**21eb**}: methyl 1-(5-methyl-1*H*-pyrazol-3-yl)triazole-4-carboxylate; Formula: $C_8H_9N_5O_2$; Smiles: COC(=O)c1nnn(c1)c1n[nH]c(c1)C. InChIKey: AXYCMAQNQSYSIG-UHFFFAOYSA-N

In a vial, 3-azido-5-methyl-1*H*-pyrazole (50.0 mg, 406 µmol, 1.00 equiv) was dissolved in THF (6 mL) and water (6 mL) (1:1) and copper;sulfate (13.0 mg, 81.2 µmol, 0.20 equiv), sodium ascorbate (80.5 mg, 406 µmol, 1.00 equiv) and methyl prop-2-ynoate (44.4 mg, 47.0 µL, 528 µmol, 1.30 equiv) were added. The reaction mixture was stirred at 50 °C for 16 hours. For workup, the reaction mixture was diluted in water and extracted three times with methylene chloride. The combined organic phases were washed with water and brine, dried over Na_2SO_4, filtered and the solvent was removed under reduced pressure. As preparation for column chromatography (dry load), Celite was added (0.2 g) and the reaction mixture with Celite was evaporated. The obtained

crude product was purified *via* flash-chromatography (Biotage devices) on silica gel (PF-15SIHP-F0012) using dichloromethane/ methanol with 0% to 10% methanol in 11 column volumes. The isolated product methyl 1-(5-methyl-1*H*-pyrazol-3-yl)triazole-4-carboxylate (32.2 mg, 155 µmol) was obtained as a light yellow solid in 38% yield.

R_f = 0.16 (cyclohexane/ethyl acetate 1:1). MP = 251–253 °C. ^{1}H NMR (400 MHz, DMSO-d_6 [2.50 ppm], ppm) δ = 13.04 (s, 1H), 9.10 (s, 1H), 6.55 – 6.50 (m, 1H), 3.87 (s, 3H), 2.34 – 2.29 (m, 3H). ^{13}C NMR (100 MHz, DMSO-d_6 [39.5 ppm], ppm) δ = 160.4 (C_q), 145.2 (C_q), 141.3 (C_q), 138.9 (C_q), 126.4 (CH), 96.1 (CH), 51.9 (CH_3), 10.6 (CH_3); MS (EI, 70 eV, 170 °C), m/z (%): 207 (53) [M]$^+$, 191 (13), 178 (39), 176 (12), 148 (100), 136 (29), 120 (71), 108 (21), 57 (13). HRMS–EI ($C_8H_9O_2N_5$) *(m/z)*: [M]$^+$ Calcd 207.0751; found 207.0749; IR (ATR, ṽ) = 3203 (w), 3148 (m), 3119 (w), 2945 (w), 2928 (w), 2853 (w), 1720 (vs), 1572 (m), 1561 (s), 1543 (s), 1477 (w), 1466 (w), 1449 (m), 1428 (m), 1377 (w), 1336 (vs), 1303 (s), 1258 (m), 1230 (vs), 1210 (vs), 1150 (m), 1133 (m), 1038 (vs), 1016 (s), 946 (vs), 840 (s), 823 (vs), 812 (vs), 768 (vs), 696 (w), 686 (w), 629 (w), 465 (w) cm^{-1}.

Additional information on the chemical synthesis is available *via* Chemotion repository: https://doi.org/10.14272/reaction/SA-FUHFF-UHFFFADPSC-AXYCMAQNQS-UHFFFADPSC-NUHFF-NUHFF-NUHFF-ZZZ

Additional information on the analysis of the target compound is available *via* Chemotion repository:
https://doi.org/10.14272/AXYCMAQNQSYSIG-UHFFFAOYSA-N.1

This compound has been previously synthesized and the analytical data reported herein are consistent with the literature. [9]

(1-(5-Methyl-1*H*-pyrazol-3-yl)-1*H*-1,2,3-triazol-4-yl)methyl acetate (21ec)

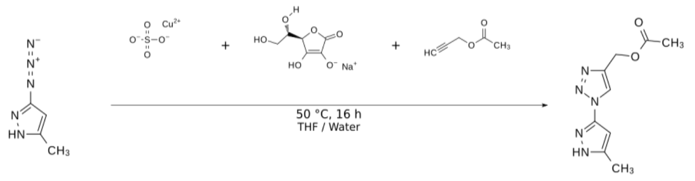

Name {P1|**21ec**}: (1-(5-methyl-1*H*-pyrazol-3-yl)-1*H*-1,2,3-triazol-4-yl)methyl acetate; Formula: $C_9H_{11}N_5O_2$; Smiles: CC(=O)OCc1nnn(c1)c1n[nH]c(c1)C. InChIKey: LUTGSYAMTQUJGA-UHFFFAOYSA-N

In a vial, 3-azido-5-methyl-1*H*-pyrazole (50.0 mg, 406 µmol, 1.00 equiv) was dissolved in THF (6 mL) and water (6 mL) (1:1) and copper;sulfate (13.0 mg, 81.2 µmol, 0.20 equiv), sodium ascorbate (80.5 mg, 406 µmol, 1.00 equiv) and prop-2-ynyl acetate (51.8 mg, 52.4 µL, 528 µmol, 1.30 equiv) were added. The reaction mixture was stirred at 50 °C for 16 hours. For work up the reaction mixture was diluted in water and extracted three times with methylene chloride. The combined organic phase was washed with water and brine, dried over Na_2SO_4, filtered and the solvent was removed under reduced pressure. As preparation for the column chromatography (dryload), Celite was added (0.2 g) and the reaction mixture with Celite were evaporated. The obtained crude product was purified *via* flash-chromatography (Interchim devices puriFLASH 5.125) on silica gel (PF-15SIHP-F0012) using cyclohexane/ethyl acetate

0% to 100% ethyl acetate in 35 column volumes. The isolated product (1-(5-methyl-1*H*-pyrazol-3-yl)-1*H*-1,2,3-triazol-4-yl)methyl acetate (73.2 mg, 331 µmol) was obtained as a colorless solid in 81% yield.

R_f = 0.14 (cyclohexane/ethyl acetate 1:1). ^{1}H NMR (400 MHz, Chloroform-d [7.27 ppm], ppm) δ = 11.13 (s, 1H), 8.26 (s, 1H), 6.49 (s, 1H), 5.28 (s, 2H), 2.39 (s, 3H), 2.08 (s, 3H); ^{13}C NMR (100 MHz, Chloroform-d [77.0 ppm], ppm) δ = 171.0, 147.0, 142.8, 141.7, 121.7, 96.2, 57.5, 20.9, 11.3; MS (EI, 70 eV, 120 °C), m/z (%): 221 (7) $[M]^+$, 181 (15), 151 (37), 150 (55), 134 (100), 131 (14), 123 (19), 108 (20), 82 (16), 69 (14). HRMS–EI ($C_9H_{11}O_2N_5$) *(m/z)*: $[M]^+$ Calcd 221.0907; Found 221.0909; IR (ATR, ṽ) = 3203 (w), 3174 (w), 3152 (w), 3104 (w), 3064 (w), 2969 (w), 2912 (w), 2868 (w), 2772 (w), 1734 (vs), 1659 (w), 1594 (w), 1558 (w), 1538 (m), 1475 (w), 1438 (m), 1431 (w), 1395 (m), 1367 (m), 1332 (w), 1299 (w), 1281 (w), 1242 (vs), 1227 (vs), 1218 (vs), 1176 (m), 1054 (vs), 1037 (vs), 1026 (s), 1014 (s), 999 (vs), 959 (s), 948 (vs), 834 (vs), 823 (vs), 775 (vs), 765 (vs), 701 (m), 677 (m), 636 (s), 606 (m), 550 (w), 507 (m), 492 (w), 418 (w), 401 (w), 391 (w) cm^{-1}.

Additional information on the chemical synthesis is available *via* Chemotion repository: https://doi.org/10.14272/reaction/SA-FUHFF-UHFFFADPSC-LUTGSYAMTQ-UHFFFADPSC-NUHFF-NUHFF-NUHFF-ZZZ

Additional information on the analysis of the target compound is available *via* Chemotion repository:
https://doi.org/10.14272/LUTGSYAMTQUJGA-UHFFFAOYSA-N.1

1-(5-Methyl-1*H*-pyrazol-3-yl)-4-phenyl-1*H*-1,2,3-triazole (21ed)

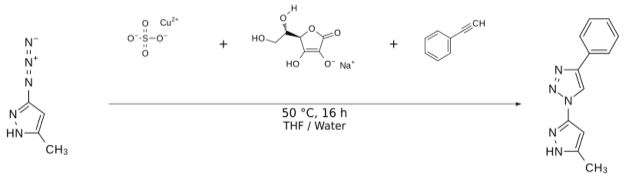

Name {P1|**21ed**}: 1-(5-methyl-1*H*-pyrazol-3-yl)-4-phenyl-1*H*-1,2,3-triazole; Formula: $C_{12}H_{11}N_5$; Smiles: Cc1[nH]nc(c1)n1nnc(c1)c1ccccc1. InChIKey: BTYPCTNIHDBBSH-UHFFFAOYSA-N

In a vial, 3-azido-5-methyl-1*H*-pyrazole (50.0 mg, 406 µmol, 1.00 equiv) was dissolved in THF (8.00 mL) and water (8.00 mL) (1:1) and copper;sulfate (13.0 mg, 81.2 µmol, 0.20 equiv), sodium ascorbate (80.5 mg, 406 µmol, 1.00 equiv) and ethynylbenzene (53.9 mg, 58.0 µL, 528 µmol, 1.30 equiv) were added. The reaction mixture was stirred at 50 °C for 16 hours. For workup, the reaction mixture was diluted in water and extracted three times with methylene chloride. The combined organic phases were washed with water and brine, dried over Na_2SO_4, filtered and the solvent was removed under reduced pressure. As preparation for column chromatography (dry load), Celite was added (0.2 g) and the reaction mixture with additional Celite was evaporated. The obtained crude product was purified *via* flash-chromatography (Interchim devices puriFLASH 5.125) on silica gel (PF-15SIHP-F0012) using cyclohexane/ethyl acetate with 0% to 50% ethyl acetate in 25 column volumes. The isolated product 1-(5-methyl-

1*H*-pyrazol-3-yl)-4-phenyl-1*H*-1,2,3-triazole (52.0 mg, 231 µmol) was obtained as colourless solid in 57% yield.

R_f = 0.14 (cyclohexane/ethyl acetate 2:1). MP = 174–176 °C. ^{1}H NMR (400 MHz, DMSO-d_6 [2.50 ppm], ppm) δ = 12.97 (s, 1H), 9.05 (s, 1H), 7.99 (d, *J* = 7.3 Hz, 2H), 7.46 (t, *J* = 7.6 Hz, 2H), 7.36 (t, *J* = 7.4 Hz, 1H), 6.51 (s, 1H), 2.33 (s, 3H). ^{13}C NMR (100 MHz, DMSO-d_6 [39.5 ppm], ppm) δ = 146.6 (C_q), 146.0 (C_q), 141.1 (C_q), 130.2 (C_q), 128.9 (CH, 2C), 128.1 (CH), 125.4 (CH, 2C), 118.8 (CH), 95.5 (CH), 10.6 (CH_3); MS (EI, 70 eV, 130 °C), m/z (%): 225 (9) $[M]^+$, 197 (100), 182 (16), 168 (15), 156 (34), 129 (14), 116 (22), 102 (44), 89 (32), 63 (11). HRMS–EI ($C_{12}H_{11}N_5$) *(m/z)*: $[M]^+$ Calcd 225.1009; Found 225.1008; IR (ATR, ṽ) = 3189 (w), 3143 (w), 3114 (w), 3057 (w), 2982 (w), 2958 (w), 2922 (w), 2861 (w), 2751 (w), 1584 (w), 1557 (w), 1537 (m), 1490 (w), 1479 (m), 1449 (m), 1366 (w), 1302 (w), 1224 (w), 1194 (w), 1180 (w), 1143 (vw), 1074 (w), 1054 (m), 1037 (w), 1027 (w), 1011 (m), 979 (w), 945 (m), 914 (w), 813 (m), 805 (m), 761 (vs), 707 (w), 691 (vs), 671 (s), 654 (w), 618 (w), 535 (w), 504 (m), 497 (w), 422 (w), 391 (w) cm^{-1}.

Additional information on the chemical synthesis is available *via* Chemotion repository: https://doi.org/10.14272/reaction/SA-FUHFF-UHFFFADPSC-BTYPCTNIHD-UHFFFADPSC-NUHFF-NUHFF-NUHFF-ZZZ

Additional information on the analysis of the target compound is available *via* Chemotion repository:
https://doi.org/10.14272/BTYPCTNIHDBBSH-UHFFFAOYSA-N.1

4-(4-Methoxyphenyl)-1-(5-methyl-1*H*-pyrazol-3-yl)-1*H*-1,2,3-triazole (21ee)

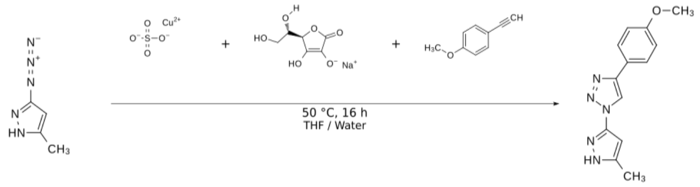

Name {P1|**21ee**}: 4-(4-methoxyphenyl)-1-(5-methyl-1*H*-pyrazol-3-yl)-1*H*-1,2,3-triazole; Formula: $C_{13}H_{13}N_5O$; Smiles: COc1ccc(cc1)c1nnn(c1)c1n[nH]c(c1)C. InChIKey: WNRIJNCEJHFAPB-UHFFFAOYSA-N

In a vial, 3-azido-5-methyl-1*H*-pyrazole (50.0 mg, 406 µmol, 1.00 equiv) was dissolved in THF (8.00 mL) and water (8.00 mL) (1:1) and copper sulfate (13.0 mg, 81.2 µmol, 0.20 equiv), sodium ascorbate (80.5 mg, 406 µmol, 1.00 equiv) and 1-ethynyl-4-methoxybenzene (69.8 mg, 68.5 µL, 528 µmol, 1.30 equiv) were added. The reaction mixture was stirred at 50 °C for 16 hours. For workup, the reaction mixture was diluted in water and extracted three times with methylene chloride. The combined organic phase was washed with water and brine, dried over Na_2SO_4, filtered and the solvent was removed under reduced pressure. As preparation for the column chromatography (dryload), Celite was added (0.2 g) and the reaction mixture with Celite was evaporated. The obtained crude product was purified *via* flash-chromatography (Interchim devices puriFLASH 5.125) on silica gel (PF-15SIHP-F0012) using cyclohexane/ethyl acetate 0% to 65% ethyl acetate in 25 column volumes. The isolated

product 4-(4-methoxyphenyl)-1-(5-methyl-1*H*-pyrazol-3-yl)-1*H*-1,2,3-triazole (62.7 mg, 246 µmol) was obtained as a colorless solid in 60% yield.

R_f = 0.26 (cyclohexane/ethyl acetate1:1). ^{1}H NMR (400 MHz, DMSO-d_6 [2.50 ppm], ppm) δ = 12.94 (s, 1H), 8.92 (s, 1H), 7.92–7.89 (m, 2H), 7.04–7.01 (m, 2H), 6.49 (s, 1H), 3.79 (s, 3H), 2.32 (s, 3H); ^{13}C NMR (100 MHz, DMSO-d_6 [39.5 ppm], ppm) δ = 159.2, 146.6, 146.1, 141.1, 126.8 (2C), 122.8, 117.8, 114.3 (2C), 95.4, 55.1, 10.6; MS (EI, 70 eV, 150 °C), m/z (%): 255 (19) [M]$^+$, 227 (100), 212 (89), 186 (59), 171 (10), 158 (12), 155 (12), 132 (45), 117 (13), 89 (30), 77 (13), 63 (10), 57 (10). HRMS–EI ($C_{13}H_{13}ON_5$) *(m/z)*: [M]$^+$ Calcd 255.1115; Found 255.1115; IR (ATR, ṽ) = 3196 (w), 3108 (w), 3054 (w), 2980 (w), 2958 (w), 2944 (w), 2924 (w), 2897 (w), 2866 (w), 2837 (w), 2758 (w), 1615 (w), 1592 (w), 1581 (w), 1562 (w), 1538 (w), 1497 (s), 1480 (m), 1470 (m), 1453 (m), 1442 (m), 1421 (w), 1366 (w), 1324 (vw), 1305 (vw), 1285 (m), 1245 (vs), 1224 (s), 1194 (w), 1176 (m), 1147 (w), 1111 (w), 1057 (s), 1028 (s), 1013 (s), 980 (m), 958 (w), 945 (s), 836 (vs), 795 (vs), 783 (vs), 762 (s), 727 (w), 705 (w), 686 (w), 656 (w), 636 (w), 615 (s), 543 (s), 517 (s), 453 (w), 418 (w), 390 (w) cm^{-1}.

Additional information on the chemical synthesis is available *via* Chemotion repository: https://doi.org/10.14272/reaction/SA-FUHFF-UHFFFADPSC-WNRIJNCEJH-UHFFFADPSC-NUHFF-NUHFF-NUHFF-ZZZ.1

Additional information on the analysis of the target compound is available *via* Chemotion repository:
https://doi.org/10.14272/WNRIJNCEJHFAPB-UHFFFAOYSA-N.2

Methyl 4-(1-(5-methyl-1*H*-pyrazol-3-yl)-1*H*-1,2,3-triazol-4-yl)benzoate (21ef)

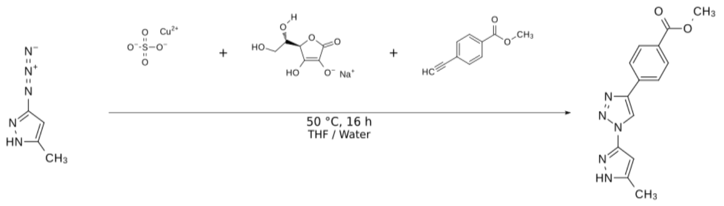

Name {P1|**21ef**}: methyl 4-(1-(5-methyl-1*H*-pyrazol-3-yl)-1*H*-1,2,3-triazol-4-yl)benzoate; Formula: $C_{14}H_{13}N_5O_2$; Smiles: COC(=O)c1ccc(cc1)c1nnn(c1)c1n[nH]c(c1)C. InChIKey: ICPVQAYXOPZIKF-UHFFFAOYSA-N

In a vial, 3-azido-5-methyl-1*H*-pyrazole (50.0 mg, 406 µmol, 1.00 equiv) was dissolved in THF (8.00 mL) and water (8.00 mL) (1:1) and copper;sulfate (13.0 mg, 81.2 µmol, 0.20 equiv), sodium ascorbate (80.5 mg, 406 µmol, 1.00 equiv) and methyl 4-ethynylbenzoate (84.6 mg, 528 µmol, 1.30 equiv) were added. The reaction mixture was stirred at 50 °C for 16 hours. For workup, the reaction mixture was diluted in water and extracted three times with methylene chloride. The combined organic phase was washed with water and brine, dried over Na_2SO_4 and filtered. As preparation for the column chromatography (dry load), Celite was added (0.2 g) and the reaction mixture with Celite was evaporated. The obtained crude product was purified *via* flash-chromatography (Interchim devices puriFLASH 5.125) on silica gel (PF-15SIHP-F0012) using cyclohexane/ethyl acetate 0% to 100% ethyl acetate in 30 column volumes (1 column volume = 39.3 mL; flow: 15 mL/min). The isolated product methyl

4-(1-(5-methyl-1*H*-pyrazol-3-yl)-1*H*-1,2,3-triazol-4-yl)benzoate (49.0 mg, 173 µmol) was obtained as light yellow solid in 43% yield.

R_f = 0.28 (cyclohexane/ethyl acetate 1:1). MP = 191–236.7 °C. ^{1}H NMR (400 MHz, DMSO-d_6 [2.50 ppm], ppm) δ = 12.99 (s, 1H), 9.20 (s, 1H), 8.14–8.12 (m, 2H), 8.05–8.03 (m, 2H), 6.51 (dd, *J* = 2.2 Hz, *J* = 0.9 Hz, 1H), 3.87 (s, 3H), 2.33 (s, 3H); ^{13}C NMR (100 MHz, DMSO-d_6 [39.5 ppm], ppm) δ = 165.9, 145.9, 145.5, 141.2, 134.7, 129.8 (2C), 128.9, 125.5 (2C), 120.0, 95.5, 52.1, 10.6; MS (EI, 70 eV, 170 °C), m/z (%): 531 (10), 441 (11), 283 (4) $[M]^+$, 255 (100), 224 (28), 214 (18), 196 (14), 162 (10), 155 (15), 129 (16), 101 (12), 89 (15). HRMS–EI ($C_{14}H_{13}O_2N_5$) *(m/z)*: $[M]^+$ Calcd 283.1064; Found 283.1065; IR (ATR, ṽ) = 3190 (w), 3148 (w), 3121 (w), 2990 (w), 2951 (m), 2924 (m), 2853 (m), 1717 (vs), 1679 (w), 1613 (w), 1584 (w), 1533 (m), 1493 (w), 1458 (w), 1432 (s), 1417 (m), 1384 (w), 1363 (w), 1310 (w), 1283 (s), 1272 (vs), 1232 (s), 1224 (m), 1191 (s), 1177 (s), 1147 (w), 1120 (w), 1106 (s), 1095 (s), 1055 (s), 1037 (m), 1014 (s), 980 (m), 969 (s), 945 (s), 905 (m), 888 (m), 854 (vs), 812 (vs), 765 (vs), 721 (m), 707 (s), 690 (vs), 652 (m), 635 (m), 606 (w), 581 (w), 541 (w), 511 (s), 500 (s), 477 (w), 459 (w), 435 (m), 421 (w), 404 (w) cm^{-1}.

Additional information on the chemical synthesis is available *via* Chemotion repository: https://doi.org/10.14272/reaction/SA-FUHFF-UHFFFADPSC-ICPVQAYXOP-UHFFFADPSC-NUHFF-NUHFF-NUHFF-ZZZ

Additional information on the analysis of the target compound is available *via* Chemotion repository:
https://doi.org/10.14272/ICPVQAYXOPZIKF-UHFFFAOYSA-N.1

1-(1-benzyl-5-methyl-1*H*-pyrazol-3-yl)-4-butyl-1*H*-1,2,3-triazole (21fa)

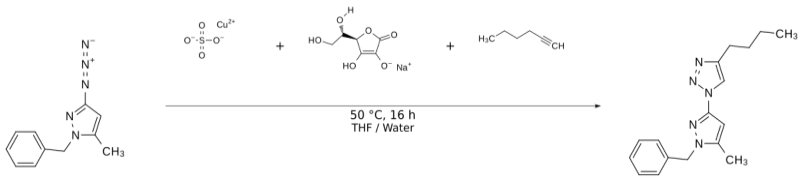

Name {P1|**21fa**}: 1-(1-benzyl-5-methyl-1*H*-pyrazol-3-yl)-4-butyl-1*H*-1,2,3-triazole; Formula: $C_{17}H_{21}N_5$; CAS: - ; Molecular Mass: 295.3821; Exact Mass: 295.1797; EA: C, 69.12; H, 7.17; N, 23.71. Smiles: CCCCc1nnn(c1)c1cc(n(n1)Cc1ccccc1)C. InChIKey: PKNOMBYYAWYDCG-UHFFFAOYSA-N

In a vial, 3-azido-1-benzyl-5-methyl-pyrazole (100 mg, 469 µmol, 1.00 equiv) was dissolved in THF (8.00 mL) and water (8.00 mL) (1:1) and copper sulfate (15.0 mg, 93.8 µmol, 0.200 equiv), sodium ascorbate (92.9 mg, 469 µmol, 1.00 equiv) and hex-1-yne (50.1 mg, 69.6 µL, 610 µmol, 1.30 equiv) were added. The reaction mixture was stirred at 50 °C for 16 hours. For work up the reaction mixture was diluted in water and extracted three times with methylene chloride. The combined organic phase was washed with water and brine, dried over Na_2SO_4 and filtered. As preparation for the column chromatography (dryload), Celite was added (0.3 g) and the reaction mixture with Celite were evaporated. The obtained crude product was purified *via* flash-chromatography (Interchim devices puriFLASH 5.125) on silica gel (PF-15SIHP-F0025) using cyclohexane/ethyl acetate 0% to 20% ethyl acetate in 12 column

volumes (1 column volume = 63.8 mL; flow: 15 mL/min). The isolated product 1-(1-benzyl-5-methyl-1H-pyrazol-3-yl)-4-butyl-1H-1,2,3-triazole (96.2 mg, 326 µmol) was obtained as a light yellow solid in 69% yield.

R_f = 0.33 (cyclohexane/ethyl acetate 2:1). MP = 56.5–58.3 °C. ^{1}H NMR (400 MHz, Chloroform-d [7.26 ppm], ppm) δ = 7.91 (s, 1H), 7.36–7.28 (m, 3H), 7.13 (d, *J* = 6.7 Hz, 2H), 6.53 (d, *J* = 0.5 Hz, 1H), 5.28 (s, 2H), 2.78 (t, *J* = 7.6 Hz, 2H), 2.26 (d, *J* = 0.4 Hz, 3H), 1.70 (quint, *J* = 7.5 Hz, 2H), 1.41 (h, *J* = 7.3 Hz, 2H), 0.94 (t, *J* = 7.4 Hz, 3H); ^{13}C NMR (100 MHz, Chloroform-d [77.16 ppm], ppm) δ = 148.4 (C_q), 145.9 (C_q), 141.2 (C_q), 136.3 (C_q), 129.0 (2C, CH), 128.1 (CH), 126.8 (2C, CH), 118.5 (CH), 97.5 (CH), 53.3 (CH_2), 31.5 (CH_2), 25.4 (CH_2), 22.4 (CH_2), 14.0 (CH_3), 11.6 (CH_3); MS (EI, 70 eV, 160 °C), m/z (%): 295 (2) $[M]^+$, 267 (37), 224 (100), 176 (33), 169 (11), 91 (74), 69 (25). HRMS–EI ($C_{17}H_{21}N_5$) *(m/z)*: $[M]^+$ Calcd 295.1791; Found 295.1790; IR (ATR, ṽ) = 3145 (w), 3131 (vw), 3064 (w), 3033 (vw), 2955 (m), 2927 (m), 2871 (w), 2861 (w), 1558 (vs), 1545 (s), 1536 (s), 1494 (m), 1490 (m), 1453 (vs), 1436 (s), 1404 (s), 1366 (s), 1315 (m), 1303 (w), 1241 (w), 1218 (vs), 1122 (w), 1077 (w), 1037 (vs), 1017 (m), 984 (w), 963 (w), 945 (s), 925 (w), 795 (vs), 755 (w), 728 (vs), 694 (vs), 679 (m), 663 (m), 642 (m), 637 (m), 616 (w), 575 (m), 456 (m), 419 (w), 401 (w), 391 (m) cm^{-1}.

Additional information on the chemical synthesis is available *via* Chemotion repository: https://doi.org/10.14272/reaction/SA-FUHFF-UHFFFADPSC-PKNOMBYYAW-UHFFFADPSC-NUHFF-NUHFF-NUHFF-ZZZ

Additional information on the analysis of the target compound is available *via* Chemotion repository:
https://doi.org/10.14272/PKNOMBYYAWYDCG-UHFFFAOYSA-N.1

1-(1-Benzyl-5-methyl-1*H*-pyrazol-3-yl)-4-(4-methoxyphenyl)-1*H*-1,2,3-triazole (21fe)

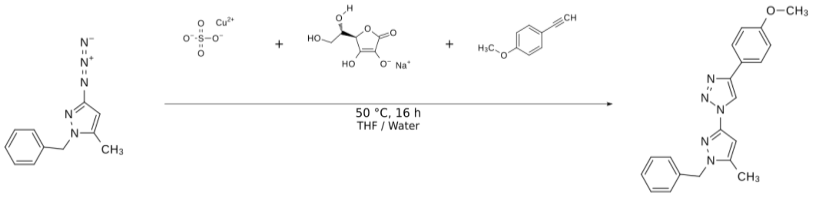

Name {P1|**21fe**}: 1-(1-benzyl-5-methyl-1*H*-pyrazol-3-yl)-4-(4-methoxyphenyl)-1*H*-1,2,3-triazole; Formula: $C_{20}H_{19}N_5O$; Smiles: COc1ccc(cc1)c1nnn(c1)c1nn(c(c1)C)Cc1ccccc1. InChIKey: HKYHACNKJZVDCN-UHFFFAOYSA-N

In a vial, 3-azido-1-benzyl-5-methyl-pyrazole (69.9 mg, 328 µmol, 1.00 equiv) was dissolved in THF (8.00 mL) and water (8.00 mL) (1:1) and copper;sulfate (10.5 mg, 65.6 µmol, 0.200 equiv), sodium ascorbate (64.9 mg, 328 µmol, 1.00 equiv) and 1-ethynyl-4-methoxybenzene (56.3 mg, 55.3 µL, 426 µmol, 1.30 equiv) were added. The reaction mixture was stirred at 50 °C for 16 hours. Then it was diluted with water and extracted three times with methylene chloride. The combined organic phase was washed with water and brine, dried over Na_2SO_4 and filtered. As preparation for the column chromatography (dryload), Celite was added (0.3 g) and the reaction mixture with Celite was evaporated. The obtained crude product was purified *via* flash-

chromatography (Interchim devices puriFLASH 5.125) on silica gel (PF-15SIHP-F0025) using cyclohexane/ethyl acetate 0% to 35% ethyl acetate in 12 column volumes (1 column volume = 63.8 mL; flow: 15 mL/min). The isolated product 1-(1-benzyl-5-methyl-1*H*-pyrazol-3-yl)-4-(4-methoxyphenyl)-1*H*-1,2,3-triazole (100 mg, 290 µmol) was obtained as a colorless solid in 88% yield.

R_f = 0.61 (cyclohexane/ethyl acetate 1:1). MP = 109–110.8 °C. ^{1}H NMR (400 MHz, Chloroform-d [7.27 ppm], ppm) δ = 8.34 (s, 1H), 7.86–7.82 (m, 2H), 7.38–7.29 (m, 3H), 7.17–7.15 (m, 2H), 7.01–6.97 (m, 2H), 6.61 (d, *J* = 0.6 Hz, 1H), 5.31 (s, 2H), 3.86 (s, 3H), 2.28 (s, 3H); ^{13}C NMR (100 MHz, Chloroform-d [77.0 ppm], ppm) δ = 159.7 (C_q), 147.4 (C_q), 145.6 (C_q), 141.1 (C_q), 136.1 (C_q), 128.9 (CH, 2C), 128.0 (CH), 127.2 (CH, 2C), 126.7 (CH, 2C), 123.1 (C_q), 116.2 (CH), 114.3 (CH, 2C), 97.4 (CH), 55.3 (CH_3), 53.3 (CH_2), 11.5 (CH_3); MS (FAB, 3-NBA), m/z (%): 346 (100) $[M+1]^+$, 318 (10), 226 (16), 91 (34). HRMS–FAB ($C_{20}H_{20}ON_5$) *(m/z)*: $[M+H]^+$ Calcd 346.1662; Found 346.1661; IR (ATR, ṽ) = 3148 (w), 2928 (w), 2834 (w), 1615 (w), 1561 (s), 1538 (m), 1499 (vs), 1482 (vs), 1456 (w), 1442 (m), 1425 (m), 1397 (w), 1384 (w), 1353 (w), 1329 (w), 1302 (w), 1288 (s), 1241 (vs), 1200 (w), 1176 (s), 1116 (w), 1103 (w), 1055 (w), 1028 (vs), 989 (w), 973 (m), 959 (w), 948 (s), 924 (w), 834 (vs), 820 (w), 800 (m), 783 (vs), 732 (vs), 703 (vs), 666 (s), 633 (m), 615 (vs), 577 (s), 545 (w), 528 (s), 470 (m), 460 (m), 399 (w) cm^{-1}.

Additional information on the chemical synthesis is available *via* Chemotion repository: https://doi.org/10.14272/reaction/SA-FUHFF-UHFFFADPSC-HKYHACNKJZ-UHFFFADPSC-NUHFF-NUHFF-NUHFF-ZZZ

Additional information on the analysis of the target compound is available *via* Chemotion repository:
https://doi.org/10.14272/HKYHACNKJZVDCN-UHFFFAOYSA-N.1

4-(3-(4-butyl-1*H*-1,2,3-triazol-1-yl)-5-methyl-1*H*-pyrazol-1-yl)benzonitrile (**21ga**)

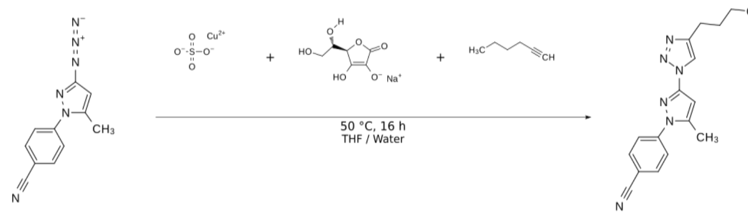

Name {P1|**21ga**}: 4-(3-(4-butyl-1*H*-1,2,3-triazol-1-yl)-5-methyl-1*H*-pyrazol-1-yl)benzonitrile; Formula: $C_{17}H_{18}N_6$; CAS: - ; Molecular Mass: 306.3650; Exact Mass: 306.1593; EA: C, 66.65; H, 5.92; N, 27.43. Smiles: CCCCc1nnn(c1)c1nn(c(c1)C)c1ccc(cc1)C#N. InChIKey: WZTARLNNZGQBDW-UHFFFAOYSA-N.

In a vial, 4-(3-azido-5-methyl-1H-pyrazol-1-yl)benzonitrile (111 mg, 497 µmol, 1.00 equiv) was dissolved in THF (8.00 mL) and water (8.00 mL) (1:1) and copper;sulfate (15.9 mg, 99.4 µmol, 0.200 equiv), sodium ascorbate (98.4 mg, 497 µmol, 1.00 equiv) and hex-1-yne (53.1 mg, 73.7 µL, 646 µmol, 1.30 equiv) were added. The reaction mixture was stirred at 50 °C for 16 hours. For work up the reaction mixture was diluted in water and extracted three times with methylene chloride. The

combined organic phase was washed with water and brine, dried over Na_2SO_4 and filtered. As preparation for the column chromatography (dryload), Celite was added (0.3 g) and the reaction mixture with Celite were evaporated. The obtained crude product was purified *via* flash-chromatography (Interchim devices puriFLASH 5.125) on silica gel (PF-15SIHP-F0025) using cyclohexane/ethyl acetate 0% to 30% ethyl acetate in 15 column volumes (1 column volume = 63.8 mL; flow: 15 mL/min). The isolated product 4-(3-(4-butyl-1*H*-1,2,3-triazol-1-yl)-5-methyl-1*H*-pyrazol-1-yl)benzonitrile (141 mg, 462 µmol) was obtained as a colorless solid in 93% yield.

R_f = 0.31 (cyclohexane/ethyl acetate 2:1). MP = 84.2–85.1 °C. ^{1}H NMR (400 MHz, Chloroform-d [7.26 ppm], ppm) δ = 7.96 (s, 1H), 7.81–7.79 (m, 2H), 7.67–7.65 (m, 2H), 6.76 (s, 1H), 2.78 (t, J = 7.6 Hz, 2H), 2.48 (s, 3H), 1.70 (quint, J = 7.6 Hz, 2H), 1.40 (h, J = 7.3 Hz, 2H), 0.93 (t, J = 7.3 Hz, 3H); ^{13}C NMR (100 MHz, Chloroform-d [77.16 ppm], ppm) δ = 148.8 (C_q), 148.0 (C_q), 142.6 (C_q), 141.8 (C_q), 133.4 (2C, CH), 124.7 (2C, CH), 118.2 (CH), 118.1 (C_q), 111.6 (C_q), 100.1 (CH), 31.4 (CH_2), 25.3 (CH_2), 22.3 (CH_2), 13.9 (CH_3), 13.3 (CH_3); MS (FAB, 3-NBA), m/z (%): 307 (27) $[M+H]^+$, 178 (10), 165 (16), 154 (42), 143 (16), 133 (76), 119 (30), 105 (46), 91 (100). HRMS–FAB ($C_{17}H_{19}N_6$) *(m/z)*: $[M+H]^+$ Calcd 307.1666; Found 307.1665; IR (ATR, ṽ) = 3106 (w), 2959 (w), 2928 (m), 2859 (w), 2225 (m), 1605 (m), 1584 (w), 1561 (m), 1548 (vs), 1537 (s), 1510 (vs), 1460 (w), 1438 (w), 1412 (w), 1388 (vs), 1371 (s), 1315 (w), 1292 (w), 1217 (vs), 1180 (w), 1167 (w), 1129 (w), 1113 (w), 1075 (w), 1038 (vs), 1026 (s), 1011 (s), 984 (m), 943 (s), 844 (vs), 799 (s), 783 (vs), 732 (w), 686 (w), 650 (w), 639 (w), 628 (w), 582 (s), 571 (m), 545 (vs), 462 (w), 431 (w), 415 (w), 408 (w) cm^{-1}.

Additional information on the chemical synthesis is available *via* Chemotion repository: https://doi.org/10.14272/reaction/SA-FUHFF-UHFFFADPSC-WZTARLNNZG-UHFFFADPSC-NUHFF-NUHFF-NUHFF-ZZZ

Additional information on the analysis of the target compound is available *via* Chemotion repository: https://doi.org/10.14272/WZTARLNNZGQBDW-UHFFFAOYSA-N.1

4-(5-methyl-3-(4-phenyl-1*H*-1,2,3-triazol-1-yl)-1*H*-pyrazol-1-yl)benzonitrile (**21gd**)

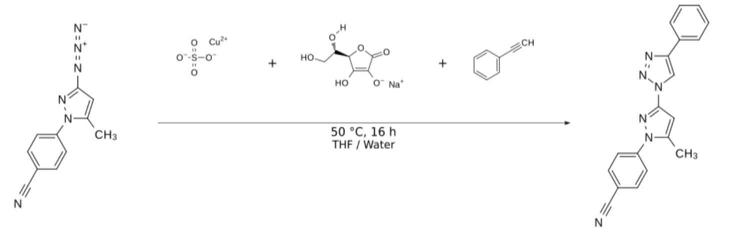

Name {P1|**21gd**}: 4-(5-methyl-3-(4-phenyl-1*H*-1,2,3-triazol-1-yl)-1*H*-pyrazol-1-yl)benzonitrile; Formula: $C_{19}H_{14}N_6$; Molecular Mass: 326.3547; Exact Mass: 326.1280; EA: C, 69.92; H, 4.32; N, 25.75. Smiles: N#Cc1ccc(cc1)n1nc(cc1C)n1nnc(c1)c1ccccc1. InChIKey: QJHTUPAVPUUSNY-UHFFFAOYSA-N

In a vial, 4-(3-azido-5-methyl-1*H*-pyrazol-1-yl)benzonitrile (134 mg, 599 µmol, 1.00 equiv) was dissolved in THF (8.00 mL) and water (8.00 mL) (1:1) and copper;sulfate (19.1 mg, 120 µmol, 0.200 equiv), sodium ascorbate (119 mg, 599 µmol,

1.00 equiv) and ethynylbenzene (79.6 mg, 85.6 µL, 779 µmol, 1.30 equiv) were added. The reaction mixture was stirred at 50 °C for 16 hours. For work up the reaction mixture was diluted in water and extracted three times with methylene chloride. The combined organic phase was washed with water and brine, dried over Na_2SO_4 and filtered. As preparation for the column chromatography (dryload), Celite was added (0.3 g) and the reaction mixture with Celite were evaporated. The obtained crude product was purified *via* flash-chromatography (Interchim devices puriFLASH 5.125) on silica gel (PF-15SIHP-F0025) using cyclohexane/ethyl acetate 0% to 30% ethyl acetate in 15 column volumes (1 column volume = 63.8 mL; flow: 15 mL/min). The isolated product 4-(5-methyl-3-(4-phenyl-1*H*-1,2,3-triazol-1-yl)-1*H*-pyrazol-1-yl)benzonitrile (176 mg, 538 µmol) was obtained as a colorless solid in 90% yield.

R_f = 0.35 (cyclohexane/ethyl acetate 2:1). MP = 163.1–174.1 °C. ^{1}H NMR (400 MHz, Chloroform-d [7.26 ppm], ppm) δ = 8.45 (s, 1H), 7.91–7.89 (m, 2H), 7.82–7.80 (m, 2H), 7.69–7.67 (m, 2H), 7.46–7.43 (m, 2H), 7.38–7.34 (m, 1H), 6.82 (d, *J* = 0.6 Hz, 1H), 2.50 (s, 3H); ^{13}C NMR (100 MHz, Chloroform-d [77.16 ppm], ppm) δ = 148.0 (C_q), 147.7 (C_q), 142.6 (C_q), 142.0 (C_q), 133.5 (2C, CH), 130.2 (C_q), 129.0 (2C, CH), 128.6 (CH), 126.0 (2C, CH), 124.7 (2C, CH), 118.1 (C_q), 116.9 (CH), 111.7 (C_q), 100.2 (CH), 13.4 (CH_3); MS (FAB, 3-NBA), m/z (%): 327 (100) $[M+H]^+$, 298 (22), 154 (36), 136 (27), 107 (10), 91 (24). HRMS–FAB ($C_{19}H_{15}N_6$) *(m/z)*: $[M+H]^+$ Calcd 327.1353; Found 327.1354; IR (ATR, ṽ) = 3135 (w), 2227 (w), 1605 (w), 1584 (w), 1560 (w), 1551 (m), 1534 (m), 1510 (s), 1485 (s), 1445 (m), 1415 (w), 1383 (s), 1351 (m), 1315 (w), 1293 (w), 1232 (m), 1181 (w), 1130 (w), 1103 (w), 1061 (w), 1023 (s), 982 (w), 970 (w), 941 (m), 919 (w), 841 (vs), 783 (m), 772 (vs), 727 (w), 708 (m), 694 (vs), 683 (m), 650 (w), 626 (w), 584 (m), 572 (w), 544 (s), 520 (s), 469 (w), 435 (w) cm^{-1}.

Additional information on the chemical synthesis is available *via* Chemotion repository: https://doi.org/10.14272/reaction/SA-FUHFF-UHFFFADPSC-QJHTUPAVPU-UHFFFADPSC-NUHFF-NUHFF-NUHFF-ZZZ

Additional information on the analysis of the target compound is available *via* Chemotion repository: https://doi.org/10.14272/QJHTUPAVPUUSNY-UHFFFAOYSA-N.1

4-(3-(4-((1,3-Dioxoisoindolin-2-yl)methyl)-1*H*-1,2,3-triazol-1-yl)-5-methyl-1*H*-pyrazol-1-yl)benzonitrile (21gh)

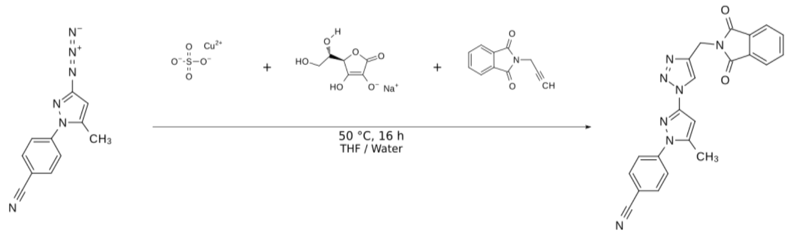

Name {P1|**21gh**}: 4-(3-(4-((1,3-dioxoisoindolin-2-yl)methyl)-1*H*-1,2,3-triazol-1-yl)-5-methyl-1*H*-pyrazol-1-yl)benzonitrile; Formula: $C_{22}H_{15}N_7O_2$; Smiles: N#Cc1ccc(cc1)n1nc(cc1C)n1nnc(c1)CN1C(=O)c2c(C1=O)cccc2. InChIKey: DWYBPGPEFGKMQS-UHFFFAOYSA-N

In a vial 4-(3-azido-5-methyl-1*H*-pyrazol-1-yl)benzonitrile (73.9 mg, 330 µmol, 1.00 equiv) was dissolved in THF (4.00 mL) and water (4.00 mL) (1:1) and

copper;sulfate (10.5 mg, 65.9 µmol, 0.20 equiv), sodium ascorbate (65.3 mg, 330 µmol, 1.00 equiv) and 2-prop-2-ynylisoindole-1,3-dione (79.3 mg, 428 µmol, 1.30 equiv) were added. The reaction mixture was stirred at 50 °C for 16 hours. For work up the reaction mixture was diluted in water and extracted three times with methylene chloride. The combined organic phase was washed with water and brine, dried over Na_2SO_4, filtered and the solvent was removed under reduced pressure. As preparation for the column chromatography (dryload), Celite was added (0.3 g) and the reaction mixture with additional Celite were evaporated. The obtained crude product was purified *via* flash-chromatography (Interchim devices puriFLASH 5.125) on silica gel (PF-15SIHP-F0012) using cyclohexane/ethyl acetate 0% to 50% ethyl acetate in 15 column volumes. The isolated product 4-(3-(4-((1,3-dioxoisoindolin-2-yl)methyl)-1*H*-1,2,3-triazol-1-yl)-5-methyl-1*H*-pyrazol-1-yl)benzonitrile (108.7 mg, 266 µmol) was obtained as a colorless solid in 81% yield.

R_f = 0.32 (cyclohexane/ethyl acetate 1:1). ^{1}H NMR (400 MHz, DMSO-d_6 [2.50 ppm], ppm) δ = 8.65 (s, 1H), 8.04–8.02 (m, 2H), 7.91–7.84 (m, 6H), 6.84 (s, 1H), 4.94 (s, 2H), 2.47 (s, 3H); ^{13}C NMR (100 MHz, DMSO-d_6 [39.5 ppm], ppm) δ = 167.4 (2C), 146.5, 143.3, 142.8, 142.0, 134.4 (2C), 133.6 (2C), 131.7, 124.7 (2C), 123.2 (2C), 120.9, 118.2 (2C), 110.4, 99.7, 32.8, 12.6; MS (FAB, 3-NBA), m/z (%): 410 (100) $[M+H]^+$, 235 (27), 219 (23), 191 (29), 175 (16), 167 (10), 154 (43), 147 (55), 136 (40), 105 (26), 91 (49). HRMS–FAB ($C_{22}H_{16}O_2N_7$) *(m/z)*: $[M+H]^+$ Calcd 410.1360; Found 410.1362; IR (ATR, ṽ) = 3143 (w), 3131 (w), 3106 (w), 2958 (w), 2924 (w), 2223 (m), 1768 (m), 1713 (vs), 1605 (s), 1588 (s), 1568 (w), 1555 (s), 1540 (s), 1510 (vs), 1483 (w), 1466 (w), 1435 (m), 1398 (vs), 1366 (vs), 1344 (vs), 1319 (s), 1309 (s), 1273 (m), 1224 (vs), 1210 (m), 1191 (s), 1111 (s), 1085 (s), 1043 (vs), 1023 (m), 1011 (m), 994 (m), 956 (vs), 942 (vs), 892 (w), 843 (s), 834 (vs), 813 (vs), 793 (m), 751 (s), 720 (s), 711 (vs), 687 (m), 625 (vs), 582 (w), 568 (m), 548 (vs), 530 (vs), 500 (w), 482 (w), 473 (w), 439 (w), 419 (m), 409 (m), 399 (m), 387 (w), 375 (m) cm^{-1}.

Additional information on the chemical synthesis is available *via* Chemotion repository: https://doi.org/10.14272/reaction/SA-FUHFF-UHFFFADPSC-DWYBPGPEFG-UHFFFADPSC-NUHFF-NUHFF-NUHFF-ZZZ

Additional information on the analysis of the target compound is available *via* Chemotion repository:
https://doi.org/10.14272/DWYBPGPEFGKMQS-UHFFFAOYSA-N.1

(1-(5-Methyl-1-(4-nitrophenyl)-1*H*-pyrazol-3-yl)-1*H*-1,2,3-triazol-4-yl)methyl acetate (**21hc**)

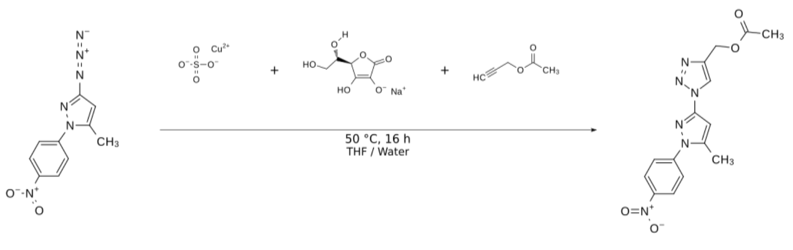

Name {P1|**21hc**}: (1-(5-methyl-1-(4-nitrophenyl)-1*H*-pyrazol-3-yl)-1*H*-1,2,3-triazol-4-yl)methyl acetate; Formula: $C_{15}H_{14}N_6O_4$; Smiles: CC(=O)OCc1nnn(c1)c1nn(c(c1)C)c1ccc(cc1)[N+](=O)[O-]. InChIKey: GFUNAXVZSJSISA-UHFFFAOYSA-N

In a vial, 3-azido-5-methyl-1-(4-nitrophenyl)-1*H*-pyrazole (59.5 mg, 244 µmol, 1.00 equiv) was dissolved in THF (4.00 mL) and water (4.00 mL) (1:1) and copper;sulfate (7.78 mg, 48.7 µmol, 0.200 equiv), sodium ascorbate (48.3 mg, 244 µmol, 1.00 equiv) and prop-2-ynyl acetate (31.1 mg, 31.4 µL, 317 µmol, 1.30 equiv) were added. The reaction mixture was stirred at 50 °C for 16 hours. For workup, the reaction mixture was diluted in water and extracted three times with methylene chloride. The combined organic phases were washed with water and brine, dried over Na_2SO_4, filtered and the solvent was removed under reduced pressure. As preparation for column chromatography (dry load), Celite was added (0.2 g) and the reaction mixture with Celite was evaporated. The obtained crude product was purified *via* flash-chromatography (Interchim devices puriFLASH 5.125) on silica gel (PF-15SIHP-F0012) using cyclohexane/ethyl acetate with 0% to 50% ethyl acetate in 15 column volumes. The isolated product (1-(5-methyl-1-(4-nitrophenyl)-1*H*-pyrazol-3-yl)-1*H*-1,2,3-triazol-4-yl)methyl acetate (43.0 mg, 126 µmol) was obtained as a yellow solid in 52% yield.

R_f = 0.17 (cyclohexane/ethyl acetate 2:1). MP = 217 – 253 °C. ^{1}H NMR (400 MHz, Chloroform-d [7.27 ppm], ppm) δ = 8.43–8.39 (m, 2H), 8.32 (s, 1H), 7.76–7.73 (m, 2H), 6.82 (d, *J* = 0.7 Hz, 1H), 5.31 (s, 2H), 2.55 (s, 3H), 2.11 (s, 3H). ^{13}C NMR (100 MHz, Chloroform-d [77.0 ppm], ppm) δ = 170.8 (C_q), 147.6 (C_q), 146.6 (C_q), 143.9 (C_q), 143.3 (C_q), 142.0 (C_q), 124.9 (CH, 2C), 124.4 (CH, 2C), 121.2 (CH), 100.4 (CH), 57.4 (CH_2), 20.8 (CH_3), 13.4 (CH_3); MS (FAB, 3-NBA), m/z (%): 343 (7) $[M+H]^+$, 219 (22), 203 (12), 191 (25), 175 (16), 159 (26), 154 (44), 147 (43), 136 (46), 115 (38), 105 (50), 91 (100). HRMS–FAB ($C_{15}H_{15}O_4N_6$) *(m/z)*: $[M+H]^+$ Calcd 343.1149; Found 343.1150; IR (ATR, ṽ) = 3138 (w), 3116 (w), 3091 (w), 2955 (w), 2921 (m), 2851 (w), 1721 (s), 1609 (w), 1594 (m), 1574 (m), 1561 (m), 1541 (m), 1511 (s), 1499 (vs), 1479 (s), 1451 (w), 1419 (w), 1380 (m), 1361 (m), 1333 (vs), 1312 (vs), 1254 (vs), 1230 (vs), 1190 (s), 1174 (s), 1132 (m), 1113 (s), 1081 (s), 1048 (s), 1023 (vs), 992 (s), 967 (vs), 941 (vs), 925 (vs), 850 (vs), 819 (s), 798 (vs), 766 (m), 751 (vs), 738 (m), 704 (m), 683 (s), 652 (w), 632 (w), 615 (s), 579 (m), 528 (m), 516 (s), 500 (m), 486 (m), 422 (m), 398 (w), 382 (w), 375 (w) cm^{-1}.

Additional information on the chemical synthesis is available *via* Chemotion repository: https://doi.org/10.14272/reaction/SA-FUHFF-UHFFFADPSC-GFUNAXVZSJ-UHFFFADPSC-NUHFF-NUHFF-NUHFF-ZZZ

Additional information on the analysis of the target compound is available *via* Chemotion repository:
https://doi.org/10.14272/GFUNAXVZSJSISA-UHFFFAOYSA-N.1

1-(5-Methyl-1-(4-nitrophenyl)-1*H*-pyrazol-3-yl)-4-phenyl-1*H*-1,2,3-triazole (21hd)

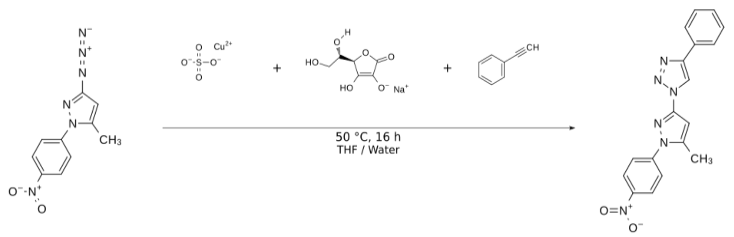

Name {P1|**21hd**}: 1-(5-methyl-1-(4-nitrophenyl)-1*H*-pyrazol-3-yl)-4-phenyl-1*H*-1,2,3-triazole; Formula: $C_{18}H_{14}N_6O_2$; Smiles: [O-][N+](=O)c1ccc(cc1)n1nc(cc1C)n1nnc(c1)c1ccccc1. InChIKey: UFCSIROIFJMCPW-UHFFFAOYSA-N

In a vial, 3-azido-5-methyl-1-(4-nitrophenyl)-1*H*-pyrazole (54.7 mg, 224 µmol, 1.00 equiv) was dissolved in THF (4.00 mL) and water (4.00 mL) (1:1) and copper;sulfate (7.15 mg, 44.8 µmol, 0.200 equiv), sodium ascorbate (44.4 mg, 224 µmol, 1.00 equiv) and ethynylbenzene (29.7 mg, 32.0 µL, 291 µmol, 1.30 equiv) were added. The reaction mixture was stirred at 50 °C for 16 hours. For workup, the reaction mixture was diluted in water and extracted three times with methylene chloride. The combined organic phases were washed with water and brine, dried over Na_2SO_4, filtered and the solvent was removed under reduced pressure. As preparation for column chromatography (dry load), Celite was added (0.2 g) and the reaction mixture with additional Celite was evaporated. The obtained crude product was purified *via* flash-chromatography (Interchim devices puriFLASH 5.125) on silica gel (PF-15SIHP-F0012) using cyclohexane/ethyl acetate with 0% to 30% ethyl acetate in 20 column volumes. The isolated product 1-(5-methyl-1-(4-nitrophenyl)-1*H*-pyrazol-3-yl)-4-phenyl-1*H*-1,2,3-triazole (68.8 mg, 199 µmol) was obtained as a colorless solid in 89% yield.

R_f = 0.41 (cyclohexane/ethyl acetate 4:1). MP = 200–202 °C. ^{1}H NMR (400 MHz, Chloroform-d [7.27 ppm], ppm) δ = 8.49 (s, 1H), 8.43–8.39 (m, 2H), 7.94–7.92 (m, 2H), 7.47 (dd, *J* = 8.4, 6.9 Hz, 2H), 7.47 (t, *J* = 7.5 Hz, 2H), 7.40–7.36 (m, 1H), 6.88 (s, 1H), 2.56 (s, 3H). ^{13}C NMR (100 MHz, Chloroform-d [77.0 ppm], ppm) δ = 148.0 (C_q), 147.8 (C_q), 146.6 (C_q), 144.0 (C_q), 142.0 (C_q), 130.0 (C_q), 128.9 (CH, 2C), 128.5 (CH), 125.9 (CH, 2C), 124.9 (CH, 2C), 124.4 (CH, 2C), 116.8 (CH), 100.4 (CH), 13.4 (CH_3); MS (FAB, 3-NBA), m/z (%): 347 (44) $[M+H]^+$, 307 (12), 289 (13), 219 (14), 165 (11), 154 (100), 147 (12), 136 (83), 128 (12), 115 (18), 107 (33), 91 (53). HRMS–FAB ($C_{18}H_{15}O_2N_6$) *(m/z)*: $[M+H]^+$ Calcd 347.1251; Found 347.1250; IR (ATR, ṽ) = 3146 (w), 1611 (w), 1594 (m), 1567 (m), 1537 (s), 1520 (vs), 1502 (vs), 1485 (s), 1448 (m), 1422 (w), 1381 (m), 1340 (vs), 1271 (w), 1228 (s), 1210 (w), 1183 (m), 1157 (w), 1133 (w), 1111 (m), 1075 (w), 1058 (m), 1021 (s), 1006 (m), 987 (m), 970 (m), 939 (s), 931 (m), 857 (vs), 822 (m), 815 (m), 796 (vs), 764 (vs), 751 (vs), 742 (s), 707 (s), 698 (vs), 690 (vs), 677 (s), 639 (m), 622 (w), 579 (w), 516 (vs), 501 (m), 473 (m), 416 (m), 404 (w), 392 (w), 378 (w) cm^{-1}.

Additional information on the chemical synthesis is available *via* Chemotion repository: https://doi.org/10.14272/reaction/SA-FUHFF-UHFFFADPSC-UFCSIROIFJ-UHFFFADPSC-NUHFF-NUHFF-NUHFF-ZZZ

Additional information on the analysis of the target compound is available *via* Chemotion repository:
https://doi.org/10.14272/UFCSIROIFJMCPW-UHFFFAOYSA-N.1

4-(4-Methoxyphenyl)-1-(5-methyl-1-(4-nitrophenyl)-1*H*-pyrazol-3-yl)-1*H*-1,2,3-triazole (**21he**)

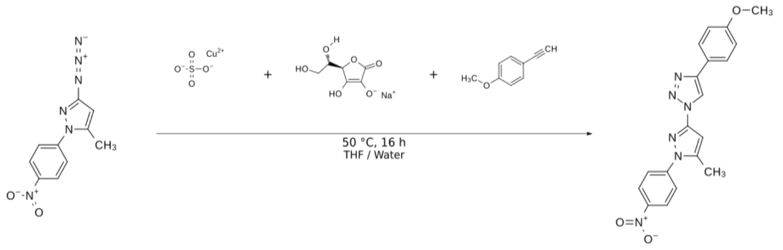

Name {P1|**21he**}: 4-(4-methoxyphenyl)-1-(5-methyl-1-(4-nitrophenyl)-1*H*-pyrazol-3-yl)-1*H*-1,2,3-triazole; Formula: $C_{19}H_{16}N_6O_3$; Smiles: COc1ccc(cc1)c1nnn(c1)c1cc(n(n1)c1ccc(cc1)[N+](=O)[O-])C. InChIKey: IZEQOYLPBRFYRC-UHFFFAOYSA-N

In a vial, 3-azido-5-methyl-1-(4-nitrophenyl)-1*H*-pyrazole (64.4 mg, 264 µmol, 1.00 equiv) was dissolved in THF (6.00 mL) and water (6.00 mL) (1:1) and copper;sulfate (8.42 mg, 52.7 µmol, 0.200 equiv), sodium ascorbate (52.2 mg, 264 µmol, 1.00 equiv) and 1-ethynyl-4-methoxybenzene (45.3 mg, 44.5 µL, 343 µmol, 1.30 equiv) were added. The reaction mixture was stirred at 50 °C for 16 hours. For workup, the reaction mixture was diluted in water and extracted three times with methylene chloride. The combined organic phases were washed with water and brine, dried over Na_2SO_4 and filtered. As preparation for column chromatography (dry load), Celite was added (0.2 g) and the reaction mixture with Celite was evaporated. The obtained crude product was purified *via* flash-chromatography (Interchim devices puriFLASH 5.125) on silica gel (PF-15SIHP-F0012) using cyclohexane/ethyl acetate with 0% to 50% ethyl acetate in 20 column volumes (1 column volume = 39.3 mL; flow: 15 mL/min). The isolated product 4-(4-methoxyphenyl)-1-(5-methyl-1-(4-nitrophenyl)-1*H*-pyrazol-3-yl)-1*H*-1,2,3-triazole (60.3 mg, 160 µmol) was obtained as a yellow solid in 61% yield.

R_f = 0.57 (cyclohexane/ethyl acetate 1:1). MP = 201.7–215.7 °C. ^{1}H NMR (400 MHz, DMSO-d_6 [2.50 ppm], ppm) δ = 9.09 (s, 1H), 8.43 (d, J = 8.9 Hz, 2H), 7.95 (dd, J = 21.9 Hz, J = 8.8 Hz, 4H), 7.04 (d, J = 8.7 Hz, 2H), 6.94 (s, 1H), 3.80 (s, 3H), 2.55 (s, 3H); ^{13}C NMR (100 MHz, DMSO-d_6 [39.5 ppm], ppm) δ = 159.4 (C_q), 147.0 (C_q), 146.9 (C_q), 146.1 (C_q), 143.6 (C_q), 143.1 (C_q), 126.9 (CH, 2C), 124.9 (CH, 2C), 124.7 (CH, 2C), 122.4 (C_q), 117.8 (CH), 114.4 (CH, 2C), 100.1 (CH), 55.2 (CH_3), 12.8 (CH_3); MS (FAB, 3-NBA), m/z (%): 377 (45) $[M+1]^+$, 348 (13), 219 (15), 191 (17), 159 (15), 154 (39), 147 (29), 136 (31), 131 (17), 121 (19), 115 (12), 105 (16), 91 (33). HRMS–FAB ($C_{19}H_{17}O_3N_6$) *(m/z)*: $[M+H]^+$ Calcd 377.1357; Found 377.1355; IR (ATR, ṽ) = 3159 (w), 3121 (w), 2914 (w), 2830 (w), 1619 (w), 1609 (w), 1591 (m), 1571 (s), 1543 (m), 1517 (s), 1494 (vs), 1438 (w), 1425 (w), 1377 (m), 1330 (vs), 1290 (m), 1248 (vs), 1231 (s), 1184 (m), 1173 (s), 1130 (w), 1109 (m), 1058 (w), 1031 (vs), 996 (m), 973 (w), 941 (m), 854 (s), 829 (s), 806 (w), 793 (m), 783 (m), 773 (s), 751 (s), 739 (w), 693 (w), 683 (w), 637 (w), 618 (m), 581 (w), 527 (m), 520 (m), 500 (w), 475 (m), 407 (w) cm^{-1}.

Additional information on the chemical synthesis is available *via* Chemotion repository: https://doi.org/10.14272/reaction/SA-FUHFF-UHFFFADPSC-IZEQOYLPBR-UHFFFADPSC-NUHFF-NUHFF-NUHFF-ZZZ

Additional information on the analysis of the target compound is available *via* Chemotion repository:
https://doi.org/10.14272/IZEQOYLPBRFYRC-UHFFFAOYSA-N.1

4-Butyl-1-(1-(2-chloro-4-nitrophenyl)-5-methyl-1*H*-pyrazol-3-yl)-1*H*-1,2,3-triazole (21ia)

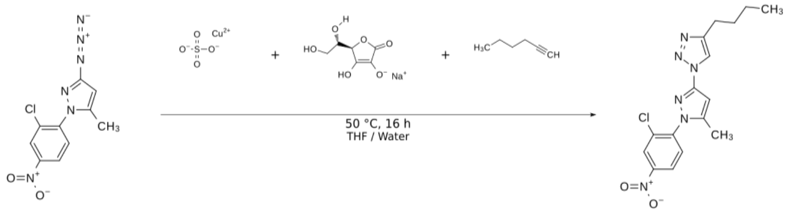

Name {P1|**21ia**}: 4-butyl-1-(1-(2-chloro-4-nitrophenyl)-5-methyl-1*H*-pyrazol-3-yl)-1*H*-1,2,3-triazole; Formula: $C_{16}H_{17}ClN_6O_2$; Smiles: CCCCc1nnn(c1)c1nn(c(c1)C)c1ccc(cc1Cl)[N+](=O)[O-]. InChIKey: JCVTZTZCOPFHML-UHFFFAOYSA-N

In a vial, 3-azido-1-(2-chloro-4-nitrophenyl)-5-methyl-1*H*-pyrazole (195 mg, 701 µmol, 1.00 equiv) was dissolved in THF (10.00 mL) and water (10.00 mL) (1:1) and copper;sulfate (22.4 mg, 140 µmol, 0.20 equiv), sodium ascorbate (139 mg, 701 µmol, 1.00 equiv) and hex-1-yne (74.8 mg, 104 µL, 911 µmol, 1.30 equiv) were added. The reaction mixture was stirred at 50 °C for 16 hours. For workup, the reaction mixture was diluted in water and extracted three times with methylene chloride. The combined organic phases were washed with water and brine, dried over Na_2SO_4, filtered and the solvent was removed under reduced pressure. As preparation for column chromatography (dry load), Celite was added (0.6 g) and the reaction mixture Celite were evaporated. The obtained crude product was purified *via* flash-chromatography (Interchim devices puriFLASH 5.125) on silica gel (PF-15SIHP-F0025) using cyclohexane/ethyl acetate with 0% to 35% ethyl acetate in 12 column volumes. The isolated product 4-butyl-1-(1-(2-chloro-4-nitrophenyl)-5-methyl-1*H*-pyrazol-3-yl)-1*H*-1,2,3-triazole (191 mg, 529 µmol) was obtained as a yellow oil in 75% yield.

R_f = 0.55 (cyclohexane/ethyl acetate 1:1). ^{1}H NMR (400 MHz, Chloroform-d [7.27 ppm], ppm) δ = 8.47 (d, *J* = 2.4 Hz, 1H), 8.31 (dd, *J* = 2.5 Hz, *J* = 8.6 Hz, 1H), 7.93 (s, 1H), 7.69 (d, *J* = 8.7 Hz, 1H), 6.78 (d, *J* = 0.6 Hz, 1H), 2.79 (t, *J* = 7.6 Hz, 2H), 2.27 (d, *J* = 0.5 Hz, 3H), 1.73–1.66 (m, 2H), 1.41 (h, *J* = 7.3 Hz, 2H), 0.93 (t, *J* = 7.3 Hz, 3H); ^{13}C NMR (100 MHz, Chloroform-d [77.0 ppm], ppm) δ = 148.7 (C_q), 148.4 (C_q), 148.2 (C_q), 143.4 (C_q), 141.7 (C_q), 133.9 (C_q), 130.8 (CH), 125.8 (CH), 122.8 (CH), 118.1 (CH), 98.3 (CH), 31.3 (CH_2), 25.2 (CH_2), 22.2 (CH_2), 13.8 (CH_3), 11.7 (CH_3). MS (FAB, 3-NBA), m/z (%): 361/363 (100/35) $[M+1]^+$, 345 (10), 333 (11), 315 (16), 133 (24). HRMS–FAB ($C_{16}H_{18}O_2N_6Cl$) *(m/z)*: $[M+H]^+$ Calcd 361.1174; Found 361.1176; IR (ATR, ṽ) = 3150 (vw), 3111 (vw), 3091 (vw), 2956 (w), 2929 (w), 2870 (w), 2860 (w), 1604 (w), 1587 (w), 1567 (s), 1550 (s), 1531 (vs), 1497 (vs), 1466 (m), 1443 (m), 1404 (w), 1367 (m), 1346 (vs), 1293 (m), 1217 (s), 1170 (w), 1136 (m), 1119 (m), 1072 (m), 1037 (vs), 1013 (s), 986 (w), 945 (s), 887 (s), 840 (m), 785 (s), 773 (vs), 748 (s), 724 (m), 700 (w), 688 (w), 664 (w), 645 (w), 575 (w), 554 (w), 534 (w), 484 (w), 462 (w), 450 (w), 425 (w), 411 (w), 387 (w), 377 (w) cm^{-1}.

Additional information on the chemical synthesis is available *via* Chemotion repository: https://doi.org/10.14272/reaction/SA-FUHFF-UHFFFADPSC-JCVTZTZCOP-UHFFFADPSC-NUHFF-NUHFF-NUHFF-ZZZ

Additional information on the analysis of the target compound is available *via* Chemotion repository:
https://doi.org/10.14272/JCVTZTZCOPFHML-UHFFFAOYSA-N.1

(1-(1-(2-Chloro-4-nitrophenyl)-5-methyl-1*H*-pyrazol-3-yl)-1*H*-1,2,3-triazol-4-yl)methyl acetate (21ic)

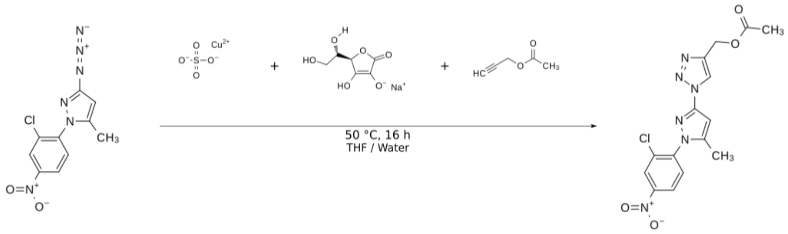

Name {P1|**21ic**}: (1-(1-(2-chloro-4-nitrophenyl)-5-methyl-1*H*-pyrazol-3-yl)-1*H*-1,2,3-triazol-4-yl)methyl acetate; Formula: $C_{15}H_{13}ClN_6O_4$; Smiles: CC(=O)OCc1nnn(c1)c1nn(c(c1)C)c1ccc(cc1Cl)[N+](=O)[O-]. InChIKey: QIRBWRVTFHDFHS-UHFFFAOYSA-N

In a vial, 3-azido-1-(2-chloro-4-nitrophenyl)-5-methyl-1H-pyrazole (50.0 mg, 179 µmol, 1.00 equiv) was dissolved in THF (4.00 mL) and water (4.00 mL) (1:1) and copper;sulfate (5.73 mg, 35.9 µmol, 0.20 equiv), sodium ascorbate (35.5 mg, 179 µmol, 1.00 equiv) and prop-2-ynyl acetate (22.9 mg, 23.1 µL, 233 µmol, 1.30 equiv) were added. The reaction mixture was stirred at 50 °C for 16 hours, then it was diluted with water and extracted three times with methylene chloride. The combined organic phase was washed with water and brine, dried over Na_2SO_4 and filtered. As preparation for the column chromatography (dryload), Celite was added (0.2 g) and the reaction mixture with Celite was evaporated. The obtained crude product was purified *via* flash-chromatography (Interchim devices puriFLASH 5.125) on silica gel (PF-15SIHP-F0012) using cyclohexane/ethyl acetate 0% to 35% ethyl acetate in 12 column volumes (1 column volume = 39.3 mL; flow: 15 mL/min). The isolated product (1-(1-(2-chloro-4-nitrophenyl)-5-methyl-1*H*-pyrazol-3-yl)-1*H*-1,2,3-triazol-4-yl)methyl acetate (54.1 mg, 144 µmol) was obtained as a colorless solid in 80% yield.

R_f = 0.50 (cyclohexane/ethyl acetate 1:1). MP = 113.3–114.3 °C. ^{1}H NMR (400 MHz, Chloroform-d [7.27 ppm], ppm) δ = 8.46 (d, *J* = 2.4 Hz, 1H), 8.31 (dd, *J* = 2.4 Hz, *J* = 8.7 Hz, 1H), 8.23 (s, 1H), 7.68 (d, *J* = 8.7 Hz, 1H), 6.77 (d, *J* = 0.7 Hz, 1H), 5.26 (s, 2H), 2.27 (d, *J* = 0.6 Hz, 3H), 2.07 (s, 3H); ^{13}C NMR (100 MHz, Chloroform-d [77.0 ppm], ppm) δ = 170.7 (C_q), 148.4 (C_q), 147.7 (C_q), 143.5 (C_q), 143.1 (C_q), 141.5 (C_q), 133.8 (C_q), 130.7 (CH), 125.8 (CH), 122.8 (CH), 121.2 (CH), 98.3 (CH), 57.4 (CH_2), 20.8 (CH_3), 11.7 (CH_3); MS (FAB, 3-NBA), m/z (%): 377/379 (100/33) $[M+H]^+$, 307 (11), 289 (47), 243 (17), 191 (11), 154 (40), 147 (14), 136 (33), 131 (13), 121 (10), 109 (19), 105 (13), 95 (29). HRMS–FAB ($C_{15}H_{14}O_4N_6Cl$) *(m/z)*: $[M+H]^+$ Calcd 377.0760; Found 377.0761; IR (ATR, ṽ) = 3149 (vw), 3108 (vw), 3091 (vw), 1740 (vs), 1604 (w), 1587 (w), 1558 (s), 1530 (vs), 1497 (vs), 1468 (w), 1442 (w), 1404 (w), 1346 (vs), 1289 (w), 1224 (vs), 1174 (w), 1137 (w), 1119 (w), 1072 (m), 1028 (vs), 1013 (vs), 967 (m), 945 (s), 911 (m), 887 (vs), 837 (m), 773 (vs), 748 (vs), 724 (s), 690 (m), 667 (w), 637 (w),

620 (w), 603 (w), 574 (w), 555 (w), 533 (m), 480 (m), 452 (w), 409 (w), 399 (w), 388 (w), 375 (w) cm^{-1}.

Additional information on the chemical synthesis is available *via* Chemotion repository: https://doi.org/10.14272/reaction/SA-FUHFF-UHFFFADPSC-QIRBWRVTFH-UHFFFADPSC-NUHFF-NUHFF-NUHFF-ZZZ

Additional information on the analysis of the target compound is available *via* Chemotion repository: https://doi.org/10.14272/QIRBWRVTFHDFHS-UHFFFAOYSA-N.1

1-(1-(2-Chloro-4-nitrophenyl)-5-methyl-1*H*-pyrazol-3-yl)-4-phenyl-1*H*-1,2,3-triazole (21id)

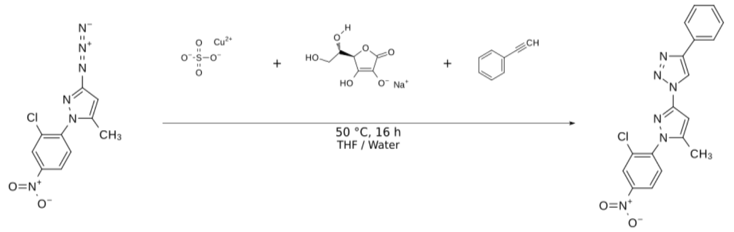

Name {P1|**21id**}: 1-(1-(2-chloro-4-nitrophenyl)-5-methyl-1*H*-pyrazol-3-yl)-4-phenyl-1*H*-1,2,3-triazole; Formula: $C_{18}H_{13}ClN_6O_2$; Smiles: Clc1cc(ccc1n1nc(cc1C)n1nnc(c1)c1ccccc1)[N+](=O)[O-]. InChIKey: NASFABBFAHTRIZ-UHFFFAOYSA-N

In a vial, 3-azido-1-(2-chloro-4-nitrophenyl)-5-methyl-1*H*-pyrazole (50.0 mg, 179 µmol, 1.00 equiv) was dissolved in THF (4.00 mL) and water (4.00 mL) (1:1) and copper;sulfate (5.73 mg, 35.9 µmol, 0.200 equiv), sodium ascorbate (35.5 mg, 179 µmol, 1.00 equiv) and ethynylbenzene (23.8 mg, 25.6 µL, 233 µmol, 1.30 equiv) were added. The reaction mixture was stirred at 50 °C for 16 hours. For workup, the reaction mixture was diluted in water and extracted three times with methylene chloride. The combined organic phases were washed with water and brine, dried over Na_2SO_4, filtered and the solvent was removed under reduced pressure. As preparation for column chromatography (dry load), Celite was added (0.2 g) and the reaction mixture with Celite was evaporated. The obtained crude product was purified *via* flash-chromatography (Interchim devices puriFLASH 5.125) on silica gel (PF-15SIHP-F0012) using cyclohexane/ethyl acetate with 0% to 25% ethyl acetate in 12 column volumes. The isolated product 1-(1-(2-chloro-4-nitrophenyl)-5-methyl-1*H*-pyrazol-3-yl)-4-phenyl-1*H*-1,2,3-triazole (56.1 mg, 147 µmol) was obtained as yellow oil in 82% yield.

R_f = 0.76 (cyclohexane/ethyl acetate 1:1). ^{1}H NMR (400 MHz, Chloroform-d [7.27 ppm], ppm) δ = 8.47 (d, *J* = 2.4 Hz, 1H), 8.43 (s, 1H), 8.31 (dd, *J* = 2.4 Hz, *J* = 8.7 Hz, 1H), 7.90–7.87 (m, 2H), 7.71 (d, *J* = 8.7 Hz, 1H), 7.46–7.42 (m, 2H), 7.37–7.33 (m, 1H), 6.83 (d, *J* = 0.6 Hz, 1H), 2.27 (d, *J* = 0.5 Hz, 3H). ^{13}C NMR (100 MHz, Chloroform-d [77.0 ppm], ppm) δ = 148.4 (C_q), 147.9 (C_q), 147.8 (C_q), 143.5 (C_q), 141.5 (C_q), 133.8 (CH), 130.7 (C_q), 130.0 (C_q), 128.8 (CH, 2C), 128.4 (CH), 125.8 (CH), 125.8 (CH, 2C), 122.8 (CH), 116.8 (CH), 98.3 (CH), 11.7 (CH_3); MS (FAB, 3-NBA), m/z (%): 381/383 (55/19) $[M+1]^+$, 154 (11), 136 (13), 133 (100), 91 (25). HRMS–FAB ($C_{18}H_{14}O_2N_6Cl$) *(m/z)*: $[M+H]^+$ Calcd 381.0861; Found 381.0860; IR (ATR, ṽ) = 3155 (m), 3109 (w), 3072 (w), 3043 (w), 2953 (w), 2924 (w), 2870 (w), 2853 (w), 1599 (vw), 1584 (w), 1567

(s), 1537 (s), 1520 (vs), 1493 (vs), 1480 (vs), 1465 (m), 1446 (s), 1374 (m), 1350 (vs), 1290 (m), 1234 (s), 1177 (w), 1163 (w), 1136 (w), 1122 (m), 1101 (w), 1074 (m), 1061 (m), 1034 (s), 1026 (s), 1011 (s), 987 (m), 970 (m), 941 (s), 912 (m), 902 (s), 887 (s), 840 (s), 817 (m), 789 (vs), 772 (s), 762 (vs), 748 (vs), 722 (vs), 705 (s), 691 (vs), 681 (vs), 670 (s), 652 (m), 633 (m), 619 (w), 601 (w), 582 (m), 554 (m), 523 (m), 507 (m), 494 (s), 460 (m), 448 (m), 424 (m), 415 (w), 404 (w), 395 (m), 375 (w) cm^{-1}.

Additional information on the chemical synthesis is available *via* Chemotion repository: https://doi.org/10.14272/reaction/SA-FUHFF-UHFFFADPSC-NASFABBFAH-UHFFFADPSC-NUHFF-NUHFF-NUHFF-ZZZ

Additional information on the analysis of the target compound is available *via* Chemotion repository:
https://doi.org/10.14272/NASFABBFAHTRIZ-UHFFFAOYSA-N.1

Ethyl 3-(4-butyl-1*H*-1,2,3-triazol-1-yl)-1*H*-pyrazole-4-carboxylate (**21ja**)

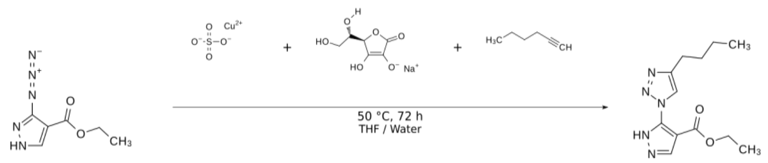

Name {P1|**21ja**}: ethyl 3-(4-butyl-1*H*-1,2,3-triazol-1-yl)-1*H*-pyrazole-4-carboxylate; Formula: $C_{12}H_{17}N_5O_2$; Smiles: CCCCc1nnn(c1)c1n[nH]cc1C(=O)OCC. InChIKey: CDDXWUAJGLDUKH-UHFFFAOYSA-N

In a vial, ethyl 5-azido-1*H*-pyrazole-4-carboxylate (50.0 mg, 276 µmol, 1.00 equiv) was dissolved in THF (6.00 mL) and water (6.00 mL) (1:1) and copper;sulfate (8.81 mg, 55.2 µmol, 0.20 equiv), sodium ascorbate (54.7 mg, 276 µmol, 1.00 equiv) and hex-1-yne (29.5 mg, 40.9 µL, 359 µmol, 1.30 equiv) were added. The reaction mixture was stirred at 50 °C for 72 hours. For work up the reaction mixture was diluted in water and extracted three times with methylene chloride. The combined organic phase was washed with water and brine, dried over Na_2SO_4 and filtered. As preparation for the column chromatography (dryload), Celite was added (0.2 g) and the reaction mixture with Celite were evaporated. The obtained crude product was purified *via* flash-chromatography (Biotage devices) on silica gel (PF-15SIHP-F0012) using dichloromethane/methanol 0% to 10% methanol in 15 column volumes (1 column volume = 39.3 mL; flow: 15 mL/min). The isolated product ethyl 3-(4-butyl-1*H*-1,2,3-triazol-1-yl)-1*H*-pyrazole-4-carboxylate (30.1 mg, 114 µmol) was obtained as light green solid in 41% yield.

R_f = 0.14 (CH_2Cl_2/MeOH 30:1). MP = 122.7–150.2 °C. ^{1}H NMR (400 MHz, DMSO-d_6 [2.50 ppm], ppm) δ = 13.90 (s, 1H), 8.56 (s, 1H), 8.21 (s, 1H), 4.10 (q, *J* = 7.1 Hz, 2H), 2.70 (t, *J* = 7.5 Hz, 2H), 1.64 (quint, *J* = 7.6 Hz, 2H), 1.37 (h, *J* = 7.0 Hz, 2H), 1.11 (t, *J* = 7.1 Hz, 3H), 0.92 (t, *J* = 7.3 Hz, 3H); ^{13}C NMR (100 MHz, DMSO-d_6 [39.5 ppm], ppm) δ = 160.8, 146.3, 144.7, 134.8, 124.2, 107.3, 59.9, 31.0, 24.4, 21.6, 13.9, 13.6; MS (FAB, 3-NBA), m/z (%): 264 (100) $[M+H]^+$, 175 (10), 161 (18), 154 (23), 136 (22), 109 (15), 95 (26). HRMS–FAB ($C_{12}H_{18}O_2N_5$) *(m/z)*: $[M+H]^+$ Calcd 264.1455; Found 264.1453; IR (ATR, ṽ) = 3157 (m), 2955 (m), 2929 (m), 2871 (w), 2860 (w), 1711 (vs),

1558 (w), 1523 (vs), 1477 (w), 1466 (w), 1446 (w), 1408 (w), 1394 (w), 1373 (m), 1334 (w), 1306 (m), 1254 (m), 1222 (m), 1211 (m), 1184 (w), 1171 (w), 1111 (vs), 1085 (s), 1038 (vs), 1020 (s), 983 (m), 941 (w), 902 (w), 860 (m), 829 (s), 772 (vs), 732 (w), 694 (w), 646 (w), 619 (w), 578 (s), 511 (w), 455 (w), 408 (w), 382 (w) cm^{-1}.

Additional information on the chemical synthesis is available *via* Chemotion repository: https://doi.org/10.14272/reaction/SA-FUHFF-UHFFFADPSC-CDDXWUAJGL-UHFFFADPSC-NUHFF-NUHFF-NUHFF-ZZZ

Additional information on the analysis of the target compound is available *via* Chemotion repository: https://doi.org/10.14272/CDDXWUAJGLDUKH-UHFFFAOYSA-N.1

Ethyl 3-(4-(acetoxymethyl)-1*H*-1,2,3-triazol-1-yl)-1*H*-pyrazole-4-carboxylate (21jc)

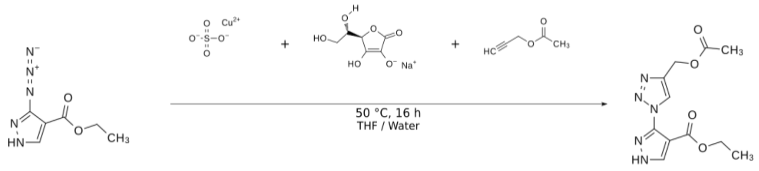

Name {P1|**21jc**}: ethyl 3-(4-(acetoxymethyl)-1*H*-1,2,3-triazol-1-yl)-1*H*-pyrazole-4-carboxylate; Formula: $C_{11}H_{13}N_5O_4$; Smiles: CCOC(=O)c1c[nH]nc1n1nnc(c1)COC(=O)C. InChIKey: RJHHGQMRVYAAGU-UHFFFAOYSA-N

In a vial, ethyl 5-azido-1H-pyrazole-4-carboxylate (173 mg, 955 µmol, 1.00 equiv) was dissolved in THF (5.00 mL) and water (5.00 mL) (1:1) and copper sulfate (30.5 mg, 191 µmol, 0.20 equiv), sodium ascorbate (189 mg, 955 µmol, 1.00 equiv) and prop-2-ynyl acetate (122 mg, 123 µL, 1.24 mmol, 1.30 equiv) were added. The reaction mixture was stirred at 50 °C for 16 hours. For workup, the reaction mixture was diluted in water and extracted three times with methylene chloride. The combined organic phases were washed with water and brine, dried over Na_2SO_4 and filtered. As preparation for column chromatography (dry load), Celite was added (0.6 g) and the reaction mixture with Celite was evaporated. The obtained crude product was purified *via* flash-chromatography (Interchim devices puriFLASH 5.125) on silica gel (PF-15SIHP-F0025) using cyclohexane/ethyl acetate with 0% to 100% ethyl acetate in 20 column volumes (1 column volume = 63.8 mL; flow: 15 mL/min). The isolated product ethyl 3-(4-(acetoxymethyl)-1*H*-1,2,3-triazol-1-yl)-1*H*-pyrazole-4-carboxylate (134 mg, 479 µmol) was obtained as a colorless solid in 50% yield.

R_f = 0.28 (cyclohexane/ethyl acetate 1:2). MP = 126.1–139.8 °C. ^{1}H NMR (400 MHz, DMSO-d_6 [2.50 ppm], ppm) δ = 13.97 (s, 1H), 8.59 (s, 1H), 8.55 (s, 1H), 5.21 (s, 2H), 4.10 (q, *J* = 7.1 Hz, 2H), 2.06 (s, 3H), 1.11 (t, *J* = 7.1 Hz, 3H); ^{13}C NMR (100 MHz, DMSO-d_6 [39.5 ppm], ppm) δ = 170.0 (C_q), 160.7 (C_q), 144.3 (C_q), 141.4 (C_q), 134.9 (CH), 127.2 (CH), 107.5 (C_q), 60.0 (CH_2), 56.8 (CH_2), 20.6 (CH_3), 13.8 (CH_3); MS (EI, 70 eV, 140 °C), m/z (%): 279 (1) $[M]^+$, 192 (74), 181 (13), 164 (100), 155 (15), 146 (15), 135 (39), 110 (35), 55 (25). HRMS–EI ($C_{11}H_{13}O_4N_5$) *(m/z)*: $[M]^+$ Calcd 279.0962; Found 279.0961; IR (ATR, ṽ) = 3150 (m), 2941 (w), 2799 (w), 1740 (s), 1717 (vs), 1530 (s),

1466 (w), 1446 (w), 1409 (w), 1383 (m), 1363 (m), 1337 (m), 1306 (m), 1283 (w), 1238 (vs), 1225 (vs), 1183 (m), 1113 (vs), 1085 (s), 1045 (vs), 1023 (vs), 999 (s), 980 (s), 976 (s), 956 (s), 943 (m), 925 (w), 832 (vs), 769 (vs), 693 (w), 662 (w), 637 (w), 620 (w), 609 (w), 575 (s), 565 (w), 518 (w), 477 (w), 456 (w), 398 (w), 391 (w), 384 (w) cm^{-1}.

Additional information on the chemical synthesis is available *via* Chemotion repository: https://doi.org/10.14272/reaction/SA-FUHFF-UHFFFADPSC-RJHHGQMRVY-UHFFFADPSC-NUHFF-NUHFF-NUHFF-ZZZ

Additional information on the analysis of the target compound is available *via* Chemotion repository:
https://doi.org/10.14272/RJHHGQMRVYAAGU-UHFFFAOYSA-N.1

Ethyl 3-(4-phenyl-1*H*-1,2,3-triazol-1-yl)-1*H*-pyrazole-4-carboxylate (21jd)

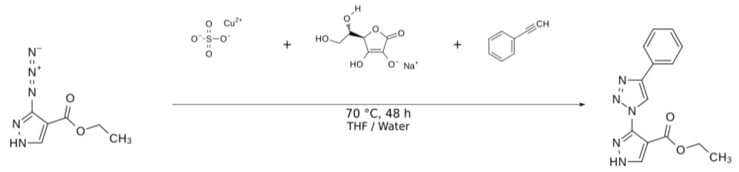

Name {P1|**21jd**}: ethyl 3-(4-phenyl-1*H*-1,2,3-triazol-1-yl)-1*H*-pyrazole-4-carboxylate; Formula: $C_{14}H_{13}N_5O_2$; Smiles: CCOC(=O)c1c[nH]nc1n1nnc(c1)c1ccccc1. InChIKey: JZSBUPSAIWVYFE-UHFFFAOYSA-N

In a vial, ethyl 5-azido-1*H*-pyrazole-4-carboxylate (50.0 mg, 276 µmol, 1.00 equiv) was dissolved in THF (6.00 mL) and water (6.00 mL) (1:1) and copper;sulfate (8.81 mg, 55.2 µmol, 0.20 equiv), sodium ascorbate (54.7 mg, 276 µmol, 1.00 equiv) and ethynylbenzene (36.6 mg, 39.4 µL, 359 µmol, 1.30 equiv) were added. The reaction mixture was stirred at 70 °C for 48 hours. For work, up the reaction mixture was diluted in water and extracted three times with methylene chloride. The combined organic phases were washed with water and brine, dried over Na_2SO_4 and filtered. As preparation for column chromatography (dry load), Celite was added (0.2 g) and the reaction mixture with additional Celite was evaporated. The obtained crude product was purified *via* flash-chromatography (Interchim devices puriFLASH 5.125) on silica gel (PF-15SIHP-F0012) using cyclohexane/ethyl acetate with 0% to 70% ethyl acetate in 25 column volumes (1 column volume = 39.3 mL; flow: 15 mL/min). The isolated product ethyl 3-(4-phenyl-1*H*-1,2,3-triazol-1-yl)-1*H*-pyrazole-4-carboxylate (22.0 mg, 77.7 µmol) was obtained as a colorless solid in 28% yield.

R_f = 0.23 (cyclohexane/ethyl acetate 1:1). MP = 172.2–188 °C. ^{1}H NMR (400 MHz, DMSO-d_6 [2.50 ppm], ppm) δ = 14.02 (s, 1H), 9.00 (s, 1H), 8.63 (s, 1H), 7.95–7.93 (m, 2H), 7.49 (t, J = 7.7 Hz, 2H), 7.40–7.36 (m, 1H), 4.12 (q, J = 7.1 Hz, 2H), 1.10 (t, J = 7.1 Hz, 3H); ^{13}C NMR (100 MHz, DMSO-d_6 [39.5 ppm], ppm) δ = 160.8 (C_q), 145.9 (C_q), 144.5 (C_q), 135.0 (CH), 130.2 (C_q), 129.0 (CH, 2C), 128.1 (CH), 125.3 (CH, 2C), 123.8 (CH), 107.5 (C_q), 60.0 (CH_2), 13.8 (CH_3); MS (EI, 70 eV, 160 °C), m/z (%): 283 (5) $[M]^+$, 255 (93), 227 (93), 209 (100), 198 (40), 182 (15), 169 (13), 155 (14), 136 (21), 127 (14), 118 (51), 110 (25), 102 (40), 89 (31), 77 (14), 69 (26). HRMS–EI ($C_{14}H_{13}O_2N_5$) *(m/z)*: $[M]^+$ Calcd 283.1064; Found 283.1062; IR (ATR, ṽ) = 3150 (s),

3060 (w), 3034 (w), 2941 (m), 2851 (w), 2800 (w), 2741 (w), 2681 (w), 1711 (vs), 1523 (vs), 1477 (m), 1466 (m), 1449 (m), 1401 (m), 1373 (m), 1341 (m), 1307 (s), 1281 (w), 1251 (s), 1218 (s), 1183 (m), 1171 (w), 1119 (vs), 1086 (vs), 1077 (vs), 1030 (s), 1018 (vs), 979 (s), 967 (m), 939 (m), 908 (w), 830 (s), 772 (vs), 759 (vs), 687 (vs), 647 (w), 618 (m), 577 (vs), 545 (m), 504 (m), 466 (w), 426 (w), 401 (w), 377 (m) cm^{-1}.

Additional information on the chemical synthesis is available *via* Chemotion repository: https://doi.org/10.14272/reaction/SA-FUHFF-UHFFFADPSC-JZSBUPSAIW-UHFFFADPSC-NUHFF-NUHFF-NUHFF-ZZZ.1

Additional information on the analysis of the target compound is available *via* Chemotion repository:
https://doi.org/10.14272/JZSBUPSAIWVYFE-UHFFFAOYSA-N.2

This compound has been previously synthesized in literature. [8]

Ethyl 3-(4-((1,3-dioxoisoindolin-2-yl)methyl)-1*H*-1,2,3-triazol-1-yl)-1*H*-pyrazole-4-carboxylate (21jh)

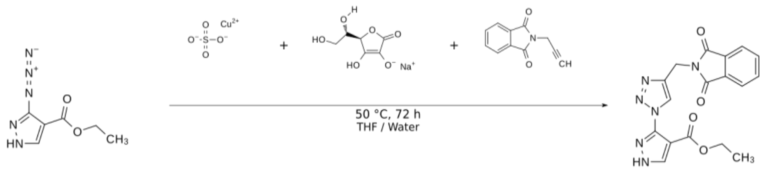

Name {P1|**21jh**}: ethyl 3-(4-((1,3-dioxoisoindolin-2-yl)methyl)-1*H*-1,2,3-triazol-1-yl)-1*H*-pyrazole-4-carboxylate; Formula: $C_{17}H_{14}N_6O_4$; Smiles: CCOC(=O)c1c[nH]nc1n1nnc(c1)CN1C(=O)c2c(C1=O)cccc2. InChIKey: GMOYQWJQIYZFIE-UHFFFAOYSA-N

In a vial, ethyl 5-azido-1*H*-pyrazole-4-carboxylate (50.0 mg, 276 µmol, 1.00 equiv) was dissolved in THF (6.00 mL) and water (6.00 mL) (1:1) and copper;sulfate (8.81 mg, 55.2 µmol, 0.20 equiv), sodium ascorbate (54.7 mg, 276 µmol, 1.00 equiv) and 2-prop-2-ynylisoindole-1,3-dione (66.4 mg, 359 µmol, 1.30 equiv) were added. The reaction mixture was stirred at 50 °C for 72 hours. For workup, the reaction mixture was diluted in water and extracted three times with methylene chloride. The combined organic phases were washed with water and brine, dried over Na_2SO_4, filtered and the solvent was removed under reduced pressure. As preparation for column chromatography (dry load), Celite was added (0.2 g) and the reaction mixture with Celite was evaporated. The obtained crude product was purified *via* flash-chromatography (Biotage Isolera) on silica gel (PF-15SIHP-F0012) using dichloromethane/methanol with 0% to 10% methanol in 15 column volumes. The isolated product ethyl 3-(4-((1,3-dioxoisoindolin-2-yl)methyl)-1*H*-1,2,3-triazol-1-yl)-1*H*-pyrazole-4-carboxylate (26.6 mg, 72.6 µmol) was obtained as light green solid in 26% yield.

R_f = 0.23 (CH_2Cl_2/MeOH 30:1). MP = 200.9–225 °C. ^{1}H NMR (400 MHz, DMSO-d_6 [2.50 ppm], ppm) δ = 13.94 (s, 1H), 8.56 (s, 1H), 8.48 (s, 1H), 7.93–7.84 (m, 4H), 4.94 (s, 2H), 4.05 (q, *J* = 7.1 Hz, 2H), 1.04 (t, *J* = 7.1 Hz, 3H); ^{13}C NMR (100 MHz, DMSO-d_6 [39.5 ppm], ppm) δ = 167.3 (C_q, 2C), 160.7 (C_q), 144.3 (C_q), 141.9 (C_q), 134.9 (CH), 134.5 (CH, 2C), 131.6 (C_q, 2C), 125.6 (CH), 123.2 (CH, 2C), 107.5 (C_q), 60.0 (CH_2), 32.7 (CH_2), 13.7 (CH_3); MS (FAB, 3-NBA), m/z (%): 367 (53) $[M+H]^+$, 333 (33), 305

(30), 289 (11), 261 (21),245 (23), 231 (32), 189 (26), 175 (62), 161 (100), 154 (87), 147 (44), 136 (85), 119 (30), 105 (48), 91 (79). HRMS–FAB ($C_{17}H_{15}O_4N_6$) *(m/z)*: $[M+H]^+$ Calcd 367.1149; Found 367.1149; IR (ATR, ṽ) = 3272 (w), 3138 (w), 3094 (w), 2993 (vw), 2961 (w), 2914 (w), 1765 (w), 1704 (vs), 1613 (w), 1564 (m), 1551 (m), 1538 (s), 1492 (w), 1480 (w), 1465 (m), 1422 (s), 1397 (vs), 1380 (s), 1370 (s), 1353 (m), 1339 (s), 1316 (s), 1306 (m), 1288 (w), 1239 (vs), 1179 (s), 1162 (w), 1122 (vs), 1101 (vs), 1086 (s), 1077 (m), 1043 (vs), 1021 (s), 1010 (s), 987 (s), 933 (vs), 853 (w), 833 (m), 800 (w), 768 (vs), 711 (vs), 654 (vs), 612 (s), 572 (s), 551 (m), 531 (vs), 490 (m), 415 (m), 387 (w) cm^{-1}.

Additional information on the chemical synthesis is available *via* Chemotion repository: https://doi.org/10.14272/reaction/SA-FUHFF-UHFFFADPSC-GMOYQWJQIY-UHFFFADPSC-NUHFF-NUHFF-NUHFF-ZZZ

Additional information on the analysis of the target compound is available *via* Chemotion repository:
https://doi.org/10.14272/GMOYQWJQIYZFIE-UHFFFAOYSA-N.1

Ethyl 1-benzyl-3-(4-phenyl-1*H*-1,2,3-triazol-1-yl)-1*H*-pyrazole-4-carboxylate (21kd)

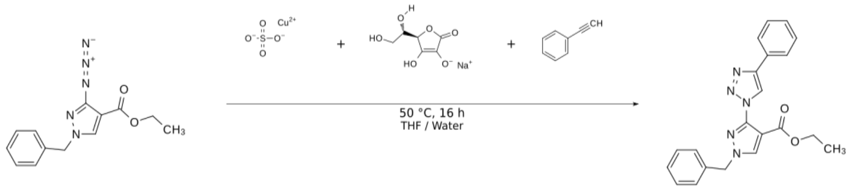

Name {P1|**21kd**}: ethyl 1-benzyl-3-(4-phenyl-1*H*-1,2,3-triazol-1-yl)-1*H*-pyrazole-4-carboxylate; Formula: $C_{21}H_{19}N_5O_2$; Smiles: CCOC(=O)c1cn(nc1n1nnc(c1)c1ccccc1)Cc1ccccc1. InChIKey: BFHMQIVAONQIIQ-UHFFFAOYSA-N

In a vial, 3-azido-1-benzyl-pyrazole-4-carboxylic acid ethyl ester (43.8 mg, 161 µmol, 1.00 equiv) was dissolved in THF (5.00 mL) and water (5.00 mL) (1:1) and copper sulfate (5.15 mg, 32.3 µmol, 0.20 equiv), sodium ascorbate (32.0 mg, 161 µmol, 1.00 equiv) and ethynylbenzene (21.4 mg, 23.1 µL, 210 µmol, 1.30 equiv) were added. The reaction mixture was stirred at 50 °C for 16 hours. For work up the reaction mixture was diluted in water and extracted three times with methylene chloride. The combined organic phase was washed with water and brine, dried over Na_2SO_4 and filtered. As preparation for the column chromatography (dryload), Celite was added (0.2 g) and the reaction mixture with Celite were evaporated. The obtained crude product was purified *via* flash-chromatography (Interchim devices puriFLASH 4125) on silica gel (PF-15SIHP-F0012) using cyclohexane/ethyl acetate 0% to 30% ethyl acetate in 20 column volumes (1 column volume = 39.3 mL; flow: 15 mL/min). The isolated product ethyl 1-benzyl-3-(4-phenyl-1*H*-1,2,3-triazol-1-yl)-1*H*-pyrazole-4-carboxylate (52.1 mg, 140 µmol) was obtained as a colorless solid in 86% yield.

R_f = 0.45 (cyclohexane/ethyl acetate 2:1). MP = 114–115 °C. ^1H NMR (400 MHz, Chloroform-d [7.27 ppm], ppm) δ = 8.09 (s, 1H), 7.93 (s, 1H), 7.85–7.82 (m, 2H), 7.49–7.44 (m, 2H), 7.41–7.37 (m, 1H), 7.26–7.22 (m, 3H), 7.11–7.07 (m, 2H), 5.40 (s, 2H),

4.19 (q, J = 7.1 Hz, 2H), 1.19 (t, J = 7.2 Hz, 3H); ^{13}C NMR (100 MHz, Chloroform-d [77.0 ppm], ppm) δ = 160.9, 147.4, 141.0, 135.5, 134.4, 129.5, 128.9 (2C), 128.7 (2C), 128.6, 128.4, 127.8 (2C), 125.9 (2C), 123.3, 109.5, 60.7, 54.3, 14.0; MS (EI, 70 eV, 120 °C), m/z (%): 375 (0.11) [M]$^+$, 345 (100), 317 (37), 299 (12), 288 (15), 198 (12), 91 (61). HRMS–EI ($C_{21}H_{19}O_2N_5$) *(m/z)*: [M]$^+$ Calcd 373.1533; Found 373.1534; IR (ATR, ṽ) = 3149 (w), 3121 (w), 3034 (w), 2992 (w), 2961 (w), 2942 (w), 2924 (w), 2902 (w), 1710 (vs), 1584 (vs), 1558 (w), 1510 (vs), 1482 (m), 1476 (m), 1456 (m), 1443 (m), 1422 (w), 1401 (w), 1387 (m), 1377 (m), 1363 (w), 1347 (m), 1281 (m), 1255 (vs), 1215 (vs), 1186 (vs), 1160 (m), 1120 (s), 1084 (s), 1074 (s), 1026 (vs), 1016 (vs), 1000 (vs), 980 (m), 959 (w), 926 (w), 914 (w), 875 (w), 836 (m), 822 (m), 776 (s), 761 (vs), 714 (vs), 704 (s), 691 (vs), 656 (m), 645 (m), 603 (s), 561 (w), 523 (w), 501 (m), 470 (m), 450 (w), 399 (w) cm^{-1}.

Additional information on the chemical synthesis is available *via* Chemotion repository: https://doi.org/10.14272/reaction/SA-FUHFF-UHFFFADPSC-BFHMQIVAON-UHFFFADPSC-NUHFF-NUHFF-NUHFF-ZZZ

Additional information on the analysis of the target compound is available *via* Chemotion repository:
https://doi.org/10.14272/BFHMQIVAONQIIQ-UHFFFAOYSA-N.1

Ethyl 3-(4-(acetoxymethyl)-1*H*-1,2,3-triazol-1-yl)-1-(3,5-difluorobenzyl)-1*H*-pyrazole-4-carboxylate (**21lc**)

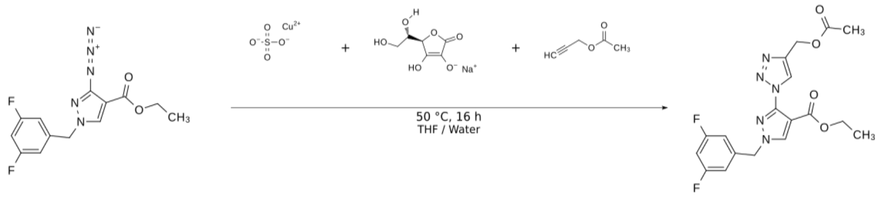

Name {P1|**21lc**}: ethyl 3-(4-(acetoxymethyl)-1*H*-1,2,3-triazol-1-yl)-1-(3,5-difluorobenzyl)-1*H*-pyrazole-4-carboxylate; Formula: $C_{18}H_{17}F_2N_5O_4$; Smiles: CCOC(=O)c1cn(nc1n1nnc(c1)COC(=O)C)Cc1cc(F)cc(c1)F. InChIKey: OEMVMGJIOKDNBC-UHFFFAOYSA-N

In a vial, ethyl 3-azido-1-(3,5-difluorobenzyl)-1*H*-pyrazole-4-carboxylate (50.0 mg, 163 µmol, 1.00 equiv) was dissolved in THF (5 mL) and water (5 mL) (1:1) and copper;sulfate (5.19 mg, 32.5 µmol, 0.20 equiv), sodium ascorbate (32.2 mg, 163 µmol, 1.00 equiv) and prop-2-ynyl acetate (20.8 mg, 21.0 µL, 212 µmol, 1.30 equiv) were added. The reaction mixture was stirred at 50 °C for 16 hours. For workup, the reaction mixture was diluted in water and extracted three times with methylene chloride. The combined organic phases were washed with water and brine, dried over Na_2SO_4, filtered and the solvent was removed under reduced pressure. As preparation for the column chromatography (dry load), Celite was added (0.2 g) and the reaction mixture with Celite was evaporated. The obtained crude product was purified *via* flash-chromatography (Interchim devices puriFLASH 5.125) on silica gel (PF-15SIHP-F0012) using cyclohexane/ethyl acetate with 0% to 50% ethyl acetate in 15 column volumes. The isolated product ethyl 3-(4-(acetoxymethyl)-1*H*-1,2,3-triazol-1-yl)-1-(3,5-difluorobenzyl)-1*H*-pyrazole-4-carboxylate (58.5 mg, 144 µmol) was obtained as a colourless oil in 89% yield.

R_f = 0.31 (cyclohexane/ethyl acetate 1:1). ^{1}H NMR (400 MHz, Chloroform-d [7.27 ppm], ppm) δ = 8.28 (s, 1H), 8.06 (s, 1H), 6.85–6.77 (m, 3H), 5.32 (s, 2H), 5.28 (s, 2H), 4.21 (q, *J* = 7.1 Hz, 2H), 2.06 (s, 3H), 1.23 (t, *J* = 7.2 Hz, 3H); ^{13}C NMR (100 MHz, Chloroform-d [77.0 ppm], ppm) δ = 170.7 (C_q), 163.29 (dd, *J* = 250.8, 12.7 Hz, C_q, 2C), 160.6 (C_q), 144.7 (C_q), 142.1 (C_q), 137.8 (t, *J* = 9.2 Hz, C_q), 135.2 (CH), 126.2 (CH), 111.0 (dd, *J* = 6.9 Hz, *J* = 18.5 Hz, CH, 2C), 108.5 (C_q), 104.4 (t, *J* = 25.0 Hz, CH), 60.9 (CH_2), 57.3 (CH_2), 56.0 (t, *J* = 1.9 Hz, CH_2), 20.7 (CH_3), 14.0 (CH_3); ^{19}F NMR (376 MHz, ppm) δ = -107.74; MS (FAB, 3-NBA), m/z (%): 406 (100) $[M+1]^+$, 318 (23), 290 (33), 127 (63), 95 (11), 91 (12). HRMS–FAB ($C_{18}H_{18}O_4N_5F_2$) *(m/z)*: $[M+H]^+$ Calcd 406.1321; Found 406.1322; IR (ATR, ṽ) = 3084 (vw), 2982 (w), 2962 (vw), 1718 (vs), 1625 (s), 1598 (s), 1550 (s), 1540 (s), 1462 (s), 1446 (s), 1408 (w), 1381 (m), 1364 (s), 1319 (s), 1235 (vs), 1160 (s), 1118 (vs), 1026 (vs), 999 (vs), 919 (m), 841 (s), 771 (vs), 734 (m), 686 (m), 654 (m), 622 (m), 605 (m), 589 (m), 554 (w), 510 (m), 494 (w), 466 (w), 455 (w), 442 (w), 438 (w), 424 (w), 405 (w), 395 (w), 381 (w) cm^{-1}.

Additional information on the chemical synthesis is available *via* Chemotion repository: https://doi.org/10.14272/reaction/SA-FUHFF-UHFFFADPSC-OEMVMGJIOK-UHFFFADPSC-NUHFF-NUHFF-NUHFF-ZZZ

Additional information on the analysis of the target compound is available *via* Chemotion repository:
https://doi.org/10.14272/OEMVMGJIOKDNBC-UHFFFAOYSA-N.1

Ethyl 1-(3,5-difluorobenzyl)-3-(4-(4-(methoxycarbonyl)phenyl)-1*H*-1,2,3-triazol-1-yl)-1*H*-pyrazole-4-carboxylate (21lf)

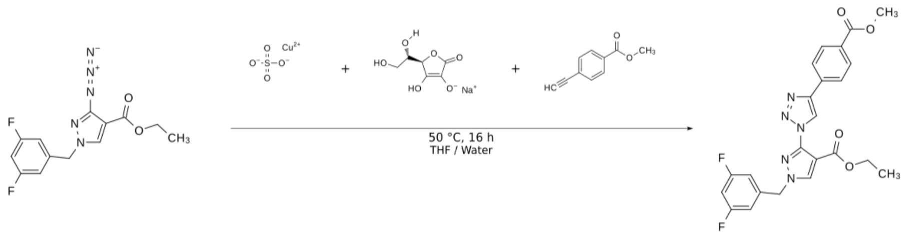

Name {P1|**21lf**}: ethyl 1-(3,5-difluorobenzyl)-3-(4-(4-(methoxycarbonyl)phenyl)-1*H*-1,2,3-triazol-1-yl)-1*H*-pyrazole-4-carboxylate; Formula: $C_{23}H_{19}F_2N_5O_4$; Smiles: CCOC(=O)c1cn(nc1n1nnc(c1)c1ccc(cc1)C(=O)OC)Cc1cc(F)cc(c1)F. InChIKey: WJPCYFBJDHGFTN-UHFFFAOYSA-N

In a vial, ethyl 3-azido-1-(3,5-difluorobenzyl)-1H-pyrazole-4-carboxylate (50.0 mg, 163 µmol, 1.00 equiv) was dissolved in THF (6.00 mL) and water (6.00 mL) (1:1) and copper;sulfate (5.19 mg, 32.5 µmol, 0.20 equiv), sodium ascorbate (32.2 mg, 163 µmol, 1.00 equiv) and methyl 4-ethynylbenzoate (33.9 mg, 212 µmol, 1.30 equiv) were added. The reaction mixture was stirred at 50 °C for 16 hours. For work up the reaction mixture was diluted in water and extracted three times with methylene chloride. The combined organic phase was washed with water and brine, dried over Na_2SO_4 and filtered. As preparation for the column chromatography (dryload), Celite was added (0.2 g) and the reaction mixture with Celite was evaporated. The obtained crude product was purified *via* flash-chromatography (Interchim devices puriFLASH 5.125) on silica gel (PF-15SIHP-F0012) using cyclohexane/ethyl acetate 0% to 50% ethyl acetate in 15 column volumes (1 column volume = 39.3 mL; flow: 15 mL/min). The isolated product ethyl 1-(3,5-difluorobenzyl)-3-(4-(4-(methoxycarbonyl)phenyl)-1*H*-

1,2,3-triazol-1-yl)-1*H*-pyrazole-4-carboxylate (70.8 mg, 151 µmol) was obtained as a colorless solid in 93% yield.

R_f = 0.53 (cyclohexane/ethyl acetate 1:1). MP = 160.4–162.2°C. ^{1}H NMR (400 MHz, Chloroform-d [7.27 ppm], ppm) δ = 8.59 (s, 1H), 8.10–8.08 (m, 3H), 7.99–7.96 (m, 2H), 6.87–6.76 (m, 3H), 5.34 (s, 2H), 4.23 (q, *J* = 7.1 Hz, 2H), 3.91 (s, 3H), 1.23 (t, *J* = 7.2 Hz, 3H); ^{13}C NMR (100 MHz, Chloroform-d [77.0 ppm], ppm) δ = 166.6, 163.2 (d, *J* = 251.2 Hz), 163.1 (d, *J* = 250.5 Hz), 160.7, 146.0, 144.7, 137.8, 137.8 (d, *J* = 18.5 Hz), 135.3, 134.4, 130.1, 129.6, 125.6, 122.8, 111.0 (dd, *J* = 7.7 Hz, *J* = 19.3 Hz, 2C), 108.4, 104.3 (d, *J* = 50.9 Hz), 104.3, 61.0, 56.0, 52.0, 14.0; ^{19}F NMR (376 MHz, ppm) δ = -107.70–-107.78 (m); MS (FAB, 3-NBA), m/z (%): 468 (100) $[M+1]^+$, 439 (18), 307 (15), 154 (54), 136 (39), 127 (14), 107 (13), 91 (10), 89 (12). HRMS–FAB ($C_{23}H_{20}O_4N_5F_2$) *(m/z)*: $[M+H]^+$ Calcd 468.1478; Found 468.1480; IR (ATR, ṽ) = 3128 (w), 1713 (w), 1696 (vs), 1618 (m), 1601 (s), 1544 (w), 1517 (s), 1493 (w), 1469 (m), 1439 (s), 1424 (w), 1397 (m), 1368 (w), 1353 (w), 1337 (w), 1313 (vs), 1298 (s), 1285 (vs), 1264 (s), 1211 (s), 1179 (m), 1164 (s), 1140 (w), 1115 (vs), 1082 (vs), 1021 (s), 1009 (vs), 1000 (vs), 977 (m), 960 (m), 943 (m), 878 (w), 858 (m), 846 (vs), 832 (s), 769 (vs), 710 (s), 696 (m), 686 (s), 652 (m), 633 (w), 611 (w), 598 (s), 565 (s), 534 (w), 521 (w), 509 (s), 475 (m), 458 (w), 422 (w), 415 (w), 390 (w), 381 (w) cm^{-1}.

Additional information on the chemical synthesis is available *via* Chemotion repository: https://doi.org/10.14272/reaction/SA-FUHFF-UHFFFADPSC-WJPCYFBJDH-UHFFFADPSC-NUHFF-NUHFF-NUHFF-ZZZ

Additional information on the analysis of the target compound is available *via* Chemotion repository:
https://doi.org/10.14272/WJPCYFBJDHGFTN-UHFFFAOYSA-N.1

Ethyl 1-(3-cyanobenzyl)-3-(4-(4-methoxyphenyl)-1*H*-1,2,3-triazol-1-yl)-1*H*-pyrazole-4-carboxylate (21me)

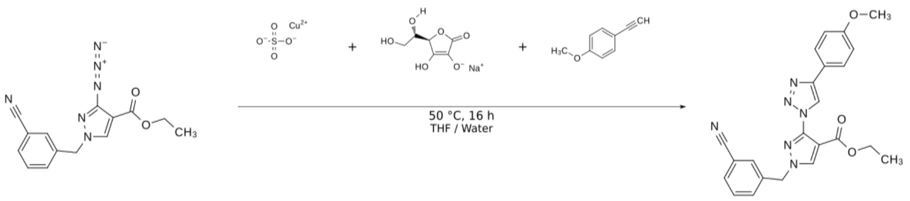

Name {P1|**21me**}: ethyl 1-(3-cyanobenzyl)-3-(4-(4-methoxyphenyl)-1*H*-1,2,3-triazol-1-yl)-1*H*-pyrazole-4-carboxylate; Formula: $C_{23}H_{20}N_6O_3$; Smiles: CCOC(=O)c1cn(nc1n1nnc(c1)c1ccc(cc1)OC)Cc1cccc(c1)C#N. InChIKey: KJMWCYALHNEPDX-UHFFFAOYSA-N

In a vial, ethyl 3-azido-1-(3-cyanobenzyl)-1*H*-pyrazole-4-carboxylate (50.0 mg, 169 µmol, 1.00 equiv) was dissolved in THF (6.00 mL) and water (6.00 mL) (1:1) and copper;sulfate (5.39 mg, 33.8 µmol, 0.20 equiv), sodium ascorbate (33.4 mg, 169 µmol, 1.00 equiv) and 1-ethynyl-4-methoxybenzene (29.0 mg, 28.5 µL, 219 µmol, 1.30 equiv) were added. The reaction mixture was stirred at 50 °C for 16 hours. For workup, the reaction mixture was diluted in water and extracted three times with methylene chloride. The combined organic phases were washed with water and brine, dried over Na_2SO_4, filtered and the solvent was removed under reduced pressure. As preparation

for column chromatography (dry load), Celite was added (0.2 g) and the reaction mixture with Celite was evaporated. The obtained crude product was purified *via* flash-chromatography (Interchim devices puriFLASH 5.125) on silica gel (PF-15SIHP-F0012) using cyclohexane/ethyl acetate with 0% to 100% ethyl acetate in 30 column volumes. The isolated product ethyl 1-(3-cyanobenzyl)-3-(4-(4-methoxyphenyl)-1*H*-1,2,3-triazol-1-yl)-1*H*-pyrazole-4-carboxylate (71.5 mg, 167 µmol) was obtained as a colourless oil in 99% yield.

R_f = 0.25 (cyclohexane/ethyl acetate 1:1). ^{1}H NMR (400 MHz, Chloroform-d [7.27 ppm], ppm) δ = 8.37 (s, 1H), 8.09 (s, 1H), 7.83–7.79 (m, 2H), 7.64–7.56 (m, 3H), 7.49 (pseudo-t, *J* = 7.7 Hz, 1H), 6.98–6.94 (m, 2H), 5.39 (s, 2H), 4.22 (q, *J* = 7.1 Hz, 2H), 3.83 (s, 3H), 1.22 (t, *J* = 7.2 Hz, 3H); ^{13}C NMR (100 MHz, Chloroform-d [77.0 ppm], ppm) δ = 160.7 (C_q), 159.6 (C_q), 146.9 (C_q), 145.0 (C_q), 135.9 (C_q), 135.3 (CH), 132.4 (CH), 132.3 (CH), 131.4 (CH), 130.0 (CH), 127.1 (CH, 2C), 122.7 (C_q), 121.1 (CH), 118.0 (C_q), 114.2 (CH, 2C), 113.1 (C_q), 108.4 (C_q), 60.9 (CH_2), 56.0 (CH_2), 55.2 (CH_3), 14.0 (CH_3); MS (FAB, 3-NBA), m/z (%): 429 (100) $[M+1]^+$, 400 (13), 284 (12), 154 (11), 147 (17), 136 (19), 131 (12), 129 (12), 121 (20), 119 (11), 116 (22), 109 (14), 107 (11), 105 (15), 97 (17), 95 (21), 93 (12), 91 (41), 89 (11). HRMS–FAB ($C_{23}H_{21}O_3N_6$) *(m/z)*: $[M+H]^+$ Calcd 429.1670; Found 429.1668; IR (ATR, ṽ) = 3138 (w), 2992 (w), 2955 (w), 2938 (w), 2929 (w), 2907 (w), 2232 (w), 1715 (vs), 1615 (w), 1547 (s), 1537 (s), 1494 (s), 1486 (s), 1480 (s), 1458 (m), 1441 (m), 1428 (w), 1402 (m), 1373 (w), 1361 (w), 1336 (w), 1323 (w), 1288 (m), 1262 (vs), 1247 (vs), 1235 (vs), 1205 (w), 1179 (vs), 1167 (vs), 1136 (vs), 1113 (m), 1085 (vs), 1020 (vs), 989 (s), 966 (w), 952 (w), 932 (w), 908 (w), 873 (w), 843 (vs), 813 (m), 802 (vs), 773 (vs), 755 (vs), 724 (s), 703 (w), 687 (s), 662 (w), 652 (w), 639 (w), 618 (s), 605 (w), 586 (s), 564 (w), 552 (w), 533 (s), 509 (w), 466 (m), 435 (w), 411 (w), 402 (w), 382 (w) cm^{-1}.

Additional information on the chemical synthesis is available *via* Chemotion repository: https://doi.org/10.14272/reaction/SA-FUHFF-UHFFFADPSC-KJMWCYALHN-UHFFFADPSC-NUHFF-NUHFF-NUHFF-ZZZ

Additional information on the analysis of the target compound is available *via* Chemotion repository:
https://doi.org/10.14272/KJMWCYALHNEPDX-UHFFFAOYSA-N.1

Ethyl 1-(3-cyanobenzyl)-3-(4-((1,3-dioxoisoindolin-2-yl)methyl)-1*H*-1,2,3-triazol-1-yl)-1*H*-pyrazole-4-carboxylate (21mh)

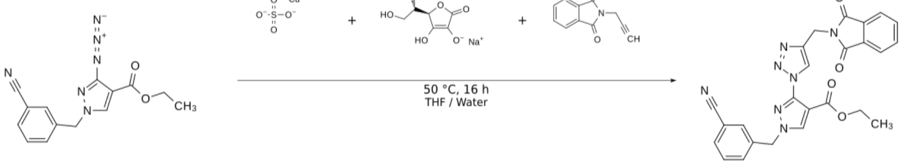

Name {P1|**21mh**}: ethyl 1-(3-cyanobenzyl)-3-(4-((1,3-dioxoisoindolin-2-yl)methyl)-1*H*-1,2,3-triazol-1-yl)-1*H*-pyrazole-4-carboxylate; Formula: $C_{25}H_{19}N_7O_4$; Smiles: CCOC(=O)c1cn(nc1n1nnc(c1)CN1C(=O)c2c(C1=O)cccc2)Cc1cccc(c1)C#N.
InChIKey: RJXQILXRDFYRBG-UHFFFAOYSA-N

In a vial, ethyl 3-azido-1-(3-cyanobenzyl)-1*H*-pyrazole-4-carboxylate (50.0 mg, 169 µmol, 1.00 equiv) was dissolved in THF (5.00 mL) and water (5.00 mL) (1:1) and copper;sulfate (5.39 mg, 33.8 µmol, 0.20 equiv), sodium ascorbate (33.4 mg, 169 µmol, 1.00 equiv) and 2-prop-2-ynylisoindole-1,3-dione (40.6 mg, 219 µmol, 1.30 equiv) were added. The reaction mixture was stirred at 50 °C for 16 hours. For workup, the reaction mixture was diluted in water and extracted three times with methylene chloride. The combined organic phases were washed with water and brine, dried over Na_2SO_4, filtered and the solvent was removed under reduced pressure. As preparation for column chromatography (dry load), Celite was added (0.2 g) and the reaction mixture with Celite was evaporated. The obtained crude product was purified *via* flash-chromatography (Interchim devices puriFLASH 5.125) on silica gel (PF-15SIHP-F0012) using cyclohexane/ethyl acetate 0% to 75% ethyl acetate in 12 column volumes. The isolated product ethyl 1-(3-cyanobenzyl)-3-(4-((1,3-dioxoisoindolin-2-yl)methyl)-1*H*-1,2,3-triazol-1-yl)-1*H*-pyrazole-4-carboxylate (75.5 mg, 157 µmol) was obtained as a colorless oil in 93% yield.

R_f = 0.13 (cyclohexane/ethyl acetate 1:1). ^{1}H NMR (400 MHz, Chloroform-d [7.27 ppm], ppm) δ = 8.23 (s, 1H), 8.08 (s, 1H), 7.84–7.79 (m, 2H), 7.72–7.67 (m, 2H), 7.61 (dt, *J* = 7.6, 1.5 Hz, 1H), 7.58–7.52 (m, 2H), 7.47 (t, *J* = 7.7 Hz, 1H), 5.37 (s, 2H), 5.06 (s, 2H), 4.16 (q, *J* = 7.2 Hz, 2H), 1.15 (t, *J* = 7.2 Hz, 3H); ^{13}C NMR (100 MHz, Chloroform-d [77.0 ppm], ppm) δ = 167.4 (C_q, 2C), 160.5 (C_q), 144.6 (C_q), 142.0 (C_q), 135.9 (C_q), 135.3 (CH), 134.0 (CH, 2C), 132.4 (CH), 132.3 (CH), 131.9 (C_q, 2C), 131.4 (CH), 129.9 (CH), 125.4 (CH), 123.3 (CH, 2C), 117.9 (C_q), 113.1 (C_q), 108.7 (C_q), 60.9 (CH_2), 56.0 (CH_2), 32.8 (CH_2), 13.9 (CH_3); MS (FAB, 3-NBA), m/z (%): 482 (100) $[M+1]^+$, 279 (12), 154 (17), 136 (15), 123 (12), 116 (22), 111 (11), 109 (16), 105 (11), 97 (25), 95 (23), 91 (20). HRMS–FAB ($C_{25}H_{20}O_4N_7$) *(m/z)*: $[M+H]^+$ Calcd 482.1571; Found 482.1572; IR (ATR, ṽ) = 3139 (vw), 2982 (vw), 2231 (w), 1771 (w), 1707 (vs), 1611 (w), 1585 (w), 1551 (m), 1537 (m), 1485 (w), 1466 (w), 1428 (m), 1392 (vs), 1371 (s), 1357 (s), 1326 (m), 1299 (w), 1242 (vs), 1188 (w), 1159 (m), 1112 (vs), 1098 (s), 1088 (s), 1040 (s), 1024 (m), 986 (w), 935 (s), 914 (m), 863 (w), 840 (w), 798 (w), 768 (s), 752 (s), 713 (vs), 686 (s), 657 (m), 647 (m), 613 (m), 586 (w), 550 (m), 528 (s), 487 (w), 460 (w), 439 (w), 399 (w), 384 (w) cm^{-1}.

Additional information on the chemical synthesis is available *via* Chemotion repository: https://doi.org/10.14272/reaction/SA-FUHFF-UHFFFADPSC-RJXQILXRDF-UHFFFADPSC-NUHFF-NUHFF-NUHFF-ZZZ

Additional information on the analysis of the target compound is available *via* Chemotion repository:
https://doi.org/10.14272/RJXQILXRDFYRBG-UHFFFAOYSA-N.1

Ethyl 1-(4-bromobenzyl)-3-(4-butyl-1*H*-1,2,3-triazol-1-yl)-1*H*-pyrazole-4-carboxylate (21na)

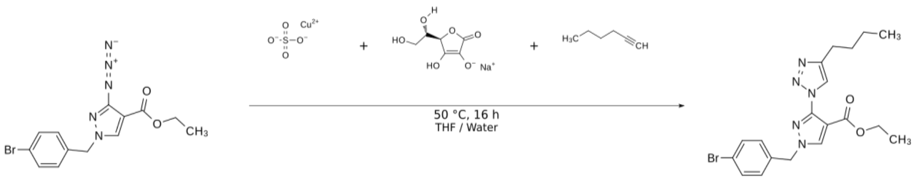

Name {P1|**21na**}: ethyl 1-(4-bromobenzyl)-3-(4-butyl-1*H*-1,2,3-triazol-1-yl)-1*H*-pyrazole-4-carboxylate; Formula: $C_{19}H_{22}BrN_5O_2$; Smiles: CCCCc1nnn(c1)c1nn(cc1C(=O)OCC)Cc1ccc(cc1)Br. InChIKey: YERIOOVHZOGENU-UHFFFAOYSA-N

In a vial, ethyl 3-azido-1-(4-bromobenzyl)-1*H*-pyrazole-4-carboxylate (50.0 mg, 143 µmol, 1.00 equiv) was dissolved in THF (5.00 mL) and water (5.00 mL) (1:1) and copper;sulfate (4.56 mg, 28.6 µmol, 0.20 equiv), sodium ascorbate (28.3 mg, 143 µmol, 1.00 equiv) and hex-1-yne (15.2 mg, 21.2 µL, 186 µmol, 1.30 equiv) were added. The reaction mixture was stirred at 50 °C for 16 hours. For workup, the reaction mixture was diluted in water and extracted three times with methylene chloride. The combined organic phases were washed with water and brine, dried over Na_2SO_4, filtered and the solvent was removed under reduced pressure. As preparation for column chromatography (dry load), Celite was added (0.2 g) and the reaction mixture with Celite was evaporated. The obtained crude product was purified *via* flash-chromatography (Interchim devices puriFLASH 5.125) on silica gel (PF-15SIHP-F0012) using cyclohexane/ethyl acetate with 0% to 50% ethyl acetate in 17 column volumes. The isolated product ethyl 1-(4-bromobenzyl)-3-(4-butyl-1*H*-1,2,3-triazol-1-yl)-1*H*-pyrazole-4-carboxylate (53.6 mg, 124 µmol) was obtained as a colourless oil in 87% yield.

R_f = 0.50 (cyclohexane/ethyl acetate 1:1). ^{1}H NMR (400 MHz, Chloroform-d [7.27 ppm], ppm) δ = 7.94 (s, 2H), 7.52–z7.49 (m, 2H), 7.21–7.18 (m, 2H), 5.28 (s, 2H), 4.19 (q, *J* = 7.2 Hz, 2H), 2.79 (t, *J* = 7.6 Hz, 2H), 1.70 (quint, *J* = 7.5 Hz, 2H), 1.41 (h, *J* = 7.4 Hz, 2H), 1.22 (t, *J* = 7.1 Hz, 3H), 0.93 (t, *J* = 7.4 Hz, 3H); ^{13}C NMR (100 MHz, Chloroform-d [77.0 ppm], ppm) δ = 160.8 (C_q), 147.6 (C_q), 144.9 (C_q), 134.7 (CH), 133.1 (C_q), 132.2 (CH, 2C), 129.9 (CH, 2C), 123.1 (C_q), 123.0 (CH), 108.1 (C_q), 60.7 (CH_2), 56.4 (CH_2), 31.4 (CH_2), 25.1 (CH_2), 22.2 (CH_2), 14.0 (CH_3), 13.7 (CH_3); MS (FAB, 3-NBA), m/z (%): 432/434 (100/92), 404 (10), 169/171 (49/48), 109 (12), 105 (13), 91 (31). HRMS–FAB ($C_{19}H_{23}O_2N_5Br$) *(m/z)*: $[M+H]^+$ Calcd 432.1030; Found 432.1031; IR (ATR, ṽ) = 2956 (w), 2929 (w), 2870 (w), 2860 (w), 1717 (vs), 1594 (w), 1548 (vs), 1533 (vs), 1489 (s), 1458 (m), 1441 (m), 1405 (m), 1368 (m), 1329 (m), 1300 (m), 1241 (vs), 1154 (vs), 1112 (vs), 1071 (s), 1038 (vs), 1011 (vs), 983 (s), 911 (m), 839 (s), 803 (vs), 771 (vs), 755 (vs), 731 (vs), 693 (m), 650 (m), 613 (m), 585 (m), 516 (m), 475 (m) cm^{-1}.

Additional information on the chemical synthesis is available *via* Chemotion repository: https://doi.org/10.14272/reaction/SA-FUHFF-UHFFFADPSC-YERIOOVHZO-UHFFFADPSC-NUHFF-NUHFF-NUHFF-ZZZ

Additional information on the analysis of the target compound is available *via* Chemotion repository: https://doi.org/10.14272/YERIOOVHZOGENU-UHFFFAOYSA-N.1

Ethyl 3-(4-(acetoxymethyl)-1*H*-1,2,3-triazol-1-yl)-1-(4-bromobenzyl)-1*H*-pyrazole-4-carboxylate (**21nc**)

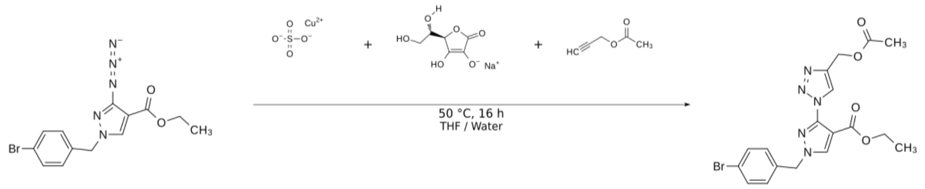

Name {P1|**21nc**}: ethyl 3-(4-(acetoxymethyl)-1*H*-1,2,3-triazol-1-yl)-1-(4-bromobenzyl)-1*H*-pyrazole-4-carboxylate; Formula: $C_{18}H_{18}BrN_5O_4$; Smiles: CCOC(=O)c1cn(nc1n1nnc(c1)COC(=O)C)Cc1ccc(cc1)Br. InChIKey: JIGNGCLNWAMKPG-UHFFFAOYSA-N

In a vial, ethyl 3-azido-1-(4-bromobenzyl)-1*H*-pyrazole-4-carboxylate (50.0 mg, 143 µmol, 1.00 equiv) was dissolved in THF (5 mL) and water (5 mL) (1:1) and copper;sulfate (4.56 mg, 28.6 µmol, 0.20 equiv), sodium ascorbate (28.3 mg, 143 µmol, 1.00 equiv) and prop-2-ynyl acetate (18.2 mg, 18.4 µL, 186 µmol, 1.30 equiv) were added. The reaction mixture was stirred at 50 °C for 16 hours. For workup, the reaction mixture was diluted in water and extracted three times with methylene chloride. The combined organic phases were washed with water and brine, dried over Na_2SO_4, filtered and the solvent was removed under reduced pressure. As preparation for column chromatography (dry load), Celite was added (0.2 g) and the reaction mixture with Celite was evaporated. The obtained crude product was purified *via* flash-chromatography (Interchim devices puriFLASH 5.125) on silica gel (PF-15SIHP-F0012) using cyclohexane/ethyl acetate with 0% to 70% ethyl acetate in 15 column volumes. The isolated product ethyl 3-(4-(acetoxymethyl)-1*H*-1,2,3-triazol-1-yl)-1-(4-bromobenzyl)-1*H*-pyrazole-4-carboxylate (59.1 mg, 132 µmol) was obtained as a colorless oil in 92% yield.

R_f = 0.34 (cyclohexane/ethyl acetate 1:1). ^{1}H NMR (400 MHz, Chloroform-d [7.27 ppm], ppm) δ = 8.26 (s, 1H), 7.96 (s, 1H), 7.53–7.50 (m, 2H), 7.22–7.19 (m, 2H), 5.29 (d, *J* = 4.0 Hz, 4H), 4.20 (q, *J* = 7.2 Hz, 2H), 2.07 (s, 3H), 1.22 (t, *J* = 7.1 Hz, 3H). ^{13}C NMR (100 MHz, Chloroform-d [77.0 ppm], ppm) δ = 170.7 (C_q), 160.7 (C_q), 144.5 (C_q), 142.0 (C_q), 134.8 (CH), 133.0 (C_q), 132.3 (CH, 2C), 129.9 (CH, 2C), 126.2 (CH), 123.1 (C_q), 108.3 (C_q), 60.9 (CH_2), 57.3 (CH_2), 56.5 (CH_2), 20.8 (CH_3), 14.0 (CH_3); MS (FAB, 3-NBA), m/z (%): 448/450 (20/20) $[M+1]^+$, 169/171 (100/96), 154 (11), 146 (11), 136 (17), 133 (70), 128 (12), 115 (16), 105/107 (16/12), 105 (16), 91 (48). HRMS–FAB ($C_{18}H_{19}O_4N_5Br$) *(m/z)*: $[M+H]^+$ Calcd 448.0615; Found 448.0613; IR (ATR, ṽ) = 3133 (vw), 2980 (w), 1717 (vs), 1595 (w), 1540 (s), 1489 (m), 1441 (m), 1407 (m), 1381 (m), 1364 (s), 1333 (m), 1302 (w), 1237 (vs), 1156 (vs), 1113 (vs), 1071 (s), 1026 (vs), 1013 (vs), 987 (s), 912 (m), 839 (s), 805 (s), 771 (vs), 755 (s), 731 (vs), 694 (m), 663 (w), 647 (m), 620 (m), 603 (m), 586 (m), 514 (m), 489 (m), 477 (m), 402 (w) cm^{-1}.

Additional information on the chemical synthesis is available *via* Chemotion repository: https://doi.org/10.14272/reaction/SA-FUHFF-UHFFFADPSC-JIGNGCLNWA-UHFFFADPSC-NUHFF-NUHFF-NUHFF-ZZZ

Additional information on the analysis of the target compound is available *via* Chemotion repository:
https://doi.org/10.14272/JIGNGCLNWAMKPG-UHFFFAOYSA-N.1

Ethyl 1-(4-bromobenzyl)-3-(4-phenyl-1*H*-1,2,3-triazol-1-yl)-1*H*-pyrazole-4-carboxylate (21nd)

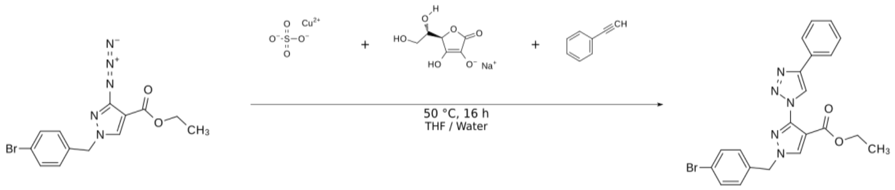

Name {P1|**21nd**}: ethyl 1-(4-bromobenzyl)-3-(4-phenyl-1*H*-1,2,3-triazol-1-yl)-1*H*-pyrazole-4-carboxylate; Formula: $C_{21}H_{18}BrN_5O_2$; Smiles: CCOC(=O)c1cn(nc1n1nnc(c1)c1ccccc1)Cc1ccc(cc1)Br. InChIKey: VTYPYIREEKHMKL-UHFFFAOYSA-N

In a vial, ethyl 3-azido-1-(4-bromobenzyl)-1H-pyrazole-4-carboxylate (50.0 mg, 143 µmol, 1.00 equiv) was dissolved in THF (5.00 mL) and water (5.00 mL) (1:1) and copper;sulfate (4.56 mg, 28.6 µmol, 0.20 equiv), sodium ascorbate (28.3 mg, 143 µmol, 1.00 equiv) and ethynylbenzene (19.0 mg, 20.4 µL, 186 µmol, 1.30 equiv) were added. The reaction mixture was stirred at 50 °C for 16 hours. For workup, the reaction mixture was diluted in water and extracted three times with methylene chloride. The combined organic phases were washed with water and brine, dried over Na_2SO_4, filtered and the solvent was removed under reduced pressure. As preparation for the column chromatography (dry load), Celite was added (0.2 g) and the reaction mixture with Celite was evaporated. The obtained crude product was purified *via* flash-chromatography (Interchim devices puriFLASH 5.125) on silica gel (PF-15SIHP-F0012) using cyclohexane/ethyl acetate with 0% to 50% ethyl acetate in 20 column volumes. The isolated product ethyl 1-(4-bromobenzyl)-3-(4-phenyl-1*H*-1,2,3-triazol-1-yl)-1*H*-pyrazole-4-carboxylate (62.8 mg, 139 µmol) was obtained as colourless oil in 97% yield.

R_f = 0.54 (cyclohexane/ethyl acetate 1:1). ^{1}H NMR (400 MHz, Chloroform-d [7.27 ppm], ppm) δ = 8.46 (s, 1H), 7.98 (s, 1H), 7.92–7.89 (m, 2H), 7.54–7.51 (m, 2H), 7.46–7.42 (m, 2H), 7.37–7.33 (m, 1H), 7.24–7.21 (m, 2H), 5.30 (s, 2H), 4.22 (q, *J* = 7.1 Hz, 2H), 1.23 (t, *J* = 7.1 Hz, 3H); ^{13}C NMR (100 MHz, Chloroform-d [77.0 ppm], ppm) δ = 160.8 (C_q), 147.0 (C_q), 144.7 (C_q), 134.8 (CH), 133.0 (C_q), 132.3 (CH, 2C), 130.2 (C_q), 130.0 (CH, 2C), 128.8 (CH, 2C), 128.2 (CH), 125.9 (CH, 2C), 123.1 (C_q), 121.9 (CH), 108.2 (C_q), 60.9 (CH_2), 56.4 (CH_2), 14.1 (CH_3); MS (FAB, 3-NBA), m/z (%): 452/454 (97/100) $[M+1]^+$, 426 (10), 423/425 (12/14), 219 (14), 191 (14), 180 (10), 169/171 (59/59), 165/167 (12/11), 154 (33), 147 (25), 136 (43), 115/117 (26/23), 105/107 (28/27), 91 (88). HRMS–FAB ($C_{21}H_{19}O_2N_5Br$) *(m/z)*: $[M+H]^+$ Calcd 452.0717; Found 452.0716; IR (ATR, ṽ) = 3138 (w), 3091 (w), 3031 (w), 2993 (w), 2976 (w), 2966 (w), 2948 (w), 2928 (w), 2853 (w), 2122 (w), 1703 (vs), 1553 (vs), 1533 (vs), 1487 (m), 1477 (s), 1459 (w), 1448 (m), 1438 (m), 1407 (w), 1394 (w), 1370 (m), 1356 (w), 1337 (m), 1299 (w), 1259 (vs), 1237 (s), 1214 (m), 1184 (m), 1171 (vs), 1123 (s), 1086 (s), 1071 (s), 1035 (vs), 1020 (vs), 1010 (vs), 983 (m), 973 (m), 965 (m), 915 (w), 867 (w), 856 (w), 841 (s), 827 (m), 802 (m), 772 (vs), 762 (vs), 755 (vs), 710 (w), 691 (vs), 666 (s), 652 (m), 630 (w),

611 (w), 598 (m), 527 (w), 509 (s), 479 (s), 459 (w), 429 (w), 422 (w), 404 (w), 392 (w), 375 (m) cm^{-1}.

Additional information on the chemical synthesis is available *via* Chemotion repository: https://doi.org/10.14272/reaction/SA-FUHFF-UHFFFADPSC-VTYPYIREEK-UHFFFADPSC-NUHFF-NUHFF-NUHFF-ZZZ

Additional information on the analysis of the target compound is available *via* Chemotion repository:
https://doi.org/10.14272/VTYPYIREEKHMKL-UHFFFAOYSA-N.1

3-(4-Butyl-1*H*-1,2,3-triazol-1-yl)-1*H*-pyrazole-4-carbonitrile (**21oa**)

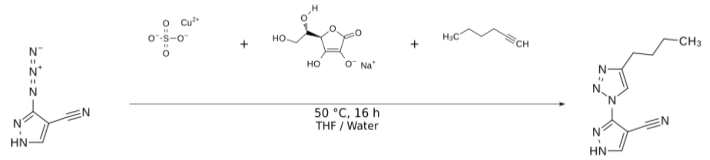

Name {P1|**21oa**}: 3-(4-butyl-1*H*-1,2,3-triazol-1-yl)-1*H*-pyrazole-4-carbonitrile; Formula: $C_{10}H_{12}N_6$; Smiles: CCCCc1nnn(c1)c1n[nH]cc1C#N. InChIKey: USLFBJCHBNQGJB-UHFFFAOYSA-N

In a vial, 5-azido-1*H*-pyrazole-4-carbonitrile (50.0 mg, 373 µmol, 1.00 equiv) was dissolved in THF (6.00 mL) and water (6.00 mL) (1:1) and copper;sulfate (11.9 mg, 74.6 µmol, 0.20 equiv), sodium ascorbate (73.9 mg, 373 µmol, 1.00 equiv) and hex-1-yne (39.8 mg, 55.3 µL, 485 µmol, 1.30 equiv) were added. The reaction mixture was stirred at 50 °C for 16 hours. For workup, the reaction mixture was diluted in water and extracted three times with methylene chloride. The combined organic phases were washed with water and brine, dried over Na_2SO_4, filtered and the solvent was removed under reduced pressure. As preparation for column chromatography (dry load), Celite was added (0.2 g) and the reaction mixture with Celite was evaporated. The obtained crude product was purified *via* flash-chromatography (Interchim devices puriFLASH 4125) on silica gel (PF-15SIHP-F0025) using cyclohexane/ethyl acetate with 0% to 63% ethyl acetate in 10 column volumes. The isolated product 3-(4-butyl-1*H*-1,2,3-triazol-1-yl)-1*H*-pyrazole-4-carbonitrile (36.8 mg, 170 µmol) was obtained as a colorless solid in 46% yield.

R_f = 0.35 (cyclohexane/ethyl acetate 1:2). MP = 224.9–247.9 °C. ^{1}H NMR (400 MHz, DMSO-d_6 [2.50 ppm], ppm) δ = 14.17 (br, s, 1H), 8.81 (s, 1H), 8.42 (s, 1H), 2.71 (t, *J* = 7.6 Hz, 2H), 1.64 (quint, *J* = 7.6 Hz, 2H), 1.39–1.30 (m, 2H), 0.90 (t, *J* = 7.3 Hz, 3H); ^{13}C NMR (100 MHz, DMSO-d_6 [39.5 ppm], ppm) δ = 147.9 (C_q), 146.0 (C_q), 138.3 (CH), 120.4 (CH), 112.5 (C_q), 83.3 (C_q), 30.8 (CH_2), 24.3 (CH_2), 21.6 (CH_2), 13.6 (CH_3); MS (EI, 70 eV, 180 °C), m/z (%): 216 (1) $[M]^+$, 145 (100), 119 (18), 81 (18), 64 (12). HRMS–EI ($C_{10}H_{12}N_6$) *(m/z)*: $[M]^+$ Calcd 216.1118; Found 216.1118; IR (ATR, ṽ) = 3167 (w), 3121 (m), 3087 (w), 3057 (w), 3027 (w), 2955 (m), 2927 (s), 2895 (m), 2868 (s), 2861 (s), 2844 (m), 2782 (s), 2757 (m), 2703 (m), 2683 (m), 2652 (m), 2468 (w), 2242 (vs), 1846 (w), 1687 (w), 1562 (m), 1538 (vs), 1514 (s), 1459 (m), 1435 (m), 1407 (w), 1383 (w), 1358 (w), 1340 (w), 1317 (m), 1298 (w), 1252 (m), 1228 (vs), 1201 (m), 1152 (w), 1105 (w), 1079 (m), 1065 (m), 1051 (vs), 1028 (s), 1007 (m), 989 (vs), 922 (vs), 901

(s), 857 (vs), 812 (vs), 785 (s), 762 (w), 734 (m), 694 (vs), 686 (m), 640 (w), 628 (s), 582 (s), 507 (m), 480 (m), 422 (w), 397 (m), 382 (w) cm^{-1}.

Additional information on the chemical synthesis is available *via* Chemotion repository: https://doi.org/10.14272/reaction/SA-FUHFF-UHFFFADPSC-USLFBJCHBN-UHFFFADPSC-NUHFF-NUHFF-NUHFF-ZZZ

Additional information on the analysis of the target compound is available *via* Chemotion repository:
https://doi.org/10.14272/USLFBJCHBNQGJB-UHFFFAOYSA-N.1

(1-(4-Cyano-1*H*-pyrazol-3-yl)-1*H*-1,2,3-triazol-4-yl)methyl acetate (**21oc**)

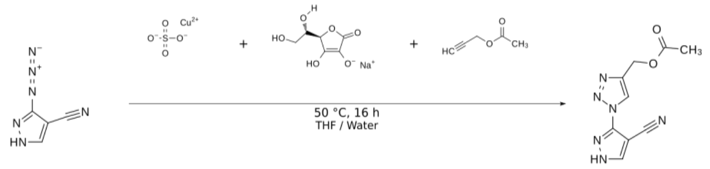

Name {P1|**21oc**}: (1-(4-cyano-1*H*-pyrazol-3-yl)-1*H*-1,2,3-triazol-4-yl)methyl acetate; Formula: $C_9H_8N_6O_2$; Smiles: N#Cc1c[nH]nc1n1nnc(c1)COC(=O)C. InChIKey: QROBBVJEUOFHFJ-UHFFFAOYSA-N

In a vial, 5-azido-1*H*-pyrazole-4-carbonitrile (50.0 mg, 373 µmol, 1.00 equiv) was dissolved in THF (6 mL) and water (6 mL) (1:1) and copper;sulfate (11.9 mg, 74.6 µmol, 0.20 equiv), sodium ascorbate (73.9 mg, 373 µmol, 1.00 equiv) and prop-2-ynyl acetate (47.6 mg, 48.1 µL, 485 µmol, 1.30 equiv) were added. The reaction mixture was stirred at 50 °C for 16 hours. For work up the reaction mixture was diluted in water and extracted three times with methylene chloride. The combined organic phase was washed with water and brine, dried over Na_2SO_4 and filtered. As preparation for the column chromatography (dryload), Celite was added (0.2 g) and the reaction mixture with Celite were evaporated. The obtained crude product was purified *via* flash-chromatography (Interchim devices puriFLASH 4125) on silica gel (PF-15SIHP-F0012) using cyclohexane/ethyl acetate 0% to 100% ethyl acetate in 20 column volumes (1 column volume = 39.3 mL; flow: 15 mL/min). The isolated product (1-(4-cyano-1*H*-pyrazol-3-yl)-1*H*-1,2,3-triazol-4-yl)methyl acetate (56.1 mg, 242 µmol) was obtained as a colorless solid in 65% yield.

R_f = 0.21 (cyclohexane/ethyl acetate 1:2). MP = 167.8–205.3 °C. ^{1}H NMR (400 MHz, DMSO-d_6 [2.50 ppm], ppm) δ = 14.25 (s, 1H), 8.83 (s, 1H), 8.70 (s, 1H), 5.23 (s, 2H), 2.06 (s, 3H); ^{13}C NMR (100 MHz, DMSO-d_6 [39.5 ppm], ppm) δ = 170.1, 145.7, 143.0, 138.4, 123.4, 112.3, 83.8, 56.6, 20.6; MS (FAB, 3-NBA), m/z (%): 233 (86) [M+1]$^+$, 231 (13), 219 (42), 191 (53), 175 (29), 161 (50), 159 (39), 154 (78), 147 (74), 145 (79), 136 (100), 109 (53), 95 (90), 91 (99). HRMS–FAB ($C_9H_9O_2N_6$) *(m/z)*: [M+H]$^+$ Calcd 233.0782; Found 233.0781; IR (ATR, ṽ) = 3159 (w), 3126 (m), 3108 (w), 3030 (w), 2925 (m), 2907 (m), 2851 (m), 2809 (m), 2655 (w), 2245 (m), 1744 (vs), 1572 (w), 1541 (vs), 1446 (w), 1426 (w), 1408 (w), 1385 (m), 1368 (s), 1316 (w), 1281 (m), 1225 (vs), 1211 (vs), 1197 (vs), 1149 (s), 1079 (w), 1058 (m), 1043 (vs), 1027 (vs), 1011 (vs), 989 (vs), 963 (s), 929 (s), 844 (vs), 823 (vs), 772 (vs), 694 (s), 659 (w), 637 (m), 623 (m), 606 (m), 584 (m), 507 (s), 494 (w), 435 (m), 416 (w) cm^{-1}.

Additional information on the chemical synthesis is available *via* Chemotion repository: https://doi.org/10.14272/reaction/SA-FUHFF-UHFFFADPSC-QROBBVJEUO-UHFFFADPSC-NUHFF-NUHFF-NUHFF-ZZZ

Additional information on the analysis of the target compound is available *via* Chemotion repository:
https://doi.org/10.14272/QROBBVJEUOFHFJ-UHFFFAOYSA-N.1

3-(4-Phenyl-1*H*-1,2,3-triazol-1-yl)-1*H*-pyrazole-4-carbonitrile (**21od**)

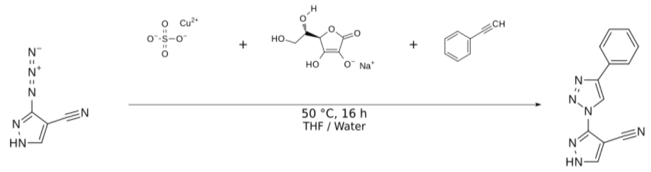

Name {P1|**21od**}: 3-(4-phenyl-1*H*-1,2,3-triazol-1-yl)-1*H*-pyrazole-4-carbonitrile; Formula: $C_{12}H_8N_6$; Smiles: N#Cc1c[nH]nc1n1nnc(c1)c1ccccc1. InChIKey: VSYUPFQKDFVQCM-UHFFFAOYSA-N

In a vial, 5-azido-1*H*-pyrazole-4-carbonitrile (50.0 mg, 373 µmol, 1.00 equiv) was dissolved in THF (6.00 mL) and water (6.00 mL) (1:1) and copper;sulfate (11.9 mg, 74.6 µmol, 0.20 equiv), sodium ascorbate (73.9 mg, 373 µmol, 1.00 equiv) and ethynylbenzene (49.5 mg, 53.2 µL, 485 µmol, 1.30 equiv) were added. The reaction mixture was stirred at 50 °C for 16 hours. For work up the reaction mixture was diluted in water and extracted three times with methylene chloride. The combined organic phase was washed with water and brine, dried over Na_2SO_4, filtered and the solvent was removed under reduced pressure. As preparation for the column chromatography (dryload), Celite was added (0.2 g) and the reaction mixture with Celite were evaporated. The obtained crude product was purified *via* flash-chromatography (Interchim devices puriFLASH 4125) on silica gel (PF-15SIHP-F0012) using cyclohexane/ethyl acetate 0% to 100% ethyl acetate in 20 column volumes. The isolated 3-(4-phenyl-1*H*-1,2,3-triazol-1-yl)-1*H*-pyrazole-4-carbonitrile (58.0 mg, 246 µmol) was obtained as a colorless solid in 66% yield.

R_f = 0.30 (cyclohexane/ethyl acetate 1:2). MP = 198.2 °C. ^{1}H NMR (400 MHz, DMSO-d_6 [2.50 ppm], ppm) δ = 14.28 (s, 1H), 9.21 (s, 1H), 8.86 (s, 1H), 8.02–8.00 (m, 2H), 7.49 (t, *J* = 7.6 Hz, 2H), 7.40–7.37 (m, 1H); ^{13}C NMR (100 MHz, DMSO-d_6 [39.5 ppm], ppm) δ = 147.1, 145.8, 138.5, 129.6, 129.0 (2C), 128.5, 125.6 (2C), 119.8, 112.4, 83.6; MS (EI, 70 eV, 220 °C), m/z (%): 236 (3) [M]$^+$, 208 (100), 181 (20), 165 (42), 145 (39), 137 (65), 119 (16), 107 (18), 102 (13), 90 (27), 74 (40), 64 (10). HRMS–EI ($C_{12}H_8N_6$) *(m/z)*: [M]$^+$ Calcd 236.0805; Found 236.0807; IR (ATR, ṽ) = 3285 (w), 3150 (m), 3077 (w), 3026 (w), 2921 (w), 2894 (w), 2871 (w), 2850 (w), 2793 (w), 2653 (w), 2242 (w), 2231 (s), 1561 (s), 1541 (vs), 1509 (w), 1480 (s), 1452 (m), 1404 (w), 1356 (w), 1323 (w), 1303 (w), 1282 (w), 1235 (s), 1215 (w), 1157 (w), 1140 (w), 1091 (w), 1077 (w), 1037 (s), 1021 (vs), 994 (vs), 941 (m), 921 (s), 844 (w), 824 (s), 815 (s), 759 (vs), 693 (vs), 622 (m), 609 (s), 589 (m), 504 (s), 479 (w), 435 (m), 392 (w) cm^{-1}.

Additional information on the chemical synthesis is available *via* Chemotion repository: https://doi.org/10.14272/reaction/SA-FUHFF-UHFFFADPSC-VSYUPFQKDF-UHFFFADPSC-NUHFF-NUHFF-NUHFF-ZZZ

Additional information on the analysis of the target compound is available *via* Chemotion repository:
https://doi.org/10.14272/VSYUPFQKDFVQCM-UHFFFAOYSA-N.1

Methyl 4-(1-(4-cyano-1*H*-pyrazol-3-yl)-1*H*-1,2,3-triazol-4-yl)benzoate (21of)

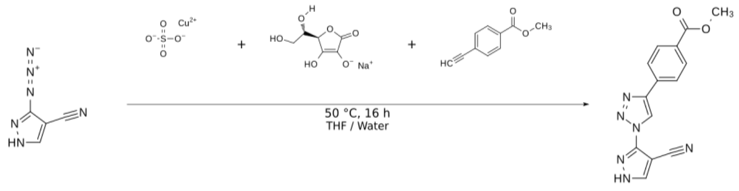

Name {P1|**21of**}: methyl 4-(1-(4-cyano-1*H*-pyrazol-3-yl)-1*H*-1,2,3-triazol-4-yl)benzoate; Formula: $C_{14}H_{10}N_6O_2$; Smiles: COC(=O)c1ccc(cc1)c1nnn(c1)c1n[nH]cc1C#N. InChIKey: FXIYGLHLKLOXQB-UHFFFAOYSA-N

In a vial, 5-azido-1*H*-pyrazole-4-carbonitrile (86.0 mg, 641 µmol, 1.00 equiv) was dissolved in THF (6.00 mL) and water (6.00 mL) (1:1) and copper;sulfate (20.5 mg, 128 µmol, 0.20 equiv), sodium ascorbate (127 mg, 641 µmol, 1.00 equiv) and methyl 4-ethynylbenzoate (134 mg, 834 µmol, 1.30 equiv) were added. The reaction mixture was stirred at 50 °C for 16 hours. For work up, the reaction mixture was diluted in water and extracted three times with methylene chloride. The combined organic phases were washed with water and brine, dried over Na_2SO_4 and filtered. As preparation for column chromatography (dry load), Celite was added (0.3 g) and the reaction mixture with Celite was evaporated. The obtained crude product was purified *via* flash-chromatography (Interchim devices puriFLASH 5.125) on silica gel (PF-15SIHP-F0025) using cyclohexane/ethyl acetate with 0% to 100% ethyl acetate in 20 column volumes (1 column volume = 63.8 mL; flow: 15 mL/min). The isolated product methyl 4-(1-(4-cyano-1*H*-pyrazol-3-yl)-1*H*-1,2,3-triazol-4-yl)benzoate (90.8 mg, 309 µmol) was obtained as a light yellow solid in 48% yield.

R_f = 0.35 (cyclohexane/ethyl acetate 1:2). MP = 200.4 – 241.9 °C. ^{1}H NMR (400 MHz, DMSO-d_6 [2.50 ppm], ppm) δ = 14.31 (br, s, 1H), 9.37 (s, 1H), 8.87 (s, 1H), 8.16 (d, J = 8.4 Hz, 2H), 8.07 (d, J = 8.3 Hz, 2H), 3.88 (s, 3H); ^{13}C NMR (100 MHz, DMSO-d_6 [39.5 ppm], ppm) δ = 165.8 (C_q), 146.0 (C_q), 145.7 (C_q), 138.5 (CH), 134.1 (C_q), 129.9 (CH, 2C), 129.3 (C_q), 125.8 (CH, 2C), 121.1 (CH), 112.4 (C_q), 83.7 (C_q), 52.2 (CH_3); MS (FAB, 3-NBA), m/z (%): 295 (33) $[M+1]^+$, 289 (12), 219 (11), 191 (12), 159 (12), 154 (100), 147 (17), 137 (63), 136 (76), 131 (13), 128 (11), 124 (10), 119 (15), 115 (14), 107 (27), 95 (10), 91 (32). HRMS–FAB ($C_{14}H_{11}O_2N_6$) *(m/z)*: $[M+H]^+$ Calcd 295.0938; Found 295.0937; IR (ATR, ṽ) = 3112 (w), 3080 (w), 3024 (w), 2945 (w), 2912 (w), 2897 (w), 2849 (w), 2798 (w), 2706 (w), 2664 (w), 2649 (w), 2234 (m), 1711 (vs), 1615 (w), 1560 (w), 1543 (vs), 1516 (w), 1490 (w), 1439 (m), 1421 (w), 1405 (w), 1312 (w), 1279 (vs), 1237 (s), 1215 (m), 1194 (m), 1173 (m), 1113 (s), 1089 (m), 1040 (s), 1020 (vs), 994 (vs), 963 (s), 931 (s), 856 (vs), 824 (vs), 773 (vs), 720 (m), 710 (s), 700 (vs), 657 (w), 629 (m), 613 (w), 595 (m), 507 (s), 476 (w), 453 (w) cm^{-1}.

Additional information on the chemical synthesis is available *via* Chemotion repository: https://doi.org/10.14272/reaction/SA-FUHFF-UHFFFADPSC-FXIYGLHLKL-UHFFFADPSC-NUHFF-NUHFF-NUHFF-ZZZ

Additional information on the analysis of the target compound is available *via* Chemotion repository:
https://doi.org/10.14272/FXIYGLHLKLOXQB-UHFFFAOYSA-N.1

3-(4-Butyl-1*H*-1,2,3-triazol-1-yl)-1-isopropyl-1*H*-pyrazole-4-carbonitrile (**21pa**)

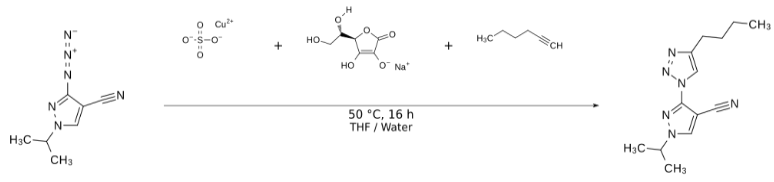

Name {P1|**21pa**}: 3-(4-butyl-1*H*-1,2,3-triazol-1-yl)-1-isopropyl-1*H*-pyrazole-4-carbonitrile; Formula: $C_{13}H_{18}N_6$; Smiles: CCCCc1nnn(c1)c1nn(cc1C#N)C(C)C. InChIKey: NOJZURFWHMPYCF-UHFFFAOYSA-N

In a vial, 3-azido-1-propan-2-yl-pyrazole-4-carbonitrile (64.0 mg, 363 µmol, 1.00 equiv) was dissolved in THF (7.00 mL) and water (7.00 mL) (1:1) and copper;sulfate (11.6 mg, 72.7 µmol, 0.20 equiv), sodium ascorbate (72.0 mg, 363 µmol, 1.00 equiv) and hex-1-yne (38.8 mg, 53.9 µL, 472 µmol, 1.30 equiv) were added. The reaction mixture was stirred at 50 °C for 16 hours. For workup, the reaction mixture was diluted in water and extracted three times with methylene chloride. The combined organic phases were washed with water and brine, dried over Na_2SO_4, filtered and the solvent was removed under reduced pressure. As preparation for column chromatography (dry load), Celite was added (0.3 g) and the reaction mixture with Celite was evaporated. The obtained crude product was purified *via* flash-chromatography (Interchim devices puriFLASH 4125) on silica gel (PF-15SIHP-F0012) using cyclohexane/ethyl acetate with 0% to 50% ethyl acetate in 15 column volumes. The isolated product 3-(4-butyl-1*H*-1,2,3-triazol-1-yl)-1-isopropyl-1*H*-pyrazole-4-carbonitrile (64.5 mg, 250 µmol) was obtained as a colourless solid in 69% yield.

R_f = 0.46 (cyclohexane/ethyl acetate 1:1). MP = 48.6–49.4 °C. ^{1}H NMR (400 MHz, Chloroform-d [7.27 ppm], ppm) δ = 8.00 (s, 1H), 7.91 (s, 1H), 4.54 (sept, *J* = 6.7 Hz, 1H), 2.77 (t, *J* = 7.6 Hz, 2H), 1.69 (quint, *J* = 7.5 Hz, 2H), 1.55 (d, *J* = 6.6 Hz, 6H), 1.39 (h, *J* = 7.3 Hz, 2H), 0.92 (t, *J* = 7.3 Hz, 3H); ^{13}C NMR (100 MHz, Chloroform-d [77.0 ppm], ppm) δ = 148.6 (C_q), 145.9 (C_q), 134.3 (CH), 119.0 (CH), 111.6 (C_q), 84.1 (C_q), 55.8 (CH), 31.2 (CH_2), 25.0 (CH_2), 22.3 (CH_3, 2C), 22.1 (CH_2), 13.7 (CH_3); MS (EI, 70 eV, 130 °C), m/z (%): 259 (1) $[M]^+$, 229 (11), 215 (11), 187 (100), 173 (12), 145 (54), 119 (14), 64 (10). HRMS–EI ($C_{13}H_{18}N_6$) *(m/z)*: $[M]^+$ Calcd 258.1587; Found 258.1587; IR (ATR, ṽ) = 3132 (m), 3105 (w), 3081 (w), 2980 (w), 2962 (m), 2928 (m), 2876 (w), 2859 (w), 2235 (s), 1557 (vs), 1534 (vs), 1466 (w), 1453 (m), 1441 (w), 1398 (w), 1375 (w), 1358 (w), 1343 (s), 1261 (w), 1228 (s), 1220 (m), 1183 (vs), 1156 (m), 1129 (w), 1099 (w), 1061 (w), 1048 (s), 1037 (vs), 1014 (w), 997 (s), 986 (s), 931 (w), 897 (w), 885 (w), 867 (s), 840 (s), 790 (w), 732 (w), 694 (s), 650 (m), 640 (w), 629 (w), 557 (w), 511 (w), 497 (w), 477 (w), 436 (w), 401 (w) cm^{-1}.

Additional information on the chemical synthesis is available *via* Chemotion repository: https://doi.org/10.14272/reaction/SA-FUHFF-UHFFFADPSC-NOJZURFWHM-UHFFFADPSC-NUHFF-NUHFF-NUHFF-ZZZ

Additional information on the analysis of the target compound is available *via* Chemotion repository:
https://doi.org/10.14272/NOJZURFWHMPYCF-UHFFFAOYSA-N.1

1-Isopropyl-3-(4-phenyl-1*H*-1,2,3-triazol-1-yl)-1*H*-pyrazole-4-carbonitrile (**21pd**)

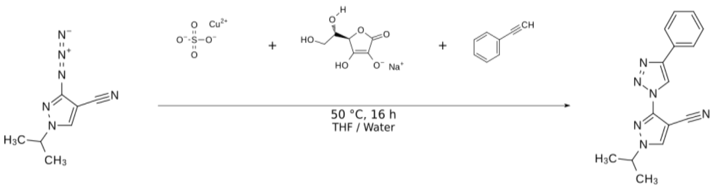

Name {P1|**21pd**}: 1-isopropyl-3-(4-phenyl-1*H*-1,2,3-triazol-1-yl)-1*H*-pyrazole-4-carbonitrile; Formula: $C_{15}H_{14}N_6$; Smiles: N#Cc1cn(nc1n1nnc(c1)c1ccccc1)C(C)C. InChIKey: OYRDJONMMXESTQ-UHFFFAOYSA-N

In a vial, 3-azido-1-propan-2-yl-pyrazole-4-carbonitrile (50.0 mg, 284 µmol, 1.00 equiv) was dissolved in THF (6.00 mL) and water (6.00 mL) (1:1) and copper;sulfate (9.06 mg, 56.8 µmol, 0.20 equiv), sodium ascorbate (56.2 mg, 284 µmol, 1.00 equiv) and ethynylbenzene (37.7 mg, 40.5 µL, 369 µmol, 1.30 equiv) were added. The reaction mixture was stirred at 50 °C for 16 hours. For workup, the reaction mixture was diluted in water and extracted three times with methylene chloride. The combined organic phases were washed with water and brine, dried over Na_2SO_4, filtered and the solvent was removed under reduced pressure. As preparation for column chromatography (dry load), Celite was added (0.2 g) and the reaction mixture with Celite was evaporated. The obtained crude product was purified *via* flash-chromatography (Interchim devices puriFLASH 4125) on silica gel (PF-15SIHP-F0012) using cyclohexane/ethyl acetate with 0% to 50% ethyl acetate in 15 column volumes. The isolated 1-isopropyl-3-(4-phenyl-1*H*-1,2,3-triazol-1-yl)-1*H*-pyrazole-4-carbonitrile (71.4 mg, 257 µmol) was obtained as a colorless solid in 90% yield.

R_f = 0.43 (cyclohexane/ethyl acetate 1:1). MP = 134.6–136.8 °C. ^{1}H NMR (400 MHz, Chloroform-d [7.27 ppm], ppm) δ = 8.39 (s, 1H), 8.00 (s, 1H), 7.92–7.89 (m, 2H), 7.47–7.43 (m, 2H), 7.39–7.34 (m, 1H), 4.57 (sept, *J* = 6.6 Hz, 1H), 1.59 (d, *J* = 6.6 Hz, 6H). ^{13}C NMR (100 MHz, Chloroform-d [77.0 ppm], ppm) δ = 147.8 (C_q), 145.6 (C_q), 134.4 (CH), 129.6 (C_q), 128.8 (CH, 2C), 128.6 (CH), 125.9 (CH, 2C), 117.6 (CH), 111.6 (C_q), 84.3 (C_q), 56.0 (CH), 22.3 (CH_3, 2C); MS (EI, 70 eV, 120 °C), m/z (%): 278 (6) $[M]^+$, 250 (75), 235 (32), 208 (100), 187 (13), 181 (20), 154 (12), 116 (24), 102 (14), 89 (19). HRMS–EI ($C_{15}H_{14}N_6$) *(m/z)*: $[M]^+$ Calcd 278.1274; Found 278.1277; IR (ATR, ṽ) = 3055 (w), 2983 (w), 2936 (w), 2234 (m), 1558 (m), 1538 (vs), 1480 (w), 1472 (w), 1452 (m), 1405 (w), 1384 (w), 1366 (w), 1353 (w), 1339 (w), 1283 (vw), 1234 (m), 1215 (w), 1184 (m), 1159 (w), 1135 (w), 1077 (w), 1038 (s), 1007 (s), 987 (w), 970 (w), 914 (w), 887 (w), 857 (w), 802 (w), 759 (vs), 704 (m), 687 (vs), 649 (m), 643 (m), 504 (m), 482 (w), 466 (w), 441 (w), 422 (w), 408 (w), 395 (w) cm^{-1}.

Additional information on the chemical synthesis is available *via* Chemotion repository: https://doi.org/10.14272/reaction/SA-FUHFF-UHFFFADPSC-OYRDJONMMX-UHFFFADPSC-NUHFF-NUHFF-NUHFF-ZZZ

Additional information on the analysis of the target compound is available *via* Chemotion repository: https://doi.org/10.14272/OYRDJONMMXESTQ-UHFFFAOYSA-N.1

Ethyl 2-(3-(4-butyl-1*H*-1,2,3-triazol-1-yl)-4-cyano-1*H*-pyrazol-1-yl)acetate (**21qa**)

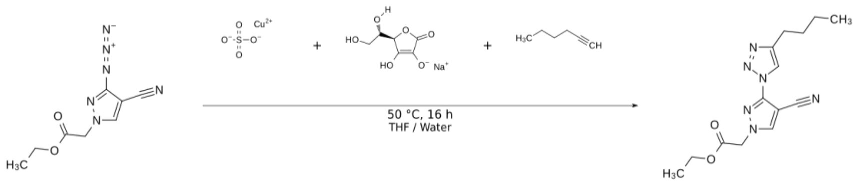

Name {P1|**21qa**}: ethyl 2-(3-(4-butyl-1*H*-1,2,3-triazol-1-yl)-4-cyano-1*H*-pyrazol-1-yl)acetate; Formula: $C_{14}H_{18}N_6O_2$; Smiles: CCCCc1nnn(c1)c1nn(cc1C#N)CC(=O)OCC. InChIKey: ZOQWDAUGOCUFLP-UHFFFAOYSA-N

In a vial, ethyl 2-(3-azido-4-cyano-1*H*-pyrazol-1-yl)acetate (100 mg, 454 µmol, 1.00 equiv) was dissolved in THF (9.00 mL) and water (9.00 mL) (1:1) and copper;sulfate (14.5 mg, 90.8 µmol, 0.20 equiv), sodium ascorbate (90.0 mg, 454 µmol, 1.00 equiv) and hex-1-yne (48.5 mg, 67.4 µL, 590 µmol, 1.30 equiv) were added. The reaction mixture was stirred at 50 °C for 16 hours. For work up the reaction mixture was diluted in water and extracted three times with methylene chloride. The combined organic phase was washed with water and brine, dried over Na_2SO_4 and filtered. As preparation for the column chromatography (dryload), Celite was added (0.3 g) and the reaction mixture with Celite were evaporated. The obtained crude product was purified *via* flash-chromatography (Interchim devices puriFLASH 5.125) on silica gel (PF-15SIHP-F0025) using cyclohexane/ethyl acetate 0% to 40% ethyl acetate in 20 column volumes (1 column volume = 63.8 mL; flow: 15 mL/min). The isolated product ethyl 2-(3-(4-butyl-1*H*-1,2,3-triazol-1-yl)-4-cyano-1*H*-pyrazol-1-yl)acetate (136 mg, 449 µmol) was obtained as a colorless solid in 99% yield.

R_f = 0.18 (cyclohexane/ethyl acetate 2:1). MP = 54.1–54.9 °C. ^{1}H NMR (400 MHz, Chloroform-d [7.27 ppm], ppm) δ = 8.07 (s, 1H), 7.92 (s, 1H), 4.97 (s, 2H), 4.30 (q, J = 7.1 Hz, 2H), 2.81 (t, J = 7.7 Hz, 2H), 1.76–1.68 (m, 2H), 1.42 (h, J = 7.3 Hz, 2H), 1.33 (t, J = 7.2 Hz, 3H), 0.96 (t, J = 7.3 Hz, 3H); ^{13}C NMR (100 MHz, Chloroform-d [77.0 ppm], ppm) δ = 165.9, 149.0, 146.6, 138.3, 119.0, 111.1, 86.2, 62.8, 53.9, 31.2, 25.1, 22.2, 14.1, 13.8; MS (FAB, 3-NBA), m/z (%): 303 (100) $[M+1]^+$, HRMS–FAB ($C_{14}H_{19}O_2N_6$)*(m/z)*: $[M+H]^+$ Calcd 303.1564; Found 303.1566; IR (ATR, ṽ) = 3139 (vw), 2958 (w), 2932 (w), 2861 (w), 2241 (m), 1748 (vs), 1557 (vs), 1543 (vs), 1463 (m), 1421 (w), 1395 (w), 1374 (s), 1353 (w), 1298 (w), 1265 (m), 1213 (vs), 1166 (vs), 1096 (m), 1034 (vs), 1024 (vs), 1010 (vs), 987 (s), 972 (m), 935 (w), 873 (m), 790 (s), 754 (m), 730 (m), 696 (s), 645 (m), 622 (m), 572 (m), 511 (m), 442 (m), 401 (m), 385 (m), 377 (m) cm^{-1}.

Additional information on the chemical synthesis is available *via* Chemotion repository: https://doi.org/10.14272/reaction/SA-FUHFF-UHFFFADPSC-ZOQWDAUGOC-UHFFFADPSC-NUHFF-NUHFF-NUHFF-ZZZ

Additional information on the analysis of the target compound is available *via* Chemotion repository:
https://doi.org/10.14272/ZOQWDAUGOCUFLP-UHFFFAOYSA-N.1

1-Benzyl-3-(4-phenyl-1*H*-1,2,3-triazol-1-yl)-*1H*-pyrazole-4-carbonitrile (**21rd**)

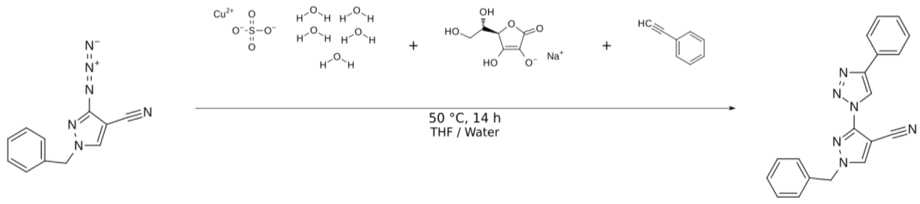

Name {P1|**rd**}: 1-benzyl-3-(4-phenyl-1*H*-1,2,3-triazol-1-yl)-1*H*-pyrazole-4-carbonitrile; Formula: $C_{19}H_{14}N_6$; CAS: - ; Molecular Mass: 326.3547; Exact Mass: 326.1280; EA: C, 69.92; H, 4.32; N, 25.75. Smiles: N#Cc1cn(nc1n1nnc(c1)c1ccccc1)Cc1ccccc1. InChIKey: DGWASOOTEXXJBK-UHFFFAOYSA-N

In a vial, 3-azido-1-benzyl-1*H*-pyrazole-4-carbonitrile (24.9 mg, 111 µmol, 1.00 equiv) was dissolved in THF (3.33 mL) and water (3.33 mL) (1:1) and copper sulfate pentahydrate (5.55 mg, 22.2 µmol, 0.200 equiv), sodium ascorbate (22.0 mg, 111 µmol, 1.00 equiv) and ethynylbenzene (68.1 mg, 73.2 µL, 167 µmol, 1.50 equiv) were added. The reaction mixture was stirred at 50 °C for 14 hours. For work up the reaction mixture was diluted in water and extracted three times with methylene chloride. The combined organic phase was washed with water and brine, dried over Na_2SO_4 and filtered. As preparation for the column chromatography (dryload), Celite was added (0.3 g) and the reaction mixture with Celite were evaporated. The obtained crude product was purified *via* flash-chromatography on silica gel using cyclohexane/ethyl acetate. The isolated product 1-benzyl-3-(4-phenyl-1*H*-1,2,3-triazol-1-yl)-1*H*-pyrazole-4-carbonitrile (33.5 mg, 103 µmol) was obtained as a colorless solid in 92% yield.

^1H NMR (400 MHz, Chloroform-d [7.26 ppm], ppm) δ = 8.38 (s, 1H), 7.92–7.90 (m, 2H), 7.87 (s, 1H), 7.48–7.33 (m, 8H), 5.36 (s, 2H). ^{13}C NMR (100 MHz, Chloroform-d [77.16 ppm], ppm) δ = 148.2 (C_q), 146.3 (C_q), 136.8 (CH), 133.5 (C_q), 129.7 (C_q), 129.5 (2C, CH), 129.5 (CH), 129.1 (2C, CH), 128.8 (CH), 128.6 (2C, CH), 126.2 (2C, CH), 117.7 (CH), 111.4 (C_q), 85.6 (C_q), 57.6 (CH_2); MS (EI, 70 eV, 17 °C), m/z (%): 326 (12) $[M]^+$, 298 (92), 181 (17), 131 (15), 91 (100), 69 (19). HRMS–EI ($C_{19}H_{14}N_6$) *(m/z)*: $[M]^+$ Calcd 326.1280; Found 326.1281; IR (ATR, $\tilde{\nu}$) = 3148, 3114, 3041, 2927, 2854, 2791, 2323, 2240, 2166, 1779, 1652, 1605, 1560, 1541, 1494, 1476, 1455, 1397, 1363, 1337, 1307, 1286, 1265, 1234, 1218, 1205, 1184, 1143, 1095, 1078, 1037, 1014, 992, 928, 857, 827, 802, 769, 730, 700, 688, 653, 640, 616, 592, 558, 530, 514, 500, 483, 466, 446, 399, 387, 375 cm^{-1}.

Additional information on the chemical synthesis is available *via* Chemotion repository: https://doi.org/10.14272/reaction/SA-FUHFF-UHFFFADPSC-DGWASOOTEX-UHFFFADPSC-NUHFF-NUHFF-NUHFF-ZZZ

Additional information on the analysis of the target compound is available *via* Chemotion repository:
https://doi.org/10.14272/DGWASOOTEXXJBK-UHFFFAOYSA-N.1

3-(4-Butyl-1*H*-1,2,3-triazol-1-yl)-1-(3,5-difluorobenzyl)-1*H*-pyrazole-4-carbonitrile (21sa)

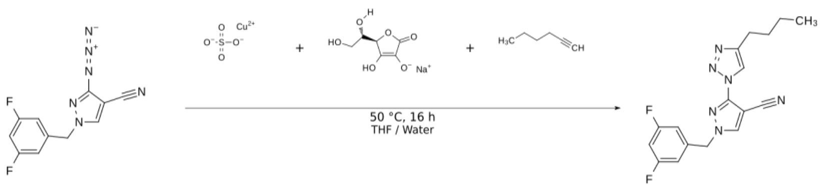

Name {P1|**21sa**}: 3-(4-butyl-1*H*-1,2,3-triazol-1-yl)-1-(3,5-difluorobenzyl)-1*H*-pyrazole-4-carbonitrile; Formula: $C_{17}H_{16}F_2N_6$; Smiles: CCCCc1nnn(c1)c1nn(cc1C#N)Cc1cc(F)cc(c1)F. InChIKey: APGSEIDPRRBFBQ-UHFFFAOYSA-N

In a vial, 3-azido-1-(3,5-difluorobenzyl)-1*H*-pyrazole-4-carbonitrile (200 mg, 769 µmol, 1.00 equiv) was dissolved in THF (18.00 mL) and water (18.00 mL) (1:1) and copper;sulfate (24.5 mg, 154 µmol, 0.20 equiv), sodium ascorbate (152 mg, 769 µmol, 1.00 equiv) and hex-1-yne (82.1 mg, 114 µL, 999 µmol, 1.30 equiv) were added. The reaction mixture was stirred at 50 °C for 16 hours. For work up the reaction mixture was diluted in water and extracted three times with methylene chloride. The combined organic phase was washed with water and brine, dried over Na_2SO_4 and filtered. As preparation for the column chromatography (dryload), Celite was added (0.6 g) and the reaction mixture with Celite were evaporated. The obtained crude product was purified *via* flash-chromatography (Interchim devices puriFLASH 5.125) on silica gel (PF-15SIHP-F0040) using cyclohexane/ethyl acetate 0% to 35% ethyl acetate in 12 column volumes (1 column volume = 91.9 mL; flow: 26 mL/min). The isolated product 3-(4-butyl-1*H*-1,2,3-triazol-1-yl)-1-(3,5-difluorobenzyl)-1*H*-pyrazole-4-carbonitrile (253 mg, 739 µmol) was obtained as a colorless solid in 96% yield.

R_f = 0.65 (cyclohexane/ethyl acetate 2:1). MP = 103.3–103.8 °C. ^{1}H NMR (400 MHz, Chloroform-d [7.27 ppm], ppm) δ = 8.06 (s, 1H), 7.92 (s, 1H), 6.87–6.80 (m, 3H), 5.35 (s, 2H), 2.80 (t, *J* = 7.7 Hz, 2H), 1.75–1.68 (m, 2H), 1.42 (h, *J* = 7.4 Hz, 2H), 0.95 (t, *J* = 7.3 Hz, 3H); ^{13}C NMR (100 MHz, Chloroform-d [77.0 ppm], ppm) δ = 163.4 (d, *J* = 251.2 Hz), 163.2 (d, *J* = 251.2 Hz), 149.0, 146.8, 137.4 (t, *J* = 9.2 Hz), 137.1, 119.0, 111.2, 111.1 (dd, *J* = 7.7 Hz, *J* = 19.3 Hz, 2C), 104.6 (t, *J* = 25.0 Hz), 85.7, 56.2 (t, *J* = 1.9 Hz), 31.2, 25.1, 22.2, 13.7; ^{19}F NMR (376 MHz, ppm) δ = -107.51; MS (FAB, 3-NBA), m/z (%): 343 (100) $[M+1]^+$, 154 (18), 136 (15), 127 (17). HRMS–FAB ($C_{17}H_{17}N_6F_2$) *(m/z)*: $[M+H]^+$ Calcd 343.1477; Found 343.1479; IR (ATR, ṽ) = 3121 (w), 3101 (w), 3055 (w), 2956 (w), 2931 (w), 2873 (w), 2861 (w), 2241 (m), 1626 (m), 1601 (s), 1557 (s), 1538 (vs), 1468 (m), 1459 (s), 1436 (s), 1404 (w), 1375 (w), 1366 (w), 1357 (w), 1316 (s), 1276 (w), 1249 (w), 1221 (m), 1164 (w), 1143 (w), 1118 (vs), 1034 (vs), 1007 (vs), 1001 (vs), 982 (m), 942 (m), 890 (m), 868 (vs), 854 (m), 844 (m), 799

(w), 778 (vs), 755 (w), 704 (m), 693 (vs), 657 (s), 640 (w), 575 (m), 531 (w), 510 (s) cm^{-1}.

Additional information on the chemical synthesis is available *via* Chemotion repository: https://doi.org/10.14272/reaction/SA-FUHFF-UHFFFADPSC-APGSEIDPRR-UHFFFADPSC-NUHFF-NUHFF-NUHFF-ZZZ

Additional information on the analysis of the target compound is available *via* Chemotion repository: https://doi.org/10.14272/APGSEIDPRRBFBQ-UHFFFAOYSA-N.1

1-(3,5-Difluorobenzyl)-3-(4-phenyl-1*H*-1,2,3-triazol-1-yl)-1*H*-pyrazole-4-carbonitrile (21sd)

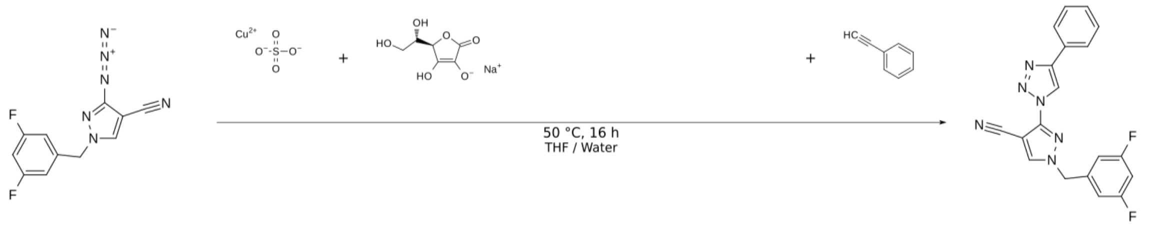

Name {P1|**21sd**}: 1-(3,5-difluorobenzyl)-3-(4-phenyl-1*H*-1,2,3-triazol-1-yl)-1*H*-pyrazole-4-carbonitrile; Formula: $C_{19}H_{12}F_2N_6$; Smiles: N#Cc1cn(nc1n1nnc(c1)c1ccccc1)Cc1cc(F)cc(c1)F. InChIKey: LBYPTGDZZSOCSK-UHFFFAOYSA-N

In a vial, 3-azido-1-(3,5-difluorobenzyl)-1*H*-pyrazole-4-carbonitrile (25.0 mg, 96.1 µmol, 1.00 equiv) was dissolved in THF (5.00 mL) and water (5.00 mL) (1:1) and copper;sulfate (3.07 mg, 19.2 µmol, 0.200 equiv), sodium ascorbate (19.0 mg, 96.1 µmol, 1.00 equiv) and ethynylbenzene (51.0 mg, 54.9 µL, 125 µmol, 1.30 equiv) were added. The reaction mixture was stirred at 50 °C for 16 hours. For work-up, the reaction mixture was diluted in water and extracted three times with methylene chloride. The combined organic phase was washed with water and brine, dried over Na_2SO_4 and filtered. As preparation for the column chromatography (dryload), Celite was added (0.1 g) and the reaction mixture with Celite was evaporated. The obtained crude product was purified *via* flash-chromatography on silica gel using cyclohexane/ethyl acetate 20:1 to 4:1. The isolated product 1-(3,5-difluorobenzyl)-3-(4-phenyl-1*H*-1,2,3-triazol-1-yl)-1*H*-pyrazole-4-carbonitrile (27.5 mg, 75.9 µmol) was obtained as a colorless oil in 79% yield.

R_f = 0.57 (cyclohexane/ethyl acetate 1:1). ^{1}H NMR (400 MHz, Chloroform-d [7.27 ppm], ppm) δ = 8.39 (s, 1H), 8.05 (s, 1H), 7.92–7.90 (m, 2H), 7.49–7.45 (m, 2H), 7.41–7.37 (m, 1H), 6.88–6.83 (m, 3H), 5.37 (s, 2H). ^{13}C NMR (100 MHz, Chloroform-d [77.0 ppm], ppm) δ = 163.3 (dd, *J* = 12.3 Hz, *J* = 251.2 Hz, 2C), 148.2, 146.5, 137.1, 137.2, 137.1, 129.4, 129.0 (2C), 128.8, 126.0 (2C), 117.5, 111.1 (dd, *J* = 7.7 Hz, *J* = 18.5 Hz, 2C), 104.7 (t, *J* = 25.0 Hz), 86.0, 56.3 (t, *J* = 1.9 Hz); ^{19}F NMR (376 MHz, ppm) δ = -107.33; MS (FAB, 3-NBA): *m/z* (%) = 363 (100) [M+H]$^+$, 334 (11), 155 (11), 154 (32), 147 (11), 138 (17), 137 (24), 136 (24), 127 (13), 109 (12), 97 (13), 95 (17), 91 (18). HRMS–FAB *(m/z)*: [M+H]$^+$ Calcd for $C_{19}H_{13}N_6F_2$, 363.1170; Found, 363.1170; IR (ATR, ṽ) = 3150 (w), 3105 (w), 3085 (w), 3053 (w), 3019 (w), 3000 (w), 2958 (w), 2922 (w), 2853 (w), 2238 (w), 1625 (m), 1596 (s), 1560 (s), 1543 (vs), 1482 (w), 1462 (s), 1449 (s), 1435

(s), 1404 (w), 1361 (w), 1351 (w), 1340 (w), 1315 (s), 1282 (w), 1239 (m), 1205 (w), 1179 (w), 1140 (w), 1119 (vs), 1071 (w), 1041 (s), 1017 (vs), 1004 (m), 989 (s), 941 (w), 911 (w), 888 (w), 873 (w), 858 (w), 847 (vs), 810 (m), 790 (w), 778 (s), 759 (vs), 704 (m), 690 (vs), 664 (s), 643 (w), 629 (w), 616 (w), 591 (w), 579 (s), 535 (w), 530 (w), 509 (s), 497 (m), 482 (w), 441 (w), 402 (w), 375 (w) cm^{-1}.

Additional information on the chemical synthesis is available *via* Chemotion repository: https://doi.org/10.14272/reaction/SA-FUHFF-UHFFFADPSC-LBYPTGDZZS-UHFFFADPSC-NUHFF-NUHFF-NUHFF-ZZZ

Additional information on the analysis of the target compound is available *via* Chemotion repository:
https://doi.org/10.14272/LBYPTGDZZSOCSK-UHFFFAOYSA-N.1

3-(4-Butyl-1*H*-1,2,3-triazol-1-yl)-1-(4-methylbenzyl)-1*H*-pyrazole-4-carbonitrile (21ta)

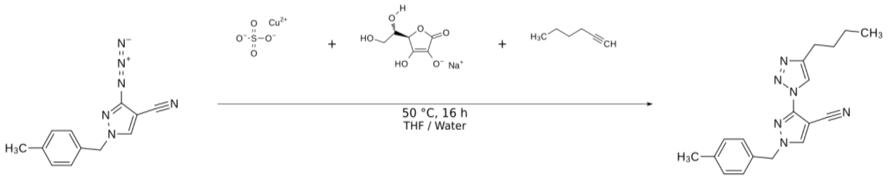

Name {P1|**21ta**}: 3-(4-butyl-1*H*-1,2,3-triazol-1-yl)-1-(4-methylbenzyl)-1*H*-pyrazole-4-carbonitrile; Formula: $C_{18}H_{20}N_6$; Smiles: CCCCc1nnn(c1)c1nn(cc1C#N)Cc1ccc(cc1)C. InChIKey: FGLOKNGZLSDWMZ-UHFFFAOYSA-N

In a vial, 3-azido-1-(4-methylbenzyl)-1*H*-pyrazole-4-carbonitrile (100 mg, 420 µmol, 1.00 equiv) was dissolved in THF (8.00 mL) and water (8.00 mL) (1:1) and copper;sulfate (13.4 mg, 83.9 µmol, 0.20 equiv), sodium ascorbate (83.2 mg, 420 µmol, 1.00 equiv) and hex-1-yne (44.8 mg, 62.3 µL, 546 µmol, 1.30 equiv) were added. The reaction mixture was stirred at 50 °C for 16 hours. For workup, the reaction mixture was diluted in water and extracted three times with methylene chloride. The combined organic phases were washed with water and brine, dried over Na_2SO_4, filtered and the solvent was removed under reduced pressure. As preparation for column chromatography (dry load), Celite was added (0.3 g) and the reaction mixture with Celite was evaporated. The obtained crude product was purified *via* flash-chromatography (Interchim devices puriFLASH 5.125) on silica gel (PF-15SIHP-F0012) using cyclohexane/ethyl acetate with 0% to 30% ethyl acetate in 15 column volumes. The isolated product 3-(4-butyl-1*H*-1,2,3-triazol-1-yl)-1-(4-methylbenzyl)-1*H*-pyrazole-4-carbonitrile (121 mg, 376 µmol) was obtained as a colourless solid in 90% yield.

R_f = 0.30 (cyclohexane/ethyl acetate 2:1). MP = 89–89.6 °C. ^{1}H NMR (400 MHz, Chloroform-d [7.27 ppm], ppm) δ = 7.91 (t, *J* = 0.7 Hz, 1H), 7.81 (s, 1H), 7.22 (s, 4H), 5.29 (s, 2H), 2.80 (t, *J* = 7.5 Hz, 2H), 2.38 (s, 3H), 1.75–1.68 (m, 2H), 1.47–1.37 (m, 2H), 0.95 (t, *J* = 7.3 Hz, 3H). ^{13}C NMR (100 MHz, Chloroform-d [77.0 ppm], ppm) δ = 148.8 (C_q), 146.4 (C_q), 139.3 (C_q), 136.2 (CH), 130.2 (C_q), 130.0 (CH, 2C), 128.5 (CH, 2C), 119.0 (CH), 111.4 (C_q), 85.1 (C_q), 57.2 (CH_2), 31.2 (CH_2), 25.1 (CH_2), 22.2 (CH_2), 21.2 (CH_3), 13.8 (CH_3). MS (FAB, 3-NBA), m/z (%): 321 (100) $[M+1]^+$, 105 (66).

HRMS–FAB ($C_{18}H_{21}N_6$) *(m/z)*: $[M+H]^+$ Calcd 321.1822; Found 321.1821; IR (ATR, ṽ) = 3159 (w), 3123 (w), 2953 (m), 2929 (m), 2870 (w), 2237 (s), 1615 (vw), 1555 (vs), 1540 (vs), 1516 (s), 1466 (w), 1455 (m), 1436 (m), 1429 (m), 1401 (w), 1380 (w), 1367 (w), 1346 (m), 1309 (w), 1300 (w), 1275 (w), 1247 (w), 1220 (m), 1204 (w), 1171 (w), 1154 (vs), 1112 (w), 1031 (vs), 1009 (m), 982 (s), 959 (w), 933 (w), 851 (w), 829 (s), 802 (w), 790 (w), 768 (vs), 754 (s), 735 (w), 694 (s), 671 (w), 642 (s), 581 (m), 521 (w), 501 (m), 473 (m), 433 (w), 390 (w), 380 (w) cm^{-1}.

Additional information on the chemical synthesis is available *via* Chemotion repository: https://doi.org/10.14272/reaction/SA-FUHFF-UHFFFADPSC-FGLOKNGZLS-UHFFFADPSC-NUHFF-NUHFF-NUHFF-ZZZ

Additional information on the analysis of the target compound is available *via* Chemotion repository:
https://doi.org/10.14272/FGLOKNGZLSDWMZ-UHFFFAOYSA-N.1

1-(4-Methylbenzyl)-3-(4-phenyl-1*H*-1,2,3-triazol-1-yl)-1*H*-pyrazole-4-carbonitrile (**21td**)

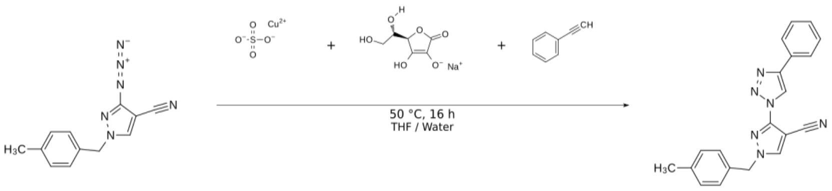

Name {P1|**21td**}: 1-(4-methylbenzyl)-3-(4-phenyl-1*H*-1,2,3-triazol-1-yl)-1*H*-pyrazole-4-carbonitrile; Formula: $C_{20}H_{16}N_6$; Smiles: N#Cc1cn(nc1n1nnc(c1)c1ccccc1)Cc1ccc(cc1)C. InChIKey: ZHUFVXOCSYMTKK-UHFFFAOYSA-N

In a vial, 3-azido-1-(4-methylbenzyl)-1*H*-pyrazole-4-carbonitrile (96.4 mg, 405 µmol, 1.00 equiv) was dissolved in THF (8.00 mL) and water (8.00 mL) (1:1) and copper;sulfate (12.9 mg, 80.9 µmol, 0.20 equiv), sodium ascorbate (80.2 mg, 405 µmol, 1.00 equiv) and ethynylbenzene (53.7 mg, 57.8 µL, 526 µmol, 1.30 equiv) were added. The reaction mixture was stirred at 50 °C for 16 hours. For work up the reaction mixture was diluted in water and extracted three times with methylene chloride. The combined organic phase was washed with water and brine, dried over Na_2SO_4, filtered and the solvent was removed under reduced pressure. As preparation for the column chromatography (dryload), Celite was added (0.3 g) and the reaction mixture with Celite were evaporated. The obtained crude product was purified *via* flash-chromatography (Interchim devices puriFLASH 5.125) on silica gel (PF-15SIHP-F0025) using cyclohexane/ethyl acetate 0% to 50% ethyl acetate in 17 column volumes. The isolated 1-(4-methylbenzyl)-3-(4-phenyl-1*H*-1,2,3-triazol-1-yl)-1*H*-pyrazole-4-carbonitrile (100 mg, 294 µmol) was obtained as a colorless solid in 73% yield.

R_f = 0.62 (cyclohexane/ethyl acetate 1:1). MP = 191.9–208.5 °C. ^1H NMR (400 MHz, Chloroform-d [7.27 ppm], ppm) δ = 8.40 (s, 1H), 7.94–7.91 (m, 2H), 7.82 (s, 1H), 7.49–7.45 (m, 2H), 7.41–7.37 (m, 1H), 7.25 (s, 4H), 5.32 (s, 2H), 2.39 (s, 3H); ^{13}C NMR (100 MHz, Chloroform-d [77.0 ppm], ppm) δ = 148.1, 146.2, 139.5, 136.3, 130.1, 130.1 (2C),

129.6, 128.9 (2C), 128.7, 128.5 (2C), 126.1 (2C), 117.5, 111.2, 85.4, 57.3, 21.2; MS (FAB, 3-NBA), m/z (%): 341 (32) [M+1]$^{+}$, 219 (15), 191 (16), 175 (10), 165 (14), 154 (31), 147 (33), 136 (33), 119 (41), 115 (31), 105 (68), 91 (100). HRMS–FAB ($C_{20}H_{17}N_6$) *(m/z)*: [M+H]$^{+}$ Calcd 341.1509; Found 341.1508. IR (ATR, ṽ) = 3143 (w), 2917 (w), 2853 (w), 2237 (m), 1655 (w), 1615 (w), 1557 (m), 1537 (s), 1514 (m), 1480 (m), 1470 (m), 1451 (m), 1419 (w), 1394 (w), 1360 (w), 1327 (m), 1307 (w), 1296 (w), 1231 (m), 1218 (w), 1204 (w), 1187 (w), 1142 (s), 1112 (w), 1091 (w), 1074 (w), 1035 (m), 1024 (w), 1013 (m), 990 (m), 965 (w), 953 (w), 948 (w), 921 (w), 846 (w), 830 (s), 810 (m), 759 (vs), 704 (m), 688 (vs), 639 (m), 619 (s), 578 (s), 518 (m), 501 (s), 482 (s), 442 (m), 399 (m), 384 (w) cm^{-1}.

Additional information on the chemical synthesis is available *via* Chemotion repository: https://doi.org/10.14272/reaction/SA-FUHFF-UHFFFADPSC-ZHUFVXOCSY-UHFFFADPSC-NUHFF-NUHFF-NUHFF-ZZZ

Additional information on the analysis of the target compound is available *via* Chemotion repository:
https://doi.org/10.14272/ZHUFVXOCSYMTKK-UHFFFAOYSA-N.1

Methyl 4-(1-(4-cyano-1-(4-methylbenzyl)-1*H*-pyrazol-3-yl)-1*H*-1,2,3-triazol-4-yl)benzoate (21tf)

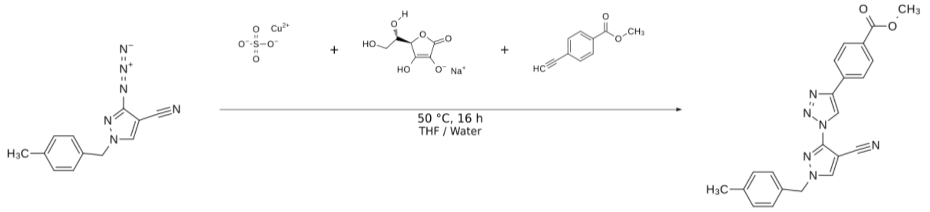

Name {P1|**21tf**}: methyl 4-(1-(4-cyano-1-(4-methylbenzyl)-1*H*-pyrazol-3-yl)-1*H*-1,2,3-triazol-4-yl)benzoate; Formula: $C_{22}H_{18}N_6O_2$; Smiles: COC(=O)c1ccc(cc1)c1nnn(c1)c1nn(cc1C#N)Cc1ccc(cc1)C. InChIKey: DMRAVMAMSLYEBQ-UHFFFAOYSA-N

In a vial, 3-azido-1-(4-methylbenzyl)-1*H*-pyrazole-4-carbonitrile (56.3 mg, 236 µmol, 1.00 equiv) was dissolved in THF (4.00 mL) and water (4.00 mL) (1:1) and copper;sulfate (7.54 mg, 47.3 µmol, 0.20 equiv), sodium ascorbate (46.8 mg, 236 µmol, 1.00 equiv) and methyl 4-ethynylbenzoate (49.2 mg, 307 µmol, 1.30 equiv) were added. The reaction mixture was stirred at 50 °C for 16 hours. For workup, the reaction mixture was diluted in water and extracted three times with methylene chloride. The combined organic phases were washed with water and brine, dried over Na_2SO_4, filtered and the solvent was removed under reduced pressure. As preparation for column chromatography (dry load), Celite was added (0.2 g) and the reaction mixture with Celite was evaporated. The obtained crude product was purified *via* flash-chromatography (Interchim devices puriFLASH 5.125) on silica gel (PF-15SIHP-F0012) using cyclohexane/ethyl acetate with 0% to 50% ethyl acetate in 15 column volumes. The isolated product methyl 4-(1-(4-cyano-1-(4-methylbenzyl)-1*H*-pyrazol-3-

yl)-1*H*-1,2,3-triazol-4-yl)benzoate (88.3 mg, 222 µmol) was obtained as colorless solid in 94% yield.

R_f = 0.32 (cyclohexane/ethyl acetate 1:1). MP = 259.1–265.1 °C. ^{1}H NMR (400 MHz, Chloroform-d [7.27 ppm], ppm) δ = 8.48 (s, 1H), 8.16–8.14 (m, 2H), 8.02–8.00 (m, 2H), 7.82 (s, 1H), 7.25 (s, 4H), 5.32 (s, 2H), 3.96 (s, 3H), 2.40 (s, 3H). ^{13}C NMR (100 MHz, Chloroform-d [77.0 ppm], ppm) δ = 166.7 (C_q), 147.1 (C_q), 146.0 (C_q), 139.6 (C_q), 136.4 (CH), 133.9 (C_q), 130.3 (CH, 2C), 130.2 (C_q), 130.1 (CH, 2C), 130.0 (C_q), 128.6 (CH, 2C), 125.9 (CH, 2C), 118.4 (CH), 85.6 (C_q), 77.2 (C_q), 57.4 (CH_2), 52.2 (CH_3), 21.2 (CH_3); MS (FAB, 3-NBA), m/z (%): 399 (7) $[M+H]^+$, 307 (29), 289 (12), 154 (100), 136 (67), 120 (11), 107 (21), 91 (16). HRMS–FAB ($C_{22}H_{19}O_2N_6$) *(m/z)*: $[M+H]^+$ Calcd 399.1564; Found 399.1562; IR (ATR, ṽ) = 3131 (w), 2237 (m), 1703 (s), 1612 (w), 1555 (s), 1544 (s), 1514 (w), 1483 (w), 1435 (m), 1418 (w), 1392 (w), 1364 (vw), 1339 (w), 1306 (vw), 1276 (s), 1245 (s), 1204 (m), 1190 (w), 1173 (m), 1145 (s), 1105 (s), 1088 (s), 1028 (m), 1009 (s), 989 (m), 959 (m), 874 (m), 863 (m), 853 (m), 824 (m), 798 (w), 764 (vs), 710 (s), 691 (s), 636 (w), 622 (w), 586 (s), 526 (w), 510 (m), 503 (m), 482 (s), 402 (w) cm^{-1}.

Additional information on the chemical synthesis is available *via* Chemotion repository: https://doi.org/10.14272/reaction/SA-FUHFF-UHFFFADPSC-DMRAVMAMSL-UHFFFADPSC-NUHFF-NUHFF-NUHFF-ZZZ

Additional information on the analysis of the target compound is available *via* Chemotion repository:
https://doi.org/10.14272/DMRAVMAMSLYEBQ-UHFFFAOYSA-N.1

3-(4-(4-(Dimethylamino)phenyl)-1*H*-1,2,3-triazol-1-yl)-1-(4-methylbenzyl)-1*H*-pyrazole-4-carbonitrile (21tg)

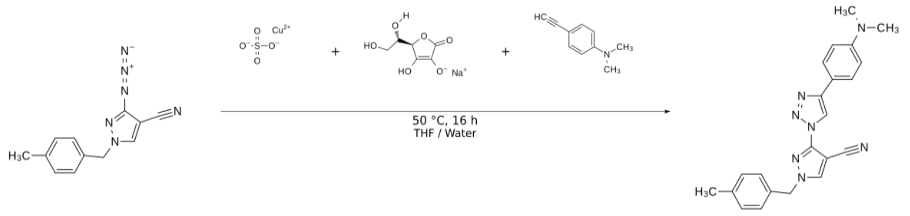

Name {P1|**21tg**}: 3-(4-(4-(dimethylamino)phenyl)-1*H*-1,2,3-triazol-1-yl)-1-(4-methylbenzyl)-1*H*-pyrazole-4-carbonitrile; Formula: $C_{22}H_{21}N_7$; Smiles: N#Cc1cn(nc1n1nnc(c1)c1ccc(cc1)N(C)C)Cc1ccc(cc1)C. InChIKey: PIHYCVSEWQEZEE-UHFFFAOYSA-N

In a vial, 3-azido-1-(4-methylbenzyl)-1*H*-pyrazole-4-carbonitrile (50.0 mg, 210 µmol, 1.00 equiv) was dissolved in THF (4.00 mL) and water (4.00 mL) (1:1) and copper;sulfate (6.70 mg, 42.0 µmol, 0.20 equiv), sodium ascorbate (41.6 mg, 210 µmol, 1.00 equiv) and 4-ethynyl-N,N-dimethylaniline (39.6 mg, 273 µmol, 1.30 equiv) were added. The reaction mixture was stirred at 50 °C for 16 hours. For workup, the reaction mixture was diluted in water and extracted three times with methylene chloride. The combined organic phases were washed with water and brine, dried over Na_2SO_4, filtered and the solvent was removed under reduced pressure. As preparation for the column chromatography (dry load), Celite was added (0.2 g) and the reaction mixture with Celite was evaporated. The obtained crude product was purified *via* flash-

chromatography (Interchim devices puriFLASH 5.125) on silica gel (PF-15SIHP-F0012) using cyclohexane/ethyl acetate with 0% to 50% ethyl acetate in 15 column volumes. The isolated product 3-(4-(4-(dimethylamino)phenyl)-1*H*-1,2,3-triazol-1-yl)-1-(4-methylbenzyl)-1*H*-pyrazole-4-carbonitrile (73.8 mg, 192 µmol) was obtained as light yellow solid in 92% yield.

R_f = 0.48 (cyclohexane/ethyl acetate 1:1). MP = 176.6–183.6 °C. ^{1}H NMR (400 MHz, Chloroform-d [7.27 ppm], ppm) δ = 8.22 (s, 1H), 7.82 (s, 1H), 7.79–7.75 (m, 2H), 7.22 (s, 4H), 6.79–6.76 (m, 2H), 5.29 (s, 2H), 3.00 (s, 6H), 2.37 (s, 3H). ^{13}C NMR (100 MHz, Chloroform-d [77.0 ppm], ppm) δ = 150.6 (C_q), 148.4 (C_q), 146.2 (C_q), 139.1 (C_q), 136.5 (CH), 130.4 (C_q), 129.9 (CH, 2C), 128.4 (CH, 2C), 126.9 (CH, 2C), 117.5 (C_q), 115.7 (CH), 112.3 (CH, 2C), 111.5 (C_q), 84.9 (C_q), 57.1 (CH_2), 40.3 (CH_3, 2C), 21.1 (CH_3). MS (EI, 70 eV, 220 °C), m/z (%): 383 (9) $[M]^+$, 355 (12), 255 (22), 251 (17), 250 (100), 159 (17), 144 (12), 105 (58). HRMS–EI ($C_{22}H_{21}N_7$) *(m/z)*: $[M]^+$ Calcd 383.1853; Found 383.1852; IR (ATR, ṽ) = 2944 (w), 2917 (w), 2887 (w), 2850 (w), 2806 (w), 2241 (w), 1618 (s), 1575 (w), 1560 (s), 1544 (s), 1502 (vs), 1483 (s), 1439 (m), 1431 (m), 1392 (w), 1354 (s), 1334 (s), 1298 (w), 1255 (w), 1231 (s), 1210 (w), 1197 (s), 1164 (m), 1142 (vs), 1119 (m), 1091 (w), 1060 (w), 1031 (vs), 1016 (s), 989 (s), 963 (m), 943 (m), 874 (w), 851 (m), 827 (vs), 805 (m), 778 (vs), 764 (vs), 755 (vs), 727 (w), 707 (w), 691 (vs), 642 (w), 619 (m), 606 (m), 575 (s), 528 (s), 503 (m), 482 (vs), 458 (w), 450 (w), 439 (w), 426 (w), 408 (w), 397 (w), 378 (w) cm^{-1}.

Additional information on the chemical synthesis is available *via* Chemotion repository: https://doi.org/10.14272/reaction/SA-FUHFF-UHFFFADPSC-PIHYCVSEWQ-UHFFFADPSC-NUHFF-NUHFF-NUHFF-ZZZ

Additional information on the analysis of the target compound is available *via* Chemotion repository:
https://doi.org/10.14272/PIHYCVSEWQEZEE-UHFFFAOYSA-N.1

3-(4-((1,3-Dioxoisoindolin-2-yl)methyl)-1*H*-1,2,3-triazol-1-yl)-1-(4-methylbenzyl)-1*H*-pyrazole-4-carbonitrile (21th)

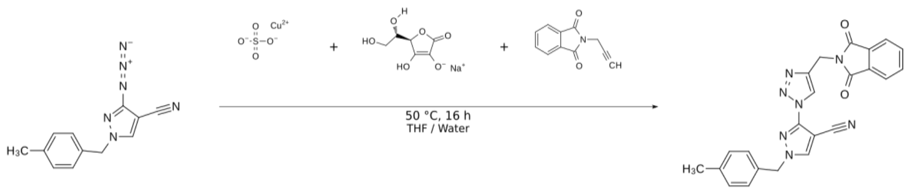

Name {P1|**21th**}: 3-(4-((1,3-dioxoisoindolin-2-yl)methyl)-1*H*-1,2,3-triazol-1-yl)-1-(4-methylbenzyl)-1*H*-pyrazole-4-carbonitrile; Formula: $C_{23}H_{17}N_7O_2$; Smiles: N#Cc1cn(nc1n1nnc(c1)CN1C(=O)c2c(C1=O)cccc2)Cc1ccc(cc1)C. InChIKey: FODYZQGXSSTMHJ-UHFFFAOYSA-N

In a vial, 3-azido-1-(4-methylbenzyl)-1*H*-pyrazole-4-carbonitrile (50.0 mg, 210 µmol, 1.00 equiv) was dissolved in THF (6.00 mL) and water (6.00 mL) (1:1) and copper;sulfate (6.70 mg, 42.0 µmol, 0.20 equiv), sodium ascorbate (41.6 mg, 210 µmol, 1.00 equiv) and 2-prop-2-ynylisoindole-1,3-dione (50.5 mg, 273 µmol, 1.30 equiv) were added. The reaction mixture was stirred at 50 °C for 16 hours. For workup, the reaction mixture was diluted in water and extracted three times with methylene chloride. The

combined organic phases were washed with water and brine, dried over Na_2SO_4, filtered and the solvent was removed under reduced pressure. As preparation for column chromatography (dry load), Celite was added (0.2 g) and the reaction mixture with Celite was evaporated. The obtained crude product was purified *via* flash-chromatography (Interchim devices puriFLASH 4125) on silica gel (PF-15SIHP-F0012) using cyclohexane/ethyl acetate with 0% to 50% ethyl acetate in 20 column volumes. The isolated product 3-(4-((1,3-dioxoisoindolin-2-yl)methyl)-1*H*-1,2,3-triazol-1-yl)-1-(4-methylbenzyl)-1*H*-pyrazole-4-carbonitrile (88.6 mg, 209 µmol) was obtained as a colorless solid in quantitative yield.

R_f = 0.34 (cyclohexane/ethyl acetate 1:1). MP = 165.2–227.3 °C. ^{1}H NMR (400 MHz, Chloroform-d [7.27 ppm], ppm) δ = 8.21 (s, 1H), 7.90 (s, 1H), 7.81 (dd, *J* = 5.5, 3.1 Hz, 2H), 7.69 (dd, *J* = 5.5, 3.1 Hz, 2H), 7.19–7.15 (m, 4H), 5.26 (s, 2H), 5.05 (s, 2H), 2.32 (s, 3H); ^{13}C NMR (100 MHz, Chloroform-d [77.0 ppm], ppm) δ = 167.4 (C_q, 2C), 145.7 (C_q), 143.0 (C_q), 139.0 (C_q), 136.7 (CH), 134.1 (CH, 2C), 131.8 (C_q, 2C), 130.3 (C_q), 129.7 (CH, 2C), 128.3 (CH, 2C), 123.3 (CH, 2C), 121.1 (CH), 111.1 (C_q), 85.1 (C_q), 57.1 (CH_2), 32.7 (CH_2), 21.0 (CH_3); MS (FAB, 3-NBA), m/z (%): 424 (100) $[M+1]^+$, 175 (11), 161 (14), 154 (39), 147 (13), 136 (33), 131 (10), 119 (10), 115 (10), 105 (67), 91 (26). HRMS–FAB ($C_{23}H_{18}O_2N_7$) *(m/z)*: $[M+H]^+$ Calcd 424.1516; Found 424.1516; IR (ATR, ṽ) = 3138 (w), 2240 (w), 1772 (w), 1713 (vs), 1613 (w), 1560 (m), 1540 (s), 1514 (w), 1465 (w), 1429 (s), 1394 (s), 1373 (vs), 1336 (m), 1323 (s), 1307 (m), 1285 (w), 1230 (m), 1203 (w), 1187 (w), 1176 (w), 1153 (s), 1111 (s), 1086 (w), 1071 (w), 1038 (s), 1006 (m), 989 (m), 970 (w), 948 (vs), 894 (w), 871 (m), 847 (w), 824 (w), 809 (m), 798 (w), 764 (s), 748 (vs), 713 (vs), 693 (s), 683 (m), 671 (w), 649 (m), 637 (m), 615 (w), 581 (m), 550 (w), 530 (s), 509 (w), 472 (s), 455 (w), 432 (w), 425 (w), 407 (w), 381 (w), 377 (w) cm^{-1}.

Additional information on the chemical synthesis is available *via* Chemotion repository: https://doi.org/10.14272/reaction/SA-FUHFF-UHFFFADPSC-FODYZQGXSS-UHFFFADPSC-NUHFF-NUHFF-NUHFF-ZZZ

Additional information on the analysis of the target compound is available *via* Chemotion repository:
https://doi.org/10.14272/FODYZQGXSSTMHJ-UHFFFAOYSA-N.1

Methyl 4-((4-cyano-3-(4-phenyl-1*H*-1,2,3-triazol-1-yl)-1*H*-pyrazol-1-yl)methyl)-benzoate (21ud)

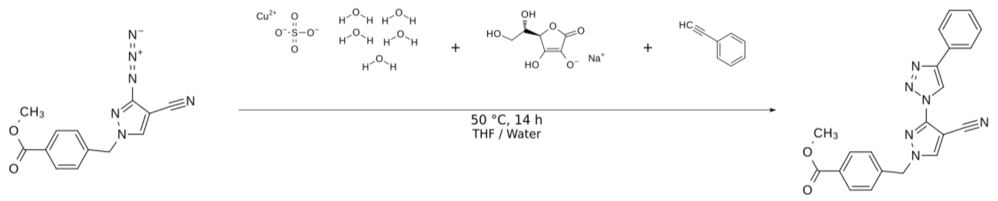

Name {P1|**21ud**}: methyl 4-((4-cyano-3-(4-phenyl-1*H*-1,2,3-triazol-1-yl)-1*H*-pyrazol-1-yl)methyl)benzoate; Formula: $C_{21}H_{16}N_6O_2$; Smiles: COC(=O)c1ccc(cc1)Cn1cc(c(n1)n1nnc(c1)c1ccccc1)C#N. InChIKey: YZSLXIHSIJQQMB-UHFFFAOYSA-N

In a vial, methyl 4-((3-azido-4-cyano-1*H*-pyrazol-1-yl)methyl)benzoate (36.0 mg, 128 µmol, 1.00 equiv) was dissolved in THF (3.33 mL) and water (3.33 mL) (1:1) and copper;sulfate;pentahydrate (6.37 mg, 6.25.5 µol, 0.200 equiv), sodium;(2R)-2-[(1S)-1,2-dihydroxyethyl]-3-hydroxy-5-oxo-2H-furan-4-olate (25.3 mg, 28 µmol, 1.00 equiv) and ethynylbenzene (78.2 mg, 84.0 µL, 191 µmol, 1.50 equiv) were added. The reaction mixture was stirred at 50 °C for 14 hours. For work-up, the reaction mixture was diluted in water and extracted three times with methylene chloride. The combined organic phase was washed with water and brine, dried over Na_2SO_4 and filtered. As preparation for the column chromatography (dryload), Celite was added and the reaction mixture with Celite was evaporated. The obtained crude product was purified *via* flash-chromatography on silica gel using cyclohexane/ethyl acetate. The isolated product methyl 4-((4-cyano-3-(4-phenyl-1*H*-1,2,3-triazol-1-yl)-1*H*-pyrazol-1-yl)methyl)benzoate (30.3 mg, 78.8 µmol) was obtained as a colorless solid in 62% yield.

R_f = 0.36 (cyclohexane/ethyl acetate 1:1). MP = 180–187.5 °C. ^{1}H NMR (400 MHz, Chloroform-d [7.26 ppm], ppm) δ = 8.37 (s, 1H), 8.10–8.07 (m, 2H), 7.96 (s, 1H), 7.92–7.89 (m, 2H), 7.48–7.44 (m, 2H), 7.40–7.38 (m, 3H), 5.42 (s, 2H), 3.93 (s, 3H); ^{13}C NMR (100 MHz, Chloroform-d [77.0 ppm], ppm) δ = 166.2 (C_q), 148.1 (C_q), 146.4 (C_q), 138.2 (C_q), 136.9 (CH), 131.0 (C_q), 130.5 (2C, CH), 129.5 (C_q), 128.9 (2C, CH), 128.8 (CH), 128.1 (2C, CH), 126.0 (2C, CH), 117.5 (CH), 111.1 (C_q), 85.8 (C_q), 57.0 (CH_2), 52.3 (CH_3); MS (FAB, 3-NBA), m/z (%): 385 (100) $[M+H]^+$, 356 (13), 165 (12), 154 (59), 149 (56), 136 (59), 121 (25), 109 (23), 107 (30), 105 (24), 95 (36), 91 (46). HRMS–FAB ($C_{21}H_{17}N_6O_2$) *(m/z)*: $[M+H]^+$ Calcd 385.1408; Found 385.1409; IR (ATR, ṽ) = 3135 (w), 3009 (w), 2955 (w), 2238 (m), 1711 (vs), 1611 (w), 1553 (m), 1543 (vs), 1510 (w), 1482 (w), 1452 (w), 1434 (m), 1418 (w), 1398 (w), 1356 (w), 1312 (w), 1282 (vs), 1238 (s), 1217 (w), 1184 (m), 1154 (w), 1146 (m), 1111 (vs), 1071 (w), 1035 (m), 1013 (vs), 990 (m), 953 (w), 916 (w), 909 (w), 866 (w), 844 (m), 830 (w), 799 (m), 785 (w), 747 (vs), 703 (m), 687 (vs), 653 (m), 635 (w), 628 (w), 616 (w), 572 (m), 544 (w), 517 (w), 499 (m), 487 (w), 476 (m), 446 (w), 432 (w), 405 (m) cm^{-1}.

Additional information on the chemical synthesis is available *via* Chemotion repository: https://doi.org/10.14272/reaction/SA-FUHFF-UHFFFADPSC-YZSLXIHSIJ-UHFFFADPSC-NUHFF-NUHFF-NUHFF-ZZZ

Additional information on the analysis of the target compound is available *via* Chemotion repository:
https://doi.org/10.14272/YZSLXIHSIJQQMB-UHFFFAOYSA-N.1

Methyl 1-(4-cyano-1-(4-fluorobenzyl)-1*H*-pyrazol-3-yl)-1*H*-1,2,3-triazole-4-carboxylate (21vb)

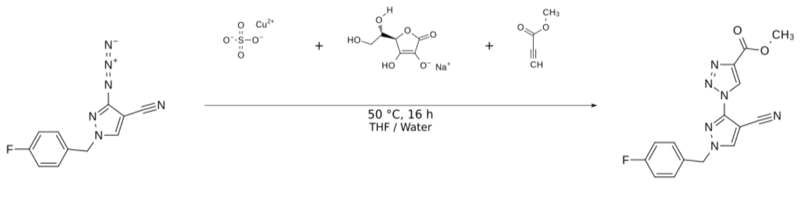

Name {P1|**21cq**}: methyl 1-(4-cyano-1-(4-fluorobenzyl)-1*H*-pyrazol-3-yl)-1*H*-1,2,3-triazole-4-carboxylate; Formula: $C_{15}H_{11}FN_6O_2$; Smiles:

COC(=O)c1nnn(c1)c1nn(cc1C#N)Cc1ccc(cc1)F. InChIKey: UUNQVGVZWMJXOZ-UHFFFAOYSA-N

In a vial, 3-azido-1-(4-fluorobenzyl)-1*H*-pyrazole-4-carbonitrile (47.6 mg, 197 µmol, 1.00 equiv) was dissolved in THF (4.00 mL) and water (4.00 mL) (1:1) and copper;sulfate (6.27 mg, 39.3 µmol, 0.20 equiv), sodium ascorbate (38.9 mg, 197 µmol, 1.00 equiv) and methyl prop-2-ynoate (21.5 mg, 22.7 µL, 255 µmol, 1.30 equiv) were added. The reaction mixture was stirred at 50 °C for 16 hours. For workup, the reaction mixture was diluted in water and extracted three times with methylene chloride. The combined organic phases were washed with water and brine, dried over Na_2SO_4, filtered and the solvent was removed under reduced pressure. As preparation for column chromatography (dry load), Celite was added (0.3 g) and the reaction mixture with Celite was evaporated. The obtained crude product was purified *via* flash-chromatography (Interchim devices puriFLASH 5.125) on silica gel (PF-15SIHP-F0012) using cyclohexane/ethyl acetate with 0% to 50% ethyl acetate in 15 column volumes. The isolated product methyl 1-(4-cyano-1-(4-fluorobenzyl)-1*H*-pyrazol-3-yl)-1*H*-1,2,3-triazole-4-carboxylate (61.4 mg, 188 µmol) was obtained as a colourless solid in 96% yield.

R_f = 0.25 (cyclohexane/ethyl acetate 1:1). MP = 186–197 °C. ^{1}H NMR (400 MHz, DMSO-d_6 [2.50 ppm], ppm) δ = 9.25 (s, 1H), 8.97 (s, 1H), 7.48–7.45 (m, 2H), 7.24–7.20 (m, 2H), 5.48 (s, 2H), 3.89 (s, 3H). ^{13}C NMR (100 MHz, DMSO-d_6 [39.5 ppm], ppm) δ = 162.0 (d, J = 244.3 Hz, C_q), 160.0 (C_q), 144.9 (C_q), 139.5 (CH), 139.3 (C_q), 131.3 (d, J = 3.1 Hz, C_q), 130.5 (d, J = 8.5 Hz, CH, 2C), 127.8 (CH), 115.6 (d, J = 21.6 Hz, CH, 2C), 111.5 (C_q), 85.1 (C_q), 55.4 (CH_2), 52.2 (CH_3); ^{19}F NMR (376 MHz, ppm) δ = -113.66; MS (EI, 70 eV, 160 °C), m/z (%): 326 (5) $[M]^+$, 267 (6), 110 (8), 109 (100), 83 (7), 69 (8). HRMS–EI ($C_{15}H_{11}O_2N_6F$) *(m/z)*: $[M]^+$ Calcd 326.0922; Found 326.0920; IR (ATR, ṽ) = 3166 (w), 3131 (w), 3108 (w), 3077 (w), 2955 (w), 2924 (w), 2856 (w), 2247 (w), 1731 (vs), 1694 (w), 1605 (w), 1548 (s), 1536 (s), 1533 (s), 1510 (vs), 1456 (w), 1442 (s), 1401 (m), 1353 (s), 1322 (w), 1262 (m), 1234 (s), 1222 (vs), 1154 (s), 1101 (m), 1033 (vs), 997 (m), 990 (m), 946 (m), 874 (w), 857 (m), 844 (s), 826 (w), 813 (m), 769 (vs), 696 (s), 640 (m), 628 (w), 579 (m), 520 (m), 504 (m), 484 (s), 453 (w), 421 (s), 378 (w) cm^{-1}.

Additional information on the chemical synthesis is available *via* Chemotion repository: https://doi.org/10.14272/reaction/SA-FUHFF-UHFFFADPSC-UUNQVGVZWM-UHFFFADPSC-NUHFF-NUHFF-NUHFF-ZZZ

Additional information on the analysis of the target compound is available *via* Chemotion repository:
https://doi.org/10.14272/UUNQVGVZWMJXOZ-UHFFFAOYSA-N.1

3-(4-(4-(Dimethylamino)phenyl)-1*H*-1,2,3-triazol-1-yl)-1-(4-fluorobenzyl)-1*H*-pyrazole-4-carbonitrile (21vg)

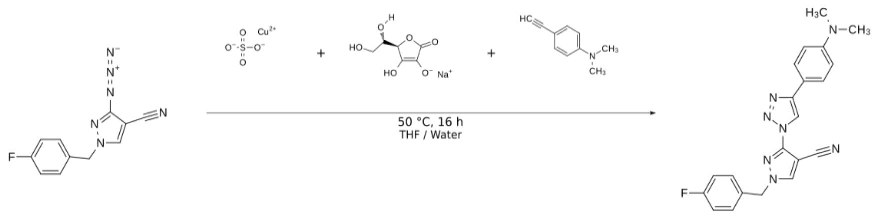

Name {P1|**21vg**}: 3-(4-(4-(dimethylamino)phenyl)-1*H*-1,2,3-triazol-1-yl)-1-(4-fluorobenzyl)-1*H*-pyrazole-4-carbonitrile; Formula: $C_{21}H_{18}FN_7$; Smiles: N#Cc1cn(nc1n1nnc(c1)c1ccc(cc1)N(C)C)Cc1ccc(cc1)F. InChIKey: UIXMTYDHDCHVFR-UHFFFAOYSA-N

In a vial, 3-azido-1-(4-fluorobenzyl)-1*H*-pyrazole-4-carbonitrile (73.0 mg, 301 μmol, 1.00 equiv) was dissolved in THF (7.00 mL) and water (7.00 mL) (1:1) and copper;sulfate (9.62 mg, 60.3 μmol, 0.20 equiv), sodium ascorbate (59.7 mg, 301 μmol, 1.00 equiv) and 4-ethynyl-N,N-dimethylaniline (56.9 mg, 392 μmol, 1.30 equiv) were added. The reaction mixture was stirred at 50 °C for 16 hours. For workup, the reaction mixture was diluted in water and extracted three times with methylene chloride. The combined organic phases were washed with water and brine, dried over Na_2SO_4, filtered and the solvent was removed under reduced pressure. As preparation for column chromatography (dry load), Celite was added (0.3 g) and the reaction mixture with Celite was evaporated. The obtained crude product was purified *via* flash-chromatography (Interchim devices puriFLASH 4125) on silica gel (PF-15SIHP-F0012) using cyclohexane/ethyl acetate with 0% to 80% ethyl acetate in 33 column volumes. The isolated product 3-(4-(4-(dimethylamino)phenyl)-1*H*-1,2,3-triazol-1-yl)-1-(4-fluorobenzyl)-1*H*-pyrazole-4-carbonitrile (88.3 mg, 228 μmol) was obtained as a light yellow solid in 76% yield.

R_f = 0.42 (cyclohexane/ethyl acetate 1:1). MP = 161.4–190.8 °C. ^{1}H NMR (400 MHz, DMSO-d_6 [2.50 ppm], ppm) δ = 8.94 (s, 1H), 8.89 (s, 1H), 7.81 (d, J = 8.9 Hz, 2H), 7.45 (dt, J = 2.1 Hz, J = 5.4 Hz, 2H), 7.25–7.20 (m, 2H), 6.77 (d, J = 8.9 Hz, 2H), 5.48 (s, 2H), 2.93 (s, 6H); ^{13}C NMR (100 MHz, DMSO-d_6 [39.5 ppm], ppm) δ = 162.0 (d, J = 244.3 Hz, C_q), 150.4 (C_q), 147.8 (C_q), 145.8 (C_q), 139.5 (CH), 131.5 (d, J = 3.1 Hz, C_q), 130.3 (d, J = 8.5 Hz, CH, 2C), 126.6 (CH, 2C), 117.4 (CH), 117.0 (C_q), 115.6 (d, J = 21.6 Hz, CH, 2C), 112.2 (CH, 2C), 112.0 (C_q), 83.9 (C_q), 55.3 (CH_2), 39.9 (CH_3, 2C); ^{19}F NMR (376 MHz, ppm) δ = -113.73; MS (FAB, 3-NBA), m/z (%): 388 (66) $[M+1]^+$, 307 (22), 289 (11), 250 (52), 154 (100), 136 (61), 120 (11), 107 (20), 91 (19). HRMS–FAB ($C_{21}H_{19}N_7F$) *(m/z)*: $[M+H]^+$ Calcd 388.1680; Found 388.1679; IR (ATR, ṽ) = 3166 (w), 3060 (w), 2847 (w), 2791 (w), 2237 (m), 1618 (s), 1577 (w), 1560 (m), 1551 (s), 1504 (vs), 1462 (w), 1442 (s), 1419 (w), 1402 (w), 1354 (s), 1298 (w), 1235 (w), 1220 (vs), 1181 (m), 1169 (w), 1156 (s), 1130 (w), 1094 (w), 1064 (w), 1043 (s), 1016 (s), 987 (w), 969 (w), 948 (m), 882 (w), 847 (m), 822 (vs), 807 (m), 792 (vs), 762 (m), 707 (w), 696 (s), 636 (m), 598 (w), 586 (w), 533 (m), 507 (s), 490 (s), 455 (w), 428 (w), 415 (w), 384 (w) cm^{-1}.

Additional information on the chemical synthesis is available *via* Chemotion repository: https://doi.org/10.14272/reaction/SA-FUHFF-UHFFFADPSC-UIXMTYDHDC-UHFFFADPSC-NUHFF-NUHFF-NUHFF-ZZZ

Additional information on the analysis of the target compound is available *via* Chemotion repository: https://doi.org/10.14272/UIXMTYDHDCHVFR-UHFFFAOYSA-N.1

Ethyl 1-(4-methoxyphenyl)-3-(4-phenyl-1*H*-1,2,3-triazol-1-yl)-1*H*-pyrazole-4-carboxylate (S4)

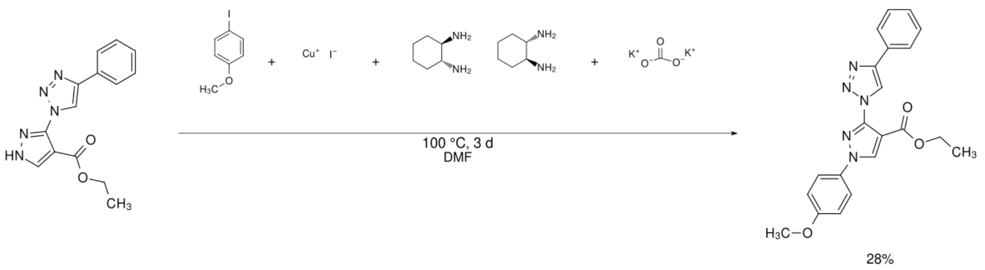

Name {P1|**S4**}: ethyl 1-(4-methoxyphenyl)-3-(4-phenyl-1*H*-1,2,3-triazol-1-yl)-1*H*-pyrazole-4-carboxylate; Formula: C21H19N5O3; Smiles: CCOC(=O)c1cn(nc1n1nnc(c1)c1ccccc1)c1ccc(cc1)OC. InChIKey: UEMJXDBBUGZBJY-UHFFFAOYSA-N

1-Iodo-4-methoxybenzene (82.6 mg, 353 µmol, 2.00 equiv) was added under argon to a stirring reaction mixture of ethyl 3-(4-phenyl-1*H*-1,2,3-triazol-1-yl)-1*H*-pyrazole-4-carboxylate (50.0 mg, 177 µmol, 1.00 equiv), copper(I) iodide (16.8 mg, 88.3 µmol, 0.500 equiv), cyclohexane-1,2-diamine (40.3 mg, 42.4 µL, 177 µmol, 1.00 equiv) and dipotassium carbonate (73.2 mg, 530 µmol, 3.00 equiv) in dry N,N-dimethylformamide (2.0 mL). The reaction was stirred for 3 days at 100 °C. The reaction mixture was cooled down to 21 °C, diluted with water and extracted with ethyl acetate. The combined organic phase was washed with aq. sodium thiosulfate solution and brine, dried over Na_2SO_4 and filtered. As preparation for the column chromatography (dryload), Celite was added (0.2 g) and the reaction mixture with Celite was evaporated. The obtained crude product was purified via flash-chromatography (Interchim devices puriFLASH 5.125) on silica gel (PF-15SIHP-F0012) using cyclohexane/ethyl acetate 0% to 100% ethyl acetate in 15 column volumes (1 column volume = 39.3 mL; flow: 15 mL/min). The isolated product ethyl 1-(4-methoxyphenyl)-3-(4-phenyl-1*H*-1,2,3-triazol-1-yl)-1*H*-pyrazole-4-carboxylate (19.1 mg, 49.0 µmol) was obtained as a colorless solid in 28% yield.R_f = 0.62 (cyclohexane/ethyl acetate 4:1). ^{1}H NMR (400 MHz, Chloroform-d [7.26 ppm], ppm) δ = 8.46 (s, 1H), 8.43 (s, 1H), 7.93 (d, *J* = 7.4 Hz, 2H), 7.68–7.65 (m, 2H), 7.46 (t, *J* = 7.4 Hz, 2H), 7.38–7.34 (m, 1H), 7.02–7.00 (m, 2H), 4.28 (q, *J* = 7.1 Hz, 2H), 3.86 (s, 3H), 1.27 (t, *J* = 7.1 Hz, 3H); ^{13}C NMR (100 MHz, Chloroform-d [77.16 ppm], ppm) δ = 161.1 (C_q), 159.8 (C_q), 147.3 (C_q), 145.5 (C_q), 132.3 (C_q), 132.2 (CH), 130.4 (C_q), 129.0 (CH, 2C), 128.4 (CH), 126.1 (CH, 2C), 122.1 (CH), 121.6 (CH, 2C), 114.9 (CH, 2C), 109.6 (C_q), 61.2 (CH_2), 55.8 (CH_3), 14.3 (CH_3); MS (FAB, 3-NBA), m/z (%): 390 (100) $[M+H]^+$, 361 (23), 307 (10), 154 (44), 136 (33), 107 (11). HRMS–FAB ($C_{21}H_{20}O_3N_5$) *(m/z)*: $[M+H]^+$ Calcd 390.1561; Found 390.1561; IR (ATR, ṽ) = 3131 (w), 3099 (w), 3084 (w), 3003 (w), 2982 (w), 2962 (w), 2938 (w), 2927 (w), 2833 (w), 1713 (s), 1697 (vs), 1609 (w), 1555 (vs), 1534 (s), 1519 (vs), 1479 (m), 1463 (w), 1449 (m), 1405 (m), 1371 (w), 1307 (w), 1275 (vs), 1264 (vs), 1247 (vs), 1221 (s), 1180 (s), 1145 (vs), 1112 (m), 1086 (m), 1065 (m), 1031 (vs), 1017 (vs), 992 (m), 965 (m), 956 (m), 916 (w), 868 (w), 857 (w), 837 (vs), 807 (w), 799 (w), 762 (vs), 691 (vs), 670 (m), 643 (m), 633 (s), 615 (m), 541 (m), 524 (m), 510 (m), 489 (w), 463 (w), 445 (w), 414 (w), 375 (w) cm^{-1}.

Additional information on the chemical synthesis is available via Chemotion repository: https://doi.org/10.14272/reaction/SA-FUHFF-UHFFFADPSC-UEMJXDBBUG-UHFFFADPSC-NUHFF-NUHFF-NUHFF-ZZZ

Additional information on the analysis of the target compound is available via Chemotion repository: https://doi.org/10.14272/UEMJXDBBUGZBJY-UHFFFAOYSA-N.1

One-Pot procedure to 4-(5-methyl-3-(4-phenyl-1*H*-1,2,3-triazol-1-yl)-1*H*-pyrazol-1-yl)benzonitrile (21gd):

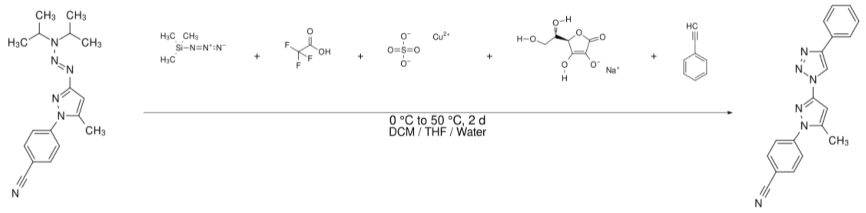

Name {P1|**21gd**}: 4-(5-methyl-3-(4-phenyl-1*H*-1,2,3-triazol-1-yl)-1*H*-pyrazol-1-yl)benzonitrile; Formula: $C_{19}H_{14}N_6$; Smiles: N#Cc1ccc(cc1)n1nc(cc1C)n1nnc(c1)c1ccccc1. InChIKey: QJHTUPAVPUUSNY-UHFFFAOYSA-N

(E)-4-(3-(3,3-diisopropyltriaz-1-en-1-yl)-5-methyl-1*H*-pyrazol-1-yl)benzonitrile (100 mg, 322 µmol, 1.00 equiv) was dissolved in methylene chloride (6.00 mL). The solution was cooled to 0 °C and then azido(trimethyl)silane (278 mg, 321 µL, 2.42 mmol, 7.50 equiv) and 2,2,2-trifluoroacetic acid (367 mg, 247 µL, 3.22 mmol, 10.0 equiv) were added. The reaction mixture was slowly warmed to 50 °C while constantly being stirred. The reaction progress was monitored *via* TLC. After full conversion (16 h), the solvent was reduced under vacuum. The azide was dissolved in THF (6.00 mL) and water (6.00 mL) (1:1) and copper sulfate (10.3 mg, 64.4 µmol, 0.200 equiv), sodium ascorbate (63.8 mg, 322 µmol, 1.00 equiv) and ethynylbenzene (42.8 mg, 46.0 µL, 419 µmol, 1.30 equiv) were added. The reaction mixture was stirred at 50 °C for 16 hours. For work up the reaction mixture was diluted in water and extracted three times with methylene chloride. The combined organic phase was washed with water and brine, dried over Na_2SO_4 and filtered. As preparation for the column chromatography (dryload), Celite was added (0.3 g) and the reaction mixture with Celite were evaporated. The obtained crude product was purified *via* flash-chromatography (Interchim devices puriFLASH 5.125) on silica gel (PF-15SIHP-F0025) using cyclohexane/ethyl acetate 0% to 20% ethyl acetate in 12 column volumes. The isolated product 4-(5-methyl-3-(4-phenyl-1*H*-1,2,3-triazol-1-yl)-1*H*-pyrazol-1-yl)benzonitrile (105 mg, 322 µmol) was obtained as a colorless solid in 100% yield.

R_f = 0.16 (cyclohexane/ethyl acetate 4:1). ^{1}H NMR (400 MHz, Chloroform-d [7.26 ppm], ppm) δ = 8.45 (s, 1H), 7.92–7.90 (m, 2H), 7.83–7.81 (m, 2H), 7.70–7.68 (m, 2H), 7.45 (t, J = 7.5 Hz, 2H), 7.38–7.35 (m, 1H), 6.83–6.83 (m, 1H), 2.51 (s, 3H).; ^{13}C NMR (100 MHz, Chloroform-d [77.2 ppm], ppm) δ = 148.0 (C_q), 147.8 (C_q), 142.6 (C_q), 142.0 (C_q), 133.5 (2C, CH), 130.2 (C_q), 129.0 (2C, CH), 128.6 (CH), 126.0 (2C, CH), 124.8 (2C, CH), 118.1 (C_q), 116.9 (CH), 111.7 (C_q), 100.2 (CH), 13.4 (CH_3). For further analytical

data, please refer to the synthesis of the triazole product **21gd** from the respective azide (page 78-79 of this Supporting Information).

Additional information on the chemical synthesis is available *via* Chemotion repository: https://dx.doi.org/10.14272/reaction/SA-FUHFF-UHFFFADPSC-QJHTUPAVPU-UHFFFADPSC-NUHFF-NUHFF-NUHFF-ZZZ.1

Additional information on the analysis of the target compound is available *via* Chemotion repository: https://dx.doi.org/10.14272/QJHTUPAVPUUSNY-UHFFFAOYSA-N.2

Solid-Phase Reactions:

3-(1*H*-5-Methylpyrazole) triazene resin (23)

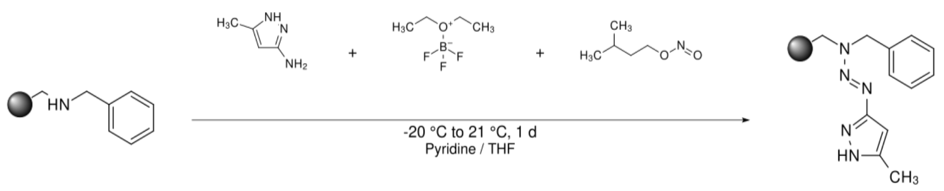

Name {P1|**23**}: 3-(1*H*-5-Methylpyrazole) triazene resin; Formula: $C_{12}H_{14}N_5$; Smiles: CCN(Cc1ccccc1)/N=N/c1n[nH]c(c1)C. InChIKey: AFYWJZVKEAQQHO-WUKNDPDISA-N.

Variation A: The benzylamine resin (4.27 g, 4.27 L, 3.98 mmol, 1.00 equiv) was swollen in a 9:1 mixture of THF/pyridine (85 mL) and then cooled to ca. -10 °C. In a separate flask, 5-methyl-1*H*-pyrazol-3-amine (1.93 g, 19.9 mmol, 5.00 equiv) was dissolved in dry THF and cooled to -20 °C. The reagent boron trifluoride diethyl etherate (4.23 g, 3.68 mL, 29.8 mmol, 7.50 equiv) was added and the solution was stirred for 5 minutes. Then isoamyl nitrite (3.49 g, 4.01 mL, 29.8 mmol, 7.50 equiv) was added and the mixture was stirred for two hours. The diazonium salt was then filtered off and washed with cold Et_2O. The salt was then dissolved in the minimal amount of MeCN with addition of a few drops of DMF and added slowly to the previously swollen and cooled resin. After addition of the diazonium salt, the resin was slowly warmed to 21 °C and was shaken over night. The supernatant was filtered off and the resin was washed as follows: 3x acetone, 3x distilled water, 3x acetone, 3x THF, 3x MeOH, 3x methylene chloride, 3x MeOH. The resin was then dried in vacuo to yield the orange triazene resin (4.74 g, 0.839 mmol/g, 3.98 mmol, 100% yield, quantitative yield according to mass difference).

Variation B: To a -20 °C cold solution of 5-methyl-1*H*-pyrazol-3-amine (768 mg, 7.91 mmol, 5.00 equiv) in dry THF (9.00 mL) was added boron trifluoride etherate (1.68 g, 1.50 mL, 11.9 mmol, 7.50 equiv) under nitrogen. After 5 minutes, 3-methylbutyl nitrite (1.39 g, 1.59 mL, 11.9 mmol, 7.50 equiv) was added dropwise at -20 °C. The mixture was stirred for 2 hours at -20 °C and the precipitate was filtered (by glass tube with frit) and washed with cold ether. In a round bottom flask, benzylamine resin (2.00 g, 1.58 mmol, 1.00 equiv) was swollen in a mixture of THF (36.0 mL) and pyridine (4.00 mL) (9:1). The diazonium salt was then taken up in a little amount of acetonitrile and added slowly to the previously swollen benzylamine resin, which was cooled to -

20 °C. The mixture was kept at -20 °C for 2 hours and manually swirled at regular intervals. The flask was then shaken at 21 °C for further 14 hours. The resin was washed with 3x acetone, 3x dist. water, 3x acetone, 3x THF, 3x MeOH, 3x methylene chloride, 3x MeOH. The resin was dried at high vacuum until constant weight was obtained. 3-(1*H*-5-Methylpyrazole) triazene resin (2.28 g, 0.695 mmol/g, 1.58 mmol) was obtained as an orange-colored resin in 100% yield (quantitative yield according to mass difference).

IR (ATR, ṽ) = 3143, 3101, 3080, 3058, 3024, 3003, 2973, 2919, 2850, 2293, 2196, 2085, 1945, 1873, 1800, 1720, 1601, 1584, 1541, 1511, 1492, 1449, 1422, 1371, 1346, 1316, 1269, 1176, 1154, 1137, 1112, 1065, 1027, 980, 946, 904, 878, 840, 789, 751, 696, 564, 538, 479, 428, 385 cm^{-1}.

Additional information on the chemical synthesis is available *via* Chemotion repository:
https://dx.doi.org/10.14272/reaction/SA-FUHFF-UHFFFADPSC-AFYWJZVKEA-UHFFFADPSC-NUHFF-NAKRX-NUHFF-ZZZ
https://dx.doi.org/10.14272/reaction/SA-FUHFF-UHFFFADPSC-AFYWJZVKEA-UHFFFADPSC-NUHFF-NAKRX-NUHFF-ZZZ.1

Additional information on the analysis of the target compound is available *via* Chemotion repository:
https://dx.doi.org/10.14272/AFYWJZVKEAQQHO-WUKNDPDISA-N.1
https://dx.doi.org/10.14272/AFYWJZVKEAQQHO-WUKNDPDISA-N.2

(Phenyl-4-carbonitrile)-1*H*-5-methylpyrazole-3-triazene resin (25)

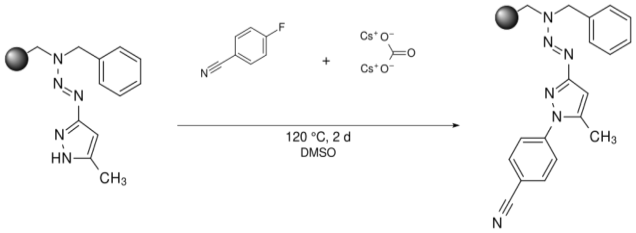

Name {P1|**25**}: (Phenyl-4-carbonitrile)-1*H*-5-methylpyrazole-3-triazene resin; Formula: $C_{19}H_{17}N_6$; Smiles: CCN(Cc1ccccc1)/N=N/c1nn(c(c1)C)c1ccc(cc1)C#N. InChIKey: HEQYPJIVZWENPL-ZNTNEXAZSA-N

Variation A: In a vial, the pyrtriazene resin obtained *via* variation A (302 mg, 302 mL, 254 µmol, 1.00 equiv) was swollen in 6 mL of DMSO. Then, caesium carbonate (413 mg, 1.27 mmol, 5.00 equiv) and 4-fluorobenzonitrile (123 mg, 1.01 mmol, 4.00 equiv) were added. The vial was closed and shaken at 120 °C for 2 days. The supernatant was filtered off and the resin was washed as follows: 3x acetone, 3x water, 3x acetone, 3x THF, 3x MeOH, 3x methylene chloride, 3x MeOH. It was then dried in vacuo to yield 318 mg of an orange resin (254 µmol, 0.500 mmol/g and 63% yield for loading from mass difference, 0.797 mmol/g for quantitative conversion and 100% yield).

Variation B: In a vial, 3-(1*H*-5-methylpyrazole) triazene resin obtained *via* variation B (1.00 g, 695 µmol, 1.00 equiv) was suspended in DMSO (20.0 mL). Cesium carbonate (1.13 g, 3.48 mmol, 5.00 equiv) and 4-fluorobenzonitrile (337 mg, 2.78 mmol, 4.00

equiv) were added. The vial was closed and shaken at 120 °C for 3 days. The resin was washed with 3x acetone, 3x dist. water, 3x acetone, 3x THF, 3x MeOH, 3x methylene chloride, 3x MeOH. The resin was then dried at high vacuum until constant weight was obtained. (Phenyl-4-carbonitrile)-1H-5-methylpyrazole-3-triazene resin (1.05 g, 531 µmol) was obtained as an orange-colored resin in 76% yield (0.504 mmol/g, loading from mass difference; 0.660 mmol/g for quantitative conversion and 100% yield).

IR (ATR, ṽ) = 3081 (vw), 3058 (vw), 3026 (w), 2918 (w), 2850 (vw), 2228 (vw), 1602 (w), 1510 (w), 1492 (w), 1449 (m), 1419 (w), 1373 (w), 1346 (w), 1177 (w), 1152 (w), 1108 (w), 1064 (w), 1027 (w), 1017 (w), 977 (w), 905 (w), 840 (w), 749 (m), 696 (vs), 622 (m), 538 (s), 392 (m) cm^{-1}.

Additional information on the chemical synthesis is available *via* Chemotion repository:
https://dx.doi.org/10.14272/reaction/SA-FUHFF-UHFFFADPSC-HEQYPJIVZW-UHFFFADPSC-NUHFF-NVVLV-NUHFF-ZZZ
https://dx.doi.org/10.14272/reaction/SA-FUHFF-UHFFFADPSC-HEQYPJIVZW-UHFFFADPSC-NUHFF-NVVLV-NUHFF-ZZZ.1

Additional information on the analysis of the target compound is available *via* Chemotion repository:
https://dx.doi.org/10.14272/HEQYPJIVZWENPL-ZNTNEXAZSA-N.1
https://dx.doi.org/10.14272/HEQYPJIVZWENPL-ZNTNEXAZSA-N.2

Cleavage to 4-(3-Azido-5-methyl-1H-pyrazol-1-yl)benzonitrile (19g)

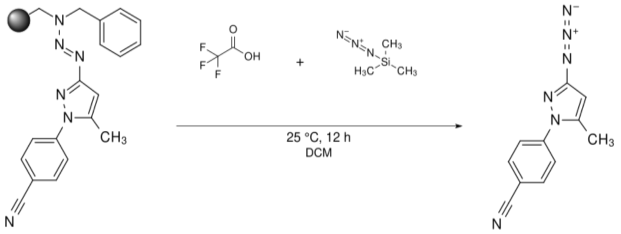

Name {P1|**19g**}: 4-(3-azido-5-methyl-1H-pyrazol-1-yl)benzonitrile; Formula: $C_{11}H_8N_6$; Smiles: [N-]=[N+]=Nc1nn(c(c1)C)c1ccc(cc1)C#N. InChIKey: KIQBPEXSAZKVRR-UHFFFAOYSA-N.

The pyr-triazene-resin obtained from variation A (213 mg, 213 mL, 170 µmol, 1.00 equiv) was swollen in 4 mL of dichloromethane. Then 2,2,2-trifluoroacetic acid (194 mg, 130 µL, 1.70 mmol, 10.0 equiv) and azido(trimethyl)silane (206 mg, 237 µL, 1.70 mmol, 10.0 equiv) were added and the vial was closed. The mixture was shaken for 12 hours at 25 °C. The resin was filtered off and washed with methylene chloride. The combined filtrates were washed with a saturated K_2CO_3-solution and the solvent was removed under reduced pressure. The crude product was put on Celite® and purified *via* flash chromatography (cyclohexane/ethyl acetate 10:1 -> 4:1) to yield 4-(3-azido-5-methyl-1*H*-pyrazol-1-yl)benzonitrile (13.9 mg, 62.0 µmol, 58% yield) as a brown solid.

^{1}H NMR (400 MHz, DMSO-*d*$_6$ [2.50 ppm], ppm) δ = 8.04–7.95 (m, 2H), 7.80–7.73 (m, 2H), 6.20 (d, *J* = 1.0 Hz, 1H), 2.40 (s, 3H); ^{13}C NMR (100 MHz, DMSO-*d*$_6$ [39.5 ppm], ppm) δ = 148.3, 142.6, 142.3, 133.5 (CH, 2C), 124.0 (CH, 2C), 118.3, 109.6, 99.8 (+,

CH), 12.7 (CH_3); EI (m/z, 70 eV, 60 °C): 224 (18) $[M]^+$, 130 (39), 103 (10), 102 (100), 75 (12), 66 (10). HRMS ($C_{11}H_8N_6$): calcd 224.0810, found 224.0809; IR (ATR, ṽ) = 3352, 3133, 3104, 2995, 2921, 2851, 2472, 2387, 2357, 2259, 2230, 2118, 2050, 1919, 1792, 1677, 1605, 1579, 1545, 1511, 1475, 1449, 1434, 1378, 1315, 1298, 1271, 1238, 1207, 1180, 1147, 1120, 1111, 1081, 1057, 1030, 1016, 976, 956, 843, 812, 783, 724, 694, 677, 654, 622, 601, 575, 560, 547, 528, 517, 500, 473, 456, 404, 384 cm^{-1}.

Additional information on the chemical synthesis is available *via* Chemotion repository: https://dx.doi.org/10.14272/reaction/SA-FUHFF-UHFFFADPSC-KIQBPEXSAZ-UHFFFADPSC-NUHFF-NUHFF-NUHFF-ZZZ.1

Additional information on the analysis of the target compound is available *via* Chemotion repository:
https://dx.doi.org/10.14272/KIQBPEXSAZKVRR-UHFFFAOYSA-N.2

Cleavage to 4-(5-Methyl-3-(4-phenyl-1*H*-1,2,3-triazol-1-yl)-1*H*-pyrazol-1-yl)benzonitrile (21gd)

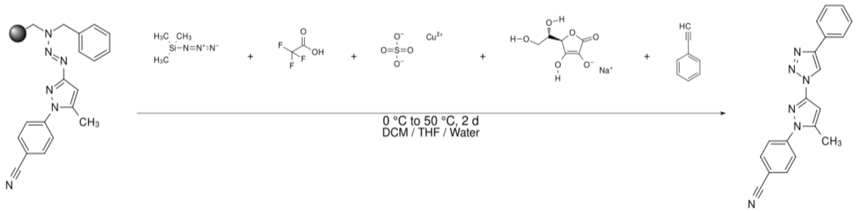

Name {P1|**21gd**}: 4-(5-methyl-3-(4-phenyl-1*H*-1,2,3-triazol-1-yl)-1*H*-pyrazol-1-yl)benzonitrile; Formula: $C_{19}H_{14}N_6$; Smiles: N#Cc1ccc(cc1)n1nc(cc1C)n1nnc(c1)c1ccccc1. InChIKey: QJHTUPAVPUUSNY-UHFFFAOYSA-N

(Phenyl-4-carbonitrile)-1*H*-5-methylpyrazole-3-triazene resin (250 mg, 126 µmol, 1.00 equiv) was suspended in methylene chloride (3.00 mL). The suspension was cooled to 0 °C and azido(trimethyl)silane (145 mg, 167 µL, 1.26 mmol, 10.0 equiv) and 2,2,2-trifluoroacetic acid (144 mg, 96.5 µL, 1.26 mmol, 10.0 equiv) were added. The reaction mixture was slowly warmed to 50 °C and shaken for 16 hours. For the click-step THF (3.00 mL) and water (3.00 mL) (1:1) was added as solvent and copper sulfate (6.03 mg, 37.8 µmol, 0.300 equiv), sodium ascorbate (25.0 mg, 126 µmol, 1.00 equiv) and ethynylbenzene (64.3 mg, 69.2 µL, 630 µmol, 5.00 equiv) were added. The reaction mixture was shaken at 50 °C for 16 hours. For work up, the reaction mixture was diluted in water and extracted three times with methylene chloride. The combined organic phase was washed with water and brine, dried over Na_2SO_4 and filtered. As preparation for the column chromatography (dryload), Celite was added (0.3 g) and the reaction mixture with Celite were evaporated. The obtained crude product was purified *via* flash-chromatography (Interchim devices puriFLASH 5.125) on silica gel (PF-15SIHP-F0012) using cyclohexane/ethyl acetate 0% to 20% ethyl acetate in 15 column volumes. The isolated product 4-(5-methyl-3-(4-phenyl-1*H*-1,2,3-triazol-1-yl)-1*H*-pyrazol-1-yl)benzonitrile (16.3 mg, 49.9 µmol) was obtained as a colorless solid in 40% yield.

R_f = 0.33 (cyclohexane/ethyl acetate 2:1). MP = 163.1–174.1 °C. ^{1}H NMR (400 MHz, DMSO-d_6 [2.50 ppm], ppm) δ = 9.19 (s, 1H), 8.08 (d, J = 8.6 Hz, 2H), 8.01–7.99 (m,

2H), 7.91 (d, J = 8.6 Hz, 2H), 7.48 (t, J = 7.5 Hz, 2H), 7.40–7.36 (m, 1H), 6.92 (s, 1H), 2.51 (s, 3H). ^{13}C NMR (100 MHz, DMSO-d_6 [39.5 ppm], ppm) δ = 147.0 (C_q), 146.7 (C_q), 142.9 (C_q), 142.1 (C_q), 133.7 (2C, CH), 129.9 (C_q), 128.9 (2C, CH), 128.3 (CH), 125.5 (2C, CH), 124.9 (2C, CH), 118.9 (C_q), 118.2 (CH), 110.5 (C_q), 99.8 (CH), 12.6 (CH_3). For further analytical data, please refer to the synthesis of the triazole product **21gd** from the respective azide (page 78-79 of this Supporting Information).

Additional information on the chemical synthesis is available *via* Chemotion repository:
https://dx.doi.org/10.14272/reaction/SA-FUHFF-UHFFFADPSC-QJHTUPAVPU-UHFFFADPSC-NUHFF-NUHFF-NUHFF-ZZZ.2

Additional information on the analysis of the target compound is available *via* Chemotion repository:
https://dx.doi.org/10.14272/QJHTUPAVPUUSNY-UHFFFAOYSA-N.3

3. Crystallographic Results

Table S1: Crystal data and structure refinement details for **18n**, **21sd** and **21vg**.

Identification code	SG-V3461-21-30 (**18n**)	SG-V3478-7-10 (**21vg**)	NiW-C1184.1; X12101 (sb1181_hy, **21sd**)
Empirical formula	$C_{17}H_{22}N_6$	$C_{21}H_{18}FN_7$	$C19H_{12}F_2N_6$
Formula weight	310.40	387.42	362.35
Temperature/K	150	150	123
Crystal system	triclinic	orthorhombic	triclinic
Space group	$P\overline{1}$	$P2_12_12_1$	$P\overline{1}$
a/Å	9.8910(5)	5.9668(4)	8.9104(3
b/Å	10.2935(5)	15.3420(8)	12.7983(4
c/Å	10.3569(5)	20.8845(10)	15.4890(5)
α/°	115.249(3)	90	77.740(1)
β/°	101.092(4)	90	78.481(1)
γ/°	106.000(4)	90	82.976(1)
Volume/Å^3	856.54(8)	1911.82(19)	1585.39(10)
Z	2	4	4
ρ_{calc}g/cm^3	1.204	1.346	1.428
μ/mm^{-1}	0.387	0.484	0.890
F(000)	332	808	744
Crystal size/mm^3	0.16 × 0.14 × 0.03	0.16 × 0.12 × 0.03	0.024 x 0.012 x 0.06
Radiation	GaKα (λ = 1.34143)	GaKα (λ = 1.34143)	CuKα (λ = 1.54178)
2Θ range for data collection/°	8.66 to 124.934	6.22 to 124.946	5.934 to 144.362
Index ranges	-12 ≤ h ≤ 13, -13 ≤ k ≤ 13, -11 ≤ l ≤ 13	-6 ≤ h ≤ 7, -7 ≤ k ≤ 20, -26 ≤ l ≤ 27	-1 ≤ h ≤ 10, -15 ≤ k ≤ 15, -19 ≤ l ≤ 19
Reflections collected	10354	11784	30147
Independent reflections	4005 [R_{int} = 0.0141]	4484 [R_{int} = 0.0216]	6589 [R_{int} = 0.027]
Indp. refl. with I≥=2σ (I)	3475	3818	6179
Data/restraints/parameters	4005/0/212	4484/0/264	6589/0/488
Goodness-of-fit on F^2	1.081	0.962	1.025
Final R indexes [I≥=2σ (I)]	R_1 = 0.0355, wR_2 = 0.0987	R_1 = 0.0308, wR_2 = 0.0690	R_1 = 0.0341, wR_2 = 0.0888
Final R indexes [all data]	R_1 = 0.0400, wR_2 = 0.1011	R_1 = 0.0387, wR_2 = 0.0715	R_1 = 0.0360, wR_2 = 0.0906
Largest diff. peak/hole / e Å^{-3}	0.21/–0.23	0.12/–0.20	23/-30
Flack parameter	-	0.37(12)	-
CCDC Number	2308695	2308696	2309318

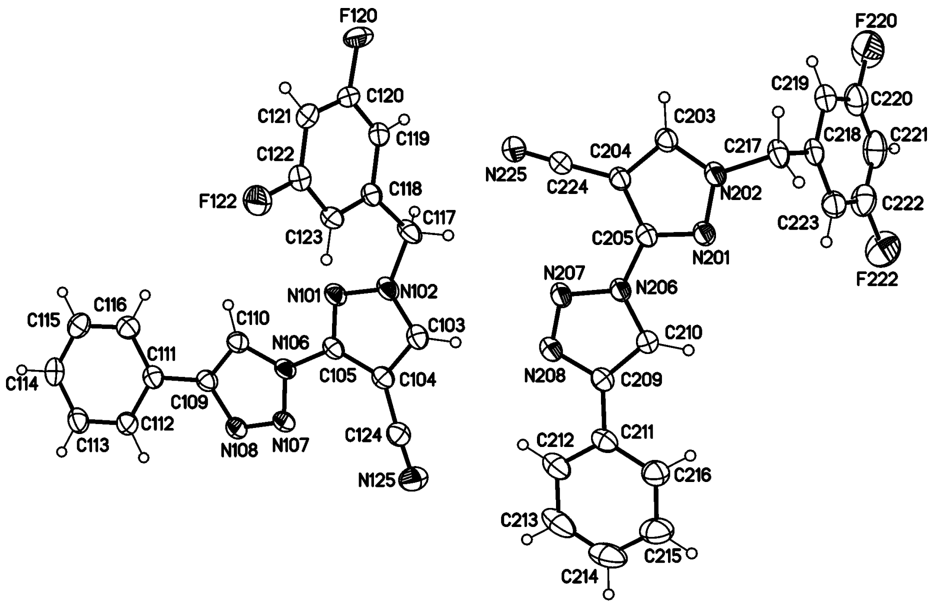

Figure S2: Molecular structure of **21sd** (NiW-C1184.1; X12101 (sb1181_hy) (displacement parameters are drawn at 50 % probability level).

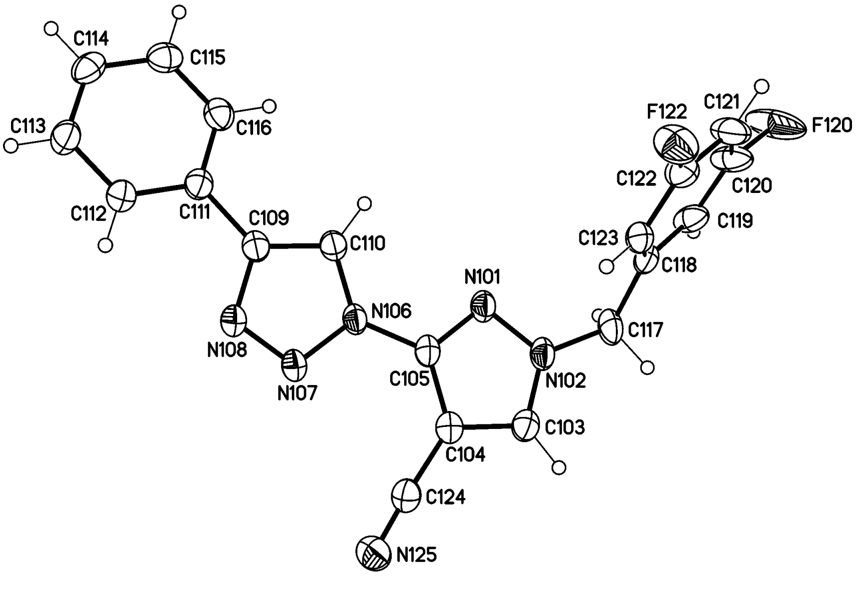

Figure S3: 1st crystallographic independent molecule of **21sd** (NiW-C1184.1; X12101 (sb1181_hy) (displacement parameters are drawn at 50 % probability level).

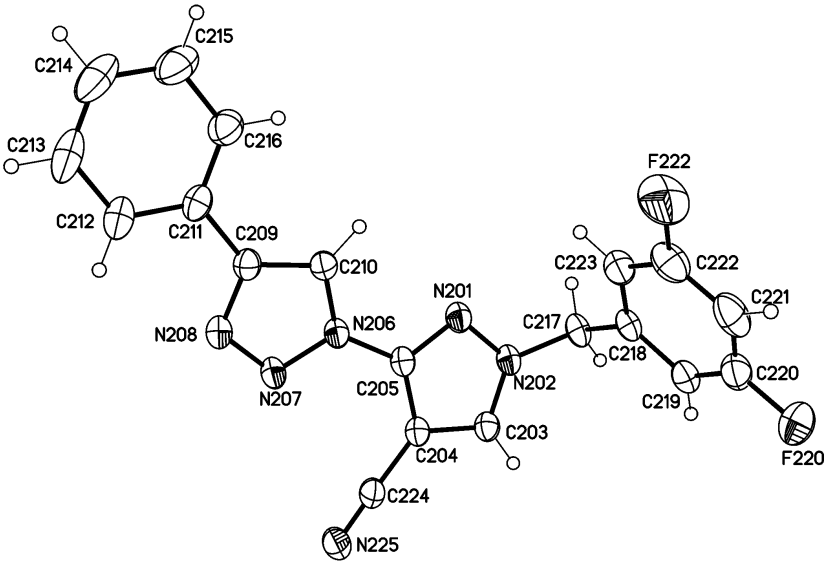

Figure S4: 2nd crystallographic independent molecule of **21sd** (NiW-C1184.1; X12101 (sb1181_hy) (displacement parameters are drawn at 50 % probability level).

4. References

1. Dolomanov, O. V.; Bourhis, L. J.; Gildea, R. J.; Howard, J. A. K.; Puschmann, H. *J Appl Crystallogr* **2009,** *42* (2), 339–341. doi:10.1107/S0021889808042726
2. Sheldrick, G. M. *Acta Crystallogr. A* **2015,** *71* (Pt 1), 3–8. doi:10.1107/S2053273314026370
3. Sheldrick, G. M. *Acta Crystallogr. C* **2015,** *71* (Pt 1), 3–8. doi:10.1107/S2053229614024218
4. Wippert, N. A.; Jung, N.; Bräse, S. *ACS Comb. Sci.* **2019,** *21* (8), 568–572. doi:10.1021/acscombsci.9b00096
5. Wippert, N.; Nieger, M.; Herlan, C.; Jung, N.; Bräse, S. *Beilstein J. Org. Chem* **2021,** *17*, 2773–2780. doi:10.3762/bjoc.17.187
6. Chen, X.; Jin, Q.; Wu, L.; Tung, C.; Tang, X. *Angew. Chem., Int. Ed.* **2014,** *53* (46), 12542–12547. doi:10.1002/anie.201408422
7. Alcalde, E.; Mendoza, J. de; Elguero, J. *J. Heterocyclic Chem.* **1974,** *11* (6), 921–928. doi:10.1002/jhet.5570110613
8. Cleemann, F.; Kum-Cheung, W. L.; Karuso, P. *Tetrahedron Lett.* **2022,** *88*, 153520. doi:10.1016/j.tetlet.2021.153520
9. Japelj, B.; Rečnik, S.; Čebašek, P.; Stanovnik, B.; Svete, J. *J. Heterocyclic Chem.* **2005,** *42* (6), 1167–1173. doi:10.1002/jhet.5570420620
