# Supplementary material for: Synthesis of substituted triazole–pyrazole hybrids using triazenylpyrazole precursors
Source: Beilstein J Org Chem. 2024 Jun 20;20:1396–404. doi: 10.3762/bjoc.20.121 (PMC11196952; doi:10.3762/bjoc.20.121)

BEILSTEIN JOURNAL OF ORGANIC CHEMISTRY

Supporting Information

for

Synthesis of substituted triazole–pyrazole hybrids using triazenylpyrazole precursors

Simone Gräßle, Laura Holzhauer, Nicolai Wippert, Olaf Fuhr, Martin Nieger, Nicole Jung and Stefan Bräse

Beilstein J. Org. Chem. **2024,** *20,* 1396–1404. doi:10.3762/bjoc.20.121

NMR spectra

License and Terms: This is a supporting information file under the terms of the Creative Commons Attribution License (https://creativecommons.org/licenses/by/4.0). Please note that the reuse, redistribution and reproduction in particular requires that the author(s) and source are credited and that individual graphics may be subject to special legal provisions.
The license is subject to the *Beilstein Journal of Organic Chemistry* terms and conditions: (https://www.beilstein-journals.org/bjoc/terms)

[**15a**] (*E*)-3-(3,3-diisopropyltriaz-1-en-1-yl)-1*H*-pyrazole

CHMO:0000593 | ^{1}H nuclear magnetic resonance spectroscopy (^{1}H NMR)

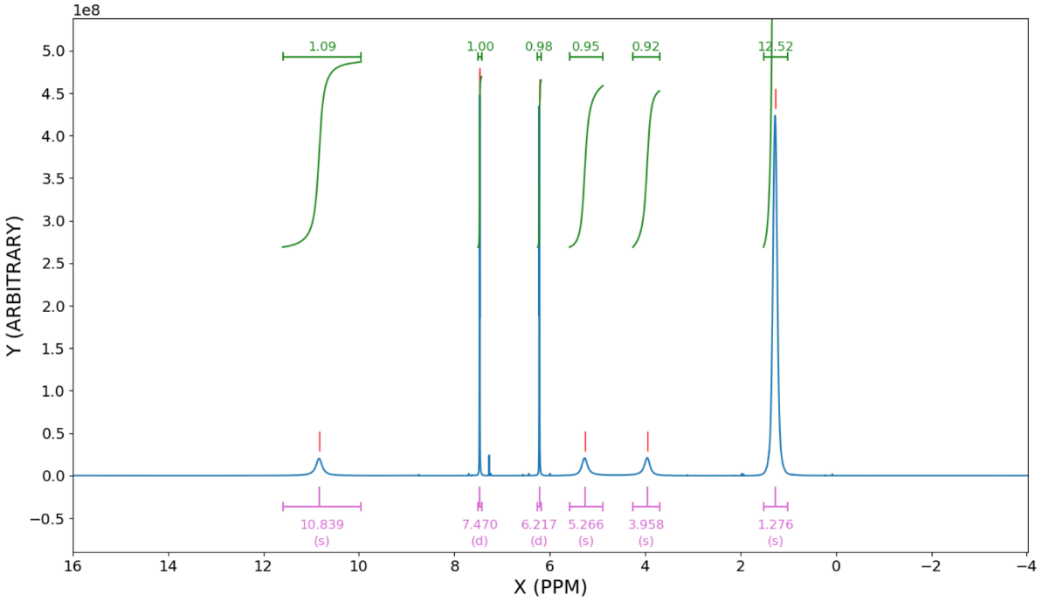

CHMO:0000595 | ^{13}C nuclear magnetic resonance spectroscopy (^{13}C NMR)

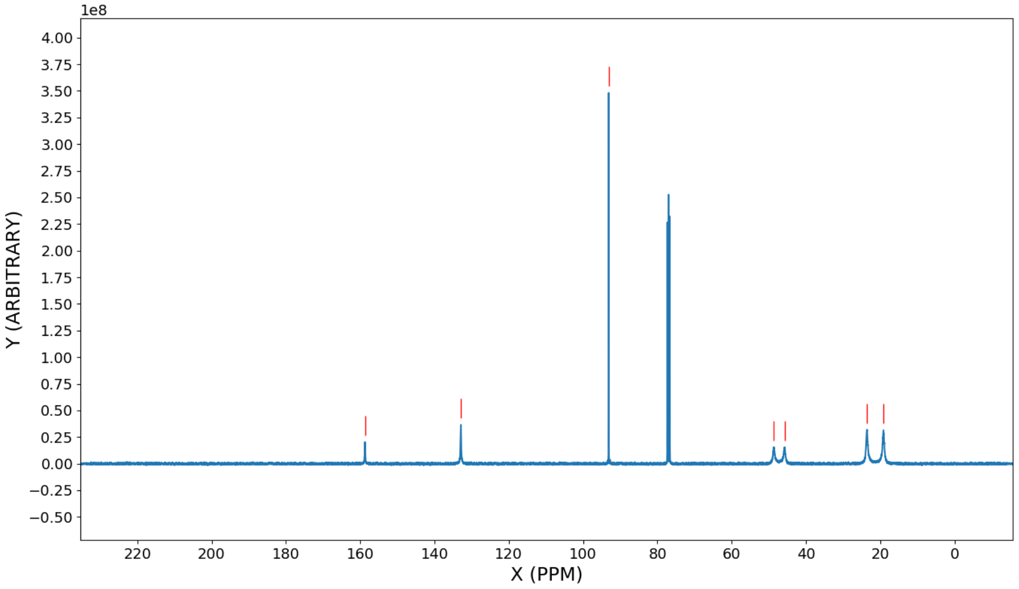

[**15b**] (*E*)-3-(3,3-diisopropyltriaz-1-en-1-yl)-5-methyl-1*H*-pyrazole

CHMO:0000593 | ^{1}H nuclear magnetic resonance spectroscopy (^{1}H NMR)

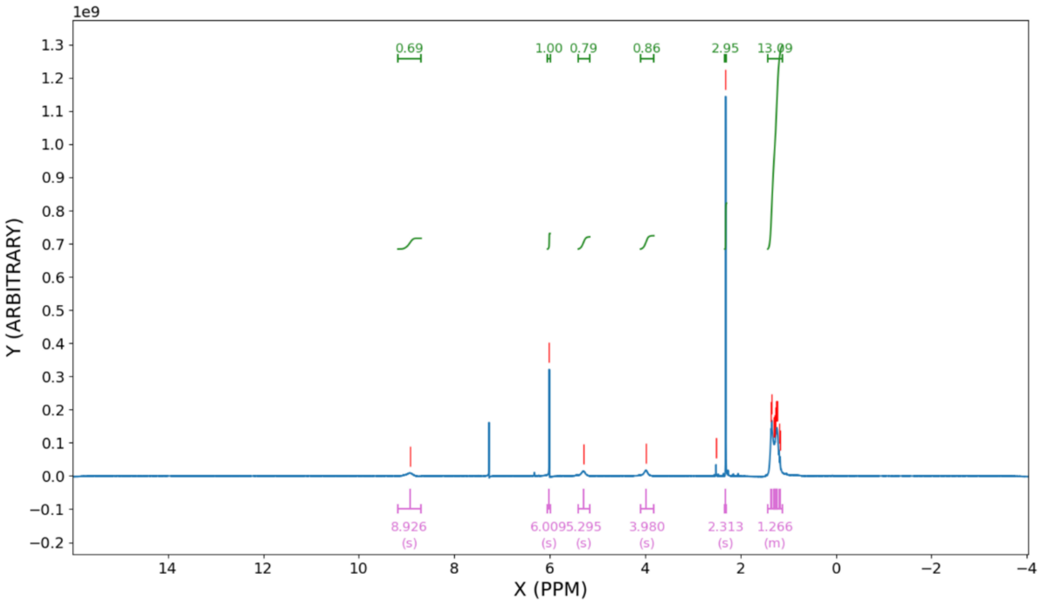

CHMO:0000595 | ^{13}C nuclear magnetic resonance spectroscopy (^{13}C NMR)

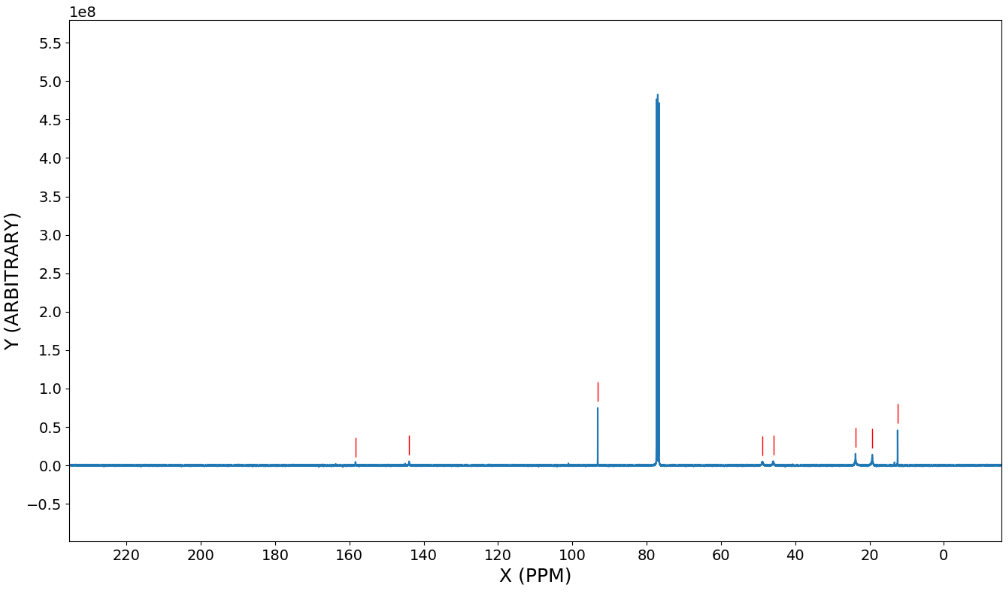

[**15c**] Ethyl (*E*)-3-(3,3-diisopropyltriaz-1-en-1-yl)-1*H*-pyrazole-4-carboxylate

CHMO:0000593 | ^{1}H nuclear magnetic resonance spectroscopy (^{1}H NMR)

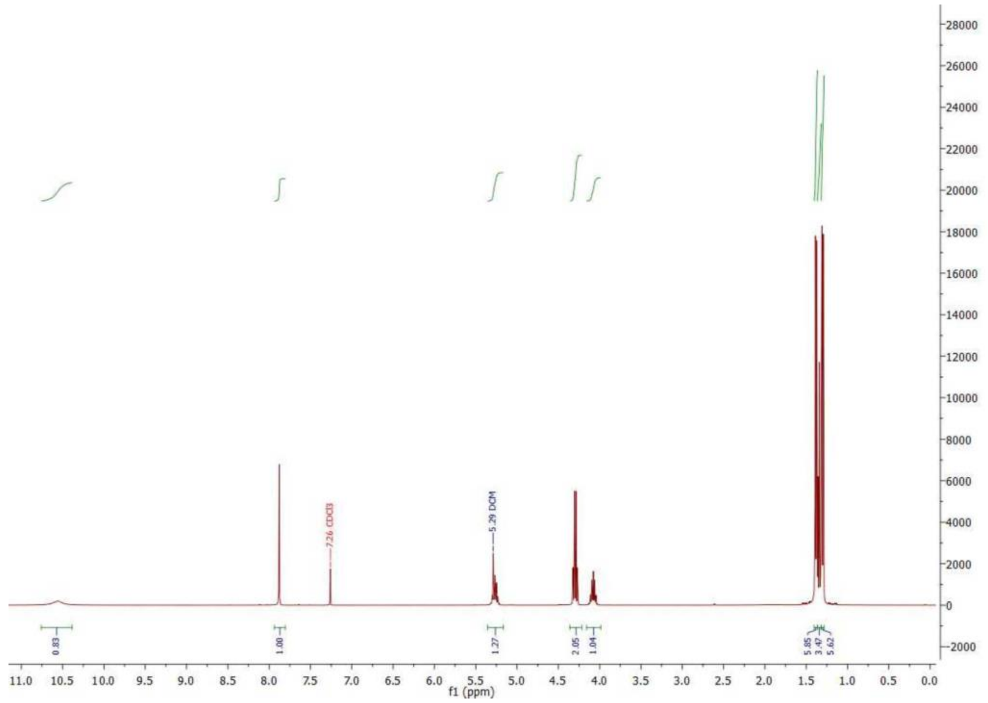

CHMO:0000595 | ^{13}C nuclear magnetic resonance spectroscopy (^{13}C NMR)

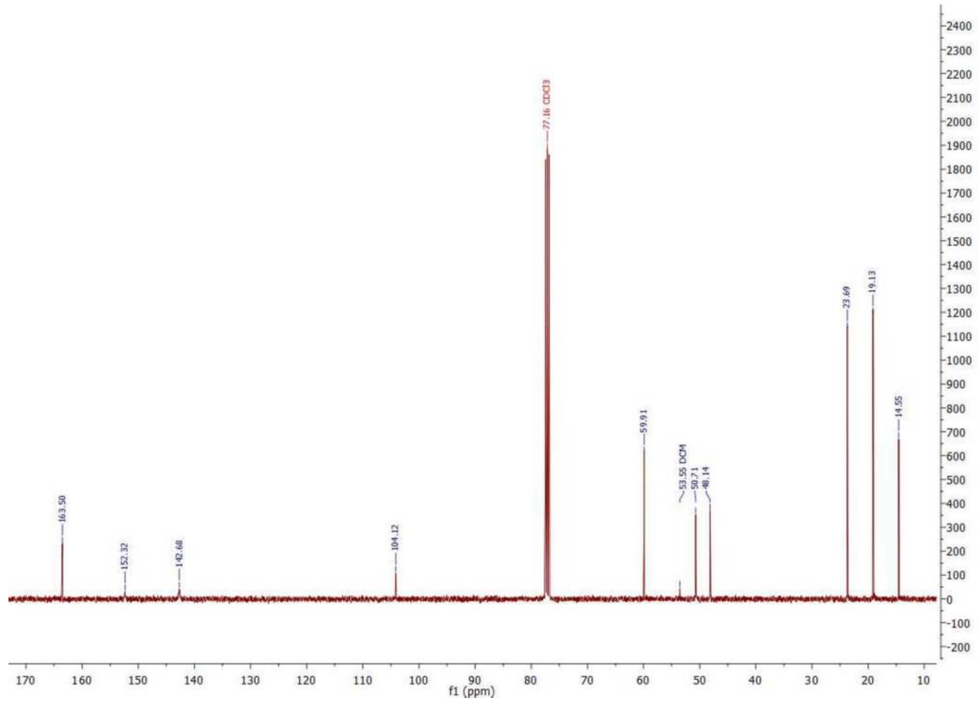

[**15d**] (*E*)-3-(3,3-Diisopropyltriaz-1-en-1-yl)-1*H*-pyrazole-4-carbonitrile

CHMO:0000593 | ^{1}H nuclear magnetic resonance spectroscopy (^{1}H NMR)

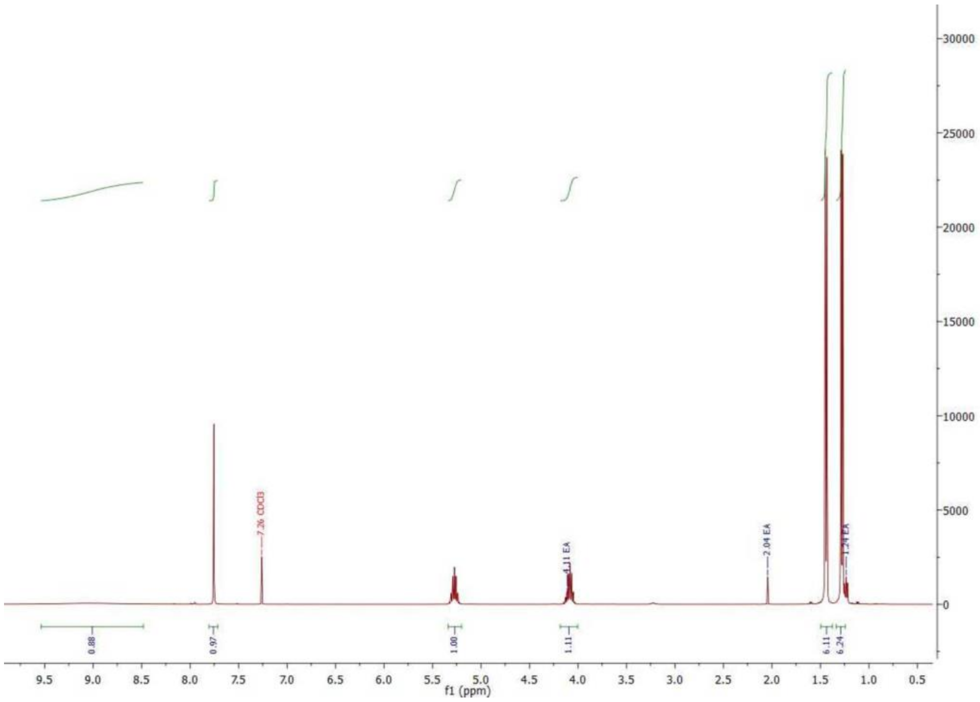

CHMO:0000595 | ^{13}C nuclear magnetic resonance spectroscopy (^{13}C NMR)

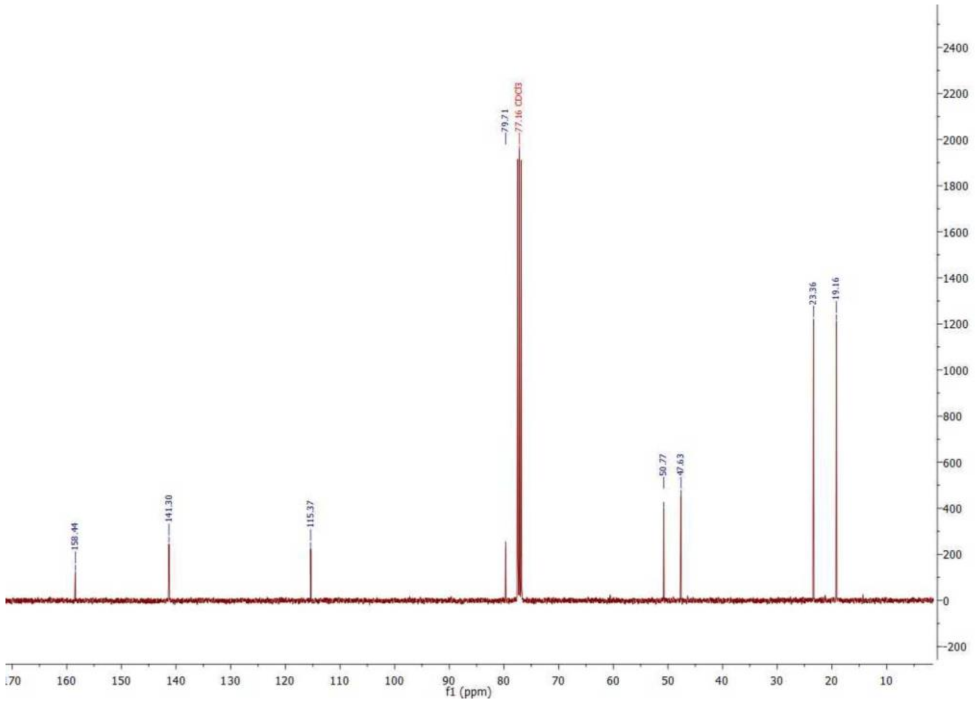

[**17a**] (*E*)-1-benzyl-5-(3,3-diisopropyltriaz-1-en-1-yl)-1*H*-pyrazole

CHMO:0000593 | ^{1}H nuclear magnetic resonance spectroscopy (^{1}H NMR)

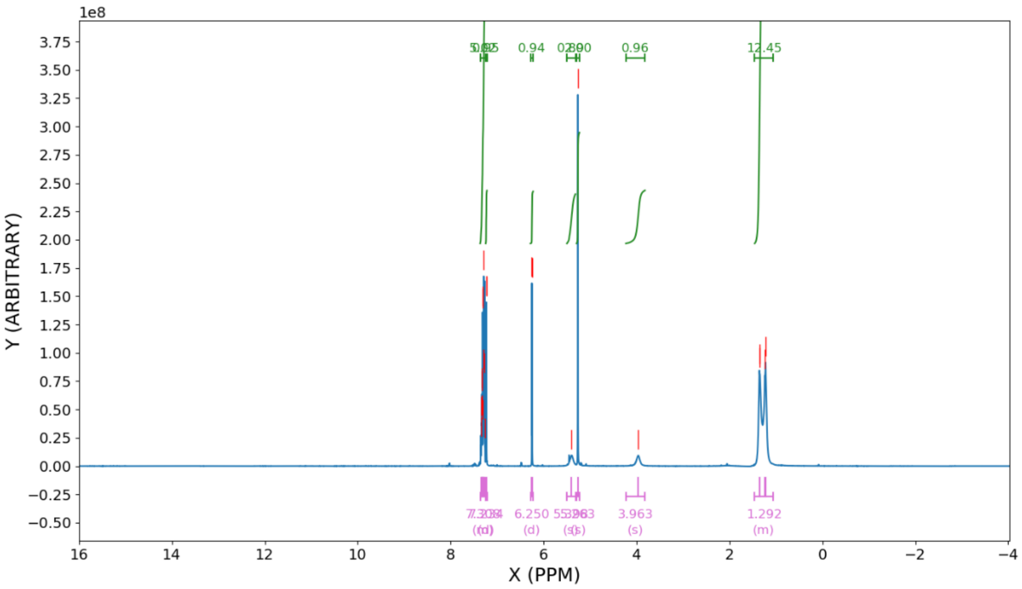

CHMO:0000595 | ^{13}C nuclear magnetic resonance spectroscopy (^{13}C NMR)

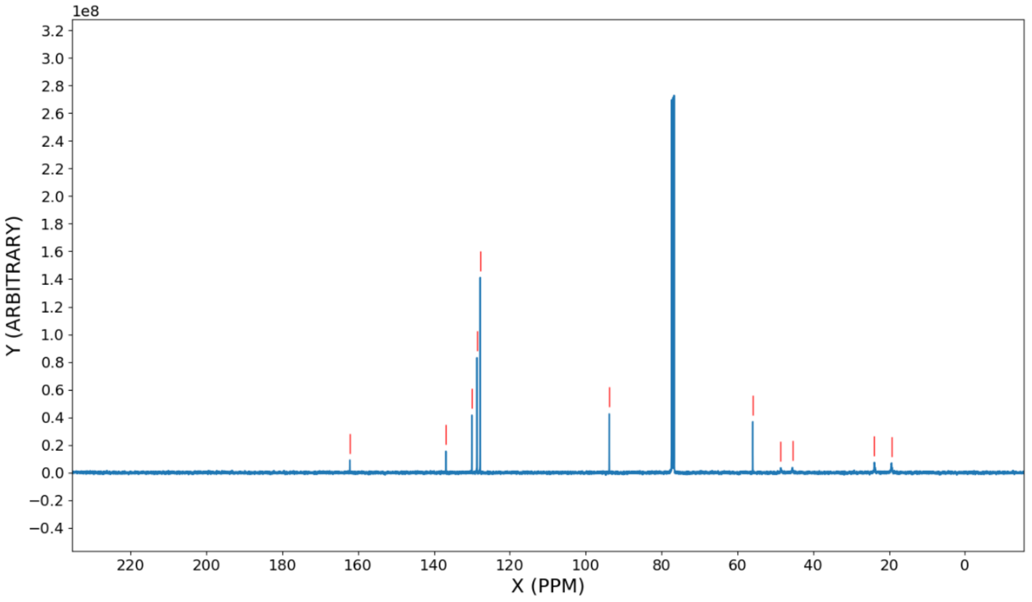

[**18a**] (*E*)-1-benzyl-5-(3,3-diisopropyltriaz-1-en-1-yl)-1*H*-pyrazole

CHMO:0000593 | ^{1}H nuclear magnetic resonance spectroscopy (^{1}H NMR)

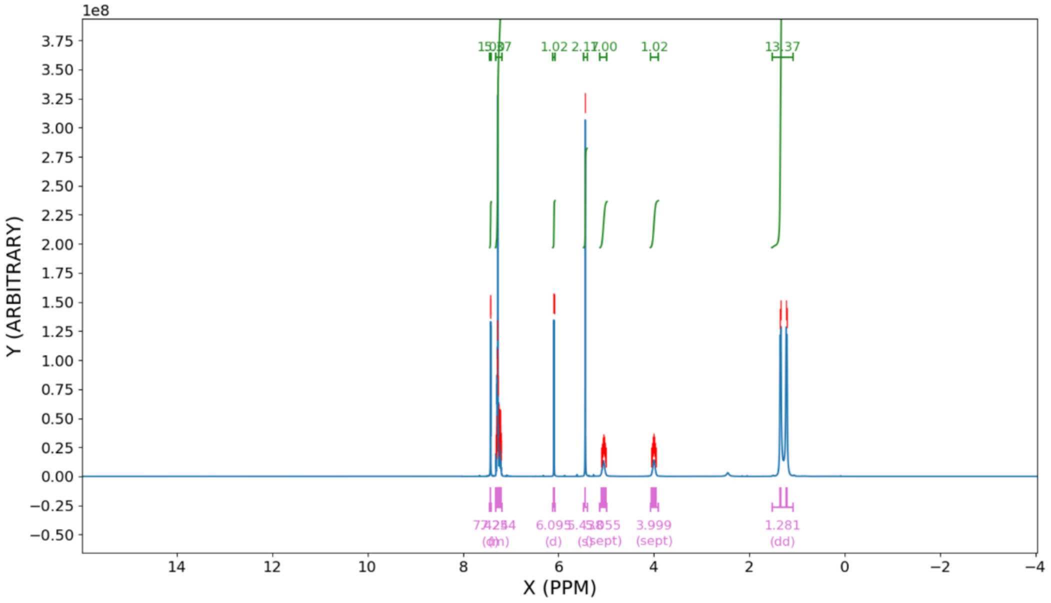

CHMO:0000595 | ^{13}C nuclear magnetic resonance spectroscopy (^{13}C NMR)

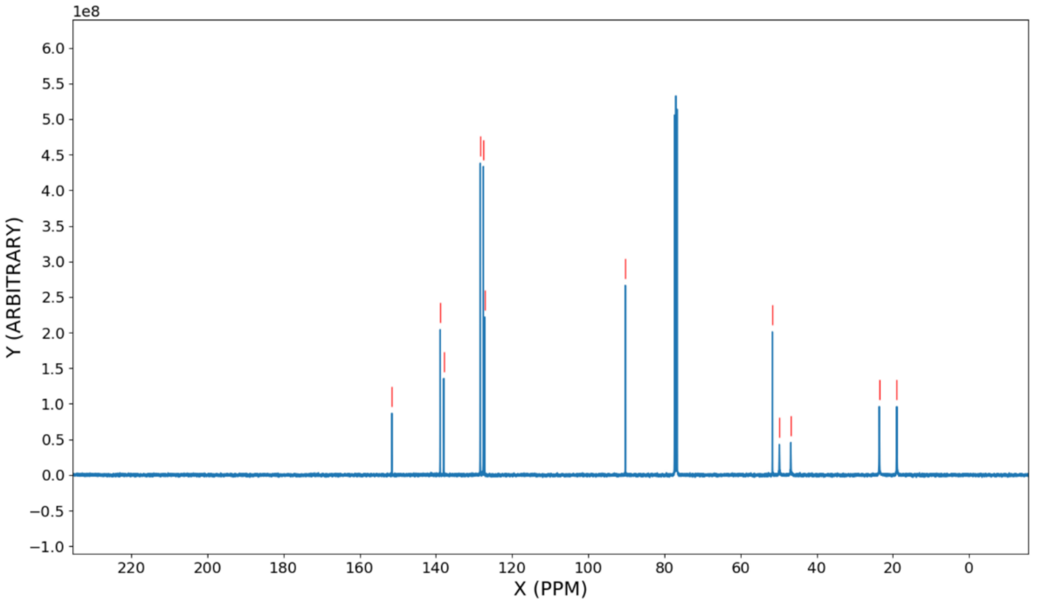

[**17b**] (*E*)-1-(4-bromobenzyl)-3-(3,3-diisopropyltriaz-1-en-1-yl)-1*H*-pyrazole

CHMO:0000593 | ^{1}H nuclear magnetic resonance spectroscopy (^{1}H NMR)

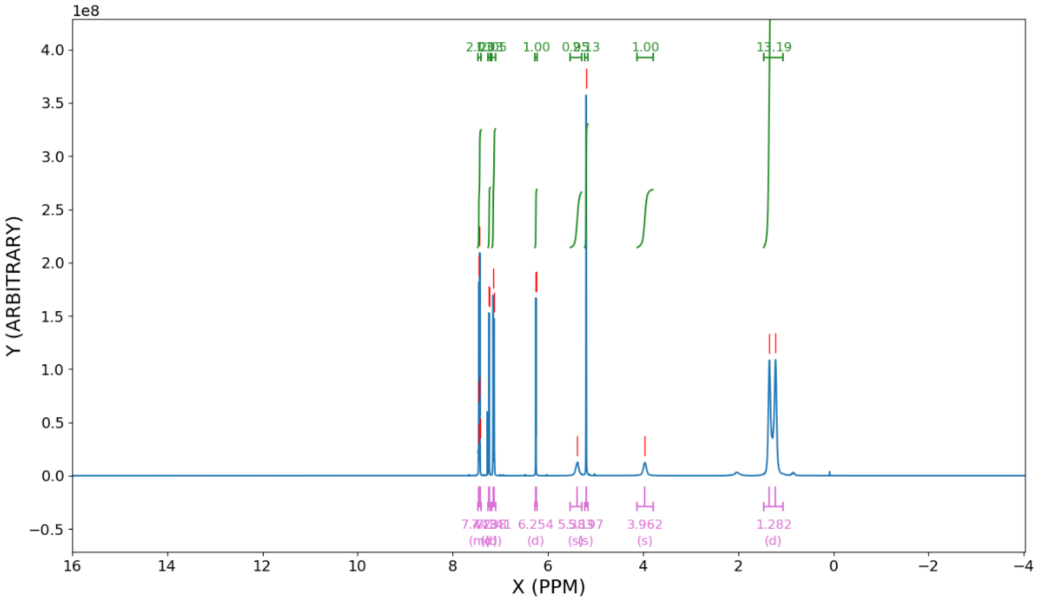

CHMO:0000595 | ^{13}C nuclear magnetic resonance spectroscopy (^{13}C NMR)

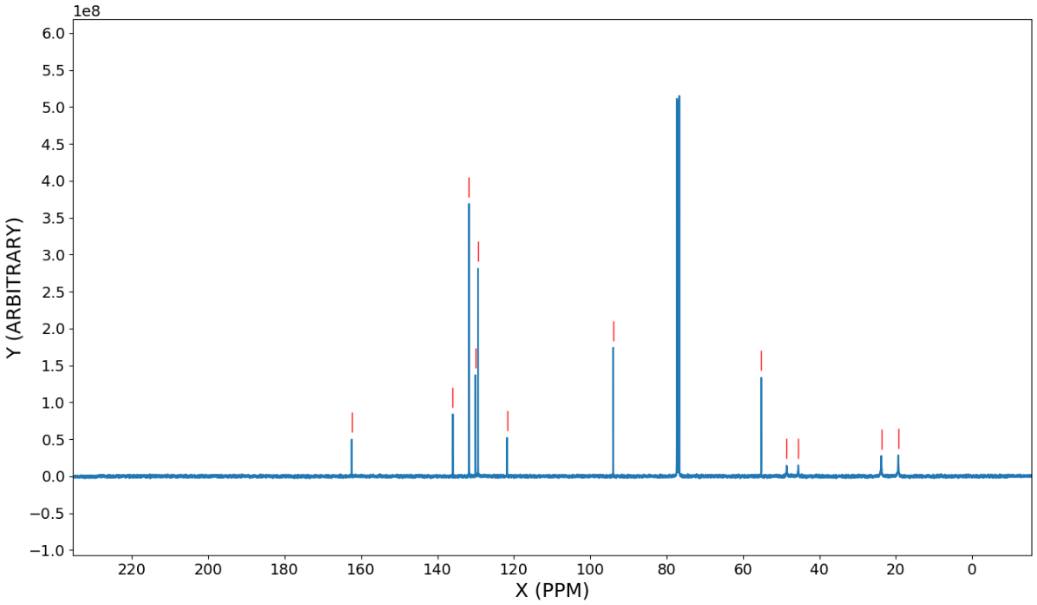

[**18b**] (*E*)-1-(4-bromobenzyl)-5-(3,3-diisopropyltriaz-1-en-1-yl)-1*H*-pyrazole

CHMO:0000593 | ^{1}H nuclear magnetic resonance spectroscopy (^{1}H NMR)

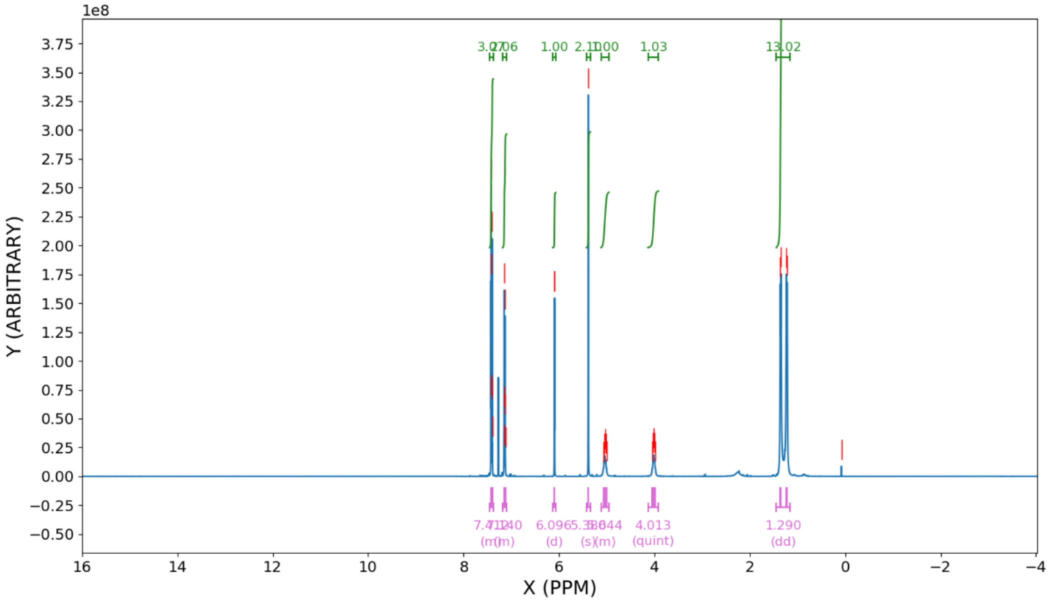

CHMO:0000595 | ^{13}C nuclear magnetic resonance spectroscopy (^{13}C NMR)

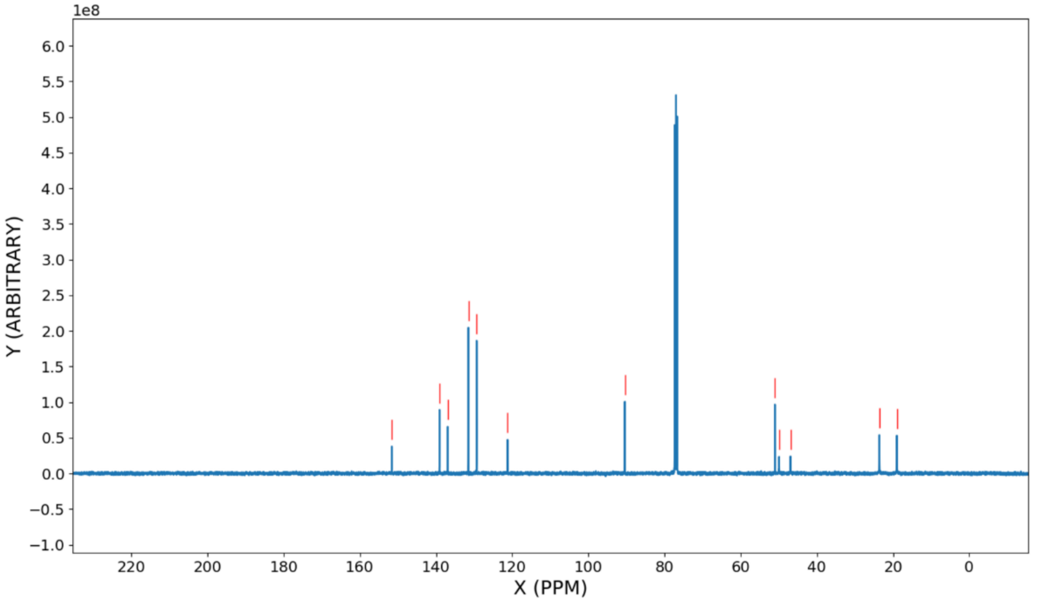

[**17c**] (*E*)-1-cyclopentyl-5-(3,3-diisopropyltriaz-1-en-1-yl)-1*H*-pyrazole

CHMO:0000593 | ^{1}H nuclear magnetic resonance spectroscopy (^{1}H NMR)

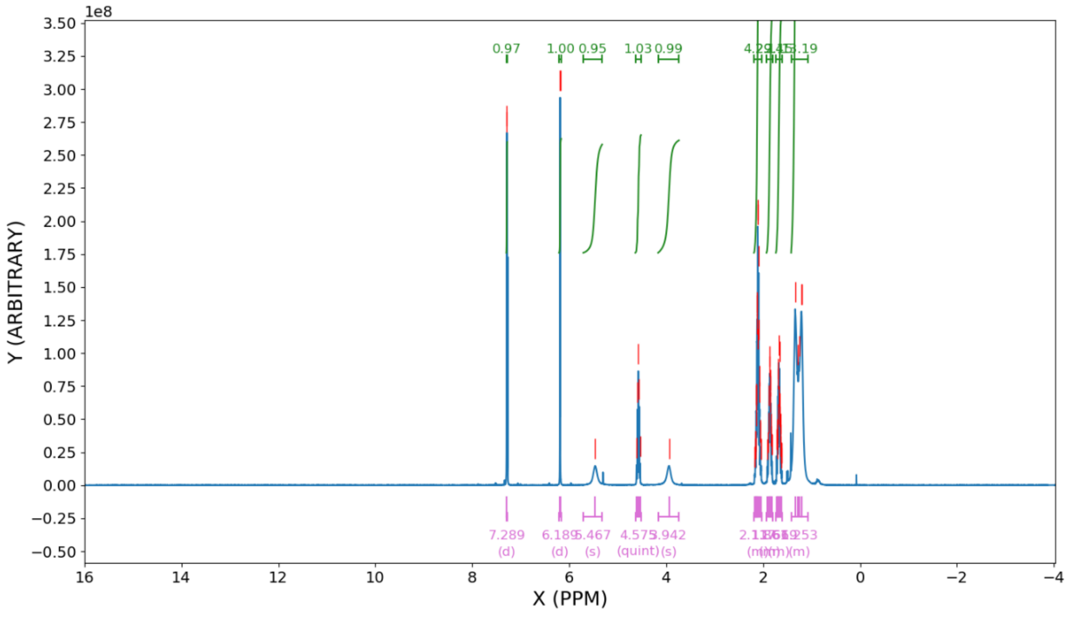

CHMO:0000595 | ^{13}C nuclear magnetic resonance spectroscopy (^{13}C NMR)

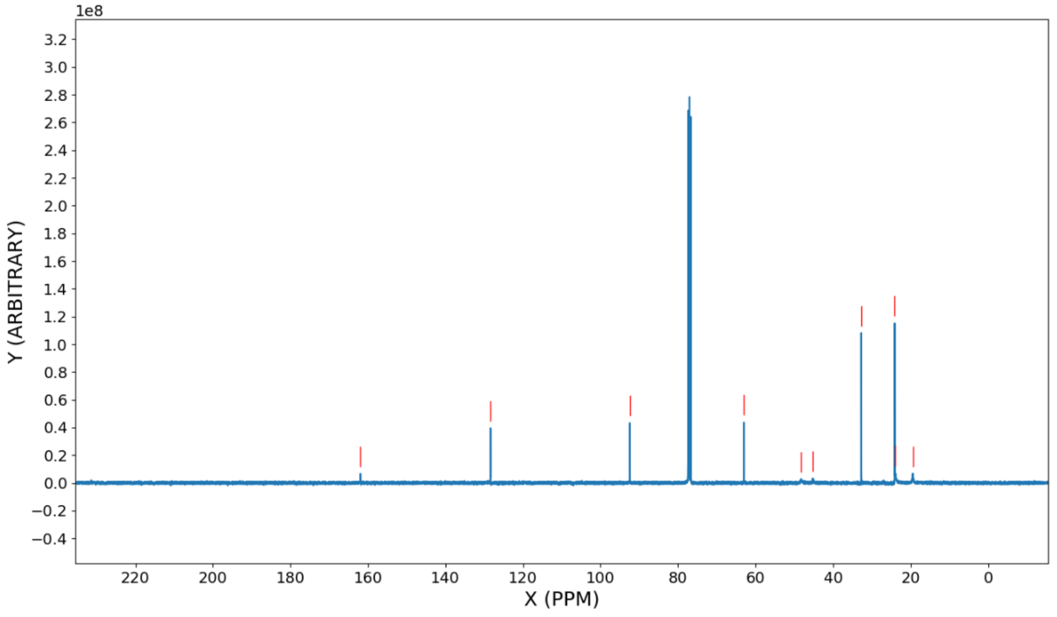

[**18c**] (*E*)-1-cyclopentyl-5-(3,3-diisopropyltriaz-1-en-1-yl)-1*H*-pyrazole

CHMO:0000593 | ^{1}H nuclear magnetic resonance spectroscopy (^{1}H NMR)

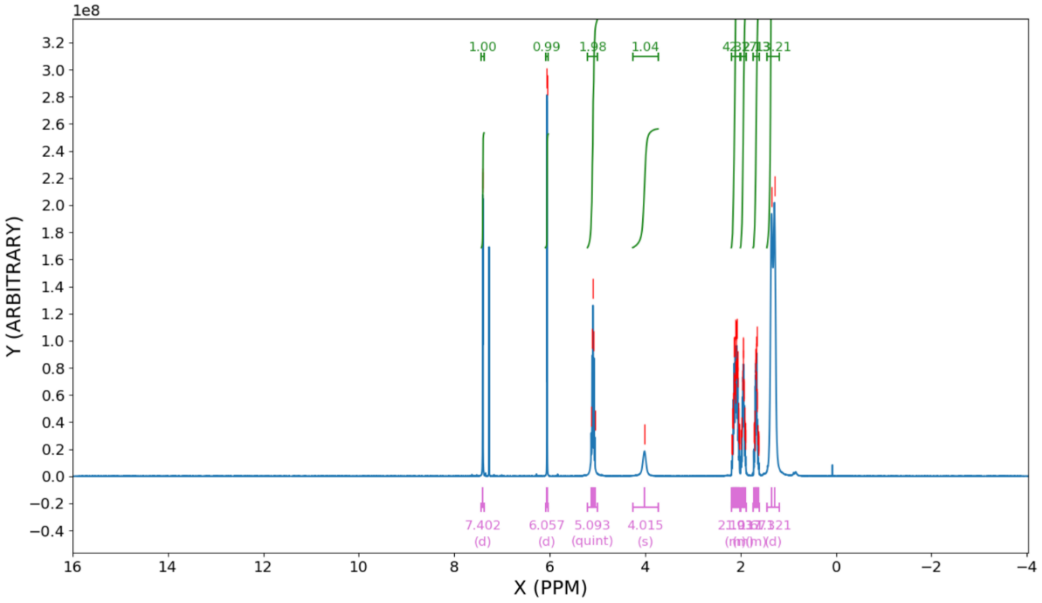

CHMO:0000595 | ^{13}C nuclear magnetic resonance spectroscopy (^{13}C NMR)

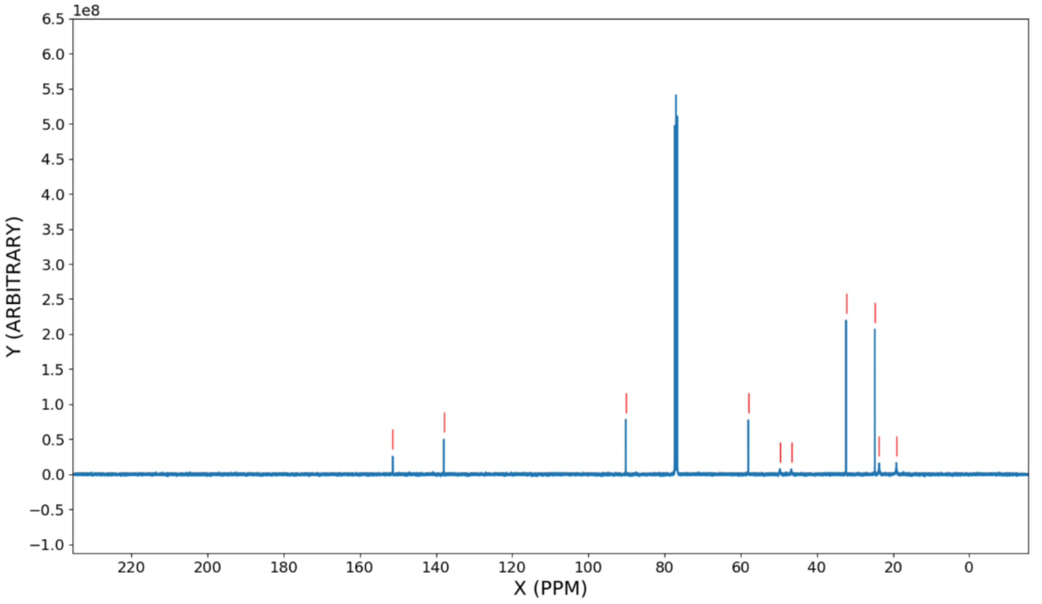

[**17d**] (*E*)-1-benzyl-3-(3,3-diisopropyltriaz-1-en-1-yl)-3-methyl-1*H*-pyrazole

CHMO:0000593 | ^{1}H nuclear magnetic resonance spectroscopy (^{1}H NMR)

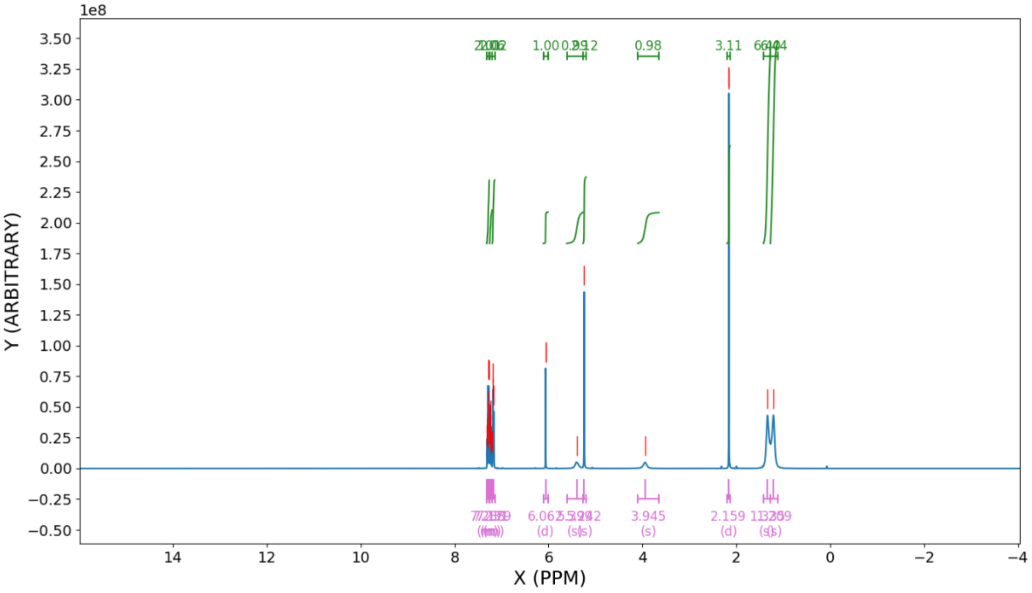

CHMO:0000595 | ^{13}C nuclear magnetic resonance spectroscopy (^{13}C NMR)

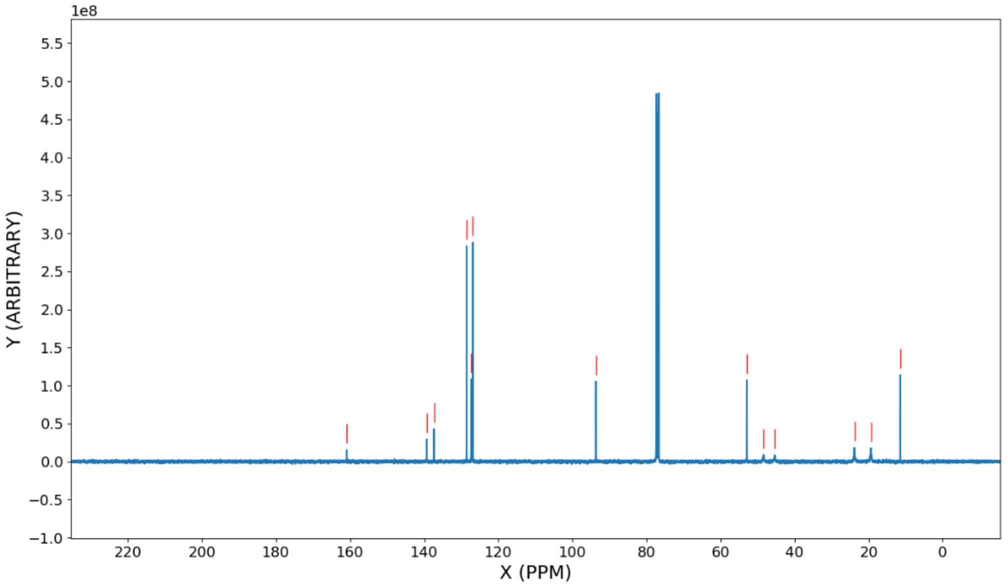

[**18d**] (*E*)-1-benzyl-5-(3,3-diisopropyltriaz-1-en-1-yl)-3-methyl-1*H*-pyrazole

CHMO:0000593 | ^{1}H nuclear magnetic resonance spectroscopy (^{1}H NMR)

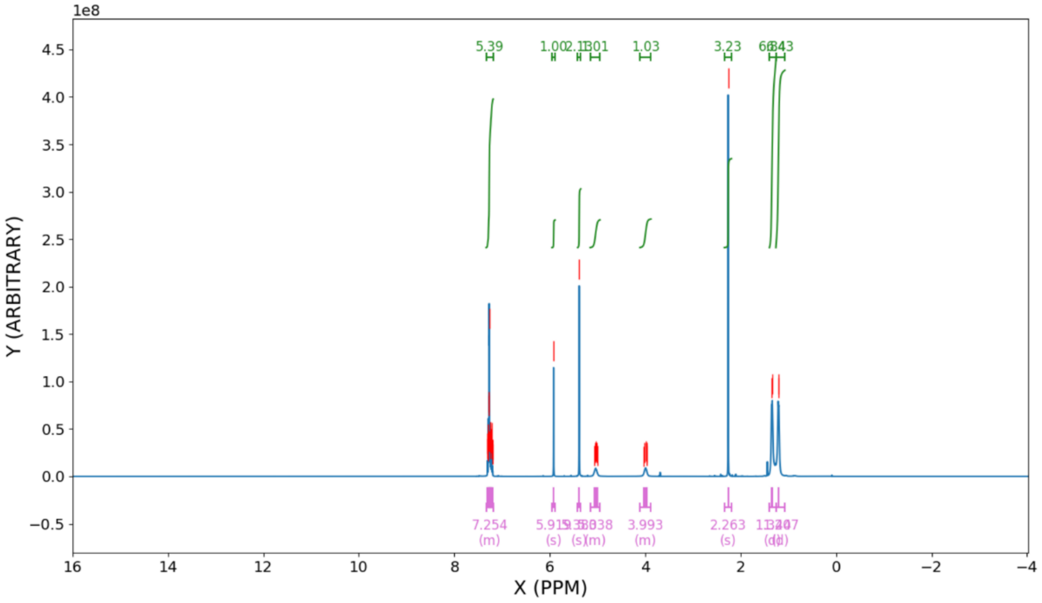

CHMO:0000595 | ^{13}C nuclear magnetic resonance spectroscopy (^{13}C NMR)

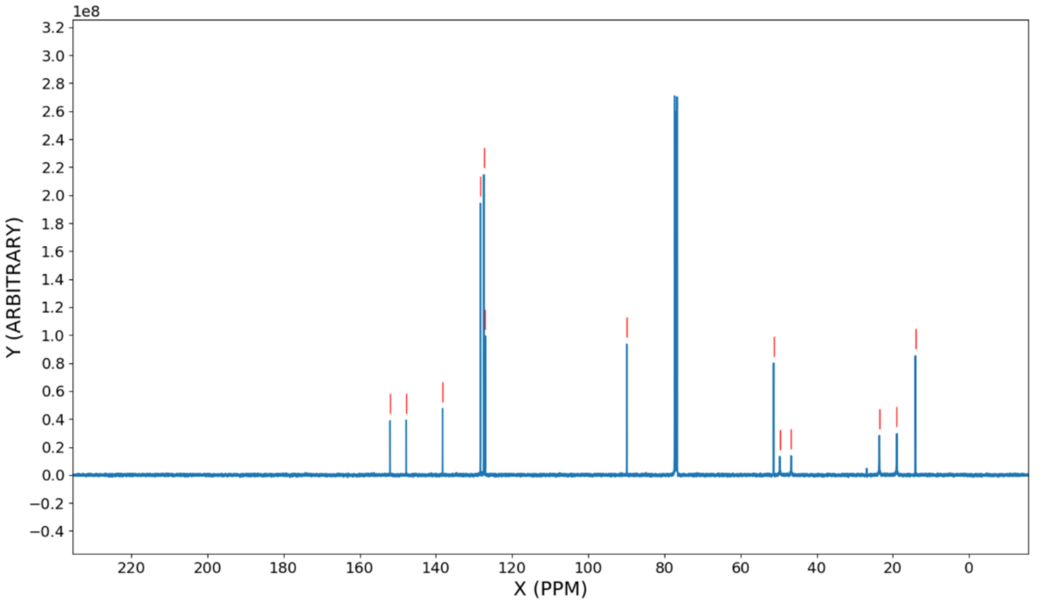

[**17e**] (*E*)-4-(3-(3,3-diisopropyltriaz-1-en-1-yl)-3-methyl-1*H*-pyrazol-1-yl)benzonitrile

CHMO:0000593 | ^{1}H nuclear magnetic resonance spectroscopy (^{1}H NMR)

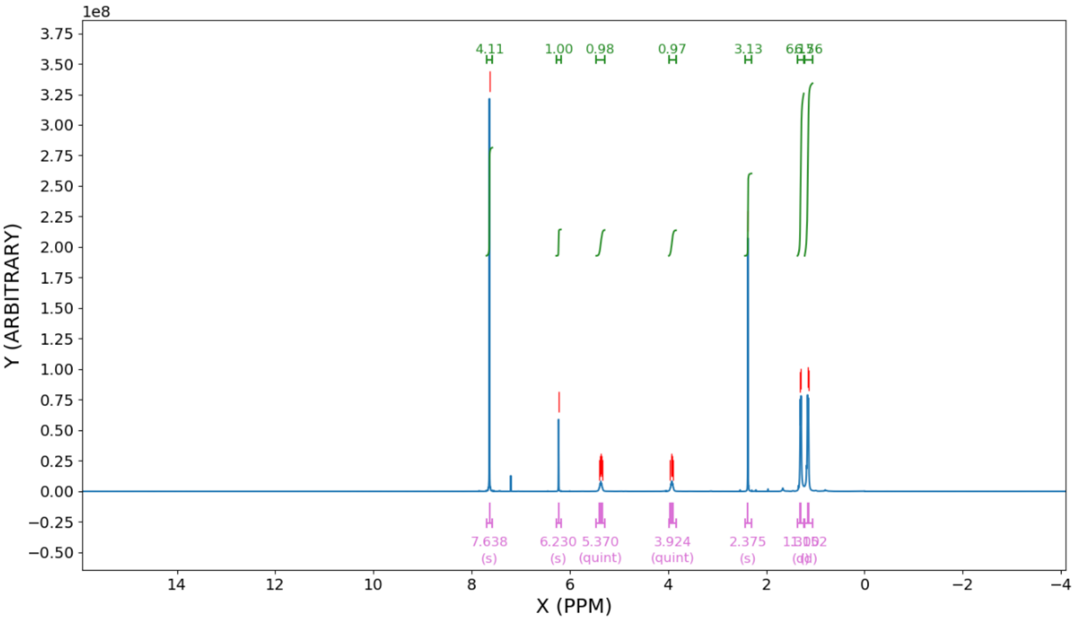

CHMO:0000595 | ^{13}C nuclear magnetic resonance spectroscopy (^{13}C NMR)

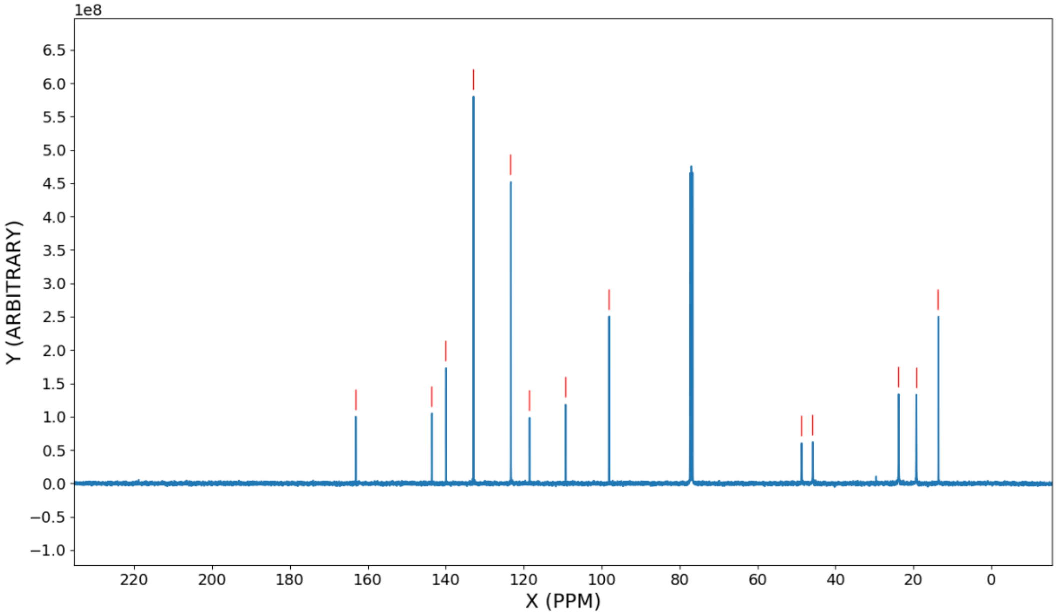

[**18e**] (*E*)-4-(5-(3,3-diisopropyltriaz-1-en-1-yl)-3-methyl-1*H*-pyrazol-1-yl)benzonitrile

CHMO:0000593 | ^{1}H nuclear magnetic resonance spectroscopy (^{1}H NMR)

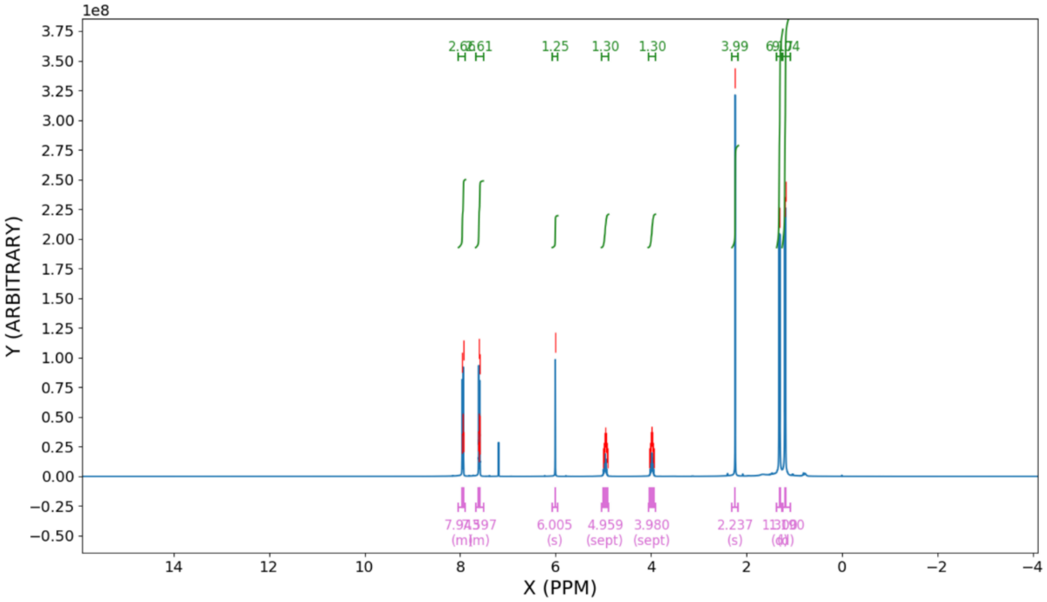

CHMO:0000595 | ^{13}C nuclear magnetic resonance spectroscopy (^{13}C NMR)

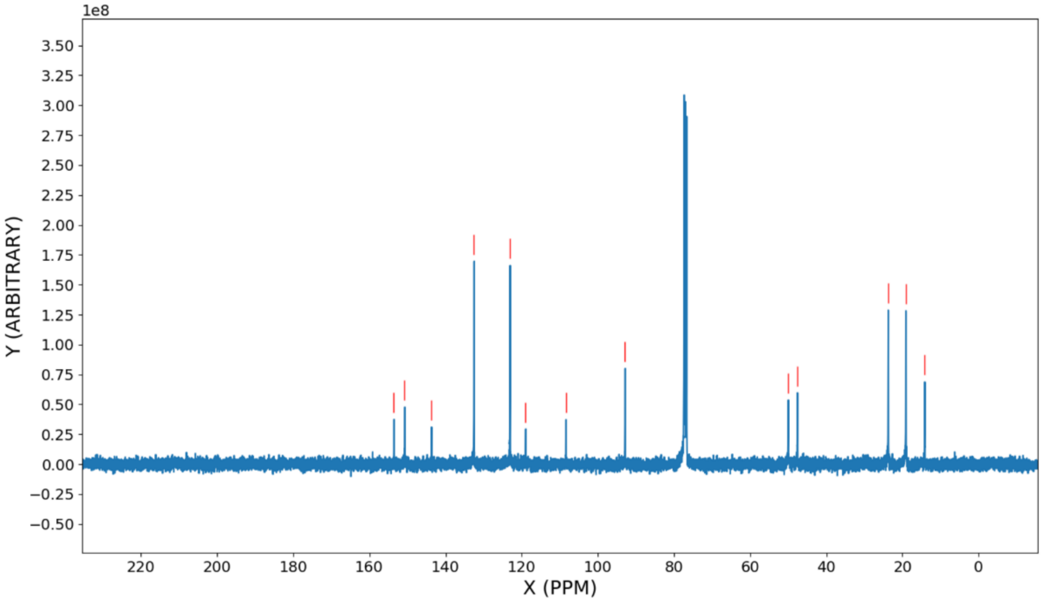

[**17f**] (*E*)-3-(3,3-diisopropyltriaz-1-en-1-yl)-3-methyl-1-(4-nitrophenyl)-1*H*-pyrazole

CHMO:0000593 | ^{1}H nuclear magnetic resonance spectroscopy (^{1}H NMR)

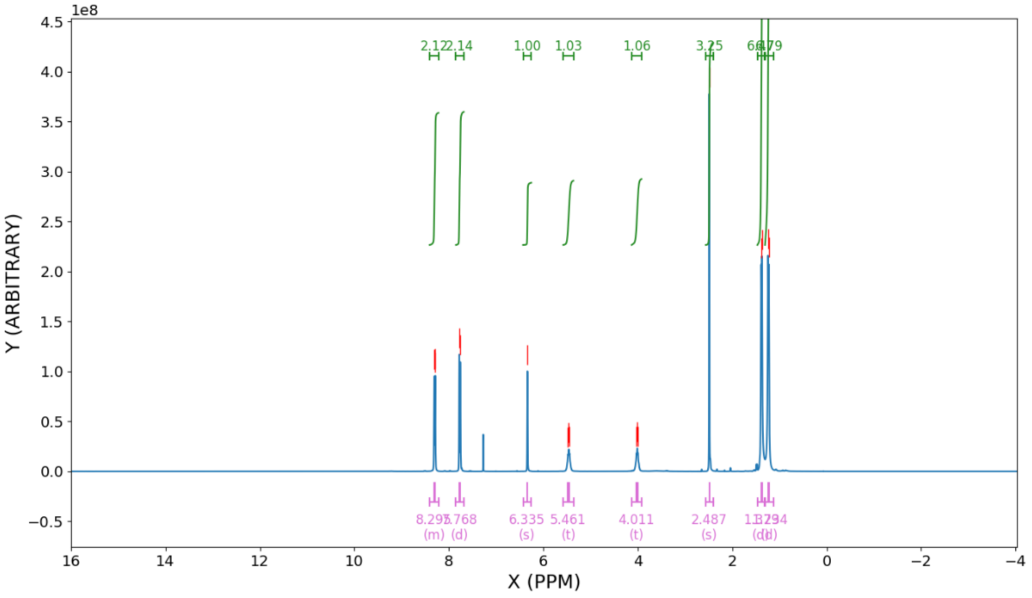

CHMO:0000595 | ^{13}C nuclear magnetic resonance spectroscopy (^{13}C NMR)

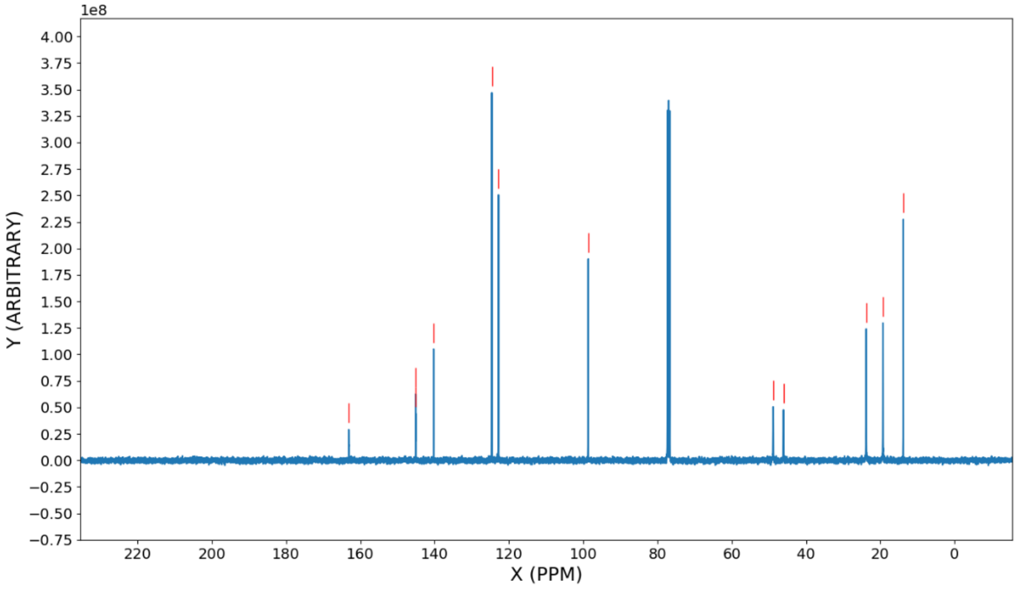

[**18f**] (*E*)-5-(3,3-diisopropyltriaz-1-en-1-yl)-3-methyl-1-(4-nitrophenyl)-1*H*-pyrazole

CHMO:0000593 | ^{1}H nuclear magnetic resonance spectroscopy (^{1}H NMR)

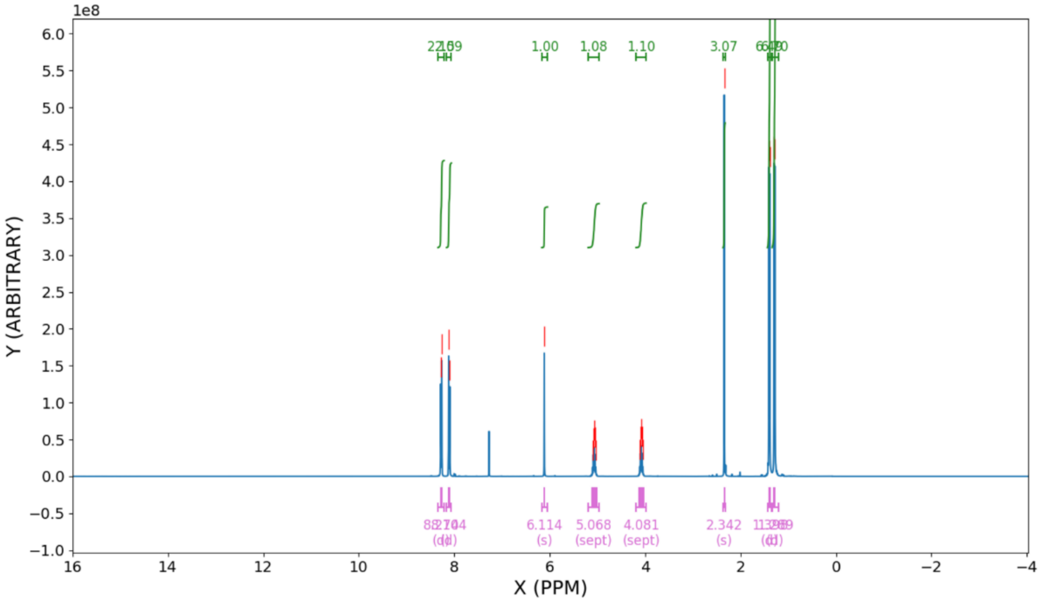

CHMO:0000595 | ^{13}C nuclear magnetic resonance spectroscopy (^{13}C NMR)

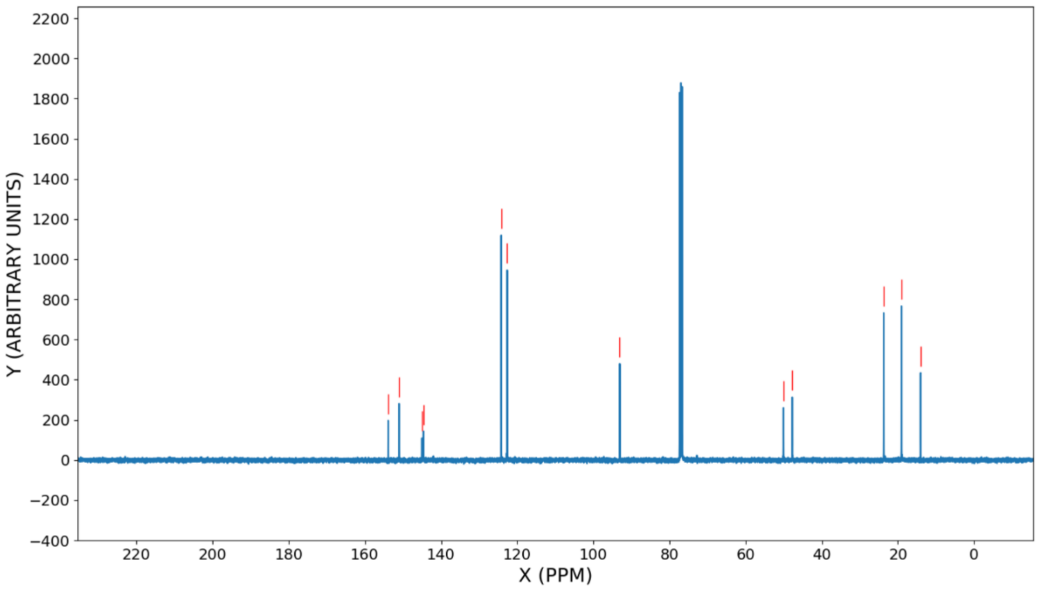

[**17g**] (*E*)-1-(2-chloro-4-nitrophenyl)-3-(3,3-diisopropyltriaz-1-en-1-yl)-3-methyl-1*H*-pyrazole

CHMO:0000593 | ^{1}H nuclear magnetic resonance spectroscopy (^{1}H NMR)

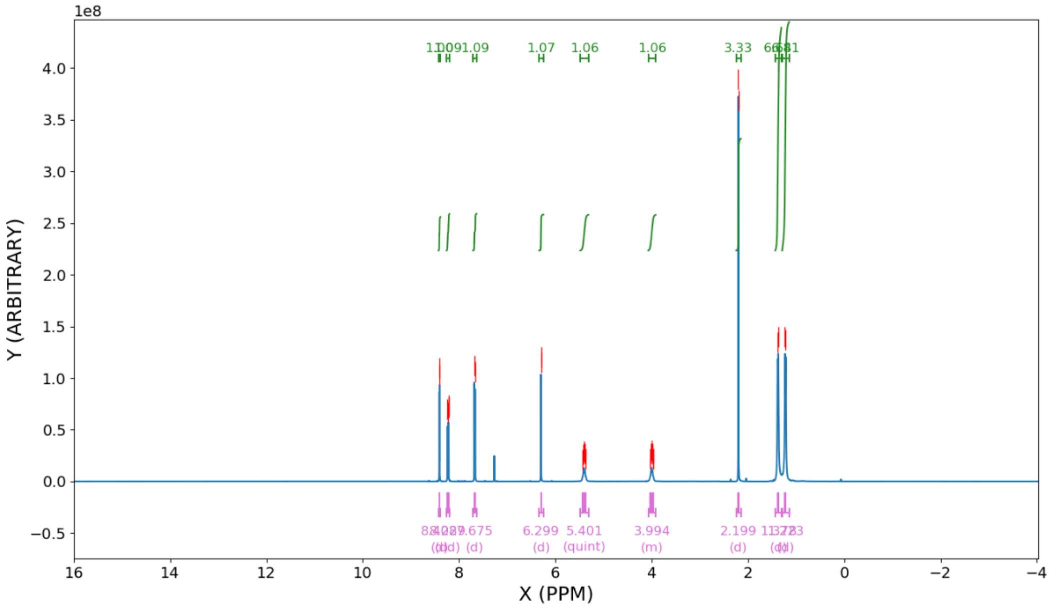

CHMO:0000595 | ^{13}C nuclear magnetic resonance spectroscopy (^{13}C NMR)

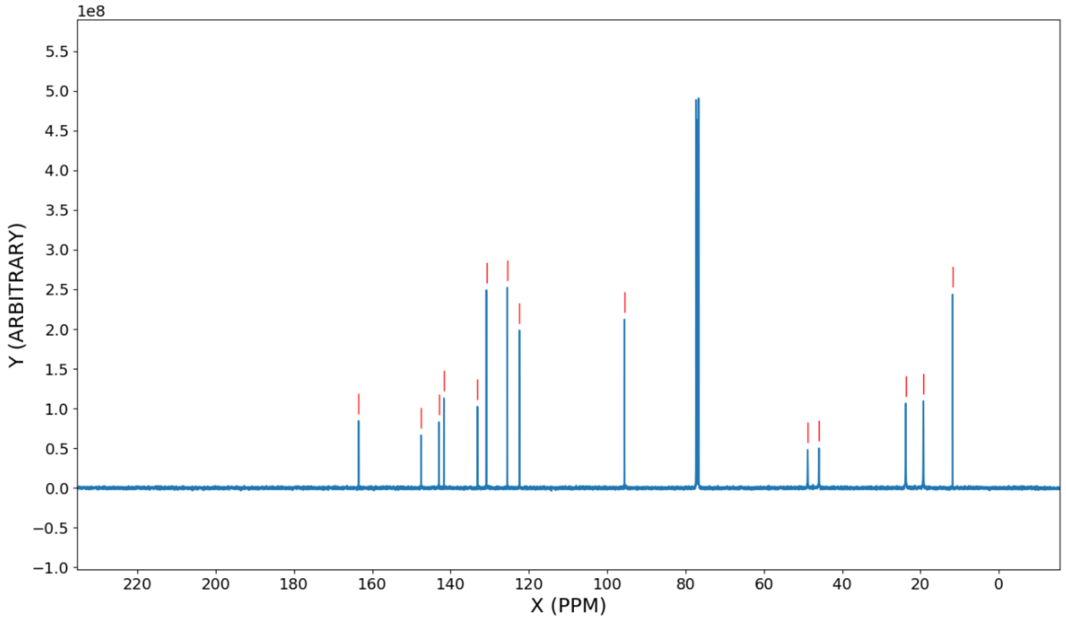

[**18g**] (*E*)-1-(2-chloro-4-nitrophenyl)-5-(3,3-diisopropyltriaz-1-en-1-yl)-3-methyl-1*H*-pyrazole

CHMO:0000593 | ^{1}H nuclear magnetic resonance spectroscopy (^{1}H NMR)

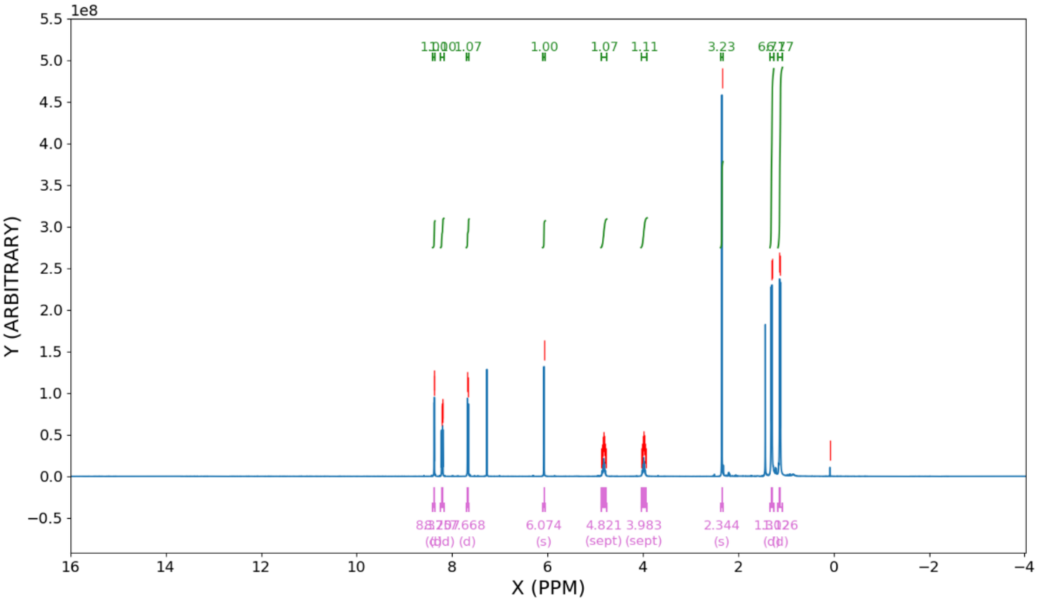

CHMO:0000595 | ^{13}C nuclear magnetic resonance spectroscopy (^{13}C NMR)

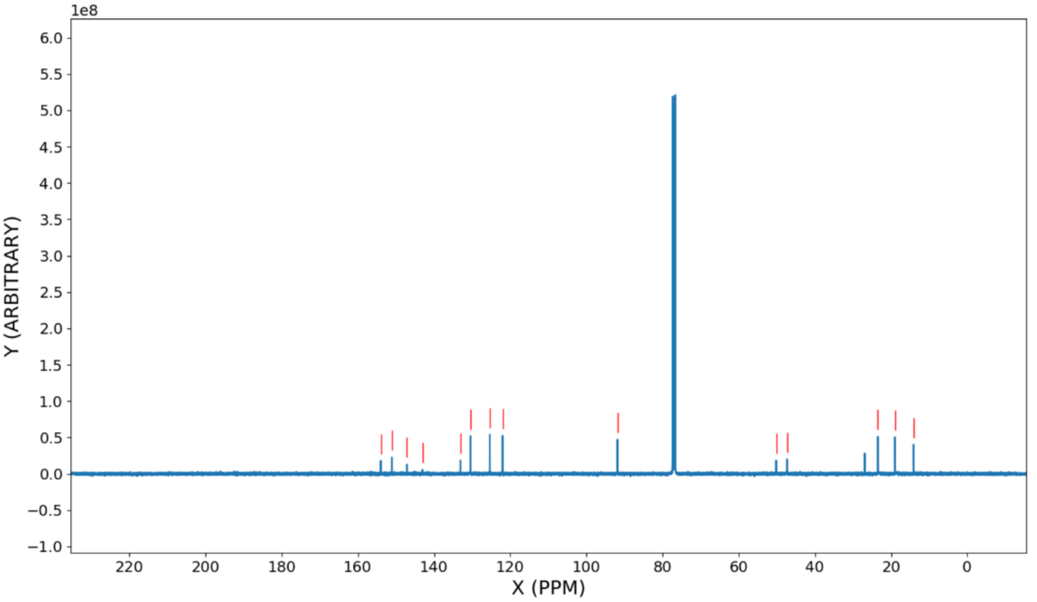

[**17h**] ethyl (*E*)-1-benzyl-3-(3,3-diisopropyltriaz-1-en-1-yl)-1*H*-pyrazole-4-carboxylate

CHMO:0000593 | ^{1}H nuclear magnetic resonance spectroscopy (^{1}H NMR)

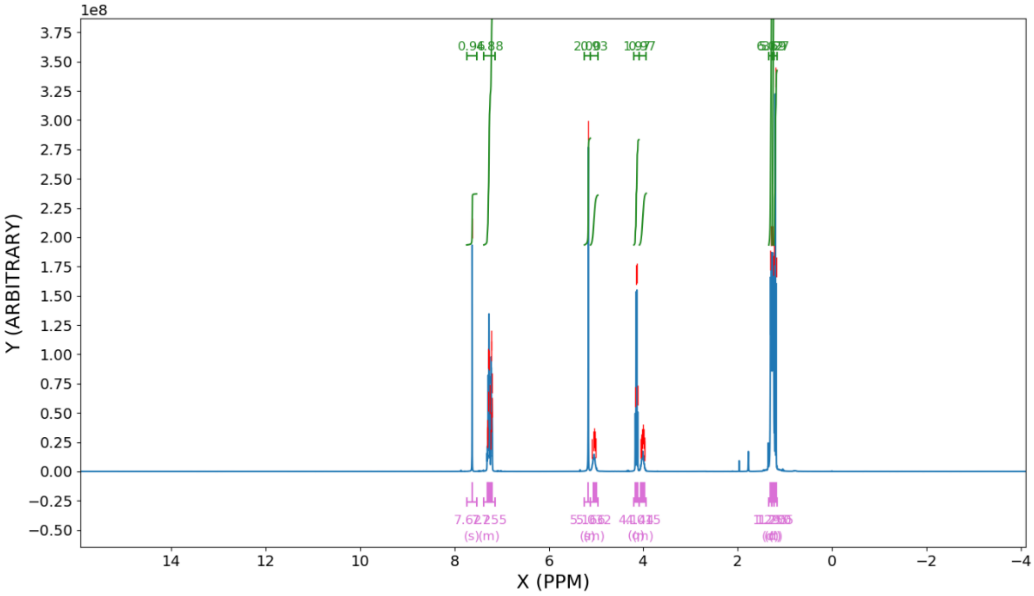

CHMO:0000595 | ^{13}C nuclear magnetic resonance spectroscopy (^{13}C NMR)

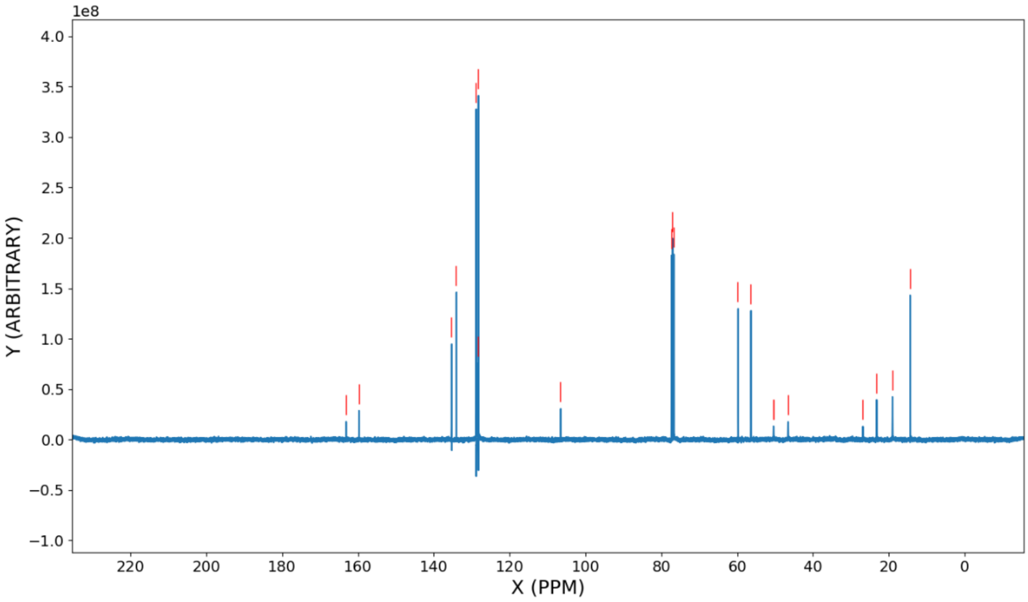

[**18h**] ethyl (*E*)-1-benzyl-5-(3,3-diisopropyltriaz-1-en-1-yl)-1*H*-pyrazole-4-carboxylate

CHMO:0000593 | ^{1}H nuclear magnetic resonance spectroscopy (^{1}H NMR)

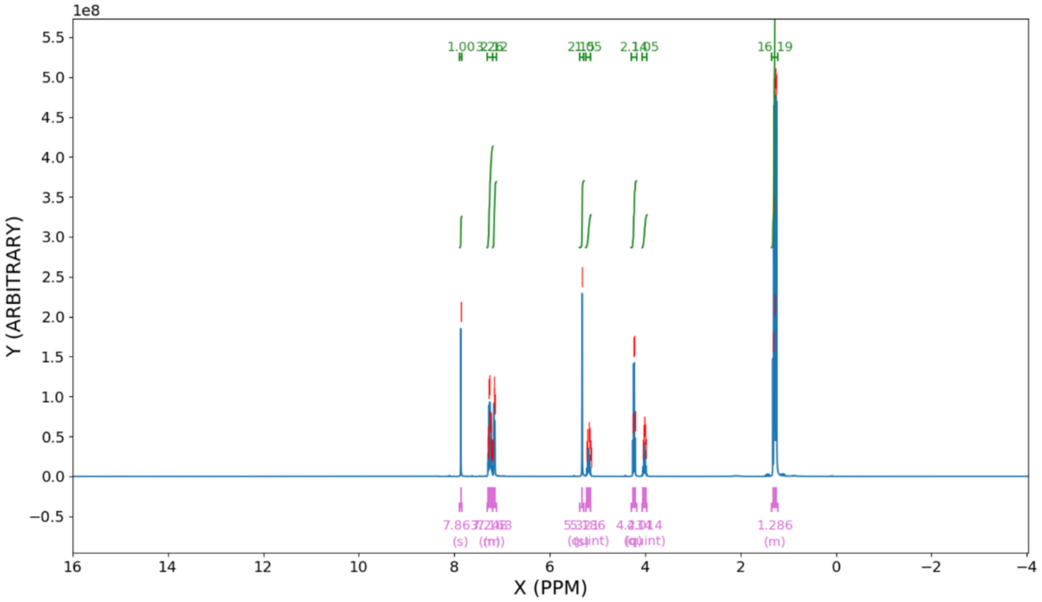

CHMO:0000595 | ^{13}C nuclear magnetic resonance spectroscopy (^{13}C NMR)

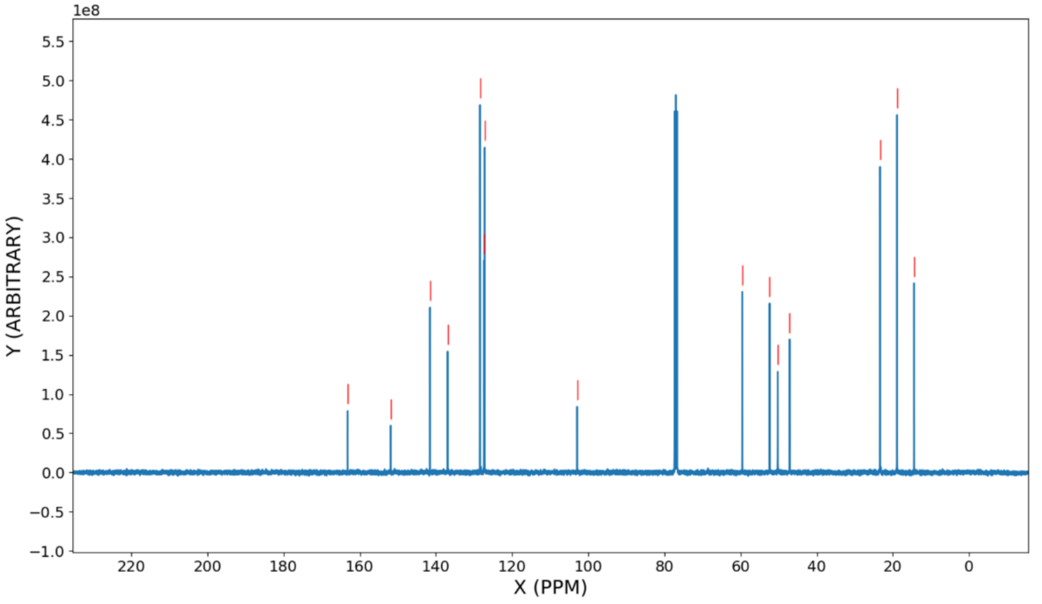

[**17i**] ethyl (*E*)-1-(3,5-difluorobenzyl)-3-(3,3-diisopropyltriaz-1-en-1-yl)-1*H*-pyrazole-4-carboxylate

CHMO:0000593 | ^{1}H nuclear magnetic resonance spectroscopy (^{1}H NMR)

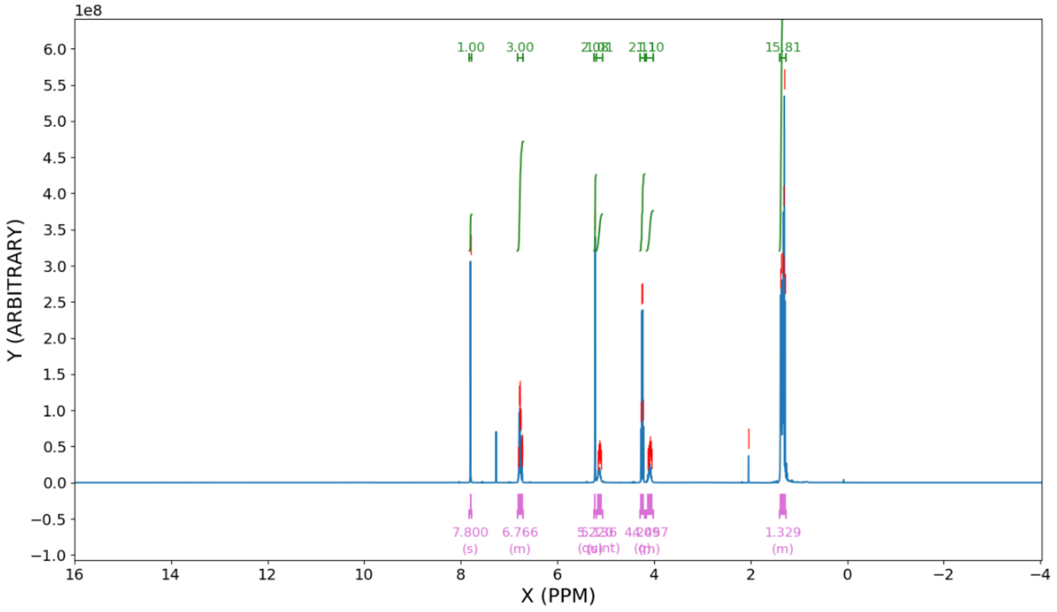

CHMO:0000595 | ^{13}C nuclear magnetic resonance spectroscopy (^{13}C NMR)

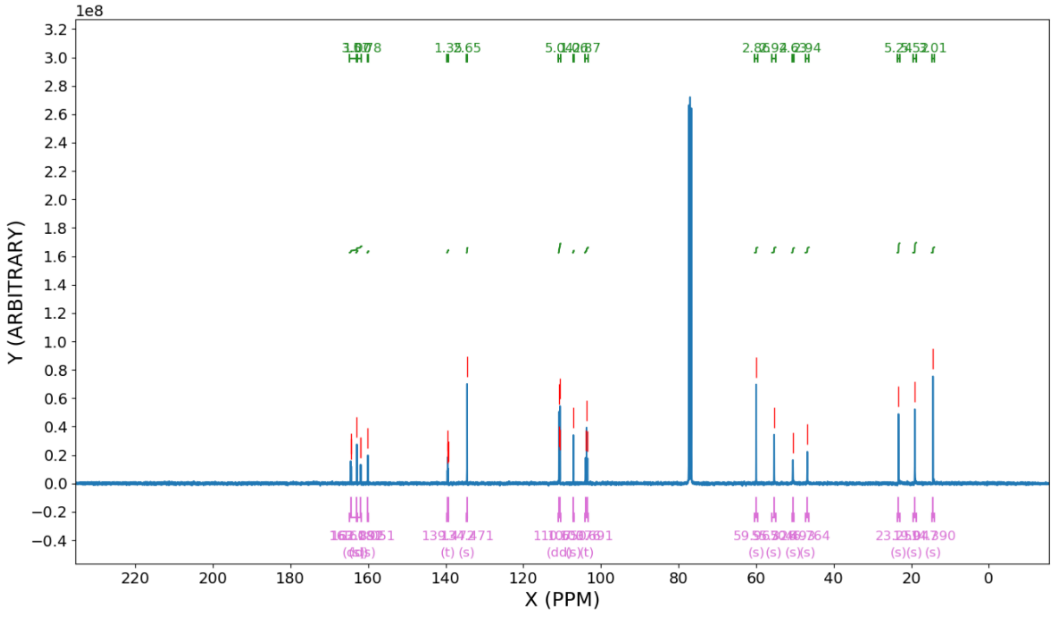

[**18i**] ethyl (*E*)-1-(3,5-difluorobenzyl)-5-(3,3-diisopropyltriaz-1-en-1-yl)-1*H*-pyrazole-4-carboxylate

CHMO:0000593 | ^{1}H nuclear magnetic resonance spectroscopy (^{1}H NMR)

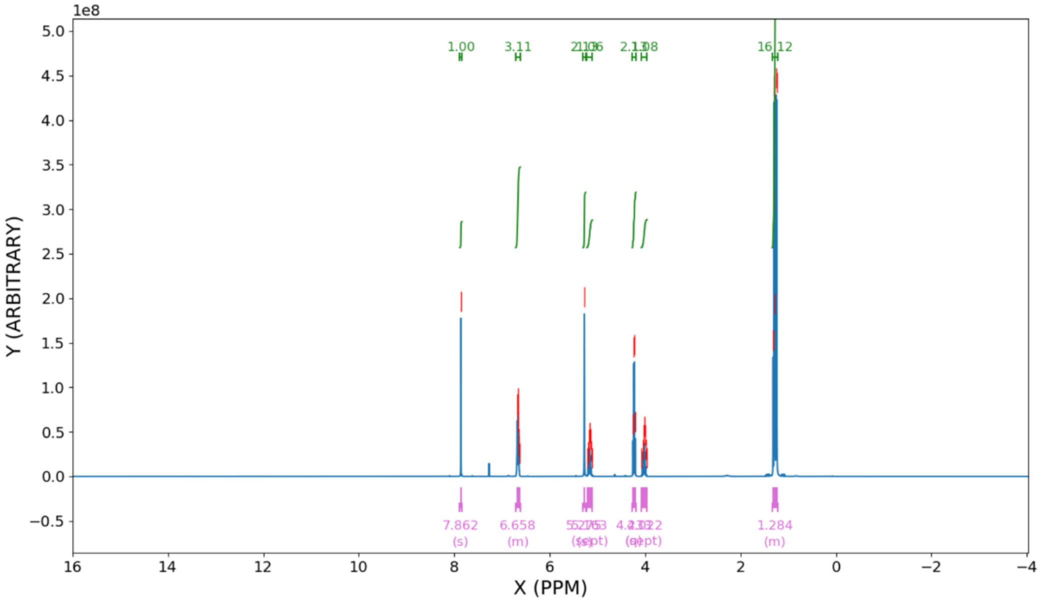

CHMO:0000595 | ^{13}C nuclear magnetic resonance spectroscopy (^{13}C NMR)

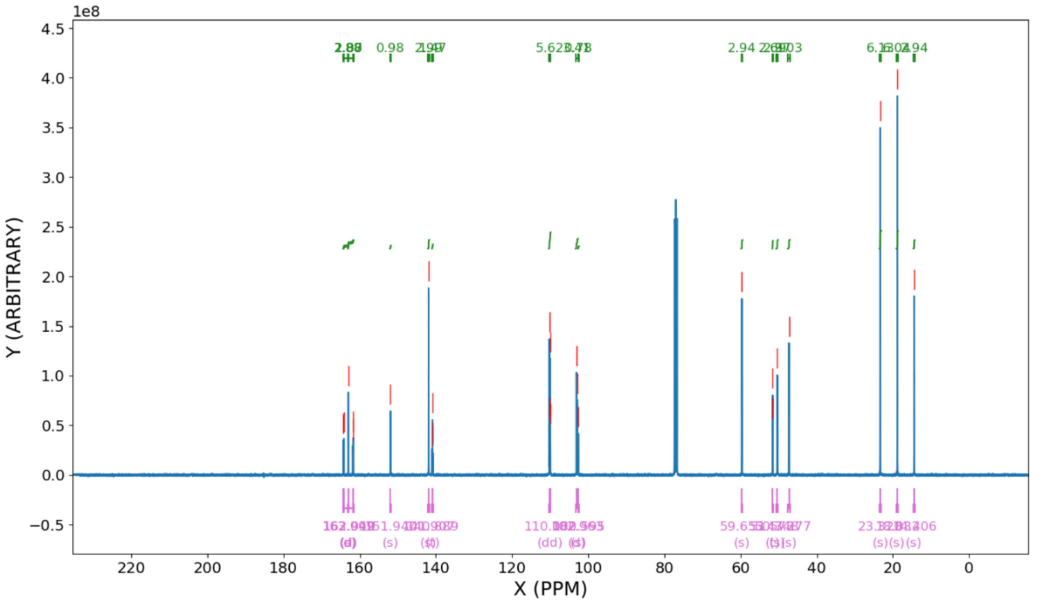

[**17j**] ethyl (*E*)-1-(3-cyanobenzyl)-3-(3,3-diisopropyltriaz-1-en-1-yl)-1*H*-pyrazole-4-carboxylate

CHMO:0000593 | ^{1}H nuclear magnetic resonance spectroscopy (^{1}H NMR)

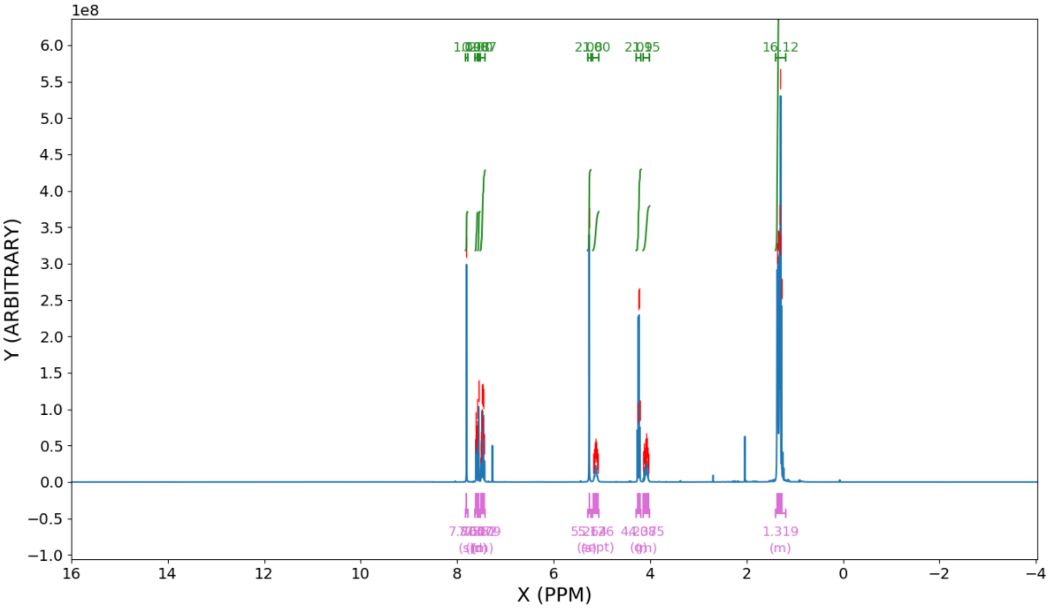

CHMO:0000595 | ^{13}C nuclear magnetic resonance spectroscopy (^{13}C NMR)

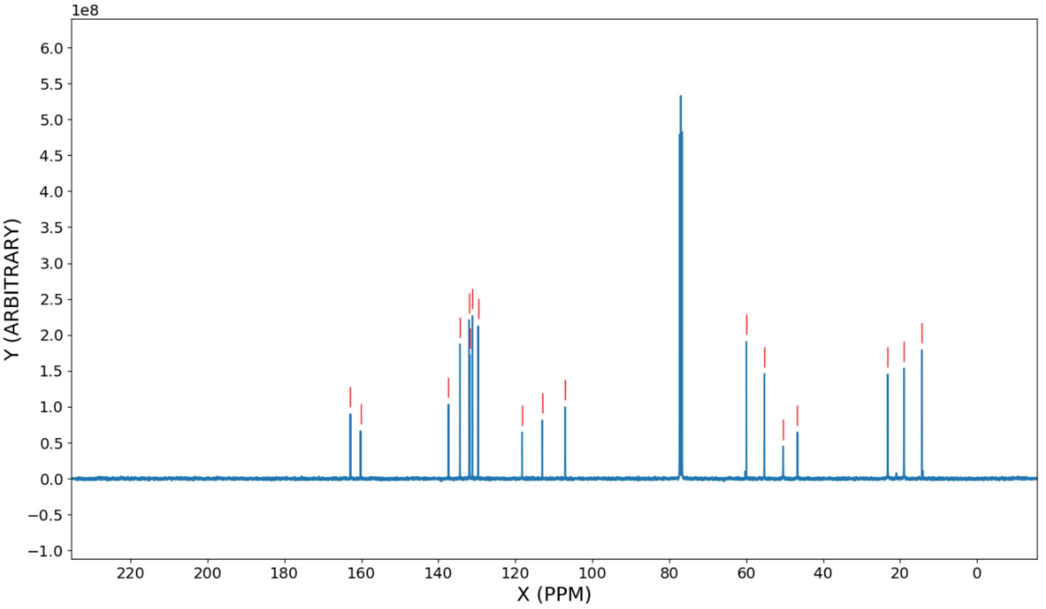

[**18j**] ethyl (*E*)-1-(3-cyanobenzyl)-5-(3,3-diisopropyltriaz-1-en-1-yl)-1*H*-pyrazole-4-carboxylate

CHMO:0000593 | ^{1}H nuclear magnetic resonance spectroscopy (^{1}H NMR)

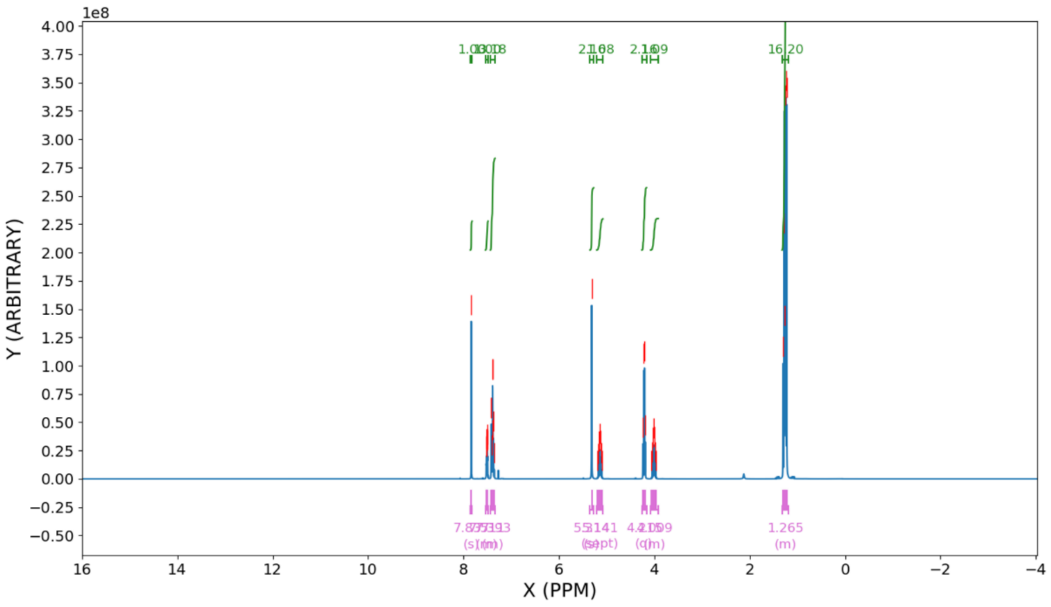

CHMO:0000595 | ^{13}C nuclear magnetic resonance spectroscopy (^{13}C NMR)

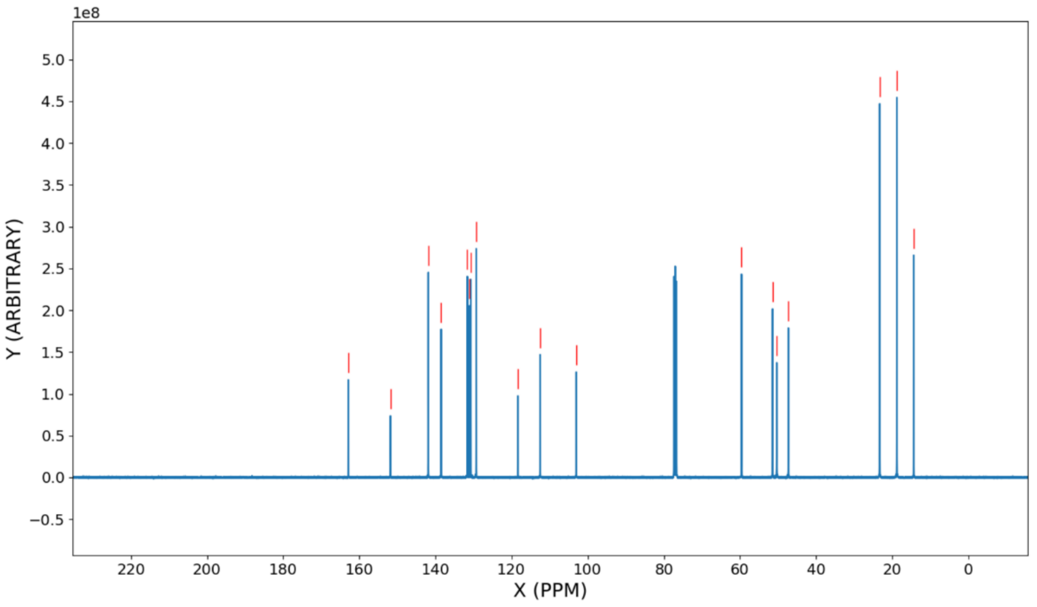

[**17k**] ethyl (*E*)-1-(4-bromobenzyl)-5-(3,3-diisopropyltriaz-1-en-1-yl)-1*H*-pyrazole-4-carboxylate

CHMO:0000593 | ^{1}H nuclear magnetic resonance spectroscopy (^{1}H NMR)

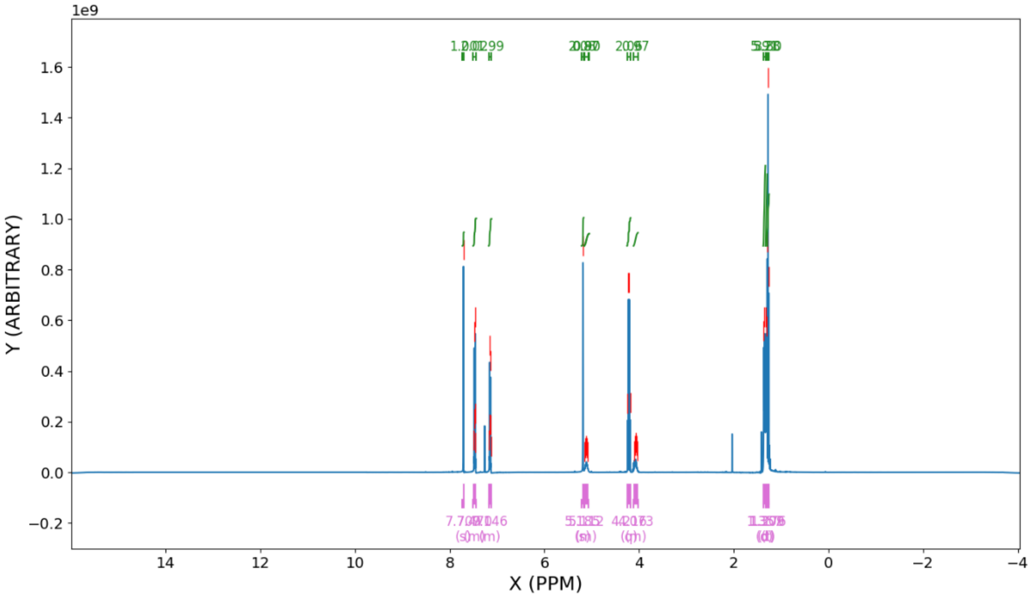

CHMO:0000595 | ^{13}C nuclear magnetic resonance spectroscopy (^{13}C NMR)

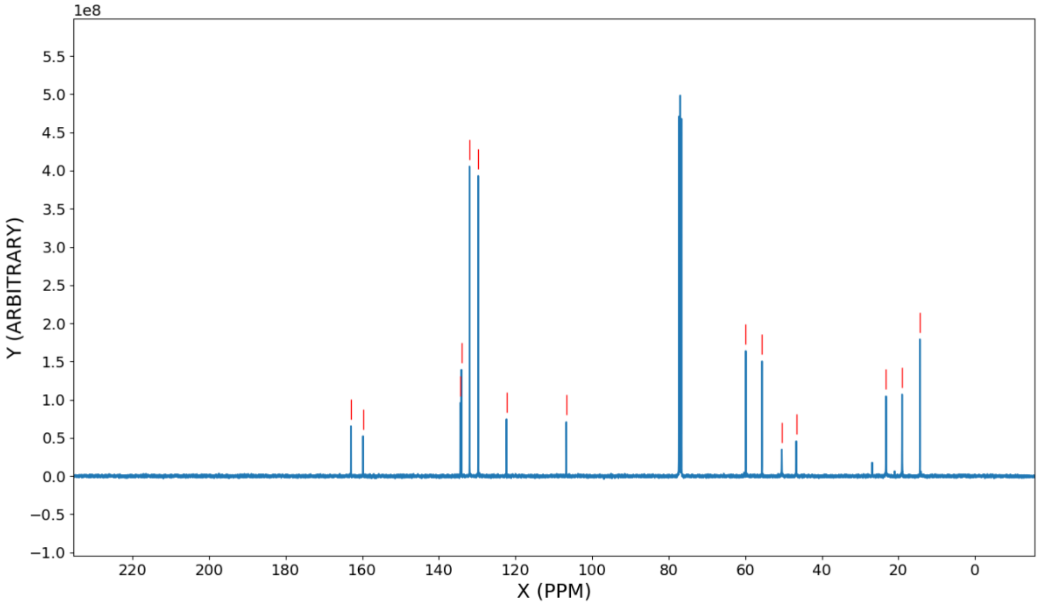

[**18k**] ethyl (*E*)-1-(4-bromobenzyl)-5-(3,3-diisopropyltriaz-1-en-1-yl)-1*H*-pyrazole-4-carboxylate

CHMO:0000593 | ^{1}H nuclear magnetic resonance spectroscopy (^{1}H NMR)

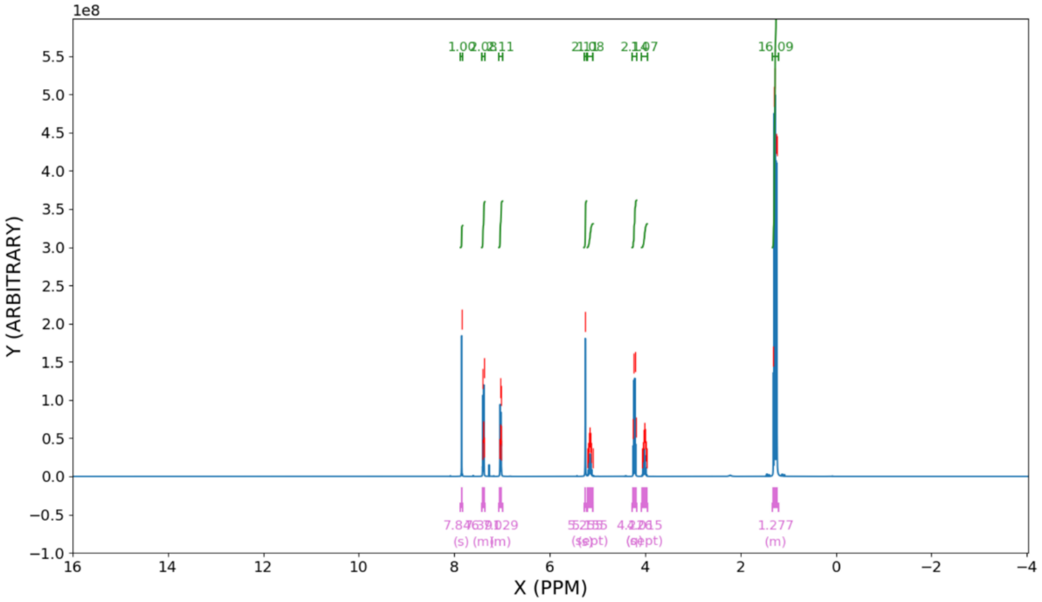

CHMO:0000595 | ^{13}C nuclear magnetic resonance spectroscopy (^{13}C NMR)

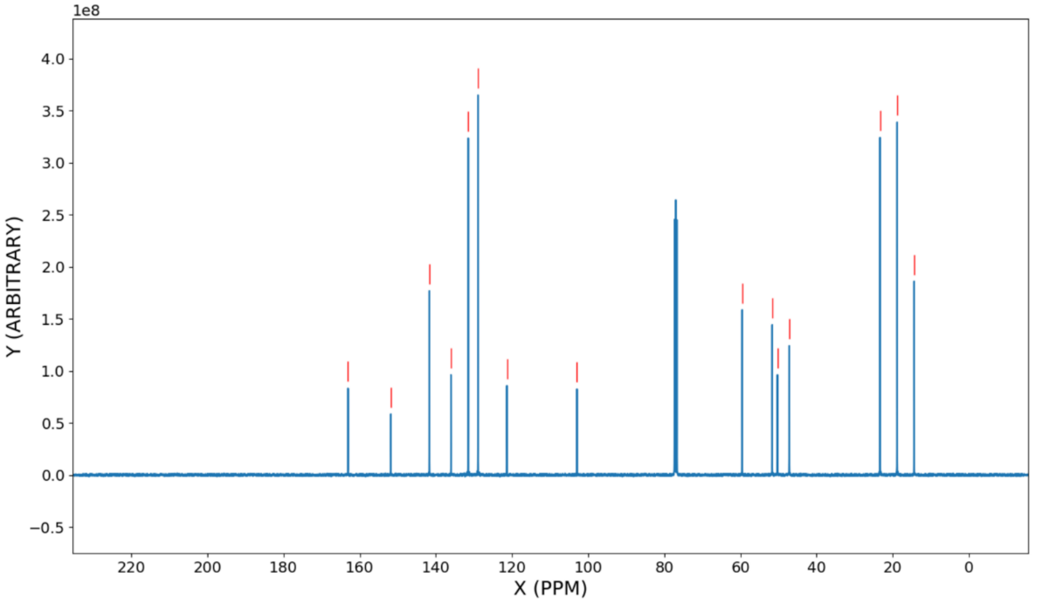

[**17l**] (*E*)-3-(3,3-diisopropyltriaz-1-en-1-yl)-1-isopropyl-1*H*-pyrazole-4-carbonitrile

CHMO:0000593 | ^{1}H nuclear magnetic resonance spectroscopy (^{1}H NMR)

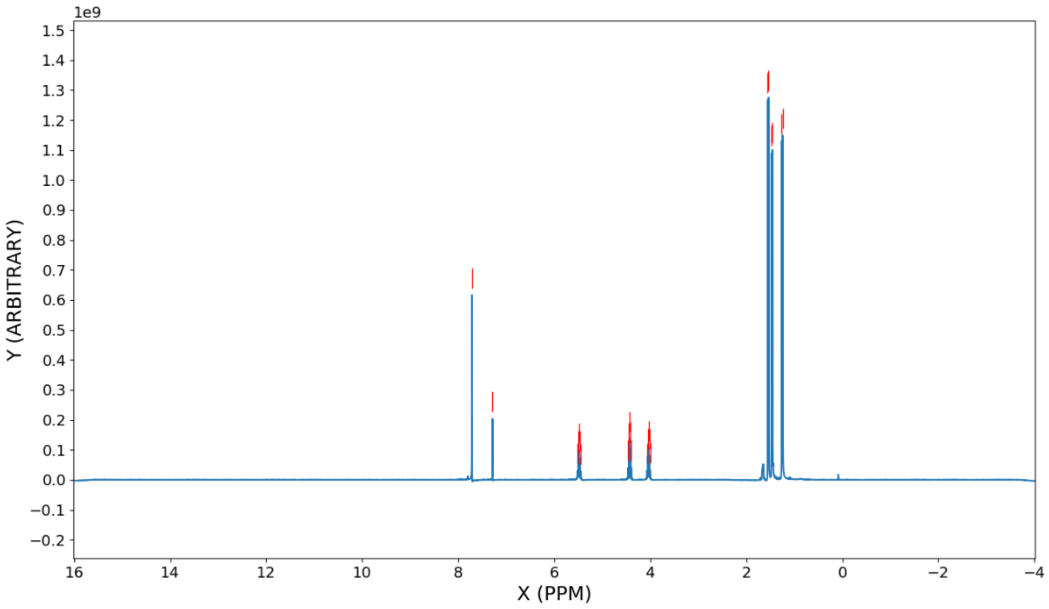

CHMO:0000595 | ^{13}C nuclear magnetic resonance spectroscopy (^{13}C NMR)

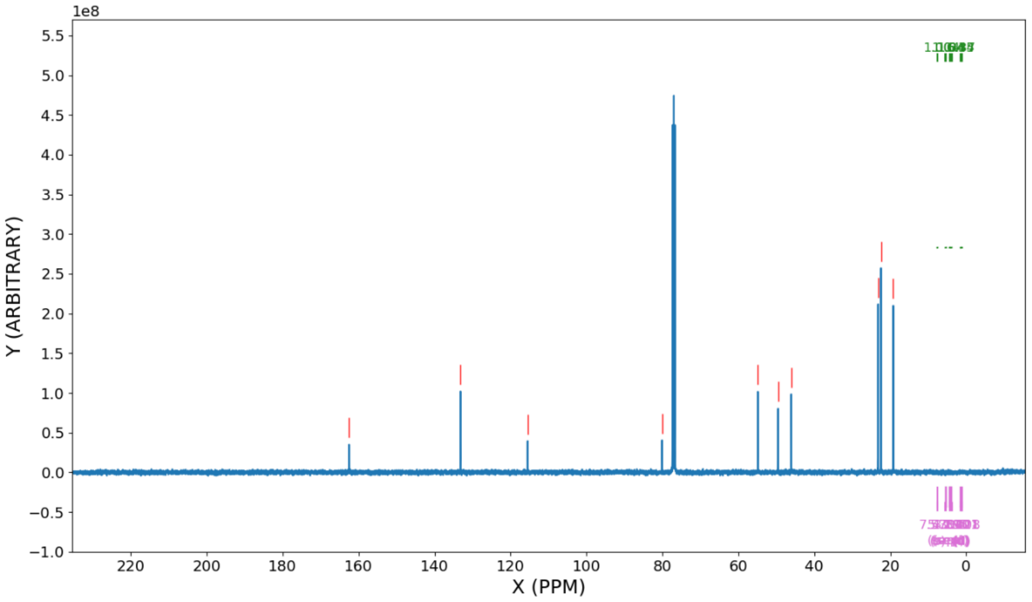

[**18l**] (*E*)-5-(3,3-diisopropyltriaz-1-en-1-yl)-1-isopropyl-1*H*-pyrazole-4-carbonitrile

CHMO:0000593 | ^{1}H nuclear magnetic resonance spectroscopy (^{1}H NMR)

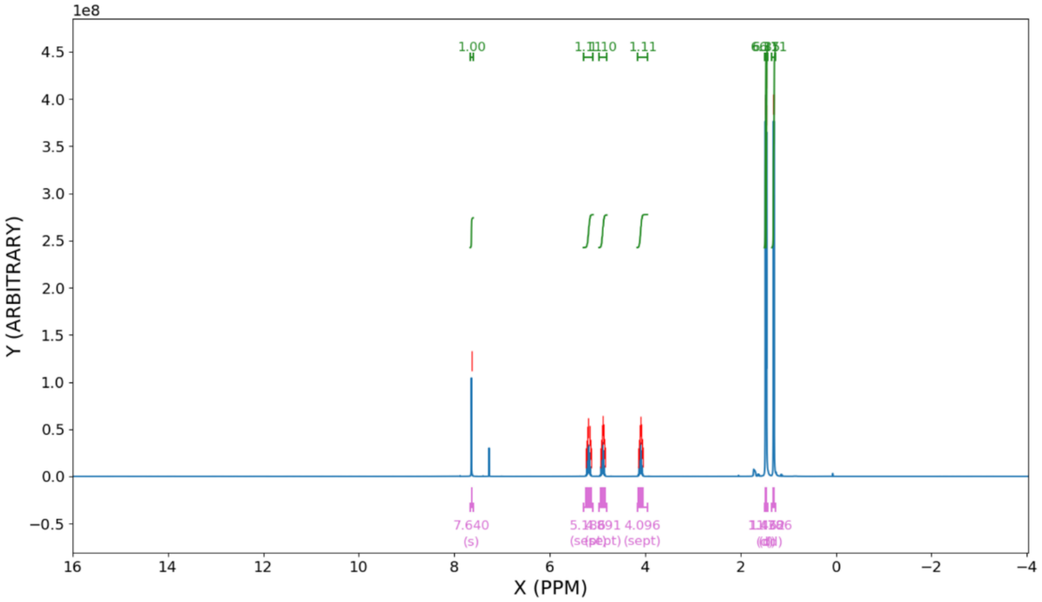

CHMO:0000595 | ^{13}C nuclear magnetic resonance spectroscopy (^{13}C NMR)

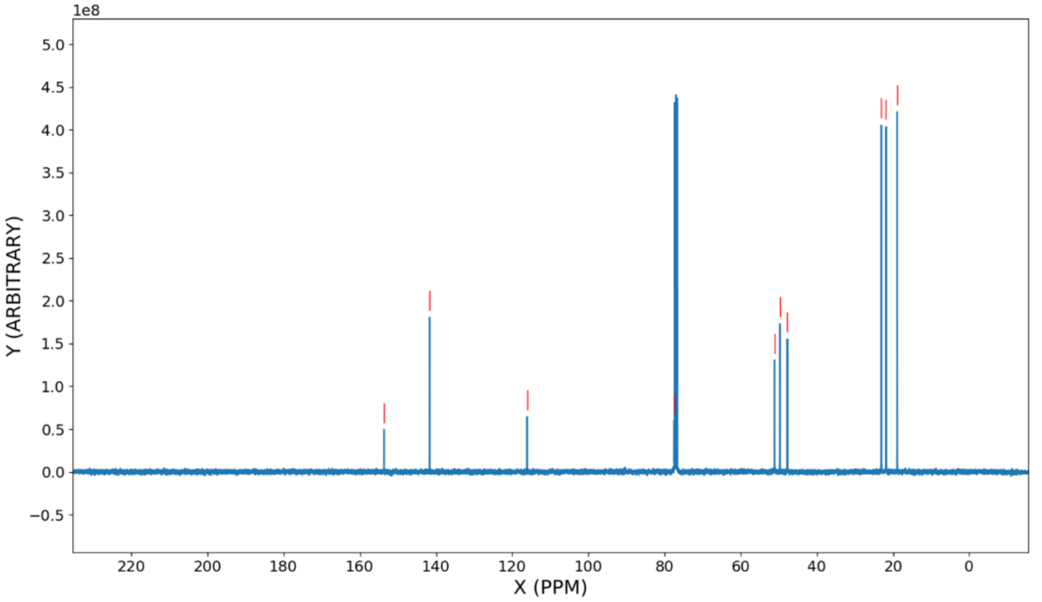

[**17m**] ethyl (*E*)-2-(4-cyano-3-(3,3-diisopropyltriaz-1-en-1-yl)-1*H*-pyrazol-1-yl)acetate

CHMO:0000593 | ^{1}H nuclear magnetic resonance spectroscopy (^{1}H NMR)

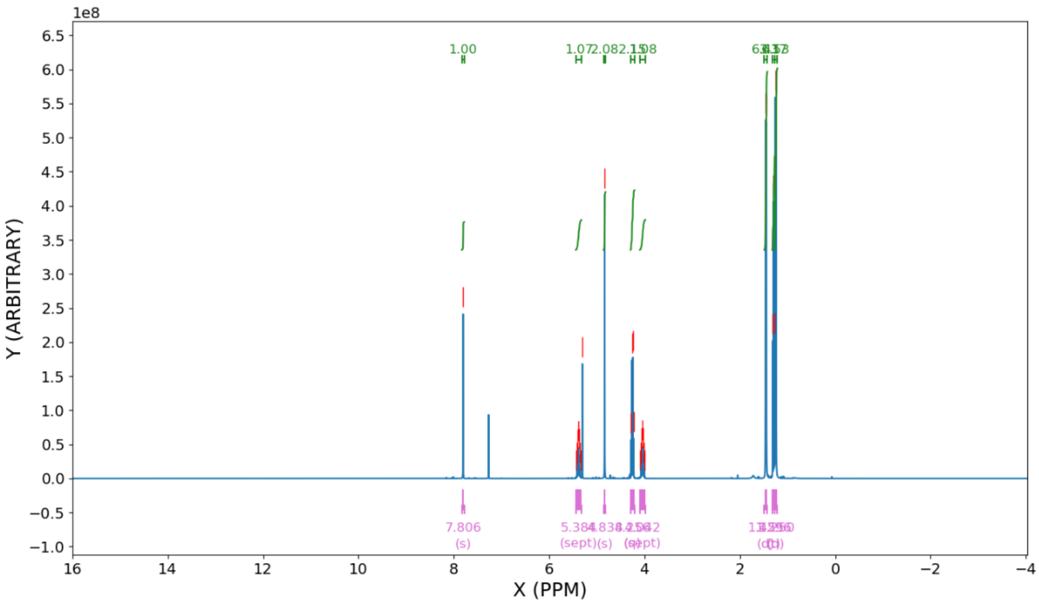

CHMO:0000595 | ^{13}C nuclear magnetic resonance spectroscopy (^{13}C NMR)

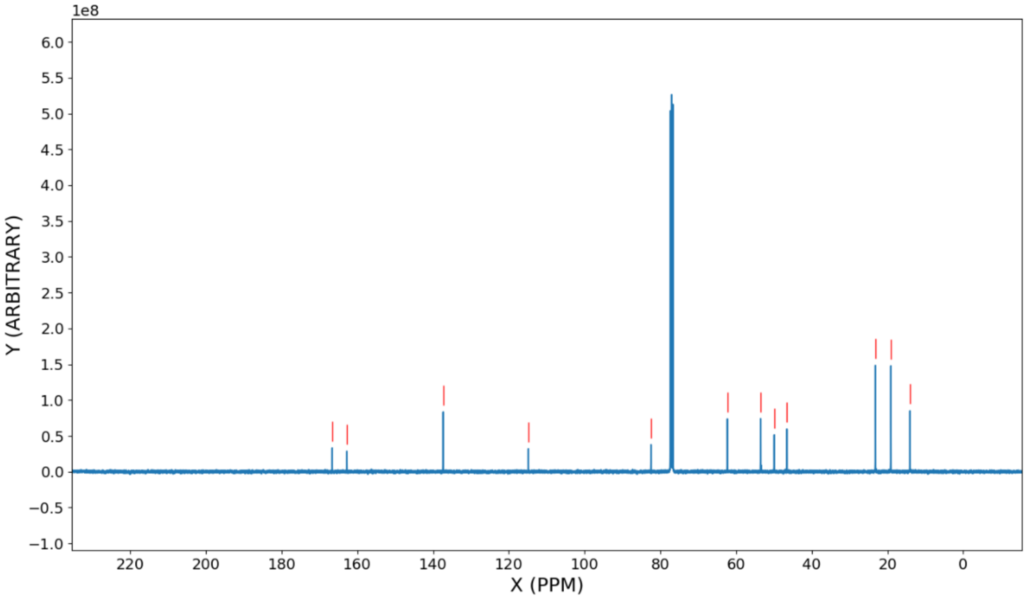

[**18m**] ethyl (*E*)-2-(4-cyano-5-(3,3-diisopropyltriaz-1-en-1-yl)-1*H*-pyrazol-1-yl)acetate

CHMO:0000593 | ^{1}H nuclear magnetic resonance spectroscopy (^{1}H NMR)

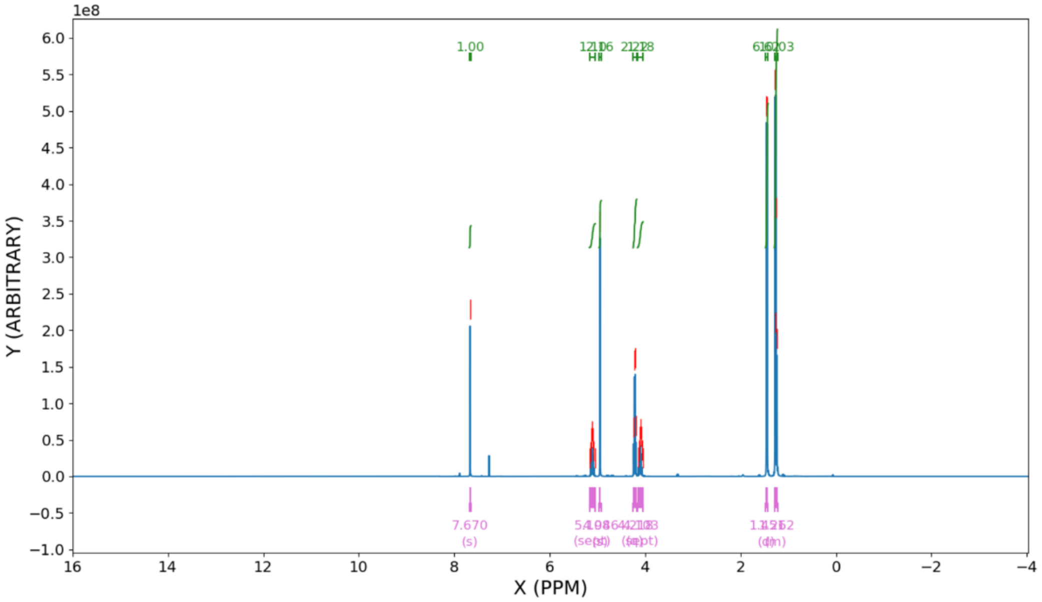

CHMO:0000595 | ^{13}C nuclear magnetic resonance spectroscopy (^{13}C NMR)

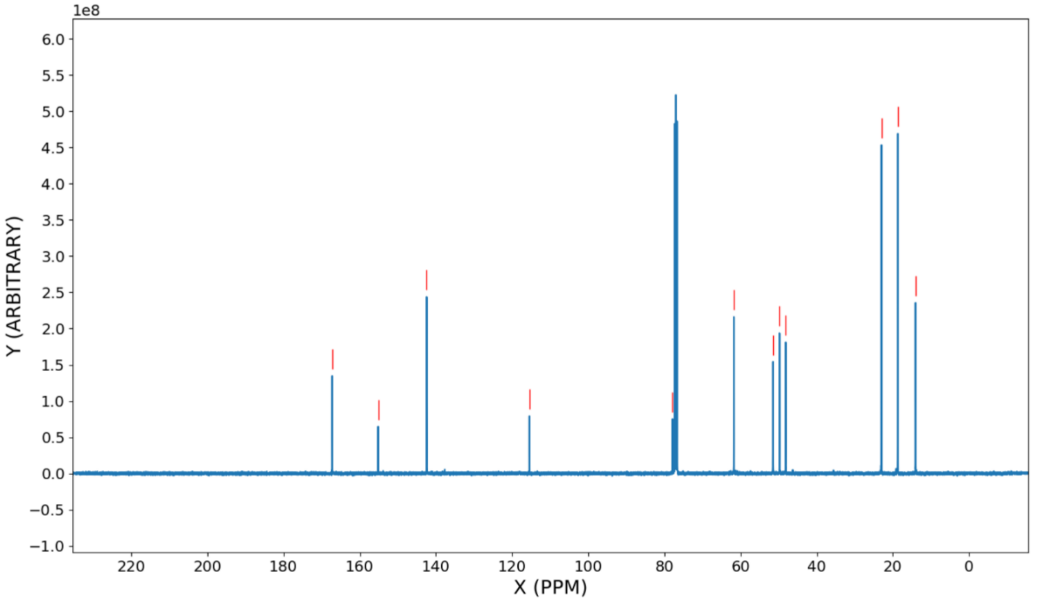

[**17n**] (*E*)-1-benzyl-3-(3,3-diisopropyltriaz-1-en-1-yl)-1*H*-pyrazole-4-carbonitrile

CHMO:0000593 | ^{1}H nuclear magnetic resonance spectroscopy (^{1}H NMR)

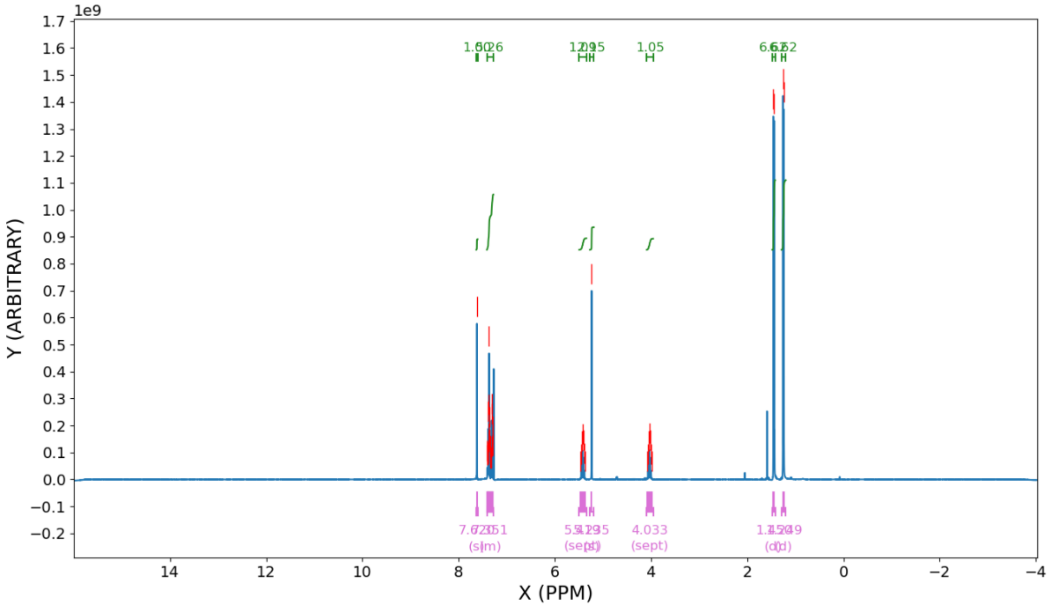

CHMO:0000595 | ^{13}C nuclear magnetic resonance spectroscopy (^{13}C NMR)

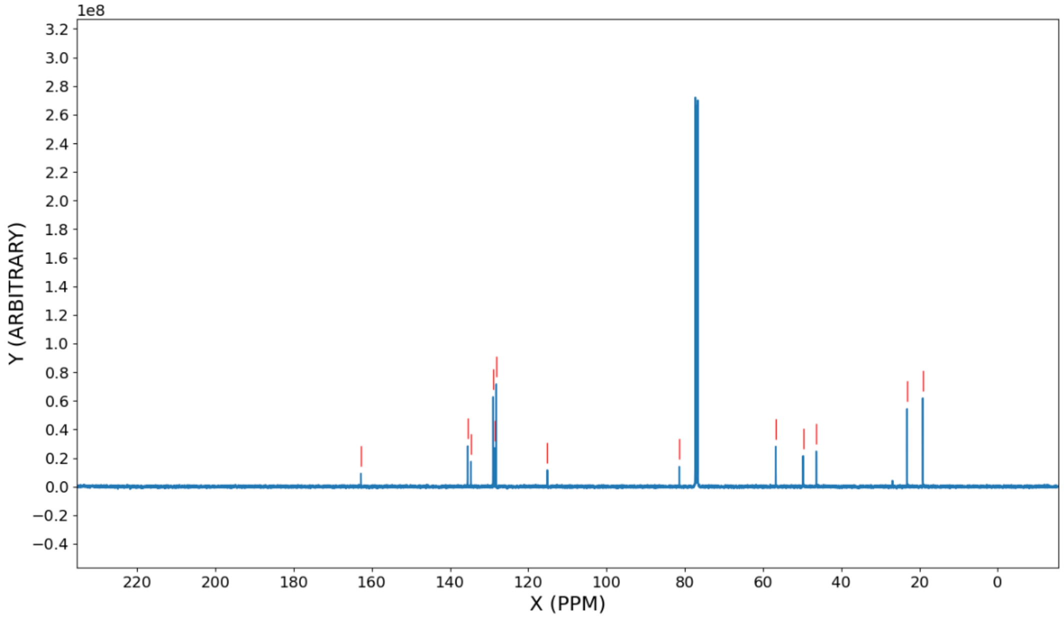

[**18n**] (*E*)-1-benzyl-5-(3,3-diisopropyltriaz-1-en-1-yl)-1*H*-pyrazole-4-carbonitrile

CHMO:0000593 | ^{1}H nuclear magnetic resonance spectroscopy (^{1}H NMR)

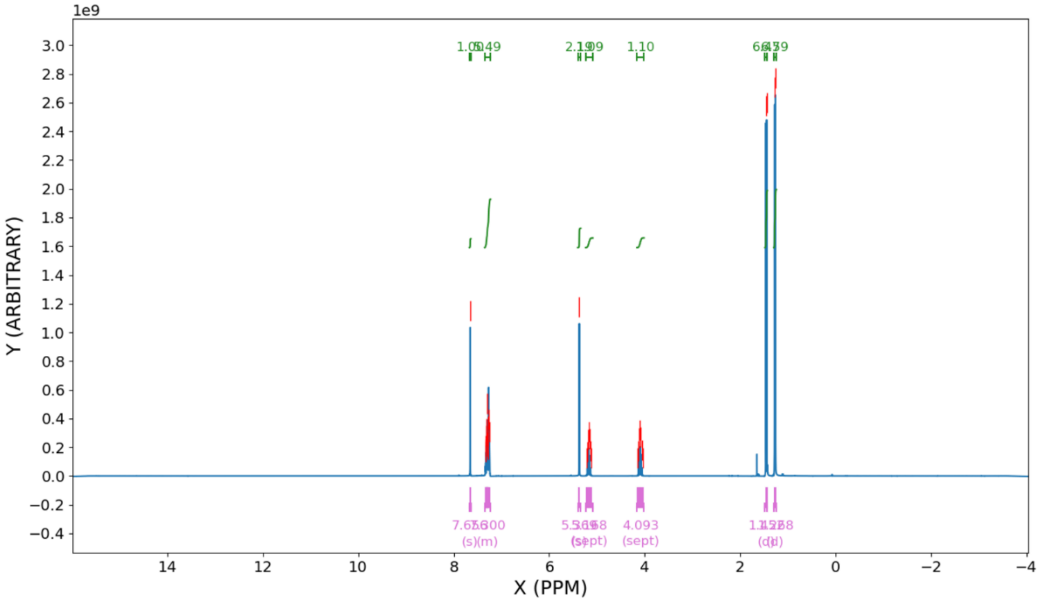

CHMO:0000595 | ^{13}C nuclear magnetic resonance spectroscopy (^{13}C NMR)

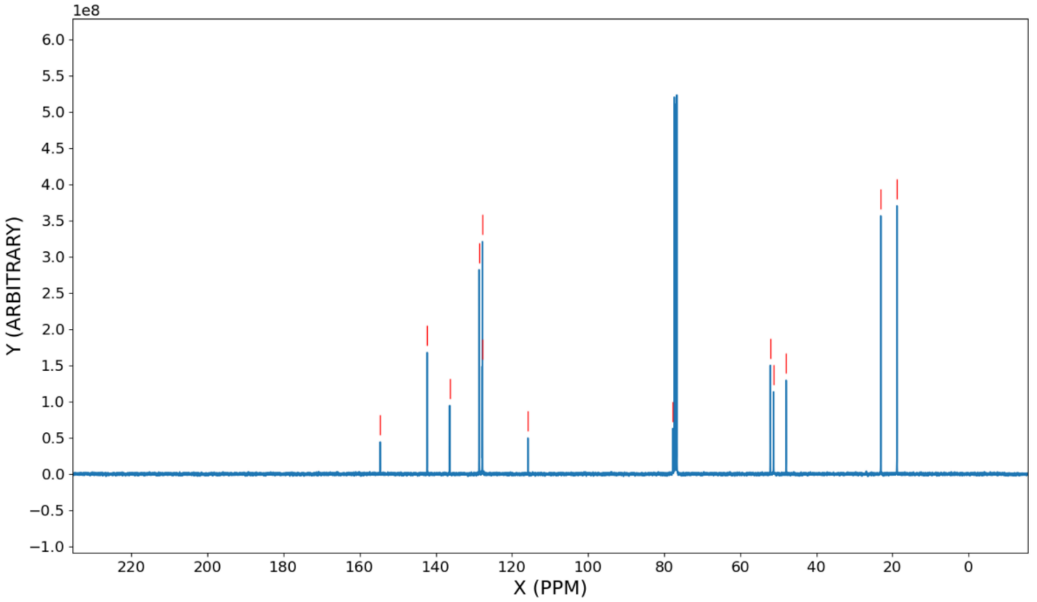

[**17o**] (*E*)-1-(3,5-difluorobenzyl)-3-(3,3-diisopropyltriaz-1-en-1-yl)-1*H*-pyrazole-4-carbonitrile

CHMO:0000593 | ^{1}H nuclear magnetic resonance spectroscopy (^{1}H NMR)

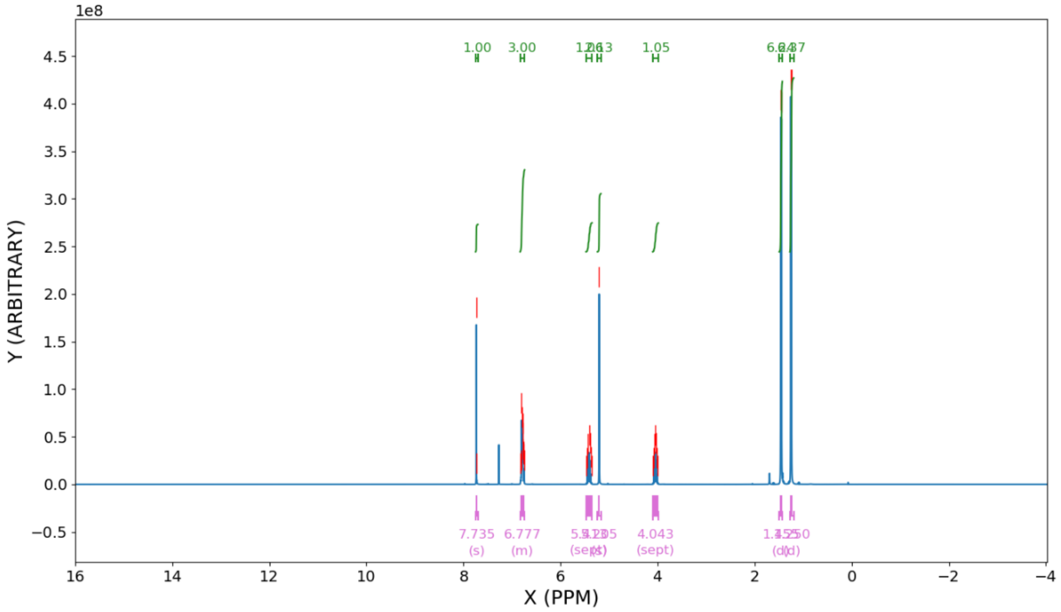

CHMO:0000595 | ^{13}C nuclear magnetic resonance spectroscopy (^{13}C NMR)

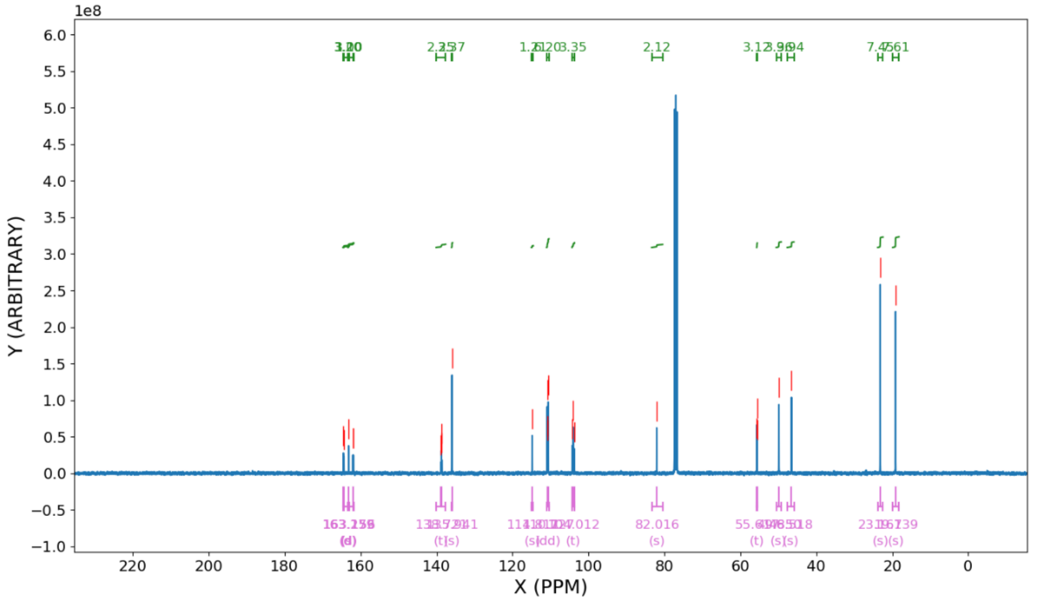

[**18o**] (*E*)-1-(3,5-difluorobenzyl)-5-(3,3-diisopropyltriaz-1-en-1-yl)-1*H*-pyrazole-4-carbonitrile

CHMO:0000593 | ^{1}H nuclear magnetic resonance spectroscopy (^{1}H NMR)

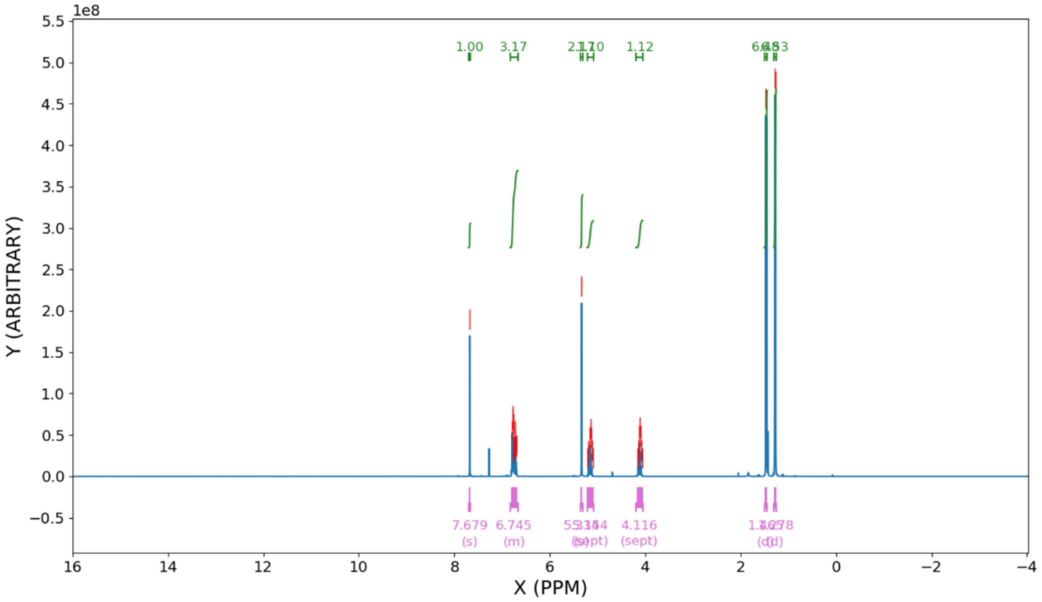

CHMO:0000595 | ^{13}C nuclear magnetic resonance spectroscopy (^{13}C NMR)

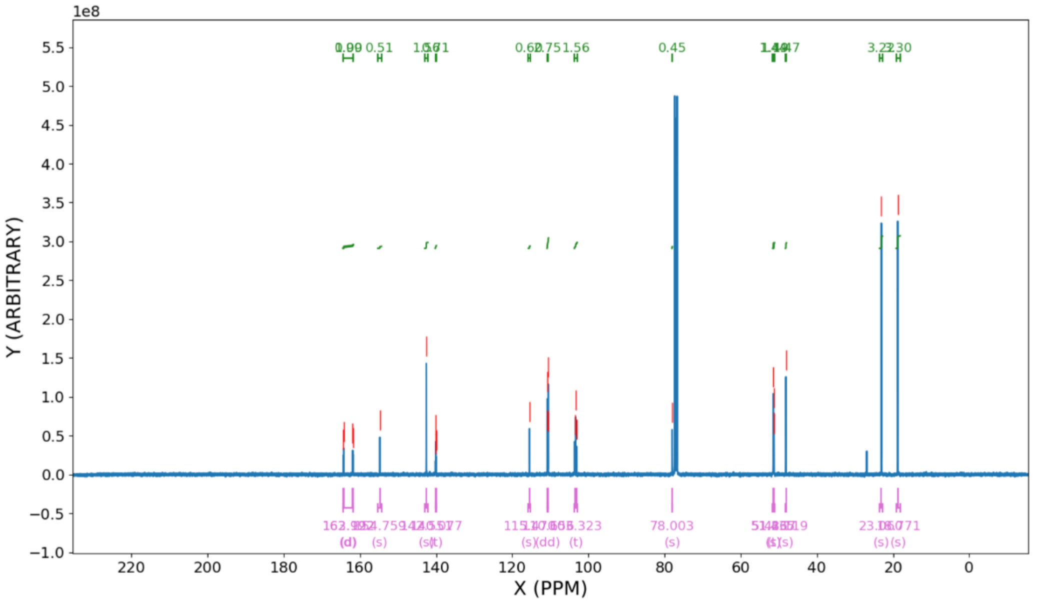

[**17p**] (*E*)-3-(3,3-diisopropyltriaz-1-en-1-yl)-1-(4-methylbenzyl)-1*H*-pyrazole-4-carbonitrile

CHMO:0000593 | ^{1}H nuclear magnetic resonance spectroscopy (^{1}H NMR)

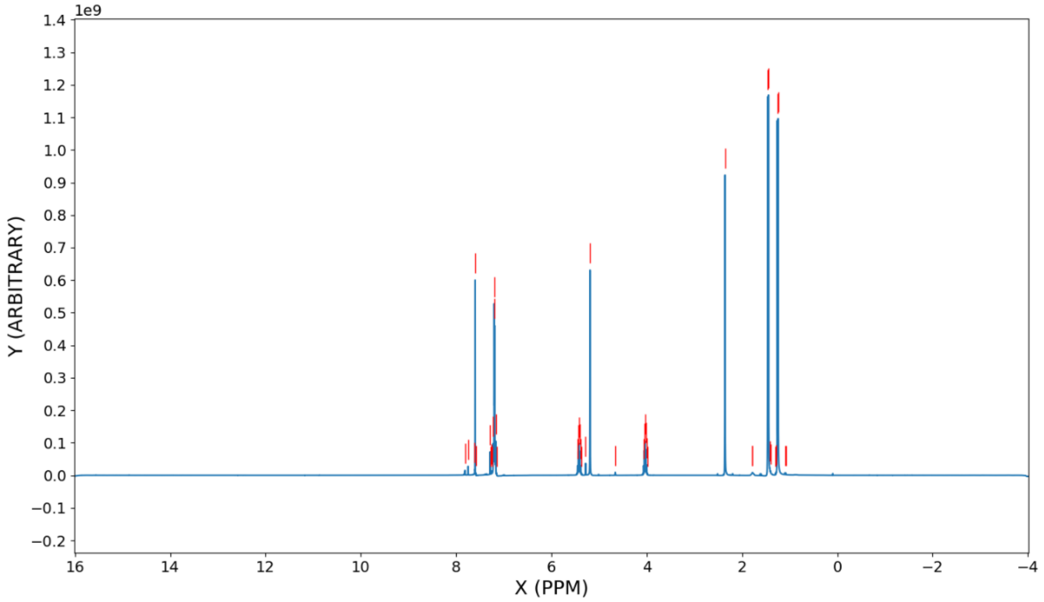

CHMO:0000595 | ^{13}C nuclear magnetic resonance spectroscopy (^{13}C NMR)

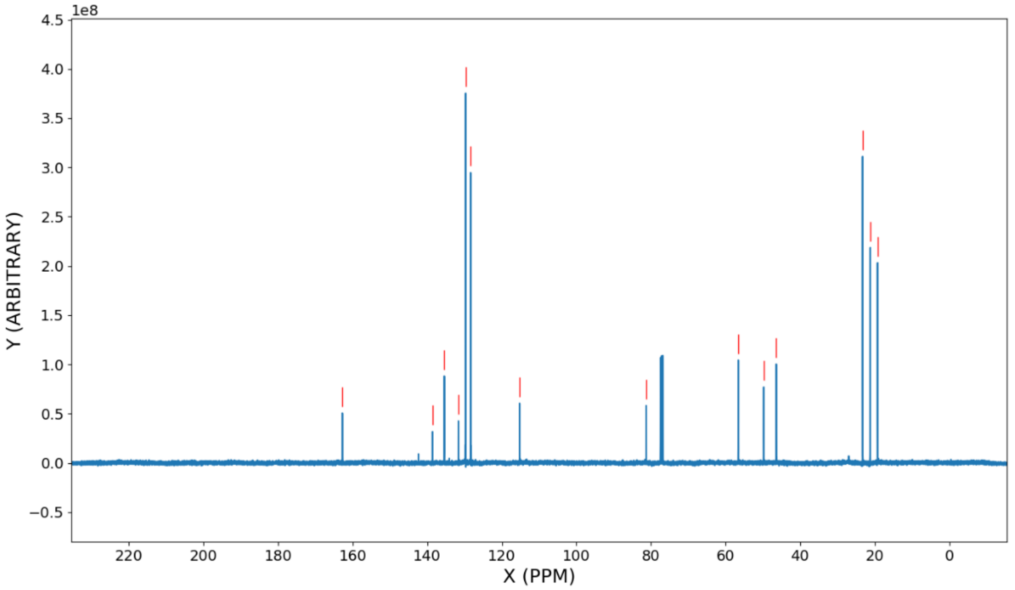

[**18p**] (*E*)-5-(3,3-diisopropyltriaz-1-en-1-yl)-1-(4-methylbenzyl)-1*H*-pyrazole-4-carbonitrile

CHMO:0000593 | ^{1}H nuclear magnetic resonance spectroscopy (^{1}H NMR)

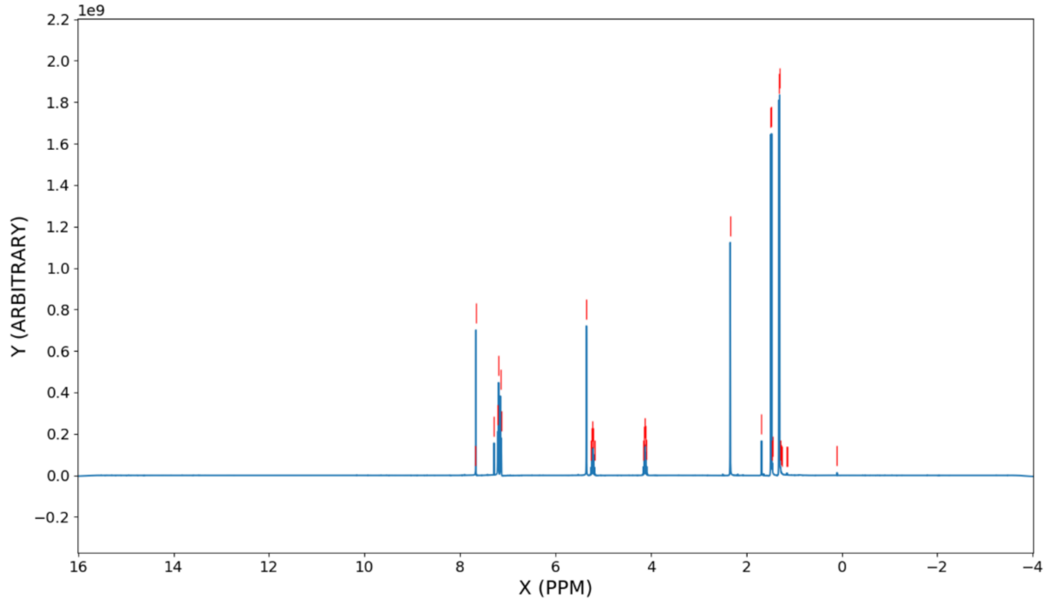

CHMO:0000595 | ^{13}C nuclear magnetic resonance spectroscopy (^{13}C NMR)

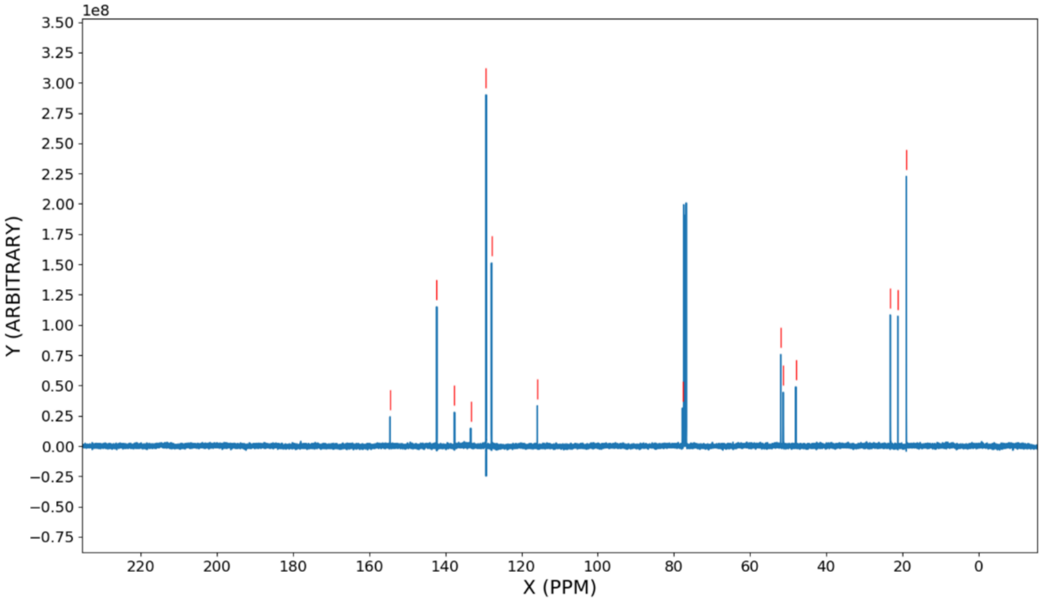

[**17q**] methyl (*E*)-4-((4-cyano-3-(3,3-diisopropyltriaz-1-en-1-yl)-1*H*-pyrazol-1-yl)methyl)benzoate

CHMO:0000593 | ^{1}H nuclear magnetic resonance spectroscopy (^{1}H NMR)

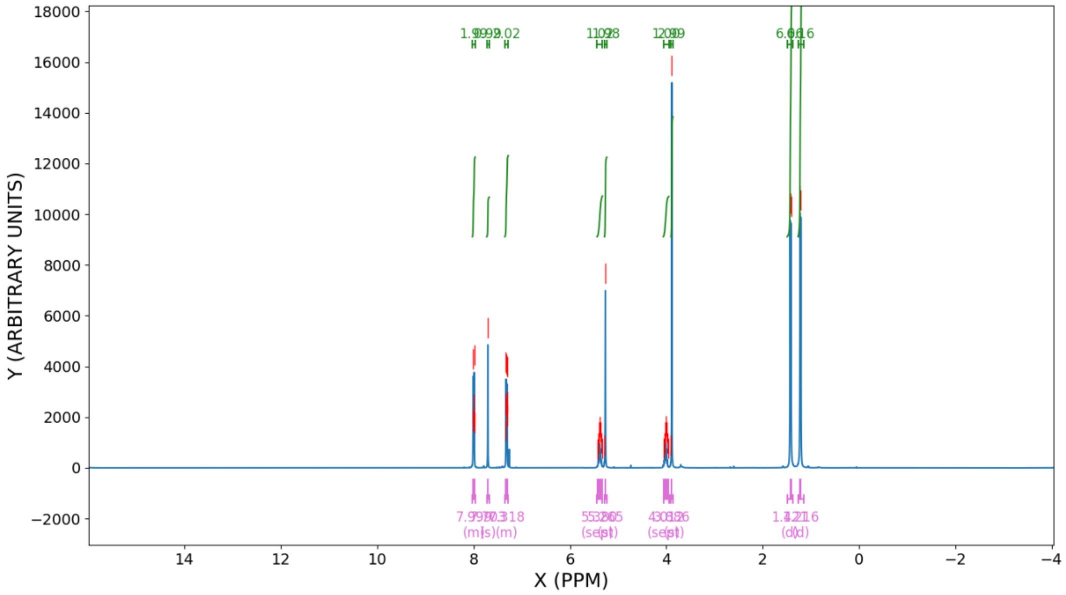

CHMO:0000595 | ^{13}C nuclear magnetic resonance spectroscopy (^{13}C NMR)

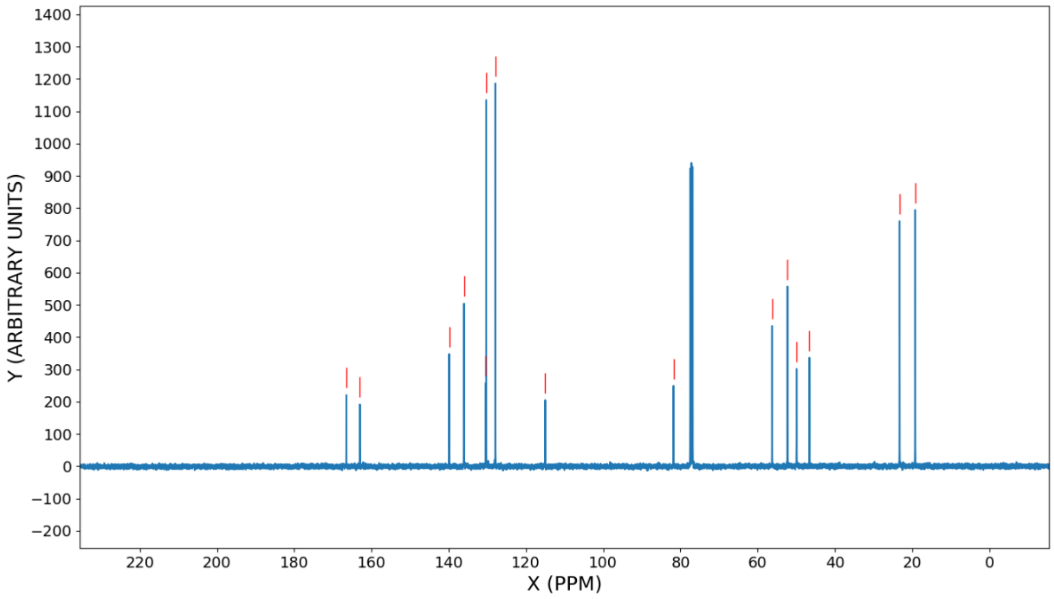

[**18q**] methyl (*E*)-4-((4-cyano-5-(3,3-diisopropyltriaz-1-en-1-yl)-1*H*-pyrazol-1-yl)methyl)benzoate

CHMO:0000593 | ^{1}H nuclear magnetic resonance spectroscopy (^{1}H NMR)

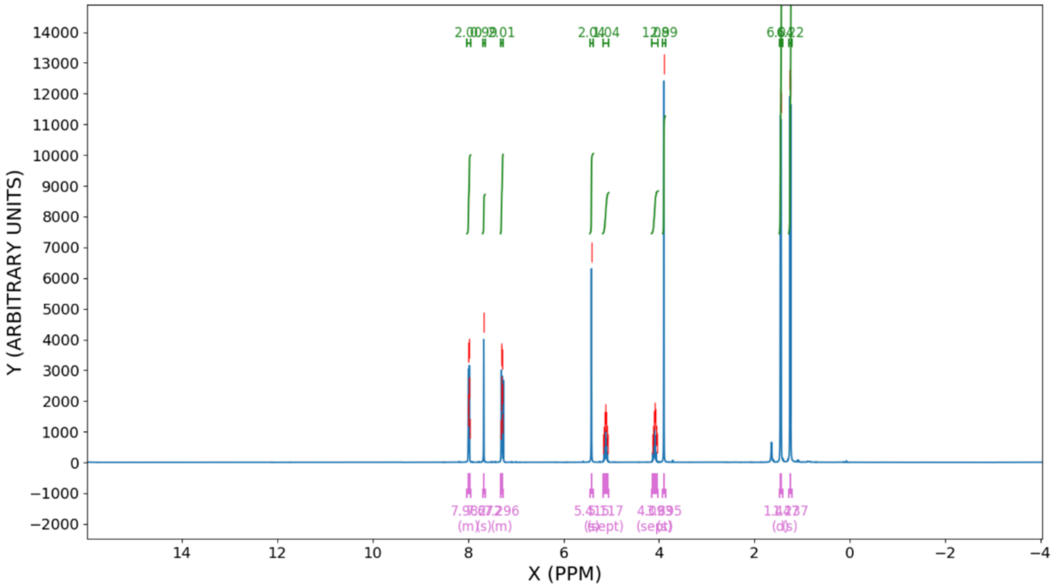

CHMO:0000595 | ^{13}C nuclear magnetic resonance spectroscopy (^{13}C NMR)

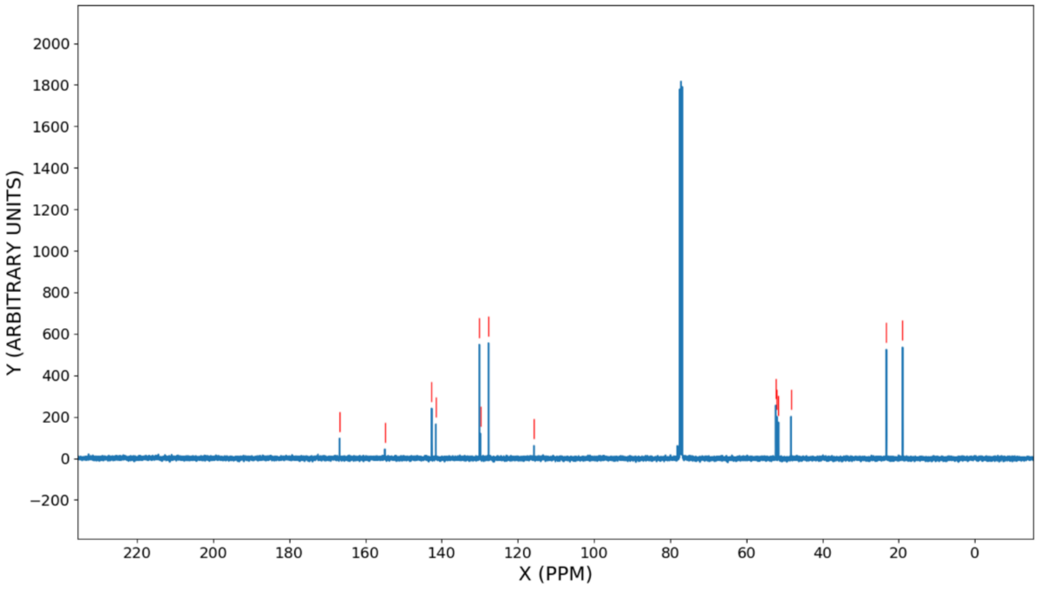

[**17r**] (*E*)-3-(3,3-diisopropyltriaz-1-en-1-yl)-1-(4-fluorobenzyl)-1*H*-pyrazole-4-carbonitrile

CHMO:0000593 | ^{1}H nuclear magnetic resonance spectroscopy (^{1}H NMR)

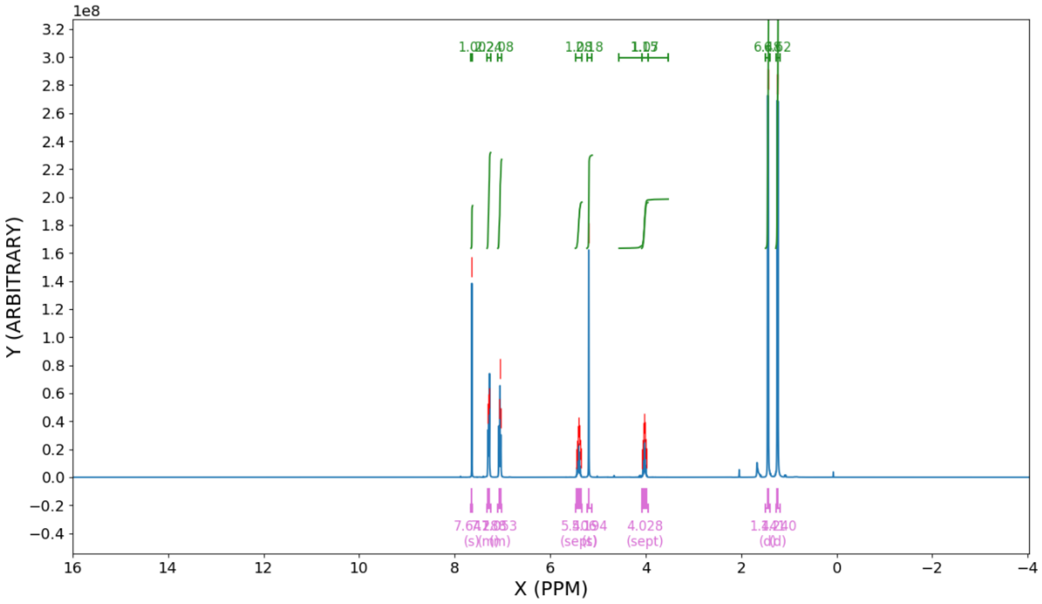

CHMO:0000595 | ^{13}C nuclear magnetic resonance spectroscopy (^{13}C NMR)

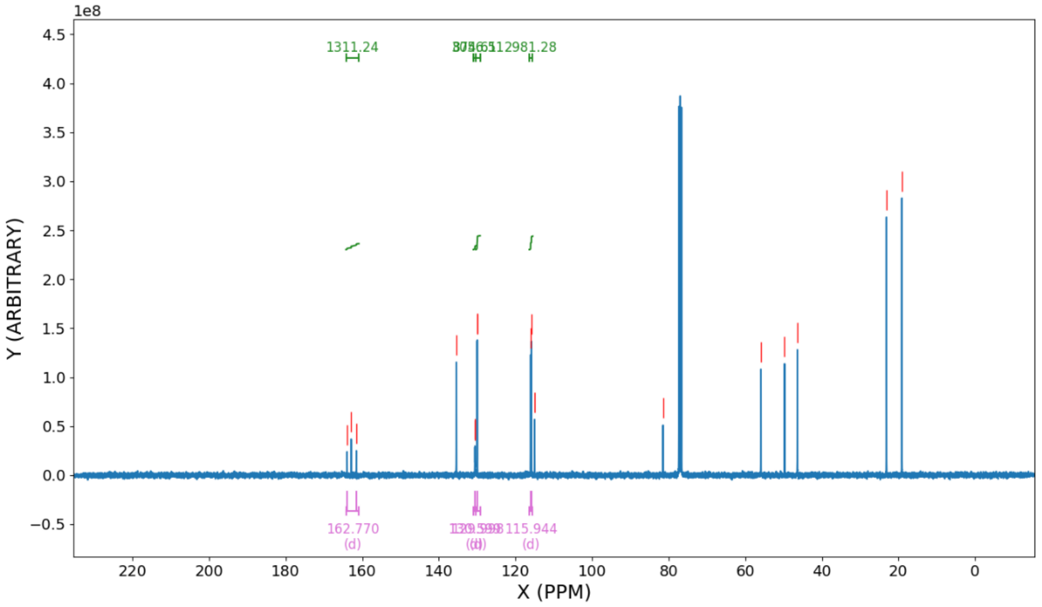

[**18r**] (*E*)-5-(3,3-diisopropyltriaz-1-en-1-yl)-1-(4-fluorobenzyl)-1*H*-pyrazole-4-carbonitrile

CHMO:0000593 | ^{1}H nuclear magnetic resonance spectroscopy (^{1}H NMR)

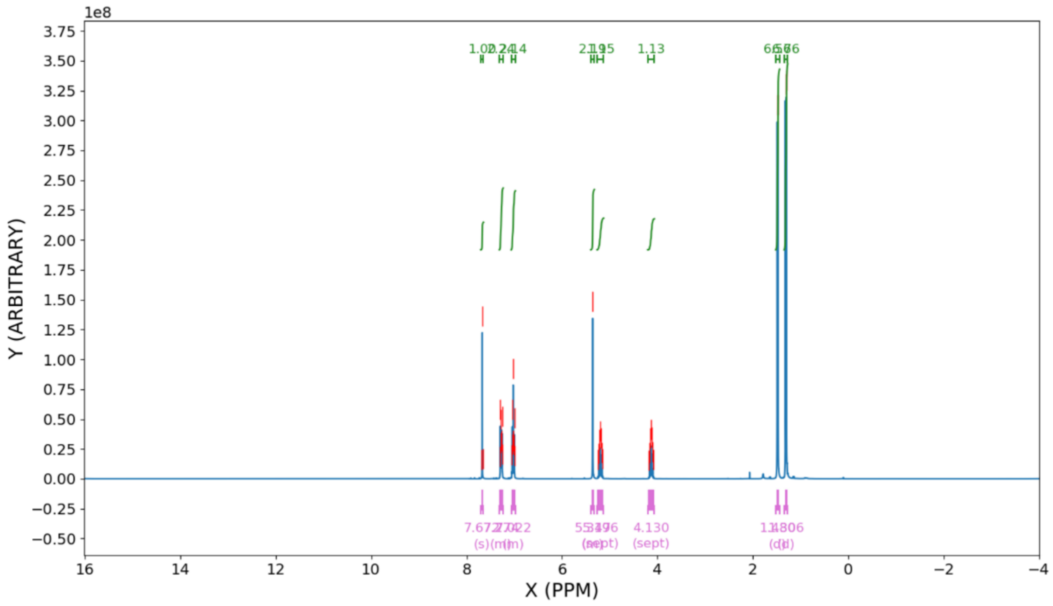

CHMO:0000595 | ^{13}C nuclear magnetic resonance spectroscopy (^{13}C NMR)

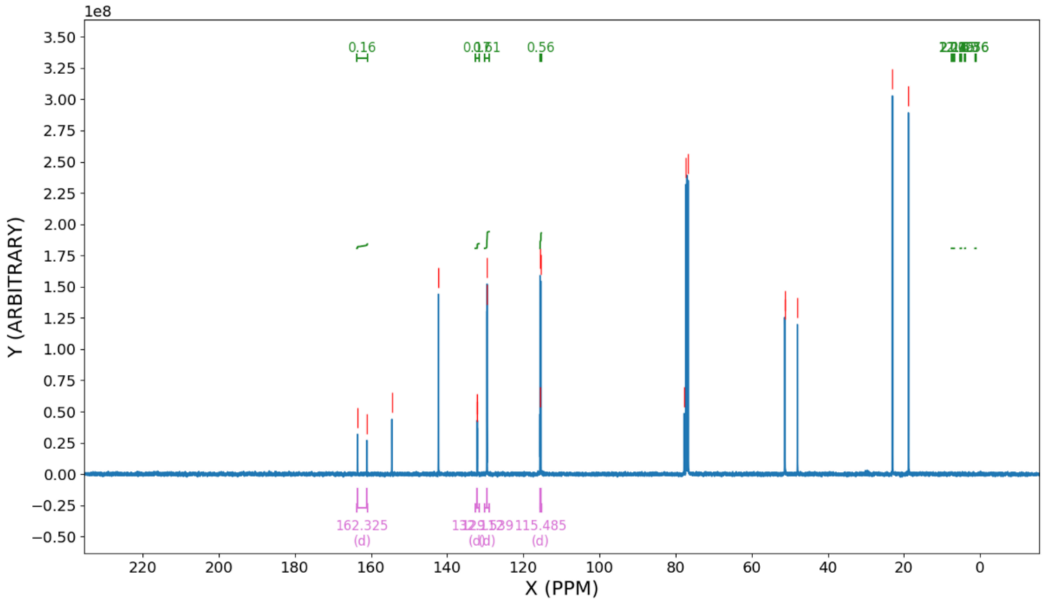

[**19a**] 5-azido-1*H*-pyrazole

CHMO:0000593 | ^{1}H nuclear magnetic resonance spectroscopy (^{1}H NMR)

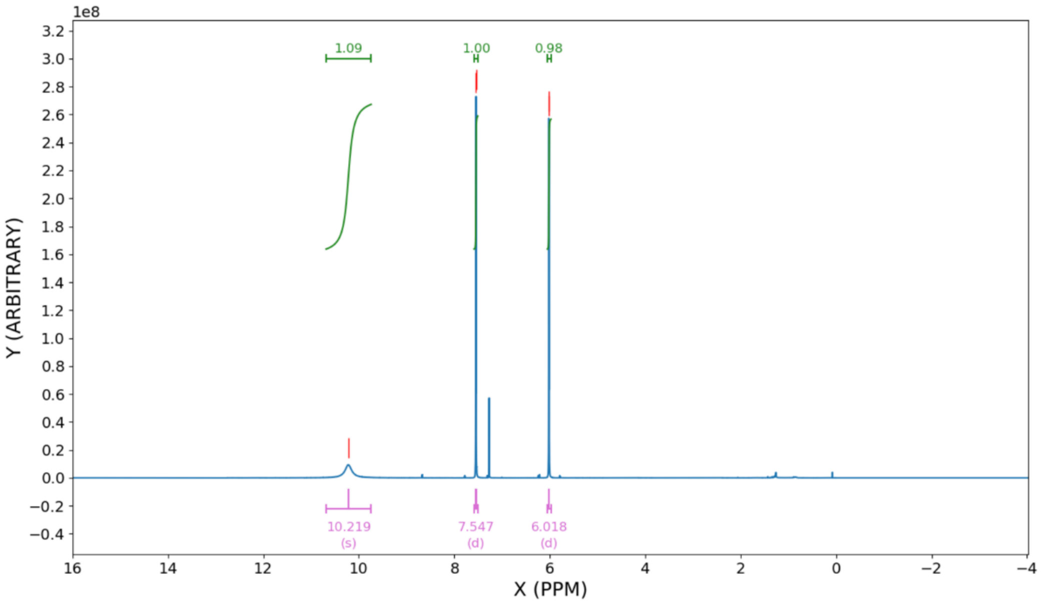

CHMO:0000595 | ^{13}C nuclear magnetic resonance spectroscopy (^{13}C NMR)

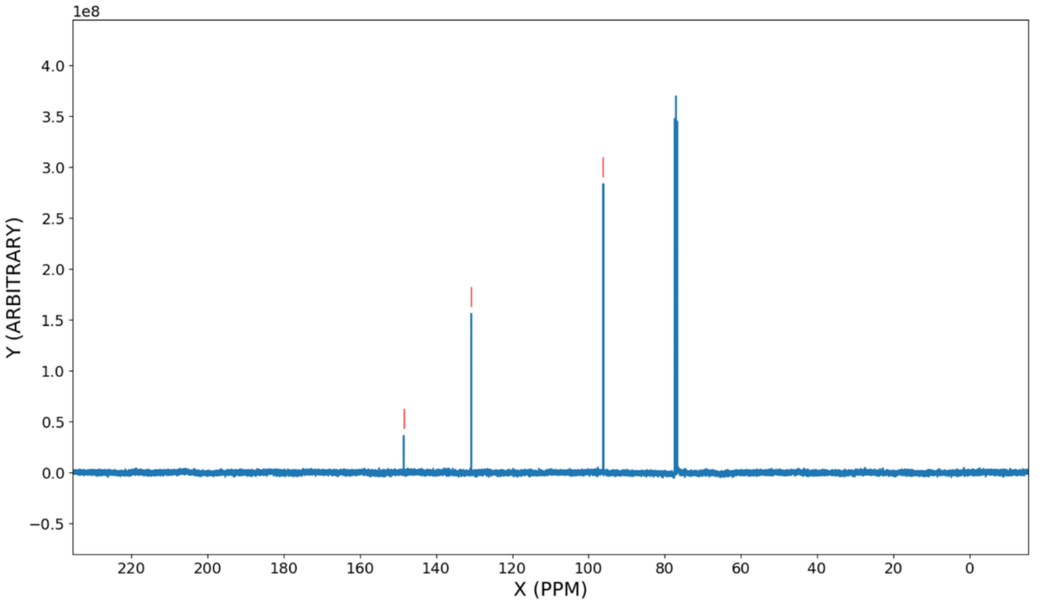

[**19b**] 3-azido-1-cyclopentylpyrazole

CHMO:0000593 | ^{1}H nuclear magnetic resonance spectroscopy (^{1}H NMR)

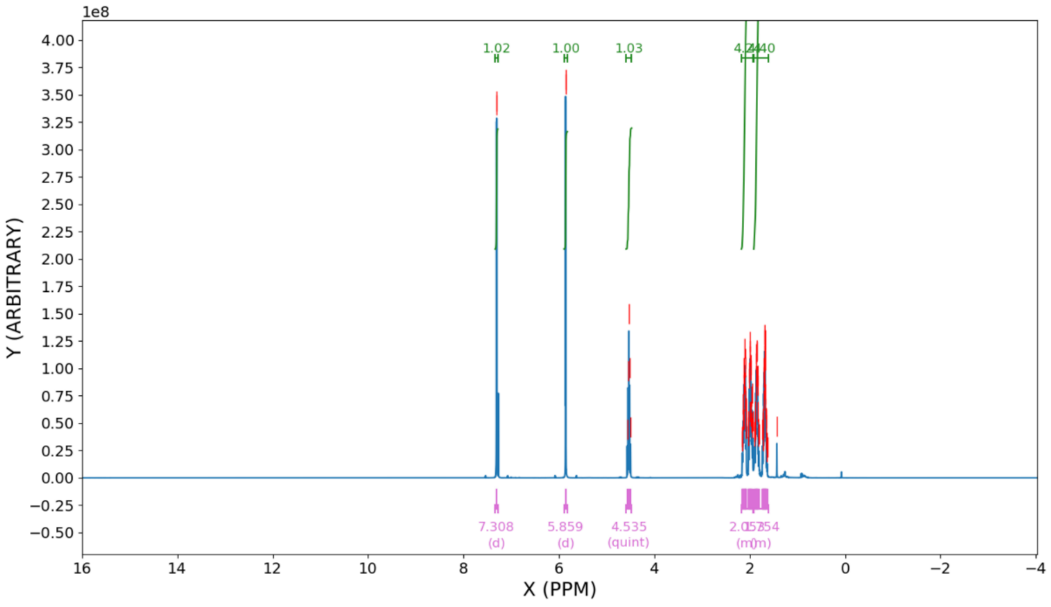

CHMO:0000595 | ^{13}C nuclear magnetic resonance spectroscopy (^{13}C NMR)

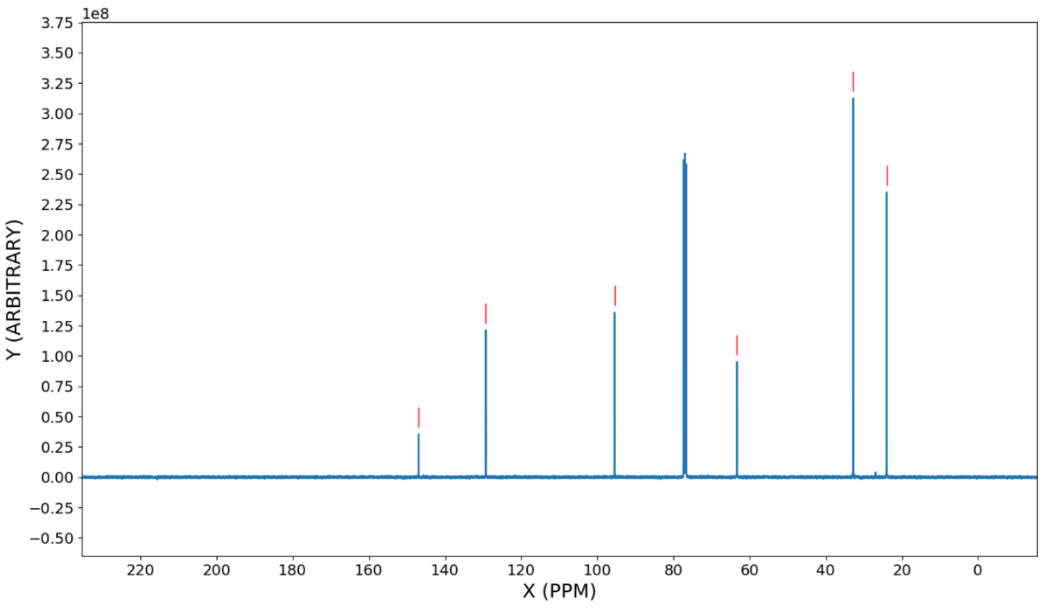

[**19c**] 3-azido-1-benzyl-pyrazole

CHMO:0000593 | ^{1}H nuclear magnetic resonance spectroscopy (^{1}H NMR)

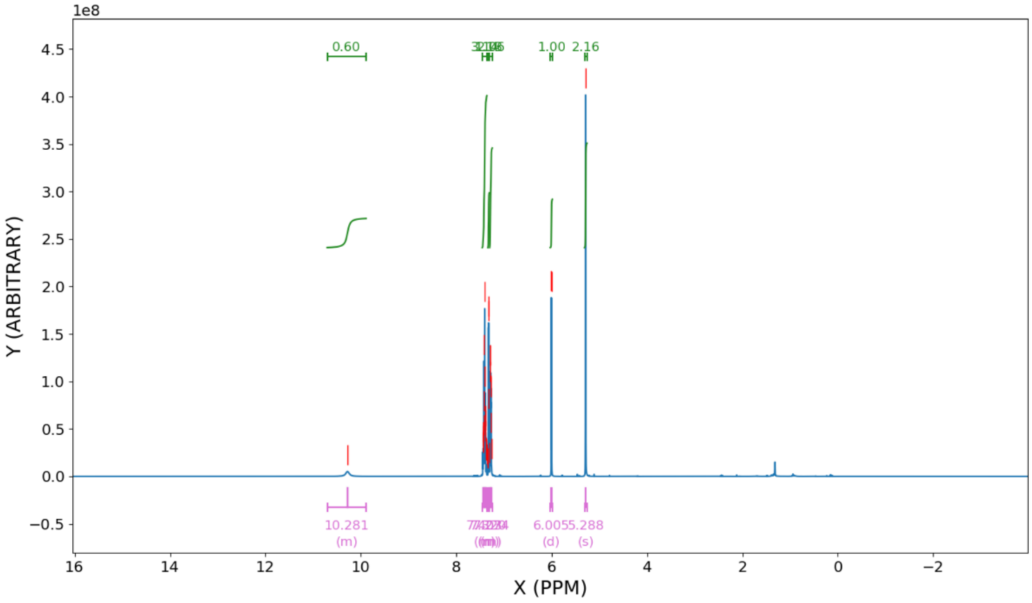

CHMO:0000595 | ^{13}C nuclear magnetic resonance spectroscopy (^{13}C NMR)

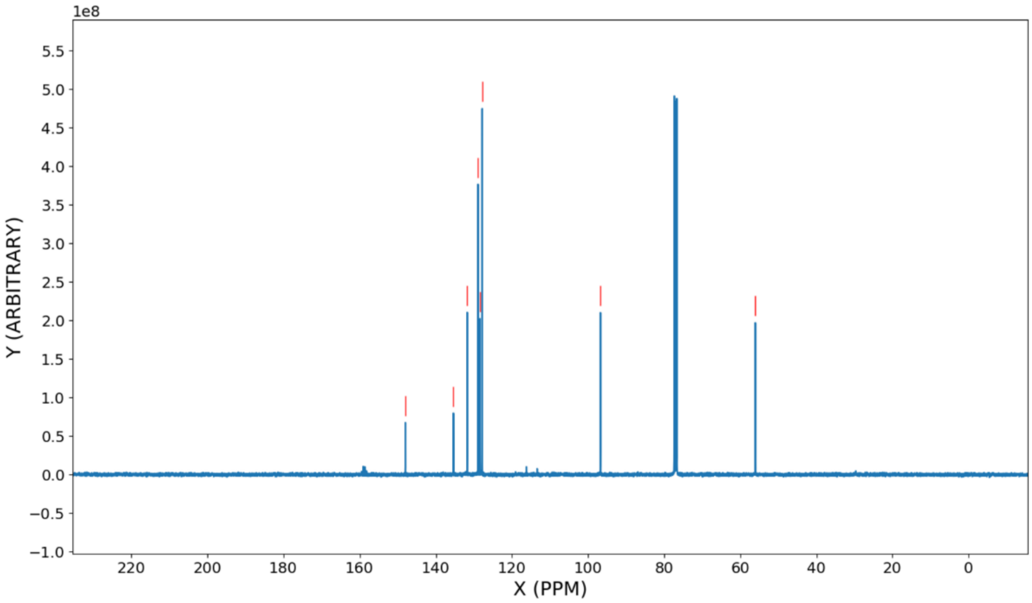

[**19d**] 3-azido-1-(4-bromobenzyl)pyrazole

CHMO:0000593 | ^{1}H nuclear magnetic resonance spectroscopy (^{1}H NMR)

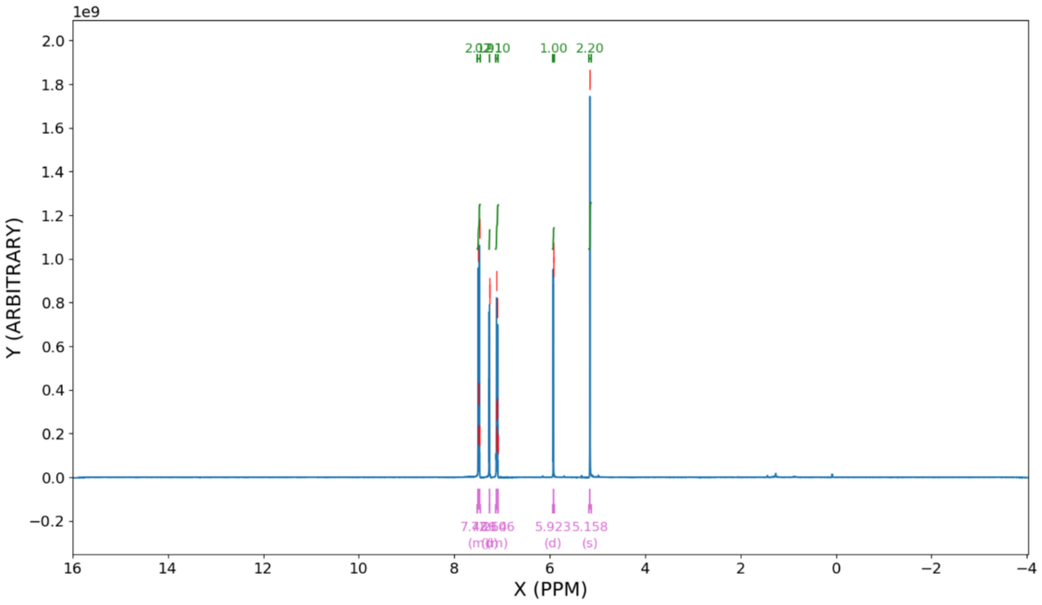

CHMO:0000595 | ^{13}C nuclear magnetic resonance spectroscopy (^{13}C NMR)

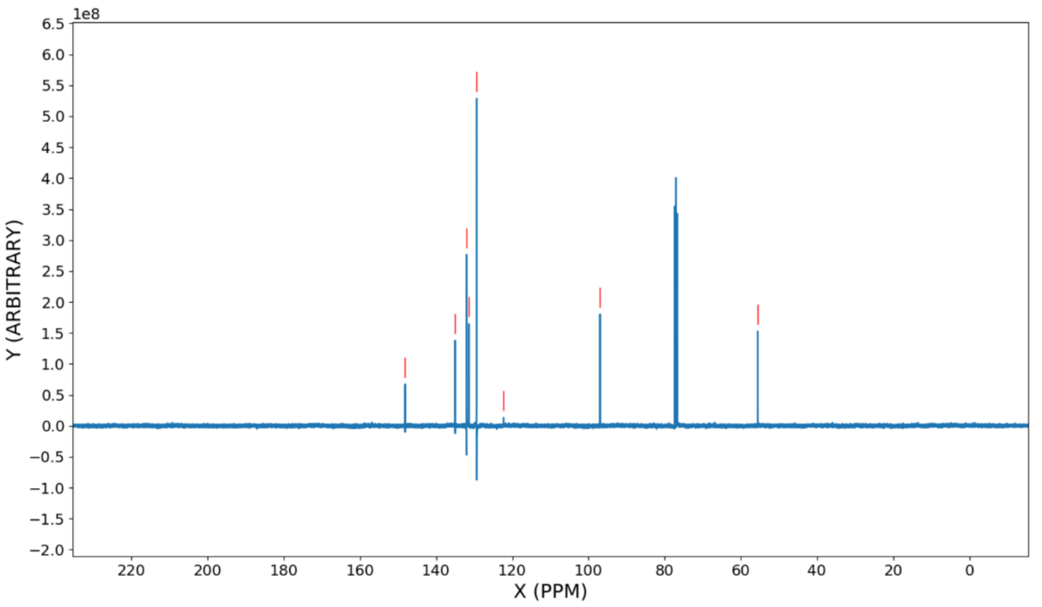

[**19e**] 3-azido-5-methyl-1*H*-pyrazole

CHMO:0000593 | ^{1}H nuclear magnetic resonance spectroscopy (^{1}H NMR)

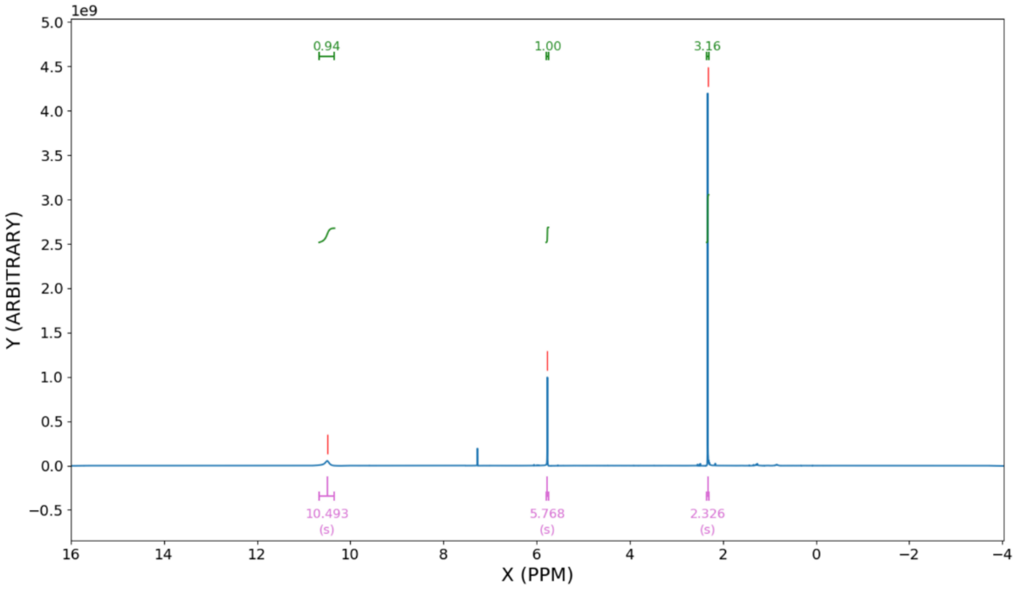

CHMO:0000595 | ^{13}C nuclear magnetic resonance spectroscopy (^{13}C NMR)

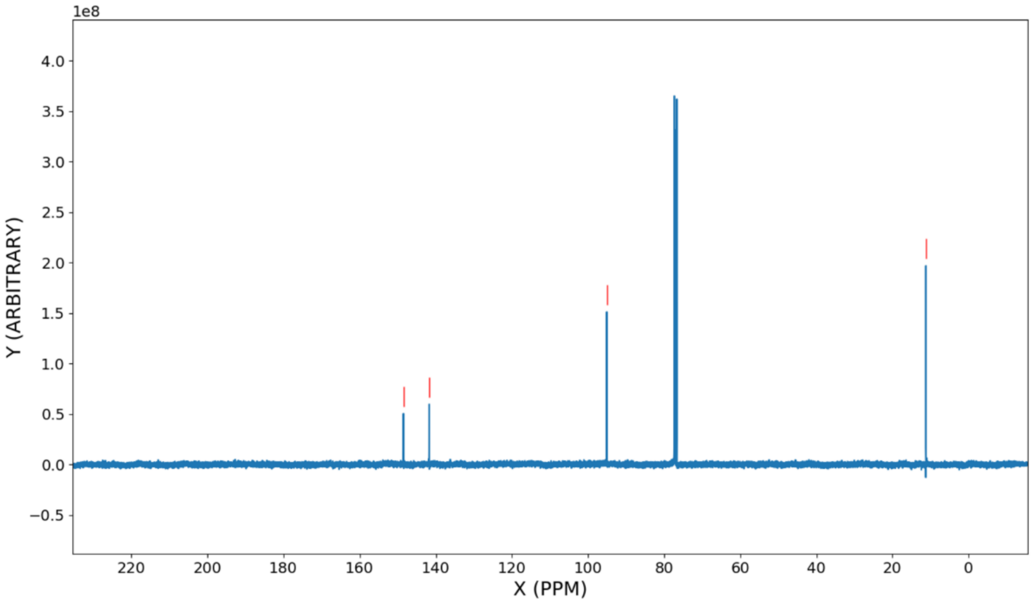

[**19f**] 3-azido-1-benzyl-5-methyl-pyrazole

CHMO:0000593 | ^{1}H nuclear magnetic resonance spectroscopy (^{1}H NMR)

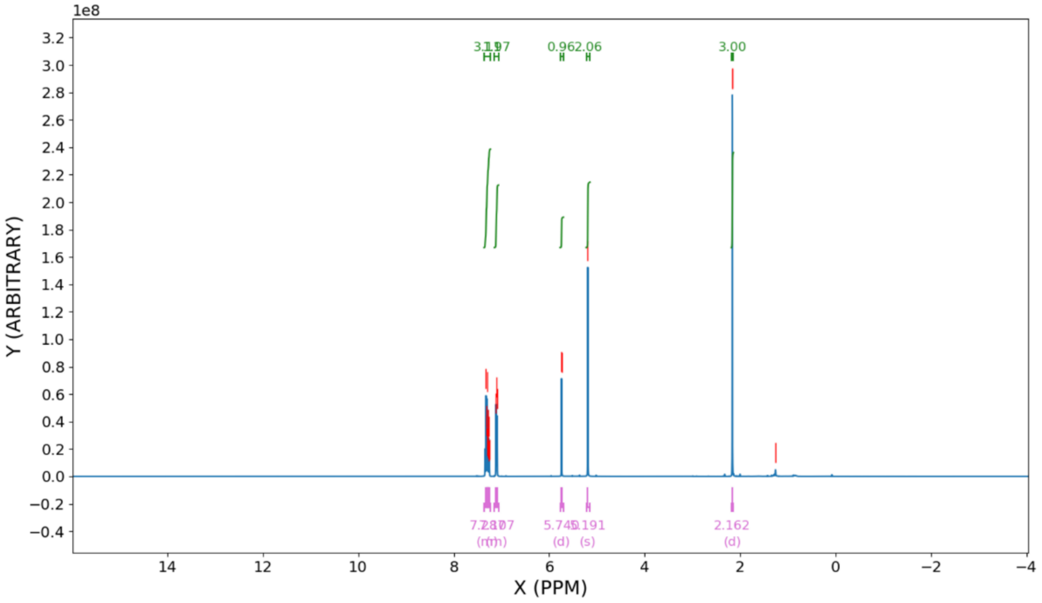

CHMO:0000595 | ^{13}C nuclear magnetic resonance spectroscopy (^{13}C NMR)

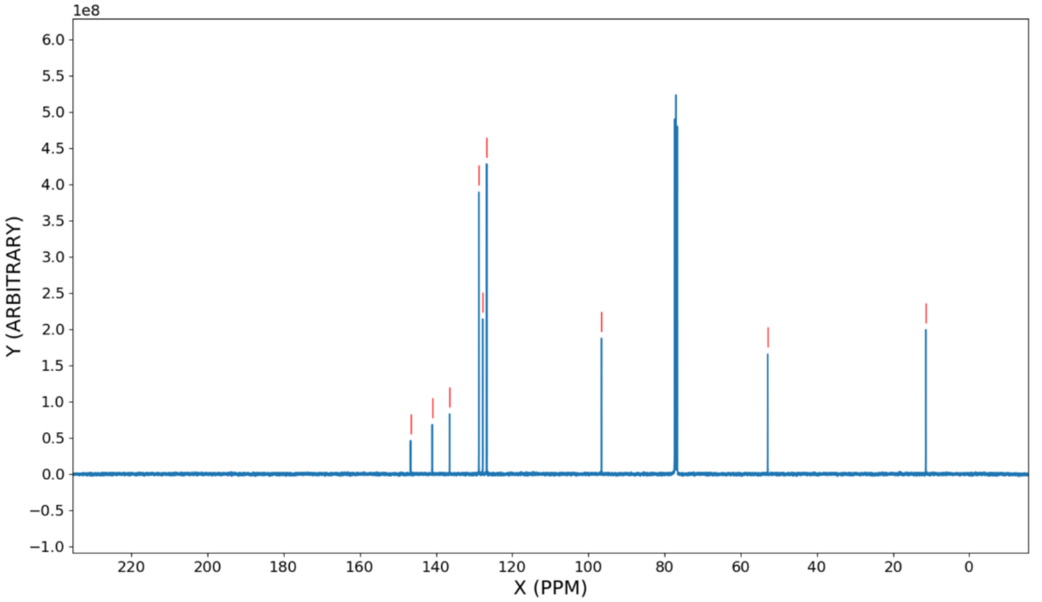

[**19g**] 4-(3-azido-5-methyl-1*H*-pyrazol-1-yl)benzonitrile

CHMO:0000593 | ^{1}H nuclear magnetic resonance spectroscopy (^{1}H NMR)

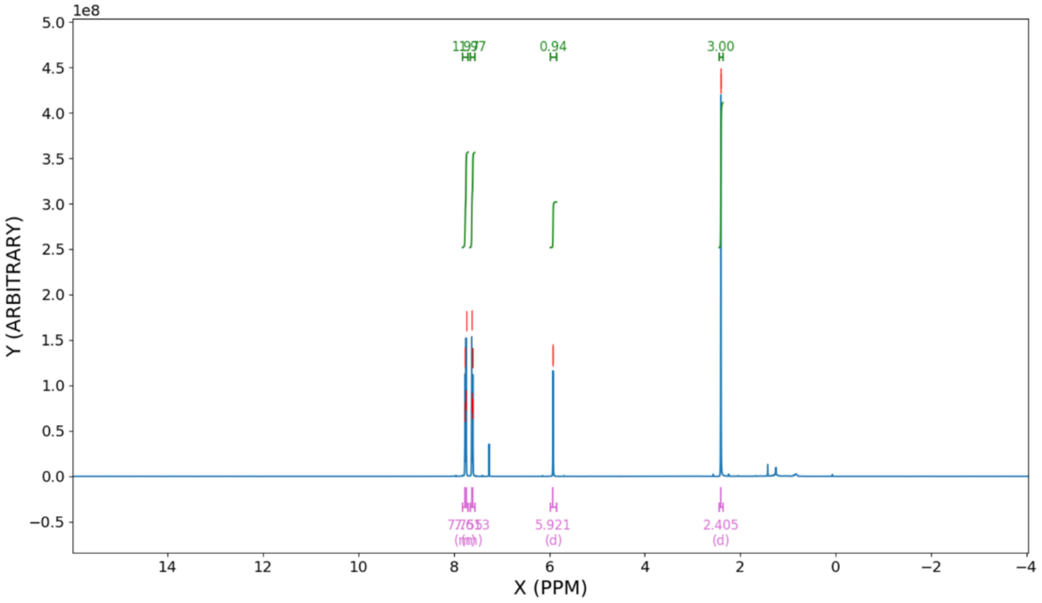

CHMO:0000595 | ^{13}C nuclear magnetic resonance spectroscopy (^{13}C NMR)

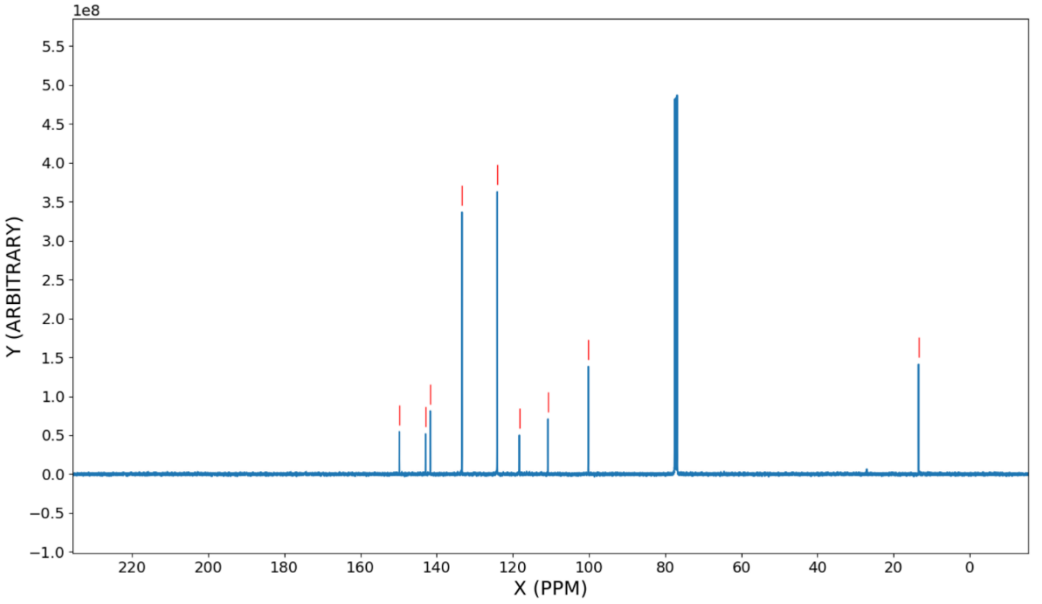

[**19h**] 3-azido-5-methyl-1-(4-nitrophenyl)-1*H*-pyrazole

CHMO:0000593 | ^{1}H nuclear magnetic resonance spectroscopy (^{1}H NMR)

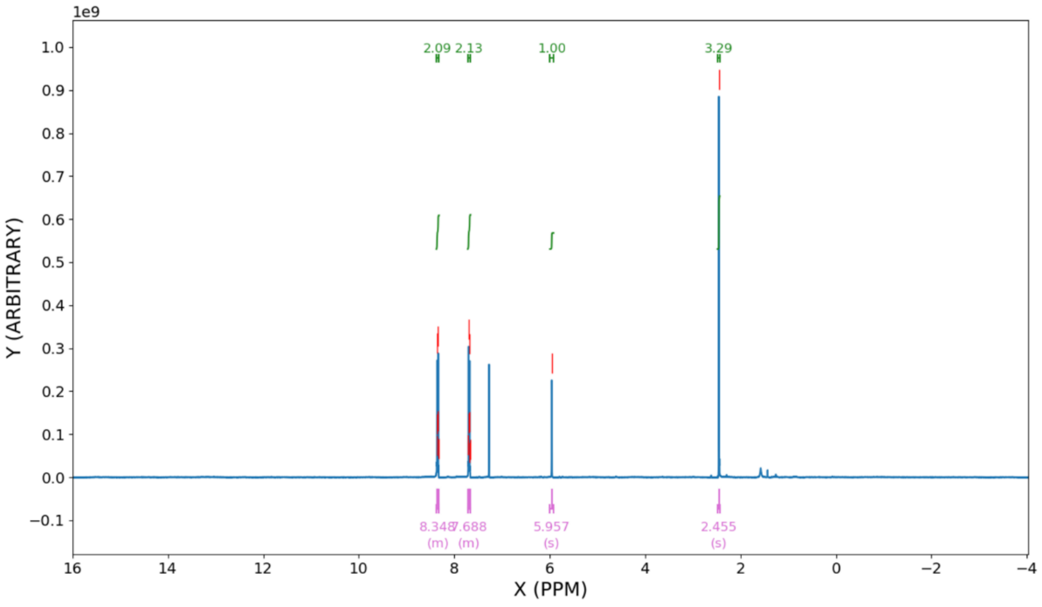

CHMO:0000595 | ^{13}C nuclear magnetic resonance spectroscopy (^{13}C NMR)

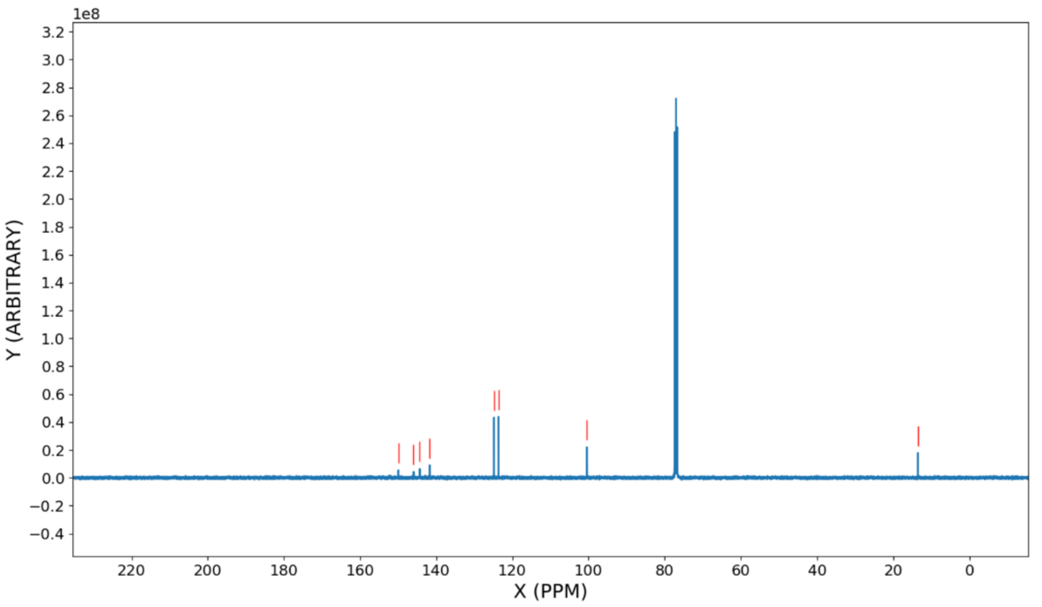

[**19i**] 3-azido-1-(2-chloro-4-nitrophenyl)-5-methyl-1*H*-pyrazole

CHMO:0000593 | ^{1}H nuclear magnetic resonance spectroscopy (^{1}H NMR)

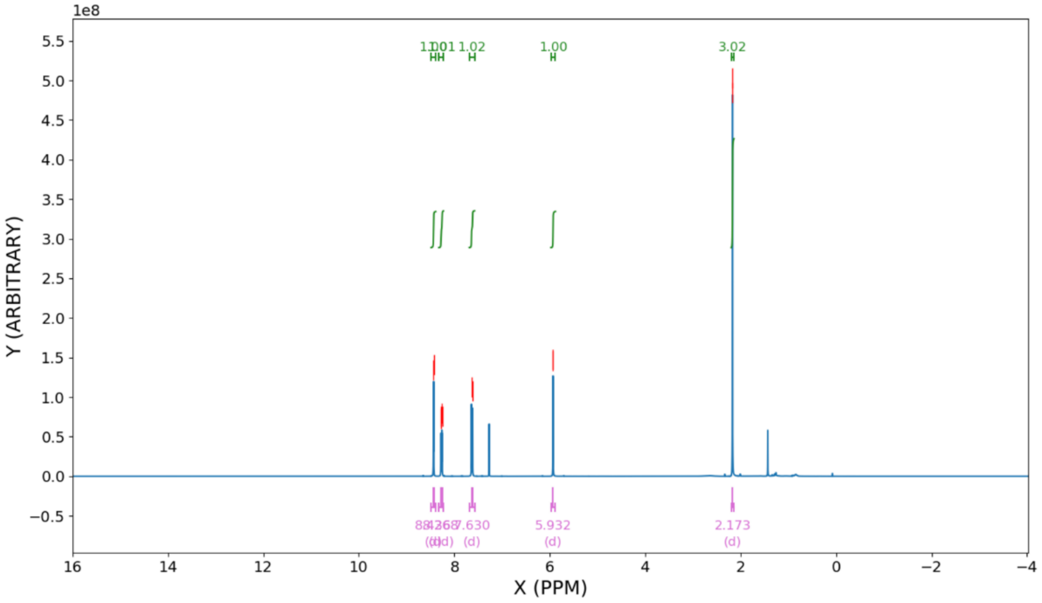

CHMO:0000595 | ^{13}C nuclear magnetic resonance spectroscopy (^{13}C NMR)

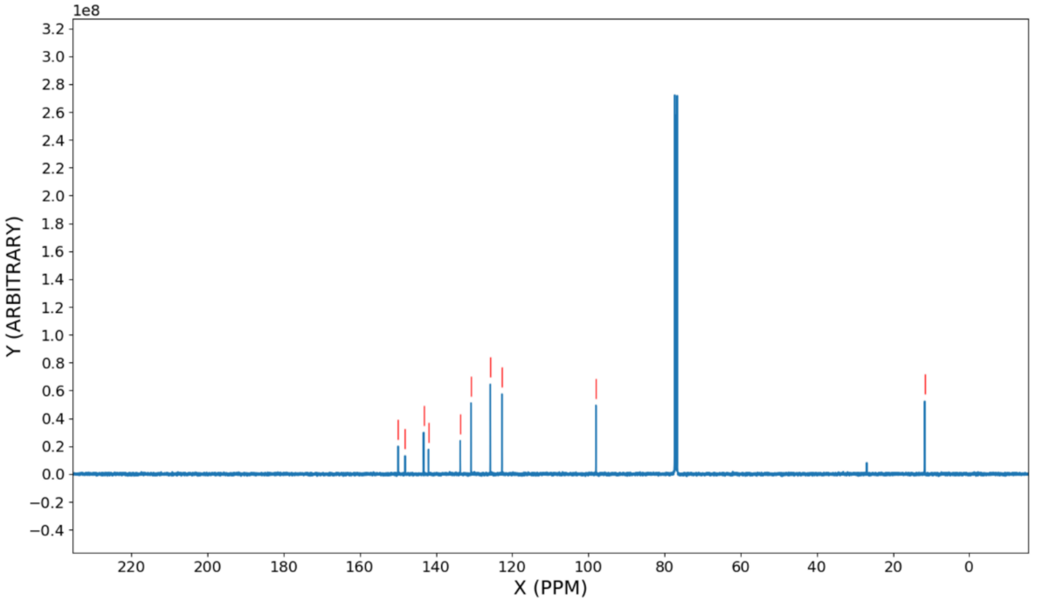

[**19j**] ethyl 5-azido-1*H*-pyrazole-4-carboxylate

CHMO:0000593 | ^{1}H nuclear magnetic resonance spectroscopy (^{1}H NMR)

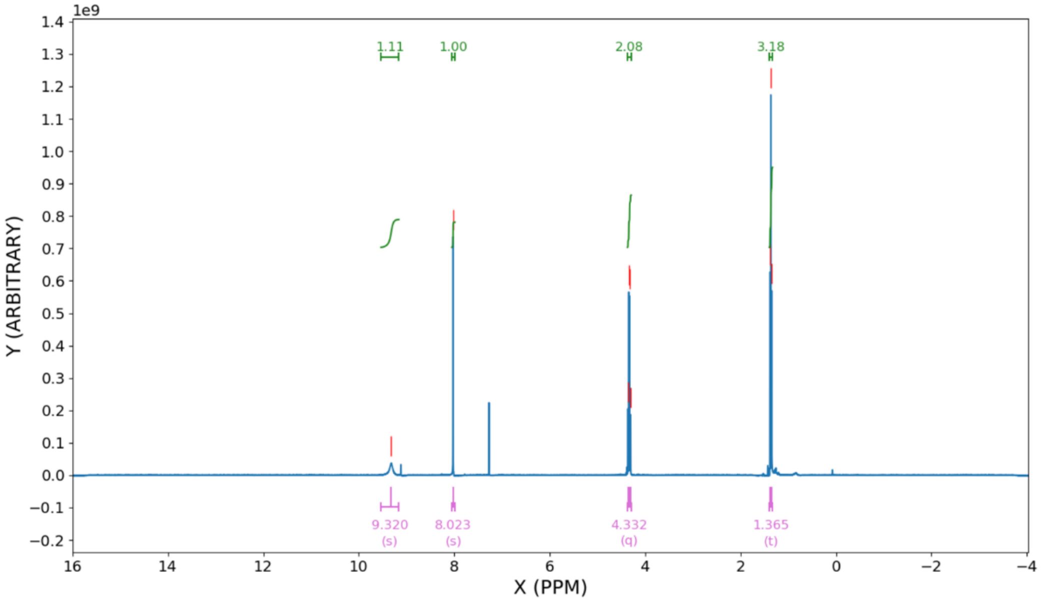

CHMO:0000595 | ^{13}C nuclear magnetic resonance spectroscopy (^{13}C NMR)

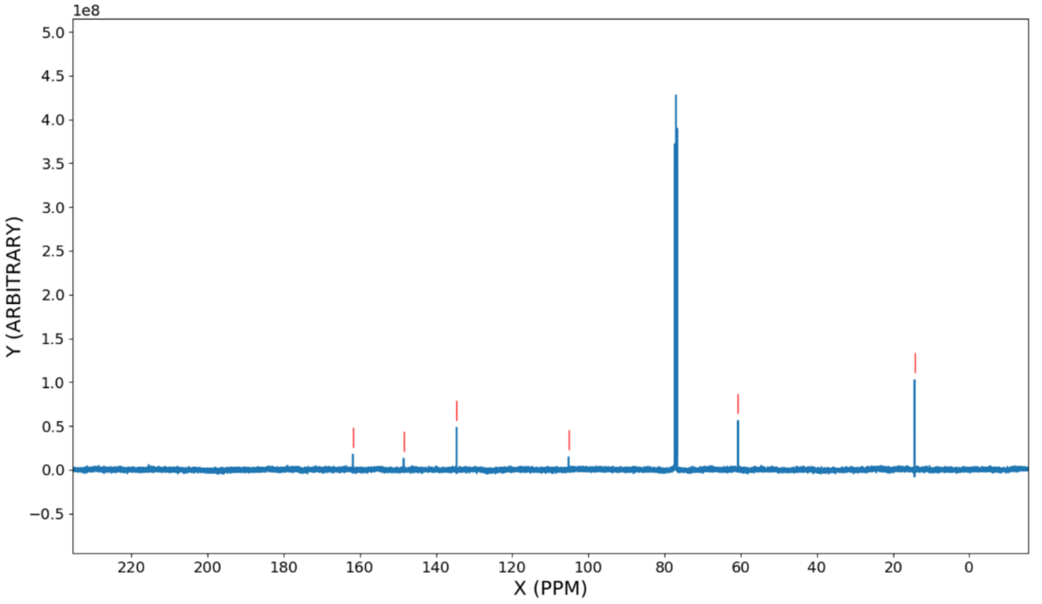

[**19k**] 3-azido-1-benzyl-pyrazole-4-carboxylic acid ethyl ester

CHMO:0000593 | ^{1}H nuclear magnetic resonance spectroscopy (^{1}H NMR)

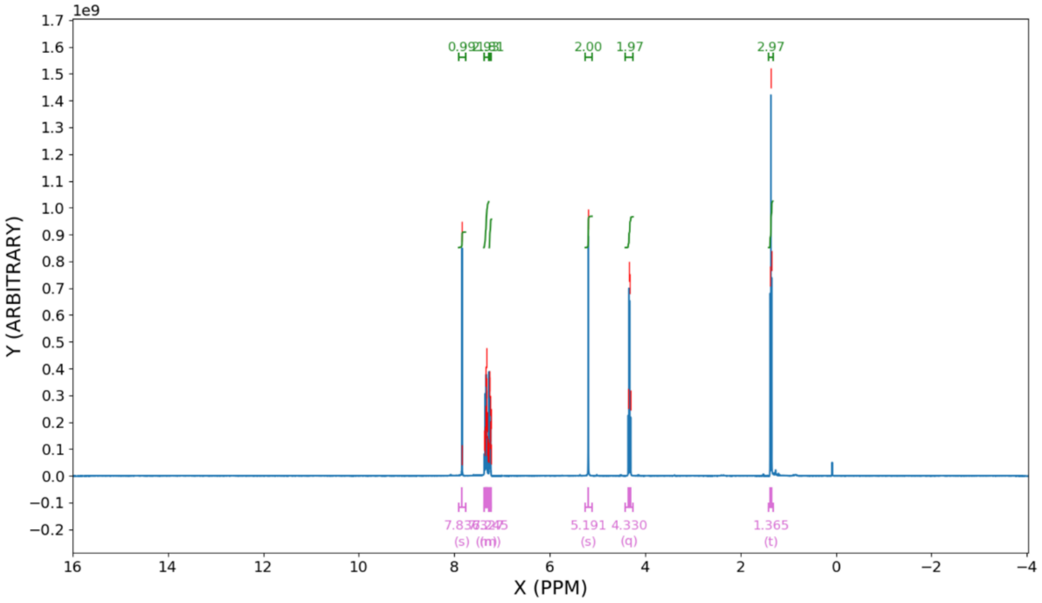

CHMO:0000595 | ^{13}C nuclear magnetic resonance spectroscopy (^{13}C NMR)

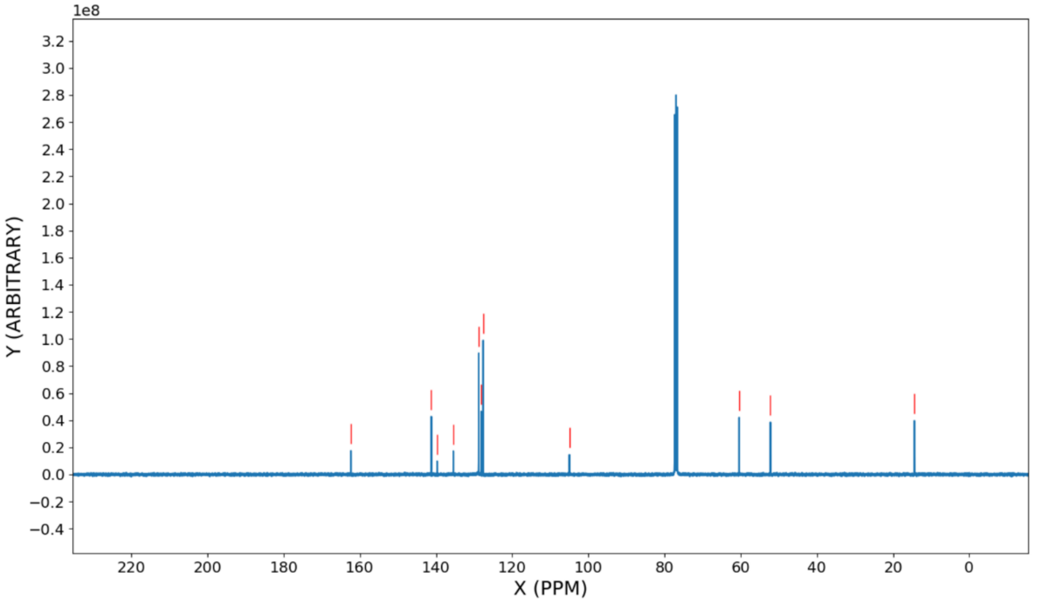

[**19l**] ethyl 3-azido-1-(3,5-difluorobenzyl)-1*H*-pyrazole-4-carboxylate

CHMO:0000593 | ^{1}H nuclear magnetic resonance spectroscopy (^{1}H NMR)

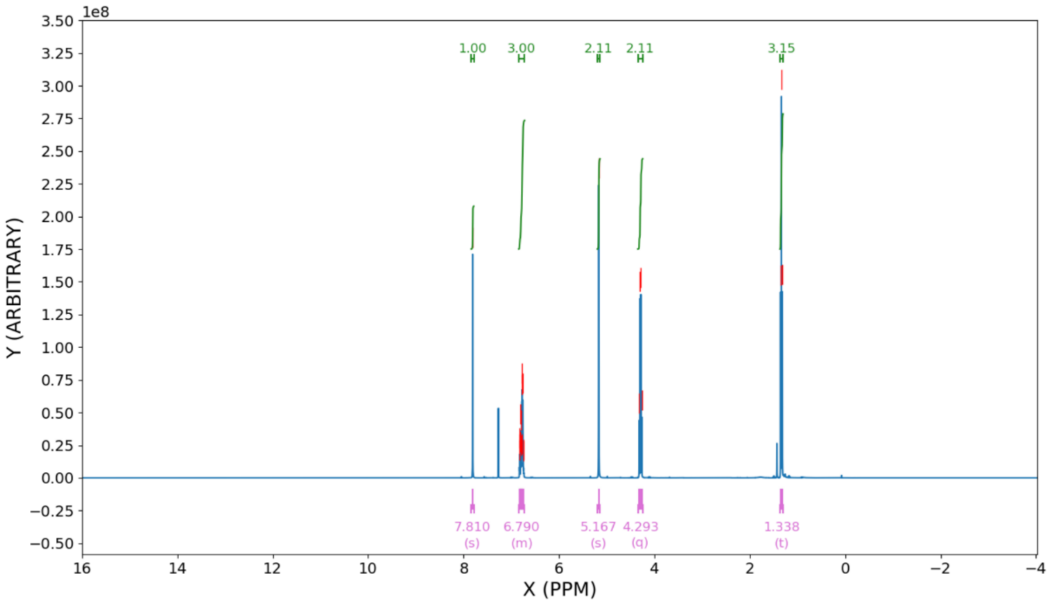

CHMO:0000595 | ^{13}C nuclear magnetic resonance spectroscopy (^{13}C NMR)

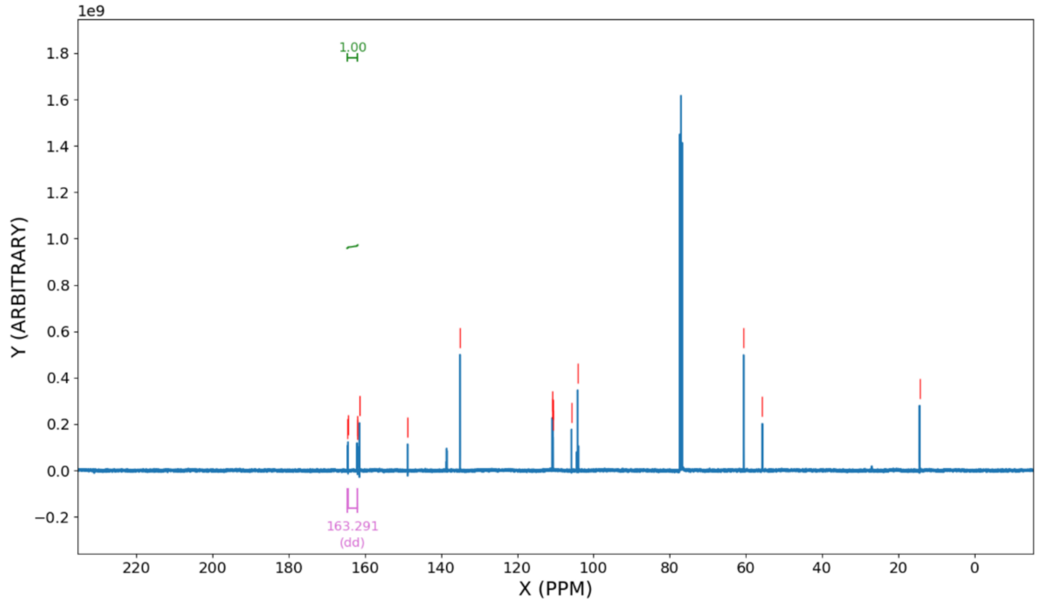

[**19m**] ethyl 3-azido-1-(3-cyanobenzyl)-1*H*-pyrazole-4-carboxylate

CHMO:0000593 | ^{1}H nuclear magnetic resonance spectroscopy (^{1}H NMR)

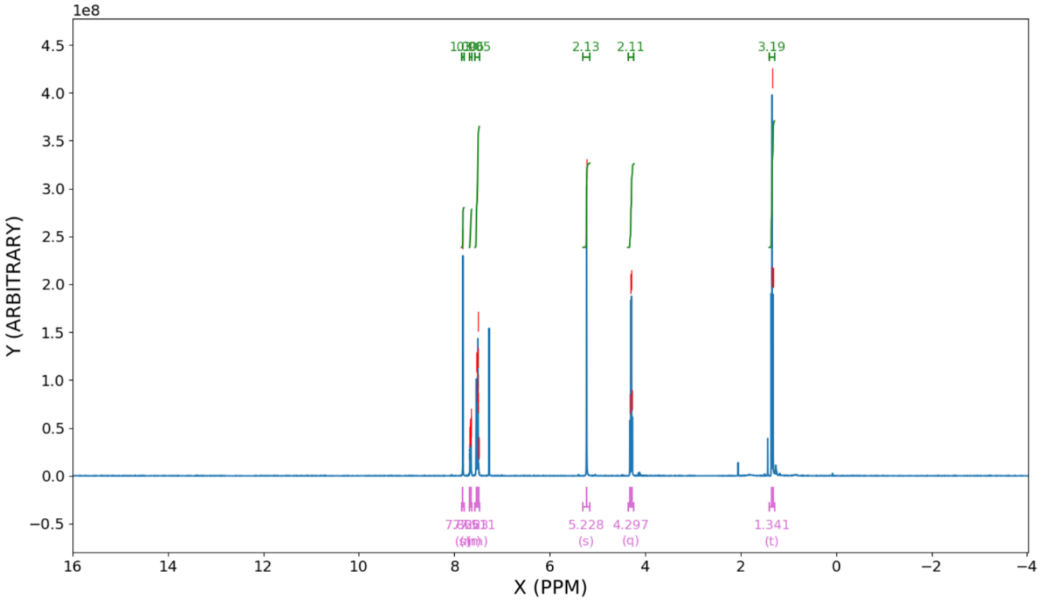

CHMO:0000595 | ^{13}C nuclear magnetic resonance spectroscopy (^{13}C NMR)

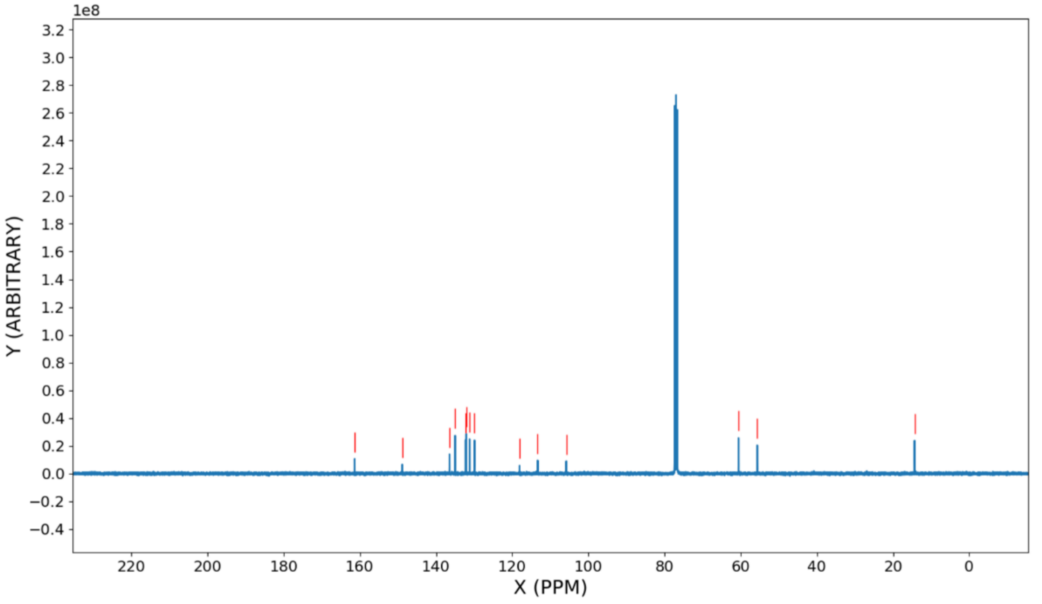

[**19n**] ethyl 3-azido-1-(4-bromobenzyl)-1*H*-pyrazole-4-carboxylate

CHMO:0000593 | ^{1}H nuclear magnetic resonance spectroscopy (^{1}H NMR)

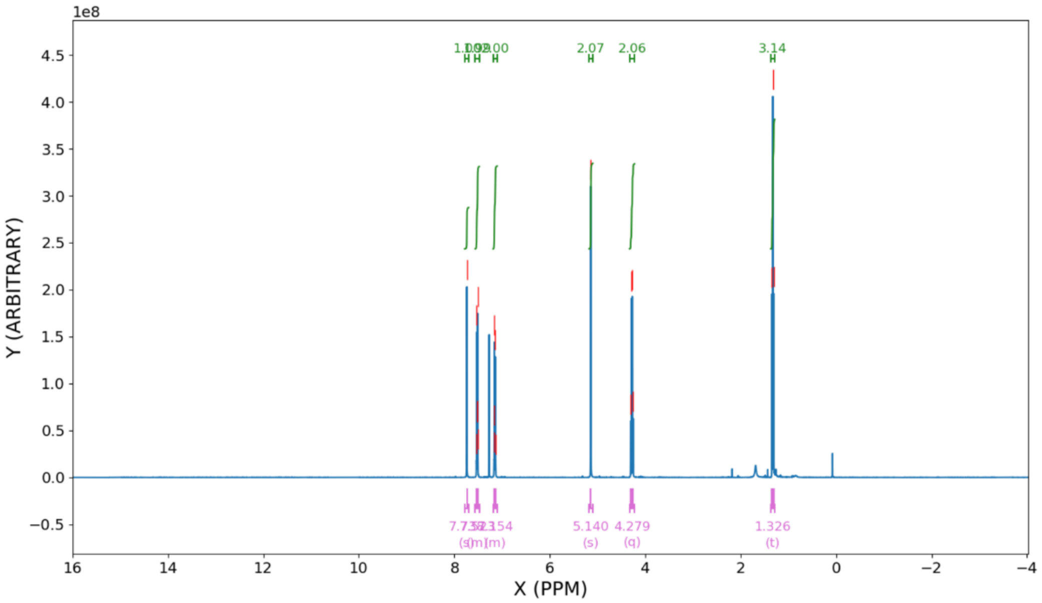

CHMO:0000595 | ^{13}C nuclear magnetic resonance spectroscopy (^{13}C NMR)

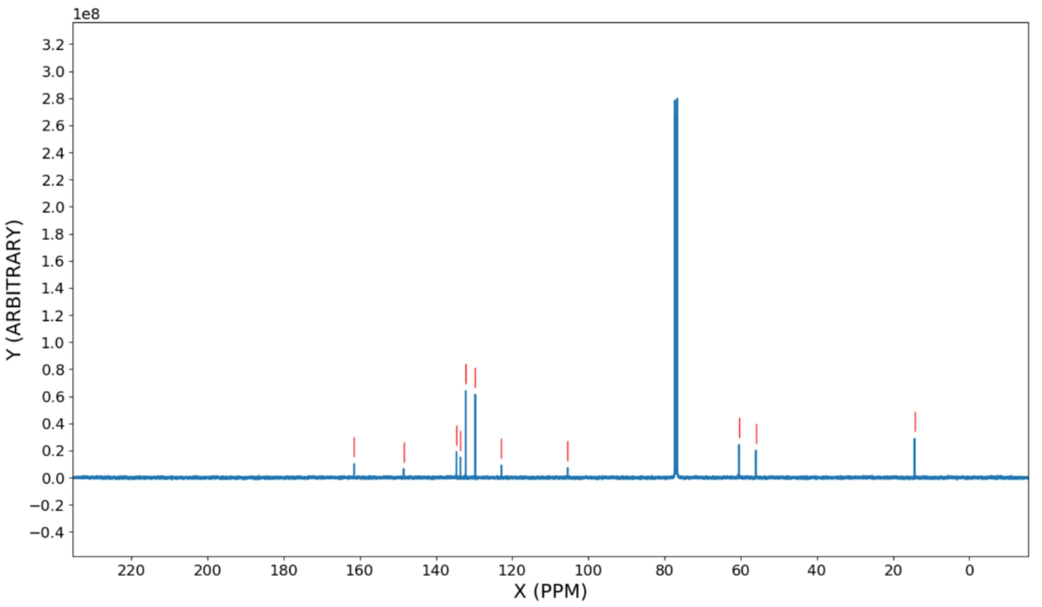

[**19o**] 5-azido-1*H*-pyrazole-4-carbonitrile

CHMO:0000593 | ^{1}H nuclear magnetic resonance spectroscopy (^{1}H NMR)

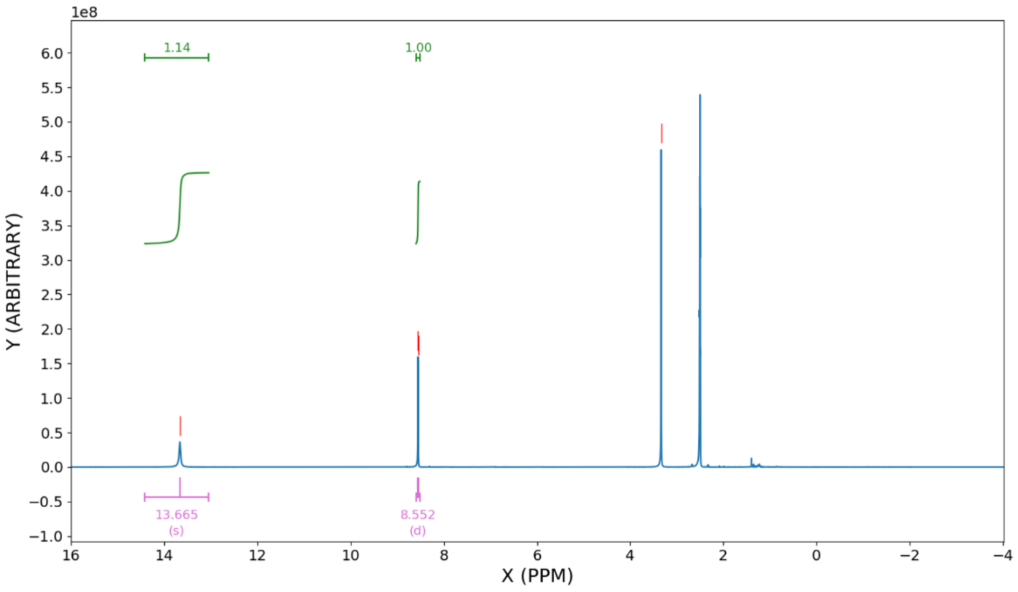

CHMO:0000595 | ^{13}C nuclear magnetic resonance spectroscopy (^{13}C NMR)

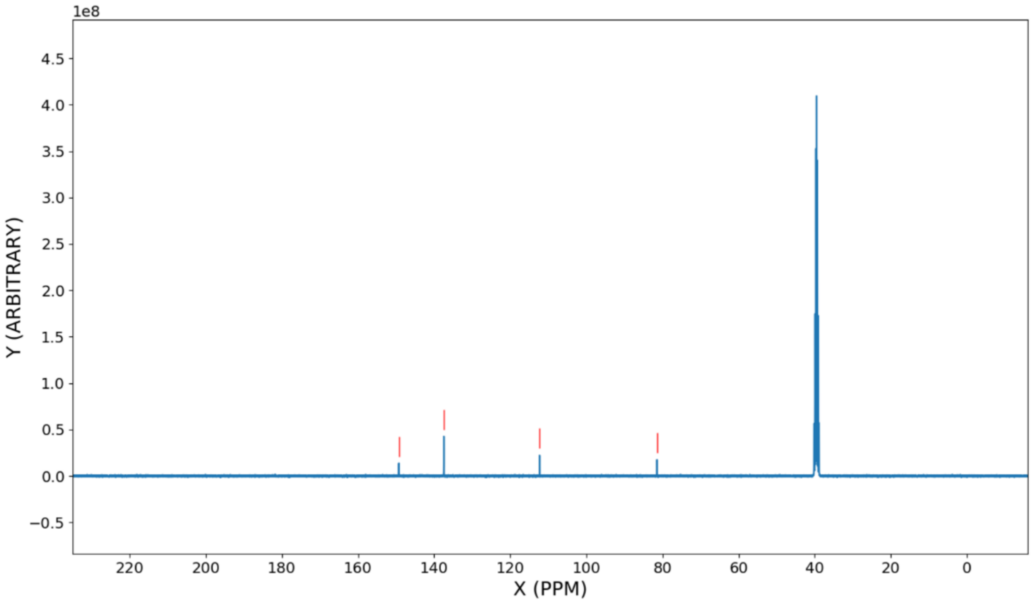

[**19p**] 3-azido-1-propan-2-yl-pyrazole-4-carbonitrile

CHMO:0000593 | ^{1}H nuclear magnetic resonance spectroscopy (^{1}H NMR)

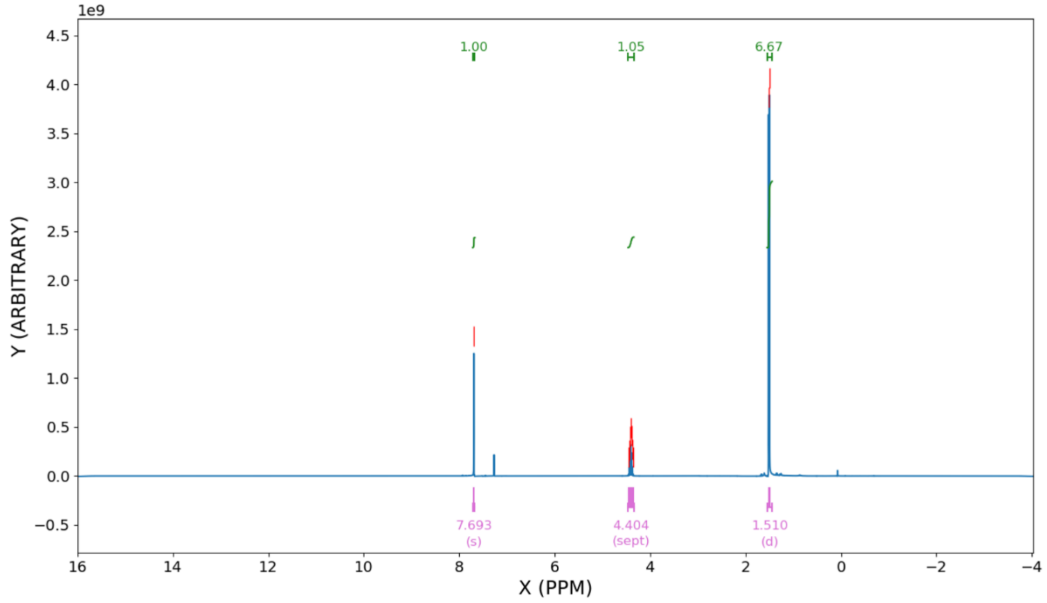

CHMO:0000595 | ^{13}C nuclear magnetic resonance spectroscopy (^{13}C NMR)

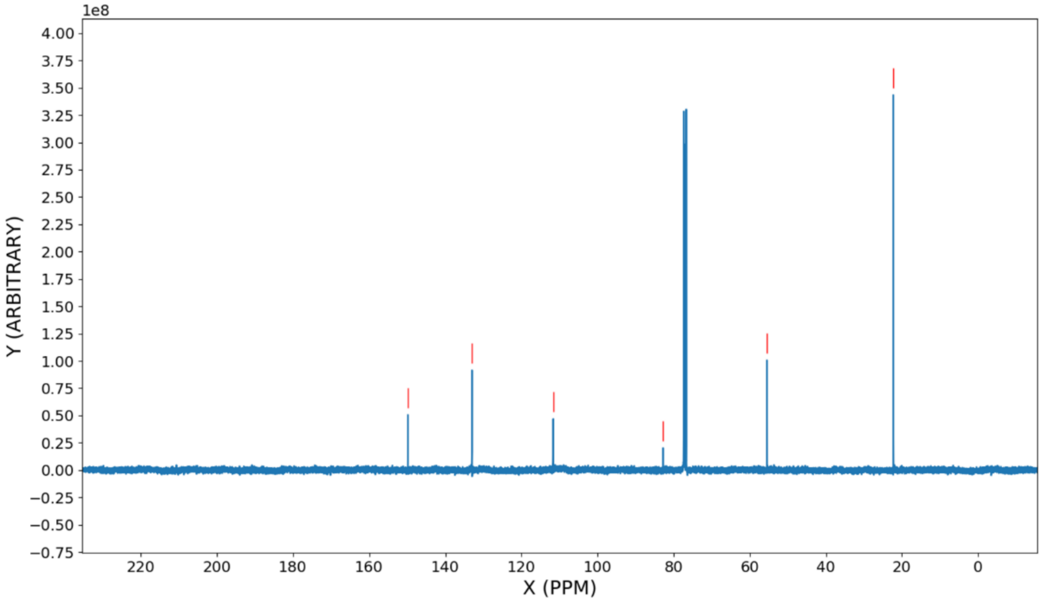

[**19q**] ethyl 2-(3-azido-4-cyano-1*H*-pyrazol-1-yl)acetate

CHMO:0000593 | ^{1}H nuclear magnetic resonance spectroscopy (^{1}H NMR)

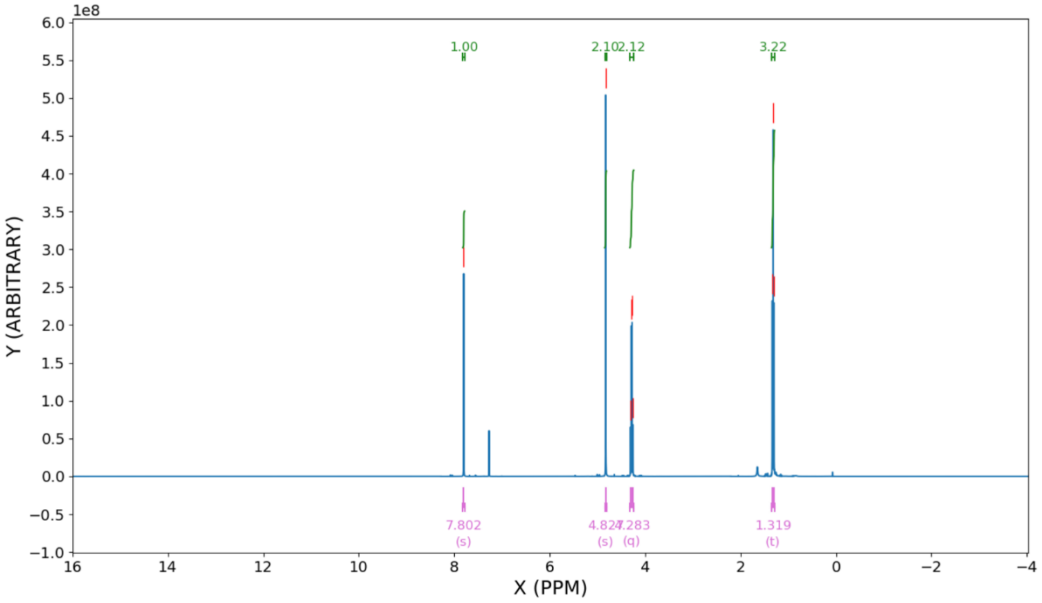

CHMO:0000595 | ^{13}C nuclear magnetic resonance spectroscopy (^{13}C NMR)

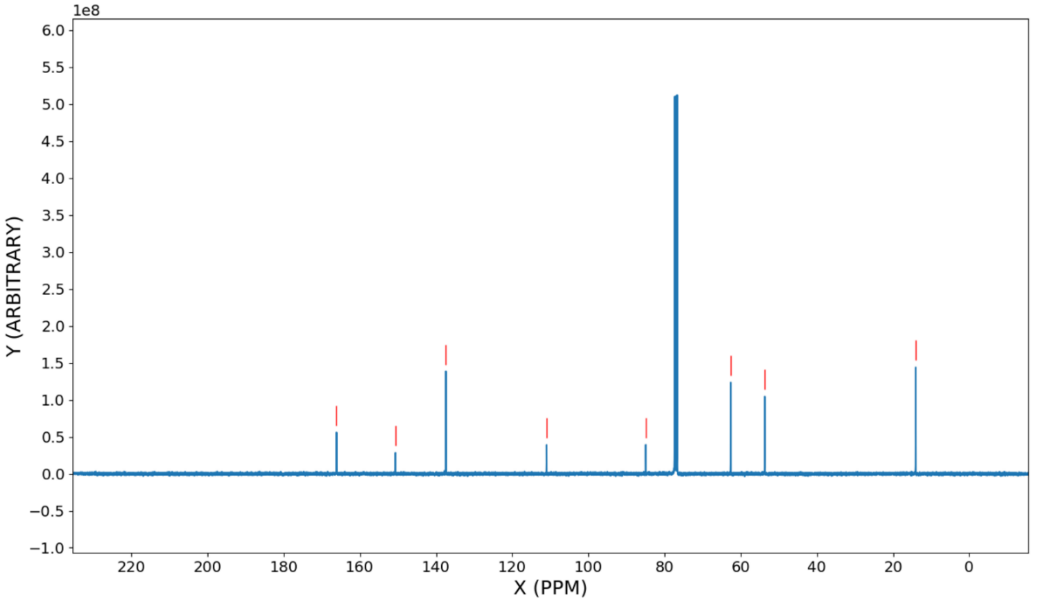

[**19r**] 3-azido-1-benzyl-1*H*-pyrazole-4-carbonitrile

CHMO:0000593 | ^{1}H nuclear magnetic resonance spectroscopy (^{1}H NMR)

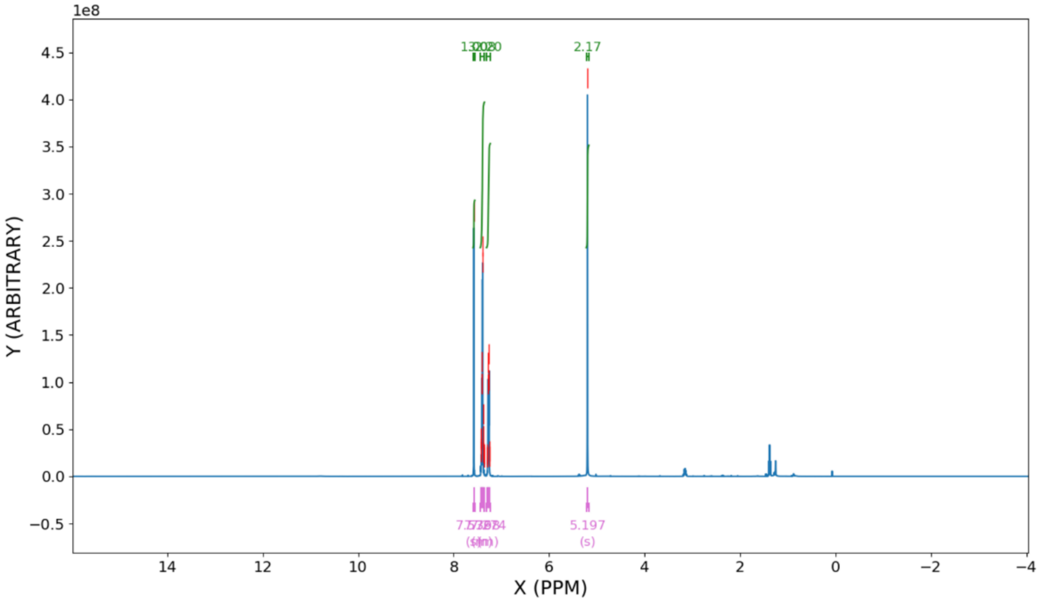

CHMO:0000595 | ^{13}C nuclear magnetic resonance spectroscopy (^{13}C NMR)

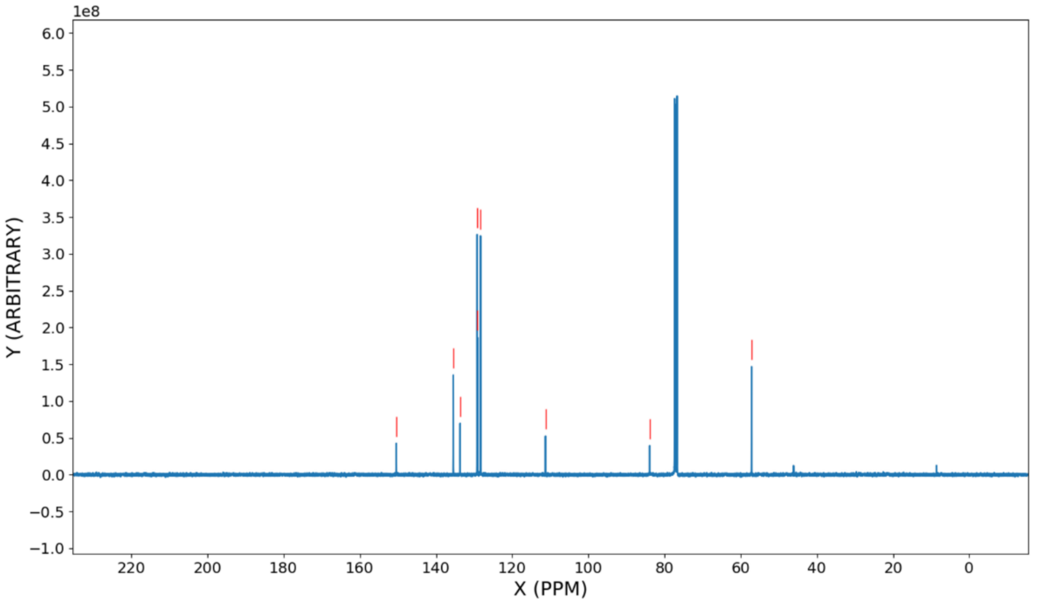

[**19s**] 3-azido-1-(3,5-difluorobenzyl)-1*H*-pyrazole-4-carbonitrile

CHMO:0000593 | ^{1}H nuclear magnetic resonance spectroscopy (^{1}H NMR)

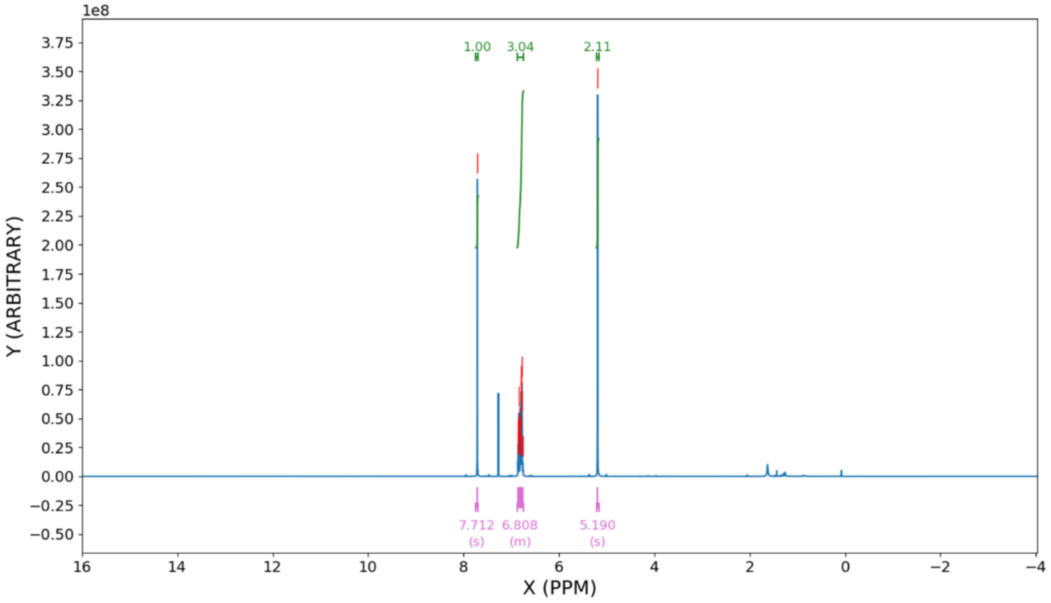

CHMO:0000595 | ^{13}C nuclear magnetic resonance spectroscopy (^{13}C NMR)

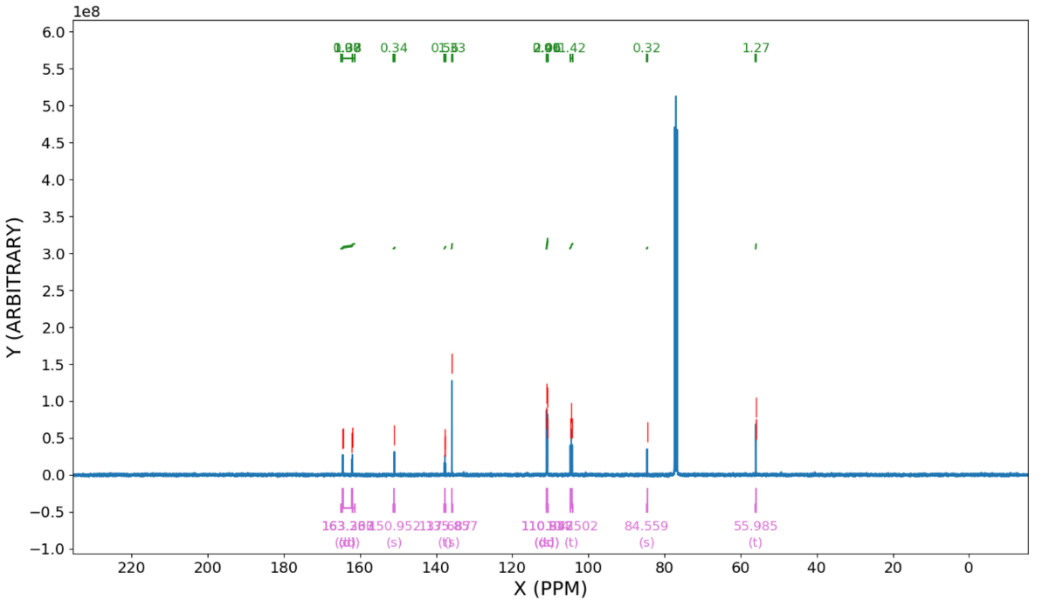

[**19t**] 3-azido-1-(4-methylbenzyl)-1*H*-pyrazole-4-carbonitrile

CHMO:0000593 | ^{1}H nuclear magnetic resonance spectroscopy (^{1}H NMR)

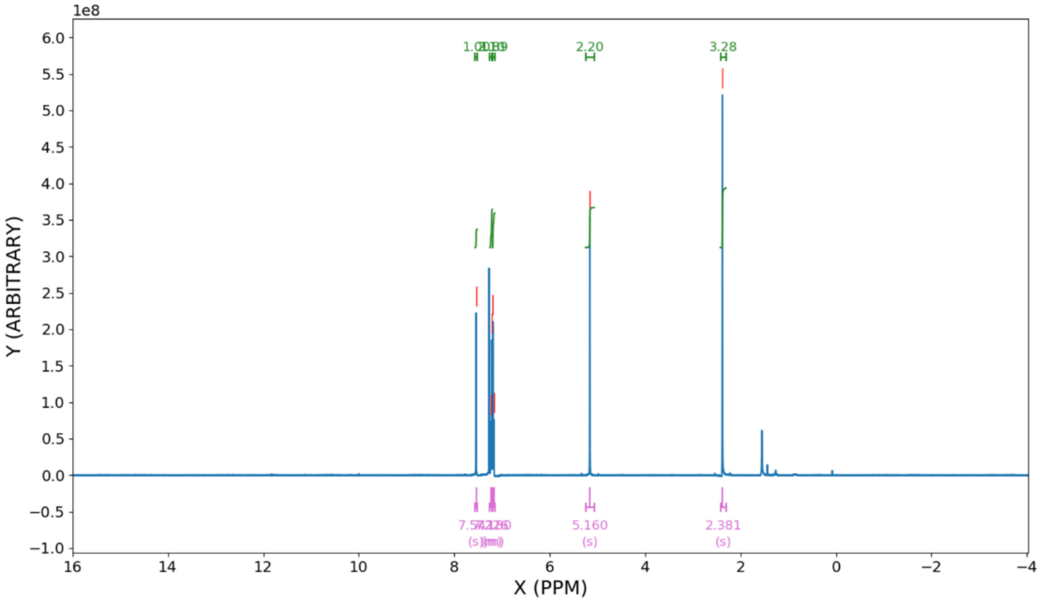

CHMO:0000595 | ^{13}C nuclear magnetic resonance spectroscopy (^{13}C NMR)

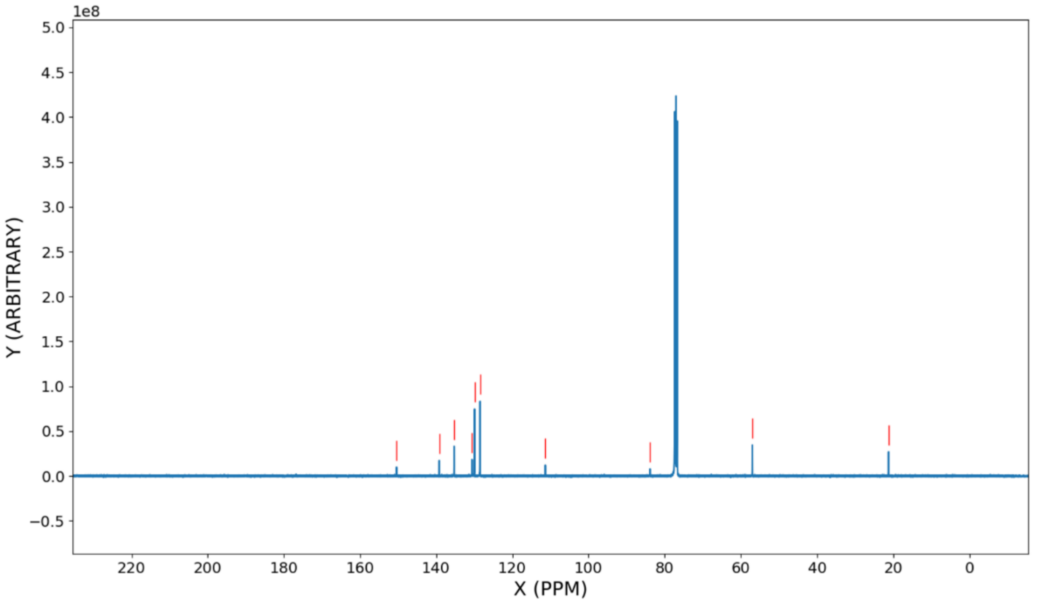

[**19v**] 3-azido-1-(4-fluorobenzyl)-1*H*-pyrazole-4-carbonitrile

CHMO:0000593 | ^{1}H nuclear magnetic resonance spectroscopy (^{1}H NMR)

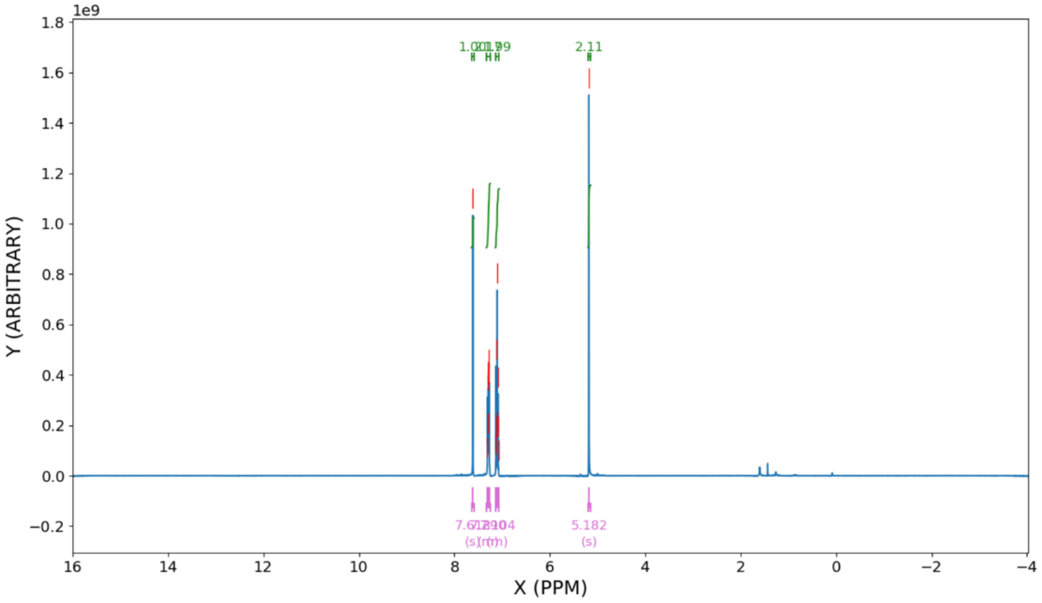

CHMO:0000595 | ^{13}C nuclear magnetic resonance spectroscopy (^{13}C NMR)

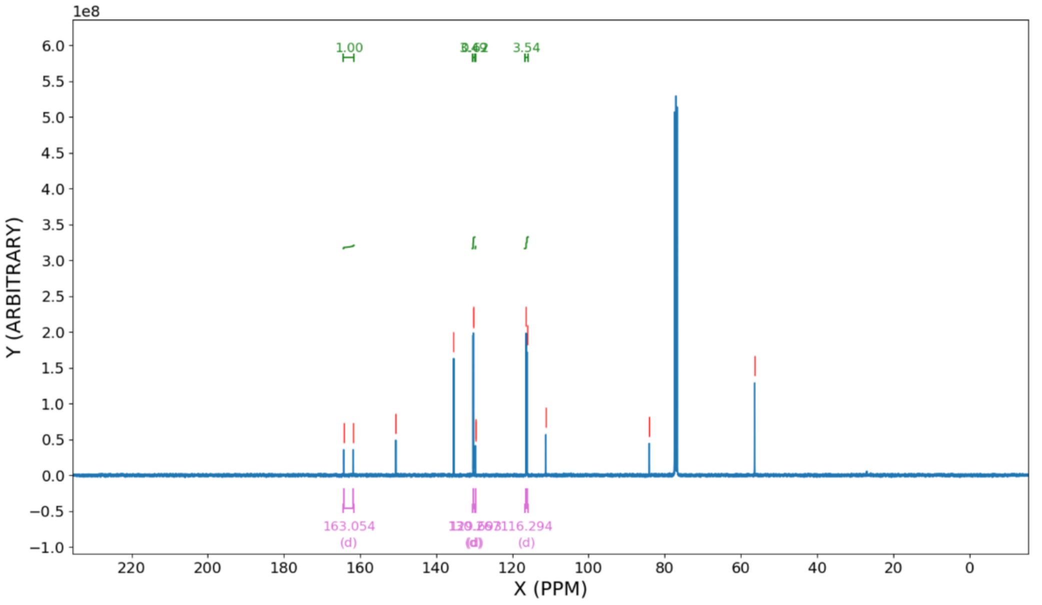

[**21aa**] 4-butyl-1-(1*H*-pyrazol-3-yl)-1*H*-1,2,3-triazole

CHMO:0000593 | ^{1}H nuclear magnetic resonance spectroscopy (^{1}H NMR)

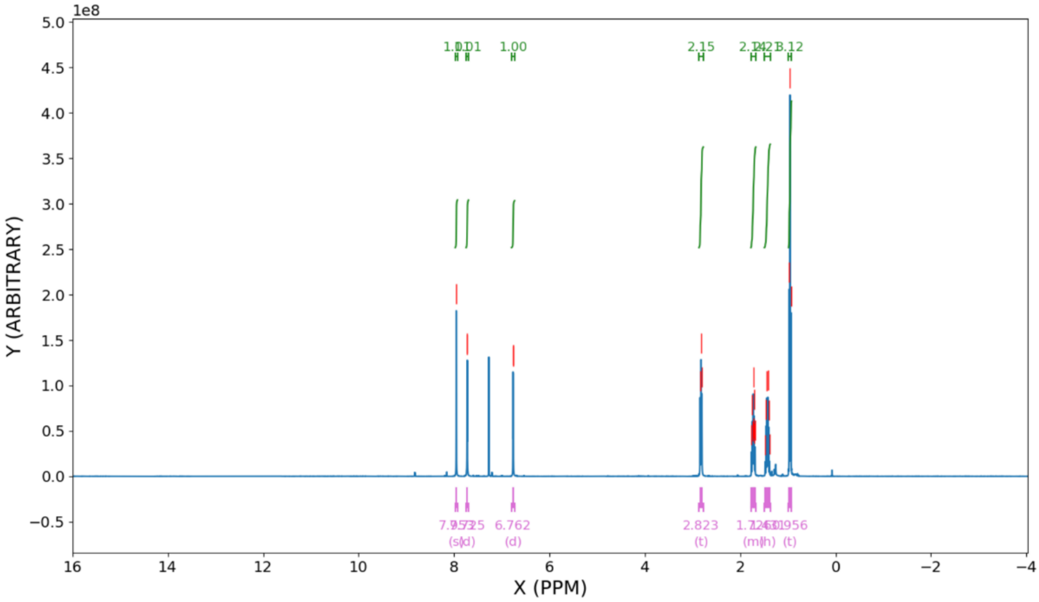

CHMO:0000595 | ^{13}C nuclear magnetic resonance spectroscopy (^{13}C NMR)

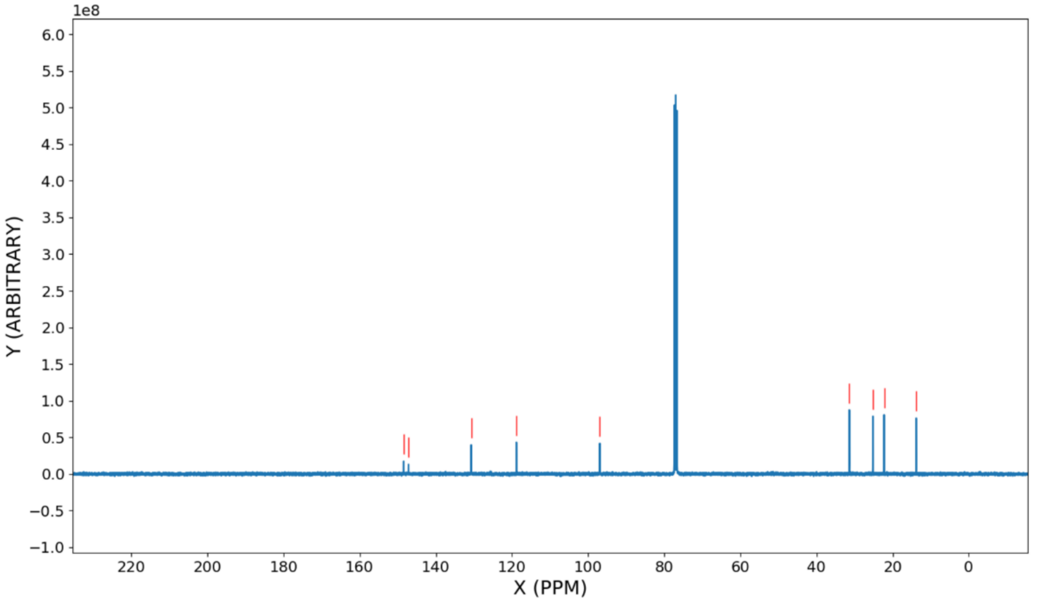

[**21ab**] methyl 1-(1*H*-pyrazol-5-yl)triazole-4-carboxylate

CHMO:0000593 | ^{1}H nuclear magnetic resonance spectroscopy (^{1}H NMR)

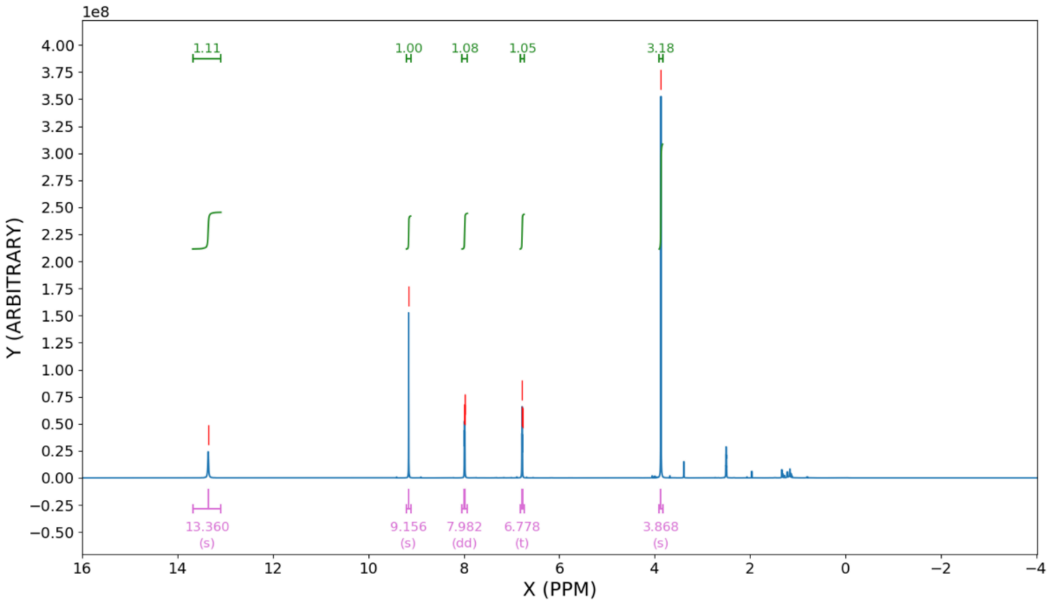

CHMO:0000595 | ^{13}C nuclear magnetic resonance spectroscopy (^{13}C NMR)

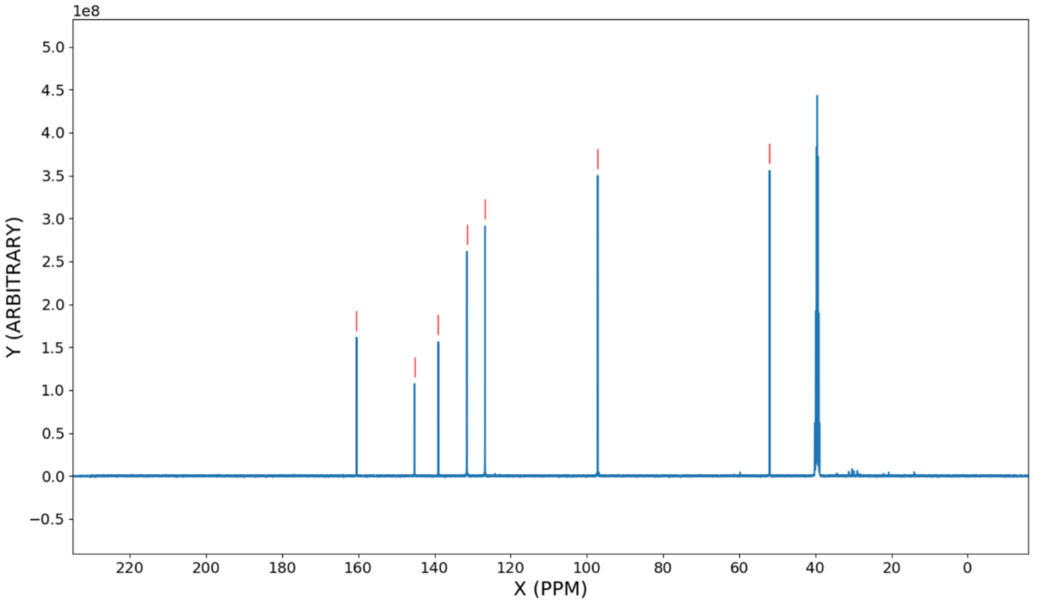

[**21ae**] 4-(4-methoxyphenyl)-1-(1*H*-pyrazol-3-yl)-1*H*-1,2,3-triazole

CHMO:0000593 | ^{1}H nuclear magnetic resonance spectroscopy (^{1}H NMR)

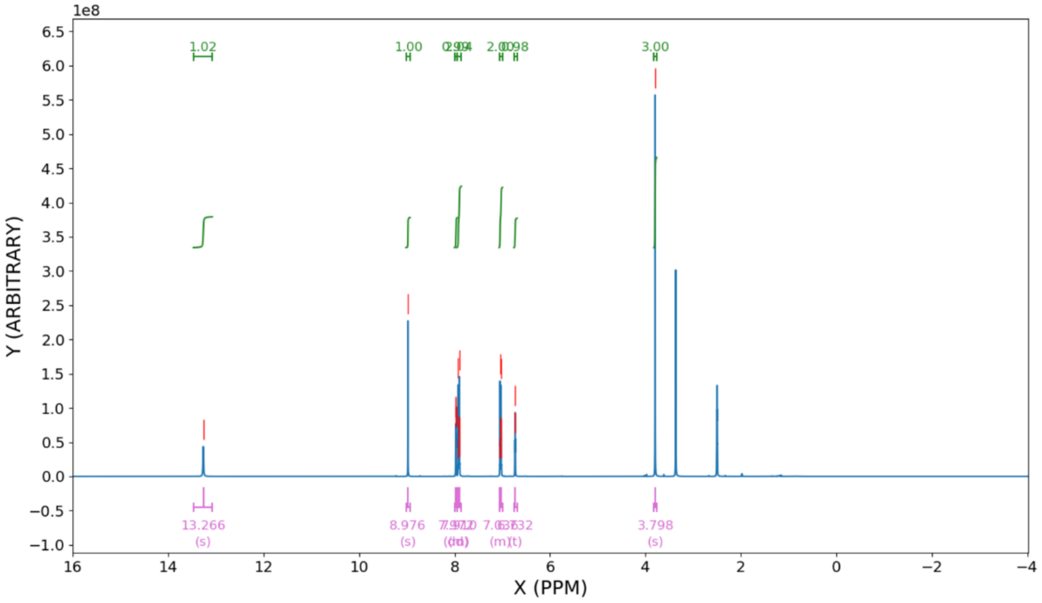

CHMO:0000595 | ^{13}C nuclear magnetic resonance spectroscopy (^{13}C NMR)

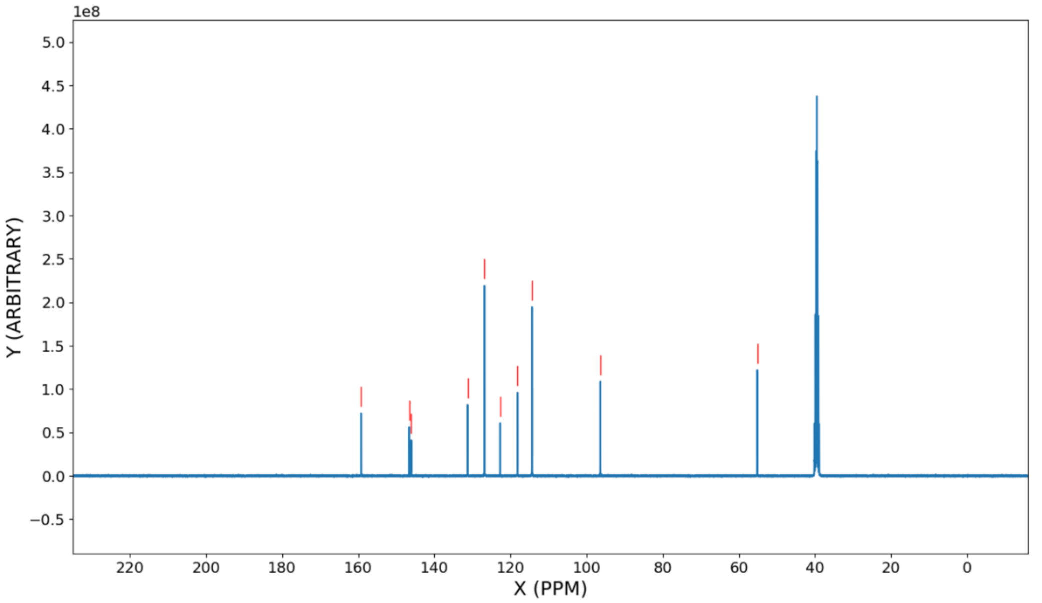

[**21bb**] methyl 1-(1-cyclopentyl-1*H*-pyrazol-3-yl)-1*H*-1,2,3-triazole-4-carboxylate

CHMO:0000593 | ^{1}H nuclear magnetic resonance spectroscopy (^{1}H NMR)

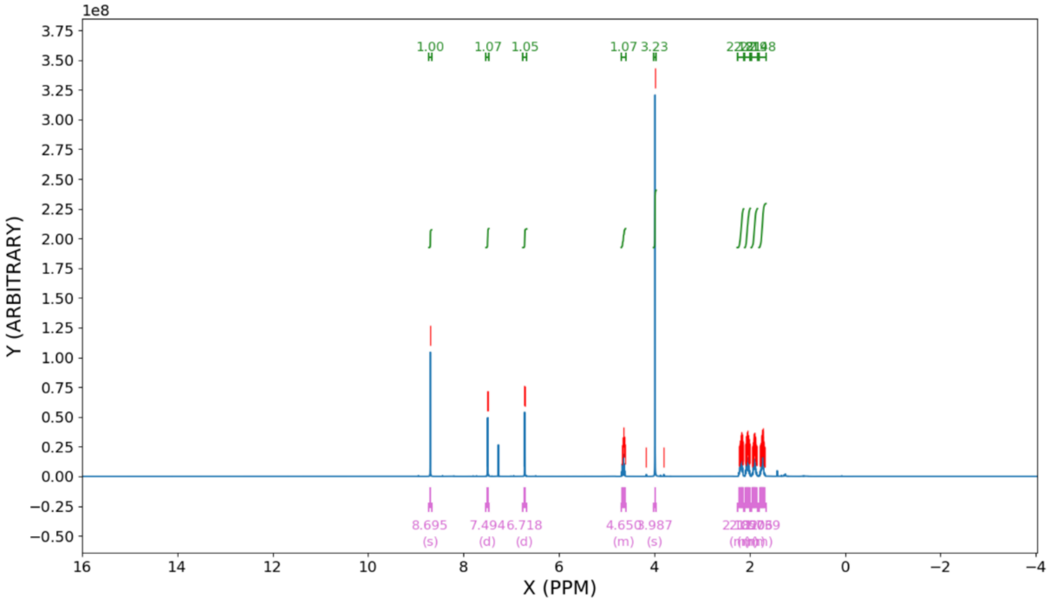

CHMO:0000595 | ^{13}C nuclear magnetic resonance spectroscopy (^{13}C NMR)

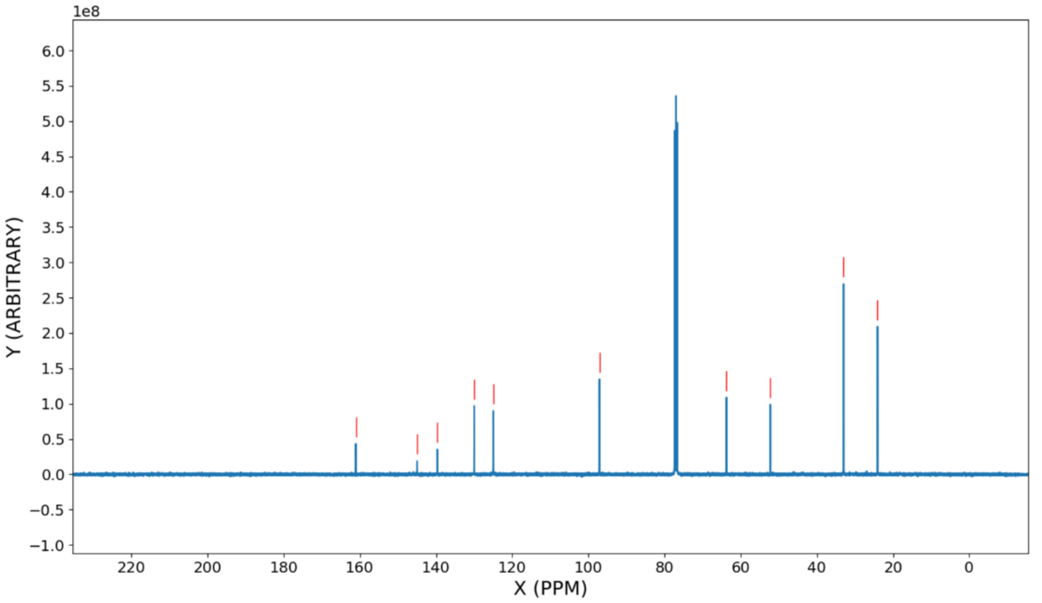

[**21be**] 1-(1-cyclopentyl-1*H*-pyrazol-3-yl)-4-(4-methoxyphenyl)-1*H*-1,2,3-triazole

CHMO:0000593 | ^{1}H nuclear magnetic resonance spectroscopy (^{1}H NMR)

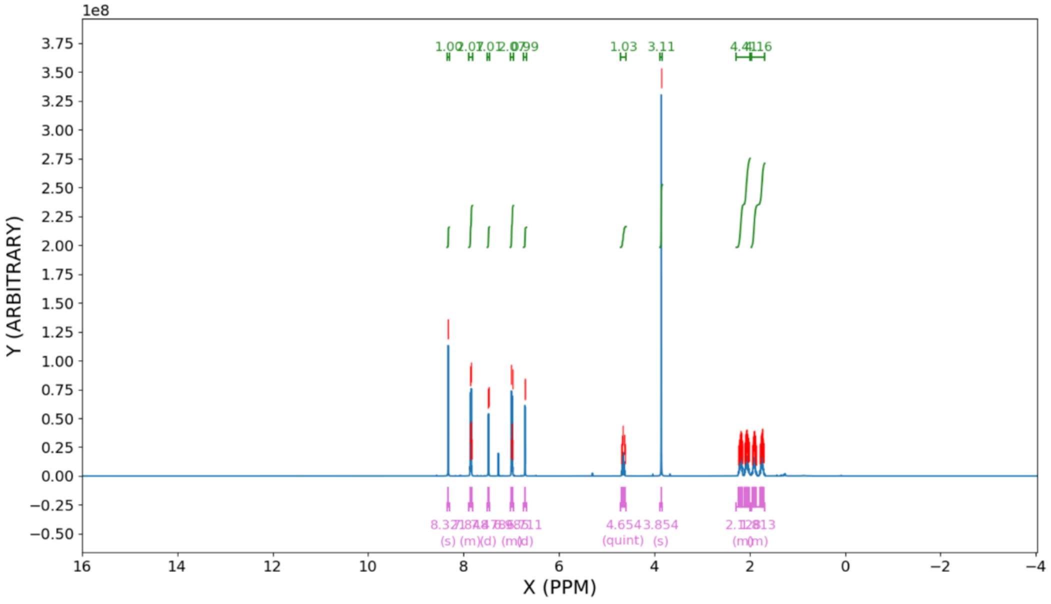

CHMO:0000595 | ^{13}C nuclear magnetic resonance spectroscopy (^{13}C NMR)

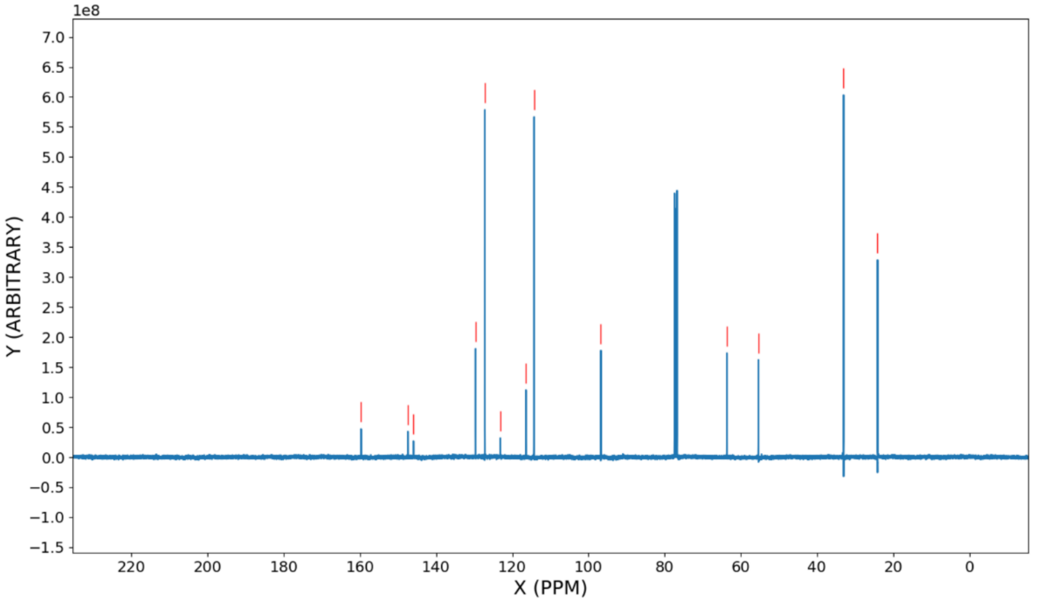

[**21ce**] 1-(1-benzyl-1*H*-pyrazol-3-yl)-4-(4-methoxyphenyl)-1*H*-1,2,3-triazole

CHMO:0000593 | ^{1}H nuclear magnetic resonance spectroscopy (^{1}H NMR)

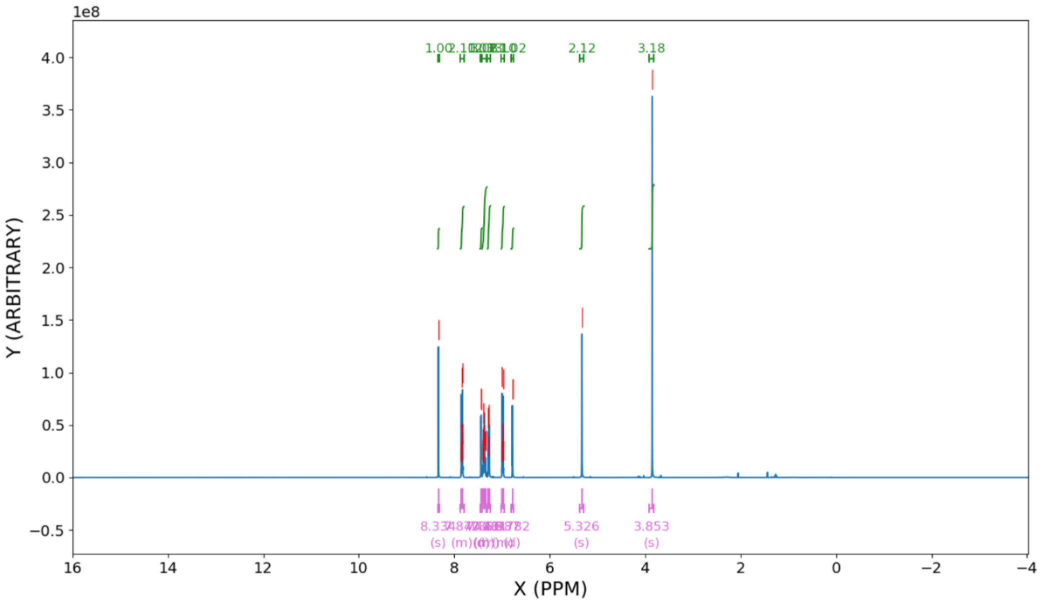

CHMO:0000595 | ^{13}C nuclear magnetic resonance spectroscopy (^{13}C NMR)

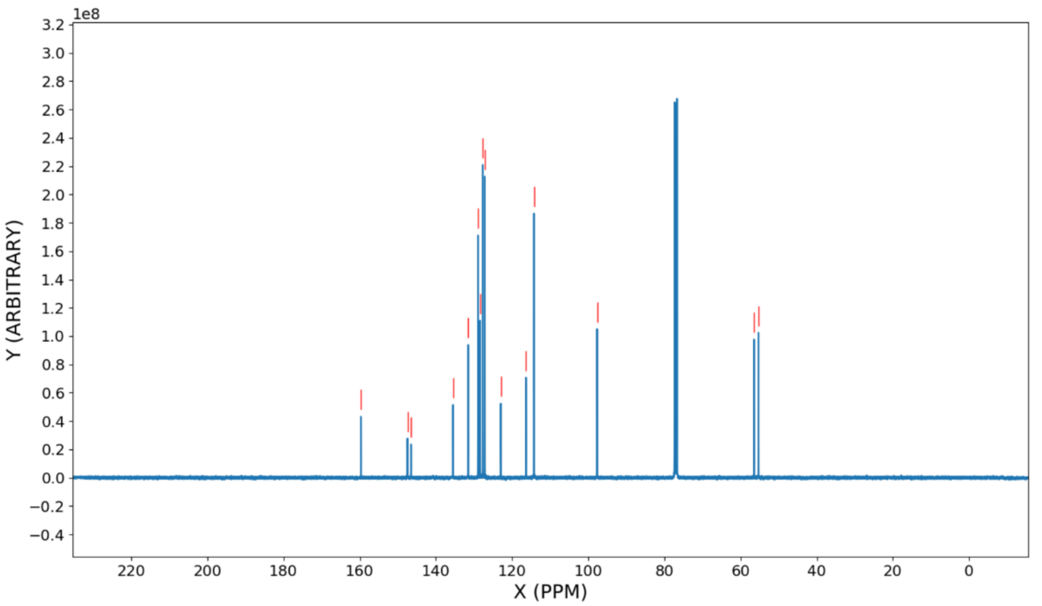

[**21db**] methyl 1-(1-(4-bromobenzyl)-1*H*-pyrazol-3-yl)-1*H*-1,2,3-triazole-4-carboxylate

CHMO:0000593 | ^{1}H nuclear magnetic resonance spectroscopy (^{1}H NMR)

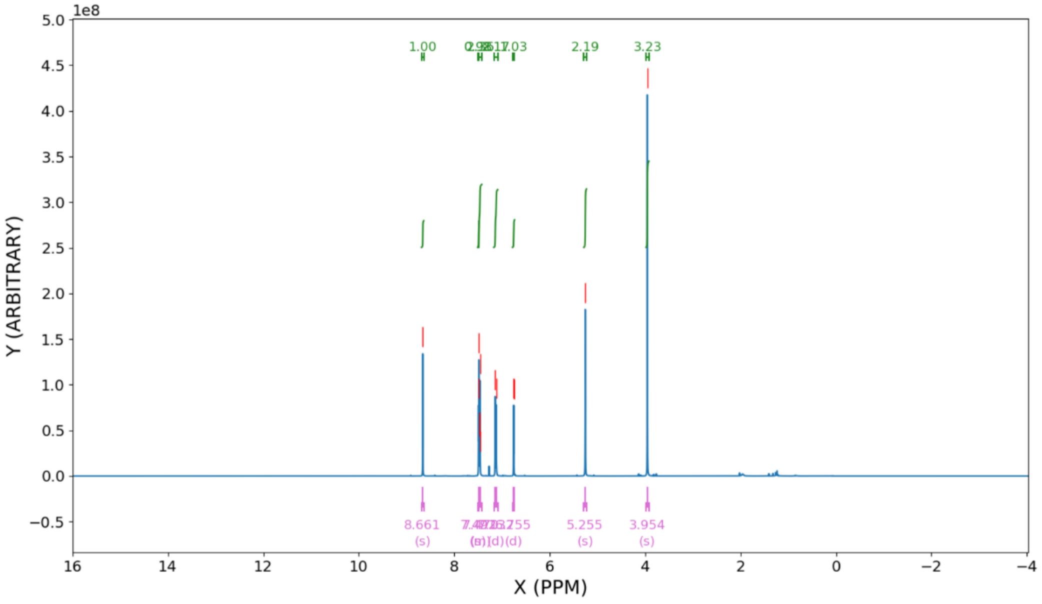

CHMO:0000595 | ^{13}C nuclear magnetic resonance spectroscopy (^{13}C NMR)

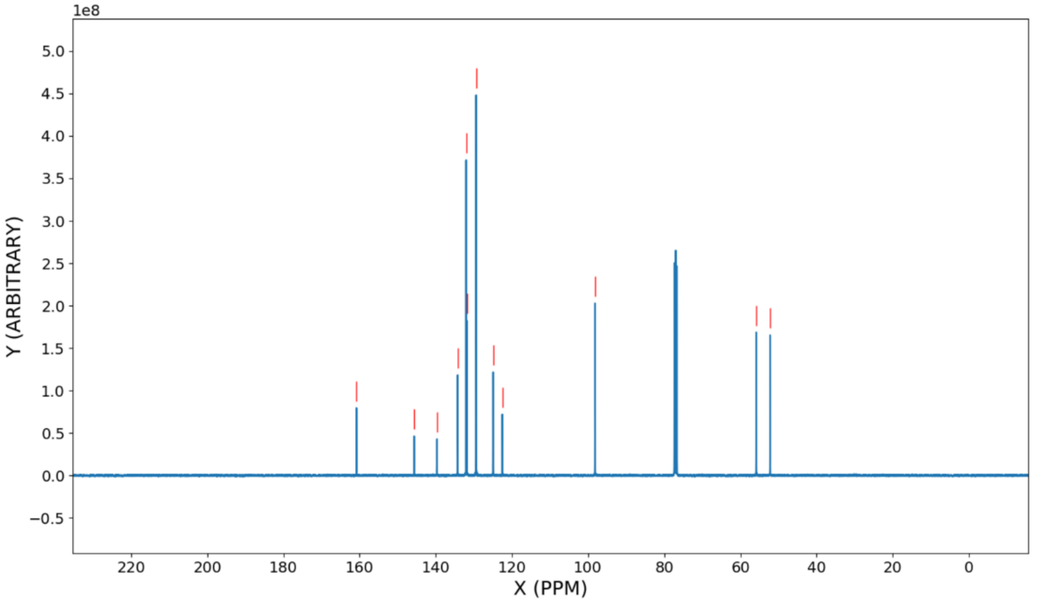

[**21de**] 1-(1-(4-bromobenzyl)-1*H*-pyrazol-3-yl)-4-(4-methoxyphenyl)-1*H*-1,2,3-triazole

CHMO:0000593 | ^{1}H nuclear magnetic resonance spectroscopy (^{1}H NMR)

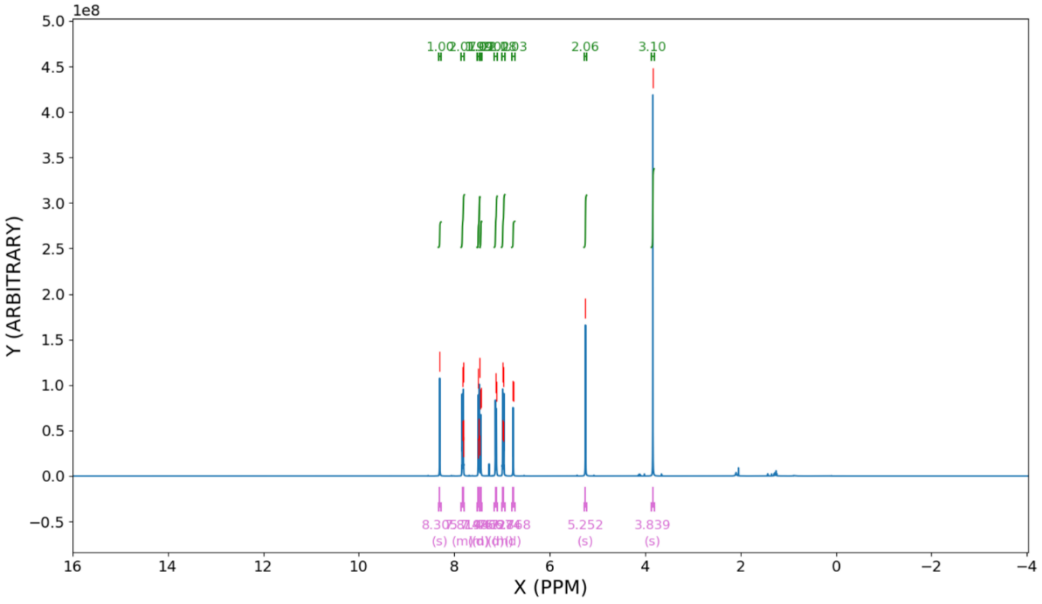

CHMO:0000595 | ^{13}C nuclear magnetic resonance spectroscopy (^{13}C NMR)

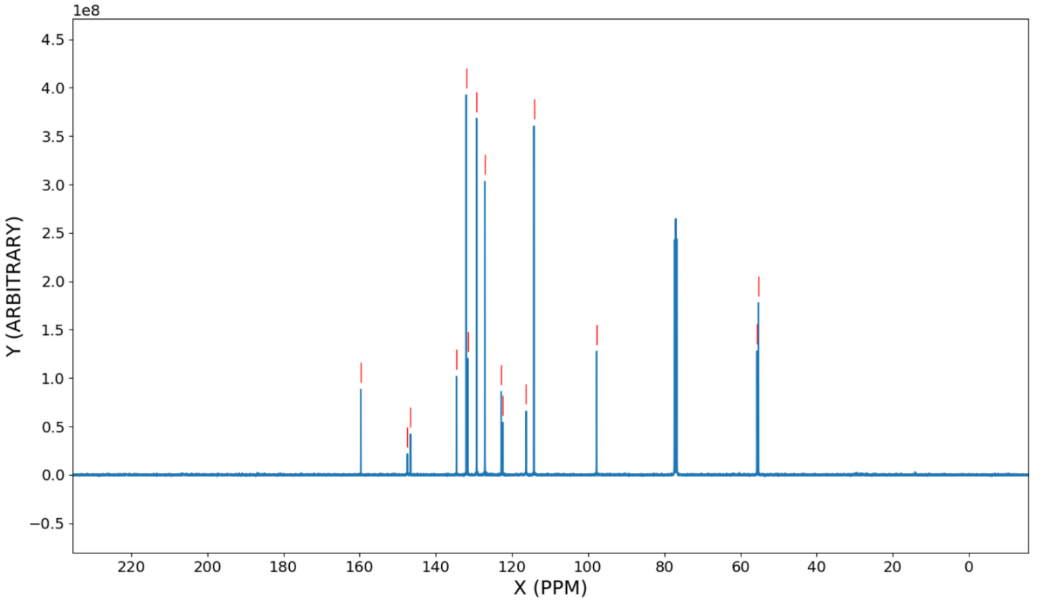

[**21ea**] 4-butyl-1-(5-methyl-1*H*-pyrazol-3-yl)-1*H*-1,2,3-triazole

CHMO:0000593 | ^{1}H nuclear magnetic resonance spectroscopy (^{1}H NMR)

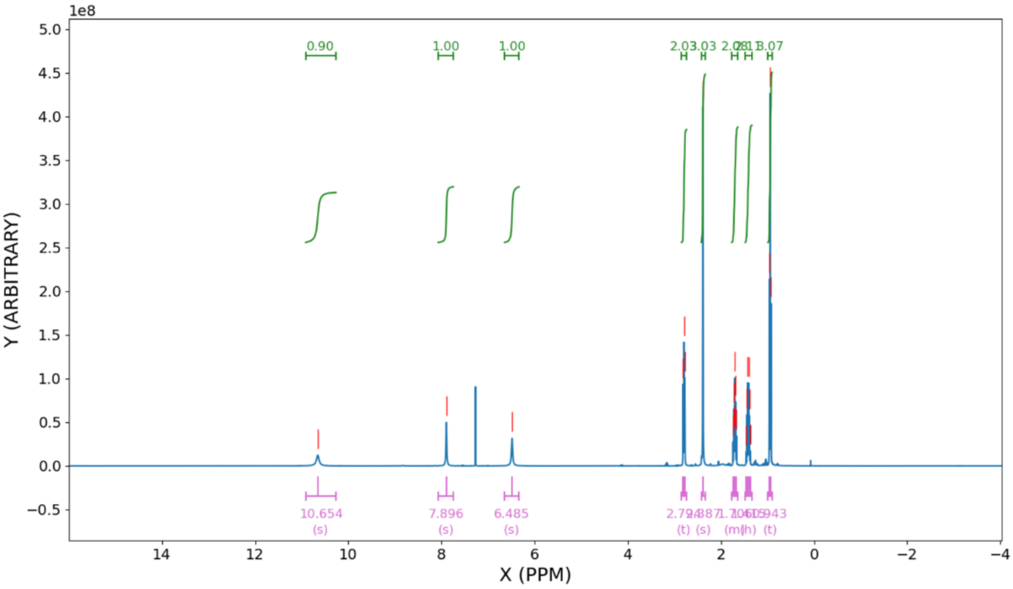

CHMO:0000595 | ^{13}C nuclear magnetic resonance spectroscopy (^{13}C NMR)

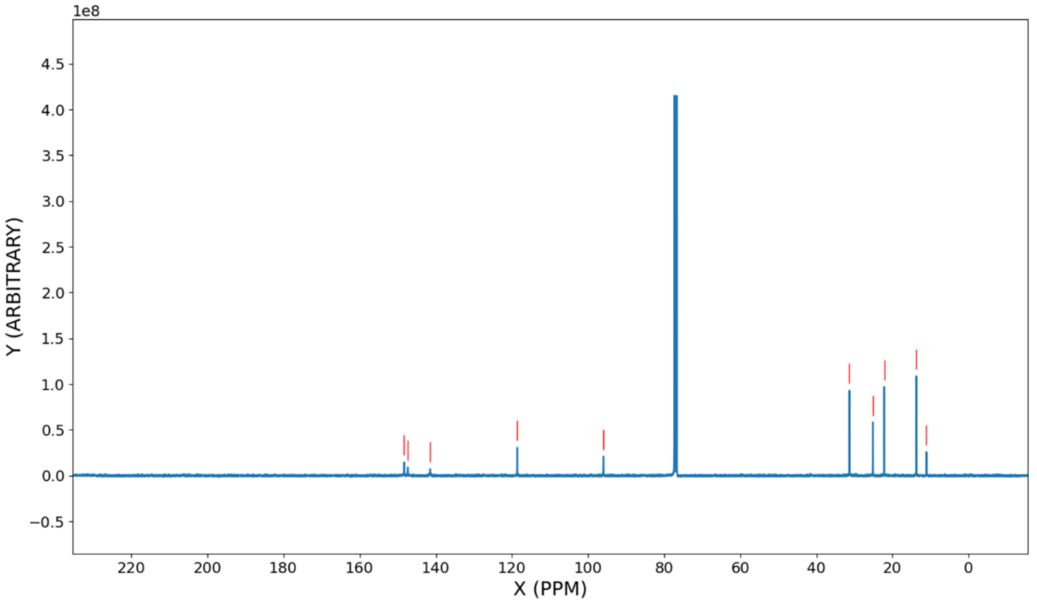

[**21eb**] methyl 1-(5-methyl-1*H*-pyrazol-3-yl)triazole-4-carboxylate

CHMO:0000593 | ^{1}H nuclear magnetic resonance spectroscopy (^{1}H NMR)

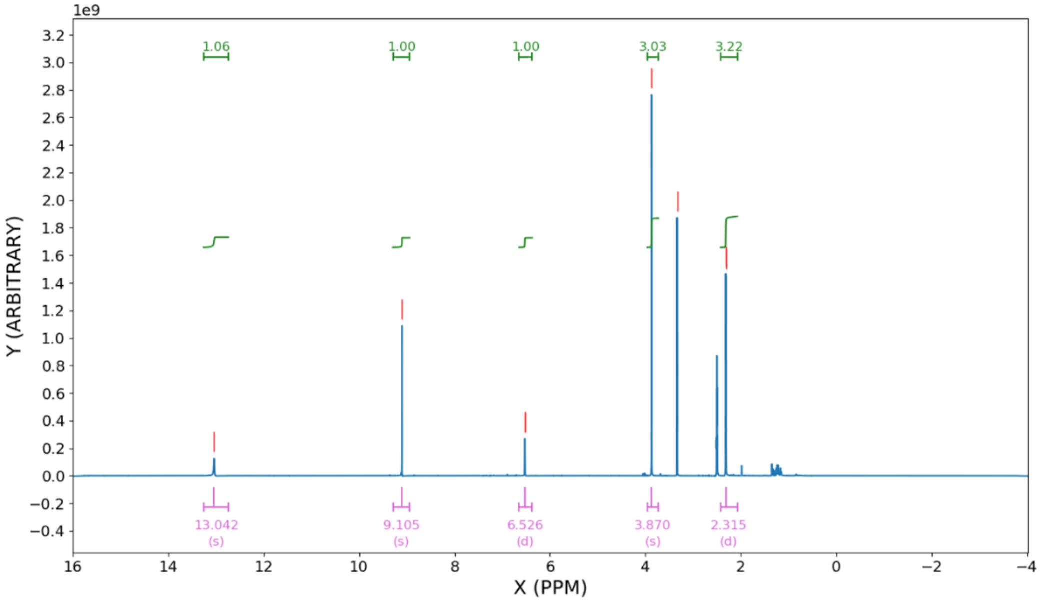

CHMO:0000595 | ^{13}C nuclear magnetic resonance spectroscopy (^{13}C NMR)

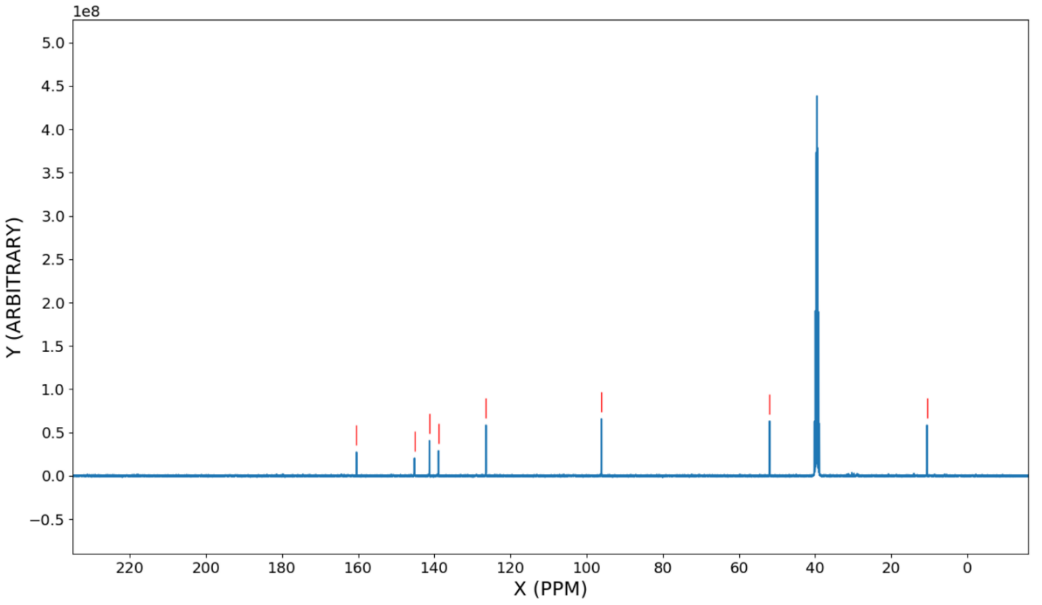

[**21ec**] (1-(5-methyl-1*H*-pyrazol-3-yl)-1*H*-1,2,3-triazol-4-yl)methyl acetate

CHMO:0000593 | ^{1}H nuclear magnetic resonance spectroscopy (^{1}H NMR)

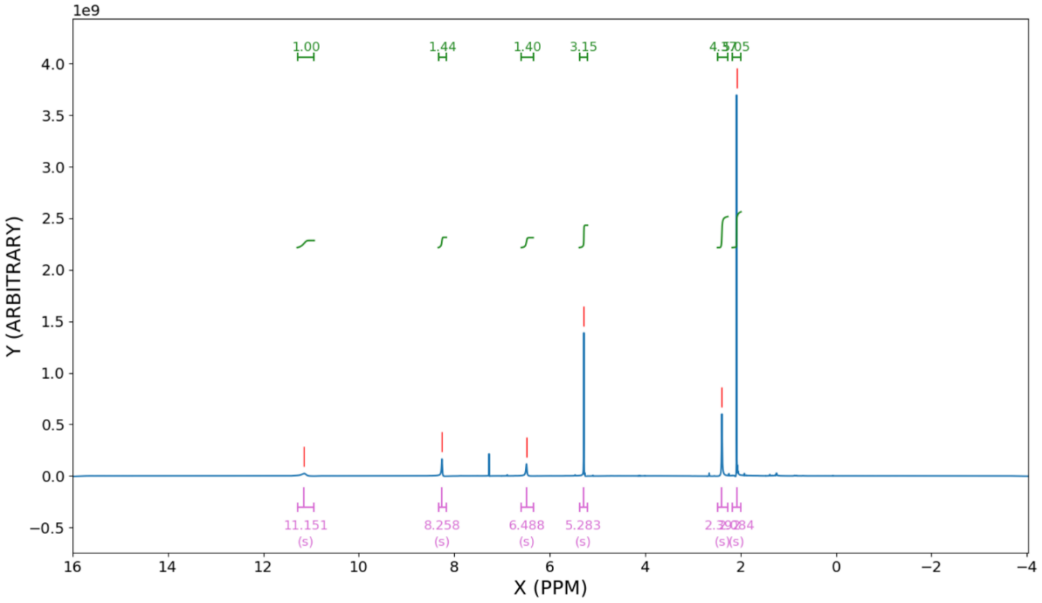

CHMO:0000595 | ^{13}C nuclear magnetic resonance spectroscopy (^{13}C NMR)

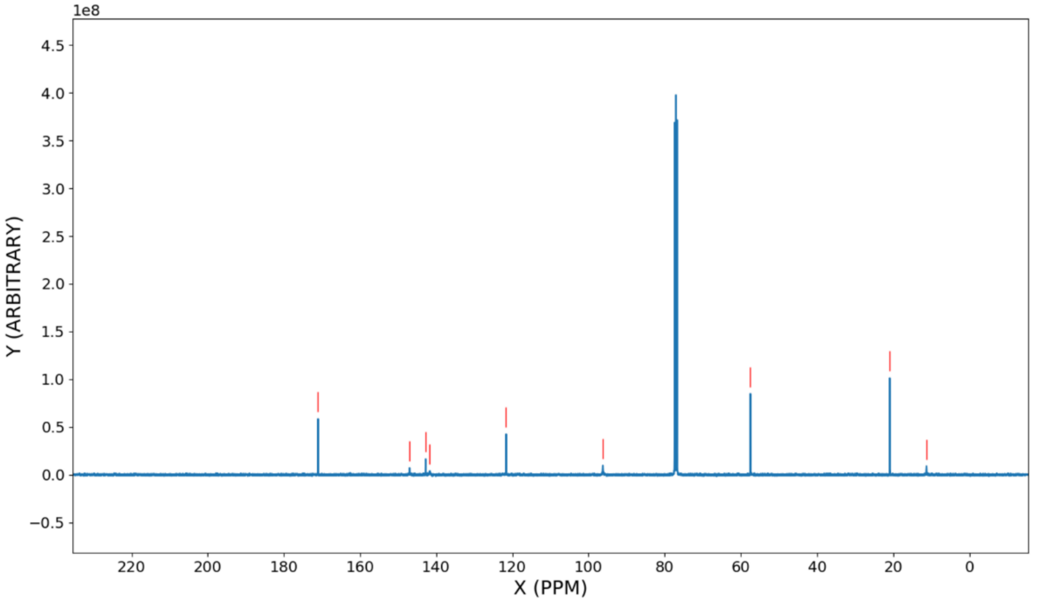

[**21ed**] 1-(5-methyl-1*H*-pyrazol-3-yl)-4-phenyl-1*H*-1,2,3-triazole

CHMO:0000593 | ^{1}H nuclear magnetic resonance spectroscopy (^{1}H NMR)

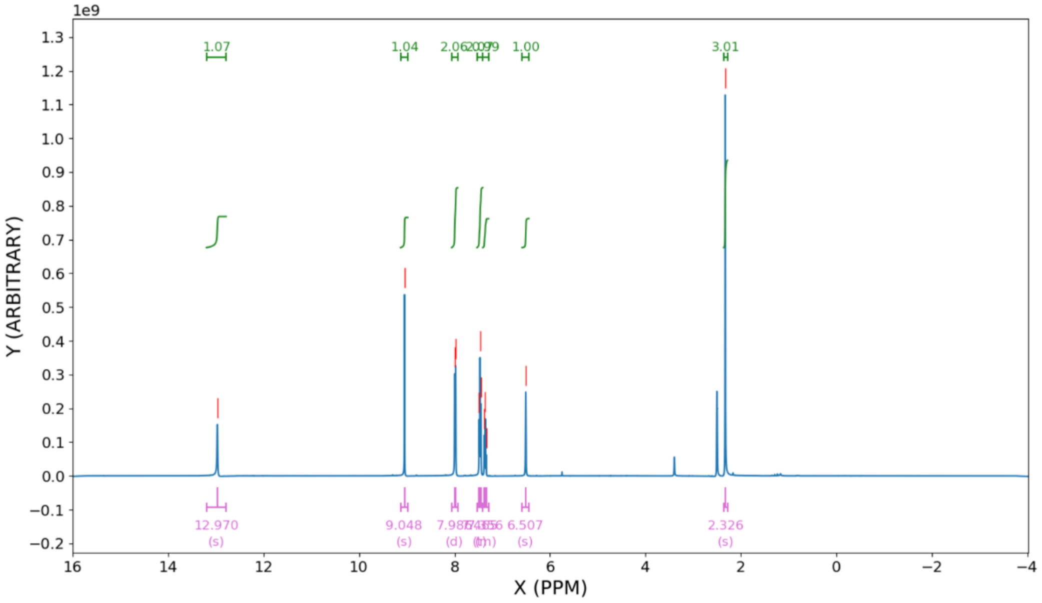

CHMO:0000595 | ^{13}C nuclear magnetic resonance spectroscopy (^{13}C NMR)

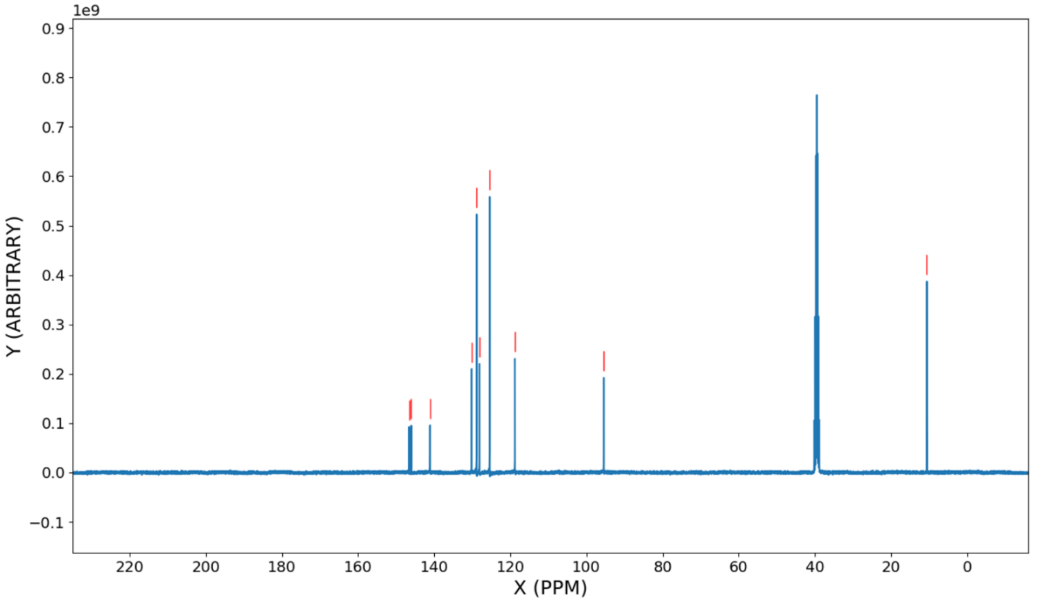

[**21ee**] 4-(4-Methoxyphenyl)-1-(5-methyl-1*H*-pyrazol-3-yl)-1*H*-1,2,3-triazole

CHMO:0000593 | ^{1}H nuclear magnetic resonance spectroscopy (^{1}H NMR)

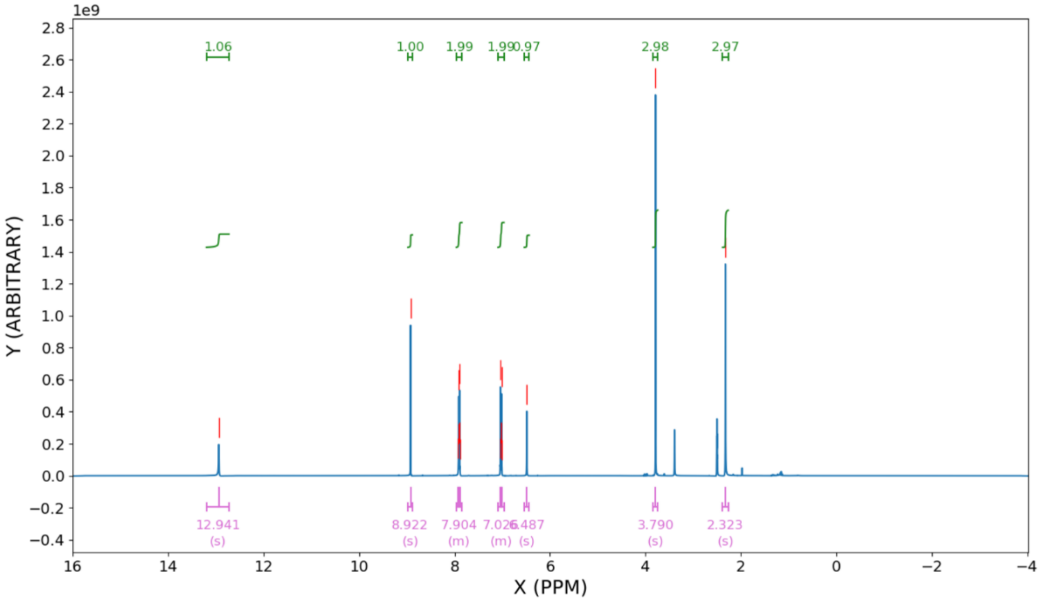

CHMO:0000595 | ^{13}C nuclear magnetic resonance spectroscopy (^{13}C NMR)

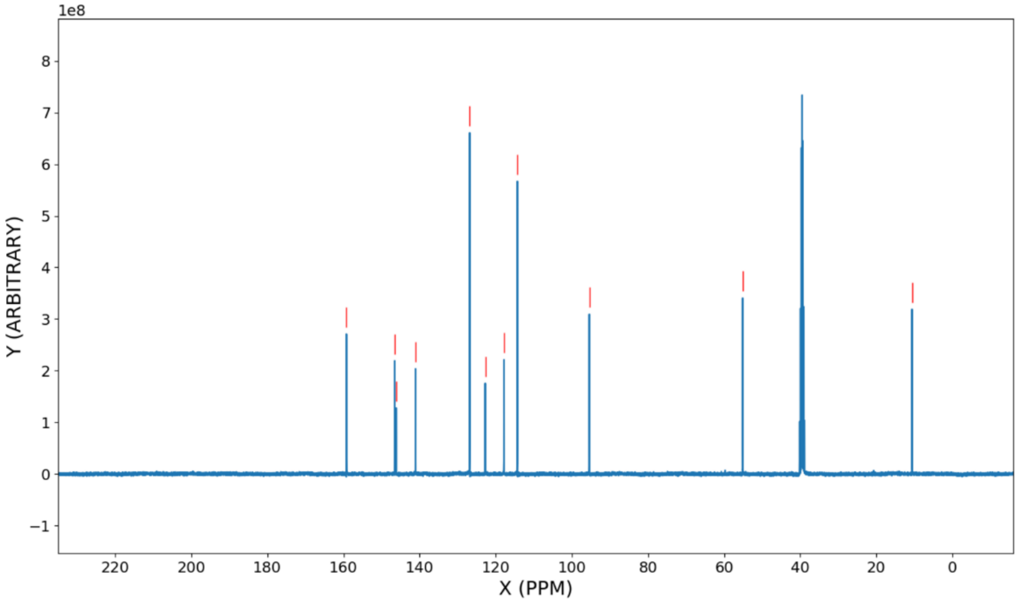

[**21ef**] methyl 4-(1-(5-methyl-1*H*-pyrazol-3-yl)-1*H*-1,2,3-triazol-4-yl)benzoate

CHMO:0000593 | ^{1}H nuclear magnetic resonance spectroscopy (^{1}H NMR)

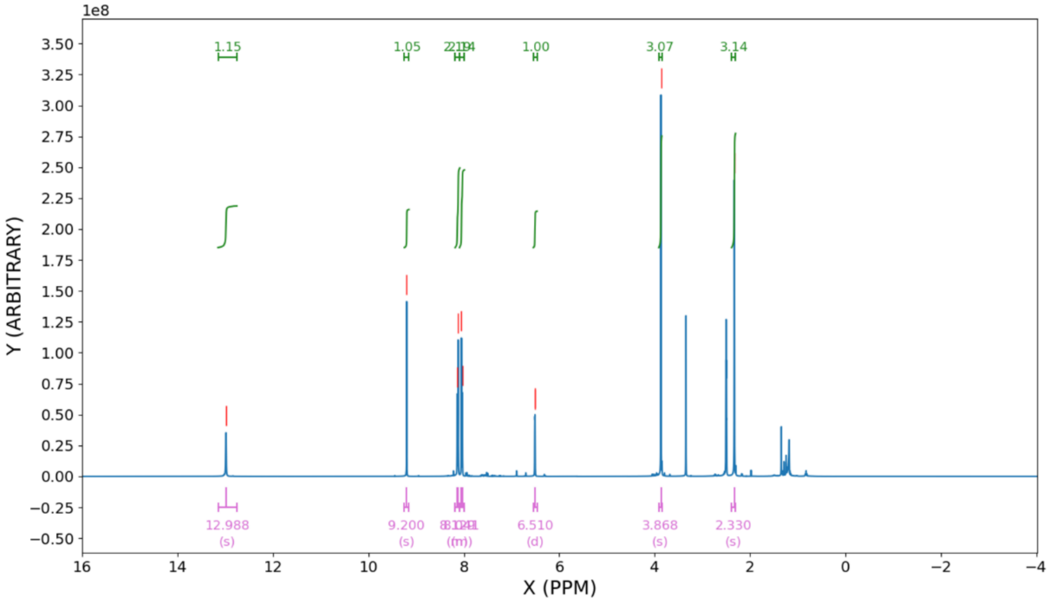

CHMO:0000595 | ^{13}C nuclear magnetic resonance spectroscopy (^{13}C NMR)

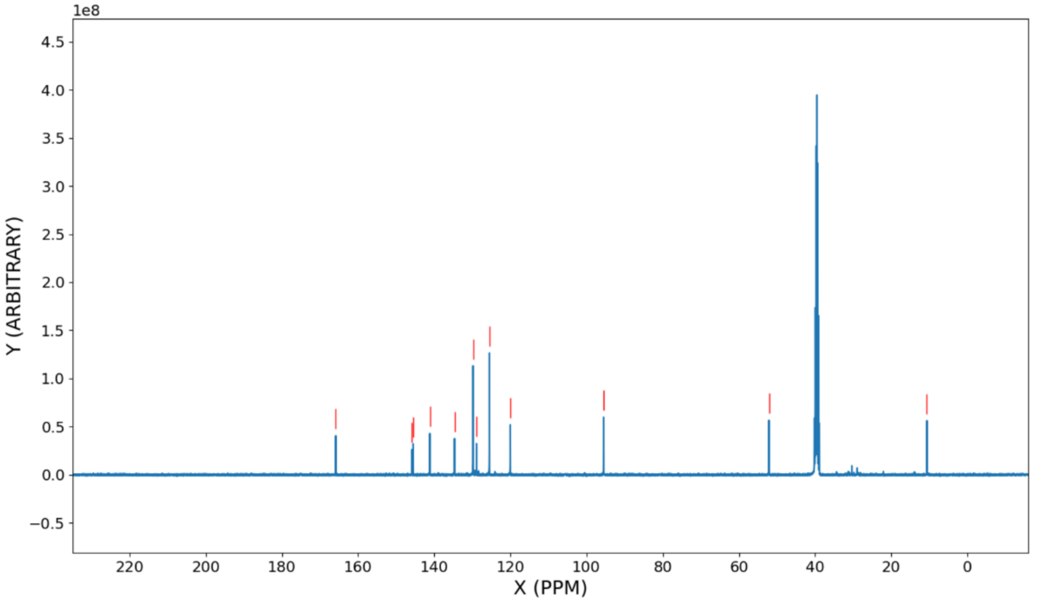

[**21fa**] 1-(1-benzyl-5-methyl-1*H*-pyrazol-3-yl)-4-butyl-1*H*-1,2,3-triazole

CHMO:0000593 | ^{1}H nuclear magnetic resonance spectroscopy (^{1}H NMR)

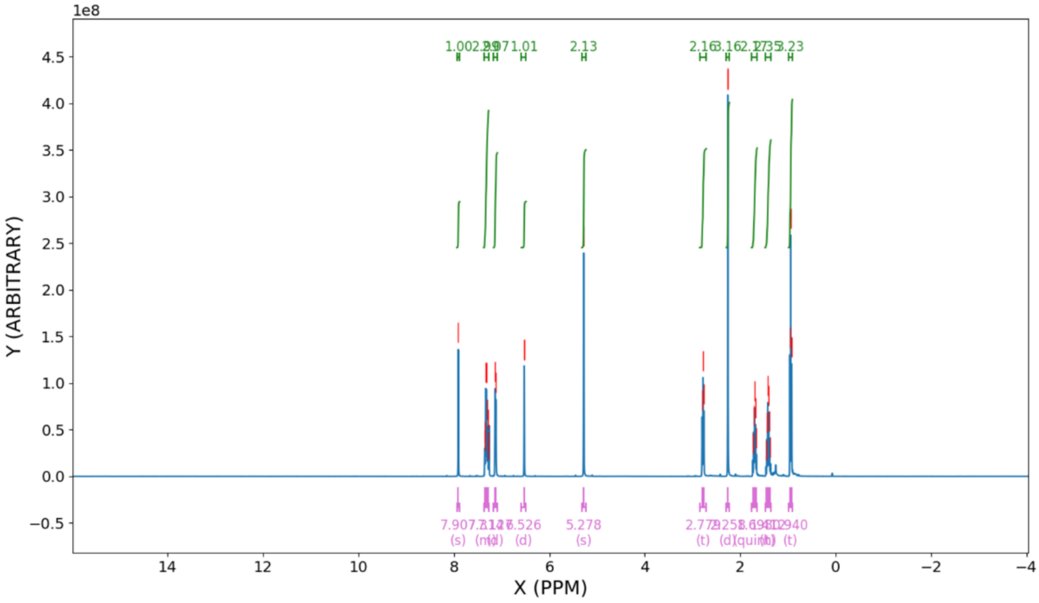

CHMO:0000595 | ^{13}C nuclear magnetic resonance spectroscopy (^{13}C NMR)

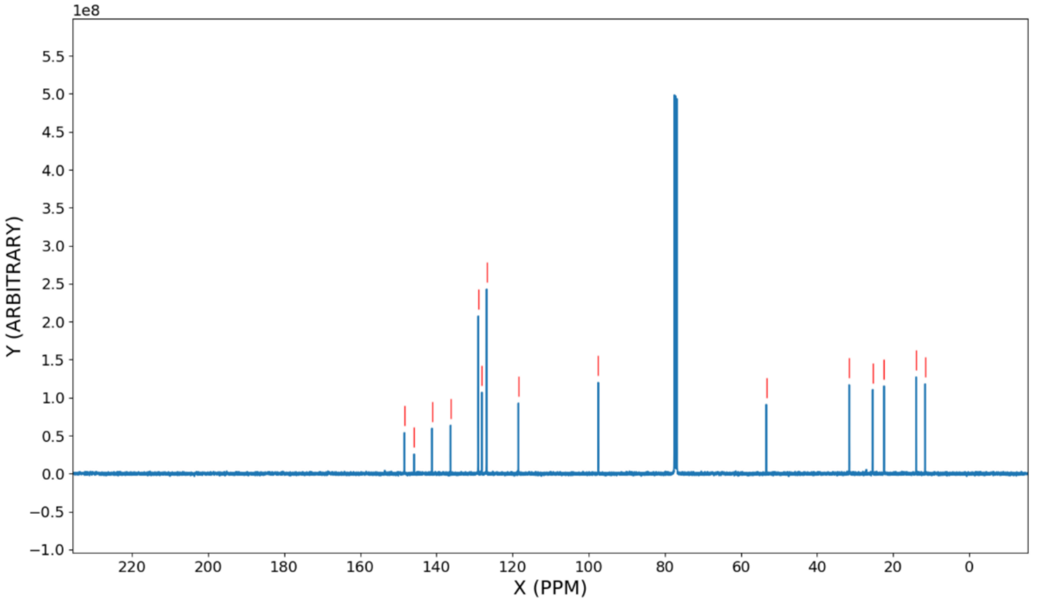

[**21fe**] 1-(1-benzyl-5-methyl-1*H*-pyrazol-3-yl)-4-(4-methoxyphenyl)-1*H*-1,2,3-triazole

CHMO:0000593 | ^{1}H nuclear magnetic resonance spectroscopy (^{1}H NMR)

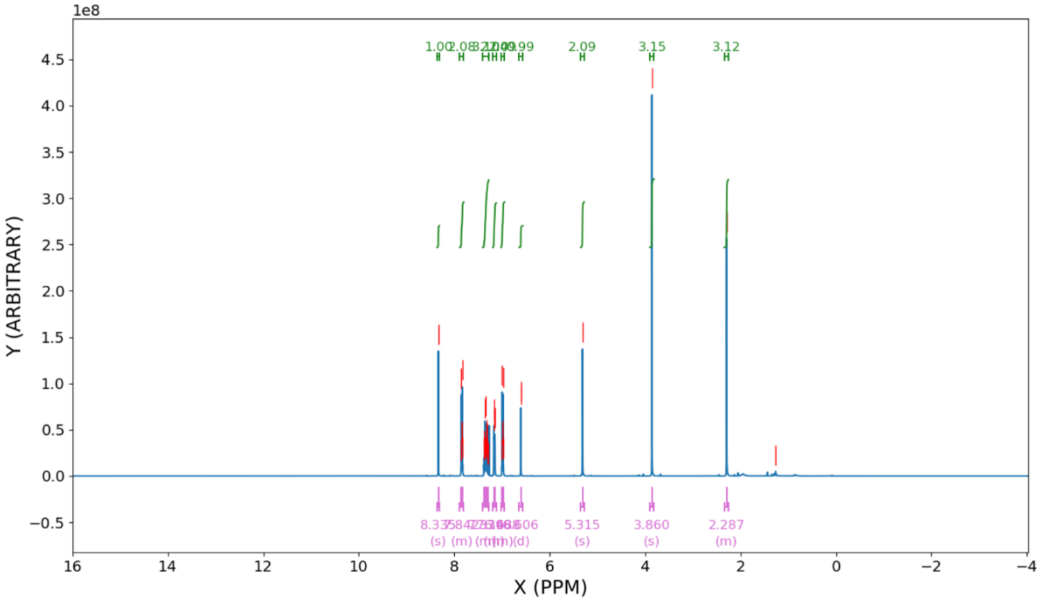

CHMO:0000595 | ^{13}C nuclear magnetic resonance spectroscopy (^{13}C NMR)

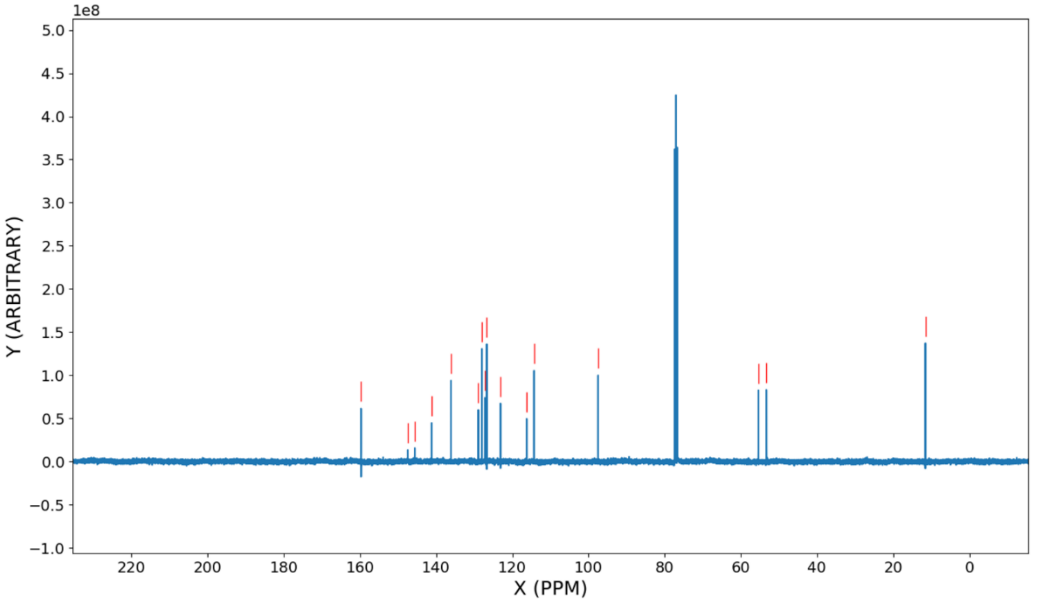

[**21ga**] 4-(3-(4-butyl-1*H*-1,2,3-triazol-1-yl)-5-methyl-1*H*-pyrazol-1-yl)benzonitrile

CHMO:0000593 | ^{1}H nuclear magnetic resonance spectroscopy (^{1}H NMR)

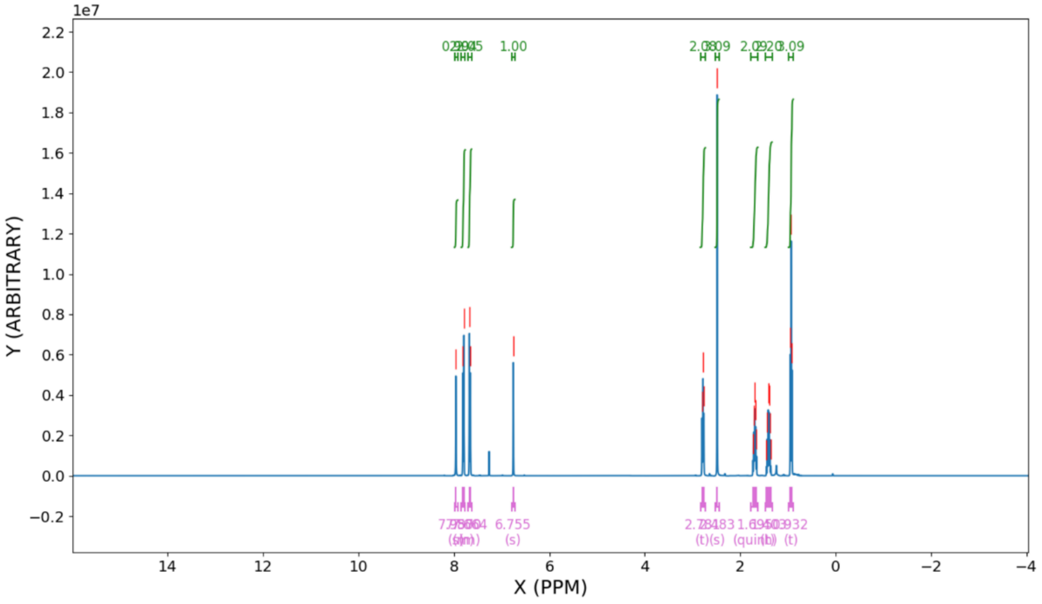

CHMO:0000595 | ^{13}C nuclear magnetic resonance spectroscopy (^{13}C NMR)

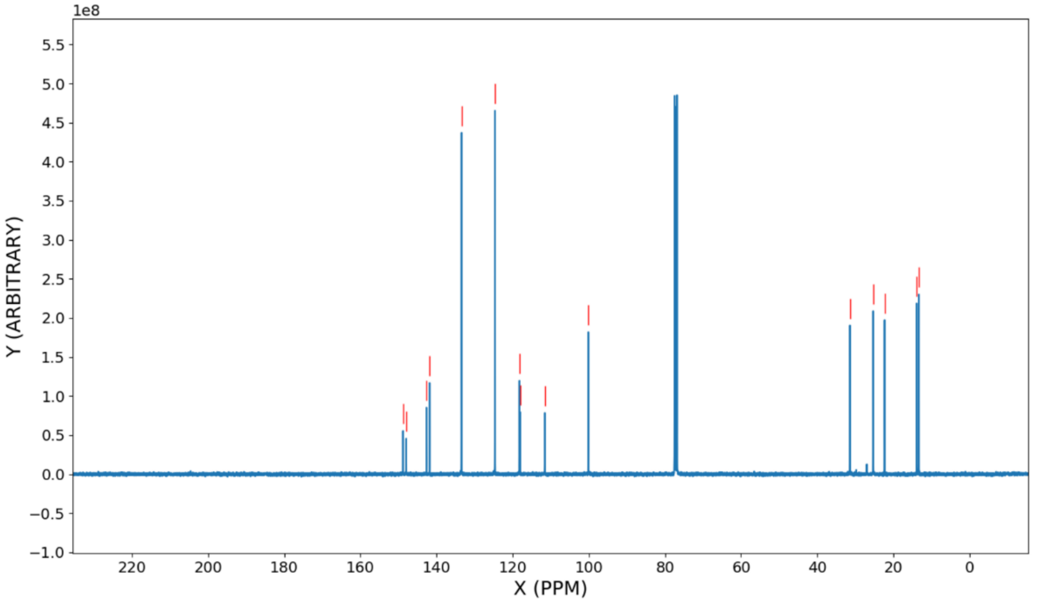

[**21gd**] 4-(5-methyl-3-(4-phenyl-1*H*-1,2,3-triazol-1-yl)-1*H*-pyrazol-1-yl)benzonitrile

CHMO:0000593 | ^{1}H nuclear magnetic resonance spectroscopy (^{1}H NMR)

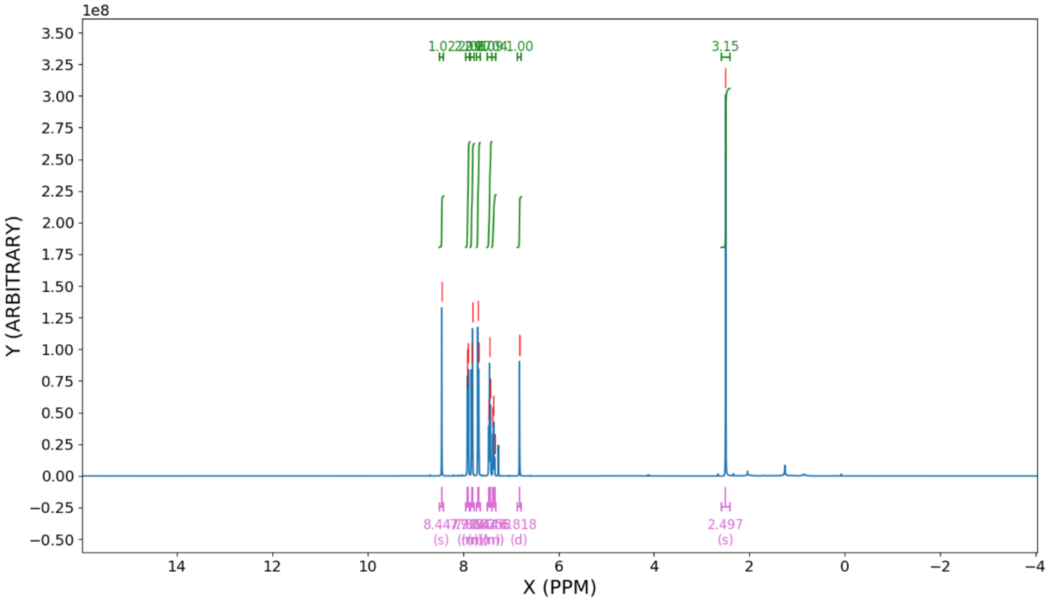

CHMO:0000595 | ^{13}C nuclear magnetic resonance spectroscopy (^{13}C NMR)

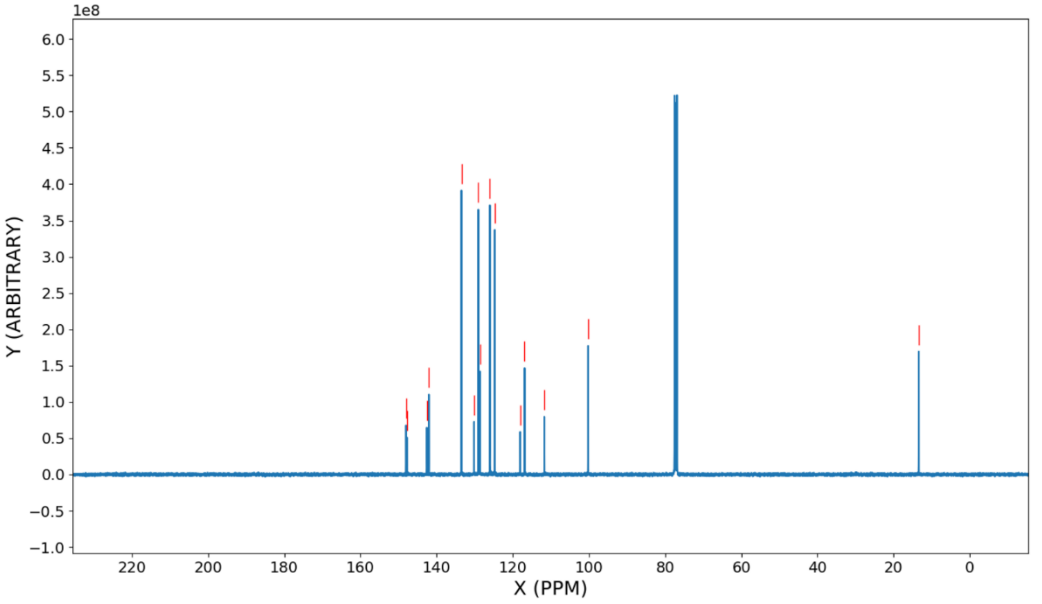

[**21gh**] 4-(3-(4-((1,3-dioxoisoindolin-2-yl)methyl)-1*H*-1,2,3-triazol-1-yl)-5-methyl-1*H*-pyrazol-1-yl)benzonitrile

CHMO:0000593 | ^{1}H nuclear magnetic resonance spectroscopy (^{1}H NMR)

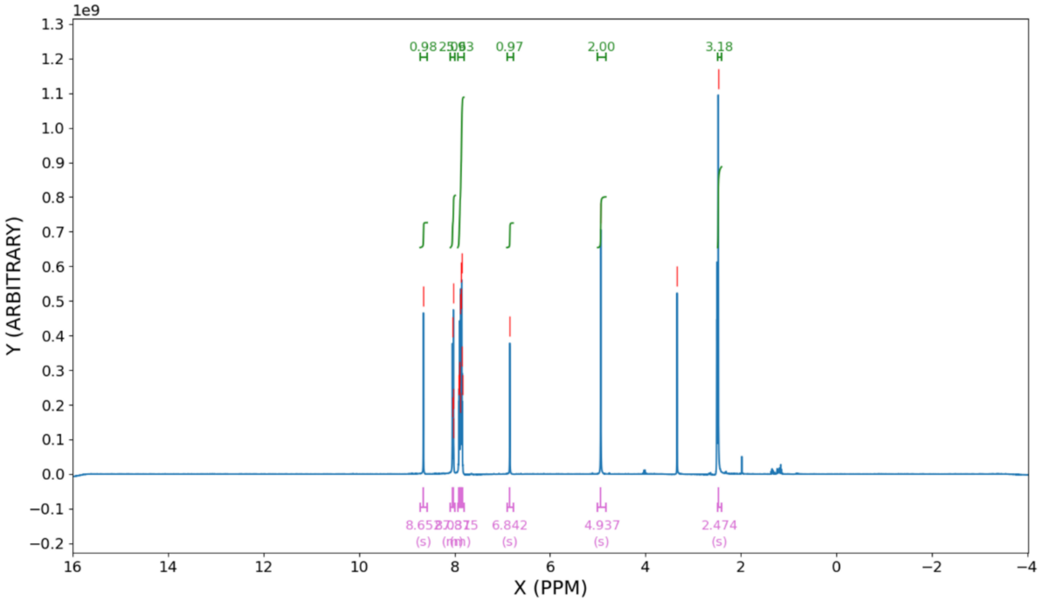

CHMO:0000595 | ^{13}C nuclear magnetic resonance spectroscopy (^{13}C NMR)

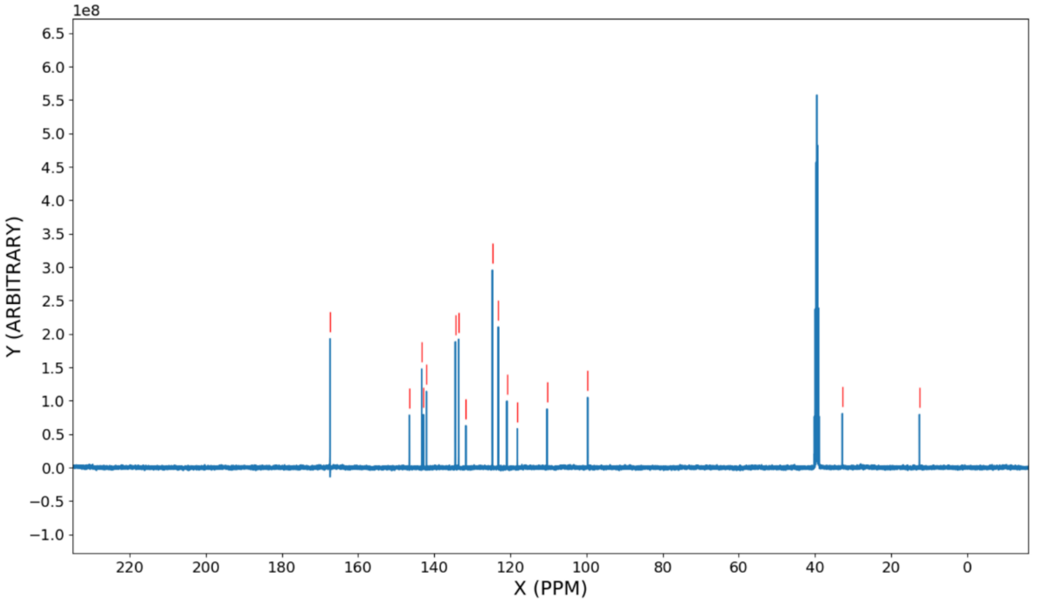

[**21hc**] (1-(5-methyl-1-(4-nitrophenyl)-1*H*-pyrazol-3-yl)-1*H*-1,2,3-triazol-4-yl)methyl acetate

CHMO:0000593 | ^{1}H nuclear magnetic resonance spectroscopy (^{1}H NMR)

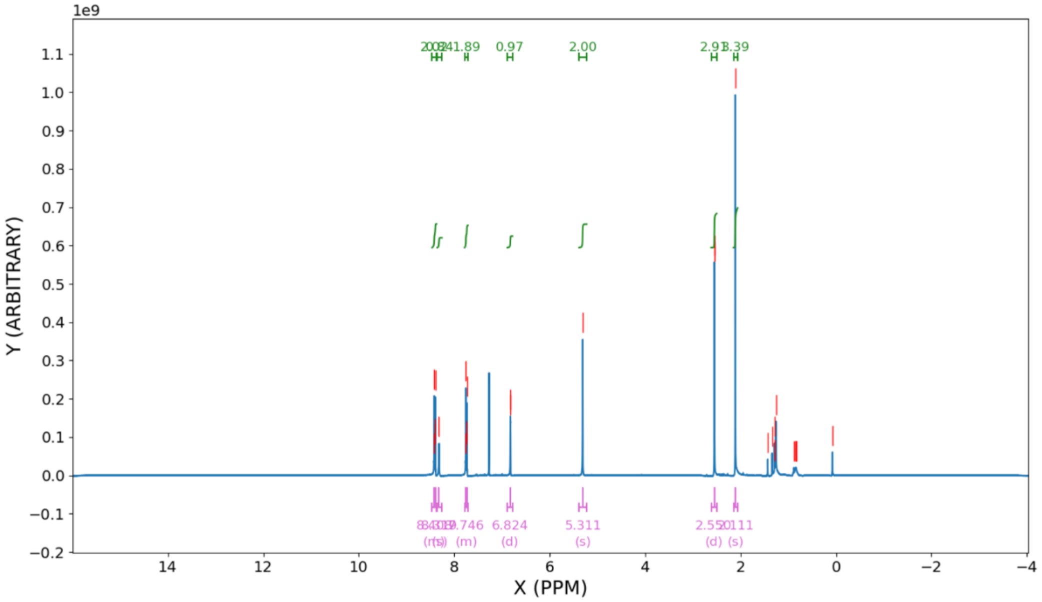

CHMO:0000595 | ^{13}C nuclear magnetic resonance spectroscopy (^{13}C NMR)

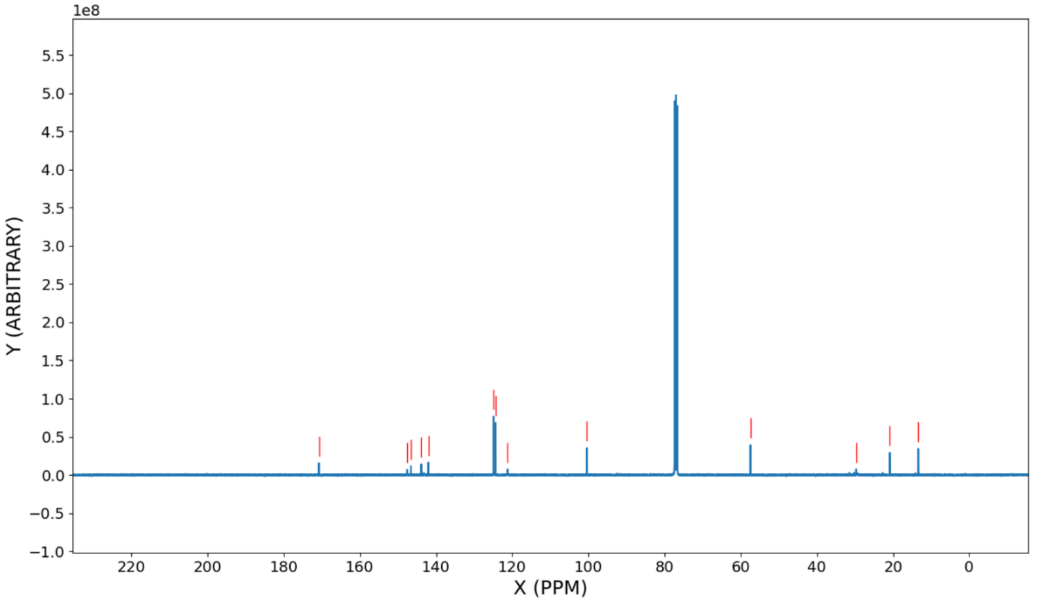

[**21hd**] 1-(5-methyl-1-(4-nitrophenyl)-1*H*-pyrazol-3-yl)-4-phenyl-1*H*-1,2,3-triazole

CHMO:0000593 | ^{1}H nuclear magnetic resonance spectroscopy (^{1}H NMR)

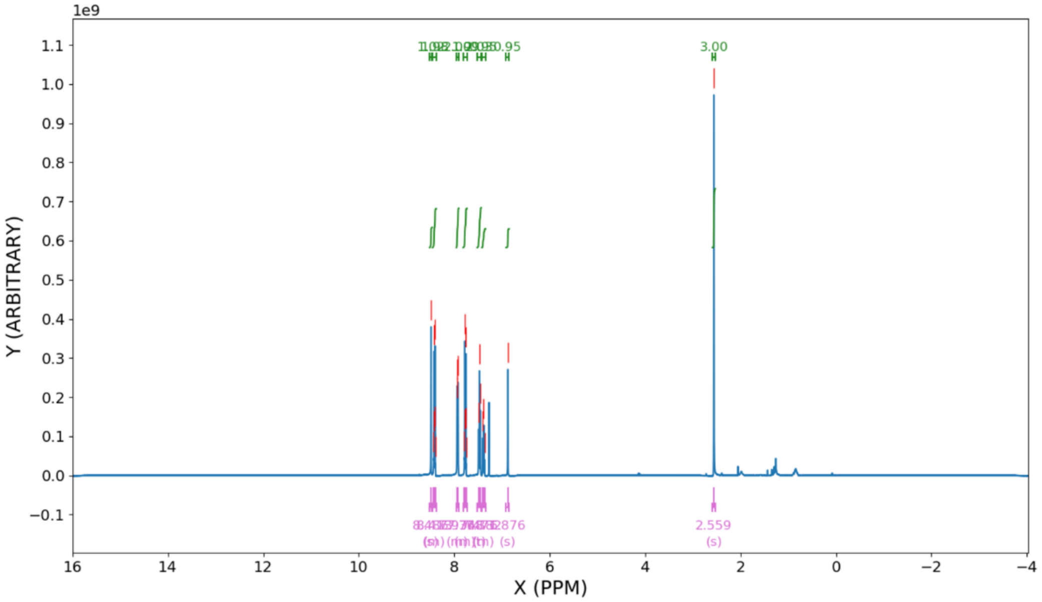

CHMO:0000595 | ^{13}C nuclear magnetic resonance spectroscopy (^{13}C NMR)

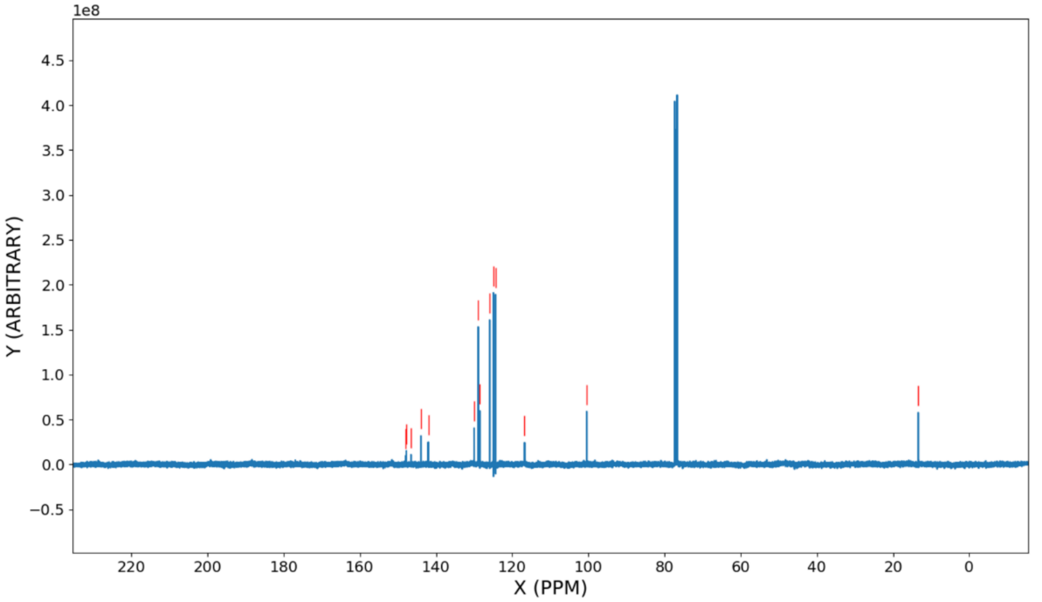

[**21he**] 4-(4-methoxyphenyl)-1-(5-methyl-1-(4-nitrophenyl)-1*H*-pyrazol-3-yl)-1*H*-1,2,3-triazole

CHMO:0000593 | ^{1}H nuclear magnetic resonance spectroscopy (^{1}H NMR)

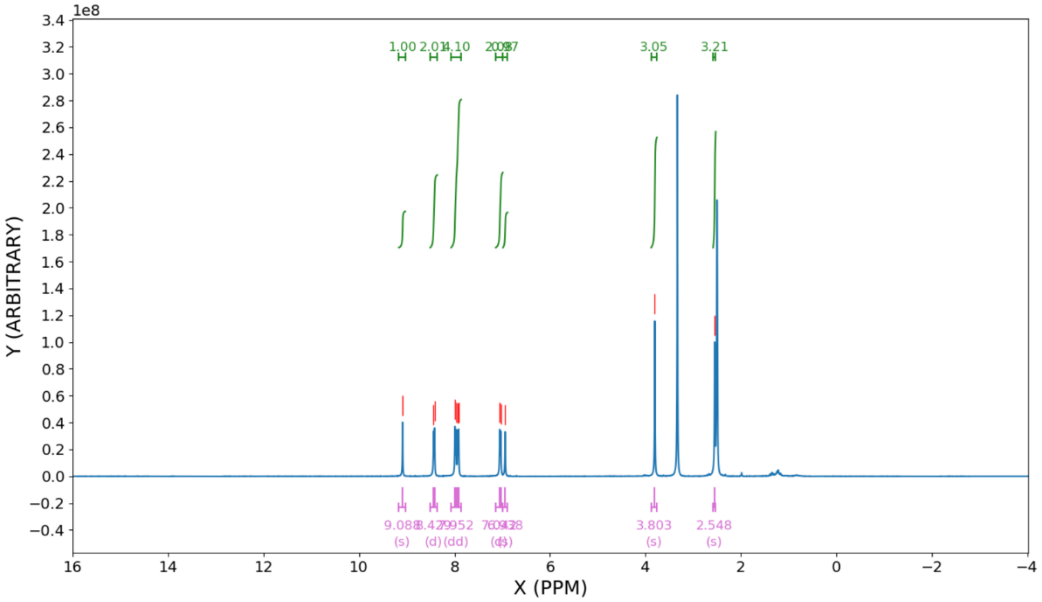

CHMO:0000595 | ^{13}C nuclear magnetic resonance spectroscopy (^{13}C NMR)

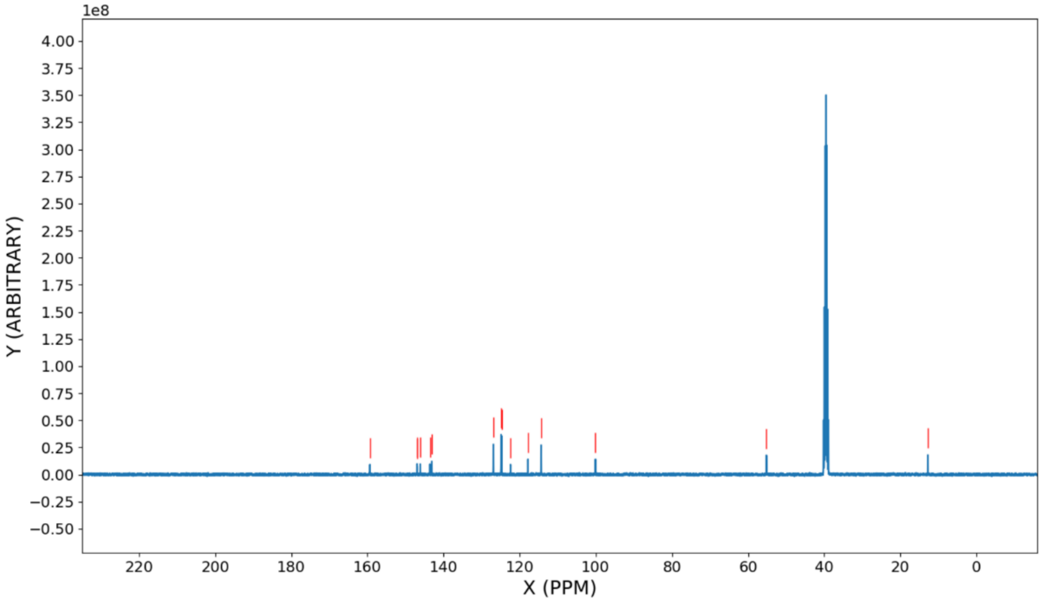

[**21ia**] 4-butyl-1-(1-(2-chloro-4-nitrophenyl)-5-methyl-1*H*-pyrazol-3-yl)-1*H*-1,2,3-triazole

CHMO:0000593 | ^{1}H nuclear magnetic resonance spectroscopy (^{1}H NMR)

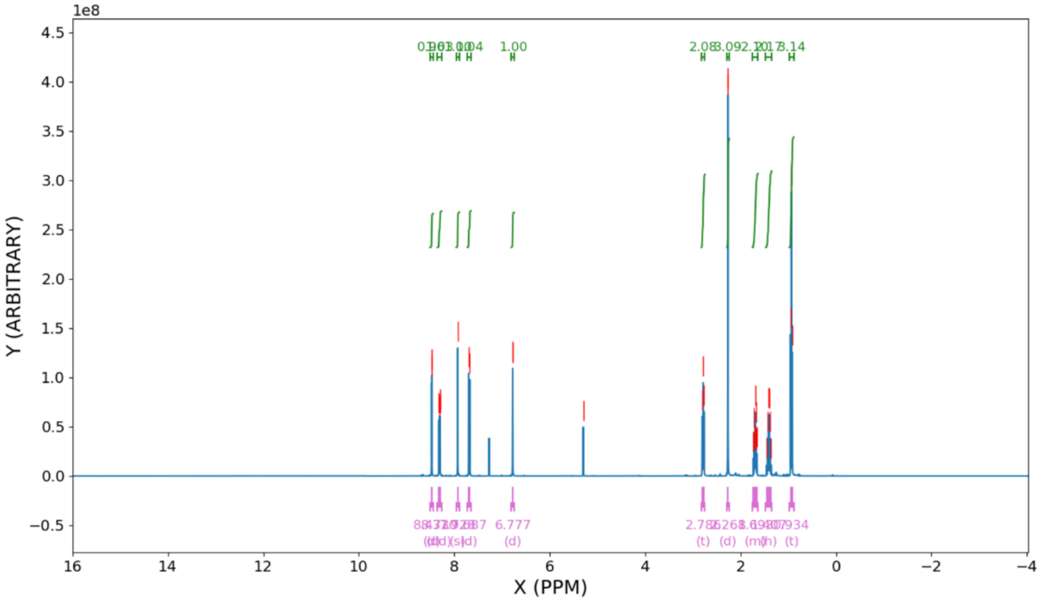

CHMO:0000595 | ^{13}C nuclear magnetic resonance spectroscopy (^{13}C NMR)

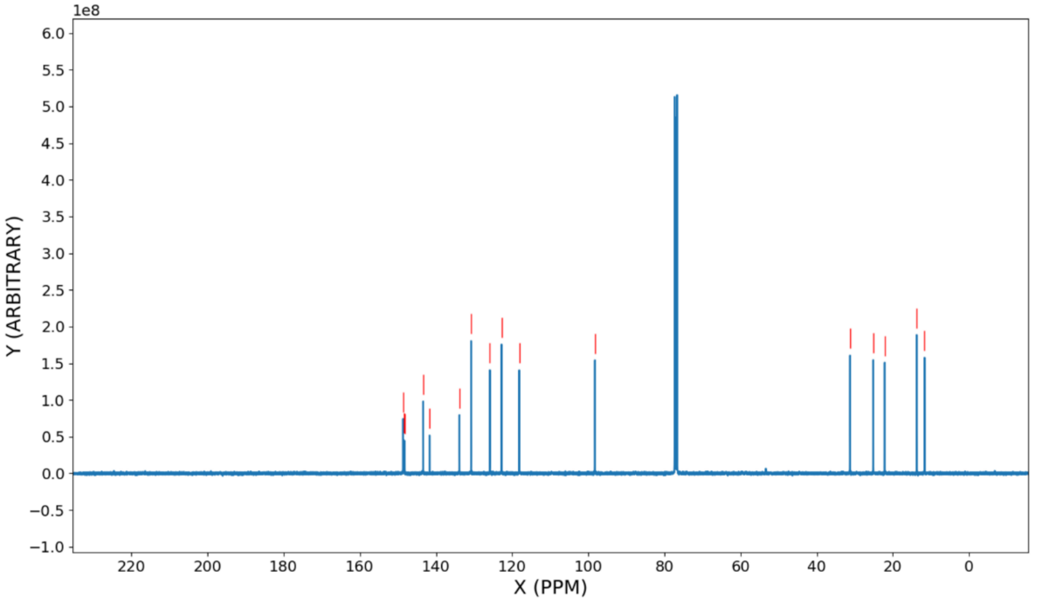

[**21ic**] (1-(1-(2-chloro-4-nitrophenyl)-5-methyl-1*H*-pyrazol-3-yl)-1*H*-1,2,3-triazol-4-yl)methyl acetate

CHMO:0000593 | ^{1}H nuclear magnetic resonance spectroscopy (^{1}H NMR)

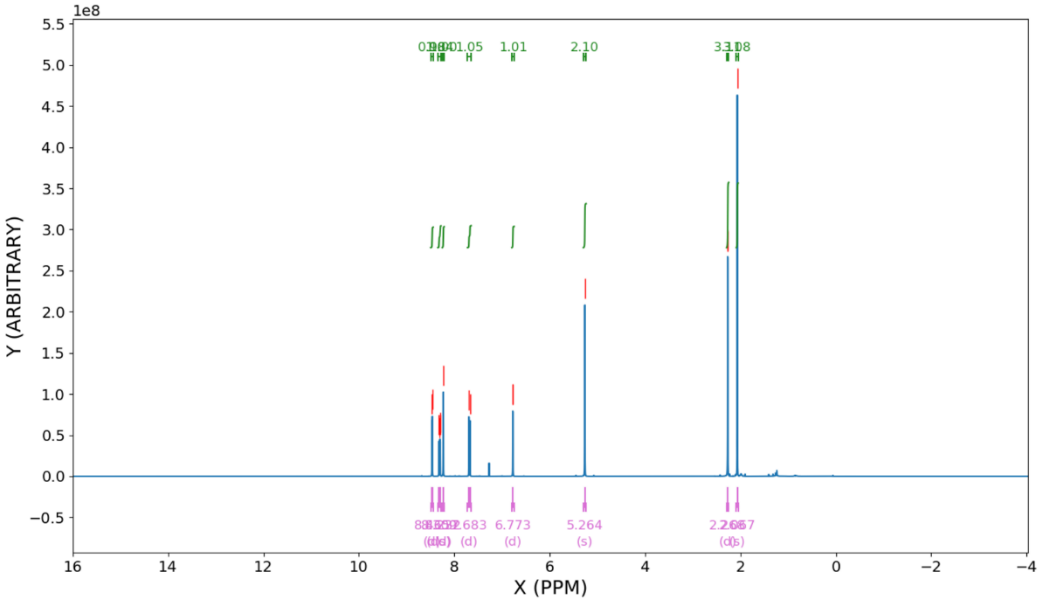

CHMO:0000595 | ^{13}C nuclear magnetic resonance spectroscopy (^{13}C NMR)

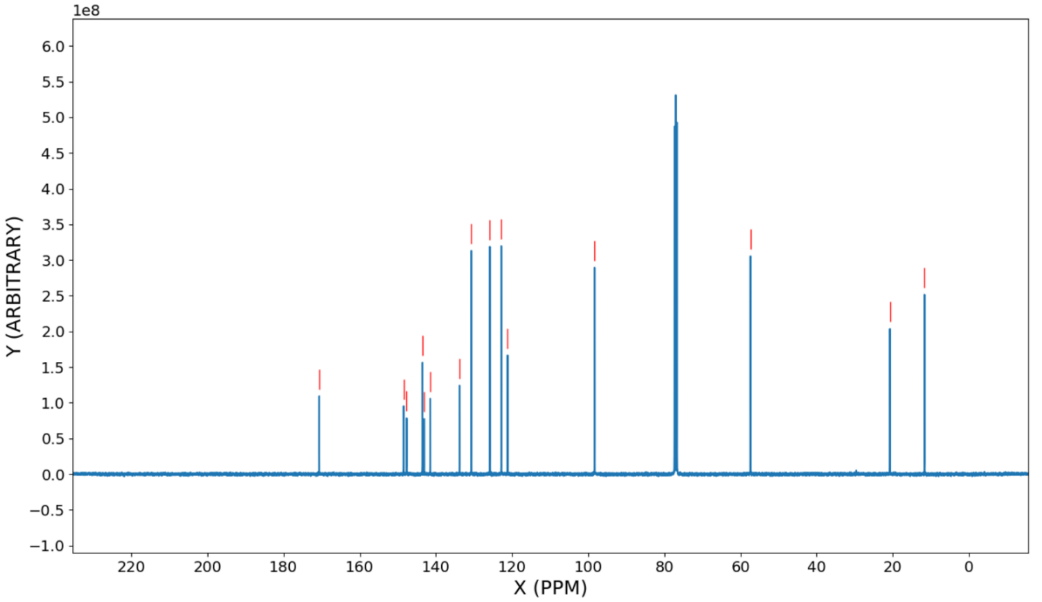

[**21id**] 1-(1-(2-chloro-4-nitrophenyl)-5-methyl-1*H*-pyrazol-3-yl)-4-phenyl-1*H*-1,2,3-triazole

CHMO:0000593 | ^{1}H nuclear magnetic resonance spectroscopy (^{1}H NMR)

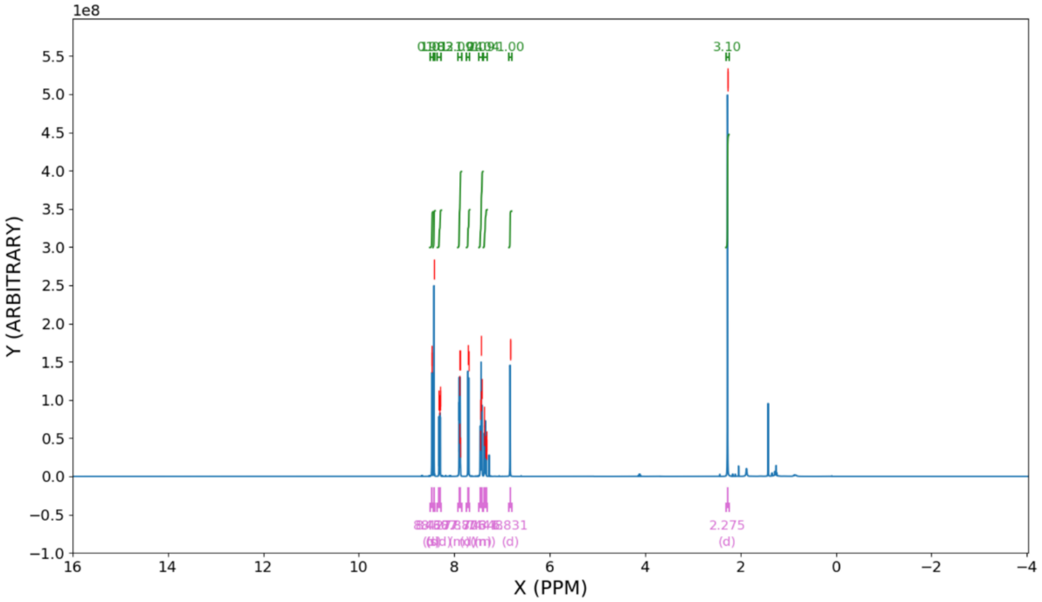

CHMO:0000595 | ^{13}C nuclear magnetic resonance spectroscopy (^{13}C NMR)

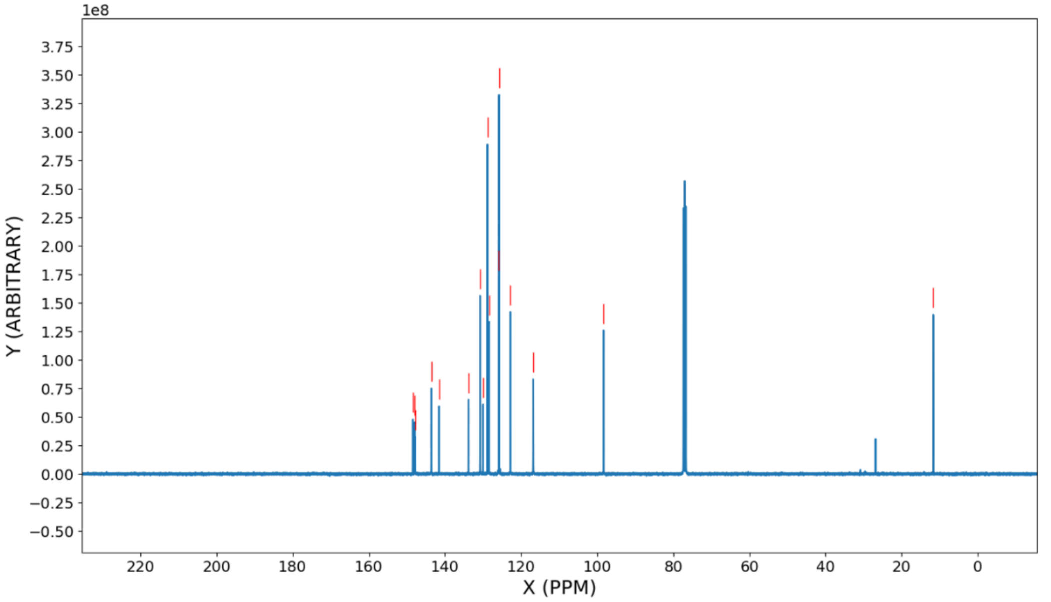

[**21ja**] ethyl 3-(4-butyl-1*H*-1,2,3-triazol-1-yl)-1*H*-pyrazole-4-carboxylate

CHMO:0000593 | ^{1}H nuclear magnetic resonance spectroscopy (^{1}H NMR)

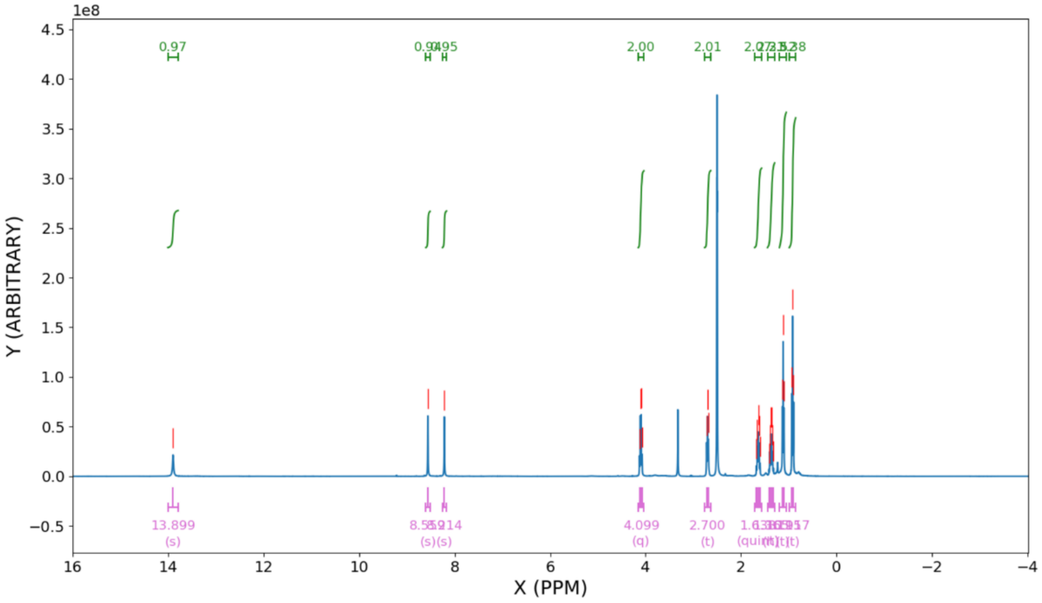

CHMO:0000595 | ^{13}C nuclear magnetic resonance spectroscopy (^{13}C NMR)

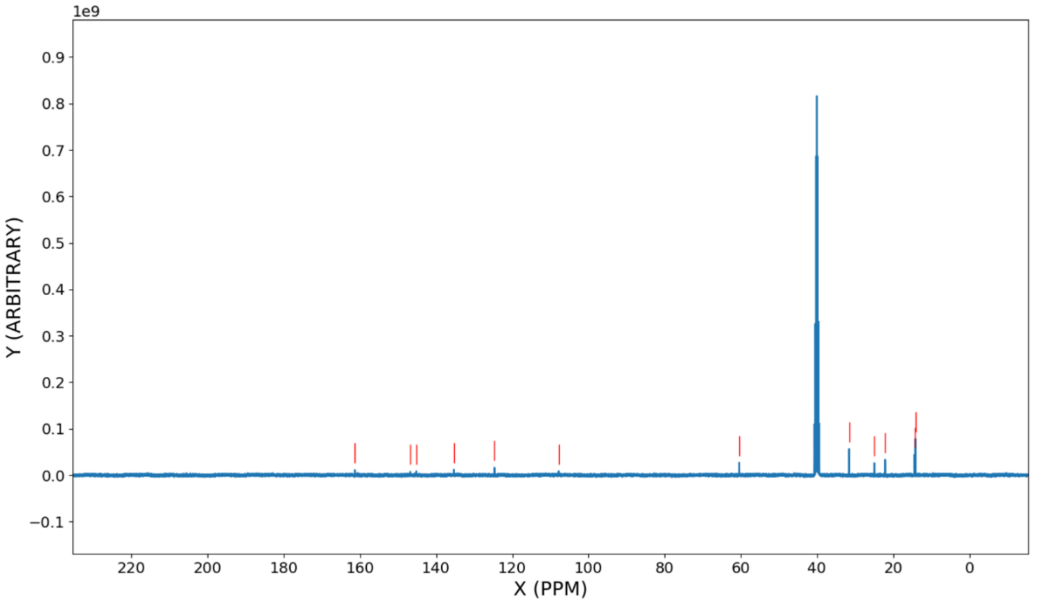

[**21jc**] ethyl 3-(4-(acetoxymethyl)-1*H*-1,2,3-triazol-1-yl)-1*H*-pyrazole-4-carboxylate

CHMO:0000593 | ^{1}H nuclear magnetic resonance spectroscopy (^{1}H NMR)

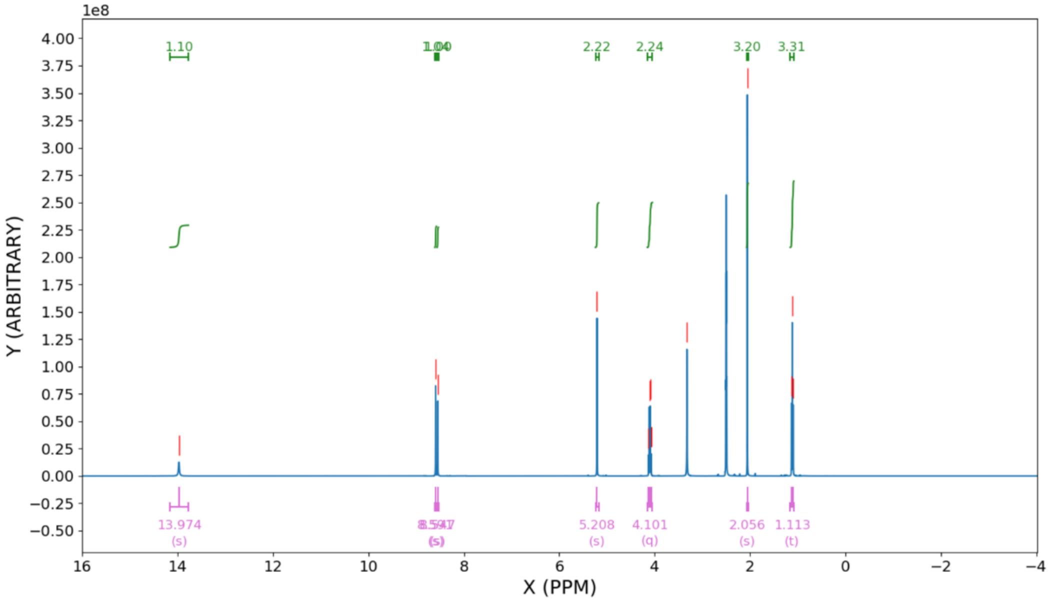

CHMO:0000595 | ^{13}C nuclear magnetic resonance spectroscopy (^{13}C NMR)

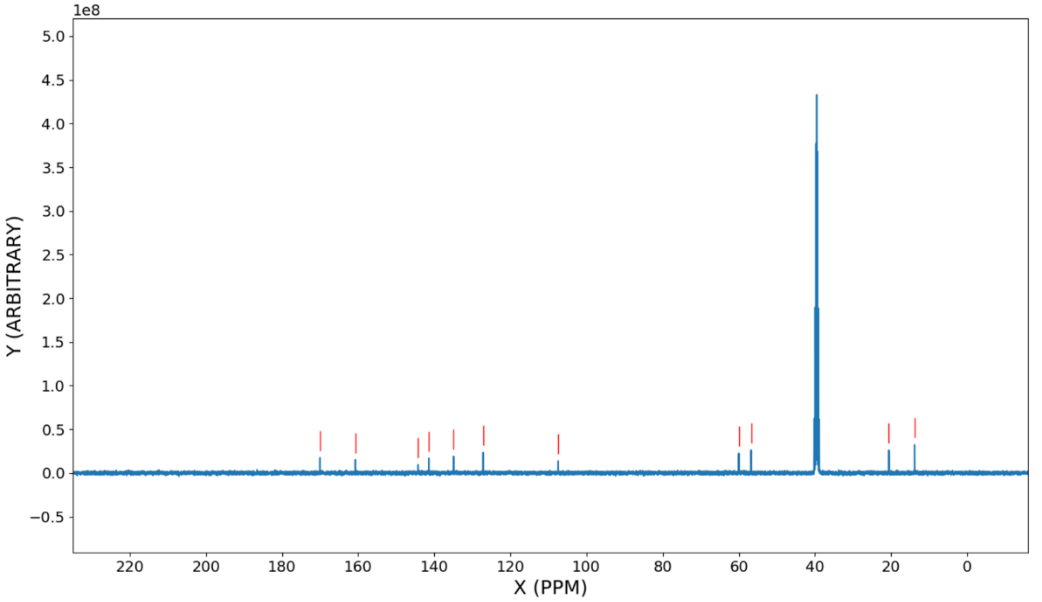

[**21jd**] ethyl 3-(4-phenyl-1*H*-1,2,3-triazol-1-yl)-1*H*-pyrazole-4-carboxylate

CHMO:0000593 | ^{1}H nuclear magnetic resonance spectroscopy (^{1}H NMR)

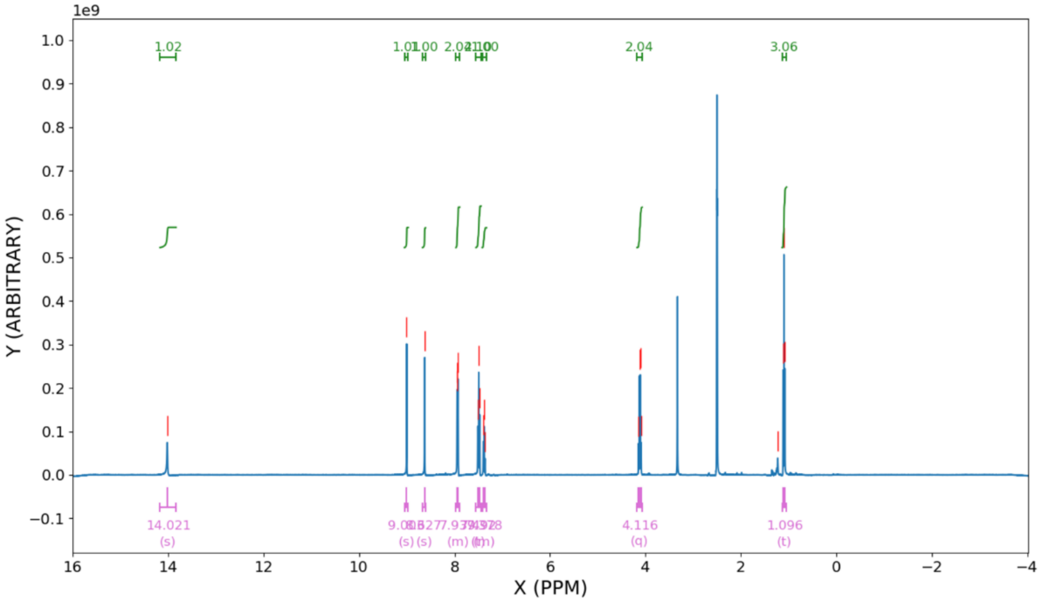

CHMO:0000595 | ^{13}C nuclear magnetic resonance spectroscopy (^{13}C NMR)

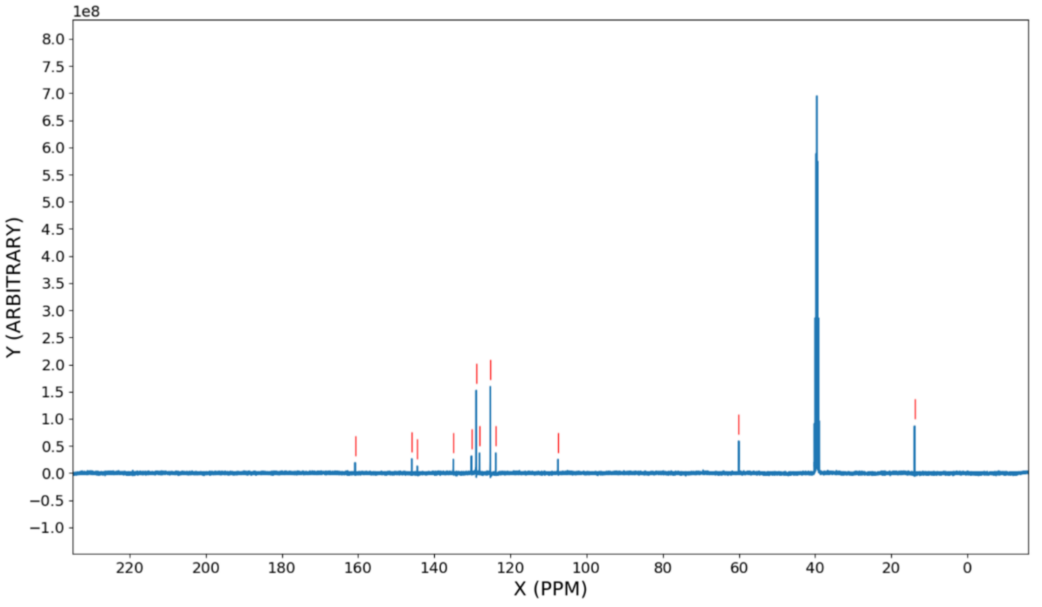

[**21jh**] ethyl 3-(4-((1,3-dioxoisoindolin-2-yl)methyl)-1*H*-1,2,3-triazol-1-yl)-1*H*-pyrazole-4-carboxylate

CHMO:0000593 | ^{1}H nuclear magnetic resonance spectroscopy (^{1}H NMR)

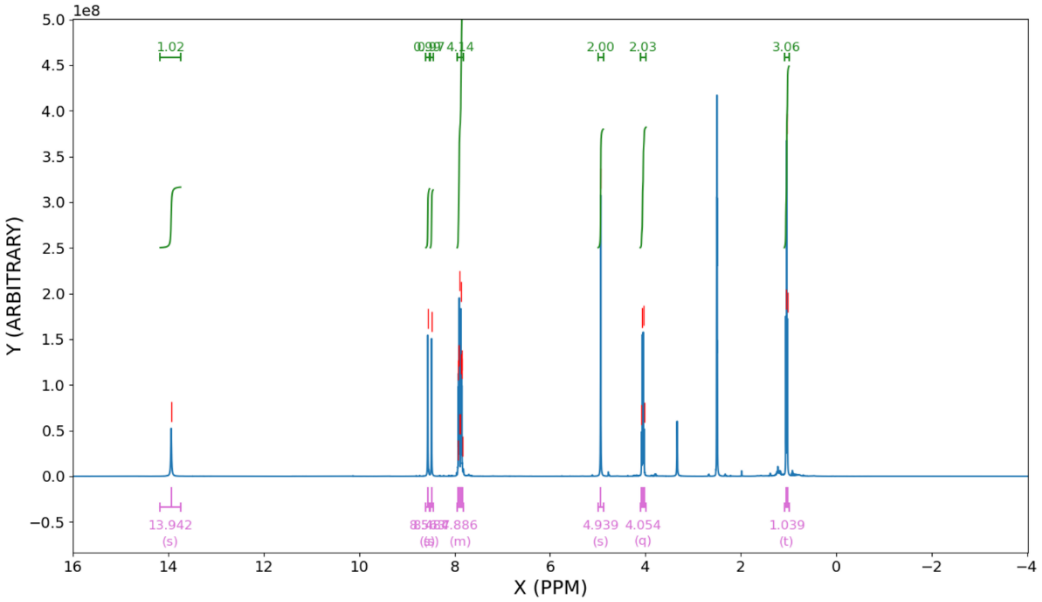

CHMO:0000595 | ^{13}C nuclear magnetic resonance spectroscopy (^{13}C NMR)

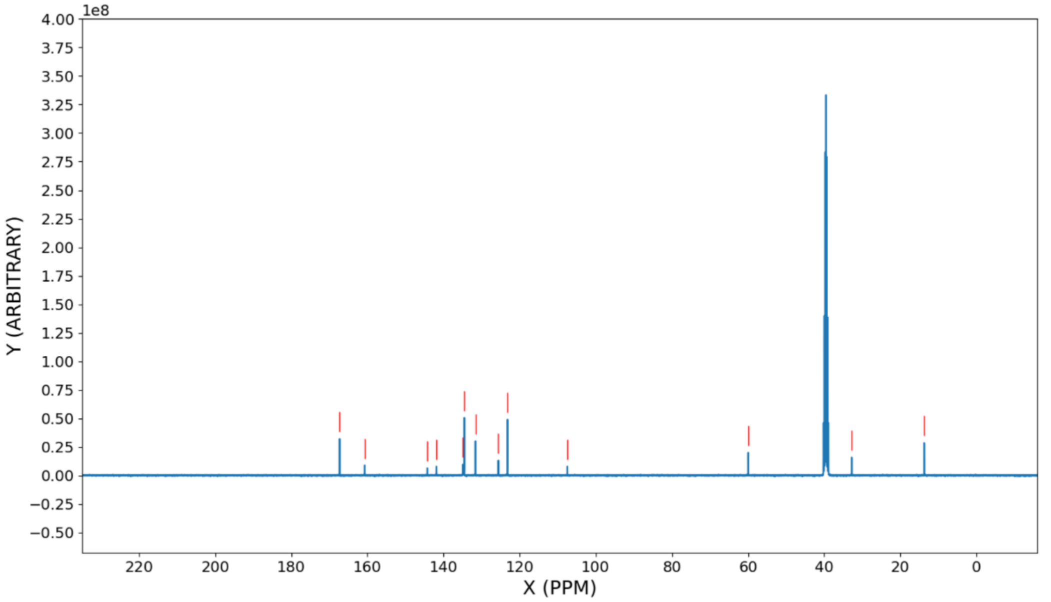

[**21kd**] ethyl 1-benzyl-3-(4-phenyl-1*H*-1,2,3-triazol-1-yl)-1*H*-pyrazole-4-carboxylate

CHMO:0000593 | ^{1}H nuclear magnetic resonance spectroscopy (^{1}H NMR)

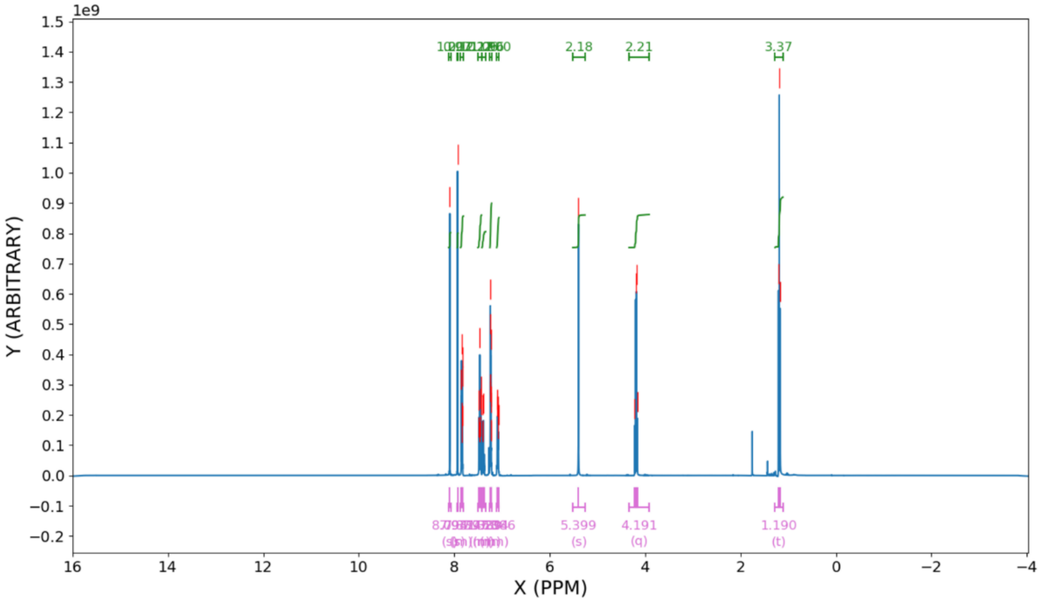

CHMO:0000595 | ^{13}C nuclear magnetic resonance spectroscopy (^{13}C NMR)

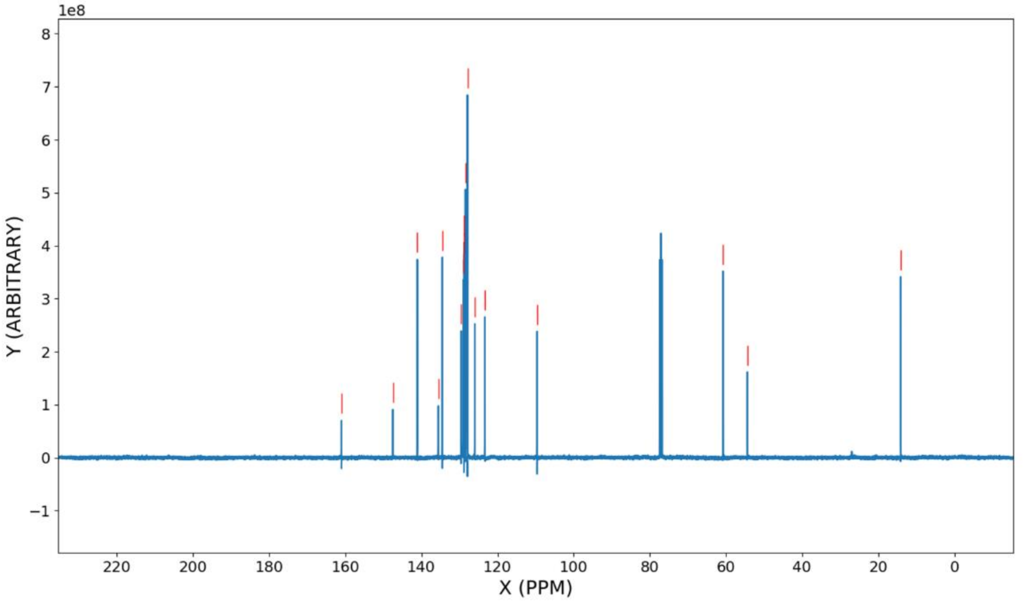

[**21lc**] ethyl 3-(4-(acetoxymethyl)-1*H*-1,2,3-triazol-1-yl)-1-(3,5-difluorobenzyl)-1*H*-pyrazole-4-carboxylate

CHMO:0000593 | ^{1}H nuclear magnetic resonance spectroscopy (^{1}H NMR)

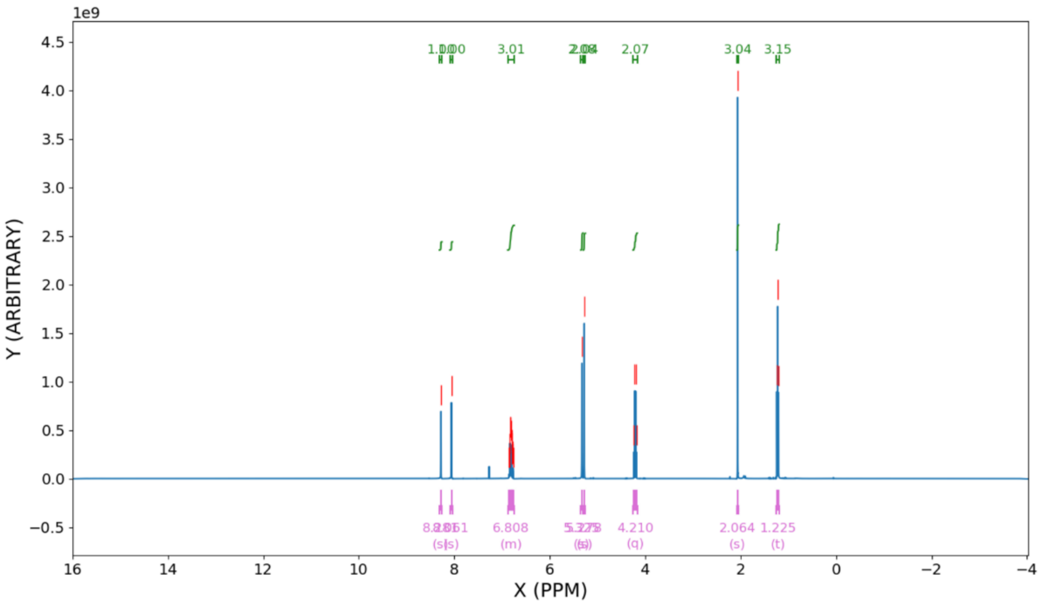

CHMO:0000595 | ^{13}C nuclear magnetic resonance spectroscopy (^{13}C NMR)

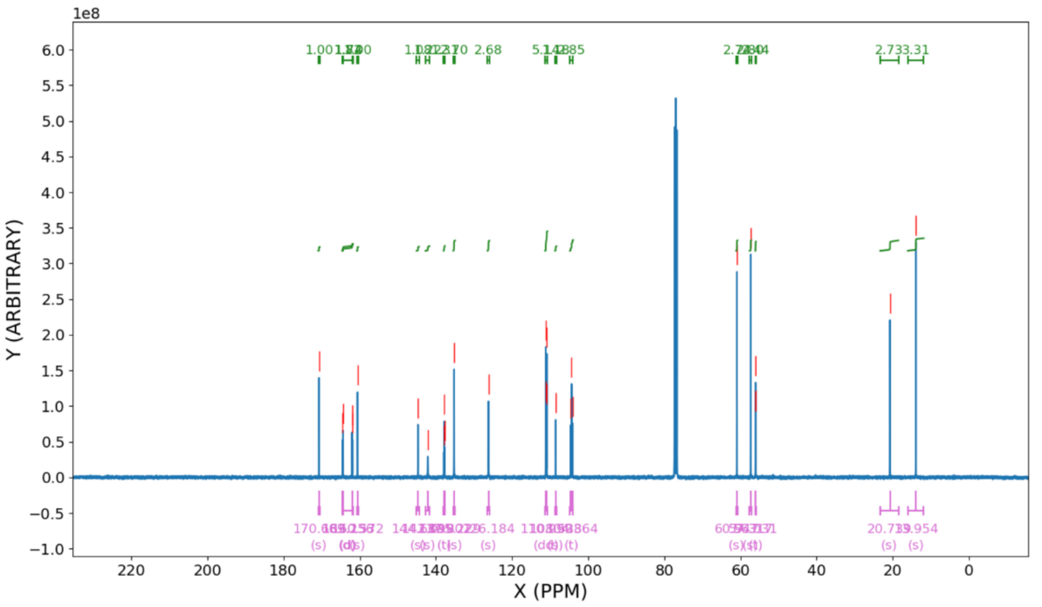

[**21lf**] ethyl 1-(3,5-difluorobenzyl)-3-(4-(4-(methoxycarbonyl)phenyl)-1*H*-1,2,3-triazol-1-yl)-1*H*-pyrazole-4-carboxylate

CHMO:0000593 | ^{1}H nuclear magnetic resonance spectroscopy (^{1}H NMR)

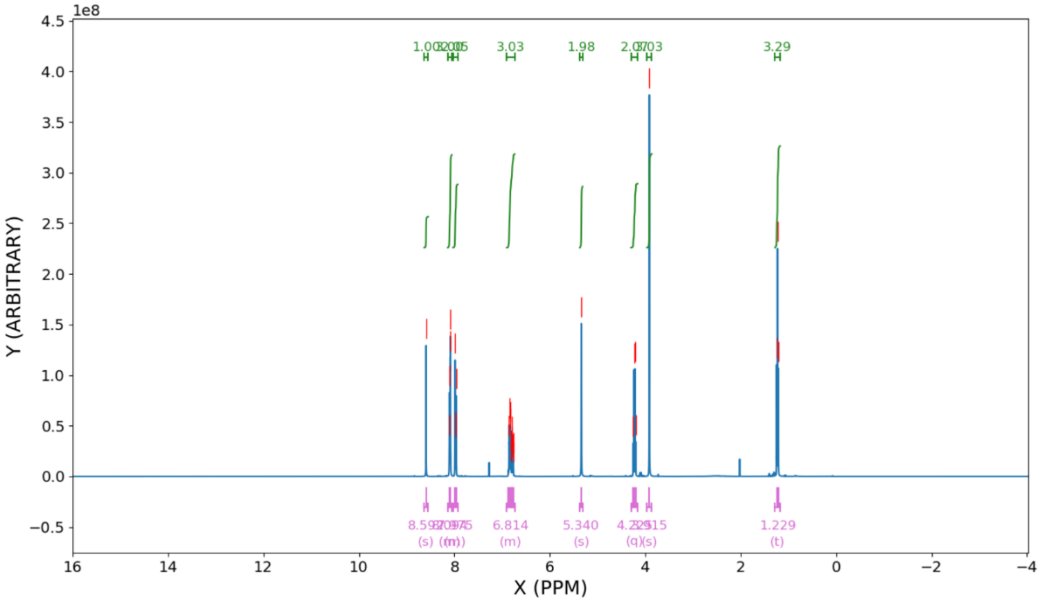

CHMO:0000595 | ^{13}C nuclear magnetic resonance spectroscopy (^{13}C NMR)

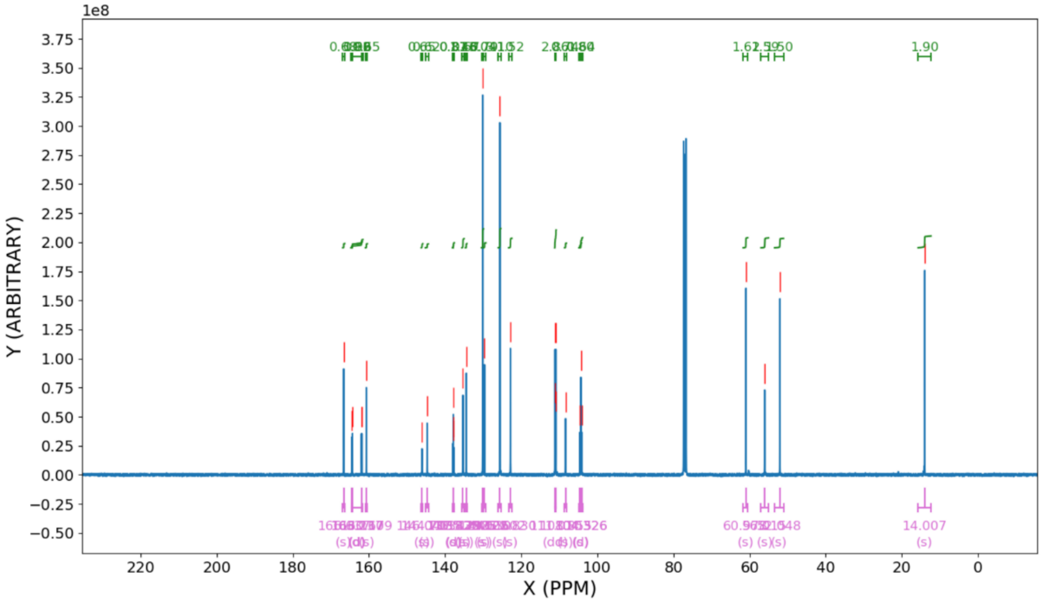

[**21me**] ethyl 1-(3-cyanobenzyl)-3-(4-(4-methoxyphenyl)-1*H*-1,2,3-triazol-1-yl)-1*H*-pyrazole-4-carboxylate

CHMO:0000593 | ^{1}H nuclear magnetic resonance spectroscopy (^{1}H NMR)

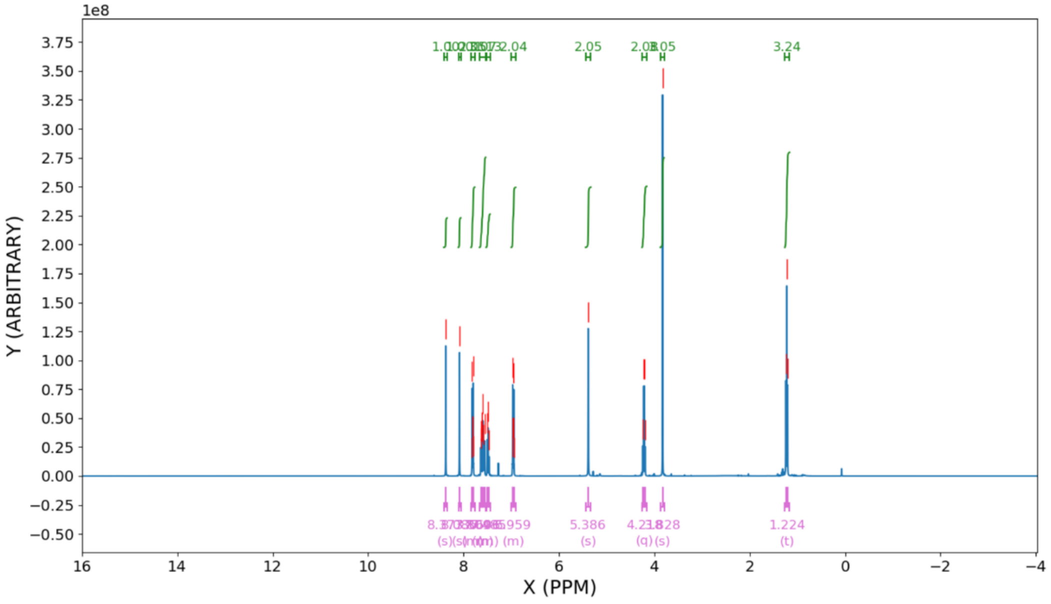

CHMO:0000595 | ^{13}C nuclear magnetic resonance spectroscopy (^{13}C NMR)

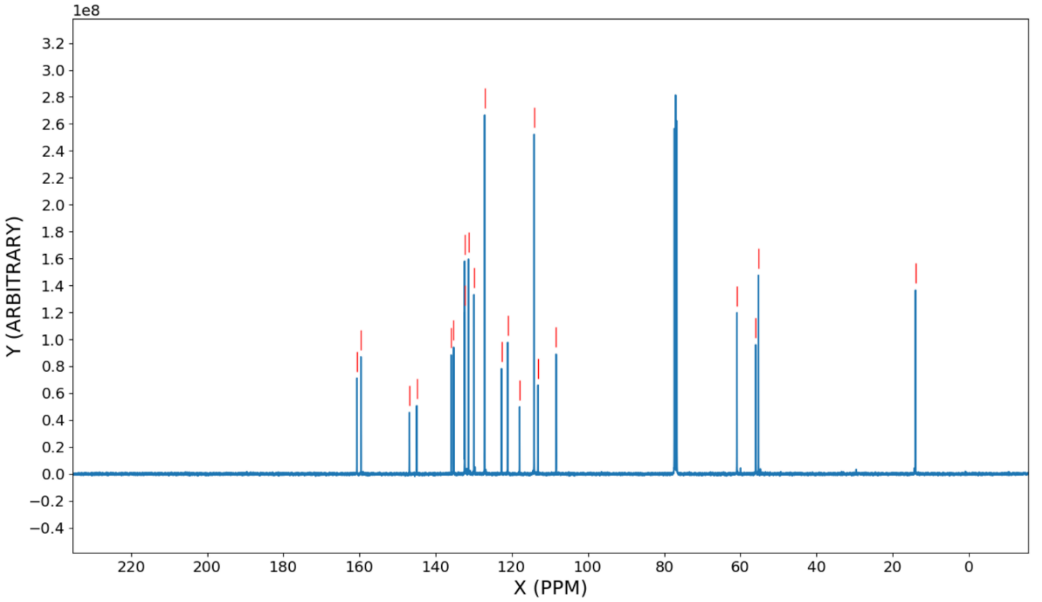

[**21mh**] ethyl 1-(3-cyanobenzyl)-3-(4-((1,3-dioxoisoindolin-2-yl)methyl)-1*H*-1,2,3-triazol-1-yl)-1*H*-pyrazole-4-carboxylate

CHMO:0000593 | ^{1}H nuclear magnetic resonance spectroscopy (^{1}H NMR)

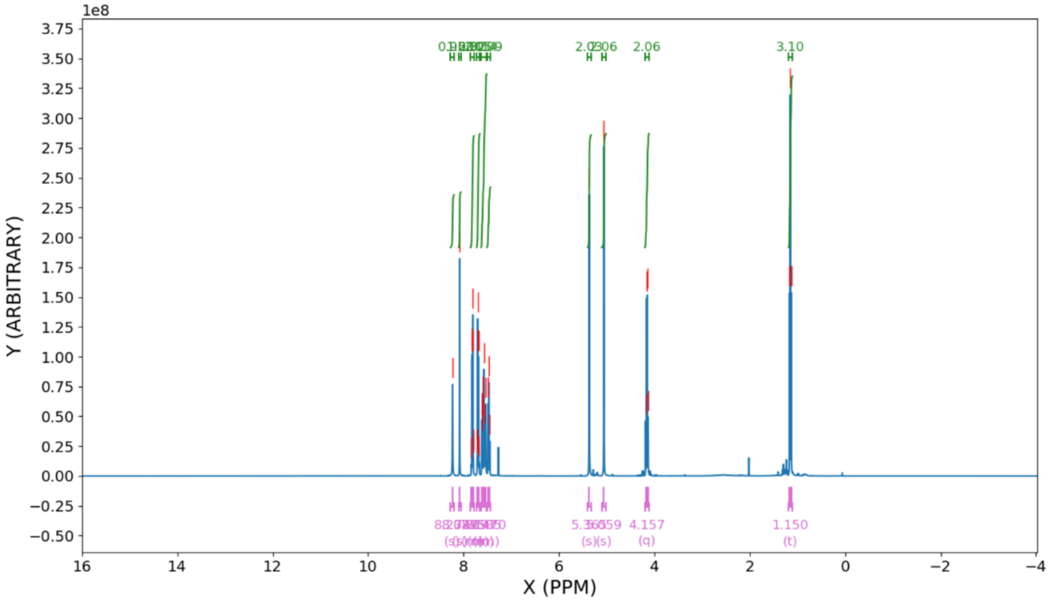

CHMO:0000595 | ^{13}C nuclear magnetic resonance spectroscopy (^{13}C NMR)

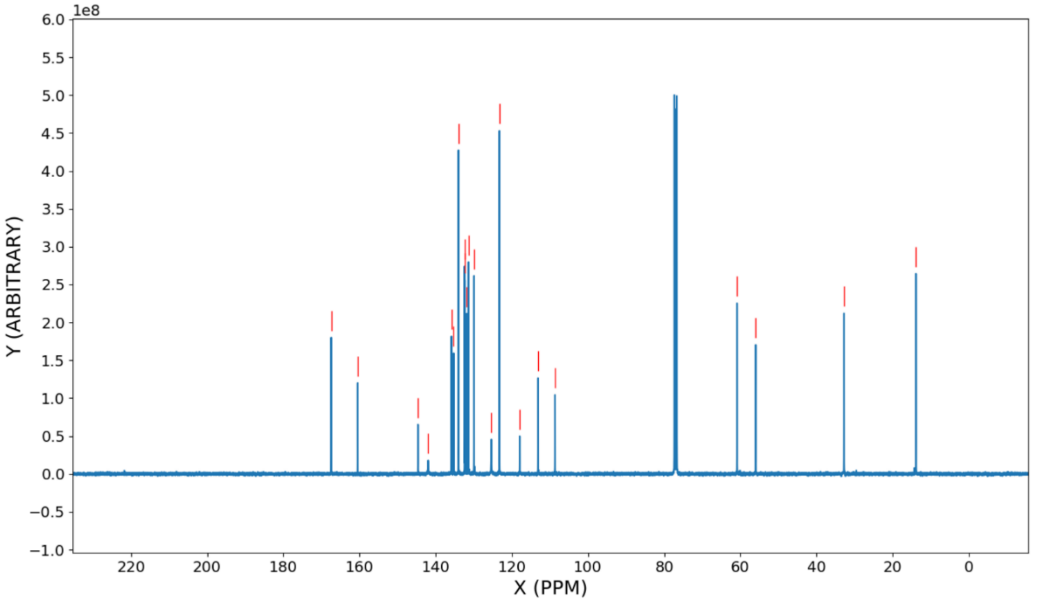

[**21na**] ethyl 1-(4-bromobenzyl)-3-(4-butyl-1*H*-1,2,3-triazol-1-yl)-1*H*-pyrazole-4-carboxylate

CHMO:0000593 | ^{1}H nuclear magnetic resonance spectroscopy (^{1}H NMR)

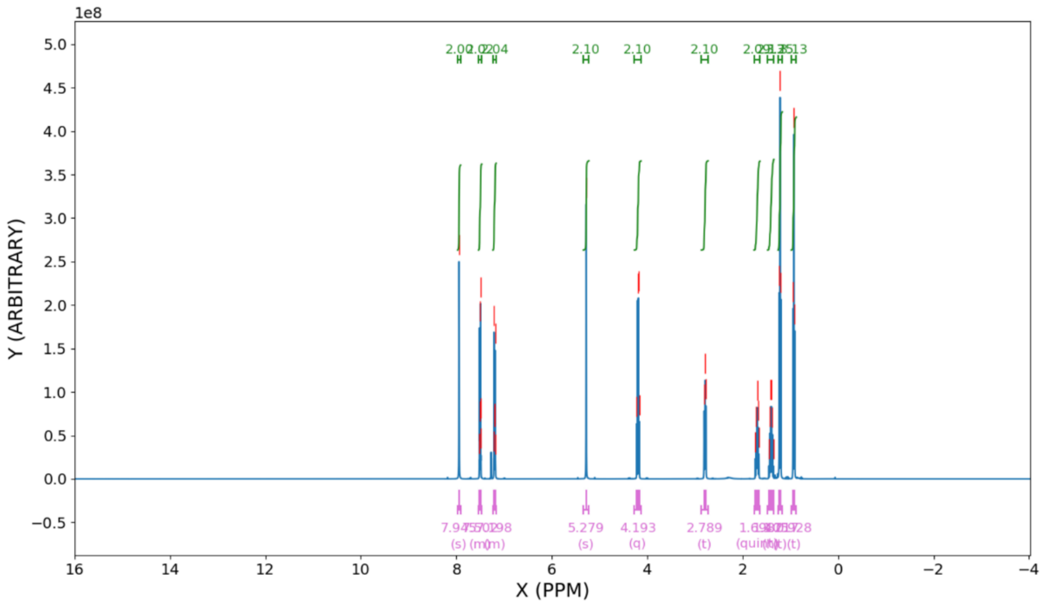

CHMO:0000595 | ^{13}C nuclear magnetic resonance spectroscopy (^{13}C NMR)

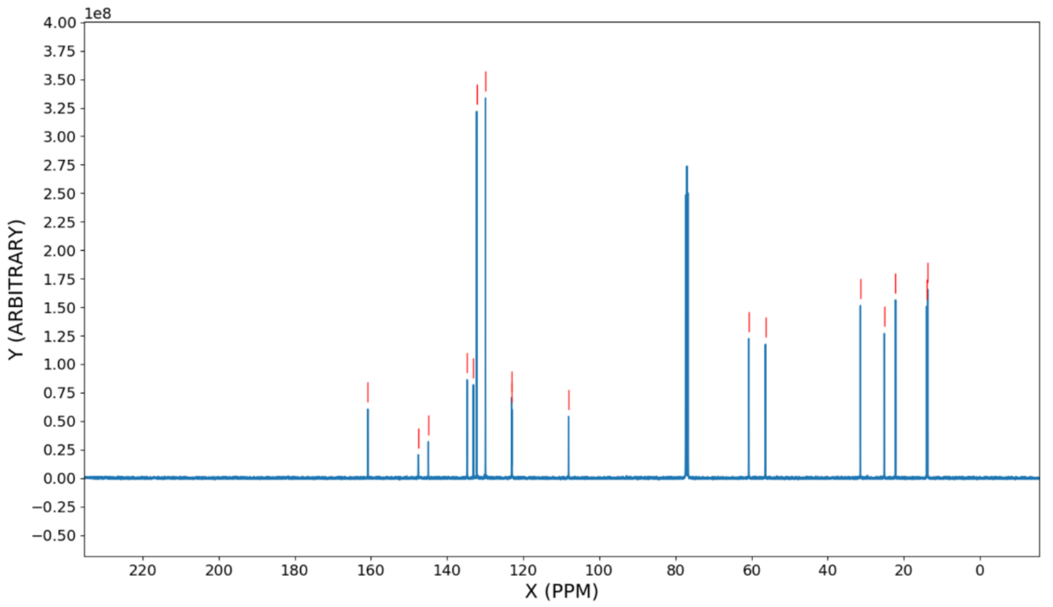

[**21nc**] ethyl 3-(4-(acetoxymethyl)-1*H*-1,2,3-triazol-1-yl)-1-(4-bromobenzyl)-1*H*-pyrazole-4-carboxylate

CHMO:0000593 | ^{1}H nuclear magnetic resonance spectroscopy (^{1}H NMR)

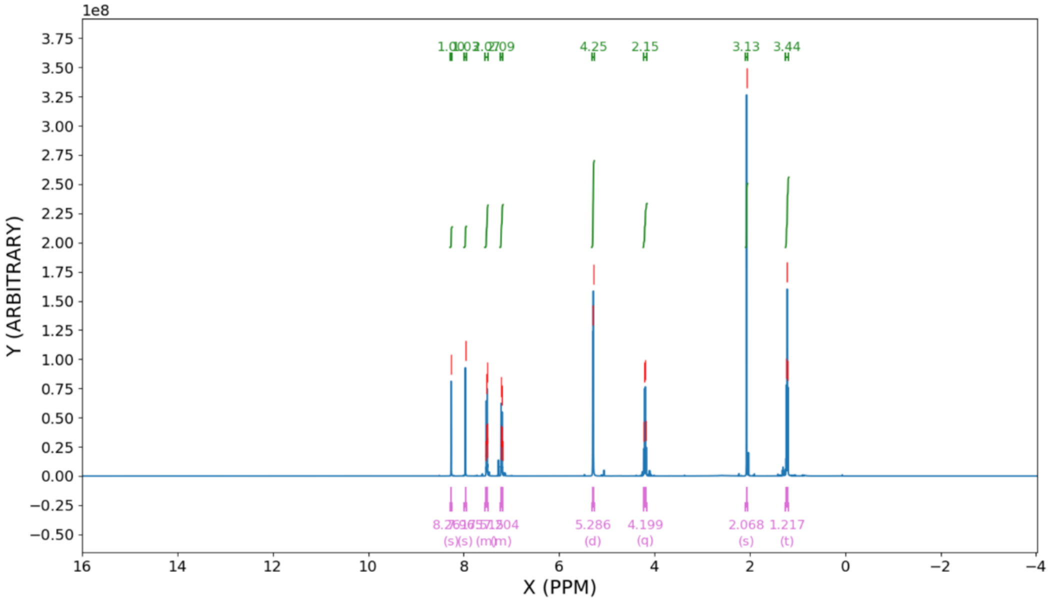

CHMO:0000595 | ^{13}C nuclear magnetic resonance spectroscopy (^{13}C NMR)

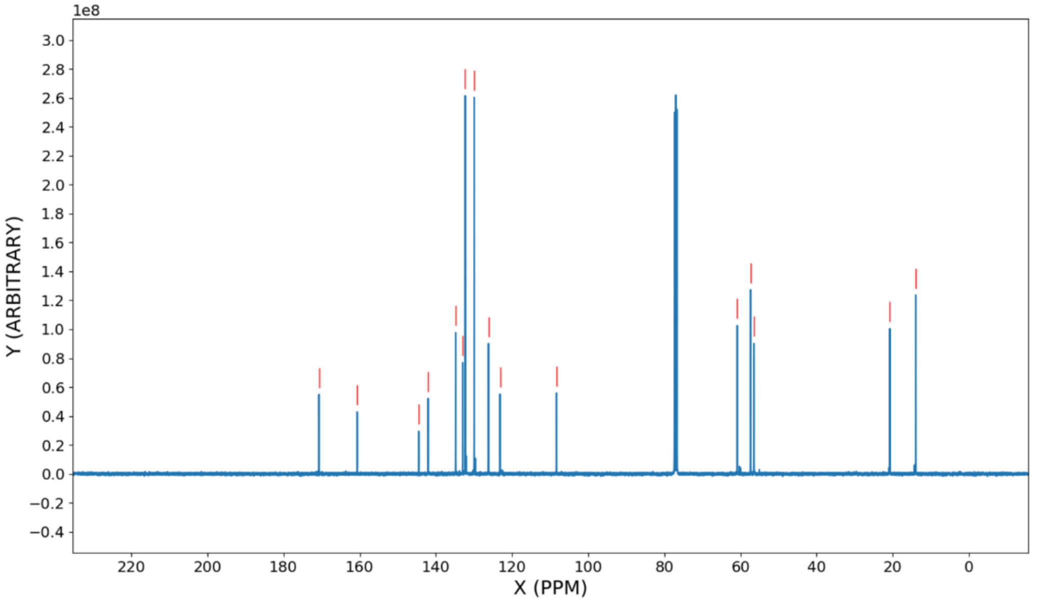

[**21nd**] ethyl 1-(4-bromobenzyl)-3-(4-phenyl-1*H*-1,2,3-triazol-1-yl)-1*H*-pyrazole-4-carboxylate

CHMO:0000593 | ^{1}H nuclear magnetic resonance spectroscopy (^{1}H NMR)

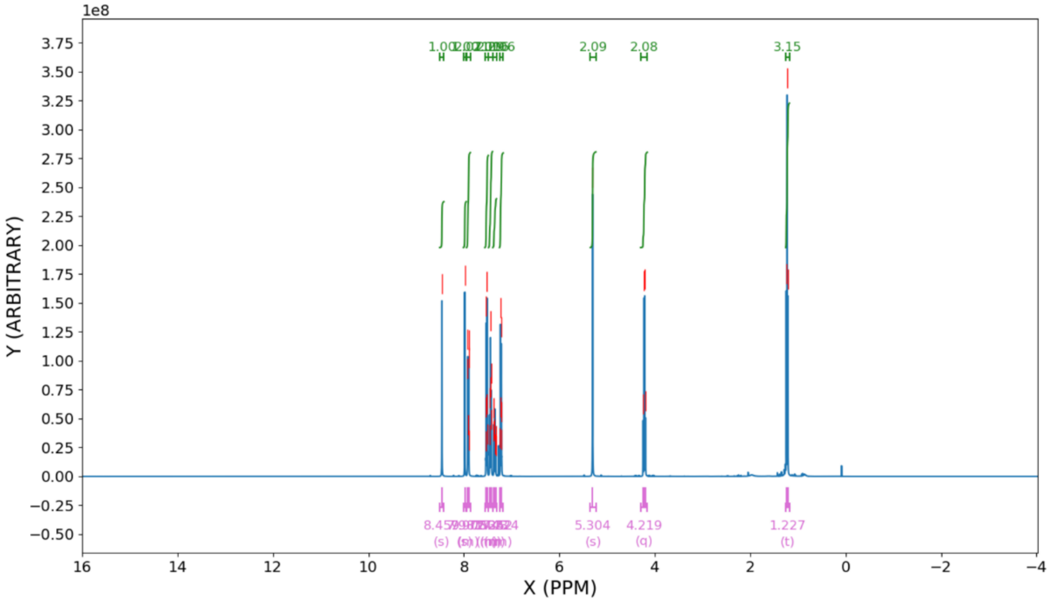

CHMO:0000595 | ^{13}C nuclear magnetic resonance spectroscopy (^{13}C NMR)

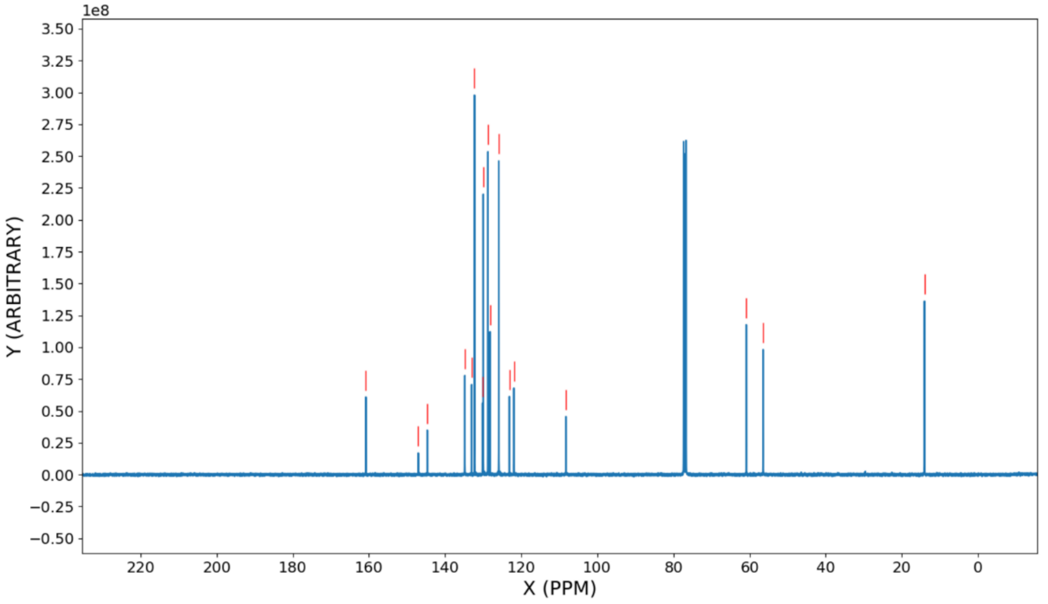

[**21oa**] 3-(4-butyl-1*H*-1,2,3-triazol-1-yl)-1*H*-pyrazole-4-carbonitrile

CHMO:0000593 | ^{1}H nuclear magnetic resonance spectroscopy (^{1}H NMR)

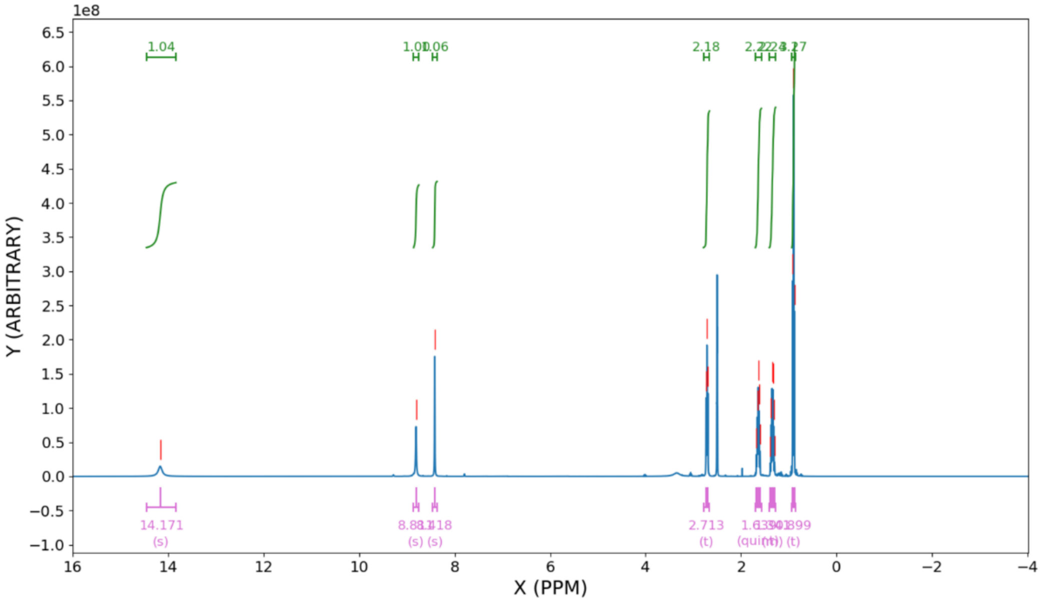

CHMO:0000595 | ^{13}C nuclear magnetic resonance spectroscopy (^{13}C NMR)

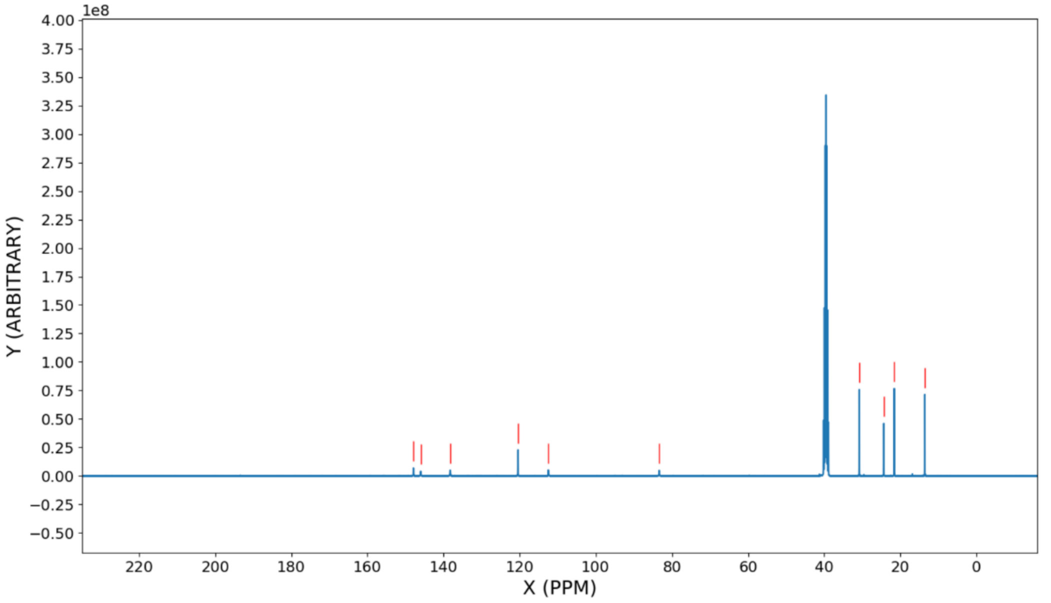

[**21oc**] (1-(4-cyano-1*H*-pyrazol-3-yl)-1*H*-1,2,3-triazol-4-yl)methyl acetate

CHMO:0000593 | ^{1}H nuclear magnetic resonance spectroscopy (^{1}H NMR)

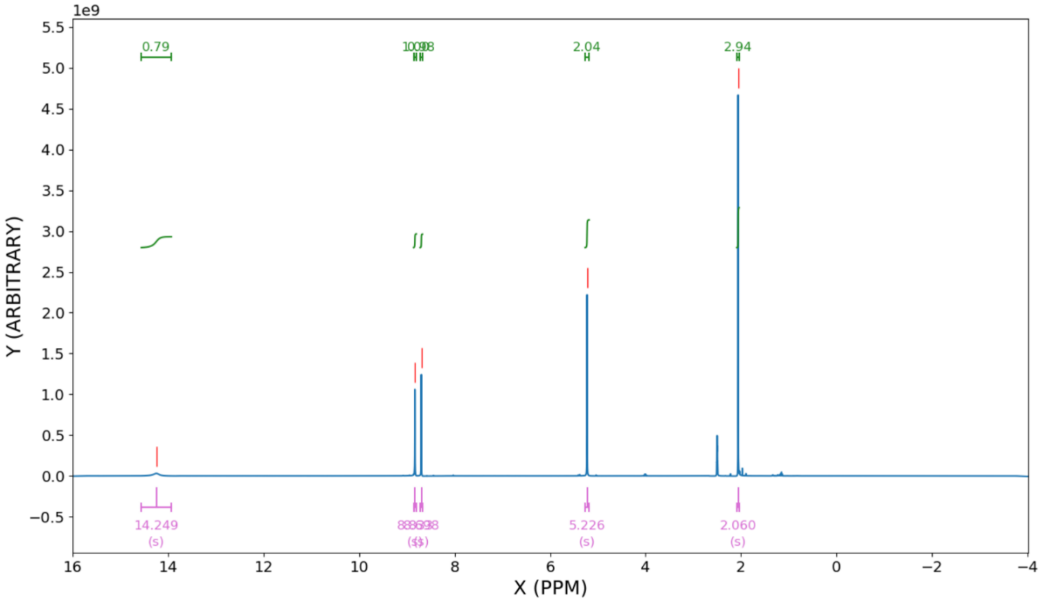

CHMO:0000595 | ^{13}C nuclear magnetic resonance spectroscopy (^{13}C NMR)

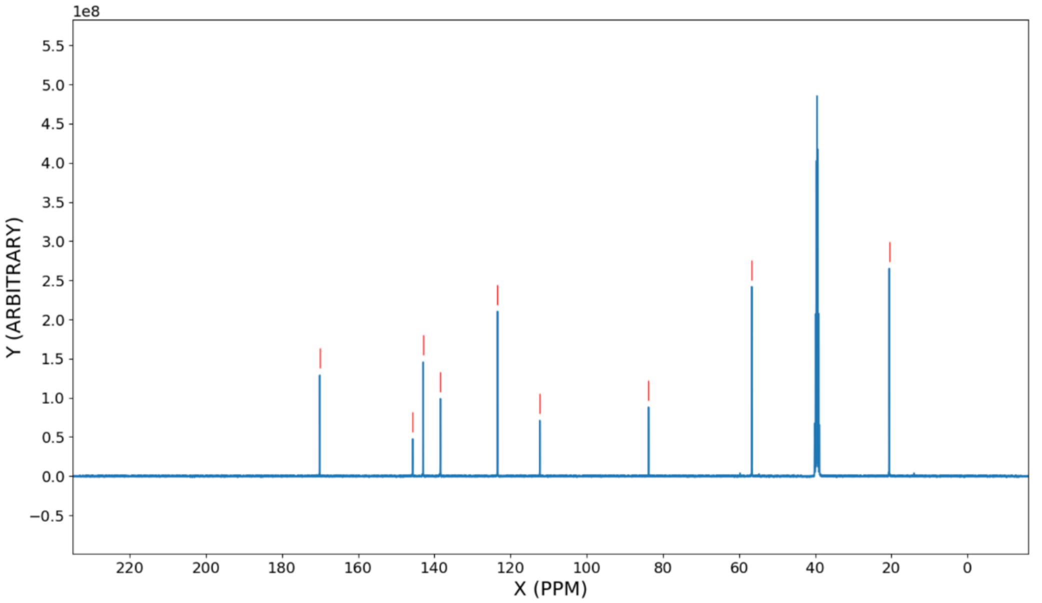

[**21od**] 3-(4-phenyl-1*H*-1,2,3-triazol-1-yl)-1*H*-pyrazole-4-carbonitrile

CHMO:0000593 | ^{1}H nuclear magnetic resonance spectroscopy (^{1}H NMR)

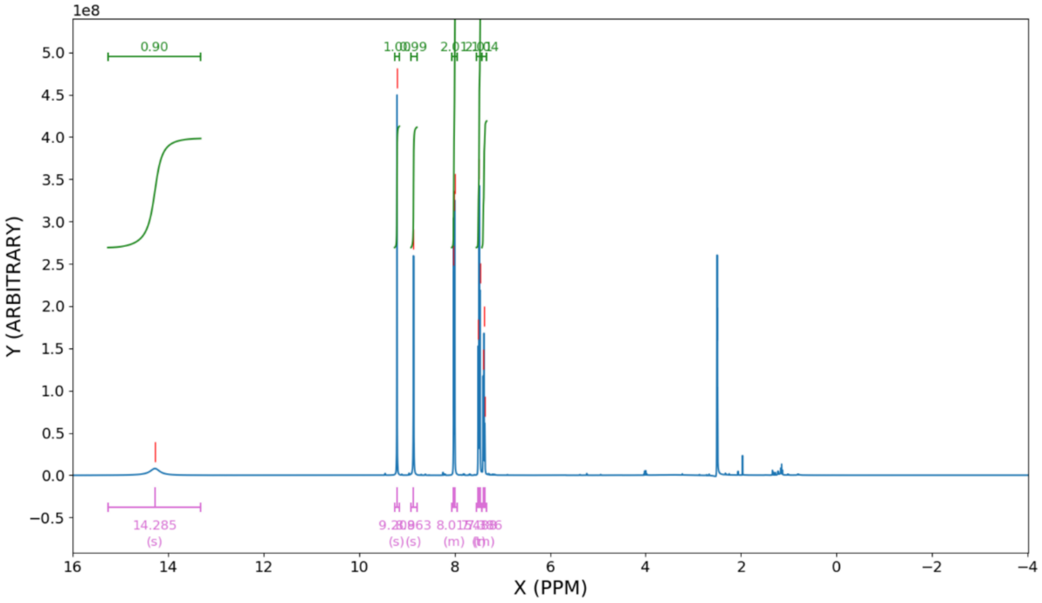

CHMO:0000595 | ^{13}C nuclear magnetic resonance spectroscopy (^{13}C NMR)

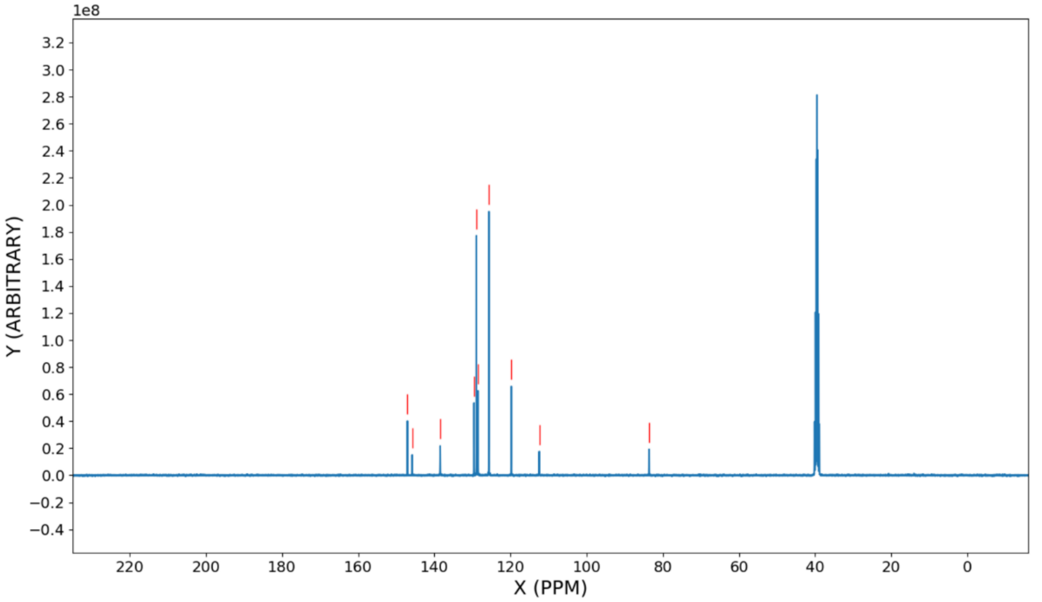

[**21of**] methyl 4-(1-(4-cyano-1*H*-pyrazol-3-yl)-1*H*-1,2,3-triazol-4-yl)benzoate

CHMO:0000593 | ^{1}H nuclear magnetic resonance spectroscopy (^{1}H NMR)

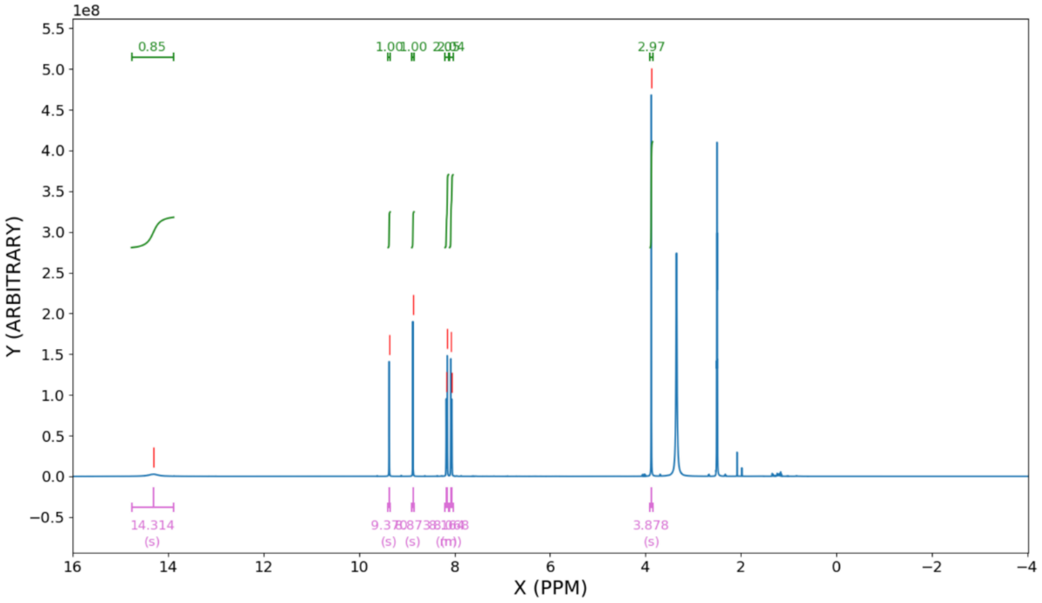

CHMO:0000595 | ^{13}C nuclear magnetic resonance spectroscopy (^{13}C NMR)

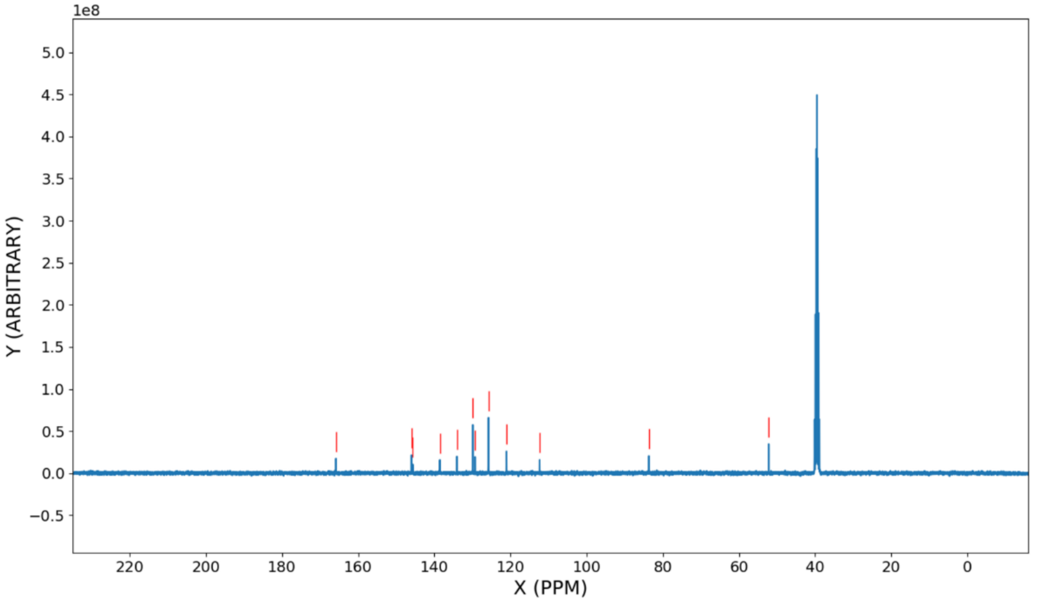

[**21pa**] 3-(4-butyl-1*H*-1,2,3-triazol-1-yl)-1-isopropyl-1*H*-pyrazole-4-carbonitrile

CHMO:0000593 | ^{1}H nuclear magnetic resonance spectroscopy (^{1}H NMR)

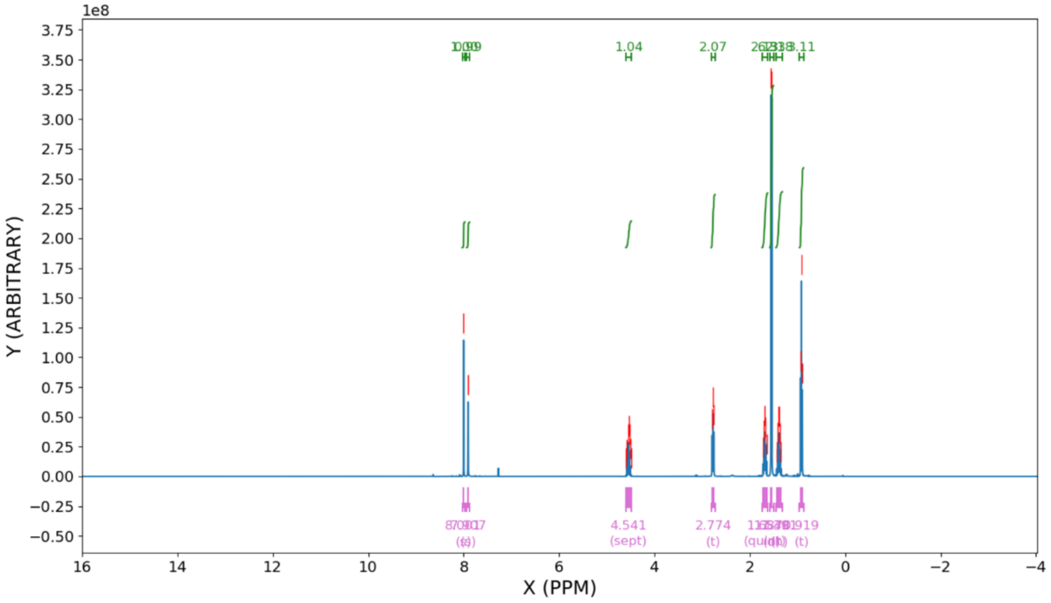

CHMO:0000595 | ^{13}C nuclear magnetic resonance spectroscopy (^{13}C NMR)

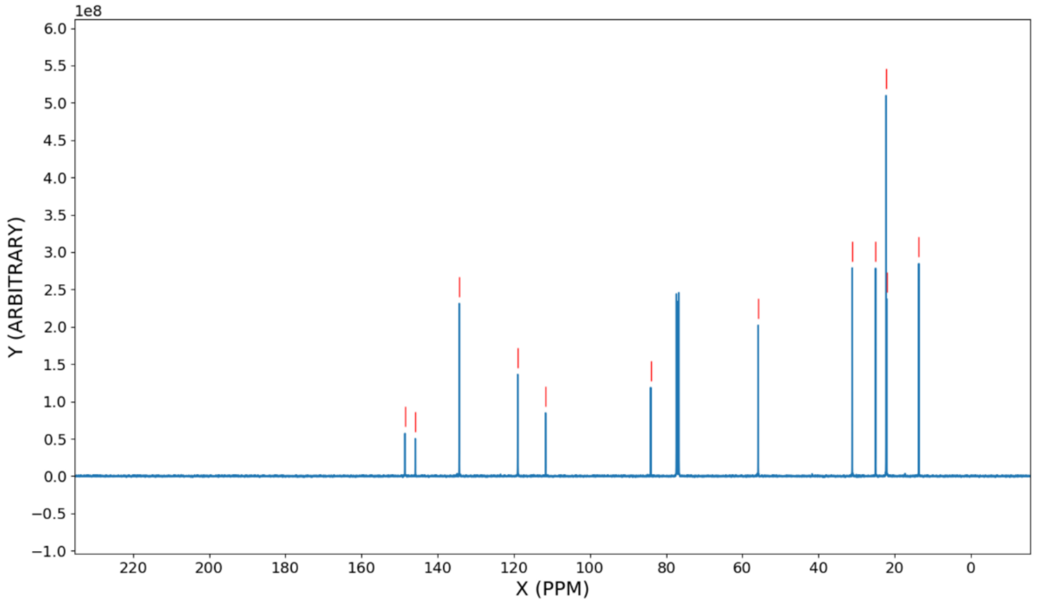

[**21pd**] 1-isopropyl-3-(4-phenyl-1*H*-1,2,3-triazol-1-yl)-1*H*-pyrazole-4-carbonitrile

CHMO:0000593 | ^{1}H nuclear magnetic resonance spectroscopy (^{1}H NMR)

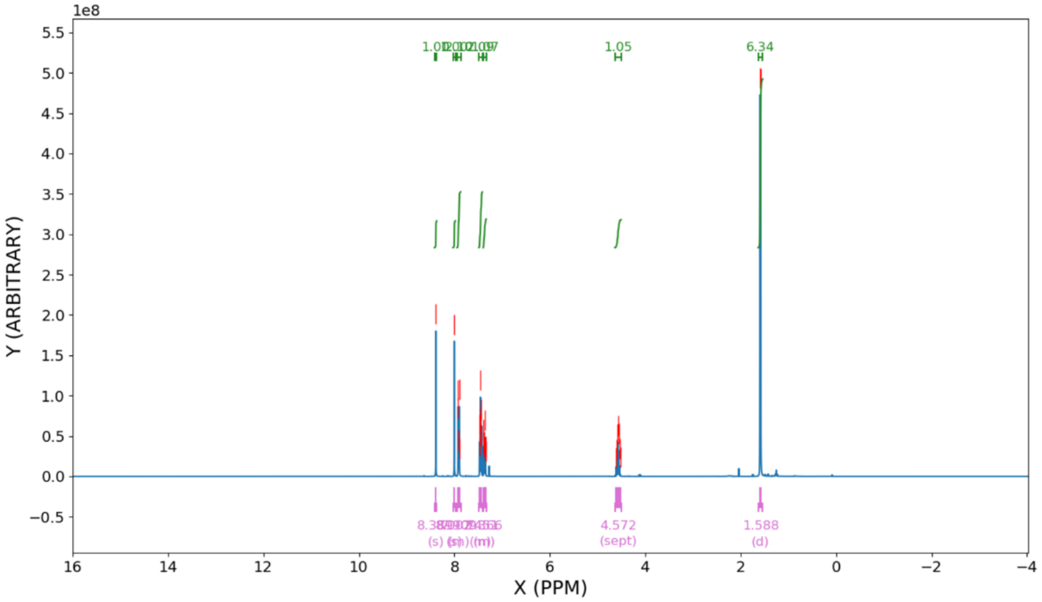

CHMO:0000595 | ^{13}C nuclear magnetic resonance spectroscopy (^{13}C NMR)

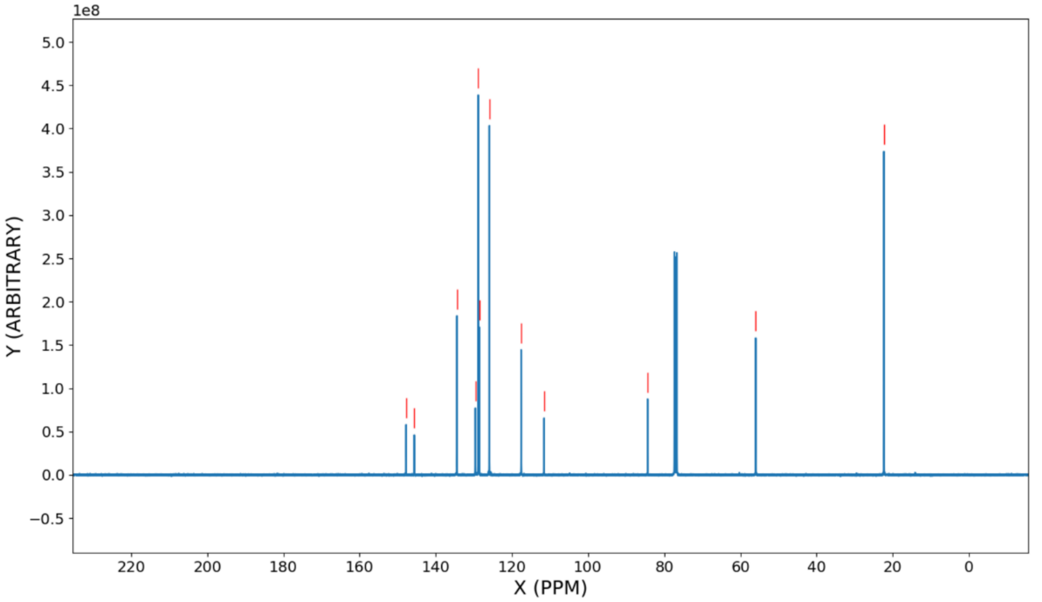

[**21qa**] ethyl 2-(3-(4-butyl-1*H*-1,2,3-triazol-1-yl)-4-cyano-1*H*-pyrazol-1-yl)acetate

CHMO:0000593 | ^{1}H nuclear magnetic resonance spectroscopy (^{1}H NMR)

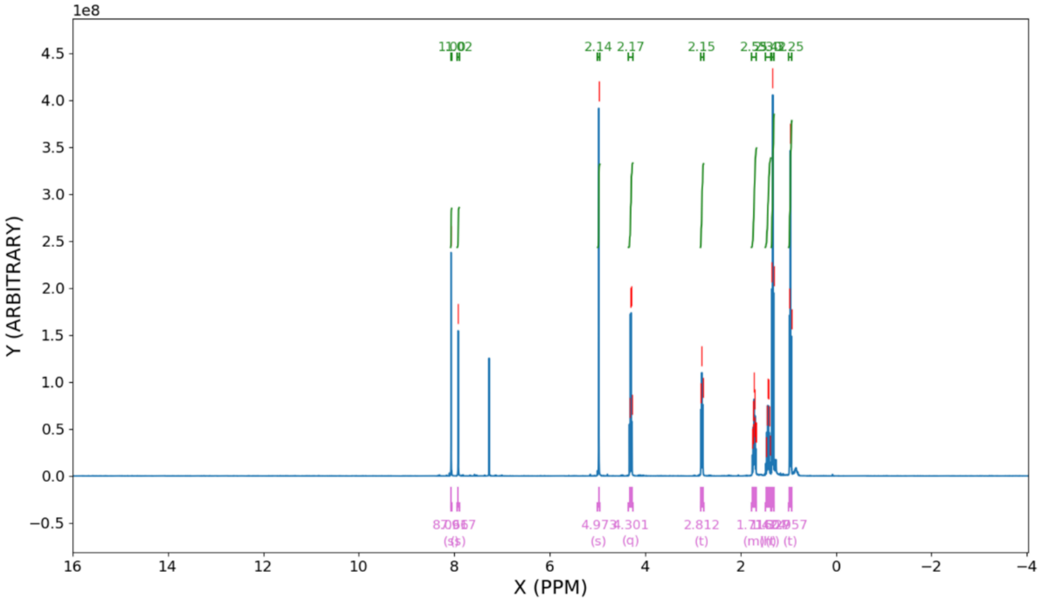

CHMO:0000595 | ^{13}C nuclear magnetic resonance spectroscopy (^{13}C NMR)

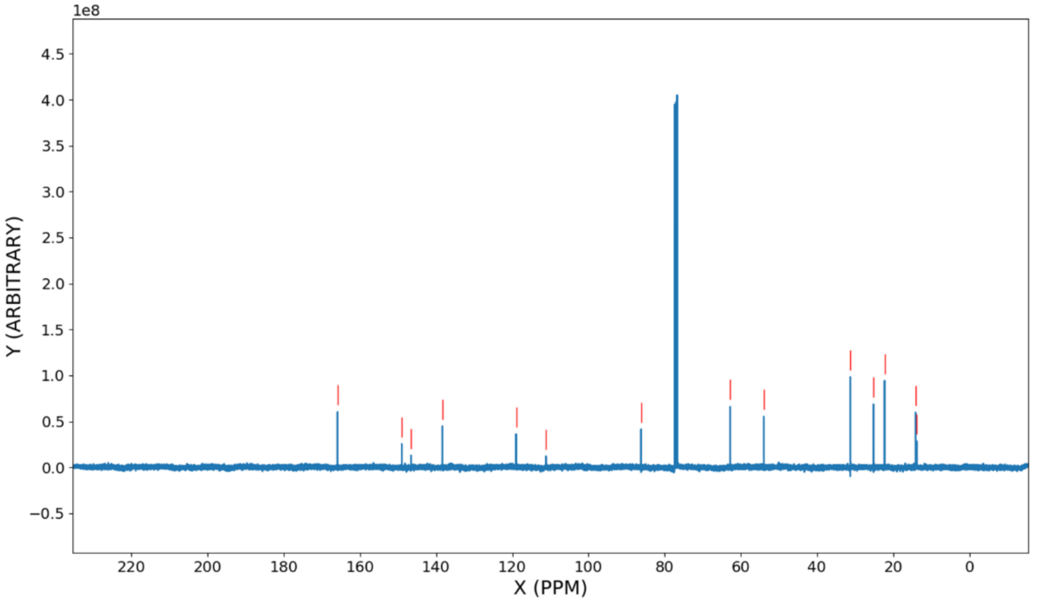

[**21rd**] 1-benzyl-3-(4-phenyl-1*H*-1,2,3-triazol-1-yl)-1*H*-pyrazole-4-carbonitrile

CHMO:0000593 | ^{1}H nuclear magnetic resonance spectroscopy (^{1}H NMR)

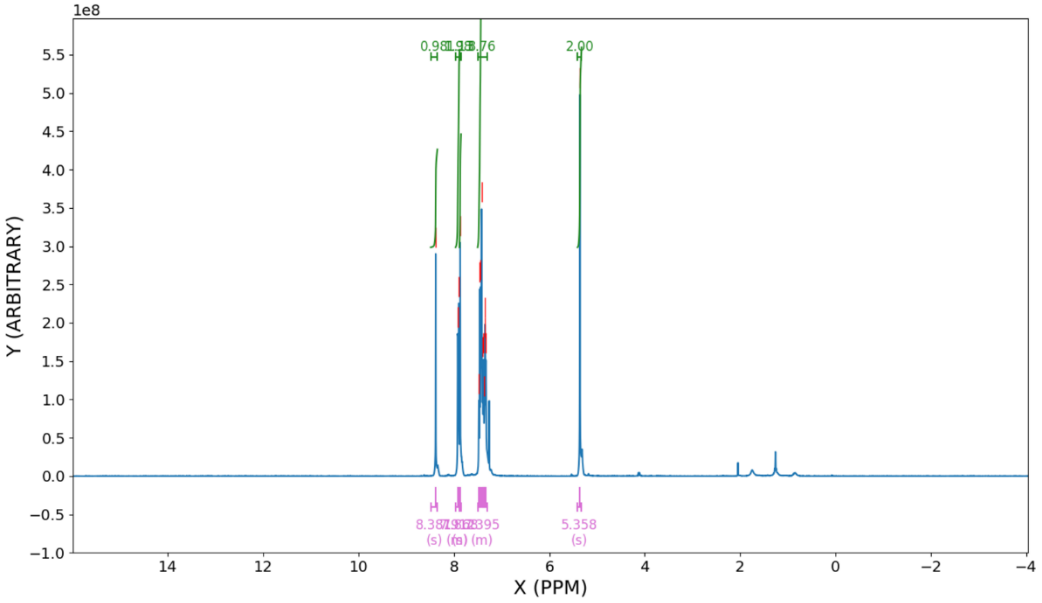

CHMO:0000595 | ^{13}C nuclear magnetic resonance spectroscopy (^{13}C NMR)

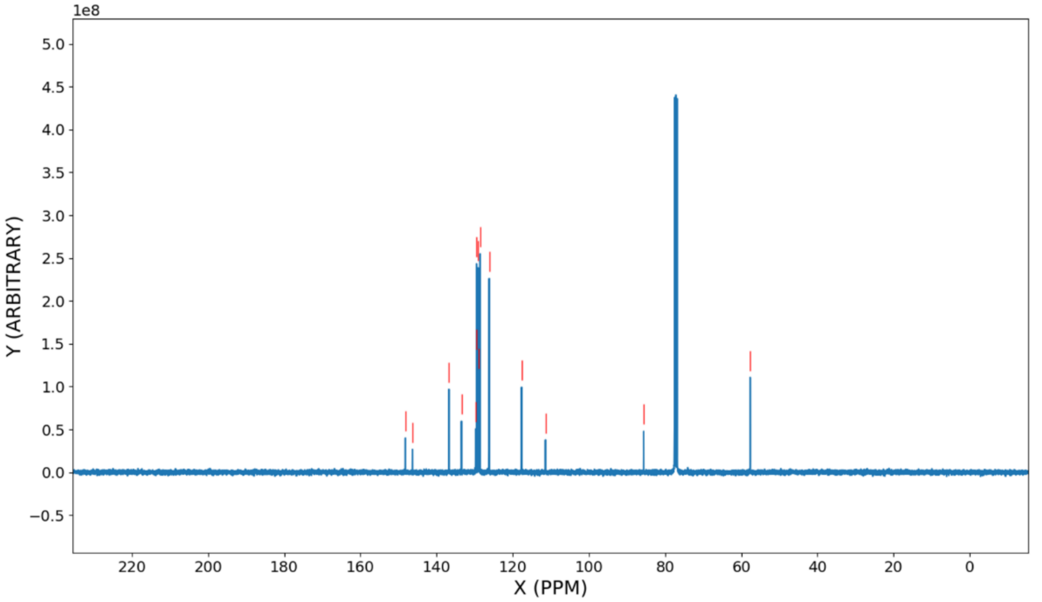

[**21sa**] 3-(4-butyl-1*H*-1,2,3-triazol-1-yl)-1-(3,5-difluorobenzyl)-1*H*-pyrazole-4-carbonitrile

CHMO:0000593 | ^{1}H nuclear magnetic resonance spectroscopy (^{1}H NMR)

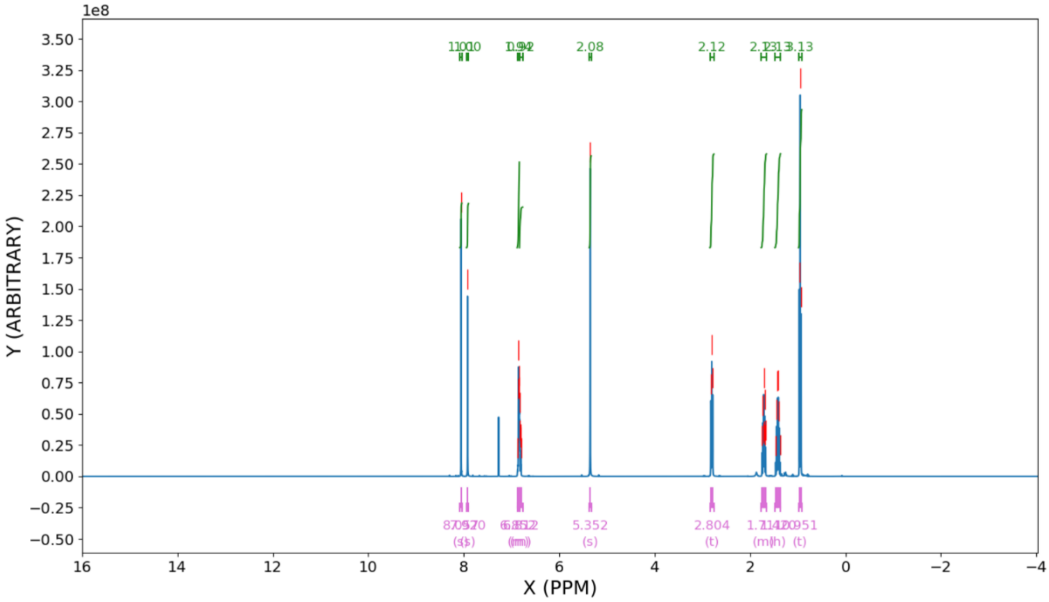

CHMO:0000595 | ^{13}C nuclear magnetic resonance spectroscopy (^{13}C NMR)

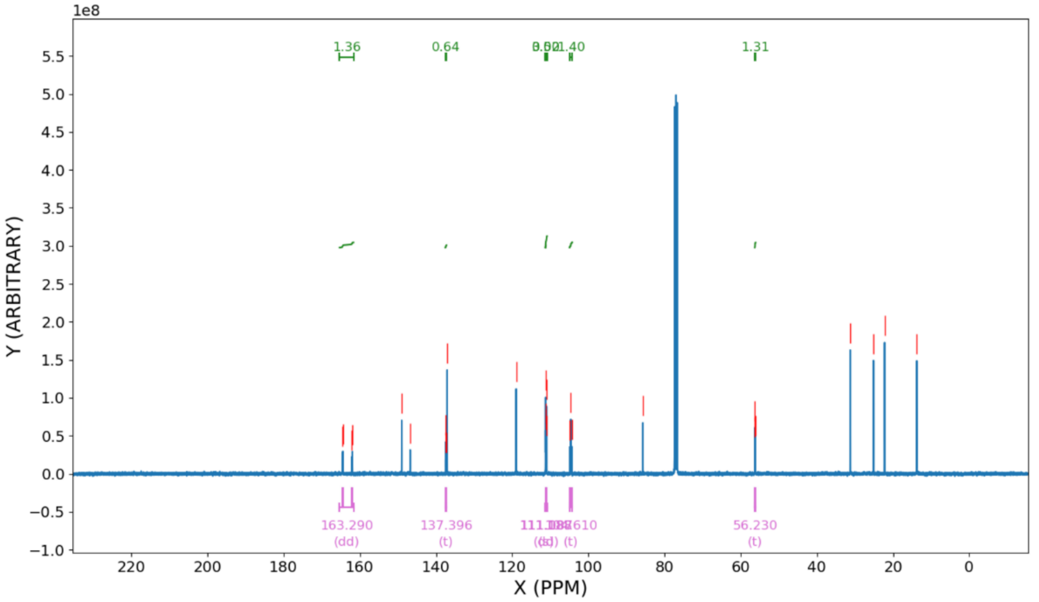

[**21sd**] 1-(3,5-difluorobenzyl)-3-(4-phenyl-1*H*-1,2,3-triazol-1-yl)-1*H*-pyrazole-4-carbonitrile

CHMO:0000593 | ^{1}H nuclear magnetic resonance spectroscopy (^{1}H NMR)

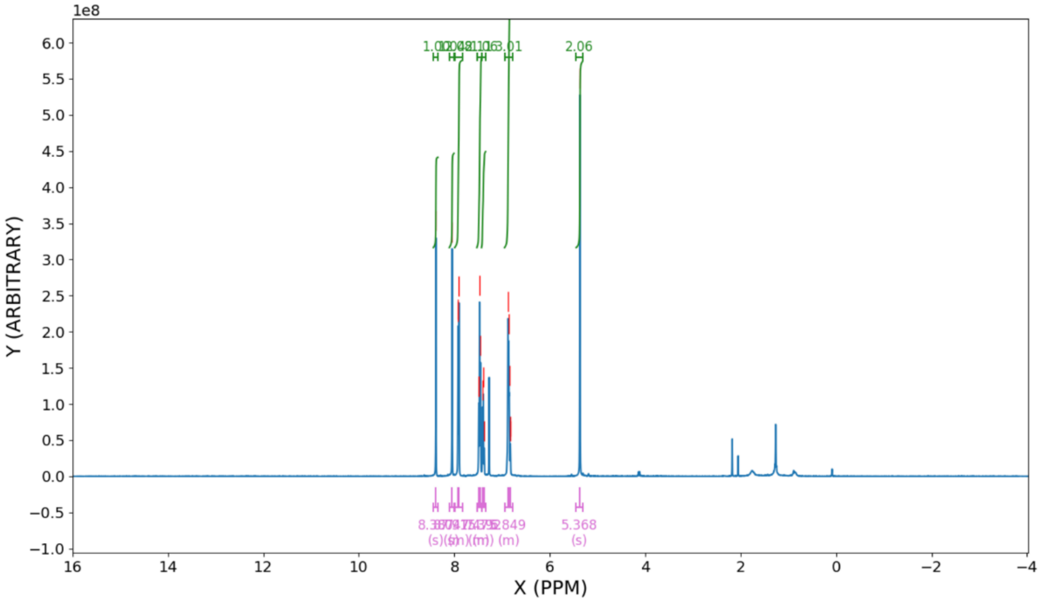

CHMO:0000595 | ^{13}C nuclear magnetic resonance spectroscopy (^{13}C NMR)

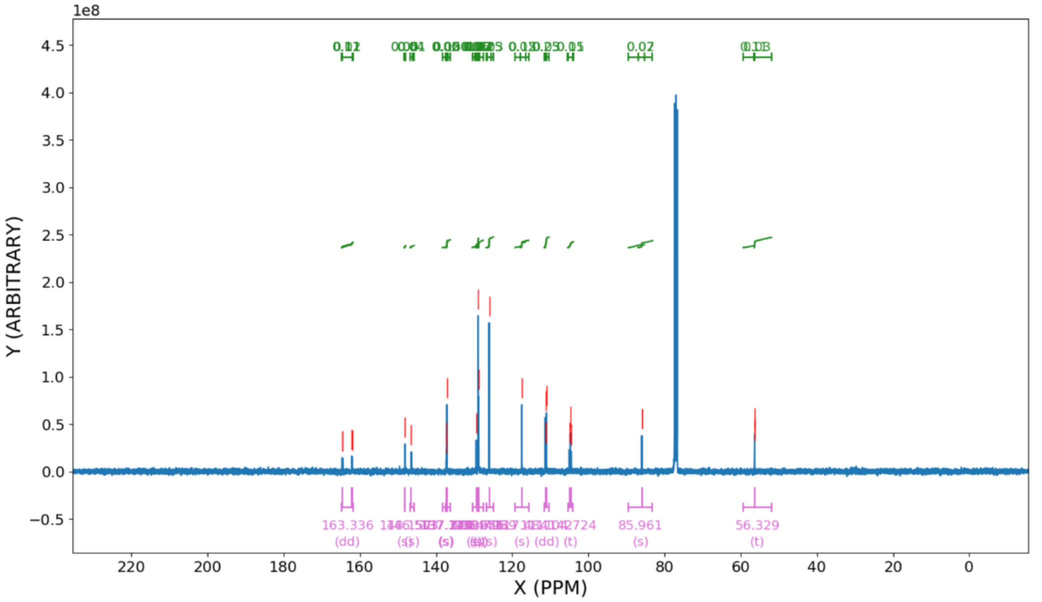

[**21ta**] 3-(4-butyl-1*H*-1,2,3-triazol-1-yl)-1-(4-methylbenzyl)-1*H*-pyrazole-4-carbonitrile

CHMO:0000593 | ^{1}H nuclear magnetic resonance spectroscopy (^{1}H NMR)

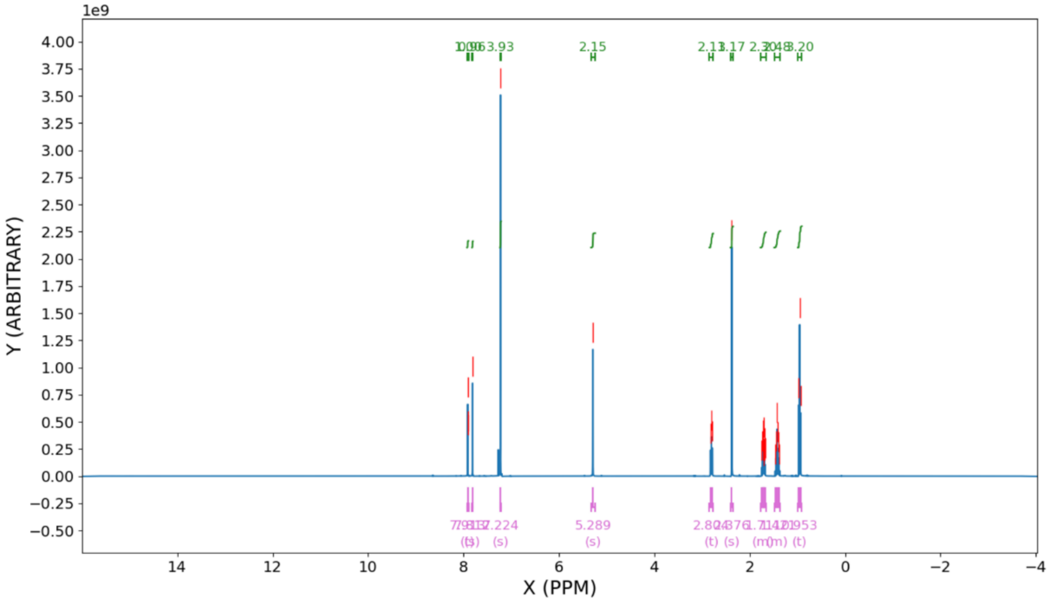

CHMO:0000595 | ^{13}C nuclear magnetic resonance spectroscopy (^{13}C NMR)

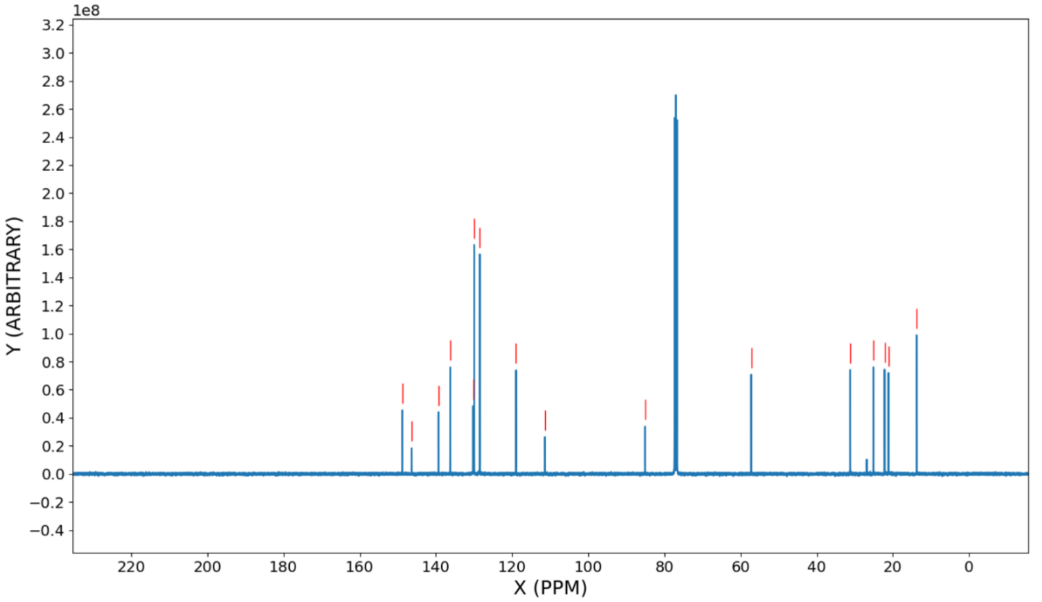

[**21td**] 1-(4-methylbenzyl)-3-(4-phenyl-1*H*-1,2,3-triazol-1-yl)-1*H*-pyrazole-4-carbonitrile

CHMO:0000593 | ^{1}H nuclear magnetic resonance spectroscopy (^{1}H NMR)

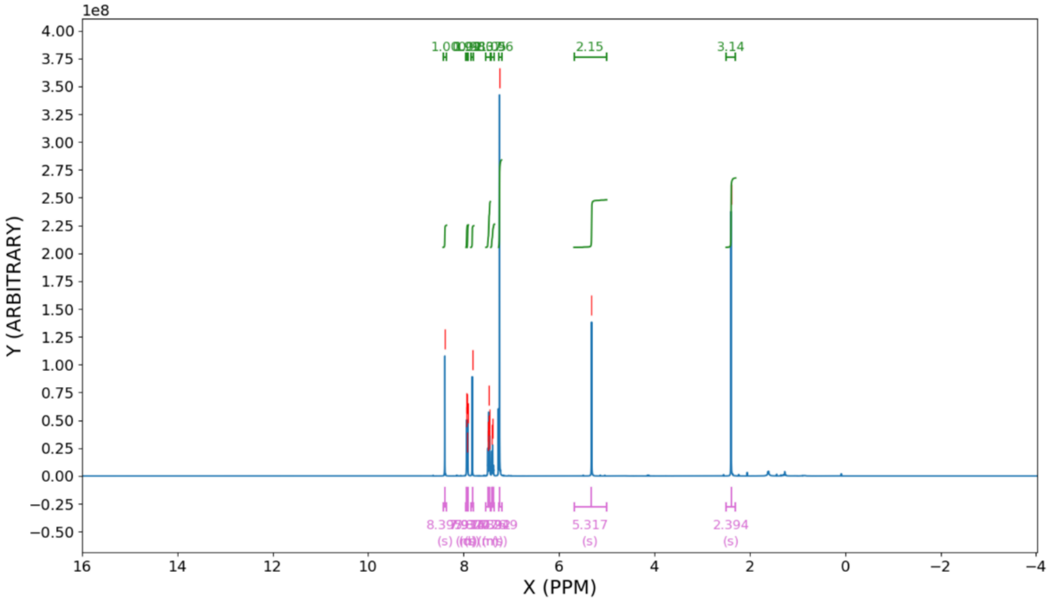

CHMO:0000595 | ^{13}C nuclear magnetic resonance spectroscopy (^{13}C NMR)

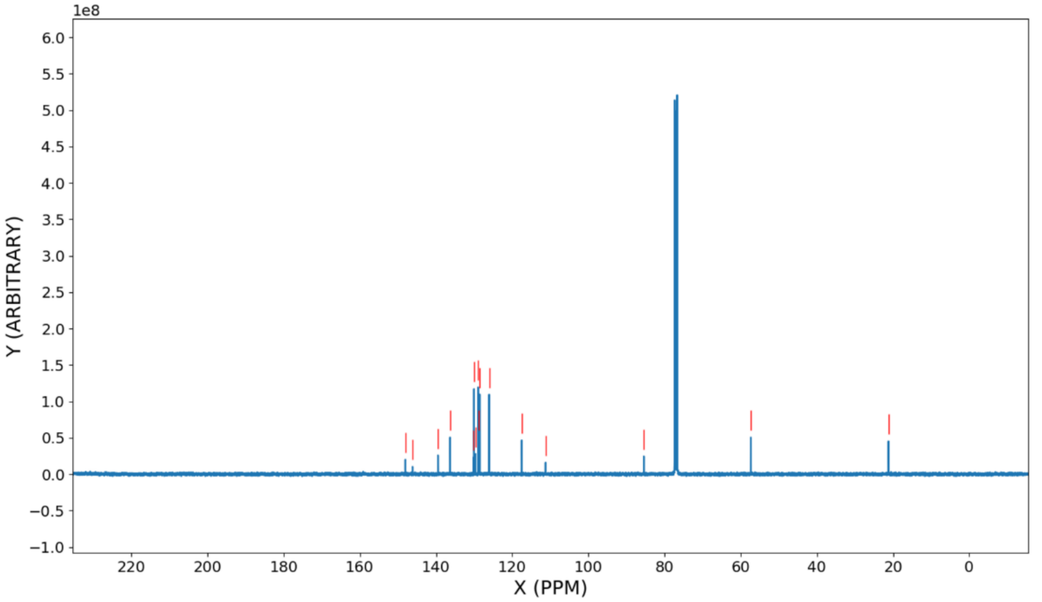

[**21tf**] methyl 4-(1-(4-cyano-1-(4-methylbenzyl)-1*H*-pyrazol-3-yl)-1*H*-1,2,3-triazol-4-yl)benzoate

CHMO:0000593 | ^{1}H nuclear magnetic resonance spectroscopy (^{1}H NMR)

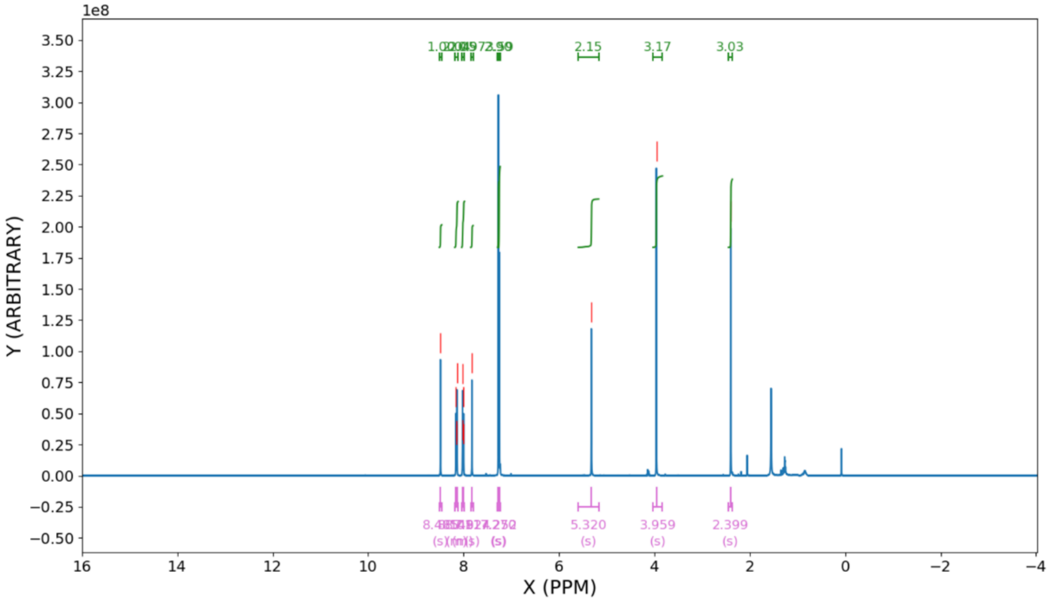

CHMO:0000595 | ^{13}C nuclear magnetic resonance spectroscopy (^{13}C NMR)

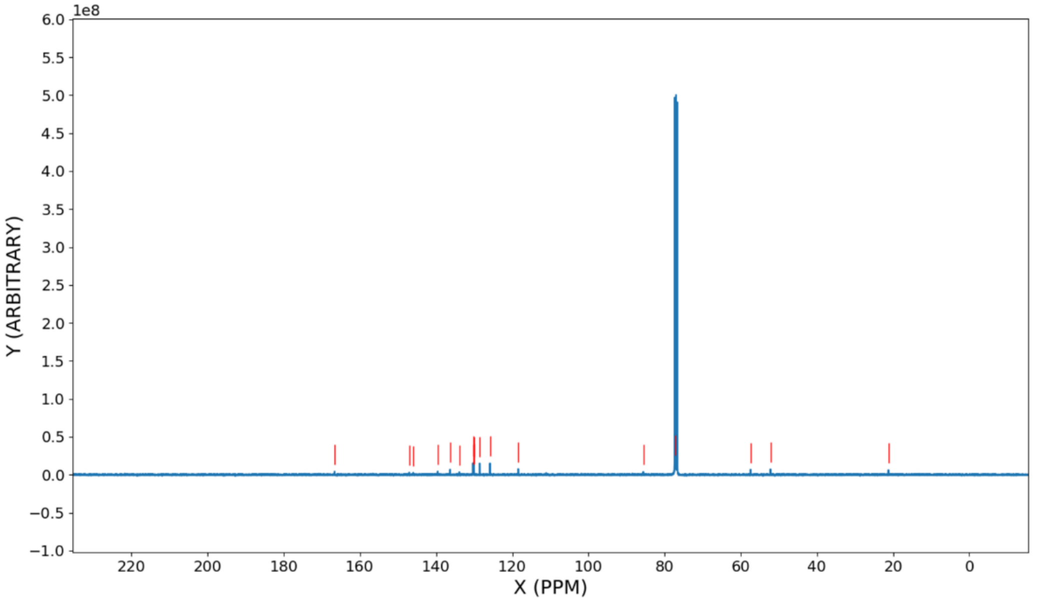

[**21tg**] 3-(4-(4-(dimethylamino)phenyl)-1*H*-1,2,3-triazol-1-yl)-1-(4-methylbenzyl)-1*H*-pyrazole-4-carbonitrile

CHMO:0000593 | ^{1}H nuclear magnetic resonance spectroscopy (^{1}H NMR)

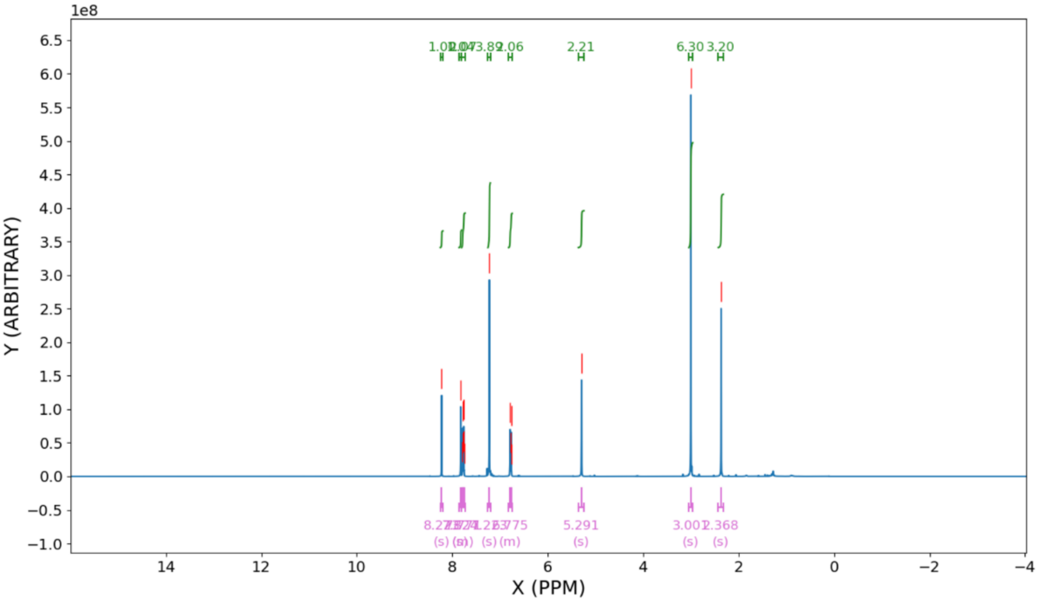

CHMO:0000595 | ^{13}C nuclear magnetic resonance spectroscopy (^{13}C NMR)

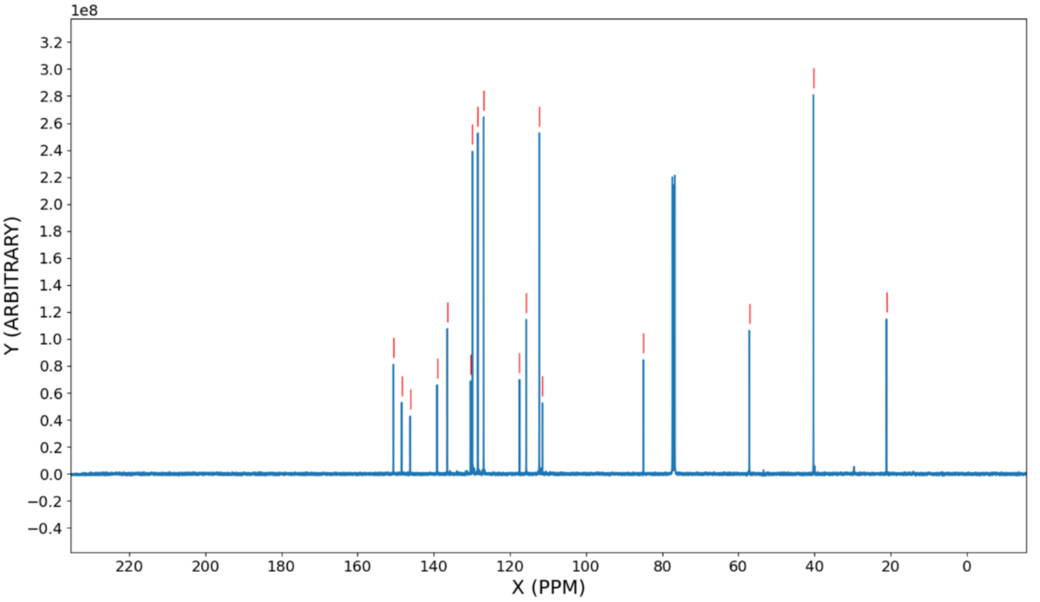

[**21th**] 3-(4-((1,3-dioxoisoindolin-2-yl)methyl)-1*H*-1,2,3-triazol-1-yl)-1-(4-methylbenzyl)-1*H*-pyrazole-4-carbonitrile

CHMO:0000593 | ^{1}H nuclear magnetic resonance spectroscopy (^{1}H NMR)

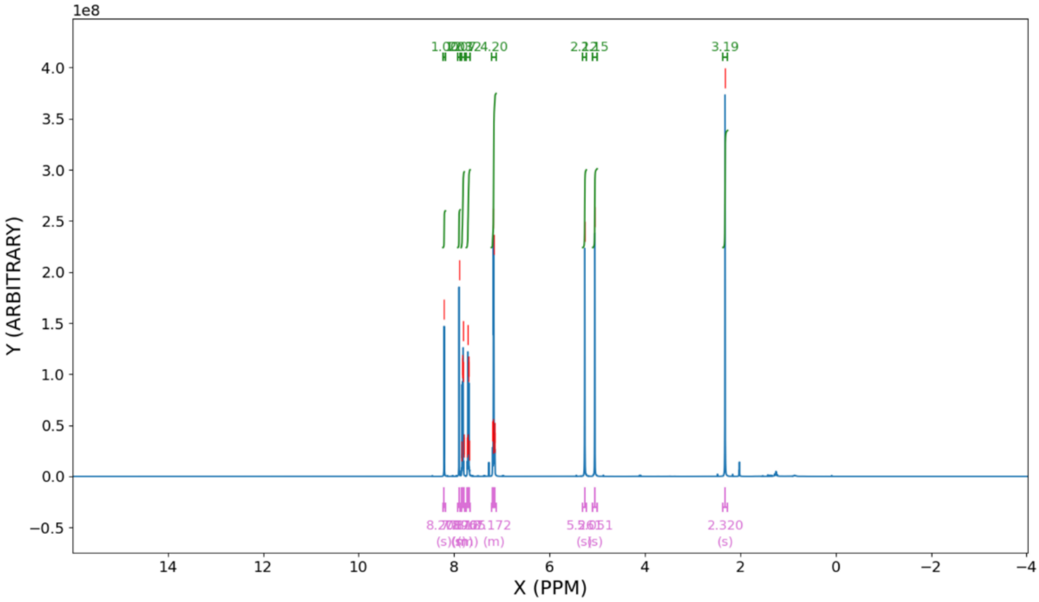

CHMO:0000595 | ^{13}C nuclear magnetic resonance spectroscopy (^{13}C NMR)

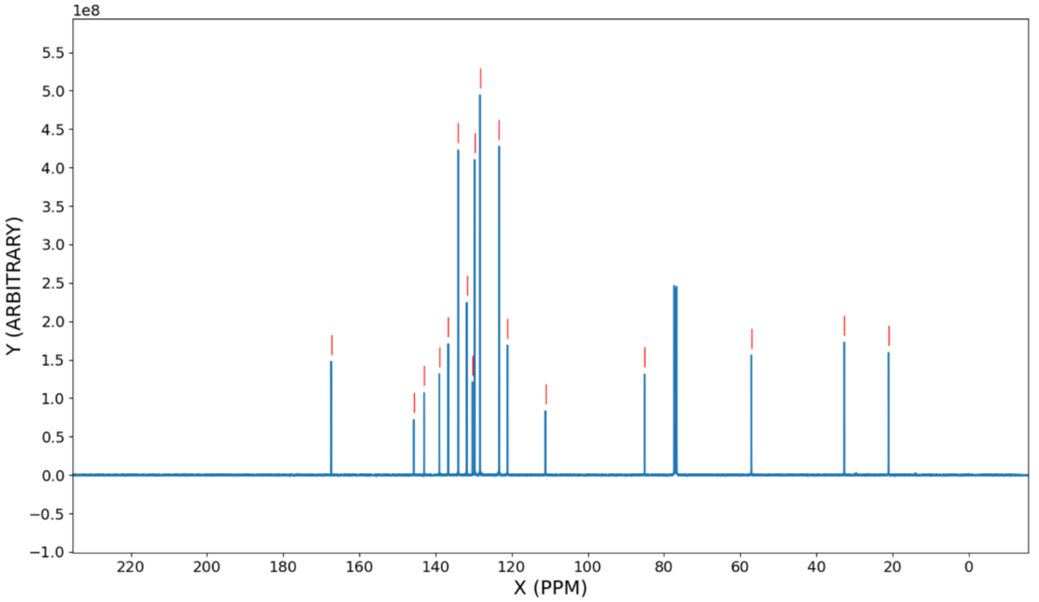

[**21ud**] methyl 4-((4-cyano-3-(4-phenyl-1*H*-1,2,3-triazol-1-yl)-1*H*-pyrazol-1-yl)methyl)benzoate

CHMO:0000593 | ^{1}H nuclear magnetic resonance spectroscopy (^{1}H NMR)

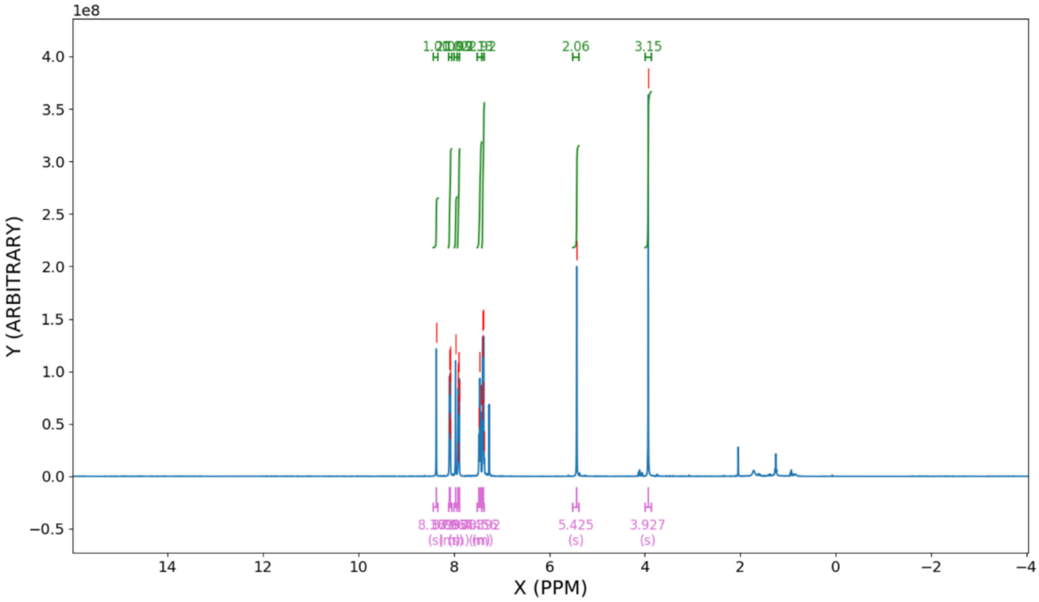

CHMO:0000595 | ^{13}C nuclear magnetic resonance spectroscopy (^{13}C NMR)

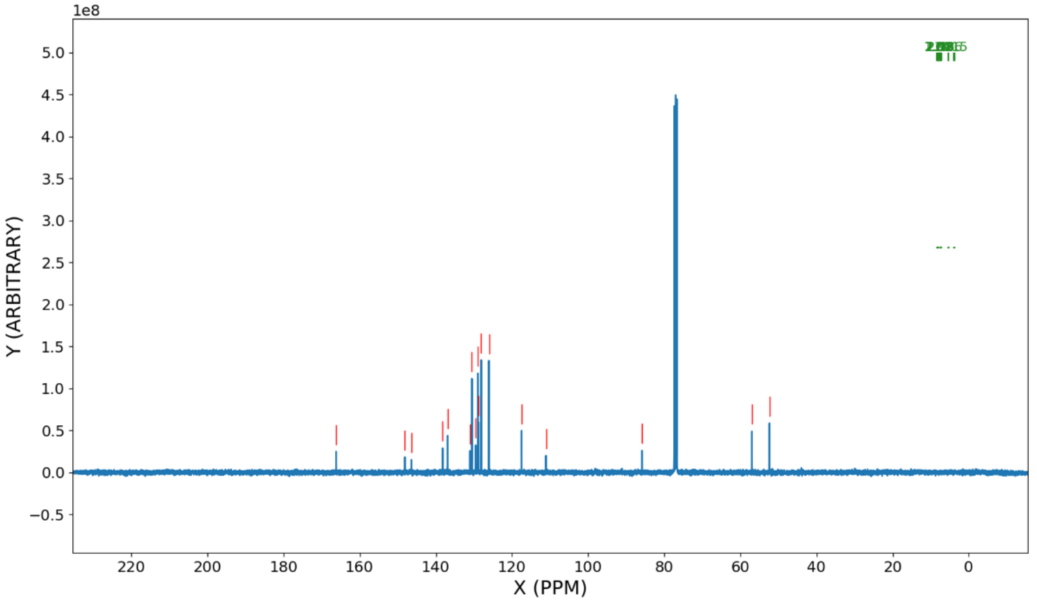

[**21vb**] methyl 1-(4-cyano-1-(4-fluorobenzyl)-1*H*-pyrazol-3-yl)-1*H*-1,2,3-triazole-4-carboxylate

CHMO:0000593 | ^{1}H nuclear magnetic resonance spectroscopy (^{1}H NMR)

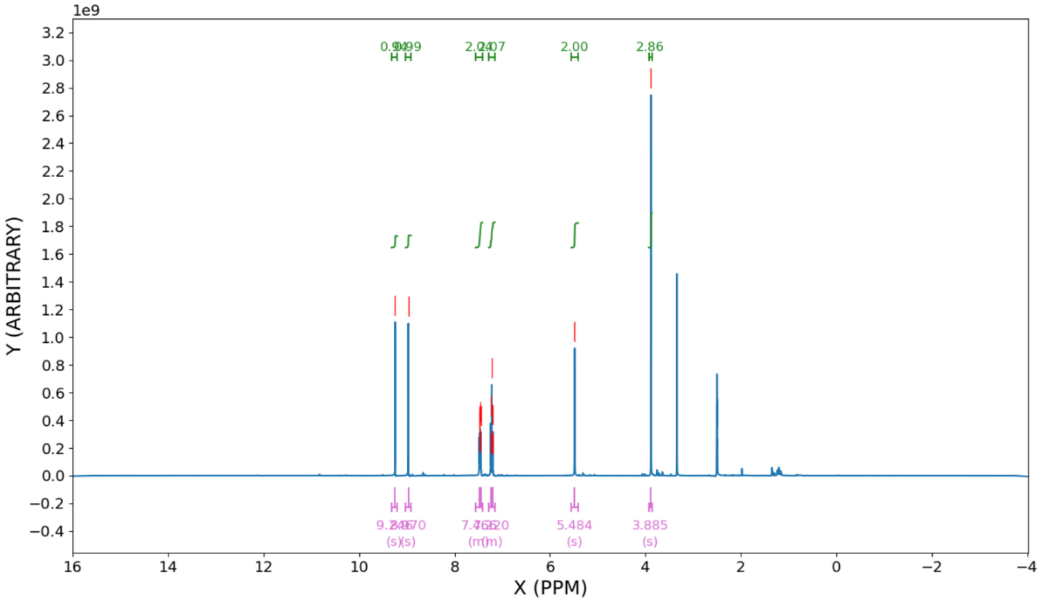

CHMO:0000595 | ^{13}C nuclear magnetic resonance spectroscopy (^{13}C NMR)

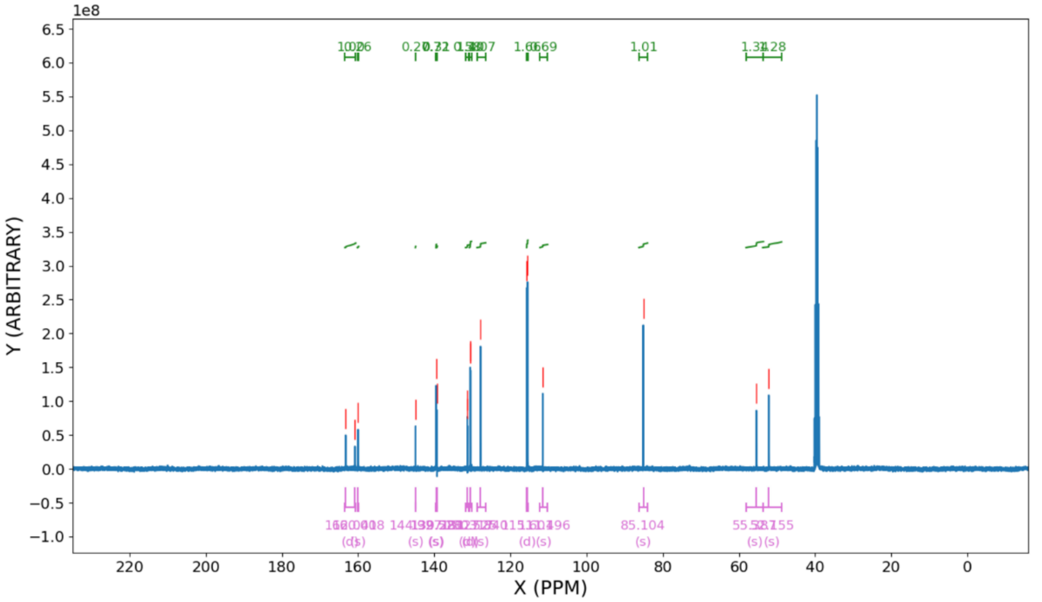

[**21vg**] 3-(4-(4-(dimethylamino)phenyl)-1*H*-1,2,3-triazol-1-yl)-1-(4-fluorobenzyl)-1*H*-pyrazole-4-carbonitrile

CHMO:0000593 | ^{1}H nuclear magnetic resonance spectroscopy (^{1}H NMR)

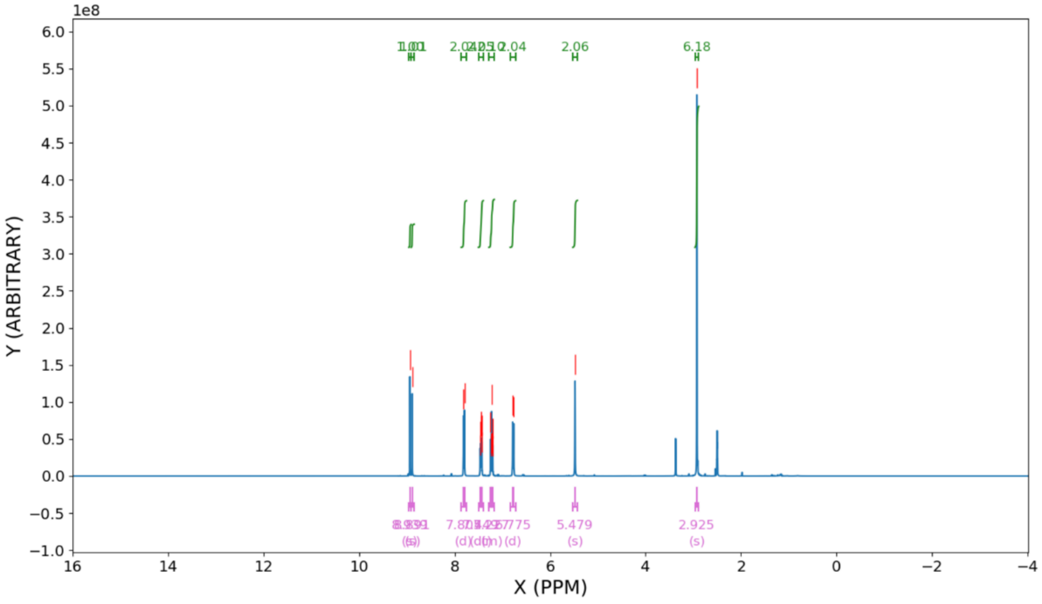

CHMO:0000595 | ^{13}C nuclear magnetic resonance spectroscopy (^{13}C NMR)

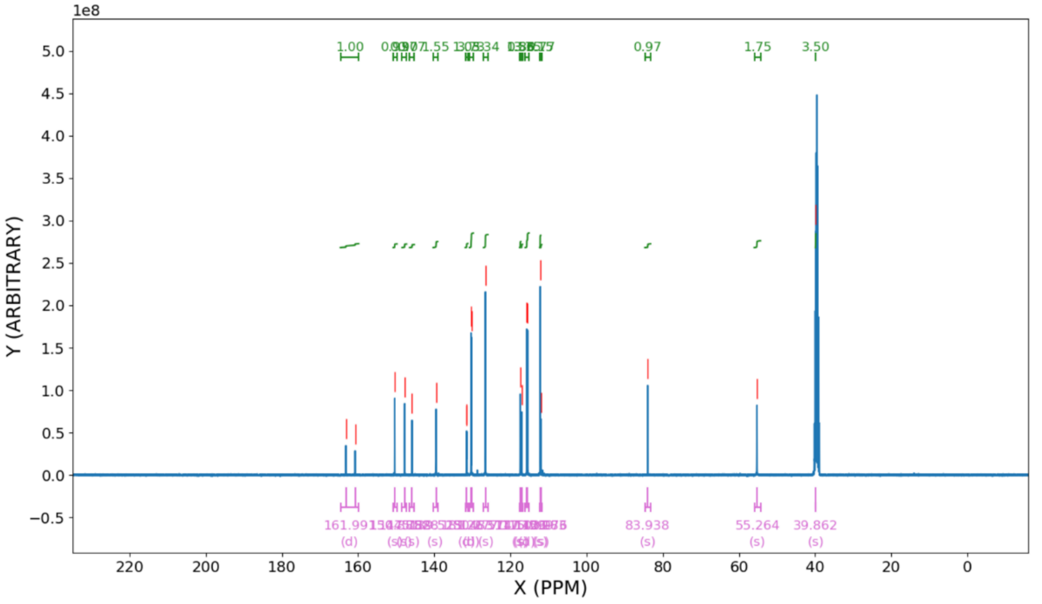

[**S4**] ethyl 1-(4-methoxyphenyl)-3-(4-phenyl-1H-1,2,3-triazol-1-yl)-1H-pyrazole-4-carboxylate

CHMO:0000593 | ^{1}H nuclear magnetic resonance spectroscopy (^{1}H NMR)

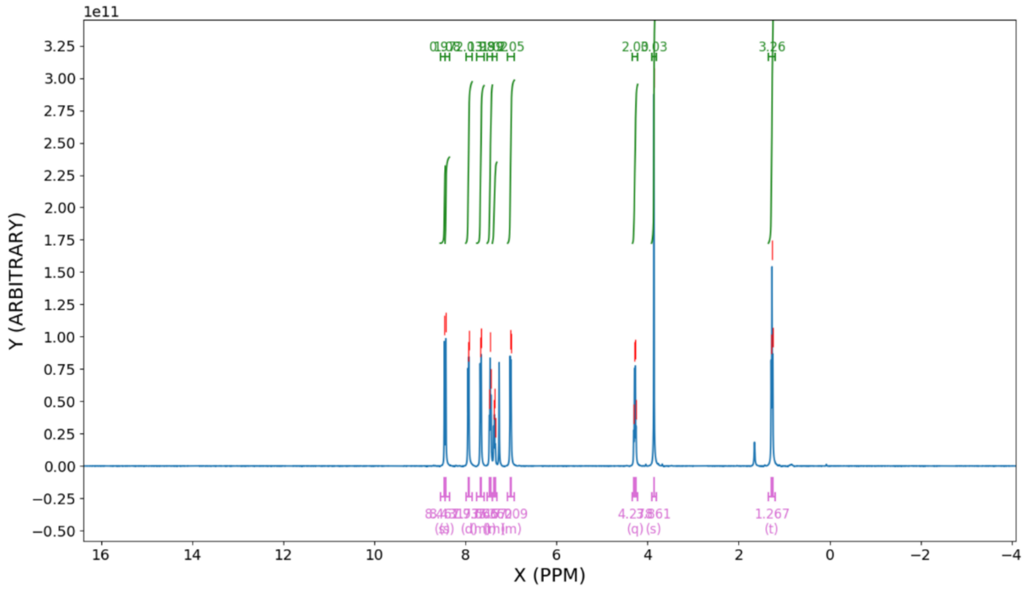

CHMO:0000595 | ^{13}C nuclear magnetic resonance spectroscopy (^{13}C NMR)

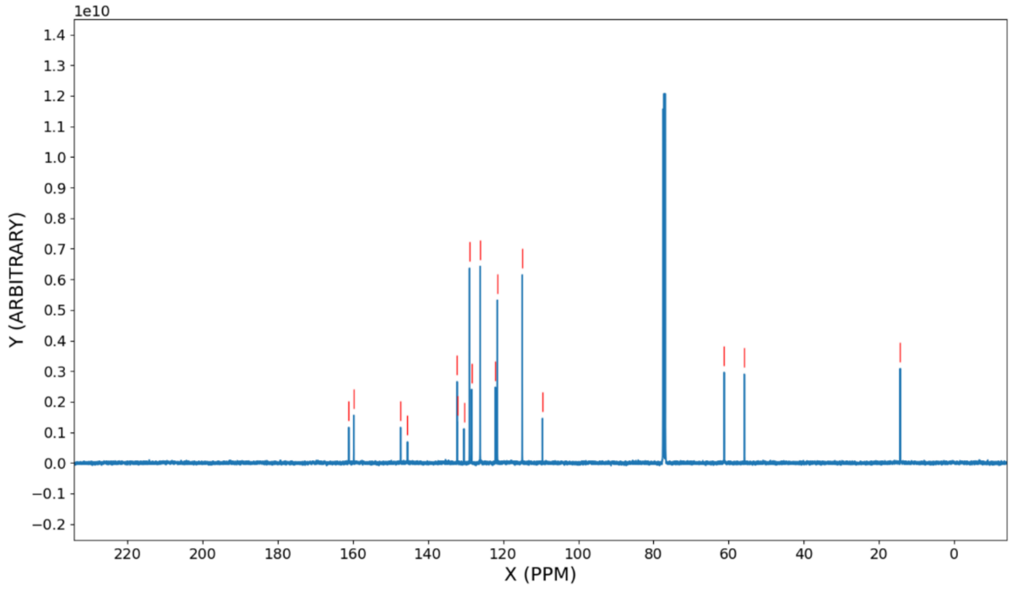

Supplement: File 2 — NMR spectra. [file Beilstein_J_Org_Chem-20-1396-s002.pdf]
