# Supplementary material for: Synthesis of substituted triazole–pyrazole hybrids using triazenylpyrazole precursors
Source: Beilstein J Org Chem. 2024 Jun 20;20:1396–404. doi: 10.3762/bjoc.20.121 (PMC11196952; doi:10.3762/bjoc.20.121)
Supplement: File 3 — Information on the availability of the data and the physical material of the target compounds. [file Beilstein_J_Org_Chem-20-1396-s003.pdf]

BEILSTEIN JOURNAL OF ORGANIC CHEMISTRY

Supporting Information

for

Synthesis of substituted triazole–pyrazole hybrids using triazenylpyrazole precursors

Simone Gräßle, Laura Holzhauer, Nicolai Wippert, Olaf Fuhr, Martin Nieger, Nicole Jung and Stefan Bräse

Beilstein J. Org. Chem. **2024,** *20,* 1396–1404. doi:10.3762/bjoc.20.121

Information on the availability of the data and the physical material of the target compounds

License and Terms: This is a supporting information file under the terms of the Creative Commons Attribution License (https://creativecommons.org/licenses/by/4.0). Please note that the reuse, redistribution and reproduction in particular requires that the author(s) and source are credited and that individual graphics may be subject to special legal provisions.
The license is subject to the *Beilstein Journal of Organic Chemistry* terms and conditions: (https://www.beilstein-journals.org/bjoc/terms)

General information: The following list includes the materials produced in this study which were made available through the Molecule Archive at KIT (https://compound-platform.eu/). The archive stores the materials for further reuse and review – interested academic scientists can order materials for different scientific collaborations or as reference compounds. The materials can be found via the DOI link given below or can be accessed through the Molecule Archive list in the Chemotion repository: https://chemotion-repository.net/home/moleculeArchive

Compound	Smiles Code	Sample DOI
**19g**	[N-]=[N+]=Nc1nn(c(c1)C)c1ccc(cc1)C#N	https://dx.doi.org/10.14272/KIQBPEXSAZKVRR-UHFFFAOYSA-N.3
**17e**	N#Cc1ccc(cc1)n1nc(cc1C)/N=N/N(C(C)C)C(C)C	https://dx.doi.org/10.14272/RSDVJJOPRTZAOO-XUTLUUPISA-N.1
**18e**	N#Cc1ccc(cc1)n1nc(cc1/N=N/N(C(C)C)C(C)C)C	https://dx.doi.org/10.14272/WPXPVVIOPAJXSL-XUTLUUPISA-N.1
**17f**	CC(N(C(C)C)/N=N/c1nn(c(c1)C)c1ccc(cc1)[N+](=O)[O-])C	https://dx.doi.org/10.14272/LQZXCDPUXUEILJ-HTXNQAPBSA-N.1
**18f**	CC(N(C(C)C)/N=N/c1cc(nn1c1ccc(cc1)[N+](=O)[O-])C)C	https://dx.doi.org/10.14272/VRKHTXPHTAWWEY-HTXNQAPBSA-N.1

**17k**	CCOC(=O)c1cn(nc1/N=N/N(C(C)C)C(C)C)Cc1ccc(cc1)Br	https://dx.doi.org/10.14272/ZYMNXIBCZDWSHX-XTQSDGFTSA-N.1
**18k**	CCOC(=O)c1cnn(c1/N=N/N(C(C)C)C(C)C)Cc1ccc(cc1)Br	https://dx.doi.org/10.14272/FFWKUULMXDRWPI-GHVJWSGMSA-N.1
**17d**	CC(N(C(C)C)/N=N/c1nn(c(c1)C)Cc1ccccc1)C	https://dx.doi.org/10.14272/FEHNUWIZIPJQLZ-CZIZESTLSA-N.1
**18d**	CC(N(C(C)C)/N=N/c1cc(nn1Cc1ccccc1)C)C	https://dx.doi.org/10.14272/OSZGZNAJFDDFLV-CZIZESTLSA-N.1
**17r**	N#Cc1cn(nc1/N=N/N(C(C)C)C(C)C)Cc1ccc(cc1)F	https://dx.doi.org/10.14272/ADGUCVKMBUJWJM-LSDHQDQOSA-N.1
**18r**	N#Cc1cnn(c1/N=N/N(C(C)C)C(C)C)Cc1ccc(cc1)F	https://dx.doi.org/10.14272/HEZWXNUXEKJBQT-QURGRASLSA-N.1
**21sd**	N#Cc1cn(nc1n1nnc(c1)c1ccccc1)Cc1cc(F)cc(c1)F	https://dx.doi.org/10.14272/LBYPTGDZZSOCSK-UHFFFAOYSA-N.1
**21ud**	COC(=O)c1ccc(cc1)Cn1cc(c(n1)n1nnc(c1)c1ccccc1)C#N	https://dx.doi.org/10.14272/YZSLXIHSIJQQMB-UHFFFAOYSA-N.1
**21ga**	CCCCc1nnn(c1)c1nn(c(c1)C)c1ccc(cc1)C#N	https://dx.doi.org/10.14272/WZTARLNNZGQBDW-UHFFFAOYSA-N.1
**21rd**	N#Cc1cn(nc1n1nnc(c1)c1ccccc1)Cc1ccccc1	https://dx.doi.org/10.14272/DGWASOOTEXXJBK-UHFFFAOYSA-N.1
**21fa**	CCCCc1nnn(c1)c1cc(n(n1)Cc1ccccc1)C	https://dx.doi.org/10.14272/PKNOMBYYAWYDCG-UHFFFAOYSA-N.1
**21gd**	N#Cc1ccc(cc1)n1nc(cc1C)n1nnc(c1)c1ccccc1	https://dx.doi.org/10.14272/QJHTUPAVPUUSNY-UHFFFAOYSA-N.1<br>https://dx.doi.org/10.14272/QJHTUPAVPUUSNY-UHFFFAOYSA-N.2
**17q**	COC(=O)c1ccc(cc1)Cn1nc(c(c1)C#N)/N=N/N(C(C)C)C(C)C	https://dx.doi.org/10.14272/FZUUXBMFWLBSAE-XTQSDGFTSA-N.1
**18q**	COC(=O)c1ccc(cc1)Cn1ncc(c1/N=N/N(C(C)C)C(C)C)C#N	https://dx.doi.org/10.14272/HQEHAWORIRDNAT-GHVJWSGMSA-N.1
**21ce**	COc1ccc(cc1)c1nnn(c1)c1ccn(n1)Cc1ccccc1	https://dx.doi.org/10.14272/JMPSOWDQCFVJPL-UHFFFAOYSA-N.1
**19q**	CCOC(=O)Cn1nc(c(c1)C#N)N=[N+]=[N-]	https://dx.doi.org/10.14272/HEIIGJFYWAHNFX-UHFFFAOYSA-N.1
**17a**	CC(N(C(C)C)/N=N/c1ccn(n1)Cc1ccccc1)C	https://dx.doi.org/10.14272/MGAVNYXVFXWSSZ-HTXNQAPBSA-N.1
**18a**	CC(N(C(C)C)/N=N/c1ccnn1Cc1ccccc1)C	https://dx.doi.org/10.14272/ZIDRUQYCDSWWJM-VHEBQXMUSA-N.1
**21ic**	CC(=O)OCc1nnn(c1)c1nn(c(c1)C)c1ccc(cc1Cl)[N+](=O)[O-]	https://dx.doi.org/10.14272/QIRBWRVTFHDFHS-UHFFFAOYSA-N.1
**17h**	CCOC(=O)c1cn(nc1/N=N/N(C(C)C)C(C)C)Cc1ccccc1	https://dx.doi.org/10.14272/GKODORMDVGUGHL-LSDHQDQOSA-N.1
**18h**	CCOC(=O)c1cnn(c1/N=N/N(C(C)C)C(C)C)Cc1ccccc1	https://dx.doi.org/10.14272/UBBFWZTUXPGXCP-QURGRASLSA-N.1
**17l**	N#Cc1cn(nc1/N=N/N(C(C)C)C(C)C)C(C)C	https://dx.doi.org/10.14272/XUZXRALOPOUFGP-BMRADRMJSA-N.1
**18l**	N#Cc1cnn(c1/N=N/N(C(C)C)C(C)C)C(C)C	https://dx.doi.org/10.14272/IJEPRRMQECALCS-WUKNDPDISA-N.1
**21fe**	COc1ccc(cc1)c1nnn(c1)c1nn(c(c1)C)Cc1ccccc1	https://dx.doi.org/10.14272/HKYHACNKJZVDCN-UHFFFAOYSA-N.1

**17n**	N#Cc1cn(nc1/N=N/N(C(C)C)C(C)C)Cc1ccccc1	https://dx.doi.org/10.14272/AONLLYWWGOIMLR-XUTLUUPISA-N.2
**18n**	CC(N(C(C)C)/N=N/c1n(ncc1C#N)Cc1ccccc1)C	https://dx.doi.org/10.14272/AQYSAXXLCHFEGV-QZQOTICOSA-N.2
**15b**	CC(N(C(C)C)/N=N/c1n[nH]c(c1)C)C	https://dx.doi.org/10.14272/SSAXAAXODLOITE-BUHFOSPRSA-N.1
**19c**	[N-]=[N+]=Nc1ccn(n1)Cc1ccccc1	https://dx.doi.org/10.14272/VYAIIDYJDVIYCE-UHFFFAOYSA-N.1
**19m**	CCOC(=O)c1cn(nc1N=[N+]=[N-])Cc1cccc(c1)C#N	https://dx.doi.org/10.14272/OZCFNQRRYBYULQ-UHFFFAOYSA-N.1
**21hd**	[O-][N+](=O)c1ccc(cc1)n1nc(cc1C)n1nnc(c1)c1ccccc1	https://dx.doi.org/10.14272/UFCSIROIFJMCPW-UHFFFAOYSA-N.1
**21vb**	COC(=O)c1nnn(c1)c1nn(cc1C#N)Cc1ccc(cc1)F	https://dx.doi.org/10.14272/UUNQVGVZWMJXOZ-UHFFFAOYSA-N.1
**19i**	[N-]=[N+]=Nc1nn(c(c1)C)c1ccc(cc1Cl)[N+](=O)[O-]	https://dx.doi.org/10.14272/GQMYHPXVWDUZCD-UHFFFAOYSA-N.1
**21tf**	COC(=O)c1ccc(cc1)c1nnn(c1)c1nn(cc1C#N)Cc1ccc(cc1)C	https://dx.doi.org/10.14272/DMRAVMAMSLYEBQ-UHFFFAOYSA-N.1
**21ed**	Cc1[nH]nc(c1)n1nnc(c1)c1ccccc1	https://dx.doi.org/10.14272/BTYPCTNIHDBBSH-UHFFFAOYSA-N.1
**21id**	Clc1cc(ccc1n1nc(cc1C)n1nnc(c1)c1ccccc1)[N+](=O)[O-]	https://dx.doi.org/10.14272/NASFABBFAHTRIZ-UHFFFAOYSA-N.1
**21nd**	CCOC(=O)c1cn(nc1n1nnc(c1)c1ccccc1)Cc1ccc(cc1)Br	https://dx.doi.org/10.14272/VTYPYIREEKHMKL-UHFFFAOYSA-N.1
**21na**	CCCCc1nnn(c1)c1nn(cc1C(=O)OCC)Cc1ccc(cc1)Br	https://dx.doi.org/10.14272/YERIOOVHZOGENU-UHFFFAOYSA-N.1
**21nc**	CCOC(=O)c1cn(nc1n1nnc(c1)COC(=O)C)Cc1ccc(cc1)Br	https://dx.doi.org/10.14272/JIGNGCLNWAMKPG-UHFFFAOYSA-N.1
**21me**	CCOC(=O)c1cn(nc1n1nnc(c1)c1ccc(cc1)OC)Cc1cccc(c1)C#N	https://dx.doi.org/10.14272/KJMWCYALHNEPDX-UHFFFAOYSA-N.1
**21mh**	CCOC(=O)c1cn(nc1n1nnc(c1)CN1C(=O)c2c(C1=O)cccc2)Cc1cccc(c1)C#N	https://dx.doi.org/10.14272/RJXQILXRDFYRBG-UHFFFAOYSA-N.1
**21lc**	CCOC(=O)c1cn(nc1n1nnc(c1)COC(=O)C)Cc1cc(F)cc(c1)F	https://dx.doi.org/10.14272/OEMVMGJIOKDNBC-UHFFFAOYSA-N.1
**19t**	[N-]=[N+]=Nc1nn(cc1C#N)Cc1ccc(cc1)C	https://dx.doi.org/10.14272/NMQYIKIIUJYZLG-UHFFFAOYSA-N.1
**19n**	CCOC(=O)c1cn(nc1N=[N+]=[N-])Cc1ccc(cc1)Br	https://dx.doi.org/10.14272/XWYLZJSXZASHCL-UHFFFAOYSA-N.1
**21hc**	CC(=O)OCc1nnn(c1)c1nn(c(c1)C)c1ccc(cc1)[N+](=O)[O-]	https://dx.doi.org/10.14272/GFUNAXVZSJSISA-UHFFFAOYSA-N.1
**21ia**	CCCCc1nnn(c1)c1nn(c(c1)C)c1ccc(cc1Cl)[N+](=O)[O-]	https://dx.doi.org/10.14272/JCVTZTZCOPFHML-UHFFFAOYSA-N.1
**21ta**	CCCCc1nnn(c1)c1nn(cc1C#N)Cc1ccc(cc1)C	https://dx.doi.org/10.14272/FGLOKNGZLSDWMZ-UHFFFAOYSA-N.1
**21tg**	N#Cc1cn(nc1n1nnc(c1)c1ccc(cc1)N(C)C)Cc1ccc(cc1)C	https://dx.doi.org/10.14272/PIHYCVSEWQEZEE-UHFFFAOYSA-N.1
**21pd**	N#Cc1cn(nc1n1nnc(c1)c1ccccc1)C(C)C	https://dx.doi.org/10.14272/OYRDJONMMXESTQ-UHFFFAOYSA-N.1

**21pa**	CCCCc1nnn(c1)c1nn(cc1C#N)C(C)C	https://dx.doi.org/10.14272/NOJZURFWHMPYCF-UHFFFAOYSA-N.1
**17j**	CCOC(=O)c1cn(nc1/N=N/N(C(C)C)C(C)C)Cc1cccc(c1)C#N	https://dx.doi.org/10.14272/NUENLEYWZUNRJY-ZNTNEXAZSA-N.1
**18j**	CCOC(=O)c1cnn(c1/N=N/N(C(C)C)C(C)C)Cc1cccc(c1)C#N	https://dx.doi.org/10.14272/WTPBPRNMORPMPY-WCWDXBQESA-N.1
**17g**	CC(N(C(C)C)/N=N/c1nn(c(c1)C)c1ccc(cc1Cl)[N+](=O)[O-])C	https://dx.doi.org/10.14272/TYZBFJHLBYOAAU-CZIZESTLSA-N.1
**18g**	CC(N(C(C)C)/N=N/c1cc(nn1c1ccc(cc1Cl)[N+](=O)[O-])C)C	https://dx.doi.org/10.14272/ACQCSRYTLZFICQ-CZIZESTLSA-N.1
**21ea**	CCCCc1nnn(c1)c1n[nH]c(c1)C	https://dx.doi.org/10.14272/QKYKTOGQTOIHKO-UHFFFAOYSA-N.1
**21eb**	COC(=O)c1nnn(c1)c1n[nH]c(c1)C	https://dx.doi.org/10.14272/AXYCMAQNQSYSIG-UHFFFAOYSA-N.1
**21vg**	N#Cc1cn(nc1n1nnc(c1)c1ccc(cc1)N(C)C)Cc1ccc(cc1)F	https://dx.doi.org/10.14272/UIXMTYDHDCHVFR-UHFFFAOYSA-N.1
**19f**	[N-]=[N+]=Nc1nn(c(c1)C)Cc1ccccc1	https://dx.doi.org/10.14272/CUZSOFWTCLMWBL-UHFFFAOYSA-N.1
**17m**	CCOC(=O)Cn1nc(c(c1)C#N)/N=N/N(C(C)C)C(C)C	https://dx.doi.org/10.14272/GMSYXQOQYOUAHU-FBMGVBCBSA-N.2
**18m**	CCOC(=O)Cn1ncc(c1/N=N/N(C(C)C)C(C)C)C#N	https://dx.doi.org/10.14272/UVXRQPKXJULFTR-ISLYRVAYSA-N.2
**17b**	CC(N(C(C)C)/N=N/c1ccn(n1)Cc1ccc(cc1)Br)C	https://dx.doi.org/10.14272/OJEKMYNEGYNUPP-CZIZESTLSA-N.1
**18b**	Brc1ccc(cc1)Cn1nccc1/N=N/N(C(C)C)C(C)C	https://dx.doi.org/10.14272/PLVZHOAUEJAYPS-FMQUCBEESA-N.1
**17i**	CCOC(=O)c1cn(nc1/N=N/N(C(C)C)C(C)C)Cc1cc(F)cc(c1)F	https://dx.doi.org/10.14272/HJNVDSRILWJTQV-ZNTNEXAZSA-N.1
**18i**	CCOC(=O)c1cnn(c1/N=N/N(C(C)C)C(C)C)Cc1cc(F)cc(c1)F	https://dx.doi.org/10.14272/XCKSGKZXEXJBOY-WCWDXBQESA-N.1
**17o**	N#Cc1cn(nc1/N=N/N(C(C)C)C(C)C)Cc1cc(F)cc(c1)F	https://dx.doi.org/10.14272/WSQPCVJPBSUDNZ-XTQSDGFTSA-N.2
**18o**	CC(N(C(C)C)/N=N/c1n(ncc1C#N)Cc1cc(F)cc(c1)F)C	https://dx.doi.org/10.14272/ZFEWCQZPKGQXCJ-GHVJWSGMSA-N.2
**21jh**	CCOC(=O)c1c[nH]nc1n1nnc(c1)CN1C(=O)c2c(C1=O)cccc2	https://dx.doi.org/10.14272/GMOYQWJQIYZFIE-UHFFFAOYSA-N.1
**21th**	N#Cc1cn(nc1n1nnc(c1)CN1C(=O)c2c(C1=O)cccc2)Cc1ccc(cc1)C	https://dx.doi.org/10.14272/FODYZQGXSSTMHJ-UHFFFAOYSA-N.1
**21oa**	CCCCc1nnn(c1)c1n[nH]cc1C#N	https://dx.doi.org/10.14272/USLFBJCHBNQGJB-UHFFFAOYSA-N.1
**21he**	COc1ccc(cc1)c1nnn(c1)c1cc(n(n1)c1ccc(cc1)[N+](=O)[O-])C	https://dx.doi.org/10.14272/IZEQOYLPBRFYRC-UHFFFAOYSA-N.1
**21jc**	CCOC(=O)c1c[nH]nc1n1nnc(c1)COC(=O)C	https://dx.doi.org/10.14272/RJHHGQMRVYAAGU-UHFFFAOYSA-N.1
**21of**	COC(=O)c1ccc(cc1)c1nnn(c1)c1n[nH]cc1C#N	https://dx.doi.org/10.14272/FXIYGLHLKLOXQB-UHFFFAOYSA-N.1
**19s**	[N-]=[N+]=Nc1nn(cc1C#N)Cc1cc(F)cc(c1)F	https://dx.doi.org/10.14272/WBMURKUEYYRUPK-UHFFFAOYSA-N.1
**19l**	CCOC(=O)c1cn(nc1N=[N+]=[N-])Cc1cc(F)cc(c1)F	https://dx.doi.org/10.14272/BZRWHBSLSABJOL-UHFFFAOYSA-N.1

**21jd**	CCOC(=O)c1c[nH]nc1n1nnc(c1)c1ccccc1	https://dx.doi.org/10.14272/JZSBUPSAIWVYFE-UHFFFAOYSA-N.2
**19e**	[N-]=[N+]=Nc1cc([nH]n1)C	https://dx.doi.org/10.14272/ASYITVIZQLPDSI-UHFFFAOYSA-N.1
**21ab**	COC(=O)c1nnn(c1)c1n[nH]cc1	https://dx.doi.org/10.14272/PWKBPRHBEDYORH-UHFFFAOYSA-N.1
**21bb**	COC(=O)c1nnn(c1)c1ccn(n1)C1CCCC1	https://dx.doi.org/10.14272/RZTMDUAYRSTLAQ-UHFFFAOYSA-N.1
**21ae**	COc1ccc(cc1)c1nnn(c1)c1n[nH]cc1	https://dx.doi.org/10.14272/PQOYNAQPLQYWLU-UHFFFAOYSA-N.1
**21aa**	CCCCc1cn(nn1)c1cc[nH]n1	https://dx.doi.org/10.14272/NLSVJZHXBPJQPM-UHFFFAOYSA-N.1
**21ef**	COC(=O)c1ccc(cc1)c1nnn(c1)c1n[nH]c(c1)C	https://dx.doi.org/10.14272/ICPVQAYXOPZIKF-UHFFFAOYSA-N.1
**19b**	[N-]=[N+]=Nc1ccn(n1)C1CCCC1	https://dx.doi.org/10.14272/CEJMUQXTWWQJBT-UHFFFAOYSA-N.2
**19d**	[N-]=[N+]=Nc1ccn(n1)Cc1ccc(cc1)Br	https://dx.doi.org/10.14272/MPLVWOQSFNIWDU-UHFFFAOYSA-N.2
**15a**	CC(N(C(C)C)/N=N/c1n[nH]cc1)C	https://dx.doi.org/10.14272/LKGAYQXGDXKVDA-OUKQBFOZSA-N.1
**21sa**	CCCCc1nnn(c1)c1nn(cc1C#N)Cc1cc(F)cc(c1)F	https://dx.doi.org/10.14272/APGSEIDPRRBFBQ-UHFFFAOYSA-N.1
**21ec**	CC(=O)OCc1nnn(c1)c1n[nH]c(c1)C	https://dx.doi.org/10.14272/LUTGSYAMTQUJGA-UHFFFAOYSA-N.1
**21od**	N#Cc1c[nH]nc1n1nnc(c1)c1ccccc1	https://dx.doi.org/10.14272/VSYUPFQKDFVQCM-UHFFFAOYSA-N.1
**21be**	COc1ccc(cc1)c1nnn(c1)c1ccn(n1)C1CCCC1	https://dx.doi.org/10.14272/SYHGOJNCDVCRGL-UHFFFAOYSA-N.1
**21qa**	CCCCc1nnn(c1)c1nn(cc1C#N)CC(=O)OCC	https://dx.doi.org/10.14272/ZOQWDAUGOCUFLP-UHFFFAOYSA-N.1
**21ja**	CCCCc1nnn(c1)c1n[nH]cc1C(=O)OCC	https://dx.doi.org/10.14272/CDDXWUAJGLDUKH-UHFFFAOYSA-N.1
**21de**	COc1ccc(cc1)c1nnn(c1)c1ccn(n1)Cc1ccc(cc1)Br	https://dx.doi.org/10.14272/QXNOXDRDOZJZFO-UHFFFAOYSA-N.1
**21db**	COC(=O)c1nnn(c1)c1ccn(n1)Cc1ccc(cc1)Br	https://dx.doi.org/10.14272/CNHRLRYEDLASIW-UHFFFAOYSA-N.1
**21lf**	CCOC(=O)c1cn(nc1n1nnc(c1)c1ccc(cc1)C(=O)OC)Cc1cc(F)cc(c1)F	https://dx.doi.org/10.14272/WJPCYFBJDHGFTN-UHFFFAOYSA-N.1
**21oc**	N#Cc1c[nH]nc1n1nnc(c1)COC(=O)C	https://dx.doi.org/10.14272/QROBBVJEUOFHFJ-UHFFFAOYSA-N.1
**17c**	CC(N(C(C)C)/N=N/c1ccn(n1)C1CCCC1)C	https://dx.doi.org/10.14272/JJSUJDRUQGTCKX-BMRADRMJSA-N.1
**18c**	CC(N(C(C)C)/N=N/c1ccnn1C1CCCC1)C	https://dx.doi.org/10.14272/BWAGQJOINMBVOL-WUKNDPDISA-N.1
**21kd**	CCOC(=O)c1cn(nc1n1nnc(c1)c1ccccc1)Cc1ccccc1	https://dx.doi.org/10.14272/BFHMQIVAONQIIQ-UHFFFAOYSA-N.1
**19g**	[N-]=[N+]=Nc1nn(c(c1)C)c1ccc(cc1)C#N	https://dx.doi.org/10.14272/KIQBPEXSAZKVRR-UHFFFAOYSA-N.1
**21td**	N#Cc1cn(nc1n1nnc(c1)c1ccccc1)Cc1ccc(cc1)C	https://dx.doi.org/10.14272/ZHUFVXOCSYMTKK-UHFFFAOYSA-N.1

**21gh**	N#Cc1ccc(cc1)n1nc(cc1C)n1nnc(c1)CN1C(=O)c2c(C1=O)cccc2	https://dx.doi.org/10.14272/DWYBPGPEFGKMQS-UHFFFAOYSA-N.1
**21ee**	COc1ccc(cc1)c1nnn(c1)c1n[nH]c(c1)C	https://dx.doi.org/10.14272/WNRIJNCEJHFAPB-UHFFFAOYSA-N.2
**19o**	[N-]=[N+]=Nc1n[nH]cc1C#N	https://dx.doi.org/10.14272/FMLLTFUXLLAWNL-UHFFFAOYSA-N.1
**19a**	[N-]=[N+]=Nc1cc[nH]n1	https://dx.doi.org/10.14272/KKKGXLQRKPKJON-UHFFFAOYSA-N.1
**19p**	[N-]=[N+]=Nc1nn(cc1C#N)C(C)C	https://dx.doi.org/10.14272/XHQDUCMTSCUCRX-UHFFFAOYSA-N.1
**19r**	[N-]=[N+]=Nc1nn(cc1C#N)Cc1ccccc1	https://dx.doi.org/10.14272/BZGHBVIXEDKDEK-UHFFFAOYSA-N.1
**19k**	CCOC(=O)c1cn(nc1N=[N+]=[N-])Cc1ccccc1	https://dx.doi.org/10.14272/KCAXRYJMUFAPLD-UHFFFAOYSA-N.1
**19v**	[N-]=[N+]=Nc1nn(cc1C#N)Cc1ccc(cc1)F	https://dx.doi.org/10.14272/KJPMFOGUNJZONN-UHFFFAOYSA-N.1
**19h**	[N-]=[N+]=Nc1nn(c(c1)C)c1ccc(cc1)[N+](=O)[O-]	https://dx.doi.org/10.14272/HISYWPICEGTOTQ-UHFFFAOYSA-N.1
**19d**	[N-]=[N+]=Nc1ccn(n1)Cc1ccc(cc1)Br	https://dx.doi.org/10.14272/MPLVWOQSFNIWDU-UHFFFAOYSA-N.1
**19j**	[N-]=[N+]=Nc1n[nH]cc1C(=O)OCC	https://dx.doi.org/10.14272/FSJHWJVEYJIYBP-UHFFFAOYSA-N.1
**15c**	CCOC(=O)c1c[nH]nc1/N=N/N(C(C)C)C(C)C	https://dx.doi.org/10.14272/XLGLVRGMYSSDFW-FOCLMDBBSA-N.1
